# Synthesis of Polysubstituted Ferrocenesulfoxides

**DOI:** 10.3390/molecules27061798

**Published:** 2022-03-09

**Authors:** Min Wen, William Erb, Florence Mongin, Yury S. Halauko, Oleg A. Ivashkevich, Vadim E. Matulis, Thierry Roisnel

**Affiliations:** 1 ISCR—Institut des Sciences Chimiques de Rennes-UMR CNRS 6226, Université de Rennes 1, F-35000 Rennes, France; min.wen@univ-rennes1.fr (M.W.); florence.mongin@univ-rennes1.fr (F.M.); thierry.roisnel@univ-rennes1.fr (T.R.); 2 UNESCO Chair of Belarusian State University, 4 Nezavisimosti Av., 220030 Minsk, Belarus; 3 Laboratory for Chemistry of Condensed Systems, Research Institute for Physical Chemical Problems of Belarusian State University, 14 Leningradskaya St., 220030 Minsk, Belarus; ivashkevicho@bsu.by; 4 Department of Inorganic Chemistry, Belarusian State University, 4 Nezavisimosti Av., 220030 Minsk, Belarus; matulisvad@bsu.by

**Keywords:** ferrocene, sulfoxide, deprotonative metalation, chiral directing group, CH acidity, stereoselectivity, one-pot synthesis

## Abstract

The purpose of the study is to design synthetic methodologies, especially directed deprotometalation using polar organometallic reagents, to access polysubstituted ferrocenesulfoxides. From enantiopure 2-substituted (SiMe_3_, PPh_2_) *S*-*tert*-butylferrocenesulfoxides, a third substituent was first introduced at the 5 position (SiMe_3_, I, D, C(OH)Ph_2_, Me, PPh_2_, CH_2_NMe_2_, F) and removal of the trimethylsilyl group then afforded 2-substituted ferrocenesulfoxides unreachable otherwise. Attempts to apply the “halogen dance” reaction to the ferrocenesulfoxide series led to unexpected results although rationalized in light of calculated p*K*_a_ values. Further functionalizations were also possible. Thus, new enantiopure, planar chiral di- and trisubstituted ferrocenes have been obtained, in addition to several original 2-substituted, 2,3- and 2,5-disubstituted, 2,3,5-trisubstituted and even 2,3,4,5-tetrasubstituted ferrocenesulfoxides, also enantiopure.

## 1. Introduction

During the last decades, the sulfoxide group (of general formula RSOR’) has attracted the interest of chemists because of its good accessibility and specific reactivity [1,2,3,4,5,6,7,8,9,10]. An additional feature of sulfoxides lies in their chirality (when R ≠ R’) which can be used to control the stereochemical outcome of many transformations [11]. This was well exploited in the synthesis of many natural products and pharmaceutical agents [12,13], and significant developments of sulfoxides as chiral ligands for asymmetric catalysis also deserve to be mentioned [14,15,16,17,18,19].

Since the discovery of their parent compound in 1952 [20,21], ferrocenes have established themselves as one of the most important families of organometallics. These three-dimensional compounds in which the iron is surrounded by two cyclopentadienyl rings are often stable to air, water, heat and light, and also exhibit a reversible redox behavior. Therefore, they can bring specific physical and chemical properties to the molecules in which they are included [22]. Thus, they have found many applications in fields such as catalysis [23,24,25], medicinal chemistry [26,27,28], and material science [29,30].

While the first ferrocene sulfoxide was reported in 1964 [31], the first stereopure members of this family were obtained 20 years later by the diastereoselective oxidation of sulfide derivatives of Ugi’s amine [32,33]. However, it was only from 1993 that the key methodologies to access enantiopure ferrocene sulfoxides and convert them by diastereoselective deprotolithiation were developed, in particular by Kagan and co-workers [34,35]. 4-Tolylsulfinyl [36,37,38] and *tert*-butylsulfinyl [39] have been mainly employed as directing groups for this purpose, and then transformed in various ways such as reduction to sulfides [36,40,41], oxidation to sulfones [42], sulfoxide/lithium exchange [36] followed by trapping [37,38], or simple removal [39].

**Figure 1:**
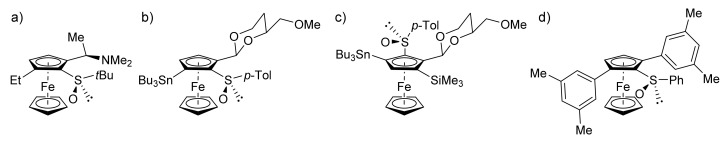
Polysubstituted ferrocenesulfoxides already reported by the groups of (**a**) Nicolosi, (**b**,**c**) Top and Jaouen, and (**d**) Tokitoh.

Through the years, these reactions allowed the synthesis of many 2-substituted ferrocenesulfoxides. However, examples of more substituted derivatives are scarce. In 2004, inspired by the work of Ugi’s group [32,33], Nicolosi and co-workers prepared a stereopure 2,5-disubstituted ferrocenesulfoxide by the diastereoselective oxidation of (*R*,*S*_P_)-2-(*tert*-butylthio)-α-(dimethylamino)ethylferrocene [33,43] followed by deprotolithiation and electrophilic trapping [39] (Figure 1a). In 2006, Top, Jaouen, and co-workers instead used Kagan’s acetal to access a stereopure 2,5-disubstituted ferrocenesulfoxide by successive introduction of sulfoxide and stannane functions [38] (Figure 1b). A trimethylsilyl group can also be employed to protect the privileged deprotonation site next to the acetal, and thus allow the introduction of sulfoxide and stannane functions on the other side [38] (Figure 1c).

In the examples above, the deprotolithiation always occurred on the side toward which the oxygen of the sulfoxide directing group is pointed. In 2012, Tokitoh’s group showed that an *S*-phenylsulfinyl group could direct an initial deprotonation-trapping sequence before being converted to the *R*-phenylsulfinyl, able to direct the functionalization on the other side, leading to a stereopure 2,5-disubstituted ferrocenesulfoxide [44,45] (Figure 1d). During the synthesis of enantiopure ferrocene-1,2-disulfoxides that we recently reported, we showed that these reduction/oxidation steps could be avoided by adapting the reaction conditions [46]. This possibility of realizing the direct functionalization of the unfavorable position next to the sulfoxide has here been applied to the synthesis of many original derivatives, otherwise unreachable.

## 2. Results and Discussion

### 2.1. In Search of an Effective Protecting Group to Functionalize the Unfavorable Position Adjacent to the Sulfoxide

In order to obtain the required 2-substituted ferrocenesulfoxides, (*S*)-*S*-*tert*-butylferrocenesulfoxide (***S*-FcSO*t*Bu**) was prepared by reacting ferrocenyllithium [47] with (*S*,*S*)-2,2-diphenyl-1,2-dihydroxypropyl 2-*O*-*tert*-butylsulfinate [34]. (*R*)-*S*-*tert*-Butylferrocenesulfoxide (***R*-FcSO*t*Bu**) was prepared according to a modified reported procedure [48]. Finally, racemic *S*-*tert*-butylferrocenesulfoxide (***rac*-FcSO*t*Bu**) was obtained by oxidation of (*tert*-butylthio)ferrocene [49] (see Materials and Methods).

**Figure 2:**
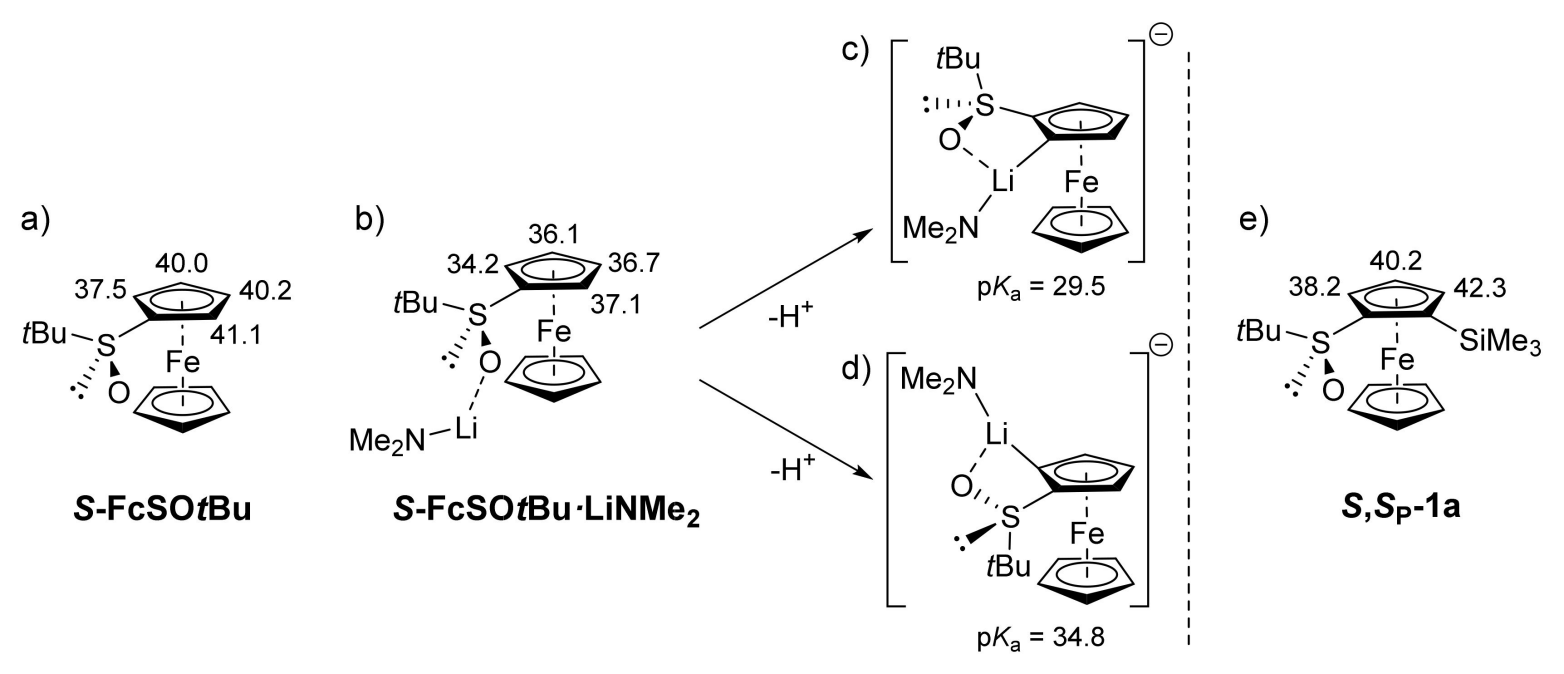
Selected calculated p*K*_a_ values of (**a**) ***S*-FcSO*t*Bu**, (**b**) its complex with LiNMe_2_, and (**c**,**d**) the corresponding anions; (**e**) selected calculated p*K*_a_ values of ***S*,*S*_P_-1a**.

In 2014, Šebesta rationalized the diastereoselective deprotolithiation of *S*-(4-tolyl)ferrocenesulfoxide by lithium amide by using DFT calculations [50]. However, to our knowledge, a related study has never been reported starting from *S*-*tert*-butylferrocenesulfoxide. Therefore, to explain the diastereoselectivity observed during the deprotolithiation of this substrate, we compared the thermodynamic acidity of different hydrogen atoms of the cyclopentadienyl ring. Since the p*K*_a_ values of (*S*)-*S*-*tert*-butylferrocenesulfoxide (***S*-FcSO*t*Bu**) have never been determined, they were calculated within the DFT framework (see Section 3.12 for details), as we had already done for other substrates [51,52,53,54]. For free ***S*-FcSO*t*Bu**, the position closest to the sulfinyl oxygen has the largest p*K*_a_ value (Figure 2a), which contradicts the results of deprotolithiation of ***S*-FcSO*t*Bu**. This indicates that, as already observed with ketone directing groups [55], the coordination of the sulfinyl oxygen to lithium has a significant effect on deprotolithiation [50] since it can lead to transition states (and lithiated products) stabilized by the formation of cyclic structures involving a lithium ion. To account for this effect, we performed p*K*_a_ calculations for the complex ***S*-FcSO*t*Bu∙LiNMe_2_**, in which the sulfinyl oxygen is coordinated to the lithium ion of a base, resulting in a 3–4 unit decrease in p*K*_a_ values (Figure 2b). At the same time, for the positions close to the sulfinyl group, the anions generated can be stabilized by the formation of cyclic structures involving a lithium ion. For the position closest to the sulfinyl oxygen, the formation of such an anionic cyclic structure leads to a decrease in p*K*_a_ of more than 11 units compared to the free ***S*-FcSO*t*Bu** (Figure 2c). The formation of a similar anionic structure with the other position next to the sulfinyl requires the rotation of the bulky *tert*-butyl moiety out of its *exo* position (Figure 2d). However, our calculations showed that the energy costs for such a rotation overcome the energy gain due to the formation of a cyclic anionic structure. As a consequence, the calculated p*K*_a_ value (34.8; Figure 2d) is slightly higher than in the case of the formation of an anion in which *t*Bu remains in the *exo* position (34.2; Figure 2b). Thus, the sulfinyl oxygen directs the deprotolithiation only at one of the two neighboring sites, leading to a stabilized cyclic structure involving a lithium ion in which the bulky part of the sulfinyl (here *t*Bu) remains in the *exo* position, in agreement with the experimental results.

The required 2-substituted derivatives of *S*-*tert*-butylferrocenesulfoxides were prepared, in the majority of cases, by treating the substrate with *n*-butyllithium in tetrahydrofuran (THF) at room temperature (rt) for 1 h before the interception by the electrophile (*Method A*) [34]. Under these conditions, the expected derivatives **1** (substituted on the position closest to the oxygen side of the sulfinyl) were isolated with good yields, whether by trapping with chlorotrimethylsilane (Table 1, entries 1–2), iodine (entries 3 and 4), heavy water (entry 5) and iodomethane (entry 6). The use of *tert*-butyllithium (1.5 equiv), this time at −80 °C (*Method B*) [56,57] in order to avoid decomposition of THF and consequent formation of undesirable products during the trapping step, difficult to separate, has also proved to be suitable for carrying out the deprotonation step; after quenching, the expected phosphine (entry 7) and stannane (entry 8) were isolated in high yields.

Deprotometalation of 2-substituted *S*-*tert*-butylferrocenesulfoxides is not favorable because the oxygen of the sulfinyl group of these compounds is now directed to the substituent and not to the neighboring free site. However, we have recently shown, using (*S*,*S*_P_)-*S*-*tert*-butyl-2-(phenylthio)ferrocenesulfoxide, that *Method A* is appropriate for such a functionalization [46]. Therefore, we applied these conditions to **1a**, a compound that benefits from p*K*_a_ values close to those of ***S*-FcSO*t*Bu** (Figure 2e) and that contains an easily removable trimethylsilyl protecting group.

This worked satisfactorily since, after interception with chlorotrimethylsilane (Table 2, entries 1 and 2), iodine (entries 3 and 4) or heavy water (entry 5), the corresponding trisubstituted derivatives **2a** were obtained with yields ranging from 63 to 88%. By applying *Method B* followed by trapping by benzophenone at ***S*,*S*_P_-1a**, the expected derivative **2ag** was also obtained, although with a moderate yield of 35% (entry 6). This last result seems to indicate that a contact of 1.5 h with *tert*-butyllithium at −80 °C is not sufficient to efficiently generate the lithiated intermediate; this is consistent with the lower reactivity predicted for the 2-substituted *S*-*tert*-butylferrocenesulfoxides.

As diphenylphosphino has already been used as a protecting group in the ferrocene series [58], we here explored this ability and tested *Method A* on the substrate ***S*,*S*_P_-1e**. Under these conditions, after iodolysis, the product **2eb** was isolated as phosphine oxide with a moderate 40% yield (entry 7). However, when the reaction was performed by using lithium 2,2,6,6-tetramethylpiperidide (LiTMP) at −80 °C for 1 h (*Method C*) before adding chlorotrimethylsilane as an electrophile, the expected ferrocene silane **2ea** was formed in 70% yield (entry 8). This better result could be explained by the higher compatibility of this electrophile toward hindered lithium amide, making it possible to shift the supposedly equilibrated deprotolithiation reaction by in situ trapping toward the formation of the silane [59]. Indeed, *Method C* proved to be unsuitable in the case of electrophiles incompatible with LiTMP, such as iodine.

Finally, we tested tributylstannyl as a protecting group [60,61,62] in the ferrocenesulfoxide series by reacting the substrate ***S*,*R*_P_-1f** with LiTMP at temperatures between −80 and −30 °C. However, all attempts were unsuccessful, even when chlorotrimethylsilane was employed as an in situ trap.

Before going further in the functionalization of ferrocene sulfoxides, we wanted to know if the approach developed here to access 2,5-disubstituted *S*-*tert*-butylferrocenesulfoxides could be extended to the synthesis of their *S*-(4-tolyl) counterparts. Indeed, the later replacement of 4-tolylsulfinyl offers more prospects than that of *tert*-butylsulfinyl, in particular by sulfoxide/lithium exchange [36,37,38]. For this purpose, (4-tolylthio)ferrocene was oxidized to racemic *S*-(4-tolyl)ferrocenesulfoxide (***rac*-FcSO-*p*-Tol**) while (*S*)-*S*-(4-tolyl)ferrocenesulfoxide (***S*-FcSO-*p*-Tol**) was prepared as previously reported [46].

Since *S*-(4-tolyl)ferrocenesulfoxides are subject to sulfoxide/lithium exchange in the presence of alkyllithiums, their deprotolithiation requires a lithium amide. While lithium diisopropylamide (LiDA) is generally used for this purpose [34,35], we have recently shown that the stronger LiTMP [63] is equally or even more suitable [46]. We therefore prepared the 2-silylated derivative by reacting ***rac*-FcSO-*p*-Tol** with LiTMP (1.2 equiv) in THF at −80 °C before trapping the lithiated intermediate with chlorotrimethylsilane (Table 3, entry 1). By increasing the amount of base to 1.5 equiv in order to improve the yield, we obtained the expected product (43% yield) but also isolated the disilylated derivative ***rac*-3a’** (19% yield; entry 2). The formation of the latter shows that the sulfoxide of the first is capable of directing a second deprotolithiation toward a neighboring position located on the tolyl group. Using iodine, an electrophile capable of instantaneously quenching the excess of LiTMP employed, no deprotonation on the tolyl ring was observed (entries 3 and 4), and the monoiodinated product was obtained in up to 81% yield. From ***S*-FcSO-*p*-Tol**, it was even possible to increase the amount of base to 2 equiv in the case of deuteriolysis (conc. DCl) with a similar result (entry 5).

**Scheme 1:**
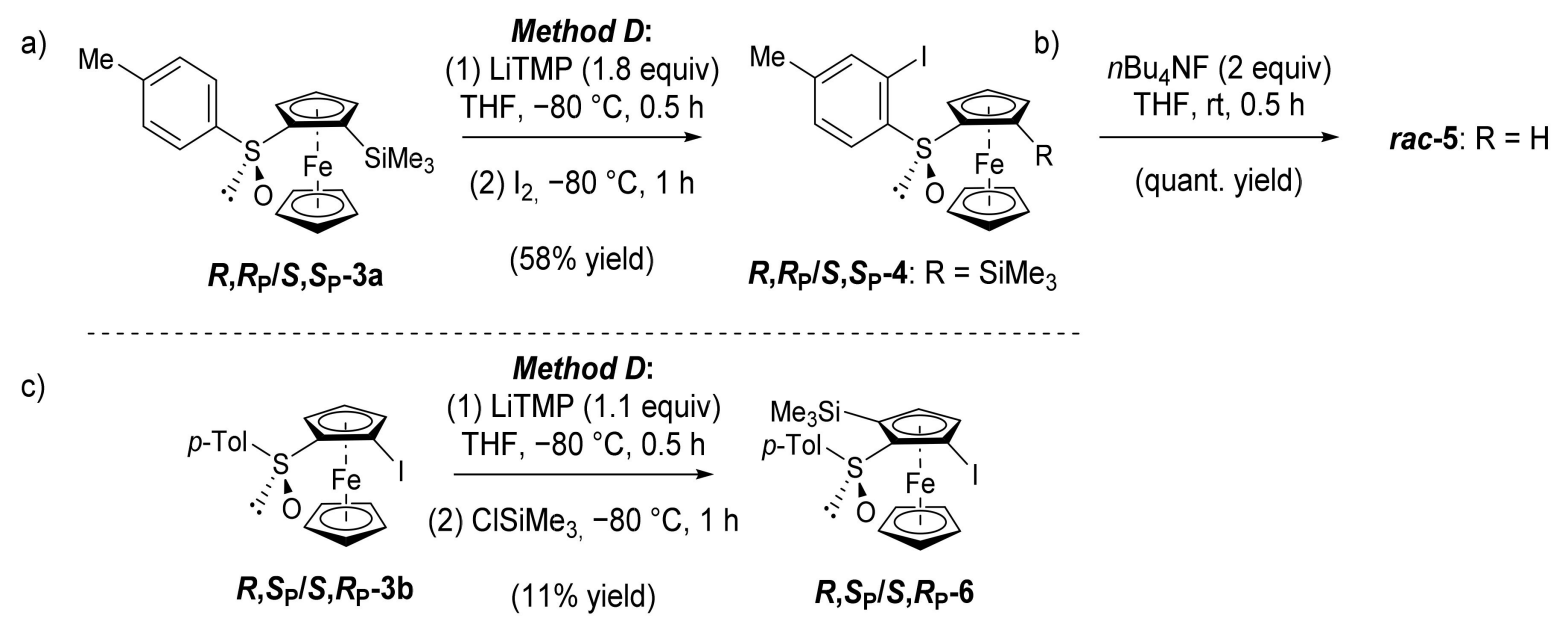
Unsuccessful attempts to access 2,5-disubstituted *S*-(4-tolyl)ferrocenesulfoxides.

Even though the competitive formation of a disilylated product during deprotolithiation-trapping from ***rac*-FcSO-*p*-Tol** was hardly engaging (Table 3, entry 2), we attempted a similar reaction from ***R*,*R*_P_/*S*,*S*_P_-3a** by using iodine as an electrophile (Scheme 1a). As feared, the sulfoxide directed the reaction toward the tolyl group, a result evidenced by the formation of the corresponding iodinated derivative ***R*,*R*_P_/*S*,*S*_P_-4**. Cleavage of the silyl group quantitatively led to *S*-(2-iodo-4-tolyl)ferrocenesulfoxide (***rac*-5**) from which derivatives of interest could be prepared (Scheme 1b).

Our objective being here to access 2,5-disubstituted *S*-(4-tolyl)ferrocenesulfoxides, we tested in the same way ***R*,*S*_P_/*S*,*R*_P_-3b** which bears an electron-withdrawing iodine that should promote the deprotonation on the ferrocene ring. However, after subsequent trapping with chlorotrimethylsilane, the expected product ***R*,*S*_P_/*S*,*R*_P_-6** was only isolated in a low 11% yield due to a high recovery (80%) of the starting material (Scheme 1c). By introducing chlorotrimethylsilane before the base, in order to displace by in situ trapping the supposedly equilibrated deprotolithiation reaction toward the formation of the silane [59], the substrate ***R*,*S*_P_/*S*,*R*_P_-3b** was completely recovered. These unsuccessful experiments led us to abandon the *S*-(4-tolyl)ferrocenesulfoxide route and turn again to *S*-(*tert*-butyl)ferrocenesulfoxides.

In order to shorten the procedure to access the 2,5-disubstituted *S*-*tert*-butylferrocenesulfoxides, we decided to attempt a one-pot deprotolithiation-trimethylsilylation-deprotolithiation-electrophilic trapping sequence from the enantiopure *S*-*tert*-butylferrocenesulfoxides. Three 2,5-disubstituted *S*-*tert*-butylferrocenesulfoxides already prepared above (***S*,*S*_P_-2ab**, ***R*,*R*_P_-2ab** and ***S*,*S*_P_-2ag**; see Table 1, entries 1 and 2, and Table 2, entries 3, 4, and 6) were thus obtained in good yields, especially considering the number of steps (Table 4, entries 1, 2 and 5). Additionally, original derivatives of ***S*-FcSO*t*Bu** were also prepared by using iodomethane (entry 3), chlorodiphenylphosphine (entry 4), Eschenmoser’s salt (entry 6), and *N*-fluorobenzenesulfonimide (NFSI; entry 7) as the second electrophiles.

Interestingly, removal of the trimethylsilyl group from these 2,5-disubstituted *S*-*tert*-butylferrocenesulfoxides **2** can lead to 2-substituted *S*-*tert*-butylferrocenesulfoxides **1**, diastereoisomers of those obtained by the direct deprotometalation-trapping from *S*-*tert*-butylferrocenesulfoxides. This desilylation was easily achieved by using tetrabutylammonium fluoride (TBAF; 2 equiv) in THF at rt [64], providing novel 2-substituted derivatives of *S*-*tert*-butylferrocenesulfoxides in high yields (Table 5, entries 1–5). From the *S*-*tert*-butyl-2,5-bis(trimethylsilyl)ferrocenesulfoxides **2aa**, reducing the amount of TBAF to 1 equiv in order to avoid bis-desilylation selectively afforded the product monodesilylated on the side of the sulfur lone pair (entries 6 and 7). Therefore, its diastereoisomer (*S*,*R*_P_)-*S*-*tert*-butyl-2-(trimethylsilyl)ferrocenesulfoxide (***S*,*R*_P_-1a**) cannot be reached by this approach. To our knowledge, there is no precedent on the topic. However, this result seems to suggest that the oxygen of the *tert*-butylsulfinyl group prevents to some extent an attack of the trimethylsilyl group by the fluoride.

### 2.2. Attempts to Apply the “Halogen Dance” Reaction to the Ferrocenesulfoxide Series

**Figure 3:**
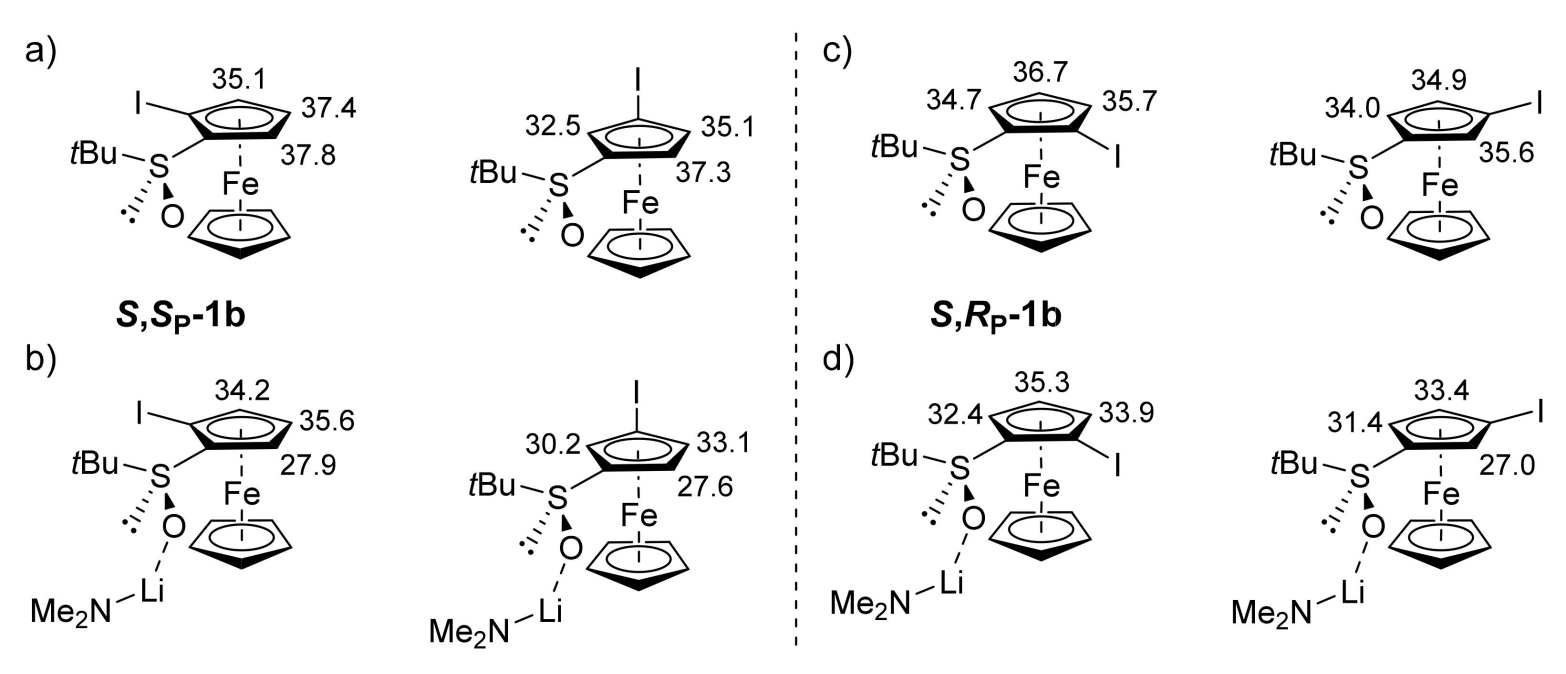
Selected calculated p*K*_a_ values of the (**a**,**c**) 2- and 3-iodinated *S*-*tert*-butylferrocenesulfoxides, and (**b**,**d**) their complexes with LiNMe_2_.

The “halogen dance” is a reaction in which halogen-substituted aromatics are isomerized [65,66,67,68,69,70,71,72,73]. Requiring a hindered lithium amide such as LiTMP, the reaction is driven by the stability of the arylmetal formed. As evidenced in the ferrocene series in the 2010s [60,74], it has since evolved to currently represent a valuable synthetic tool [51,52,53,54,64,75,76,77,78]. In the continuity of this work, we sought to implement this reaction by using the sulfoxide as a stabilizing/directing group and the trimethylsilyl as a protecting group. The p*K*_a_ values calculated for the complexes between 2- and 3-iodinated *S*-*tert*-butylferrocenesulfoxides and LiNMe_2_ seem to indicate that the *S*,*S*_P_ (or *R*,*R*_P_) stereoisomer would only be a suitable substrate after the protection of the free position next to the sulfoxide (Figure 3a,b). Indeed, as the position activated by the sulfoxide is the most acidic for both ***S*,*S*_P_-1b·LiNMe_2_** and its isomerized derivative, a migration is not expected without protection. Regarding the *S*,*R*_P_ (or *R*,*S*_P_) stereoisomer, the p*K*_a_ values indicate that this substrate could be tested protected or even as is (Figure 3c,d).

**Scheme 2:**
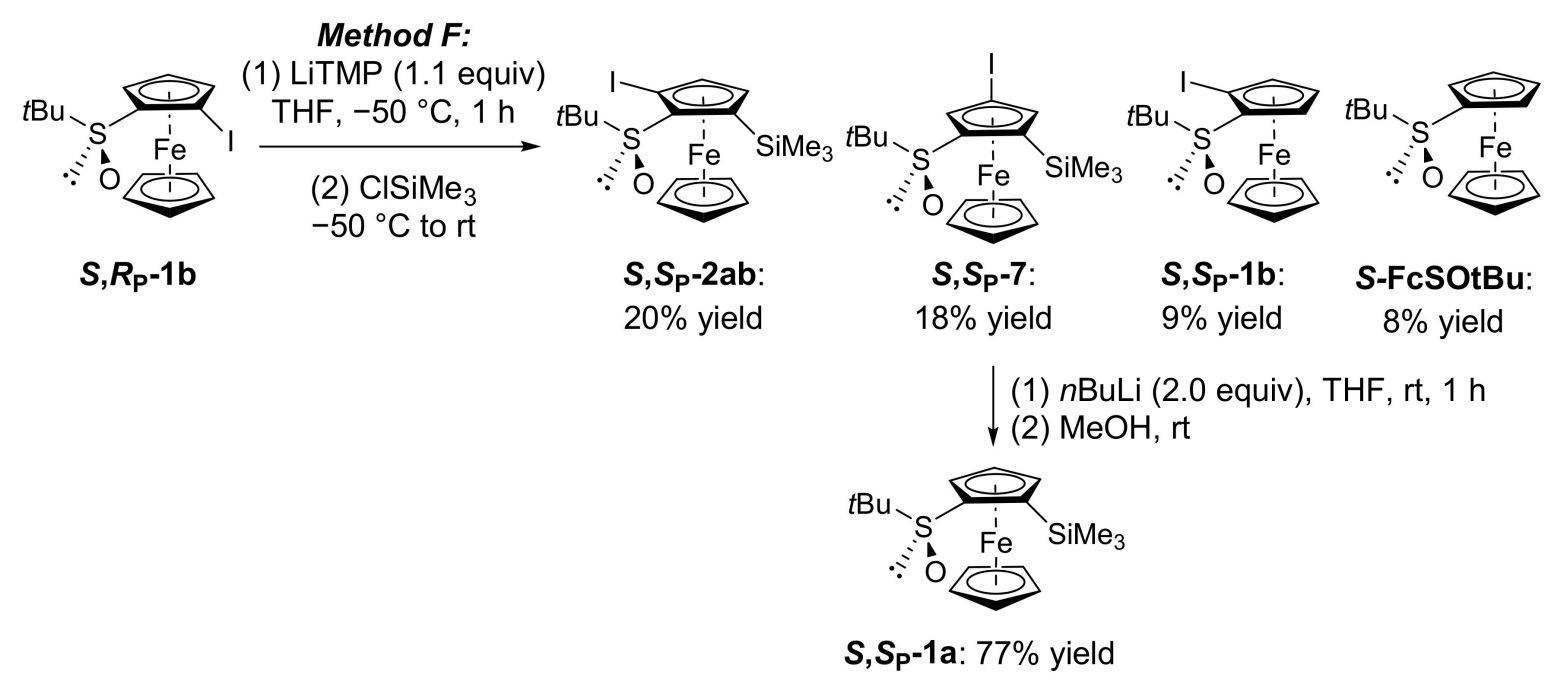
Attempt to perform “halogen dance” from (*S*,*R*_P_)-*S*-*tert*-butyl-2-iodoferrocenesulfoxide (***S*,*R*_P_-1b**).

Since the p*K*_a_ values indicated that (*S*,*R*_P_)-*S*-*tert*-butyl-2-iodoferrocenesulfoxide (***S*,*R*_P_-1b**) might be used in “halogen dance” without trimethylsilyl protection, the reaction was attempted by using our standard conditions (1.1 equiv of LiTMP, THF, −50 °C, 2 h; *Method F*) [51,52,53,54,64,75,77,78]. However, after methanolysis, a complex mixture was obtained within which only ***S*,*S*_P_-1b** could be identified. When chlorotrimethylsilane was used as the electrophile after 1 h of contact, a mixture was obtained, from which ***S*,*S*_P_-2ab**, ***S*,*S*_P_-7**, ***S*,*S*_P_-1b** and ***S*-FcSO*t*Bu** were isolated with yields of 20, 18, 9, and 8%, respectively (Scheme 2). While the structure of ***S*,*S*_P_-7** might appear as atypical for a classic “halogen dance” reaction, it is in agreement with the formation of the compound ***S*,*S*_P_-1a** by iodine/lithium exchange, and was further unambiguously confirmed by X-ray diffraction.

**Scheme 3:**
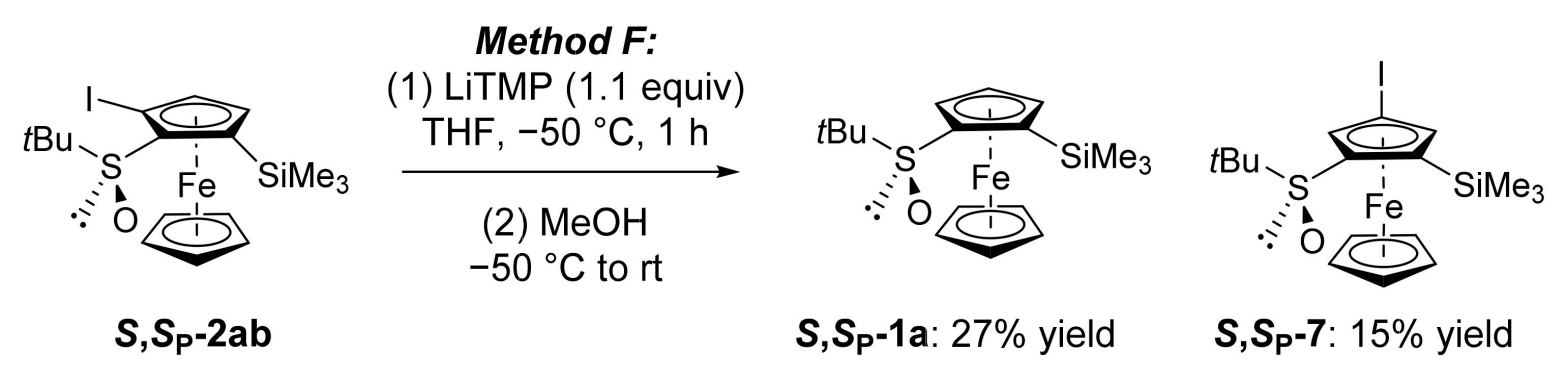
Attempt to perform “halogen dance” from (*S*,*S*_P_)-*S*-*tert*-butyl-2-iodo-5-(trimethylsilyl)ferrocenesulfoxide (***S*,*S*_P_-2ab**).

We next attempt the “halogen dance” reaction from (*S*,*S*_P_)-*S*-*tert*-butyl-2-iodo-5-(trimethylsilyl)ferrocenesulfoxide (***S*,*S*_P_-2ab**) as before (*Method F*), followed by a methanolysis. The compound ***S*,*S*_P_-7** was isolated in a similar yield as before while the large amount of ***S*,*S*_P_-1a** isolated in this reaction suggests that LiTMP is also able to exchange iodine, as already proposed in the literature [51,52,75,79] (Scheme 3).

**Scheme 4:**
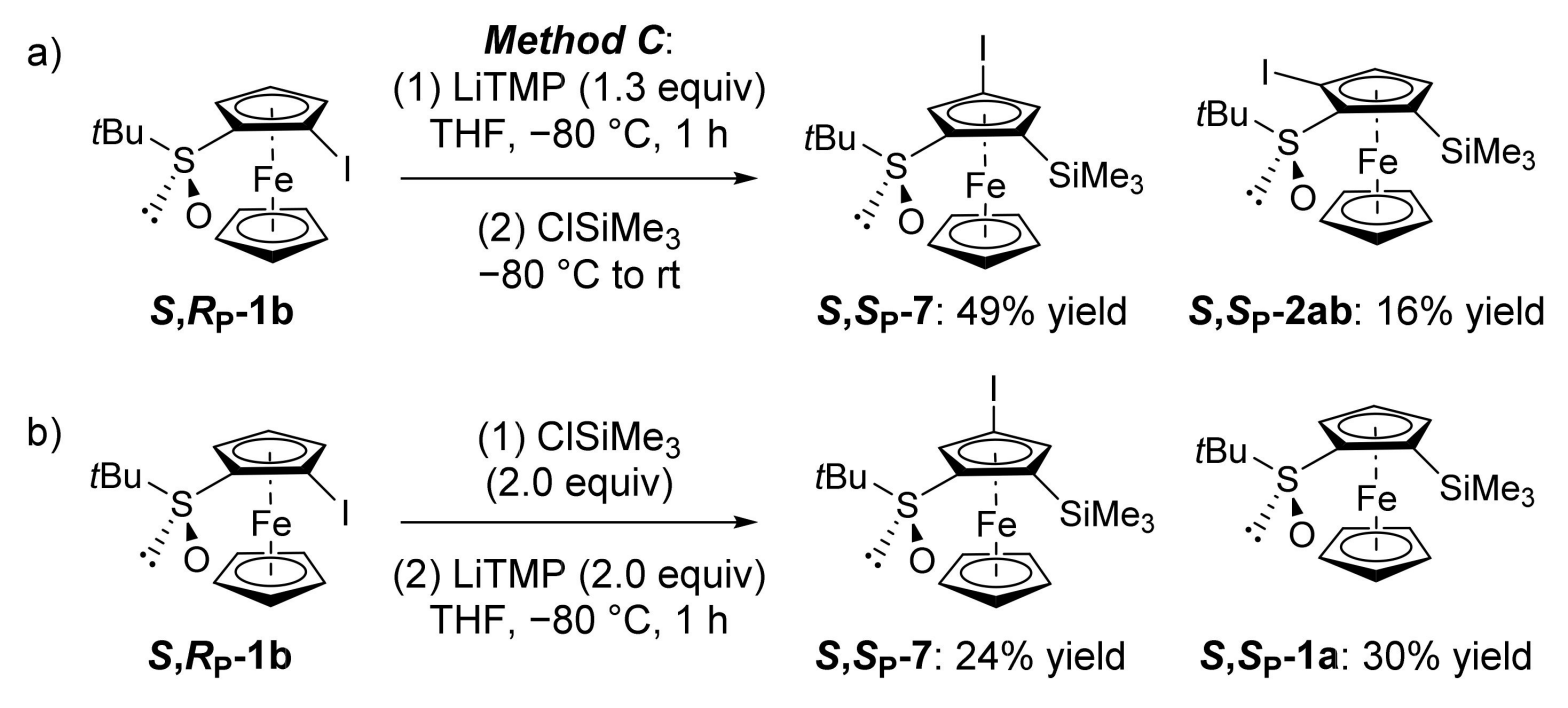
Unsuccessful attempts to access (*S*,*R*_P_)-*S*-*tert*-butyl-2-iodo-5-(trimethylsilyl)ferrocenesulfoxide (***S*,*R*_P_-2ba**).

**Scheme 5:**
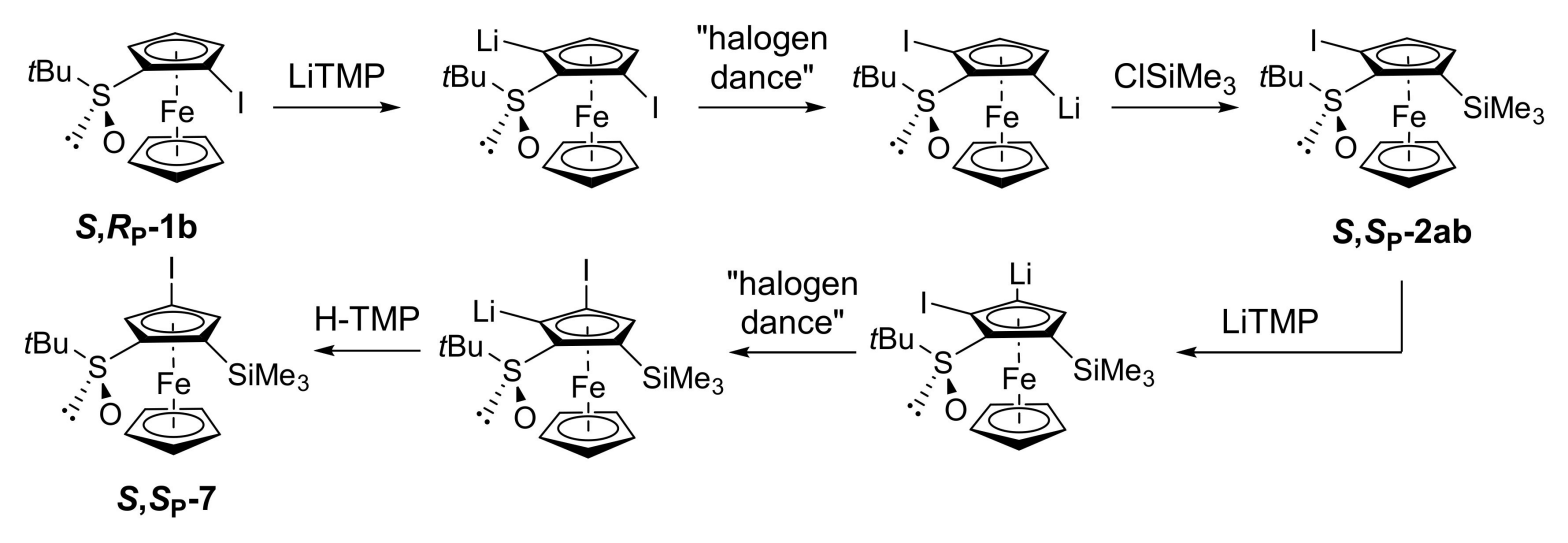
Proposed pathway to explain the formation of (*S*,*S*_P_)-*S*-*tert*-butyl-2-iodo-5-(trimethylsilyl)ferrocenesulfoxide (***S*,*S*_P_-2ab**) and (*S*,*S*_P_)-*S*-*tert*-butyl-4-iodo-2-(trimethylsilyl)ferrocenesulfoxide (***S*,*S*_P_-7**).

While we already evaluated the behavior of (*S*,*S*_P_)-*S*-*tert*-butyl-2-iodo-5-(trimethylsilyl)ferrocenesulfoxide (***S*,*S*_P_-2ab**) in the “halogen dance” reaction, it was of interest to test its diastereoisomer ***S*,*R*_P_-2ba** under similar conditions. As it is possible to deprotolithiate the sulfoxide adjacent position of ***S*,*R*_P_-1b** (Scheme 2), it was treated successively with LiTMP in THF at −80 °C for 1 h (*Method C*) and then with chlorotrimethylsilane. However, instead of the expected product, we again isolated ***S*,*S*_P_-7** (this time in 49% yield) as well as ***S*,*S*_P_-2ab** in 16% yield (Scheme 4a). Additionally, the use of LiTMP at −80 °C in the presence of chlorotrimethylsilane as an in situ trap (both used in excess) also led to the products ***S*,*S*_P_-7** (24% yield) and ***S*,*S*_P_-1a** (30% yield) (Scheme 4b). Taken together, a putative reaction pathway including two successive “halogen dance” reactions toward the compound ***S*,*S*_P_-7** could be proposed (Scheme 5).

**Scheme 6:**
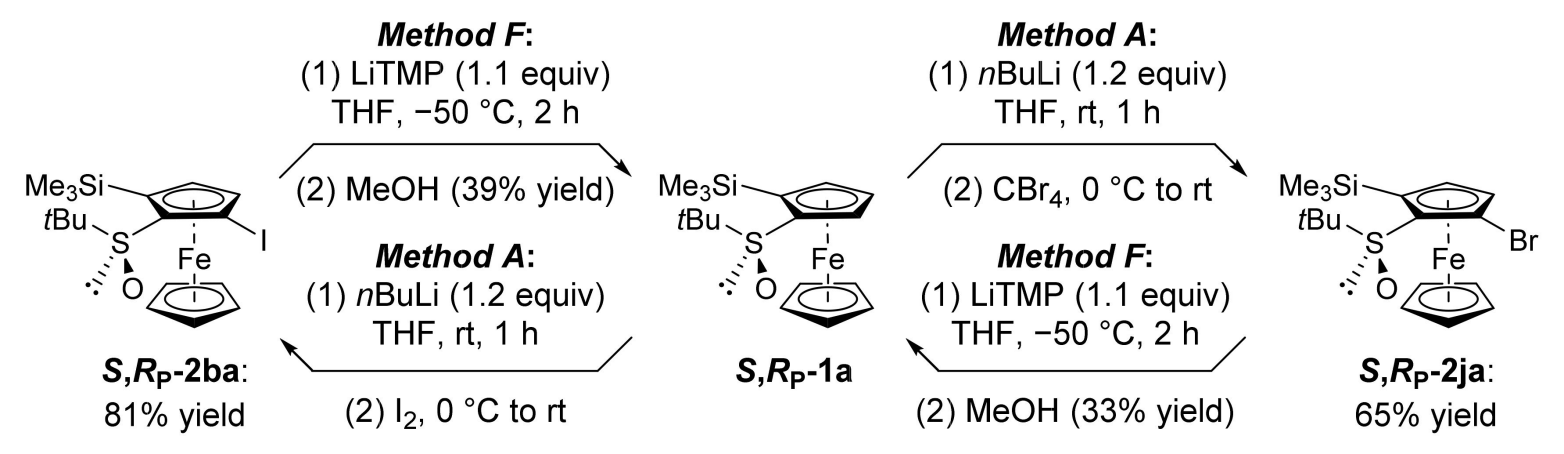
Access to (*S*,*R*_P_)-*S*-*tert*-butyl-2-(trimethylsilyl)ferrocenesulfoxide (***S*,*R*_P_-1a**) and reactivity.

Although ***S*,*R*_P_-2ba** cannot be reached directly from ***S*,*R*_P_-1b**, it can be prepared by following our recently reported work [46]. However, when it was engaged into a “halogen dance” reaction (*Method F*), we only observed an iodine/lithium exchange, leading to the compound ***S*,*R*_P_-1a** (39% yield), while 50% of starting material was recycled. Replacement of iodine from ***S*,*R*_P_-2ba** with bromine in ***S*,*R*_P_-2ja** (prepared by using *Method A*) did not change the outcome of the reaction, as ***S*,*R*_P_-1a** was this time isolated in 33% yield (Scheme 6).

### 2.3. On the Way to Polysubstituted Ferrocenesulfoxides

**Scheme 7:**
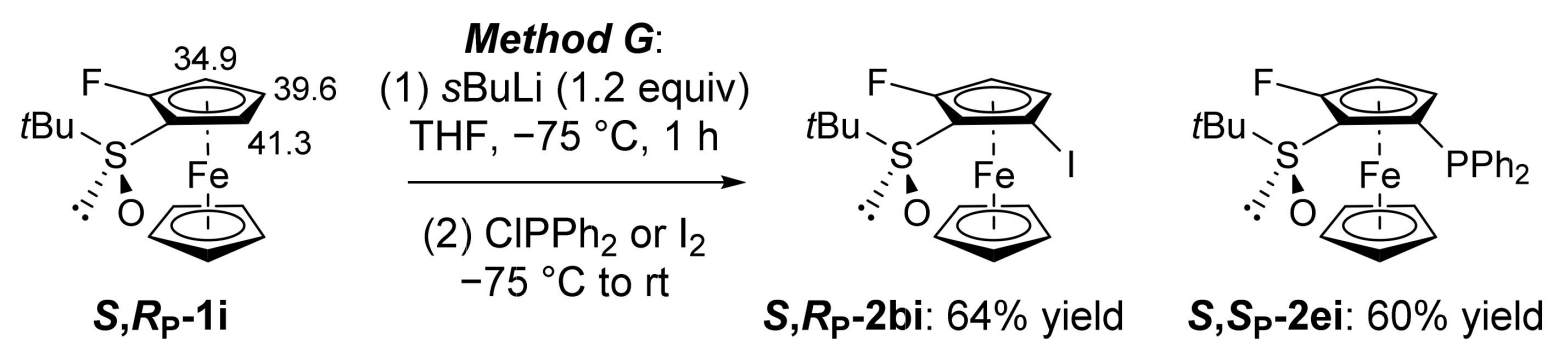
Deprotometalation-trapping sequences from (*S*,*R*_P_)-*S*-*tert*-butyl-2-fluoroferrocenesulfoxide (***S*,*R*_P_-1i**) and p*K*_a_ values.

Since fluorine is also a group able to direct deprotometalation [80,81], we then studied the reactivity of the fluorinated ferrocenesulfoxide ***S*,*R*_P_-1i** (see Table 5). Inspired by previous studies in fluoroferrocene series [52,64], we chose *sec*-butyllithium to carry out the reaction in THF at −75 °C (*Method G*). Trapping with iodine or chlorodiphenylphosphine yielded the halide ***S*,*R*_P_-2bi** and the phosphine ***S*,*S*_P_-2ei**, a result consistent with a sulfoxide-directed deprotolithiation (Scheme 7).

**Scheme 8:**
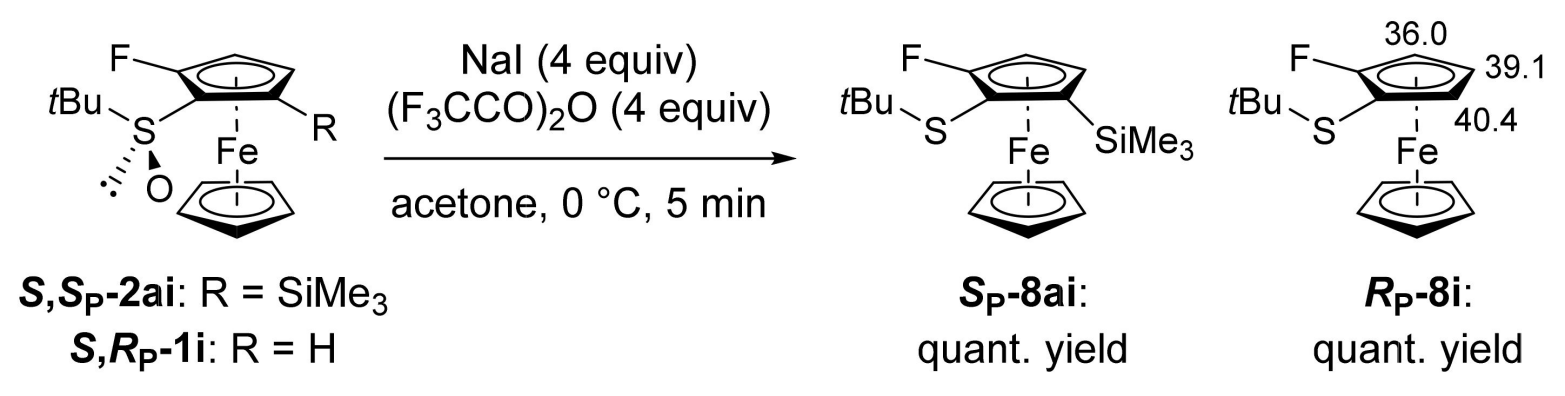
Access to (*S*_P_)-2-(*tert*-butylthio)-1-fluoro-3-(trimethylsilyl)ferrocene (***S*_P_-8ai**) and (*R*_P_)-1-(*tert*-butylthio)-2-fluoroferrocene (***R*_P_-8i**) and p*K*_a_ values of ***R*_P_-8i**.

The fluorinated ferrocenesulfoxides ***S*,*S*_P_-2ai** (see Table 4) and ***S*,*R*_P_-1i** (see Table 5) were easily reduced to the sulfides ***S*_P_-8ai** and ***R*_P_-8i**, respectively, in the presence of sodium iodide and trifluoroacetic anhydride in acetone at 0 °C [82] (quantitative yields; Scheme 8). In contrast, it proved impossible to access ***R*_P_-8i** from ***S*_P_-8ai** by trimethylsilyl cleavage under the conditions previously used (TBAF (2 equiv), THF, rt).

We then thought interesting to observe the behavior of these (*tert*-butylthio)ferrocenes in deprotometalation as there is only one mention of such a reaction in the literature [83]. In their study, Brown and co-workers employed *sec*-butyllithium in THF under different conditions to deprotonate (4-tolylthio)ferrocene quite regioselectively: either at C3 by performing the reaction at 0 °C, or at C1′ at 30 °C, or even at C2 at −75 °C in the presence of potassium *tert*-butoxide. A similar regioselectivity in the case of (*tert*-butylthio)ferrocene was also claimed, but without reporting the electrophiles used and the yields obtained. 3-(*tert*-Butylthio)ferrocenecarboxaldehyde has since been mentioned in a scheme of a patent [84], but again without further details.

In our hands, despite the presence of fluorine as a directing group, ***S*_P_-8ai** could not be deprotometalated by using *sec*-butyllithium in THF at temperatures between −75 and 0 °C, or even at −75 °C in the presence of potassium *tert*-butoxide. Indeed, after subsequent iodolysis, the starting material was always recovered. This reluctance to deprotometalation is surprising since fluoroferrocene is functionalized under similar conditions. From ***R*_P_-8i**, which benefits from p*K*_a_ values very close to those of fluoroferrocene [52] (Scheme 8), an inseparable mixture of iodides and starting material was obtained when *sec*-butyllithium was employed in the presence of potassium *tert*-butoxide at −75 °C.

**Scheme 9:**
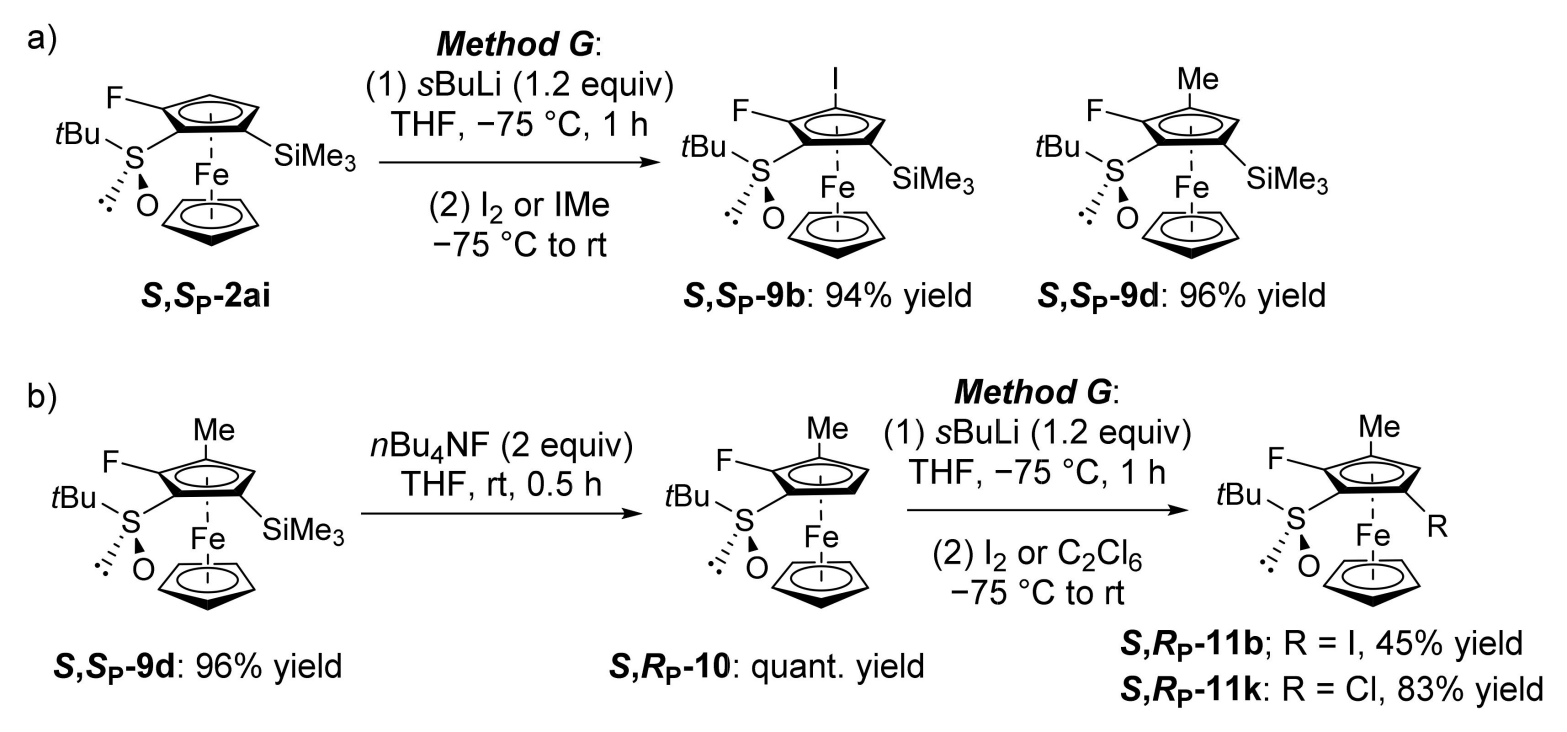
Deprotometalation-trapping sequences from (**a**) (*S*,*S*_P_)-*S*-*tert*-butyl-2-fluoro-5-(trimethylsilyl)ferrocenesulfoxide (***S*,*S*_P_-2ai**) and (**b**) (*S*,*R*_P_)-*S*-*tert*-butyl-2-fluoro-3-methylferrocenesulfoxide (***S*,*R*_P_-10**).

Thus, we turned back to the fluorinated ferrocenesulfoxide ***S*,*S*_P_-2ai** to consider its further functionalization. This time, the use of *sec*-butyllithium in THF at −75 °C for 1 h (*Method G*) and subsequent quenching with iodine or iodomethane resulted in the clean formation of the products functionalized next to fluorine with excellent yields (Scheme 9a). From the methylated product ***S*,*S*_P_-9d**, desilylation gave ***S*,*R*_P_-10**. Deprotolithiation of the latter logically occurred next to the sulfoxide group by using *Method G* to give, after trapping with iodine or hexachloroethane, the sulfoxide-containing tetrasubstituted ferrocenes ***S*,*R*_P_-11** (Scheme 9b).

**Scheme 10:**
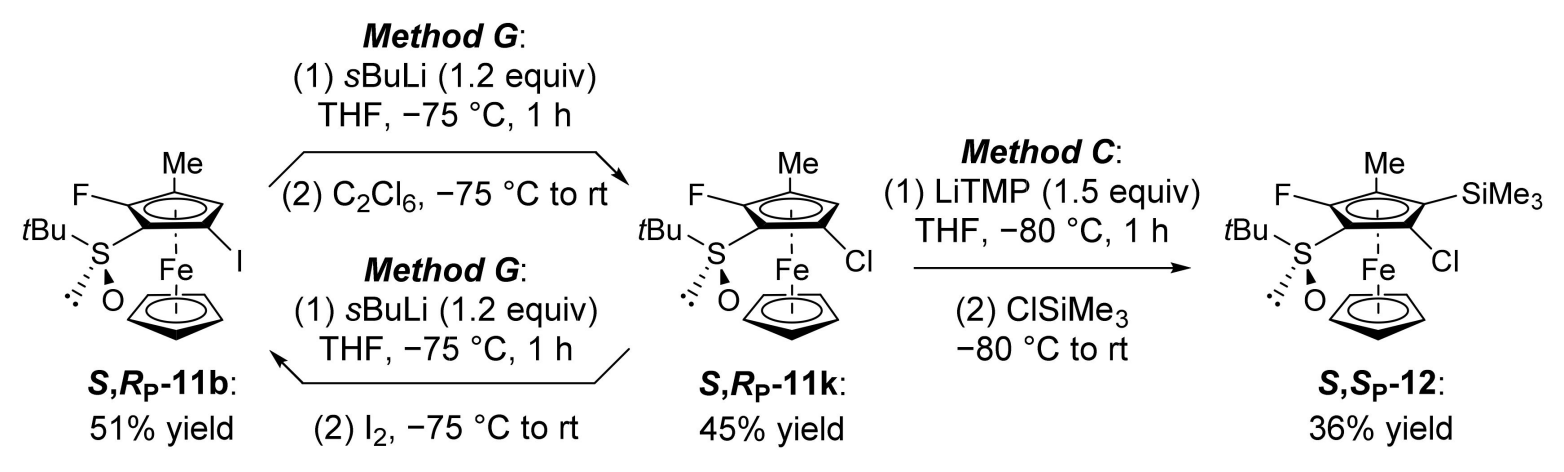
Deprotometalation-trapping sequences from (*S*,*R*_P_)-*S*-*tert*-butyl-5-chloro-2-fluoro-3-methylferrocenesulfoxide (***S*,*R*_P_-11k**).

In order to reach the first sulfoxide-based pentasubstituted ferrocene, the chlorinated compound ***S*,*R*_P_-11k** was subjected to *Method G*. Surprisingly, instead of the expected deprotometalation next to chlorine, we observed a sulfoxide-induced chlorine/lithium exchange [85,86]; this was demonstrated by subsequent trapping with iodine, leading to ***S*,*R*_P_-11b**. This iodide was therefore subjected to an iodine/lithium exchange-hexachloroethane trapping sequence in order to recover the starting chloride ***S*,*R*_P_-11k**. This could finally be converted to the expected original 2,3,4,5-tetrasubstituted ferrocenesulfoxide ***S*,*S*_P_-12** by using *Method C* with chlorotrimethylsilane as the electrophile (Scheme 10).

### 2.4. Specific Solid-State Structures of Some Ferrocenesulfoxides

**Figure 4:**
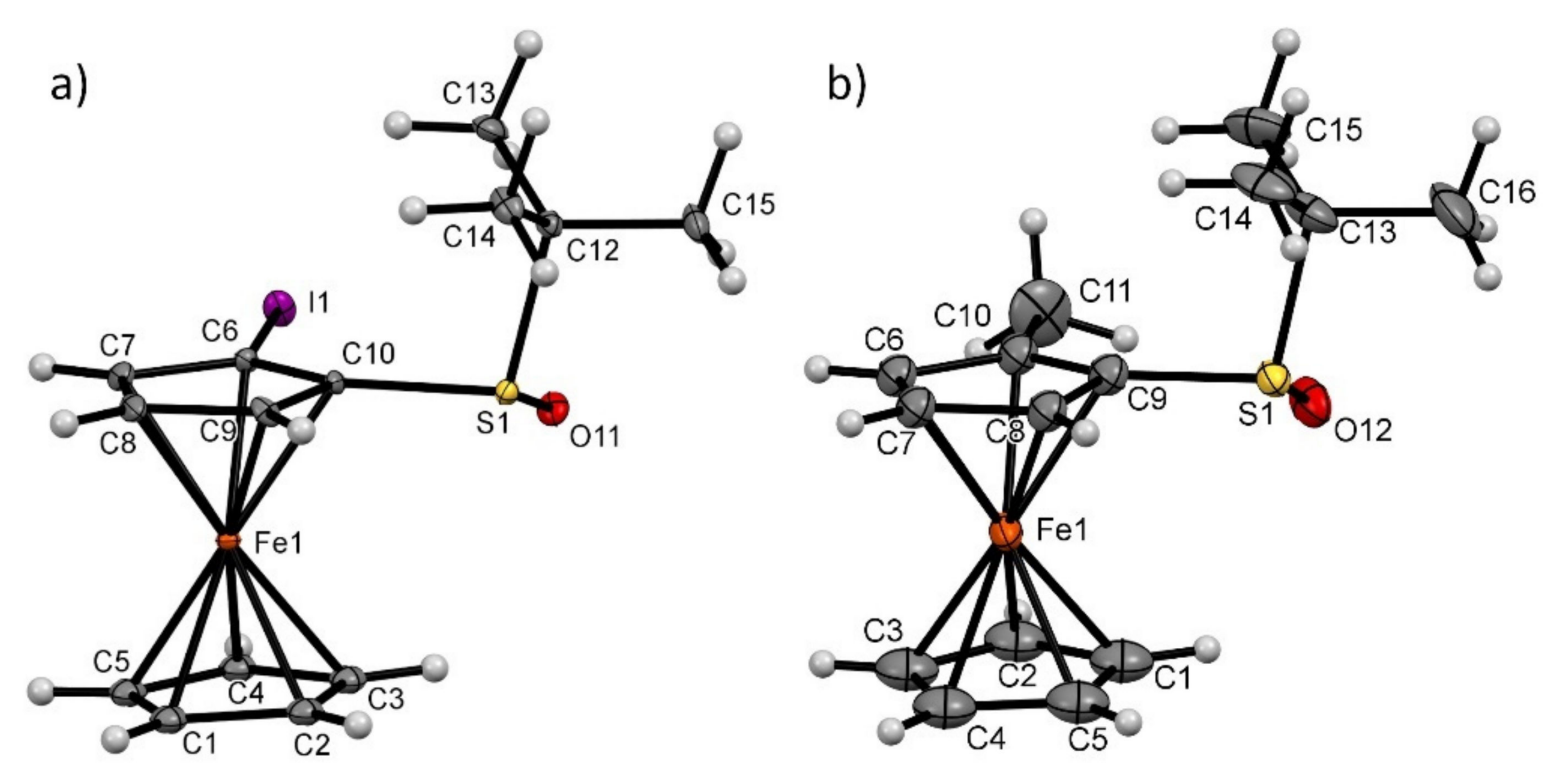
Molecular structure of the compounds (**a**) ***S*,*R*_P_-1b** and (**b**) ***S*,*S*_P_-1d** at the solid state. Thermal ellipsoids shown at the 30% probability level. Selected bond lengths [Å] and angles [°] for ***S*,*R*_P_-1b**: C10-S1 = 1.791(4), C6-I1 = 2.099(4), O11-S1-C10-C6 = −15.57. Selected bond lengths [Å] and angles [°] for ***S*,*S*_P_-1d**: C9-S1 = 1.766(7); C10-C11 = 1.500(1), O12-S1-C9-C10 = −20.14.

In the frame of this study, we were able to grow crystals suitable for X-ray diffraction analysis for the ferrocenesulfoxides ***S*,*R*_P_-1b**, ***S*,*S*_P_-1d**, ***S*,*S*_P_-7** and ***S*,*S*_P_-9b**. Although the solid-state structure of (*S*,*S*_P_)-*S*-*tert*-butyl-2-methylferrocenesulfoxide (***S*,*S*_P_-1d**) is similar to one reported by Kagan, it can be compared with the one of (*S*,*R*_P_)-*S*-*tert*-butyl-2-iodoferrocenesulfoxide (***S*,*R*_P_-1b**) as the two structures are very similar (Figure 4). Indeed, the S=O bonds are slightly bended toward the iron, with similar C10-S1 and C9-S1 bond lengths while C6-I1 and C10-C11 were found to fall in the range of such bonds [87,88]. Furthermore, both ferrocene cores were found in a staggered conformation with a torsion angle C10-Cg1···Cg2-C3 of −12.70° for ***S*,*R*_P_-1b** (Cg1 being the centroid of the C6-C7-C8-C9-C10 ring and Cg2 being the centroid of the C1-C2-C3-C4-C5 ring) and a torsion angle C9-Cg1···Cg2-C1 of −8.92° for ***S*,*S*_P_-1d** (Cg1 bring the centroid of C6-C7-C8-C9-C10 ring and Cg2 being the centroid of C1-C2-C3-C4-C5 ring).

**Figure 5:**
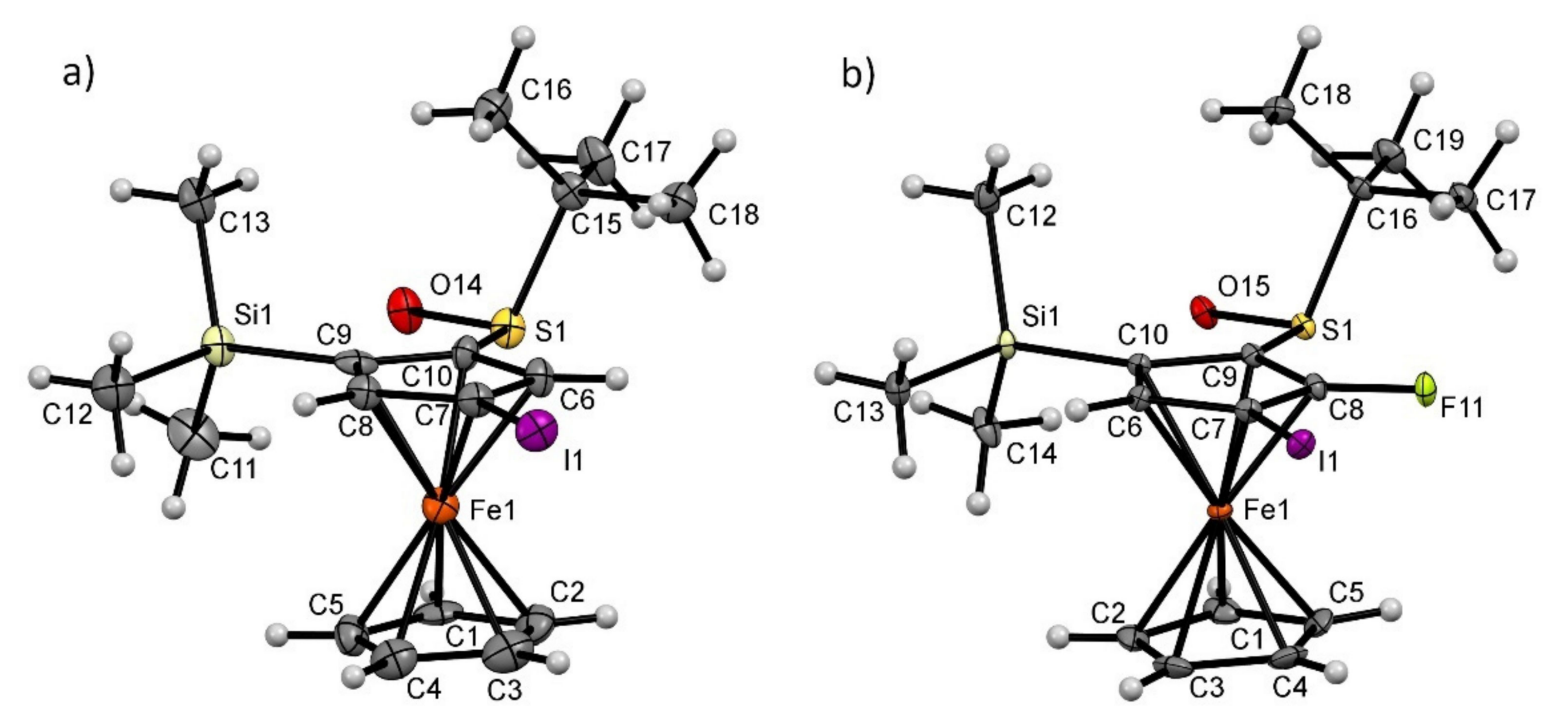
Molecular structure of the compounds (**a**) ***S*,*S*_P_-7** and (**b**) ***S*,*S*_P_-9b** at the solid state. Thermal ellipsoids shown at the 30% probability level. Selected bond lengths [Å] and angles [°] for ***S*,*S*_P_-7**: C10-S1 = 1.770(1), C9-Si1 = 1.890(1), C7-I1 = 2.090(1), O14-S1-C10-C9 = 4.18, I1···O14 = 2.926, S1-O15···I1 = 116.72, O15···I1-C7 = 172.48. Selected bond lengths [Å] and angles [°] for ***S*,*S*_P_-9b**: C9-S1 = 1.790(4), C10-Si1 = 1.893(5), C7-I1 = 2.090(5), C8-F11 = 1.349(5), O15-S1-C9-C10 = 1.36, I1···O14 = 2.965, S1-O14···I1 = 113.12, O14···I1-C7 = 173.46.

**Figure 6:**
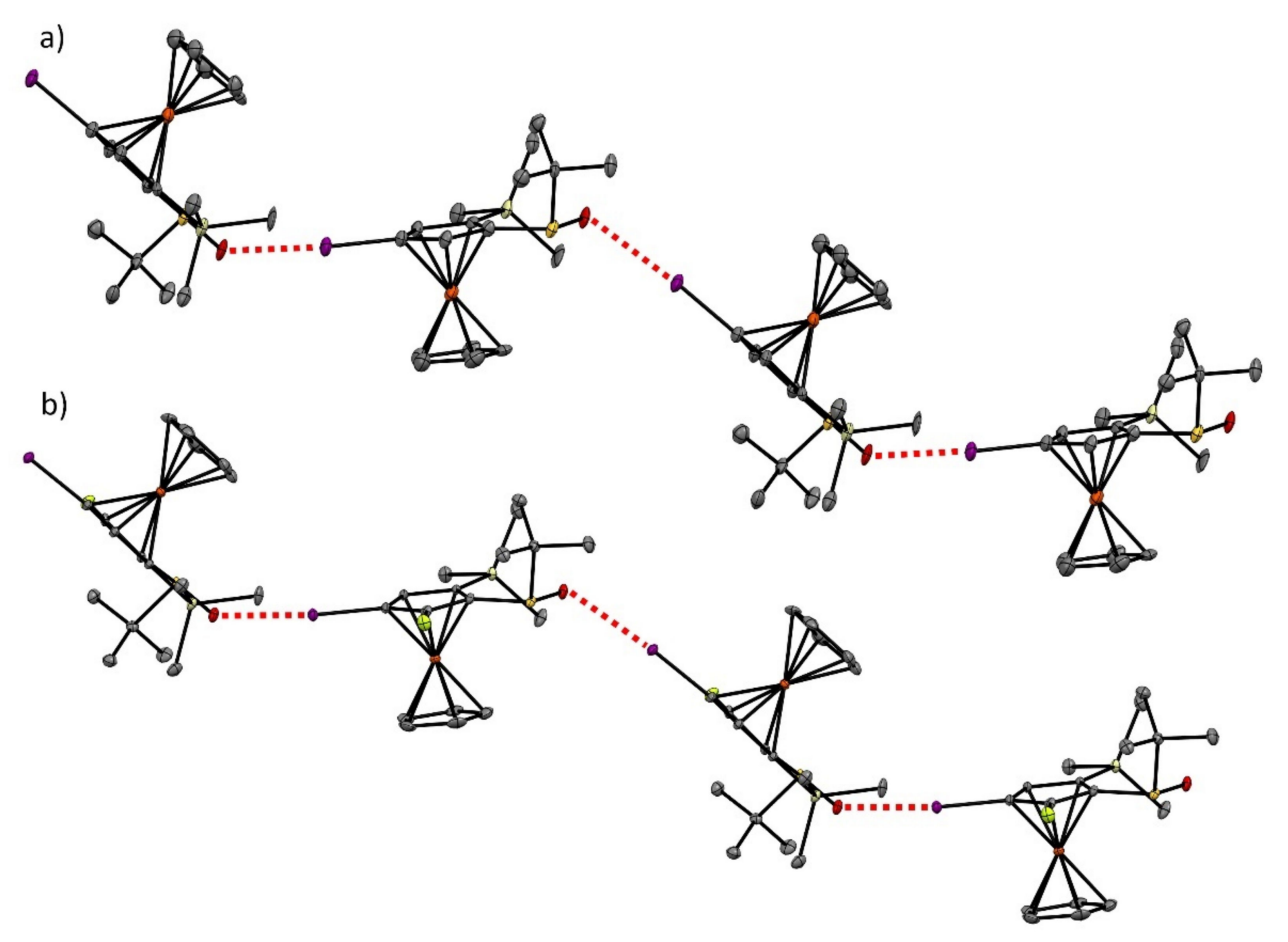
Halogen bond network observed at the solid state for the compound (**a**) ***S*,*S*_P_-7** and (**b**) ***S*,*S*_P_-9b**. Thermal ellipsoids shown at the 30% probability level. The hydrogens were omitted for clarity.

The only difference between the compounds ***S*,*S*_P_-7** and ***S*,*S*_P_-9b** is an additional fluorine atom for the latter. Therefore, they have almost identical structural characteristics in terms of C-S, C-Si, C-I bond lengths, and coplanarity between the oxygen of the sulfinyl group and the substituted cyclopentadienyl ring (Figure 5). Furthermore, both ferrocene cores were found in an eclipsed conformation with a torsion angle C10-Cg1···Cg2-C1 of −22.47° for ***S*,*S*_P_-7** and a torsion angle C9-Cg1···Cg2-C1 of −19.42° for ***S*,*S*_P_-9b** (Cg1 being the centroid of the C6-C7-C8-C9-C10 ring and Cg2 being the centroid of the C1-C2-C3-C4-C5 ring). Finally, due to the presence of iodine remote from the sulfoxide, interactions between the former and the oxygen of the latter were identified, leading to chains of molecules at the solid-state (Figure 6) [53,54,89]. For the two compounds, their I···O bond lengths and their S-O···I and O···I-C angles indicate an interaction between the lone pair of the oxygen and the σ-hole of the iodine atom, characteristic of halogen bonds [90,91].

## 3. Materials and Methods

### 3.1. General Information

All reactions were carried out in Schlenk tubes under a dry argon atmosphere. THF was freshly distilled under argon from sodium-benzophenone. All alkyllithiums were titrated before use [92]. 2,2,6,6-Tetramethypiperidine was distilled over CaH_2_ under reduced pressure and stored over KOH pellets. Room temperature (rt) refers to 25 °C. Column chromatography separations were achieved on silica gel (40–63 μm). All thin-layer chromatographies (TLC) were performed on aluminum-backed plates pre-coated with silica gel (Merck, Silica Gel 60 F254). They were visualized by exposure to UV light. Melting points were measured on a Kofler apparatus. Infrared (IR) spectra were taken on an ATR Perkin-Elmer Spectrum 100 spectrometer (Perkin-Elmer, Waltham, MA, USA) and the main absorption wavenumbers are given in cm^−1^. ^1^H and ^13^C{^1^H} nuclear magnetic resonance (NMR) spectra were recorded at 300 K either on a Bruker Avance III HD spectrometer fitted with a BBFO probe at 500 MHz and 126 MHz, respectively, or on a Bruker Avance III spectrometer fitted with a BBFO probe at 300 MHz and 75.4 MHz respectively (Bruker, Billevica, MA, USA). ^1^H chemical shifts (δ) are given in ppm relative to the solvent residual peak, and ^13^C{^1^H} chemical shifts (δ) are given in ppm relative to the central peak of the solvent signal [93]. Cp refers to the unsubstituted cyclopentadienyl ring of ferrocene. The NMR data of all compounds described, selected NOESY correlations and the numbering used below for the NMR assignment is given in the Supplementary Materials. Optical rotations were determined on a Perkin Elmer 341 polarimeter (589 nm); the concentrations (*c*) are given in g/100 mL.

### 3.2. Crystallography

The samples were studied with monochromatized Mo-Kα radiation (λ = 0.71073 Å). The X-ray diffraction data of the compounds ***S*,*R*_P_-1b**, ***S*,*S*_P_-1d** and ***S*,*S*_P_-9b** were collected at *T* = 150(2) K by using a D8 VENTURE Bruker AXS diffractometer equipped with a (CMOS) PHOTON 100 detector. The X-ray diffraction data of the compounds **FcSOFc** and ***S*,*S*_P_-7** were collected at *T* = 150(2) K by using an APEXII Kappa-CCD (Bruker-AXS) diffractometer equipped with a CCD plate detector and a CCD-LDI-APEX2 detector, respectively. The crystal structures were solved by the dual-space algorithm using *SHELXT* program [94] and then refined with full-matrix least-squares methods based on *F*^2^ (*SHELXL* program) [95]. All non-hydrogen atoms were refined with anisotropic atomic displacement parameters. H atoms were finally included in their calculated positions and treated as riding on their parent atom with constrained thermal parameters. The molecular diagrams were generated by Mercury 2020.3.0.

### 3.3. Safety Considerations

Due to its high pyrophoric character, *tert*-butyllithium has to be used under anhydrous conditions and nitrogen or argon atmosphere.

### 3.4. Starting Materials

(*R*)-*S*-*tert*-Butylferrocenesulfoxide (***R*-FcSO*t*Bu**) was prepared as reported previously [46], by modifying a reported procedure [48]. 

(*S*)-*S*-*tert*-Butylferrocenesulfoxide (***S*-FcSO*t*Bu**) was prepared as reported previously [46], by reacting ferrocenyllithium [47] with (*S*,*S*)-2,2-diphenyl-1,2-dihydroxypropyl 2-*O*-*tert*-butylsulfinate [34]. Diferrocenesulfoxide (**FcSOFc**) can also be formed as a by-product. It was identified by ^1^H NMR, and its structure was confirmed by X-ray diffraction analysis. ^1^H NMR (CDCl_3_) *δ* 4.30 (m, 2H, H2 or H5), 4.33 (s, 10H, Cp), 4.36 (t, *J* = 2.2 Hz, 4H, H3 and H4), 4.68 (q, *J* = 1.7 Hz, 2H, H2 or H5). Crystal data for **FcSOFc**. C_20_H_18_Fe_2_OS, *M* = 418.10; monoclinic *P* 2_1_/*c* (I.T.#14), *a* = 10.8267(7), *b* = 10.0035(5), *c* = 15.0821(7) Å, *β* = 99.897(2)°, *V* = 1609.16(15) Å^3^. *Z* = 4, *d* = 1.726 g.·cm^−3^, *μ* = 1.935 mm^−1^. A final refinement on *F*^2^ with 3652 unique intensities and 217 parameters converged at ω*R(F^2^)* = 0.0668 (*R_F_* = 0.0302) for 3057 observed reflections with *I* > 2σ(*I*). CCDC 2152196.

(*S*)-*S*-(4-Tolyl)ferrocenesulfoxide (***S*-FcSO-*p*-Tol**) [46], (1*R*,2*S*,5*R*)-(−)-menthyl (*S*)-4-toluenesulfinate [96], (*S*,*R*_P_)-*S*-*tert*-butyl-2-iodo-5-(trimethylsilyl)ferrocenesulfoxide (***S*,*R*_P_-2ba**) [46] and ZnCl_2_·TMEDA [81] were prepared as reported previously.

#### 3.4.1. (*tert*-Butylthio)ferrocene

To ferrocene (0.93 g, 5.0 mmol) and *t*BuOK (56 mg, 0.50 mmol) in THF (38 mL) at −80 °C was added dropwise a 1.6 M pentane solution of *t*BuLi (6.25 mL, 10 mmol). After 1 h at this temperature, di-*tert*-butyldisulfide (2.0 g, 11 mmol) was added dropwise to the red solution. The reaction mixture was then warmed to rt and stirred for 1 h. The addition of water (20 mL), extraction with EtOAc (3 × 20 mL), drying over MgSO_4_, and removal of the solvents under reduced pressure led to the crude product. This was purified by chromatography over silica gel (eluent: petroleum ether-EtOAc 90:10; Rf = 0.99) and next washed twice with 0.2 M FeCl_3_ to afford (*tert*-butylthio)ferrocene [34] in 56% yield (0.765 g) as an orange solid. Mp 76 °C. IR (ATR) *ν* 826, 896, 935, 1000, 1017, 1026, 1103, 1154, 1167, 1218, 1269, 1361, 1387, 1410, 1455, 1509, 1730, 2857, 2890, 2936, 2956, 3105 cm^−1^. ^1^H NMR (CDCl_3_) *δ* 1.20 (s, 9H, *t*Bu), 4.19 (s, 5H, Cp), 4.24 (t, 2H, *J* = 1.8 Hz, H3 and H4), 4.30 (t, 2H, *J* = 1.9 Hz, H2 and H5). ^13^C{^1^H} NMR (CDCl_3_) *δ* 30.8 (3CH_3_, C*Me*_3_), 45.1 (C, *C*Me_3_), 69.5 (5CH, Cp), 69.8 (2CH, C3 and C4), 75.9 (C, C1, *C*-SO*t*Bu), 76.3 (2CH, C2 and C5).

#### 3.4.2. Racemic *S*-*tert*-Butylferrocenesulfoxide (***rac*-FcSO*t*Bu**) 

To a solution of (*tert*-butylthio)ferrocene (0.27 g, 1.0 mmol) in CH_2_Cl_2_ (5 mL) was added portionwise, at 0 °C, 3-chloroperbenzoic acid (70%; 0.25 g, 1.0 mmol). The reaction mixture was stirred at 0 °C for 0.5 h and then warmed to rt before the addition of EtOAc (10 mL). The organic phase was washed three times with a 10% NaOH aqueous solution (3 × 10 mL). Drying over MgSO_4_ and removal of the solvents under reduced pressure led to the crude product, which was purified by chromatography over silica gel (eluent: petroleum ether-EtOAc 80:20; Rf = 0.34) to afford racemic *S*-*tert*-butylferrocenesulfoxide (***rac*-FcSO*t*Bu**) [49] in 60% yield (0.17 g) as a yellow solid. Its analyses were comparable with the ones of (*S*)-*S*-*tert*-butylferrocenesulfoxide reported previously [46]. Mp 150 °C. IR (ATR) *ν* 715, 815, 942, 1009, 1034, 1103, 1145, 1334, 1454, 2932 cm^−1^. ^1^H NMR (CDCl_3_) *δ* 1.11 (s, 9H, *t*Bu), 4.31 (m, 1H, H4), 4.33 (s, 5H, Cp), 4.36–4.37 (m, 2H, H3 and H5), 4.64 (m, 1H, H2).

#### 3.4.3. (4-Tolylthio)ferrocene

To a solution of ferrocene (9.30 g, 50.0 mmol) and potassium *tert*-butoxide (0.56 g, 5.0 mmol) in THF (375 mL) at −80 °C was added dropwise a 1.6 M hexane solution of *t*BuLi (62.5 mL, 100 mmol). The reaction mixture was stirred at −80 °C for 1 h before the addition of a solution of di-*p*-tolyl disulfide (7.8 mL, 40.0 mmol) in THF (60 mL). The reaction mixture was warmed to rt and then stirred for 1 h. Water was added and the reaction mixture was extracted with diethyl ether. The organic phase was washed three times with a 10% NaOH aqueous solution (3 × 10 mL). Drying over MgSO_4_ and removal of the solvents under reduced pressure led to the crude product, which was partially purified by column chromatography over silica gel (eluent: petroleum ether-EtOAc-CHCl_3_ 100:0:0 to 98:1:1) to give the crude product used in the oxidation step. Recrystallization of the crude can afford pure (4-tolylthio)ferrocene as an orange solid for analysis. Mp 114 °C (lit. [97] 110.5-111 °C). IR (ATR) *ν* 807, 829, 891, 998, 1052, 1105, 1166, 1410, 1491, 1644, 2923 cm^−1^. ^1^H NMR (CDCl_3_) *δ* 2.26 (s, 3H, Me), 4.26 (s, 5H, Cp), 4.32 (t, 2H, *J* = 1.9 Hz, H3 and H4), 4.40 (t, 2H, *J* = 1.8 Hz, H2 and H5), 6.98–7.01 (m, 4H, H2′, H3′, H5′ and H6′). ^13^C{^1^H} NMR (CDCl_3_) *δ* 21.0 (CH_3_, Me), 69.7 (5CH, Cp), 70.1 (2CH, C3 and C4), 74.9 (2CH, C2 and C5), 76.7 (C, C1, C-Stolyl), 126.5 (2CH, C2′ and C6′), 129.5 (2CH, C3′ and C5′), 134.9 (C, C4′), 137.1 (C, C1′). The ^1^H NMR data are as reported previously [83].

#### 3.4.4. Racemic *S*-(4-Tolyl)ferrocenesulfoxide (***rac*-FcSO-*p*-Tol**) 

To a solution of (4-tolylthio)ferrocene (0.31 g, 2.0 mmol) in CH_2_Cl_2_ (10 mL) was added portion-wise, at 0 °C, 3-chloroperbenzoic acid (70%; 0.49 g, 2.0 mmol). The reaction mixture was stirred at 0 °C for 0.5 h and then warmed to rt before the addition of EtOAc (10 mL). The organic phase was washed three times with a 10% NaOH aqueous solution (3 × 10 mL). Drying over MgSO_4_ and removal of the solvents under reduced pressure led to the crude product, which was purified by chromatography over silica gel (eluent: petroleum ether-EtOAc 80:20; Rf = 0.33) to afford racemic *S*-(4-tolyl)ferrocenesulfoxide (***rac*-FcSO-*p*-Tol**) [98] in 51% yield (0.33 g) as a yellow solid. Mp 128 °C. IR (ATR) *ν* 703, 805, 862, 895, 1011, 1031, 1043, 1081, 1106, 1162, 1248, 1304, 1386, 1355, 1491, 2973, 3075 cm^−1^. ^1^H NMR (CDCl_3_) *δ* 2.37 (s, 3H, Me), 4.32 (m, 1H, H4), 4.35–4.36 (m, 2H, H3 and H5), 4.37 (s, 5H, Cp), 4.61 (dt, 1H, *J* = 2.6 and 1.4 Hz, H2), 7.25 (d, 2H, *J* = 8.2 Hz, H3′ and H5′), 7.52 (d, 2H, *J* = 8.2 Hz, H2′ and H6′). Its analyses were comparable with the ones of (*S*)-*S*-(4-tolyl)ferrocenesulfoxide reported previously [46].

### 3.5. General Procedure A: Deprotolithiation of S-tert-Butylferrocenesulfoxides Using nBuLi Followed by Electrophilic Trapping

This was adapted from a previously reported procedure [34]. To a solution of the ferrocenesulfoxide (1.0 mmol) in THF (5 mL) at 0 °C was added dropwise a 1.4 M hexane solution of *n*BuLi (0.86 mL, 1.2 mmol). After 15 min, the reaction mixture was warmed to rt and stirred at this temperature for 1 h. The electrophile (1.5 mmol unless otherwise specified; either pure for liquids or in solution for solids, as indicated below) was next added at 0 °C. The reaction mixture was kept at 0 °C for 15 min and warmed to rt. The addition of 1 M HCl (5 mL), or saturated aqueous Na_2_S_2_O_3_ in the case of I_2_, extraction with EtOAc (3 × 20 mL), drying over MgSO_4_, and removal of the solvents under reduced pressure led to the crude product, which was purified by chromatography over silica gel (eluent given in the product description). When subsequent desilylation was performed, the protocol was as follows [64]. The silylated ferrocene (1.0 mmol) was treated by *n*Bu_4_NF (1.0 M THF solution; 1.6 mL, 2.0 mmol) in THF (5 mL) at rt for 0.5 h. The solvent was removed under reduced pressure, and the product was purified by chromatography over silica gel (eluent given in the product description).

#### 3.5.1. (*S*,*S*_P_)-*S*-*tert*-Butyl-2-(trimethylsilyl)ferrocenesulfoxide (***S*,*S*_P_-1a**)

The general procedure A from (*S*)-*S*-*tert*-butylferrocenesulfoxide (***S*-FcSO*t*Bu**; 2.9 g, 10 mmol) and using ClSiMe_3_ (1.4 mL, 11 mmol) afforded (eluent: petroleum ether-EtOAc 70:30; Rf = 0.71) the title product in 89% yield (3.2 g) as an orange solid. Mp 64–66 °C. IR (ATR) *ν* 754, 818, 948, 1002, 1045, 1107, 1173, 1242, 1362, 1408, 1457, 2953, 3097 cm^−1^. ^1^H NMR (CDCl_3_) *δ* 0.37 (s, 9H, SiMe_3_), 1.14 (s, 9H, *t*Bu), 4.31 (dd, 1H, *J* = 2.4 and 1.4 Hz, H3), 4.33 (s, 5H, Cp), 4.50–4.51 (m, 2H, H4 and H5). ^13^C{^1^H} NMR (CDCl_3_) *δ* 2.2 (3CH_3_, SiMe_3_), 23.7 (3CH_3_, C*Me*_3_), 56.1 (C, *C*Me_3_), 70.3 (5CH, Cp), 71.0 (CH, C4), 71.5 (C, C2, *C*-SiMe_3_), 72.3 (CH, C5), 77.0 (CH, C3), 91.6 (C, C1, *C*-SO*t*Bu). ${\left[\alpha \right]}_{D}^{20}$ + 20 (*c* 1.0, CHCl_3_). The IR and NMR data are similar to those previously obtained [99].

#### 3.5.2. (*R*,*R*_P_)-*S*-*tert*-Butyl-2-(trimethylsilyl)ferrocenesulfoxide (***R*,*R*_P_-1a**)

The general procedure A from (*R*)-*S*-*tert*-butylferrocenesulfoxide (***R*-FcSO*t*Bu**; 0.37 g, 1.3 mmol) and using ClSiMe_3_ (0.20 mL, 1.6 mmol) afforded (eluent: petroleum ether-EtOAc 80:20; Rf = 0.60) the title product in 53% yield (0.245 g) as an orange solid. Mp 64–66 °C. IR (ATR) *ν* 754, 818, 948, 1002, 1045, 1107, 1173, 1242, 1362, 1408, 1457, 2953, 3097 cm^−1^. ^1^H NMR (CDCl_3_) *δ* 0.37 (s, 9H, SiMe_3_), 1.14 (s, 9H, *t*Bu), 4.31 (m, 1H, H3), 4.33 (s, 5H, Cp), 4.51 (s, 2H, H4 and H5). ${\left[\alpha \right]}_{D}^{20}$ −11 (*c* 0.5, CHCl_3_). The IR and NMR data are similar to those obtained for the *S*,*S*_P_-enantiomer [99].

#### 3.5.3. (*R*,*R*_P_)- and (*S*,*S*_P_)-*S*-*tert*-Butyl-2-(trimethylsilyl)ferrocenesulfoxide (***R*,*R*_P_/*S*,*S*_P_-1a**)

The general procedure A from racemic *S*-*tert*-butylferrocenesulfoxide (***rac*-FcSO*t*Bu**; 0.29 g) and using ClSiMe_3_ (0.15 mL, 1.2 mmol) afforded (eluent: petroleum ether-EtOAc 70:30; Rf = 0.71) the title product in 88% yield (0.32 g) as an orange oil. IR (ATR) *ν* 753, 949, 1002, 1044, 1107, 1172, 1241, 1362, 1409, 1458, 2954, 3095 cm^−1^. ^1^H NMR (CDCl_3_) *δ* 0.37 (s, 9H, SiMe_3_), 1.14 (s, 9H, *t*Bu), 4.31 (m, 1H, H3), 4.33 (s, 5H, Cp), 4.51 (s, 2H, H4 and H5). The IR and NMR data are similar to those obtained for the *S*,*S*_P_-enantiomer [99].

#### 3.5.4. (*S*,*R*_P_)-*S*-*tert*-Butyl-2-iodoferrocenesulfoxide (***S*,*R*_P_-1b**)

The general procedure A from (*S*)-*S*-*tert*-butylferrocenesulfoxide (***S*-FcSO*t*Bu**; 1.45 g, 5.0 mmol) and using I_2_ (1.5 g, 6.0 mmol) in THF (5 mL) afforded (eluent: petroleum ether-EtOAc 80:20; Rf = 0.28) the title product in 85% yield (1.7 g) as a yellow solid. Mp 152 °C. IR (ATR) *ν* 820, 915, 1001, 1033, 1044, 1061, 1104, 1168, 1295, 1339, 1362, 1408, 1453, 1730, 2975, 3063 cm^−1^. ^1^H NMR (CDCl_3_) *δ* 1.24 (s, 9H, *t*Bu), 4.34 (dd, 1H, *J* = 2.7 and 1.4 Hz, H5), 4.40 (t, 1H, *J* = 2.6 Hz, H4), 4.41 (s, 5H, Cp), 4.64 (dd, 1H, *J* = 2.4 and 1.4 Hz, H3). ^13^C{^1^H} NMR (CDCl_3_) *δ* 24.2 (3CH_3_, C*Me*_3_), 32.0 (C, C2, C-I), 57.3 (C, *C*Me_3_), 70.8 (CH, C5), 71.8 (CH, C4), 73.4 (5CH, Cp), 79.0 (CH, C3), 82.5 (C, C1, *C*-SO*t*Bu). ${\left[\alpha \right]}_{D}^{20}$ +216 (*c* 1.0, CHCl_3_). Crystal data for ***S*,*R*_P_-1b.** C_14_H_17_FeIOS, *M* = 416.08; orthorhombic *P* 2_1_ 2_1_ 2_1_ (I.T.#19), *a* = 7.5042(5), *b* = 13.5729(5), *c* = 14.4725(6) Å, *V* = 1474.08(13) Å^3^. *Z* = 4, *d* = 1.875 g.·cm^−3^, *μ* = 3.244 mm^−1^. A final refinement on *F*^2^ with 3247 unique intensities and 125 parameters converged at ω*R(F^2^)* = 0.0880 (*R_F_* = 0.0359) for 3063 observed reflections with *I* > 2σ(*I*). CCDC 2152197.

#### 3.5.5. (*S*,*R*_P_)- and (*R*,*S*_P_)-*S*-*tert*-Butyl-2-iodoferrocenesulfoxide (***S*,*R*_P_/*R*,*S*_P_-1b**)

The general procedure A from racemic *S*-*tert*-butylferrocenesulfoxide (***rac*-FcSO*t*Bu**; 0.29 g) and using I_2_ (0.30 g, 1.2 mmol) in THF (1 mL) afforded (eluent: petroleum ether-EtOAc 80:20; Rf = 0.28) the title product in 84% yield (0.34 g) as a yellow solid. Mp 152 °C. IR (ATR) *ν* 726, 805, 823, 914, 1000, 1033, 1059, 1064, 1106, 1147, 1177, 1286, 1338, 1361, 1409, 1457, 1471, 1716, 2860, 2922, 3057 cm^−1^. ^1^H NMR (CDCl_3_) *δ* 1.24 (s, 9H, *t*Bu), 4.34 (dd, 1H, *J* = 2.7 and 1.4 Hz, H5), 4.40 (t, 1H, *J* = 2.6 Hz, H4), 4.42 (s, 5H, Cp), 4.64 (dd, 1H, *J* = 2.4 and 1.4 Hz, H3). The IR and NMR data are similar to those obtained for the *S*,*R*_P_ enantiomer.

#### 3.5.6. (*S*,*S*_P_)-*S*-*tert*-Butyl-2-deuterioferrocenesulfoxide (***S*,*S*_P_-1c**)

The general procedure A from (*S*)-*S*-*tert*-butylferrocenesulfoxide (***S*-FcSO*t*Bu**; 0.29 g) and using D_2_O (0.14 mL, 7.5 mmol) afforded the title product in 98% yield (0.285 g), 95% D, as a yellow solid: Rf (eluent: petroleum ether-EtOAc 80:20) = 0.18. Mp 150 °C. IR (ATR) *ν* 815, 916, 1000, 1034, 1104, 1181, 1360, 1454, 2976, 3063 cm^−1^. ^1^H NMR (CDCl_3_) *δ* 1.12 (s, 9H, *t*Bu), 4.35 (t, 1H, *J* = 2.5 Hz, H4), 4.37 (s, 5H, Cp), 4.40 (d, 1H, *J* = 2.5 Hz, H3 and H5). ^13^C{^1^H} NMR (CDCl_3_) *δ* 23.0 (3CH_3_, C*Me*_3_), 55.1 (C, *C*Me_3_), 65.4 (t, C, *J* = 27.7 Hz, C2, C-D), 69.5 (CH, C3), 69.8 (CH, C5), 70.1 (CH, C4), 70.2 (5CH, Cp), 86.5–86.8 (m, C, C1, *C*-SO*t*Bu). ${\left[\alpha \right]}_{D}^{20}$ +347 (*c* 1.0, CHCl_3_).

#### 3.5.7. (*S*,*S*_P_)-*S*-*tert*-Butyl-2-methylferrocenesulfoxide (***S*,*S*_P_-1d**)

The general procedure A from (*S*)-*S*-*tert*-butylferrocenesulfoxide (***S*-FcSO*t*Bu**; 0.29 g) and using MeI (70 μL, 1.1 mmol) afforded (eluent: petroleum ether-EtOAc 70:30; Rf = 0.35) the title product in 62% yield (0.19 g) as a yellow solid. Mp 112 °C (lit. [34] 104-106 °C). IR (ATR) *ν* 700, 743, 820, 1031, 1105, 1176, 1360, 1458, 2967, 3072 cm^−1^. ^1^H NMR (CDCl_3_) *δ* 1.17 (s, 9H, *t*Bu), 2.27 (s, 3H, Me), 4.20 (br s, 1H, H4), 4.22 (br s, 1H, H3), 4.23 (br s, 1H, H5), 4.33 (s, 5H, Cp). ^13^C{^1^H} NMR (CDCl_3_) *δ* 14.2 (CH_3_, Me), 23.5 (3CH_3_, C*Me*_3_), 56.4 (C, *C*Me_3_), 68.4 (CH, C4), 70.3 (CH, C5), 70.8 (5CH, Cp), 72.5 (CH, C3), 83.0 (C, C1, *C*-SO*t*Bu or C2, *C*-SiMe_3_), 84.8 (C, C1, *C*-SO*t*Bu or C2, *C*-SiMe_3_). ${\left[\alpha \right]}_{D}^{20}$ +211 (*c* 1.0, CHCl_3_). Crystal data for ***S*,*S*_P_-1d**. C_15_H_20_FeOS, *M* = 304.22; orthorhombic *P* 2_1_ 2_1_ 2_1_ (I.T.#19), *a* = 9.9540(10), *b* = 11.2350(15), *c* = 13.2267(15) Å, *V* = 1476.2(3) Å^3^. *Z* = 4, *d* = 1.366 g.·cm^−3^, *μ* = 1.146 mm^−1^. A final refinement on *F*^2^ with 3384 unique intensities and 110 parameters converged at ω*R(F^2^)* = 0.1649 (*R_F_* = 0.0689) for 3135 observed reflections with *I* > 2σ(*I*). These crystal data are similar to that described (CCDC 1264167; SUKXIF) [34].

#### 3.5.8. (*S*)-*S*-*tert*-Butyl-2,5-bis(trimethylsilyl)ferrocenesulfoxide (***S*-2aa**)

The general procedure A from (*S*,*S*_P_)-*S*-*tert*-butyl-2-(trimethylsilyl)ferrocenesulfoxide (***S*,*S*_P_-1a**; 0.725 g, 2.0 mmol) and using ClSiMe_3_ (0.33 mL, 2.6 mmol) afforded (eluent: petroleum ether-EtOAc 80:20; Rf = 0.87) the title product in 80% yield (0.70 g) as a yellow solid. ^1^H NMR (CDCl_3_) *δ* 0.35 (s, 9H, SiMe_3_), 0.40 (s, 9H, SiMe_3_), 1.17 (s, 9H, *t*Bu), 4.34 (s, 5H, Cp), 4.42 (d, 1H, *J* = 2.6 Hz, H3 or H4), 4.51 (d, 1H, *J* = 2.6 Hz, H3 or H4). The NMR data are similar to those obtained for the racemic product (see Section 3.5.9). Desilylation of ***S*-2aa** (0.57 g, 1.3 mmol), this time using *n*Bu_4_NF (1.6 mmol), gave back ***S*,*S*_P_-1a** in 78% yield (0.37 g).

#### 3.5.9. (*R*)- and (*S*)-*S*-*tert*-Butyl-2,5-bis(trimethylsilyl)ferrocenesulfoxide (***rac*-2aa**)

The general procedure A from (*R*,*R*_P_)/(*S*,*S*_P_)-*S*-*tert*-butyl-2-(trimethylsilyl)ferrocenesulfoxide (***R*,*R*_P_/*S*,*S*_P_-1a**; 0.36 g) and using ClSiMe_3_ (0.15 mL, 1.2 mmol) afforded (eluent: petroleum ether-EtOAc 80:20; Rf = 0.87) the title product in 63% yield (0.27 g) as a yellow solid. Mp 158 °C. IR (ATR) *ν* 718, 820, 879, 943, 1051, 1116, 1249, 1454, 2932 cm^−1^. ^1^H NMR (CDCl_3_) *δ* 0.35 (s, 9H, SiMe_3_), 0.40 (s, 9H, SiMe_3_), 1.17 (s, 9H, *t*Bu), 4.34 (s, 5H, Cp), 4.42 (d, 1H, *J* = 2.6 Hz, H3 or H4), 4.51 (d, 1H, *J* = 2.6 Hz, H3 or H4). ^13^C{^1^H} NMR (CDCl_3_) *δ* 2.2 (3CH_3_, SiMe_3_), 2.8 (3CH_3_, SiMe_3_), 24.8 (3CH_3_, C*Me*_3_), 55.7 (C, *C*Me_3_), 71.0 (5CH, Cp), 73.6 (C, C2 or C5, *C*-SiMe_3_), 79.3 (C, C2 or C5, *C*-SiMe_3_), 80.0 (CH, C3 or C4), 81.2 (CH, C3 or C4), 97.7 (C, C1, *C*-SO*t*Bu). Anal. Calc. for C_20_H_34_FeOSSi_2_ (434.57): C 55.28, H 7.89, S 7.38. Found: C 55.37, H 7.54, S 7.36. Desilylation of racemic ***rac*-2aa** (0.43 g, 1.0 mmol), this time using *n*Bu_4_NF (1.0 mmol), gave back ***R*,*R*_P_/*S*,*S*_P_-1a** in 67% yield.

#### 3.5.10. (*S*,*S*_P_)-*S*-*tert*-Butyl-2-deuterio-5-(trimethylsilyl)ferrocenesulfoxide (***S*,*S*_P_-2ac**)

The general procedure A from (*S*,*S*_P_)-*S*-*tert*-butyl-2-(trimethylsilyl)ferrocenesulfoxide (***S*,*S*_P_-1a**; 0.36 g) and using D_2_O (0.14 mL, 7.5 mmol) afforded (eluent: petroleum ether-EtOAc 70:30; Rf = 0.71) the title product in 98% yield (0.46 g), 90% D, as an orange oil. Mp 150 °C. IR (ATR) *ν* 753, 818, 834, 925, 954, 1002, 1045, 1108, 1172, 1242, 408, 1473, 2226, 2955 cm^−1^. ^1^H NMR (CDCl_3_) *δ* 0.37 (s, 9H, SiMe_3_), 1.14 (s, 9H, *t*Bu), 4.31 (d, 1H, *J* = 2.5 Hz, H3), 4.33 (s, 5H, Cp), 4.50 (d, 1H, *J* = 2.5 Hz, H4). ^13^C{^1^H} NMR (CDCl_3_) *δ* 2.2 (3CH_3_, SiMe_3_), 23.7 (3CH_3_, C*Me*_3_), 56.1 (C, *C*Me_3_), 70.3 (5CH, Cp), 70.8 (d, C, *J* = 26.9 Hz, C5, C-D), 71.5 (C, C2, *C*-SiMe_3_), 72.2 (CH, C4), 77.0 (CH, C3), 91.5 (C, C1, *C*-SO*t*Bu). ${\left[\alpha \right]}_{D}^{20}$ +56 (*c* 1.0, CHCl_3_). Desilylation of ***S*,*S*_P_-2ac** (0.34 g, 0.94 mmol) gave (eluent: petroleum ether-EtOAc 70:30; Rf = 0.33) (*S*,*R*_P_)-*S*-*tert*-butyl-2-deuterioferrocenesulfoxide (***S*,*R*_P_-1c**) in 60% yield (0.165 g) as a yellow solid. Mp 164 °C. IR (ATR) *ν* 816, 1010, 1033, 1103, 1172, 1359, 1455, 1701, 2308, 2894. ^1^H NMR (CDCl_3_) *δ* 1.11 (s, 9H, *t*Bu), 4.34 (br s, 1H, H3), 4.37 (s, 5H, Cp), 4.39 (t, 1H, *J* = 2.5 Hz, H4), 4.68 (br s, 1H, H5). ^13^C{^1^H} NMR (CDCl_3_) *δ* 22.9 (3CH_3_, C*Me*_3_), 55.1 (C, *C*Me_3_), 65.5 (CH, C5), 69.5 (CH, C4), 69.6 (t, C, *J* = 27.4 Hz, C2, C-D), 70.0 (CH, C3), 70.2 (5CH, Cp), 86.5 (C, C1, *C*-SO*t*Bu). ${\left[\alpha \right]}_{D}^{20}$ +578 (*c* 1.0, CHCl_3_).

#### 3.5.11. (*S*,*S*_P_)-*S*-*tert*-Butyl-2-iodo-5-(trimethylsilyl)ferrocenesulfoxide (***S*,*S*_P_-2ab**)

The general procedure A from (*S*,*S*_P_)-*S*-*tert*-butyl-2-(trimethylsilyl)ferrocenesulfoxide (***S*,*S*_P_-1a**; 0.725 g, 2.0 mmol) and using I_2_ (0.66 g, 2.6 mmol) in THF (2 mL) afforded (eluent: petroleum ether-EtOAc 80:20; Rf = 0.81) the titled product in 78% yield (0.76 g) as an orange-yellow oil. IR (ATR) *ν* 749, 819, 876, 896, 947, 1003, 1109, 1176, 1247, 1362, 1410, 1456, 1472, 2967 cm^−1^. ^1^H NMR (CDCl_3_) *δ* 0.37 (s, 9H, SiMe_3_), 1.27 (s, 9H, *t*Bu), 4.37 (s, 5H, Cp), 4.40 (d, 1H, *J* = 2.5 Hz, H4), 4.81 (d, 1H, *J* = 2.5 Hz, H3). ^13^C{^1^H} NMR (CDCl_3_) *δ* 2.4 (3CH_3_, SiMe_3_), 25.0 (3CH_3_, C*Me*_3_), 45.3 (C, C2), 58.1 (C, *C*Me_3_), 70.9 (C, C5), 73.9 (5CH, Cp), 79.2 (CH, C4), 80.9 (CH, C3), 92.5 (C, C1, *C*-SO*t*Bu). ${\left[\alpha \right]}_{D}^{20}$ −78 (*c* 1.0, CHCl_3_). Anal. Calc. for C_17_H_25_FeIOSSi (488.28): C 41.82, H 5.16, S 6.57. Found: C 41.46, H 4.90, S 6.19.

#### 3.5.12. (*R*,*R*_P_)-*S*-*tert*-Butyl-2-iodo-5-(trimethylsilyl)ferrocenesulfoxide (***R*,*R*_P_-2ab**)

The general procedure A from (*R*,*R*_P_)-*S*-*tert*-butyl-2-(trimethylsilyl)ferrocenesulfoxide (***R*,*R*_P_-1a**; 0.25 g, 0.70 mmol) and using I_2_ (0.21 g, 0.83 mmol) in THF (5 mL) afforded (eluent: petroleum ether-EtOAc 80:20; Rf = 0.81) the title product in 77% yield (0.26 g) as an orange-yellow oil. IR (ATR) *ν* 749, 819, 876, 896, 947, 1003, 1109, 1176, 1247, 1362, 1410, 1456, 1472, 2967 cm^−1^. ^1^H NMR (CDCl_3_) *δ* 0.37 (s, 9H, SiMe_3_), 1.27 (s, 9H, *t*Bu), 4.36 (s, 5H, Cp), 4.40 (d, 1H, *J* = 2.5 Hz, H4), 4.81 (d, 1H, *J* = 2.5 Hz, H3). The NMR data are similar to those obtained for the *S*,*S*_P_-enantiomer. ${\left[\alpha \right]}_{D}^{20}$ +78 (*c* 1.0, CHCl_3_). Desilylation of ***R*,*R*_P_-2ab** (0.26 g, 0.54 mmol) gave (eluent: petroleum ether-EtOAc 80:20; Rf = 0.10) (*R*,*R*_P_)-*S*-*tert*-butyl-2-iodoferrocenesulfoxide (***R*,*R*_P_-1b**) in 83% yield (0.19 g) as a yellow solid. Mp 150–152 °C. IR (ATR) *ν* 730, 817, 913, 1021, 1106, 1171, 1230, 1290, 1365, 1391, 1469, 2972, 3073 cm^−1^. ^1^H NMR (CDCl_3_) *δ* 1.21 (s, 9H, *t*Bu), 4.39 (s, 5H, Cp), 4.48 (t, 1H, *J* = 2.6 Hz, H4), 4.61 (dd, 1H, *J* = 2.4 and 1.4 Hz, H3), 4.69 (dd, 1H, *J* = 2.7 and 1.4 Hz, H5). ^13^C{^1^H} NMR (CDCl_3_) *δ* 23.6 (3CH_3_, C*Me*_3_), 41.8 (C, C2), 57.4 (C, *C*Me_3_), 65.8 (CH, C5), 70.8 (CH, C4), 73.4 (5CH, Cp), 77.2 (CH, C3), 89.4 (C, C1). ${\left[\alpha \right]}_{D}^{20}$ −175 (*c* 1.0, CHCl_3_). Anal. Calc. for C_14_H_17_FeIOS (416.10): C 40.41, H 4.12, S 7.70. Found: C 40.11, H 4.12, S 7.80.

#### 3.5.13. (*S*,*S*_P_)-*S*-*tert*-Butyl-2-(diphenylphosphinyl)-5-iodoferrocenesulfoxide (***S*,*S*_P_-2eb**)

The general procedure A from (*S*,*S*_P_)-*S*-*tert*-butyl-2-(diphenylphosphino)ferrocenesulfoxide (***S*,*S*_P_-1e**; 0.41 g, 0.86 mmol) and using I_2_ (0.28 g, 1.2 mmol) in THF (1 mL) afforded (eluent: petroleum ether-EtOAc 80:20; Rf = 0.35) the title product in 40% yield (0.20 g) as a brownish-yellow solid. Mp 224 °C. IR (ATR) *ν* 719, 750, 817, 829, 957, 1003, 1113, 1190, 1206, 1362, 1436, 2892 cm^−1^. ^1^H NMR (CDCl_3_) *δ* 0.97 (s, 9H, *t*Bu), 4.20 (t, 1H, *J* = 2.5 Hz, H4), 4.25 (s, 5H, Cp), 4.83 (dd, 1H, *J* = 2.75 and 2.0 Hz, H3), 7.41–7.52 (m, 6H), 7.77-7.82 (m, 4H). ^13^C{^1^H} NMR (CDCl_3_) *δ* 32.2 (3CH_3_, C*Me*_3_), 48.5 (C, C*Me*_3_), 59.6 (d, C, *J* = 8.0 Hz, C5, C-I), 74.8 (5CH, Cp), 77.6 (d, CH, *J* = 15.0 Hz, C3), 79.6 (d, CH, *J* = 10.3 Hz, C4), 85.7 (d, C, *J* = 10.7 Hz, C1, *C*-SO*t*Bu), 128.3 (d, 2CH, *J* = 11.9 Hz, C3′ and C5′), 128.4 (d, 2CH, *J* = 12.2 Hz, C3′ and C5′), 131.6 (d, CH, *J* = 2.5 Hz, C4′), 131.8 (d, CH, *J* = 2.6 Hz, C4′), 131.9 (d, 2CH, *J* = 24.0 Hz, C2′ and C6′), 132.0 (d, 2CH, *J* = 24.4 Hz, C2′ and C6′), 133.9 (d, C, *J* = 76.0 Hz, C1′), 134.7 (d, C, *J* = 75.9 Hz, C1′). The C2 peak overlaps with one of the CDCl_3_ peak. ^31^P{1H} NMR (CDCl_3_) *δ* 25.2. ${\left[\alpha \right]}_{D}^{20}$ −13 (*c* 0.5, CHCl_3_). Anal. Calc. for C_26_H_26_FeIO_2_PS (616.28): C 50.67, H 4.25, S 5.20. Found: C 50.21, H 4.13, S 5.19.

#### 3.5.14. (*S*,*R*_P_)-2-Bromo-*S*-*tert*-butyl-5-(trimethylsilyl)ferrocenesulfoxide (***S*,*R*_P_-2ja**)

The general procedure A from (*S*,*R*_P_)-*S*-*tert*-butyl-2-(trimethylsilyl)ferrocenesulfoxide (***S*,*R*_P_-1a**; 0.35 g, 0.97 mmol) and using CBr_4_ (0.42 g) in THF (5 mL) afforded (eluent: petroleum ether-EtOAc 50:50; Rf = 0.76) the title product in 65% yield (0.28 g) as an orange solid. Mp 118–120 °C. IR (ATR) *ν* 756, 836, 883, 949, 1045, 1066, 1108, 1132, 11248, 1372, 1457, 1472, 2956 cm^−1^. ^1^H NMR (CDCl_3_) *δ* 0.31 (s, 9H, SiMe_3_), 1.30 (s, 9H, *t*Bu), 4.22 (s, 1H, H4), 4.44 (s, 5H, Cp), 4.82 (s, 1H, H3). ^13^C{^1^H} NMR (CDCl_3_) *δ* 1.6 (3CH_3_, SiMe_3_), 25.3 (3CH_3_, C*Me*_3_), 58.1 (C, *C*Me_3_), 73.6 (5CH, Cp), 75.8 (C, C5, *C*-SiMe_3_), 75.9 (CH, C4), 76.0 (C, C2, C-Br), 77.4 (CH, C3), 89.3 (C, C1, *C*-SO*t*Bu). ${\left[\alpha \right]}_{D}^{20}$ +274 (*c* 0.5, CHCl_3_). Anal. Calc. for C_17_H_25_BrFeOSSi (441.28): C 46.27, H 5.71, S 7.27. Found: C 46.15, H 5.77, S 7.11.

#### 3.5.15. (*S*,*R*_P_)-*S*-*tert*-Butyl-2-iodo-5-(trimethylsilyl)ferrocenesulfoxide (***S*,*R*_P_-2ba**)

The general procedure A from (*S*,*R*_P_)-*S*-*tert*-butyl-2-(trimethylsilyl)ferrocenesulfoxide (***S*,*R*_P_-1a**; 0.39 g, 1.1 mmol) and using I_2_ (0.35 g) in THF (5 mL) afforded (eluent: petroleum ether-EtOAc 40:60; Rf = 0.89) the title product in 81% yield (425 mg) as a yellow solid. The analyses are as reported previously [46].

### 3.6. General Procedure B: Deprotolithiation of Enantiopure S-tert-Butylferrocenesulfoxides Using tBuLi Followed by Electrophilic Trapping

This was adapted from a previously reported procedure [56,57]. To a solution of the ferrocenesulfoxide (1.0 mmol) in THF (12.5 mL) at −80 °C was added dropwise a 1.6 M pentane solution of *t*BuLi (0.94 mL, 1.5 mmol), and the reaction was stirred at this temperature for 1.5 h before the addition of the electrophile (1.5 mmol unless otherwise specified; either pure for liquids or in solution for solids, as indicated below). The mixture was stirred at −80 °C for 0.5 h before being warmed to rt. The addition of 1 M HCl (5 mL), extraction with EtOAc (3 × 20 mL), drying over MgSO_4_, and removal of the solvents under reduced pressure led to the crude product, which was purified by chromatography over silica gel (eluent given in the product description).

#### 3.6.1. (*S*,*S*_P_)-*S*-*tert*-Butyl-2-(diphenylphosphino)ferrocenesulfoxide (***S*,*S*_P_-1e**)

The general procedure B from (*S*)-*S*-*tert*-butylferrocenesulfoxide (***S*-FcSO*t*Bu**; 0.435 g, 1.5 mmol) and using ClPPh_2_ (0.40 mL, 2.25 mmol) afforded (eluent: petroleum ether-EtOAc 50:50; Rf = 0.43) the title product in 92% yield (0.65 g) as a yellow solid. Mp 162 °C (lit. [37] 162-163 °C). IR (ATR) *ν* 701, 743, 818, 1041, 1072, 1177, 1359, 1433, 1472, 3080 cm^−1^. ^1^H NMR (CDCl_3_) *δ* 1.04 (s, 9H, *t*Bu), 4.16 (s, 5H, Cp), 4.25–4.26 (m, 1H, H3), 4.56 (td, 1H, *J* = 2.5 and 0.8 Hz, H4), 4.63 (dt, 1H, *J* = 2.8 and 1.6 Hz, H5), 7.23–7.25 (m, 3H, H3′ and H4′), 7.27–7.30 (m, 2H, H2′), 7.36–7.38 (m, 3H, H4′ and H5′), 7.61–7.65 (m, 2H, H6′). ^13^C{^1^H} NMR (CDCl_3_) *δ* 23.8 (d, 3CH_3_, *J* = 2.3 Hz, C*Me*_3_), 56.0 (C, *C*Me_3_), 71.5 (5CH, Cp), 72.5 (CH, C4), 73.9 (d, CH, *J* = 3.7 Hz, C5), 75.2 (d, CH, *J* = 4.9 Hz, C3), 76.6 (d, C, *J* = 25.3 Hz, C2, *C*-PPh_2_), 90.1 (d, C, *J* = 21.4 Hz, C1, *C*-SO*t*Bu), 127.8 (CH, C4′), 128.1 (d, 2CH, *J* = 11.8 Hz, C3′ and C5′), 128.1 (d, 2CH, *J* = 13.5 Hz, C3′ and C5′), 129.3 (CH, C4′), 132.9 (d, 2CH, *J* = 19.4 Hz, C2′), 135.7 (d, 2CH, *J* = 23.0 Hz, C6′), 138.8 (d, C, *J* = 14.4 Hz, C1′), 140.6 (d, C, *J* = 12.7 Hz, C1′). ^31^P{1H} NMR (CDCl_3_) *δ*-25.9. ${\left[\alpha \right]}_{D}^{20}$ +474 (*c* 1.0, CHCl_3_). The NMR data are similar to those obtained for the *R*,*R*_P_-enantiomer [36].

#### 3.6.2. (*S*,*R*_P_)-*S*-*tert*-Butyl-2-(tributylstannyl)ferrocenesulfoxide (***S*,*R*_P_-1f**)

The general procedure B from (*S*)-*S*-*tert*-butylferrocenesulfoxide (***S*-FcSO*t*Bu**; 1.45 g, 5.0 mmol) and using ClSnBu_3_ (2.0 mL) afforded (eluent: petroleum ether-EtOAc -Et_3_N 80:18:2; Rf = 0.87) the title product in 95% yield (2.8 g) as an orange oil. IR (ATR) *ν* 819, 960, 1034, 1107, 1171, 1276, 1362, 1375, 1417, 1457, 2869, 2920, 2953 cm^−1^. ^1^H NMR (CDCl_3_) *δ* 0.92 (t, 9H, *J* = 7.3 Hz, CH_2_*Me*), 1.09 (s, 9H, *t*Bu), 1.03-1.18 (m, 6H, SnCH_2_), 1.37 (h, 6H, *J* = 7.3 Hz, C*H*_2_Me), 1.50–1.63 (m, 6H, SnCH_2_C*H*_2_), 4.25 (dd, 1H, *J* = 2.1 and 1.1 Hz, H3), 4.27 (s, 5H, Cp), 4.48 (dd, 1H, *J* = 2.3 and 1.1 Hz, H5), 4.52 (t, 1H, *J* = 2.4 Hz, H4). ^13^C{^1^H} NMR (CDCl_3_) *δ* 12.5 (CH_2_, SnCH_2_), 13.9 (CH_3_, CH_2_*Me*), 23.2 (3CH_3_, C*Me*_3_), 27.8 (CH_2_, *C*H_2_Me), 29.6 (CH_2_, SnCH_2_*C*H_2_), 55.6 (C, *C*Me_3_), 67.3 (C, C2, *C*-SnBu_3_), 69.3 (CH, C5), 70.0 (5CH, Cp), 73.2 (CH, C4), 76.2 (CH, C3), 90.7 (C, C1, *C*-SO*t*Bu). ${\left[\alpha \right]}_{D}^{20}$ +98 (*c* 1.0, CHCl_3_). The ^1^H NMR data are similar to those reported previously [99].

#### 3.6.3. (*S*,*S*_P_)-*S*-*tert*-Butyl-2-[(α,α-diphenyl)hydroxymethyl]-5-(trimethylsilyl)ferrocenesulfoxide (***S*,*S*_P_-2ag**)

The general procedure B from (*S*,*S*_P_)-*S*-*tert*-butyl-2-(trimethylsilyl)ferrocenesulfoxide (***S*,*S*_P_-1a**; 0.33 g, 0.90 mmol) and using benzophenone (0.18 g, 1.35 mmol) afforded (eluent: petroleum ether-EtOAc 90:10; Rf = 0.28) the title product in 35% yield (0.17 g) as a yellow solid. Mp 190 °C. IR (ATR) *ν* 700, 756, 817, 911, 927, 1017, 1048, 1126, 1168, 1250, 1447, 2948 cm^−1^. ^1^H NMR (CDCl_3_) *δ* 0.41 (s, 9H, SiMe_3_), 0.89 (s, 9H, *t*Bu), 4.15 (br s, 1H, OH), 4.21 (s, 5H, Cp), 4.42 (br s, 1H, H3), 4.46 (d, 1H, *J* = 2.7 Hz, H4), 7.15 (t, 1H, *J* = 7.0 Hz, H4′), 7.20 (t, 2H, *J* = 7.3 Hz, H3′ or H5′), 7.23–7.28 (m, 3H, H2′ or H5′ and H4′), 7.36 (t, 2H, *J* = 7.6 Hz, H3′ or H5′), 7.56 (d, 2H, *J* = 7.4 Hz, H2′ or H5′). ^13^C{^1^H} NMR (CDCl_3_) *δ* 3.3 (3CH_3_, SiMe_3_), 25.4 (3CH_3_, C*Me*_3_), 58.6 (C, *C*Me_3_), 71.8 (5CH, Cp), 72.4 (C, C5, *C*-SiMe_3_), 76.6 (CH, C3), 78.0 (CH, C4), 79.3 (CH, CH(OH)), 91.7 (C, C1, *C*-SO*t*Bu), 101.3 (C, C2, *C*-CH(OH)), 127.0 (CH, C4′), 127.2 (4CH, C2′ and C6′), 127.3 (CH, C4′), 128.5 and 127.9 (2 × 2CH, C3′ and C5′), 146.8 (C, C1′), 148.1 (C, C1′). ${\left[\alpha \right]}_{D}^{20}$ +16 (*c* 1.0, CHCl_3_). Anal. Calc. for C_30_H_36_FeO_2_SSi (544.61): C 66.16, H 6.66, S 5.89. Found: C 66.23, H 6.82, S 5.65. In this reaction, ***S*,*S*_P_-1a** was recovered in 40% yield.

### 3.7. General Procedure C: Deprotolithiation of S-tert-Butylferrocenesulfoxides Using LiTMP Followed by Electrophilic Trapping 

This was adapted from a previously reported procedure [54]. To a stirred, cooled (−15 °C) solution of 2,2,6,6-tetramethylpiperidine (0.28 mL, 1.6 mmol) in THF (2 mL) was added dropwise a 1.4 M hexane solution of *n*BuLi (1.1 mL, 1.5 mmol). The mixture was stirred for 5 min at −15 °C and then for 2 min at −80 °C and next cannulated onto a solution of the ferrocenesulfoxide (1.0 mmol) in THF (3 mL) at −80 °C. After 1 h at this temperature, the electrophile (1.5 mmol; either pure for liquids or in solution for solids, as indicated below) was introduced at −80 °C before warming to rt. The addition of MeOH (0.5 mL) and removal of the solvents under reduced pressure led to the crude product, which was purified by chromatography over silica gel (eluent given in the product description).

#### 3.7.1. (*S*,*S*_P_)-*S*-*tert*-Butyl-2-(diphenylphosphino)-5-(trimethylsilyl)ferrocenesulfoxide (***S*,*S*_P_-2ea**)

The general procedure C from (*S*,*S*_P_)-*S*-*tert*-butyl-2-(diphenylphosphino)ferrocenesulfoxide (***S*,*S*_P_-1e**; 0.47 g) and using ClSiMe_3_ (0.19 mL) afforded (eluent: petroleum ether-EtOAc 50:50; Rf = 0.52) the title product in 70% yield (0.38 g) as a yellow solid. Mp 70 °C. IR (ATR) *ν* 742, 820, 896, 1044, 1123, 1169, 1194, 1248, 1362, 1388, 1433, 1471, 1683, 2957 cm^−1^. ^1^H NMR (CDCl_3_) *δ* 0.36 (s, 9H, SiMe_3_), 1.08 (s, 9H, *t*Bu), 4.13 (s, 5H, Cp), 4.45 (d, 1H, *J* = 2.4 Hz, H4), 4.52 (d, 1H, *J* = 2.4 Hz, H3), 7.23–7.27 (m, 3H, Ph), 7.29 (dd, 2H, *J* = 7.4 and 1.7 Hz, H2′ and/or H6′), 7.36–7.37 (m, 3H, Ph), 7.66 (td, 2H, *J* = 7.6 and 3.0 Hz, H2′ and/or H6′). ^13^C{^1^H} NMR (CDCl_3_) *δ* 1.9 (3CH_3_, SiMe_3_), 25.0 (d, 3CH_3_, *J* = 4.2 Hz, C*Me*_3_), 56.3 (C, *C*Me_3_), 72.0 (5CH, Cp), 77.8 (d, C, *J* = 27.1 Hz, C2, *C*-PPh_2_), 78.4 (d, CH, *J* = 4.4 Hz, C3), 79.6 (CH, C4), 80.5 (d, C, *J* = 2.2 Hz, C5, *C*-SiMe_3_), 96.9 (d, C, *J* = 21.3 Hz, C1, *C*-SO*t*Bu), 127.8 (CH, C4′), 128.1 (2CH, C3′ and/or C5′), 128.2 (d, 2CH, *J* = 1.8 Hz, C3′ and/or C5′), 129.4 (CH, C4′), 132.6 (d, 2CH, *J* = 19.0 Hz, C2′ and/or C6′), 136.0 (d, 2CH, *J* = 23.5 Hz, C2′ and/or C6′), 138.8 (d, C, *J* = 15.1 Hz, C1′), 141.3 (d, C, *J* = 12.1 Hz, C1′). ^31^P{1H} NMR (CDCl_3_) *δ*-24.7. ${\left[\alpha \right]}_{D}^{20}$ +395 (*c* 1.0, CHCl_3_). Anal. Calc. for C_29_H_35_FeOPSSi (546.56): C 63.73, H 6.45, S 5.87. Found: C 63.96, H 6.29, S 6.18. Using 1.1 equiv of LiTMP under the same reaction conditions led to ***S*,*S*_P_-2ea** in 49% yield, and ***S*,*S*_P_-1e** was recovered in 27% yield.

#### 3.7.2. (*S*,*S*_P_)-*S*-*tert*-Butyl-4-iodo-2-(trimethylsilyl)ferrocenesulfoxide (***S*,*S*_P_-7**)

The general procedure C, but using LiTMP (1.1 equiv) from (*S*,*R*_P_)-*S*-*tert*-butyl-2-iodoferrocenesulfoxide (***S*,*R*_P_-1b**; 0.42 g) and using ClSiMe_3_ (0.14 mL, 1.1 mmol) afforded (eluent: petroleum ether-EtOAc 70:30; Rf = 0.73) the title product in 46% yield (0.23 g) as an orange solid. Mp 144 °C. IR (ATR) *ν* 754, 820, 834, 875, 965, 1043, 1186, 1329, 1361, 1456, 2956 cm^−1^. ^1^H NMR (CDCl_3_) *δ* 0.36 (s, 9H, SiMe_3_), 1.15 (s, 9H, *t*Bu), 4.34 (s, 5H, Cp), 4.53 (d, 1H, *J* = 0.8 Hz, H3), 4.73 (d, 1H, *J* = 0.8 Hz, H5). ^13^C{^1^H} NMR (CDCl_3_) *δ* 2.1 (3CH_3_, SiMe_3_), 23.7 (3CH_3_, C*Me*_3_), 41.3 (C, C4, C-I), 56.5 (C, *C*Me_3_), 73.3 (C, C2, *C*-SiMe_3_), 73.4 (5CH, Cp), 76.4 (CH, C5), 83.2 (CH, C3), 92.1 (C, C1, *C*-SO*t*Bu). ${\left[\alpha \right]}_{D}^{20}$ -3 (*c* 0.7, CHCl_3_). Crystal data for ***S*,*S*_P_-7**. C_17_H_25_FeIOSSi, *M* = 488.27; orthorhombic *P* 2_1_ 2_1_ 2_1_ (I.T.#19), *a* = 9.243(4), *b* = 11.592(6), *c* = 18.243(8) Å, *V* = 1954.7(16) Å^3^. *Z* = 4, *d* = 1.659 g.·cm^−3^, *μ* = 2.518 mm^−1^. A final refinement on *F*^2^ with 4064 unique intensities and 205 parameters converged at ω*R(F^2^)* = 0.1322 (*R_F_* = 0.0605) for 2808 observed reflections with *I* > 2σ(*I*). CCDC 2152198. (*S*,*S*_P_)-*S*-*tert*-Butyl-2-iodo-5-(trimethylsilyl)ferrocenesulfoxide (***S*,*S*_P_-2ab**) was similarly isolated in 16% yield (79 mg). The position of the trimethylsilyl group of ***S*,*S*_P_-7** was confirmed by iodine/lithium exchange: by successively treating ***S*,*S*_P_-7** with 2 equiv *n*BuLi in THF (0 °C then rt, 1 h) and MeOH in excess, (*S*,*S*_P_)-*S*-*tert*-butyl-2-(trimethylsilyl)ferrocenesulfoxide (***S*,*S*_P_-1a**) was isolated in 77% yield. By using 1.3 equiv of LiTMP and 1.3 equiv of ClSiMe_3_ to perform the iodine migration, ***S*,*S*_P_-7** was isolated in 49% yield.

#### 3.7.3. (*S*,*S*_P_)-*S*-*tert*-Butyl-2-chloro-5-fluoro-4-methyl-3-(trimethylsilyl)ferrocenesulfoxide (***S*,*S*_P_-12**)

The general procedure C from (*S*,*R*_P_)-*S*-*tert*-butyl-5-chloro-2-fluoro-3-methylferrocenesulfoxide (***S*,*R*_P_-11k**; 0.17 g, 0.47 mmol) and using ClSiMe_3_ (90 μL, 0.70 mmol) afforded (eluent: petroleum ether-EtOAc 80:20; Rf = 0.40) the title product in 36% yield (57 mg) as a yellow solid. Mp 116 °C. IR (ATR) *ν* 759, 800, 839, 977, 1060, 1108, 1174, 1247, 1322, 1365, 1382, 1411, 1470, 2961 cm^−1^. ^1^H NMR (CDCl_3_) *δ* 0.41 (s, 9H, SiMe_3_), 1.29 (s, 9H, *t*Bu), 2.00 (s, 3H, Me), 4.47 (s, 5H, Cp). ^13^C{^1^H} NMR (CDCl_3_) *δ* 1.6 (3CH_3_, SiMe_3_), 11.6 (d, CH_3_, *J* = 2.2 Hz, Me), 23.8 (s, 3CH_3_, C*Me*_3_), 58.4 (C, *C*Me_3_), 65.9 (d, C, *J* = 1.6 Hz, C3, C-SiMe_3_), 71.7 (d, C, *J* = 12.9 Hz, C2, C-Cl), 74.6 (5CH, Cp), 75.3 (d, C, *J* = 10.0 Hz, C4, *C*-Me), 90.6 (d, C, *J* = 2.1 Hz, C1, *C*-SO*t*Bu), 134.9 (d, C, *J* = 278.9 Hz, C5, C-F). ^19^F{^1^H} NMR (CDCl_3_) *δ* −188.4. ${\left[\alpha \right]}_{D}^{20}$ +144 (*c* 1.0, CHCl_3_). Anal. Calc. for C_18_H_26_ClFFeOSSi (428.84): C 50.41, H 6.11, S 7.48. Found: C 50.19, H 6.17, S 7.31. The starting material was also recovered in 30% yield.

### 3.8. General Procedure D: Deprotolithiation of S-(4-tolyl)ferrocenesulfoxides Using LiTMP Followed by Electrophilic Trapping 

This was adapted from a previously reported procedure [54]. To a solution of the *S*-(4-tolyl)ferrocenesulfoxide (0.32 g, 1.0 mmol) in THF (3.5 mL) at −80 °C was added dropwise a solution of LiTMP [prepared at −15 °C by the addition of a 1.4 M hexane solution of *n*BuLi (0.86 mL, 1.2 mmol) to 2,2,6,6-tetramethylpiperidine (0.22 mL, 1.3 mmol) in THF followed by stirring for 5 min (1.5 mL)] cooled at −80 °C. After 0.5 h at this temperature, the electrophile (1.2 mmol unless otherwise specified; either pure for liquids or in solution for solids, as indicated below) was added. The mixture was stirred at −80 °C for 1 h before the addition of H_2_O (5 mL) or saturated aqueous Na_2_S_2_O_3_ in the case of I_2_. Extraction with EtOAc (3 × 20 mL), drying over MgSO_4_, and removal of the solvents under reduced pressure led to the crude product, which was purified by chromatography over silica gel (eluent given in the product description). When subsequent desilylation was performed, the protocol was as follows [64]. The silylated ferrocene (1.0 mmol) was treated by *n*Bu_4_NF (1.0 M THF solution; 1.6 mL, 2.0 mmol) in THF (5 mL) at rt for 0.5 h. The solvent was removed under reduced pressure, and the product was purified by chromatography over silica gel (eluent given in the product description).

#### 3.8.1. (*R*,*R*_P_)- and (*S*,*S*_P_)-*S*-(4-Tolyl)-2-(trimethylsilyl)ferrocenesulfoxide (***R*,*R*_P_/*S*,*S*_P_-3a**)

The general procedure D from racemic *S*-(4-tolyl)ferrocenesulfoxide (***rac*-FcSO-*p*-Tol**) and using ClSiMe_3_ (0.15 mL) afforded (eluent: petroleum ether-EtOAc 70:30; Rf = 0.35) the title product in 61% yield (0.24 g) as a yellow solid. Mp 162 °C. IR (ATR) *ν* 703, 783, 758, 806, 817, 859, 913, 944, 1015, 1033, 1082, 1106, 1180, 1246, 1392, 1470, 1491, 2960, 3074 cm^−1^. ^1^H NMR (CDCl_3_) *δ* 0.40 (s, 9H, SiMe_3_), 2.45 (s, 3H, C-*Me*), 3.94 (dd, 1H, *J* = 2.2 and 1.2 Hz, H5), 4.15 (s, 5H, Cp), 4.33 (dd, 1H, *J* = 2.1 and 1.3 Hz, H3), 4.39 (t, 1H, *J* = 2.4 Hz, H4), 7.35 (d, 2H, *J* = 8.0 Hz, H3′ and H5′), 7.70 (d, 2H, *J* = 8.1 Hz, H2′ and H6′). ^13^C{^1^H} NMR (CDCl_3_) *δ* 0.6 (3CH_3_, SiMe_3_), 21.6 (CH_3_, C-*Me*), 69.8 (5CH, Cp), 71.1 (CH, C5), 71.9 (CH, C4), 75.1 (C, C2, *C*-SiMe_3_), 77.6 (CH, C3), 98.9 (C, C1, *C*-SOtolyl), 125.7 (2CH, C2′ and C6′), 129.5 (2CH, C3′ and C5′), 140.5 (C, C1′), 141.5 (C, C4′). The NMR data are similar to those previously obtained for (*S*,*S*_P_)-*S*-(4-tolyl)-2-(trimethylsilyl)ferrocenesulfoxide (***S*,*S*_P_-3a**) [37]. Using 1.5 equiv of LiTMP led to the competitive formation of (*R*,*R*_P_)- and (*S*,*S*_P_)-*S*-[4-methyl-2-(trimethylsilyl)phenyl]-2-(trimethylsilyl)ferrocenesulfoxide (***R*,*R*_P_/*S*,*S*_P_-3a′**), isolated (eluent: petroleum ether-EtOAc 70:30; Rf = 0.61) in 19% yield (92 mg) as an orange solid. Mp 145 °C. IR (ATR) *ν* 753, 820, 873, 1001, 1055, 1105, 1182, 1243, 1408, 1582, 2950 cm^−1^. ^1^H NMR (CDCl_3_) *δ* 0.45 (s, 9H, C2-SiMe_3_), 0.45 (s, 9H, C2′-SiMe_3_), 2.36 (s, 3H, Me), 3.89 (dd, 1H, *J* = 2.3 and 1.4 Hz, H3 or H4 or H5), 4.29 (s, 5H, Cp), 4.32–4.34 (m, 2H, H3 and/or H4 and/or H5), 7.21 (dd, 1H, *J* = 8.0 and 1.2 Hz, H5′), 7.37 (d, 1H, *J* = 1.2 Hz, H3′), 7.46 (d, 1H, *J* = 8.0 Hz, H6′). ^13^C{^1^H} NMR (CDCl_3_) *δ* 1.5 (3CH_3_, SiMe_3_), 1.6 (3CH_3_, SiMe_3_), 21.7 (CH_3_, C-*Me*), 70.0 (5CH, Cp), 70.2 (C3 or C4 or C5), 71.8 (C3 or C4 or C5), 73.1 (C, C2, *C*-SiMe_3_), 76.3 (C3 or C4 or C5), 97.9 (C, C1, *C*-SOtolyl), 127.6 (CH, C6′), 131.0 (CH, C5′), 135.5 (CH, C3′), 139.9 (C, C4′), 140.6 (C, C2′), 150.5 (C, C1′). Anal. Calc. for C_23_H_32_FeOSSi_2_ (468.58): C 50.95, H 6.88, S 6.84. Found: C 51.06, H 7.10, S 7.04. Under these conditions, ***R*,*R*_P_/*S*,*S*_P_-3a** was only obtained in 43% yield (0.17 g).

#### 3.8.2. (*R*,*S*_P_)- and (*S*,*R*_P_)-2-Iodo-*S*-(4-tolyl)ferrocenesulfoxide (***R*,*S*_P_/*S*,*R*_P_-3b**)

The general procedure D (but using 1.5 equiv of LiTMP) from racemic *S*-(4-tolyl)ferrocenesulfoxide (***rac*-FcSO-*p*-Tol**; 0.22 g, 0.68 mmol) and using I_2_ (0.26 g, 1.0 mmol) in THF (2 mL) afforded (eluent: petroleum ether-EtOAc 70:30; Rf = 0.26) the title product in 81% yield (0.24 g) as a yellow solid. Mp 226 °C. IR (ATR) *ν* 808, 826, 909, 1028, 1081, 1181, 1410, 1490, 3074 cm^−1^. ^1^H NMR (CDCl_3_) *δ* 2.44 (s, 3H, Me), 4.07 (dd, 1H, *J* = 2.4 and 1.2 Hz, H5), 4.23 (s, 5H, Cp), 4.35 (t, 1H, *J* = 2.6 Hz, H4), 4.66 (dd, 1H, *J* = 2.5 and 1.3 Hz, H3), 7.35 (d, 2H, *J* = 8.0 Hz, H3′ and H5′), 7.71 (d, 2H, *J* = 8.1 Hz, H2′ and H6′). ^13^C{^1^H} NMR (CDCl_3_) *δ* 21.6 (CH_3_), 39.6 (C, C2, C-I), 68.7 (CH, C5), 71.1 (CH, C4), 72.8 (5CH, Cp), 78.2 (CH, C3), 94.2 (C, C1, *C*-SOtolyl), 125.7 (2CH, C2′ and C6′), 129.6 (2CH, C3′ and C5′), 140.1 (C, C1′), 141.7 (C, C4′). The NMR data are similar to those previously obtained for (*S*,*R*_P_)-2-iodo-*S*-(4-tolyl)ferrocenesulfoxide (***S*,*R*_P_-3b**) [100]. Using 1.1 equiv of LiTMP led to the recovery of starting ***rac*-FcSO-*p*-Tol** (20% yield) while ***R*,*S*_P_/*S*,*R*_P_-3b** was only obtained in 51% yield.

#### 3.8.3. (*S*,*S*_P_)-2-Deuterio-*S*-(4-tolyl)ferrocenesulfoxide (***S*,*S*_P_-3c**)

The general procedure D (but using 2 equiv of LiTMP) from 0.60 mmol of (*S*)-*S*-(4-tolyl)ferrocenesulfoxide (***S*-FcSO-*p*-Tol**; 0.195 g) and using concentrated DCl (0.13 mL) afforded (eluent: petroleum ether-EtOAc 40:60; Rf = 0.59) the title product in a quantitative yield (0.195 g), 80% D, as a yellow solid, and identified by NMR: ^1^H NMR (CDCl_3_) *δ* 2.37 (s, 3H, Me), 4.32 (t, 1H, *J* = 2.5 Hz, H4), 4.35–4.38 (m, 2H, H3 and H5), 4.37 (s, 5H, Cp), 7.25 (d, 2H, *J* = 8.4 Hz, H3′ and H5′), 7.52 (d, 2H, *J* = 8.2 Hz, H2′ and H6′). ^13^C{^1^H} NMR (CDCl_3_) *δ* 21.5 (CH_3_, Me), 65.3 (t, C, *J* = 27.7 Hz, C2, C-D), 67.9 (CH, C3 or C5), 70.0 (CH, C3, C4 or C5), 70.0 (5CH, Cp), 70.1 (CH, C3, C4 or C5), 94.7 (C, C1, *C*-SOtolyl), 124.5 (2CH, C2′ and C6′), 129.7 (2CH, C3′ and C5′), 141.1 (C, C4′), 143.1 (C, C1′). ${\left[\alpha \right]}_{D}^{20}$ +344 (*c* 1.0, CHCl_3_). The mp and IR spectra are similar to those recorded for ***S*-FcSO-*p*-Tol**.

#### 3.8.4. (*R*,*R*_P_)- and (*S*,*S*_P_)-*S*-(2-Iodo-4-tolyl)-2-(trimethylsilyl)ferrocenesulfoxide (***R*,*R*_P_/*S*,*S*_P_-4**)

The general procedure D (but using 1.8 equiv of LiTMP) from racemic (*R*,*R*_P_)- and (*S*,*S*_P_)-*S*-(4-tolyl)-2-(trimethylsilyl)ferrocenesulfoxide (***R*,*R*_P_/*S*,*S*_P_-3a**; 0.25 g, 0.64 mmol) and using I_2_ (0.29 g, 1.2 mmol) in THF (3.0 mL) afforded (eluent: petroleum ether-EtOAc 80:20; Rf = 0.35) the title product in 58% yield (0.195 g) as a yellow solid. Mp 154 °C. IR (ATR) *ν* 751, 822, 941, 1014, 1044, 1107, 1177, 1244, 1439, 1582, 2951 cm^−1^. ^1^H NMR (CDCl_3_) *δ* 0.41 (s, 9H, SiMe_3_), 2.37 (s, 3H, Me), 4.24 (dd, 1H, *J* = 2.5 and 1.3 Hz, H5), 4.28 (s, 5H, Cp), 4.34 (dd, 1H, *J* = 2.5 and 1.3 Hz, H3), 4.42 (t, 1H, *J* = 2.4 Hz, H4), 7.34 (d, 1H, *J* = 8.1 Hz, H5′), 7.71 (s, 1H, H3′), 7.75 (d, 1H, *J* = 7.9 Hz, H6′). ^13^C{^1^H} NMR (CDCl_3_) *δ* 0.9 (3CH_3_, SiMe_3_), 21.0 (CH_3_, C-*Me*), 70.3 (5CH, Cp), 71.4 (CH, C5), 71.9 (CH, C4), 74.5 (C, C2, *C*-SiMe_3_), 77.4 (CH, C3), 94.6 (C, C2′, C-I), 98.7 (C, C1, *C*-SOAr), 128.6 (CH, C6′), 130.2 (CH, C5′), 139.9 (CH, C3′), 143.6 (C, C4′), 145.9 (C, C1′). Anal. Calc. for C_20_H_23_FeIOSSi (522.30): C 45.99, H 4.44, S 6.14. Found: C 46.11, H 4.27, S 6.24. Under these conditions, starting ***R*,*R*_P_/*S*,*S*_P_-3a** was recovered in 28% yield. Desilylation of ***R*,*R*_P_/*S*,*S*_P_-4** (0.16 g, 0.30 mmol) gave (eluent: petroleum ether-EtOAc 80:20; Rf = 0.18) racemic *S*-(2-iodo-4-tolyl)ferrocenesulfoxide (***rac*-5**) in a quantitative yield (0.14 g) as a yellow solid. Mp 178 °C. IR (ATR) *ν* 754, 819, 877, 1012, 1058, 1092, 1159, 1249, 1413, 1457, 1583, 3200 cm^−1^. ^1^H NMR (CDCl_3_) *δ* 2.29 (s, 3H, Me), 4.31 (t, 2H, *J* = 2.0 Hz, H3 and H4), 4.43 (s, 5H, Cp), 4.51 (q, 1H, *J* = 1.7 Hz, H2 or H5), 4.87 (q, 1H, *J* = 1.7 Hz, H2 or H5), 7.31 (d, 1H, *J* = 8.0 Hz, H5′), 7.56 (s, 1H, H3′), 7.76 (d, 1H, *J* = 8.0 Hz, H6′). ^13^C{^1^H} NMR (CDCl_3_) *δ* 20.9 (CH_3_, C-*Me*), 64.4 (CH, C2 or C5), 69.5 (CH, C3 or C4), 69.8 (CH, C3 or C4), 69.9 (CH, C2 or C5), 70.2 (5CH, Cp), 92.9 (C, C2′, C-I), 94.8 (C, C1, *C*-SOAr), 125.6 (CH, C6′), 130.3 (CH, C5′), 139.8 (CH, C3′), 142.9 (C, C4′), 146.5 (C, C1′). Anal. Calc. for C_17_H_15_FeIOS (450.12): C 45.36, H 3.36, S 7.12. Found: C 45.78, H 3.59, S 6.87.

#### 3.8.5. (*R*,*S*_P_)- and (*S*,*R*_P_)-2-Iodo-*S*-(4-tolyl)-5-(trimethylsilyl)ferrocenesulfoxide (***R*,*S*_P_/*S*,*R*_P_-6**)

The general procedure D from racemic (*R*,*S*_P_)- and (*S*,*R*_P_)-2-iodo-*S*-(4-tolyl)ferrocenesulfoxide (***R*,*S*_P_/*S*,*R*_P_-3b**; 0.28 g, 0.62 mmol) and using ClSiMe_3_ (90 μL, 0.68 mmol) afforded (eluent: petroleum ether-EtOAc 70:30; Rf = 0.89) the title product in 11% yield (37 mg) as a yellow oil. IR (ATR) *ν* 752, 821, 1003, 1051, 1082, 1108, 1119, 1186, 1243, 1368, 1410, 1491, 1595, 2951 cm^−1^. ^1^H NMR (CDCl_3_) *δ* 0.08 (s, 9H, SiMe_3_), 2.34 (s, 3H, Me), 4.41 (d, 1H, *J* = 2.5 Hz, H4), 4.44 (s, 5H, Cp), 4.78 (d, 1H, *J* = 2.5 Hz, H3), 7.19 (d, 2H, *J* = 8.0 Hz, H3′ and H5′), 7.35 (d, 1H, *J* = 8.2 Hz, H2′ and H6′). ^13^C{^1^H} NMR (CDCl_3_) *δ* 1.0 (3CH_3_, SiMe_3_), 21.4 (CH_3_, C-*Me*), 45.9 (C, C2, C-I), 71.1 (C, C5, *C*-SiMe_3_), 73.3 (5CH, Cp), 78.3 (CH, C4), 79.0 (CH, C3), 97.6 (C, C1, *C*-SOtolyl), 125.6 (2CH, C2′ and C6′), 129.5 (2CH, C3′ and C5′), 140.4 (C, C1′), 143.3 (C, C4′). Anal. Calc. for C_20_H_23_FeIOSSi (522.30): C 45.99, H 4.44, S 6.14. Found: C 46.04, H 4.53, S 6.15. Under these conditions, starting ***R*,*S*_P_/*S*,*R*_P_-3b** was recovered in 80% yield.

### 3.9. General Procedure E: One-Pot Deprotolithiation-Trimethylsilylation-Deprotolithiation-Trapping of S-tert-Butylferrocenesulfoxides

To a solution of *S*-*tert*-butylferrocenesulfoxide (***S*-FcSO*t*Bu** or ***R*-FcSO*t*Bu**; 0.29 g, 1.0 mmol) in THF (5 mL) at 0 °C was added dropwise a 1.4 M hexane solution of *n*BuLi (0.79 mL, 1.1 mmol). After 15 min, the mixture was warmed to rt and stirred at this temperature for 1 h. ClSiMe_3_ (0.14 mL, 1.1 mmol) was introduced at 0 °C and, after 15 min, the mixture was warmed to rt and stirred at this temperature for 1 h. To this mixture, cooled at 0 °C, was next added dropwise a 1.4 M hexane solution of *n*BuLi (1.1 mL, 1.5 mmol). After 15 min at 0 °C, the mixture was warmed to rt and stirred at this temperature for 1 h. The electrophile (either pure for liquids or in solution for solids, as indicated below) was next added at 0 °C. The mixture was kept at 0 °C for 15 min and warmed to rt. The addition of 1 M HCl (5 mL), or saturated aqueous Na_2_S_2_O_3_ in the case of I_2_, extraction with EtOAc (3 × 20 mL), drying over MgSO_4_, and removal of the solvents under reduced pressure led to the crude product, which was purified by chromatography over silica gel (eluent given in the product description). When subsequent desilylation was performed, the protocol was as follows [64]. The silylated ferrocene (1.0 mmol) was treated by *n*Bu_4_NF (1.0 M THF solution; 1.6 mL, 2.0 mmol) in THF (5 mL) at rt for 0.5 h. The solvent was removed under reduced pressure, and the product was purified by chromatography over silica gel (eluent given in the product description).

#### 3.9.1. (*S*,*S*_P_)-*S*-*tert*-Butyl-2-iodoferrocenesulfoxide (***S*,*S*_P_-2ab**)

The general procedure E, from ***S*-FcSO*t*Bu**, using I_2_ (0.38 g, 1.5 mmol) in THF (3 mL), in situ followed by desilylation, this time using *n*Bu_4_NF (8.0 mmol), afforded (eluent: petroleum ether-EtOAc 80:20; Rf = 0.10) the title product in 83% yield (0.19 g) as a yellow solid. Mp 154–156 °C. IR (ATR) *ν* 807, 891, 1033, 1410, 1491, 2969 cm^−1^. ^1^H NMR (CDCl_3_) *δ* 1.22 (s, 9H, *t*Bu), 4.40 (s, 5H, Cp), 4.49 (t, 1H, *J* = 2.6 Hz, H4), 4.62 (dd, 1H, *J* = 2.4 and 1.4 Hz, H3), 4.70 (dd, 1H, *J* = 2.7 and 1.4 Hz, H5). The IR and NMR data are similar to those obtained for the *R*,*R*_P_-enantiomer. ${\left[\alpha \right]}_{D}^{20}$ +175 (*c* 1.0, CHCl_3_).

#### 3.9.2. (*R*,*R*_P_)-*S*-*tert*-Butyl-2-iodo-5-(trimethylsilyl)ferrocenesulfoxide (***R*,*R*_P_-2ab**)

The general procedure E, from ***R*-FcSO*t*Bu**, using I_2_ (0.38 g, 1.5 mmol) in THF (3 mL) afforded (eluent: petroleum ether-EtOAc 80:20; Rf = 0.82) the title product in 53% yield (0.26 g). The analyses are as described above (see Section 3.5.12).

#### 3.9.3. (*S*,*S*_P_)-*S*-*tert*-Butyl-2-methyl-5-(trimethylsilyl)ferrocenesulfoxide (***S*,*S*_P_-2ad**)

The general procedure E, from ***S*-FcSO*t*Bu**, using MeI (88 μL, 1.5 mmol) afforded (eluent: petroleum ether-EtOAc 80:20; Rf = 0.67) the title product in 74% yield (0.28 g) as an orange solid. Mp 92 °C. IR (ATR) *ν* 759, 812, 834, 917, 1014, 1043, 1129, 1239, 2962 cm^−1^. ^1^H NMR (CDCl_3_) *δ* 0.36 (s, 9H, SiMe_3_), 1.16 (s, 9H, *t*Bu), 2.14 (s, 3H, Me), 4.22 (d, 1H, *J* = 2.5 Hz, H4), 4.46 (d, 1H, *J* = 2.5 Hz, H3). ^13^C{^1^H} NMR (CDCl_3_) *δ* 2.6 (3CH_3_, SiMe_3_), 15.9 (CH_3_, Me), 24.1 (3CH_3_, C*Me*_3_), 57.1 (C, *C*Me_3_), 70.2 (C, C5, *C*-SiMe_3_), 71.3 (5CH, Cp), 75.1 (CH, C3), 76.6 (CH, C4), 89.4 (C, C2, *C*-Me), 91.4 (C, C1, *C*-SO*t*Bu). Anal. Calc. for C_18_H_28_FeOSSi (376.41): C 57.44, H 7.50, S 8.52. Found: C 57.57, H 7.52, S 8.56. Desilylation of ***S*,*S*_P_-2ad** (0.11 g, 0.30 mmol) gave (eluent: petroleum ether-EtOAc 80:20; Rf = 0.24) (*S*,*R*_P_)-*S*-*tert*-butyl-2-methylferrocenesulfoxide (***S*,*R*_P_-1d**) in a quantitative yield (91 mg) as an orange oil. IR (ATR) *ν* 750, 812, 956, 1002, 1038, 1106, 1171, 1238, 1362, 1456, 1727, 2974 cm^−1^. ^1^H NMR (CDCl_3_) *δ* 1.14 (s, 9H, *t*Bu), 2.07 (s, 3H, Me), 4.26 (dd, 1H, *J* = 2.4 and 1.5 Hz, H3), 4.28–4.30 (m, 1H, H4), 4.29 (s, 5H, Cp), 4.58 (dd, 1H, *J* = 2.6 and 1.5 Hz, H5). ^13^C{^1^H} NMR (CDCl_3_) *δ* 14.5 (CH_3_, Me), 23.1 (3CH_3_, C*Me*_3_), 56.2 (C, *C*Me_3_), 65.1 (CH, C5), 68.2 (CH, C4), 70.8 (5CH, Cp), 71.5 (CH, C3), 86.4 (C, C1 or C2), 86.6 (C, C1 or C2). ${\left[\alpha \right]}_{D}^{20}$ +325 (*c* 0.8, CHCl_3_). Anal. Calc. for C_15_H_20_FeOS (304.23): C 59.22, H 6.63, S 10.54. Found: C 59.12, H 6.66, S 10.52.

#### 3.9.4. (*S*,*R*_P_)-*S*-*tert*-Butyl-2-(diphenylphosphino)-5-(trimethylsilyl)ferrocenesulfoxide (***S*,*R*_P_-2ae**)

The general procedure E, from ***S*-FcSO*t*Bu**, using ClPPh_2_ (0.27 mL, 1.5 mmol) afforded (eluent: petroleum ether-EtOAc 80:20; Rf = 0.71) the title product in 48% yield (0.22 g) as a yellow oil. IR (ATR) *ν* 721, 750, 1037, 1119, 1248, 1437, 2956 cm^−1^. ^1^H NMR (CDCl_3_) *δ* 0.41 (s, 9H, SiMe_3_), 1.02 (s, 9H, *t*Bu), 4.06 (s, 5H, Cp), 4.57 (d, 1H, *J* = 2.6 Hz, H4), 4.59 (d, 1H, *J* = 2.6 Hz, H3), 7.21–7.32 (m, 5H, Ph), 7.38-7.39 (m, 3H, Ph), 7.65–7.69 (m, 2H, Ph). ^13^C{^1^H} NMR (CDCl_3_) *δ* 2.8 (3CH_3_, SiMe_3_), 24.8 (d, 3CH_3_, *J* = 3.3 Hz, C*Me*_3_), 57.0 (C, *C*Me_3_), 71.8 (5CH, Cp), 74.0 (C, C5, *C*-SiMe_3_), 77.7 (d, CH, *J* = 4.0 Hz, C3), 80.4 (CH, C4), 81.7 (d, C, *J* = 18.3 Hz, C2, *C*-PPh_2_), 99.1 (d, C, *J* = 24.8 Hz, C1, *C*-SO*t*Bu), 128.1, 128.2, 128.3, 128.3, 128.4 and 129.7 (6CH, C3′, C4′ and C5′), 132.6 and 135.7 (d, 2CH, *J* = 18.7 Hz and d, 2CH, *J* = 23.3 Hz; C2′ and C6′), 138.3 and 140.0 (d, C, *J* = 10.0 Hz and d, C, *J* = 8.4 Hz; C1′). ^31^P{1H} NMR (CDCl_3_) *δ* −25.8. ${\left[\alpha \right]}_{D}^{20}$ +123 (*c* 1.0, CHCl_3_). Anal. Calc. for C_29_H_35_FeOPSSi (546.56): C 63.73, H 6.45, S 5.87. Found: C 63.76, H 6.10, S 5.83.

#### 3.9.5. (*S*,*S*_P_)-*S*-*tert*-Butyl-2-[(α,α-diphenyl)hydroxymethyl]-5-(trimethylsilyl)ferrocenesulfoxide (***S*,*S*_P_-2ag**)

The general procedure E, from ***S*-FcSO*t*Bu**, using benzophenone (0.27 g, 1.5 mmol) afforded (eluent: petroleum ether-EtOAc 80:20; Rf = 0.40) the title product in 51% yield (0.30 g) as a yellow solid. ^1^H NMR (CDCl_3_) *δ* 0.42 (s, 9H, SiMe_3_), 0.89 (s, 9H, *t*Bu), 4.14 (br s, 1H, OH), 4.21 (s, 5H, Cp), 4.42 (br s, 1H, H3), 4.46 (d, 1H, *J* = 2.7 Hz, H4), 7.15–7.26 (m, 6H, ArH), 7.36 (t, 2H, *J* = 7.6 Hz, H3′ or H5′), 7.56 (d, 2H, *J* = 7.4 Hz, H2′ or H5′). The NMR data are similar to those obtained previously (see Section 3.6.3). ${\left[\alpha \right]}_{D}^{20}$ +16 (*c* 1.0, CHCl_3_).

#### 3.9.6. (*S*,*S*_P_)-*S*-*tert*-Butyl-2-(dimethylaminomethyl)-5-(trimethylsilyl)ferrocenesulfoxide (***S*,*S*_P_-2ah**)

The general procedure E, from ***S*-FcSO*t*Bu**, using *N*,*N*-dimethylmethyleneiminium iodide (0.28 g, 1.5 mmol) afforded (eluent: petroleum ether-EtOAc -NEt_3_ 72:18:10; Rf = 0.80) the title product in 66% yield (0.24 g) as an orange oil. IR (ATR) *ν* 753, 817, 835, 920, 954, 999, 1044, 1070, 1123, 1146, 1172, 1247, 1363, 1390, 1455, 1677, 2766, 2815, 2951 cm^−1^. ^1^H NMR (CDCl_3_) *δ* 0.38 (s, 9H, SiMe_3_), 1.22 (s, 9H, *t*Bu), 2.16 (s, 6H, NMe_2_), 3.09 (d, 1H, *J* = 13.0 Hz, C*H*HNMe_2_), 3.66 (d, 1H, *J* = 13.1 Hz, CH*H*NMe_2_), 4.28 (d, 1H, *J* = 2.5 Hz, H4), 4.29 (s, 5H, Cp), 4.57 (d, 1H, *J* = 2.5 Hz, H3). ^13^C{^1^H} NMR (CDCl_3_) *δ* 2.5 (3CH_3_, SiMe_3_), 24.9 (3CH_3_, C*Me*_3_), 45.4 (2CH_3_, NMe_2_), 56.6 (C, *C*Me_3_), 58.5 (CH_2_, CH_2_NMe_2_), 71.0 (C, C5, *C*-SiMe_3_), 71.3 (5CH, Cp), 76.1 (CH, C3), 76.9 (CH, C4), 91.3 (C, C1, *C*-SO*t*Bu), 93.2 (C, C2, *C*-CH_2_NMe_2_). ${\left[\alpha \right]}_{D}^{20}$ +284 (*c* 1.0, CHCl_3_). Anal. Calc. for C_20_H_33_FeNOSSi (419.48): C 57.27, H 7.93, N 3.34, S 7.64. Found: C 57.35, H 8.06, N 3.28, S 7.70. (*S*,*S*_P_)-*S*-*tert*-Butyl-2-formyl-5-(trimethylsilyl)ferrocenesulfoxide (***S*,*S*_P_-2ah’**) was also obtained (eluent: petroleum ether-EtOAc-NEt_3_ 72:18:10; Rf = 0.66) in 23% yield as an orange oil and identified by NMR: ^1^H NMR (CDCl_3_) *δ* 0.40 (s, 9H, SiMe_3_), 1.15 (s, 9H, *t*Bu), 4.45 (s, 5H, Cp), 4.77 (d, 1H, *J* = 2.7 Hz, H4), 5.07 (d, 1H, *J* = 2.7 Hz, H3), 10.1 (s, 1H, CHO). Desilylation of ***S*,*S*_P_-2ah** (0.11 g, 0.26 mmol) gave (eluent: petroleum ether-EtOAc-Et_3_N 72:18:10; Rf = 0.30) (*S*,*R*_P_)-*S*-*tert*-butyl-2-(dimethylaminomethyl)ferrocenesulfoxide (***S*,*R*_P_-1h**) in a quantitative yield (91 mg) as an orange solid. Mp 94–96 °C. IR (ATR) *ν* 753, 1050, 1247, 1457, 1677, 1903, 2300, 2951 cm^−1^. ^1^H NMR (CDCl_3_) *δ* 1.19 (s, 9H, *t*Bu), 2.15 (s, 6H, NMe_2_), 3.06 (d, 1H, *J* = 13.0 Hz, C*H*H), 3.60 (d, 1H, *J* = 13.0 Hz, CH*H*), 4.32 (s, 5H, Cp), 4.37 (s, 1H, H3 or H4), 4.37 (s, 1H, H3 or H4), 4.64 (s, 1H, H5). ^13^C{^1^H} NMR (CDCl_3_) *δ* 23.6 (3CH_3_, C*Me*_3_), 45.4 (CH_3_, NMe_2_), 56.3 (C, *C*Me_3_), 57.9 (CH_2_), 65.9 (CH, C5), 68.7 (CH, C4), 70.9 (5CH, Cp), 72.4 (CH, C3), 88.2 (C, C1 or C2), 88.5 (C, C1 or C2). ${\left[\alpha \right]}_{D}^{20}$ +36 (*c* 0.5, CHCl_3_). Anal. Calc. for C_17_H_25_FeNOS (347.30): C 58.79, H 7.26, N 4.03, S 9.23. Found: C 59.18, H 7.32, N 3.92, S 9.25.

#### 3.9.7. (*S*,*S*_P_)-*S*-*tert*-Butyl-2-fluoro-5-(trimethylsilyl)ferrocenesulfoxide (***S*,*S*_P_-2ai**)

The general procedure E, from ***S*-FcSO*t*Bu** and at a 5.0 mmol scale, using *N*-fluorobenzenesulfonimide (2.35 g, 7.5 mmol) afforded (eluent: petroleum ether-EtOAc 80:20; Rf = 0.83) the title product in 67% yield (1.27 g) as an orange solid. Mp 104 °C. IR (ATR) *ν* 757, 818, 833, 914, 1003, 1042, 1109, 1123, 1176, 1242, 1365, 1391, 1452, 1517, 2957 cm^−1^. ^1^H NMR (CDCl_3_) *δ* 0.34 (s, 9H, SiMe_3_), 1.18 (s, 9H, *t*Bu), 3.98 (t, 1H, *J* = 2.5 Hz, H4), 4.43 (s, 5H, Cp), 4.68 (t, 1H, *J* = 3.0 Hz, H3). ^13^C{^1^H} NMR (CDCl_3_) *δ* 2.1 (3CH_3_, SiMe_3_), 23.6 (3CH_3_, C*Me*_3_), 57.5 (C, *C*Me_3_), 60.9 (d, CH, *J* = 14.5 Hz, C3), 65.2 (d, C, *J* = 2.5 Hz, C5, *C*-SiMe_3_), 69.2 (d, CH, *J* = 5.1 Hz, C4), 71.8 (5CH, Cp), 80.3 (d, C, *J* = 8.9 Hz, C1, *C*-SO*t*Bu), 137.1 (d, C, *J* = 273.9 Hz, C2, C-F). ^19^F{^1^H} NMR (CDCl_3_) *δ*-180.3. ${\left[\alpha \right]}_{D}^{20}$ +118 (*c* 1.0, CHCl_3_). Anal. Calc. for C_17_H_25_FFeOSSi (380.37): C 53.68, H 6.63, S 8.43. Found: C 53.66, H 6.61, S 8.48. Reduction [82] of (*S*,*S*_P_)-*S*-*tert*-butyl-2-fluoro-5-(trimethylsilyl)ferrocenesulfoxide (***S*,*S*_P_-2ai**) was performed as follows. To a solution of ***S*,*S*_P_-2ai** (0.22 g, 0.58 mmol) and NaI (0.34 g, 2.3 mmol) in acetone (3 mL) at 0 °C was added dropwise a solution of trifluoroacetic anhydride (0.32 mL, 2.3 mmol) in acetone (6 mL). After 5 min at 0 °C, a saturated aqueous solution of Na_2_S_2_O_3_ (5 mL) was added. Extraction by EtOAc (3 × 10 mL), washing by water (10 mL), drying over MgSO_4_, and removal of the solvents under reduced pressure led to the crude product. Purification by chromatography over silica gel (eluent: petroleum ether-EtOAc 80:20; Rf = 0.91) gave (*S*_P_)-2-(*tert*-butylthio)-1-fluoro-3-(trimethylsilyl)ferrocene (***S*_P_-8ai**) in a quantitative yield (0.22 g) as an orange solid identified by NMR: ^1^H NMR (CDCl_3_) *δ* 0.32 (s, 9H, SiMe_3_), 1.25 (s, 9H, *t*Bu), 3.82 (t, 1H, *J* = 2.3 Hz, H4), 4.23 (s, 5H, Cp), 4.65 (t, 1H, *J* = 3.1 Hz, H3). Desilylation of ***S*,*S*_P_-2ai** (32 mg, 80 μmol) gave (eluent: petroleum ether-EtOAc 80:20; Rf = 0.32) (*S*,*R*_P_)-*S*-*tert*-butyl-2-fluoroferrocenesulfoxide (***S*,*R*_P_-1i**) in a quantitative yield (25 mg) as an orange oil. IR (ATR) *ν* 824, 985, 1044, 1164, 1363, 1407, 1449, 1647, 2961, 3098, 3474 cm^−1^. ^1^H NMR (CDCl_3_) *δ* 1.16 (s, 9H, *t*Bu), 4.07 (dd, 1H, *J* = 4.3 and 2.7 Hz, H4), 4.33 (t, 1H, *J* = 2.3 Hz, H5), 4.47 (s, 5H, Cp), 4.49 (dd, 1H, *J* = 4.7 and 3.0 Hz, H4). ^13^C{^1^H} NMR (CDCl_3_) *δ* 22.7 (3CH_3_, C*Me*_3_), 56.3 (C, *C*Me_3_), 58.0 (d, CH, *J* = 14.2 Hz, C3), 59.4 (CH, C5), 61.9 (d, CH, *J* = 3.5 Hz, C4), 71.6 (5CH, Cp), 75.1 (d, C, *J* = 13.5 Hz, C1, *C*-SO*t*Bu), 136.2 (d, C, *J* = 273.2 Hz, C2, C-F). ^19^F{^1^H} NMR (CDCl_3_) *δ* −188.0. ${\left[\alpha \right]}_{D}^{20}$ +504 (*c* 0.5, CHCl_3_). Anal. Calc. for C_14_H_17_FFeOS (308.19): C 54.56, H 5.56, S 10.40. Found: C 54.53, H 5.61, S 10.19. Reduction [82] of (*S*,*R*_P_)-*S*-*tert*-butyl-2-fluoroferrocenesulfoxide (***S*,*R*_P_-1i**; 0.92 g, 3.0 mmol), upon treatment by NaI (1.8 g, 12 mmol) and trifluoroacetic anhydride (2.5 g, 12 mmol) in acetone (16 mL) at 0 °C for 5 min, gave after purification by chromatography over silica gel (eluent: petroleum ether; Rf = 0.58) (*R*_P_)-1-(*tert*-butylthio)-2-fluoroferrocene (***R*_P_-8i**) in a quantitative yield (0.88 g) as an orange solid. Mp 68–70 °C. IR (ATR) *ν* 820, 886, 992, 1018, 1075, 1106, 1166, 1218, 1243, 1340, 1361, 1411, 1453, 1626, 1731, 2920, 2961, 3098, 3472 cm^−1^. ^1^H NMR (CDCl_3_) *δ* 1.23 (s, 9H, *t*Bu), 3.92 (q, 1H, *J* = 1.65 Hz, H4), 4.03 (br s, 1H, H5), 4.26 (s, 5H, Cp), 4.45 (br s, 1H, H3). ^13^C{^1^H} NMR (CDCl_3_) *δ* 30.8 (3CH_3_, C*Me*_3_), 46.3 (C, *C*Me_3_), 57.2 (d, CH, *J* = 15.6 Hz, C3), 61.4 (d, CH, *J* = 4.1 Hz, C4), 64.3 (d, C, *J* = 15.8 Hz, C1, *C*-S*t*Bu), 70.0 (CH, C5), 70.9 (5CH, Cp), 137.0 (d, C, *J* = 270.5 Hz, C2, C-F). ^19^F{^1^H} NMR (CDCl_3_) *δ* −187.2. Anal. Calc. for C_14_H_17_FFeS (292.19): C 57.55, H 5.86, S 10.97. Found: C 57.12, H 5.93, S 10.99.

### 3.10. General Procedure F: Attempted “Halogen Dance” Using LiTMP

To a stirred, cooled (−15 °C) solution of 2,2,6,6-tetramethylpiperidine (0.19 mL, 1.1 mmol) in THF (5 mL) was added a 1.4 M hexane solution of *n*BuLi (0.79 mL, 1.1 mmol). The mixture was stirred for 5 min at −15 °C and then for 2 min at −50 °C before the introduction of the iodoferrocene (1.0 mmol) in one portion. After 2 h at this temperature, methanol in excess (2 mL) was introduced at −50 °C before warming to rt and the addition of aqueous HCl (1 M, 10 mL). Extraction with EtOAc (3 × 20 mL), drying over MgSO_4_, and removal of the solvents under reduced pressure led to the crude product, which was purified by chromatography over silica gel (eluent given in the product description).

#### 3.10.1. From (*S*,*R*_P_)-*S*-*tert*-Butyl-2-iodo-5-(trimethylsilyl)ferrocenesulfoxide (***S*,*R*_P_-2ba**)

The general procedure F from 0.43 mmol of ***S*,*R*_P_-2ba** (0.21 g) afforded (*S*,*R*_P_)-*S*-*tert*-butyl-2-(trimethylsilyl)ferrocenesulfoxide (***S*,*R*_P_-1a**) (eluent: petroleum ether-EtOAc 60:40; Rf = 0.38) in 39% yield (60 mg) as an orange oil. The analyses were as described previously [46].

#### 3.10.2. From (*S*,*S*_P_)-2-Bromo-*S*-*tert*-butyl-5-(trimethylsilyl)ferrocenesulfoxide (***S*,*R*_P_-2ja**)

The general procedure F from 0.25 mmol of ***S*,*R*_P_-2ja** (0.11 g) afforded (eluent: petroleum ether-EtOAc 50:50; Rf = 0.42) (*S*,*R*_P_)-*S*-*tert*-butyl-2-(trimethylsilyl)ferrocenesulfoxide (***S*,*R*_P_-1a**) in 33% yield (30 mg) as an orange oil (see Section 3.10.1).

### 3.11. General Procedure G: Deprotolithiation of Enantiopure Ferrocenes using sBuLi Followed by Electrophilic Trapping 

This was adapted from a previously reported procedure [52,64]. To a solution of the ferrocene (1.0 mmol) in THF (3 mL) at −75 °C was added dropwise a 1.3 M cyclohexane solution of *s*BuLi (0.92 mL, 1.2 mmol), and the reaction was stirred at this temperature for 1 h before the addition of the electrophile (1.2 mmol unless otherwise specified; either pure for liquids or in solution for solids, as indicated below). The mixture was stirred at −75 °C for 15 min before being warmed to rt. The addition of 1 M HCl (5 mL), or saturated aqueous Na_2_S_2_O_3_ in the case of I_2_, extraction with EtOAc (3 × 20 mL), drying over MgSO_4_, and removal of the solvents under reduced pressure led to the crude product, which was purified by chromatography over silica gel (eluent given in the product description). When subsequent desilylation was performed, the protocol was as follows [64]. The silylated ferrocene (1.0 mmol) was treated by *n*Bu_4_NF (1.0 M THF solution; 1.6 mL, 2.0 mmol) in THF (5 mL) at rt for 0.5 h. The solvent was removed under reduced pressure, and the product was purified by chromatography over silica gel (eluent given in the product description).

#### 3.11.1. (*S*,*R*_P_)-*S*-*tert*-Butyl-2-fluoro-5-iodoferrocenesulfoxide (***S*,*R*_P_-2bi**)

The general procedure G from (*S*,*R*_P_)-*S*-*tert*-butyl-2-fluoroferrocenesulfoxide (***S*,*S*_P_-2ai**; 0.495 g, 1.6 mmol) and using I_2_ (0.49 g) in THF (5 mL) afforded (eluent: petroleum ether-EtOAc 80:20; Rf = 0.44) the title product in 8% yield (58 mg) as a yellow oil. IR (ATR) *ν* 825, 880, 992, 1058, 1107, 1129, 1174, 1250, 1339, 1363, 1384, 1412, 1498, 1704, 2960, 3084 cm^−1^. ^1^H NMR (CDCl_3_) *δ* 1.28 (s, 9H, *t*Bu), 4.37 (dd, 1H, *J* = 2.4 and 1.5 Hz, H4), 4.51 (s, 5H, Cp), 4.60 (t, 1H, *J* = 2.9 Hz, H3). ^13^C{^1^H} NMR (CDCl_3_) *δ* 23.9 (3CH_3_, C*Me*_3_), 27.1 (C, C5, C-I), 58.6 (C, *C*Me_3_), 59.7 (d, CH, *J* = 14.9 Hz, C3), 70.8 (d, CH, *J* = 3.2 Hz, C4), 71.8 (d, C, *J* = 11.6 Hz, C1, *C*-SO*t*Bu), 74.9 (5CH, Cp), 135.0 (d, C, *J* = 278.7 Hz, C2, C-F). ^19^F{^1^H} NMR (CDCl_3_) *δ* −184.2. ${\left[\alpha \right]}_{D}^{20}$ +446 (*c* 1.0, CHCl_3_). Anal. Calc. for C_14_H_16_FFeIOS (434.09): C 38.74, H 3.72, S 7.39. Found: C 38.56, H 4.15, S 7.20. The low yield was due to the isolation of an impure fraction in which the yield of ***S*,*R*_P_-2bi** can be estimated at 56%.

#### 3.11.2. (*S*,*S*_P_)-*S*-*tert*-Butyl-2-(diphenylphosphino)-5-fluoroferrocenesulfoxide (***S*,*S*_P_-2ei**)

The general procedure G from (*S*,*R*_P_)-*S*-*tert*-butyl-2-fluoroferrocenesulfoxide (***S*,*S*_P_-2ai**; 92 mg, 0.30 mmol) and using ClPPh_2_ (60 μL) afforded (eluent: petroleum ether-EtOAc 50:50; Rf = 0.67) the title product in 60% yield (88 mg) as a yellow oil. IR (ATR) *ν* 723, 743, 1026, 1061, 1119, 1175, 1216, 1247, 1284, 1364, 1391, 1414, 1452, 1548, 1709, 2975, 3056 cm^−1^. ^1^H NMR (CDCl_3_) *δ* 1.07 (s, 9H, *t*Bu), 3.95 (br s, 1H, H3), 4.74 (t, 1H, *J* = 3.0 Hz, H4), 7.24–7.29 (m, 5H, Ph), 7.33–7.37 (m, 3H, Ph), 7.55–7.58 (m, 2H, Ph). ^13^C{^1^H} NMR (CDCl_3_) *δ* 23.5 (3CH_3_, C*Me*_3_), 57.1 (C, *C*Me_3_), 60.4 (d, CH, *J* = 14.2 Hz, C4), 66.9 (t, CH, *J* = 4.1 Hz, C3), 71.0 (d, C, *J* = 27.8 Hz, C2, *C*-PPh_2_), 73.0 (5CH, Cp), 78.6 (dd, C, *J* = 22.4 and 9.8 Hz, C1, *C*-SO*t*Bu), 128.0 (CH, C4′), 128.1 (d, 2CH, *J* = 4.8 Hz, C3′ and/or C5′), 128.2 (d, 2CH, *J* = 6.5 Hz, C3′ and/or C5′), 129.4 (CH, C4′), 132.8 (d, 2CH, *J* = 19.4 Hz, C2′ and/or C6′), 135.7 (d, 2CH, *J* = 23.1 Hz, C2′ and/or C6′), 138.1 (dd, C, *J* = 276.6 and 5.2 Hz, C5, C-F), 138.1 (d, C, *J* = 14.4 Hz, C1′), 140.1 (d, C, *J* = 12.9 Hz, C1′). ^19^F{^1^H} NMR (CDCl_3_) *δ* −181.8. ^31^P{1H} NMR (CDCl_3_) *δ* −25.3. ${\left[\alpha \right]}_{D}^{20}$ +378 (*c* 1.0, CHCl_3_). Anal. Calc. for C_26_H_26_FFeOPS (492.37): C 63.43, H 5.32, S 6.51. Found: C 63.40, H 5.22, S 6.18.

#### 3.11.3. (*S*,*S*_P_)-*S*-*tert*-Butyl-2-fluoro-3-iodo-5-(trimethylsilyl)ferrocenesulfoxide (***S*,*S*_P_-9b**)

The general procedure G from (*S*,*S*_P_)-*S*-*tert*-butyl-2-fluoro-5-(trimethylsilyl)ferrocenesulfoxide (***S*,*S*_P_-2ai**; 0.38 g) and using I_2_ (0.31 g) in THF (4 mL) afforded (eluent: petroleum ether-EtOAc 80:20; Rf = 0.71) the title product in 94% yield (0.48 g) as an orange solid. Mp 170 °C. IR (ATR) *ν* 709, 749, 822, 929, 1028, 1115, 1168, 1245, 1362, 1428, 2956 cm^−1^. ^1^H NMR (CDCl_3_) *δ* 0.34 (s, 9H, SiMe_3_), 1.19 (s, 9H, *t*Bu), 4.26 (d, 1H, *J* = 0.9 Hz, H4), 4.41 (s, 5H, Cp). ^13^C{^1^H} NMR (CDCl_3_) *δ* 2.1 (3CH_3_, SiMe_3_), 23.6 (3CH_3_, C*Me*_3_), 31.2 (C, C3, C-I), 57.9 (C, *C*Me_3_), 66.5 (d, C, *J* = 3.0 Hz, C5, *C*-SiMe_3_), 74.7 (5CH, Cp), 75.2 (CH, C4), 79.3 (d, C, *J* = 9.8 Hz, C1, *C*-SO*t*Bu), 136.5 (d, C, *J* = 275.0 Hz, C2, C-F). ^19^F{^1^H} NMR (CDCl_3_) *δ* −178.2. ${\left[\alpha \right]}_{D}^{20}$ +28 (*c* 1.0, CHCl_3_). Crystal data for ***S*,*S*_P_-9b**. C_17_H_24_FFeIOSSi, *M* = 506.26; orthorhombic *P* 2_1_ 2_1_ 2_1_ (I.T.#19), *a* = 9.2687(9), *b* = 11.6233(9), *c* = 18.3737(18) Å, *V* = 1979.5(3) Å^3^. *Z* = 4, *d* = 1.699 g.·cm^−3^, *μ* = 2.497 mm^−1^. A final refinement on *F*^2^ with 4510 unique intensities and 206 parameters converged at ω*R(F^2^)* = 0.0673 (*R_F_* = 0.0286) for 4349 observed reflections with *I* > 2σ(*I*). CCDC 2152199.

#### 3.11.4. (*S*,*S*_P_)-*S*-*tert*-Butyl-2-fluoro-3-methyl-5-(trimethylsilyl)ferrocenesulfoxide (***S*,*S*_P_-9d**)

The general procedure G from (*S*,*S*_P_)-*S*-*tert*-butyl-2-fluoro-5-(trimethylsilyl)ferrocenesulfoxide (***S*,*S*_P_-2ai**; 1.1 g, 3.0 mmol) and using MeI (0.22 mL, 3.6 mmol) afforded (eluent: petroleum ether-EtOAc 80:20; Rf = 0.80) the title product in 96% yield (1.1 g) as an orange oil. IR (ATR) *ν* 755, 780, 817, 835, 962, 1003, 1051, 1116, 1174, 1243, 1289, 1364, 1378, 1408, 1456, 1473, 1497, 2899, 2957 cm^−1^. ^1^H NMR (CDCl_3_) *δ* 0.32 (s, 9H, SiMe_3_), 1.17 (s, 9H, *t*Bu), 2.06 (s, 3H, Me), 3.95 (d, 1H, *J* = 2.4 Hz, H4), 4.33 (s, 5H, Cp). ^13^C{^1^H} NMR (CDCl_3_) *δ* 2.2 (3CH_3_, SiMe_3_), 11.3 (d, CH_3_, *J* = 2.5 Hz, Me), 23.6 (3CH_3_, C*Me*_3_), 57.3 (C, *C*Me_3_), 62.2 (d, C, *J* = 2.6 Hz, C5, *C*-SiMe_3_), 70.95 (d, CH, *J* = 4.4 Hz, C4), 72.4 (5CH, Cp), 76.0 (d, C, *J* = 13.3 Hz, C3, *C*-Me), 79.3 (d, C, *J* = 9.1 Hz, C1, *C*-SO*t*Bu), 136.2 (d, C, *J* = 273.8 Hz, C2, C-F). ^19^F{^1^H} NMR (CDCl_3_) *δ* −184.3. ${\left[\alpha \right]}_{D}^{20}$ +320 (*c* 1.0, CHCl_3_). Anal. Calc. for C_18_H_27_FFeOSSi (394.40): C 54.82, H 6.90, S 8.13. Found: C 55.13, H 7.04, S 7.59. Desilylation of ***S*,*S*_P_-9d** (0.59 g, 1.5 mmol) gave (eluent: petroleum ether-EtOAc 80:20; Rf = 0.25) (*S*,*R*_P_)-*S*-*tert*-butyl-2-fluoro-3-methylferrocenesulfoxide (***S*,*R*_P_-10**) in a quantitative yield (0.48 g) as an orange oil, which was identified by NMR: ^1^H NMR (CDCl_3_) *δ* 1.18 (s, 9H, *t*Bu), 2.06 (s, 3H, Me), 4.05 (br s, 1H, H4), 4.22 (br s, 1H, H5), 4.38 (s, 5H, Cp).

#### 3.11.5. (*S*,*R*_P_)-*S*-*tert*-Butyl-2-fluoro-5-iodo-3-methylferrocenesulfoxide (***S*,*R*_P_-11b**)

The general procedure G from (*S*,*R*_P_)-*S*-*tert*-butyl-2-fluoro-3-methylferrocenesulfoxide (***S*,*R*_P_-10**; 0.33 g) and using I_2_ (0.31 g) in THF (4 mL) afforded (eluent: petroleum ether-EtOAc 80:20; Rf = 0.58) the title product in 45% yield (0.20 g) as an orange oil. IR (ATR) *ν* 749, 824, 933, 1002, 1051, 1107, 1127, 1175, 1216, 1293, 1342, 1363, 1412, 1470, 1487, 1712, 2923, 2961, 3080 cm^−1^. ^1^H NMR (CDCl_3_) *δ* 1.28 (s, 9H, *t*Bu), 2.00 (s, 3H, Me), 4.37 (d, 1H, *J* = 2.0 Hz, H4), 4.42 (s, 5H, Cp).

#### 3.11.6. (*S*,*R*_P_)-*S*-*tert*-Butyl-5-chloro-2-fluoro-3-methylferrocenesulfoxide (***S*,*R*_P_-11k**)

The general procedure G from (*S*,*R*_P_)-*S*-*tert*-butyl-2-fluoro-3-methylferrocenesulfoxide (***S*,*R*_P_-10**; 0.55 g, 1.7 mmol) and using C_2_Cl_6_ (0.44 g, 1.9 mmol) in THF (2 mL) afforded (eluent: petroleum ether-EtOAc 80:20; Rf = 0.44) the title product in 83% yield (0.50 g) as a yellow oil. IR (ATR) *ν* 749, 790, 825, 960, 1003, 1058, 1107, 1130, 1176, 1216, 1320, 1363, 1410, 1456, 1471, 1489, 1715, 2925, 2965, 3081 cm^−1^. ^1^H NMR (CDCl_3_) *δ* 1.27 (s, 9H, *t*Bu), 2.01 (s, 3H, Me), 4.30 (d, 1H, *J* = 1.5 Hz, H4), 4.48 (s, 5H, Cp). ^13^C{^1^H} NMR (CDCl_3_) *δ* 10.7 (d, CH_3_, *J* = 2.7 Hz, Me), 23.6 (3CH_3_, C*Me*_3_), 58.3 (C, *C*Me_3_), 65.3 (d, CH, *J* = 2.2 Hz, C4), 70.5 (d, C, *J* = 12.5 Hz, C1, *C*-SO*t*Bu), 70.8 (d, C, *J* = 13.3 Hz, C3, *C*-Me), 74.7 (5CH, Cp), 84.9 (C, C5, C-Cl), 133.5 (d, C, *J* = 277.7 Hz, C2, C-F). ^19^F{^1^H} NMR (CDCl_3_) *δ* −190.8. ${\left[\alpha \right]}_{D}^{20}$ +84 (*c* 0.5, CHCl_3_). Anal. Calc. for C_15_H_18_ClFFeOS (356.66): C 50.51, H 5.09, S 8.99. Found: C 50.54, H 5.13, S 90.2. ***S*,*R*_P_-11k** was converted to (*S*,*R*_P_)-*S*-*tert*-butyl-2-fluoro-5-iodo-3-methylferrocenesulfoxide (***S*,*R*_P_-11b**) by chlorine/lithium exchange as follows. To a solution of ***S*,*R*_P_-11k** (0.49 g, 1.4 mmol) in THF (5 mL) at −75 °C was added dropwise a 1.3 M cyclohexane solution of *s*BuLi (1.3 mL, 1.65 mmol), and the reaction was stirred at this temperature for 1 h before the addition of I_2_ (0.38 g, 1.5 mmol) in THF (3 mL). The mixture was stirred at −75 °C for 15 min before being warmed to rt. The addition of saturated aqueous Na_2_S_2_O_3_ (10 mL), extraction with EtOAc (3 × 20 mL), drying over MgSO_4_, and removal of the solvents under reduced pressure led to the crude product, which was purified by chromatography over silica gel (eluent: petroleum ether-EtOAc 80:20; Rf = 0.58) to afford ***S*,*R*_P_-11b** in 51% yield (0.31 g), the rest being recovered ***S*,*R*_P_-11k**. By using the same protocol with C_2_Cl_6_ as the electrophile, ***S*,*R*_P_-11b** was also converted to ***S*,*R*_P_-11k**, which was isolated in 45% yield.

### 3.12. Computational Details

The quantum chemical calculations were performed using GAUSSIAN 09 package [101]. All computations were conducted within the DFT framework. The CAM-B3LYP [102] hybrid functional was employed. The optimized geometries were obtained using the LANL2DZ basis set for both Fe and I and the 6-31G(d) basis set for the other atoms. No symmetry constraints were applied. To check stationary points and calculate zero-point vibrational energies (ZPVE) and thermal corrections, the Hessian matrices were calculated at the same level of theory. The single point energies of species were obtained at the CAM-B3LYP/LANL2DZ + 6-311 + G(d,p) level.

For the calculation of the CH acidities, we employed the approach successfully used before in the ferrocene series, including carboxamides [47], halides [48], sulfonates [71], and sulfonamides [72], and described therein. Briefly, the gas-phase acidity Δ*G*_acid_ was calculated as the Gibbs energy of deprotonation of the corresponding substrate R-H (R-H(g) → R^−^(g) + H^+^(g)):

Δ*G*_acid_ = *G*^0^_298_(R^−^) + *G*^0^_298_(H^+^) − *G*^0^_298_(R-H) (1)

The solvent influence was treated by using the polarized continuum model (IEF PCM) with the default parameters for THF [103].

The following isodesmic reaction was considered for the p*K*_a_ values calculation:

R-H(s) + Het^−^(s) → R^−^(s) + Het-H(s) (2)

where Het–H is furan. The latter was chosen as the reference compound due to its structural similarity and since its p*K*_a_(THF) = 35.6 reported by Fraser et al. [104] was expected to be close to the substrates under consideration.

Regarding the diversity of bases used, we chose LiNMe_2_ as a model compound to track the influence of lithium coordination on the p*K*_a_ values.

The calculated values of the Gibbs energies Δ*G*_acid_ [kcal·mol^−1^] for deprotonation are given in the Supplementary Materials.

## 4. Conclusions

The purpose of this article was to show that the *tert*-butylsulfinyl group, already known to direct the deprotometalation on ferrocene to a privileged neighboring site, can be used more generally to access more substituted derivatives. 

To this end, by starting from classical 2-substituted *S*-*tert*-butylferrocenesulfoxides, deprometalation conditions were found to introduce other substituents on the less activated ferrocene position next to the sulfoxide. Subsequent removal of the trimethylsilyl group led to 2-substituted *S*-*tert*-butylferrocenesulfoxides otherwise inaccessible. Their functionalization turned out to be easy, leading to many new stereopure di- to tetrasubstituted ferrocenesulfoxides. 

Because the sulfoxide function can be reduced, these methodologies open the way to new planar chiral polysubstituted ferrocenes.

## Figures and Tables

**Table 1:**
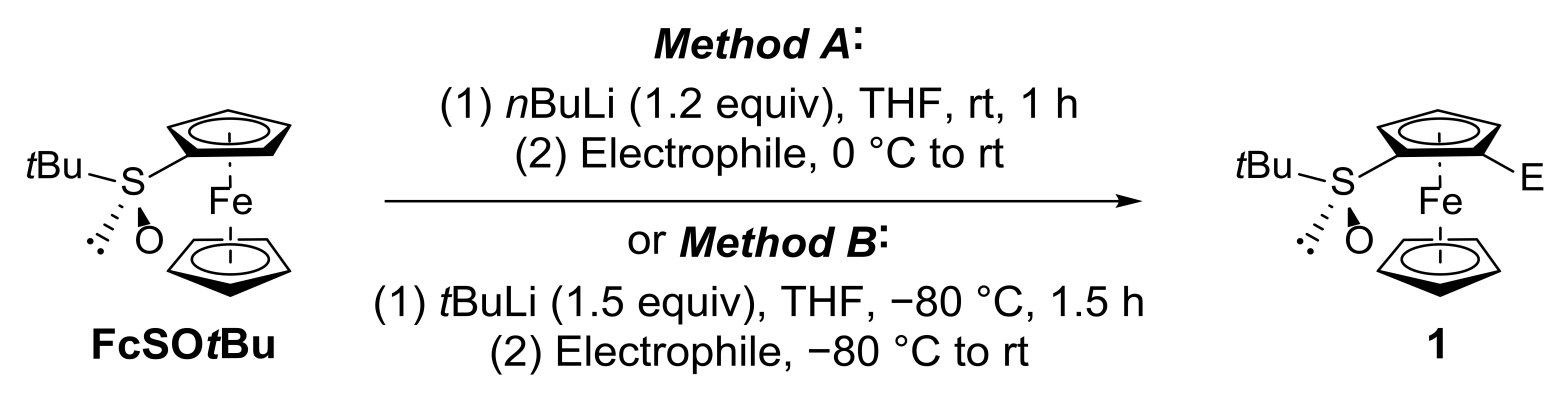
Deprotometalation of *S*-*tert*-butylferrocenesulfoxides followed by electrophilic trapping.

Entry	Substrate	Method	Electrophile ^1^	Product 1, Yield (%) ^2^	
1	***S*-FcSO*t*Bu**	**A**	ClSiMe_3_	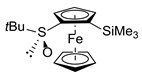	***S*,*S*_P_-1a**, 89
2	***rac*-FcSO*t*Bu**				***R*,*R*_P_/*S*,*S*_P_-1a**, 88
3	***S*-FcSO*t*Bu**	**A**	I_2_	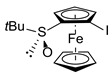	***S*,*R*_P_-1b**, 85
4	***rac*-FcSO*t*Bu**				***R*,*S*_P_/*S*,*R*_P_-1b**, 84
5	***S*-FcSO*t*Bu**	**A**	D_2_O	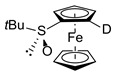	***S*,*S*_P_-1c**, 98 (95% D)
6	***S*-FcSO*t*Bu**	**A**	IMe	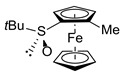	***S*,*S*_P_-1d**, 62
7	***S*-FcSO*t*Bu**	**B**	ClPPh_2_	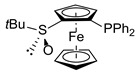	***S*,*S*_P_-1e**, 92
8	***S*-FcSO*t*Bu**	**B**	ClSnBu_3_	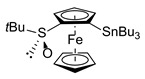	***S*,*R*_P_-1f**, 95

^1^ See the Materials and Methods section for more details on the electrophilic trapping and subsequent hydrolysis. ^2^ Yields are given after purification, as described in Materials and Methods.

**Table 2:**
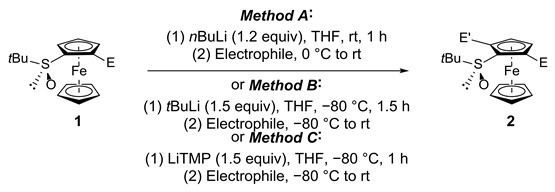
Deprotometalation of 2-substituted *S*-*tert*-butylferrocenesulfoxides followed by electrophilic trapping.

Entry	Substrate 1 (E)	Method	Electrophile ^1^	Product 2, Yield (%) ^2^	
1	***S*,*S*_P_-1a** (SiMe_3_)	**A**	ClSiMe_3_	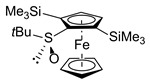	***S*-2aa**, 80
2	***R*,*R*_P_/*S*,*S*_P_-1a** (SiMe_3_)				***rac*-2aa**, 63
3	***S*,*S*_P_-1a** (SiMe_3_)	**A**	I_2_	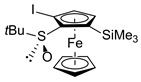	***S*,*S*_P_-2ab**, 78
4	***R*,*R*_P_-1a** (SiMe_3_)			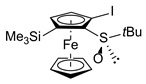	***R*,*R*_P_-2ab**, 77
5	***S*,*S*_P_-1a** (SiMe_3_)	**A**	D_2_O	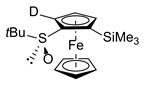	***S*,*S*_P_-2ac**, 98 (90% D)
6	***S*,*S*_P_-1a** (SiMe_3_)	**B**	Ph_2_CO	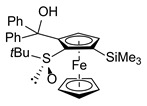	***S*,*S*_P_-2ag**, 35 ^3^
7	***S*,*S*_P_-1e** (PPh_2_)	**A**	I_2_	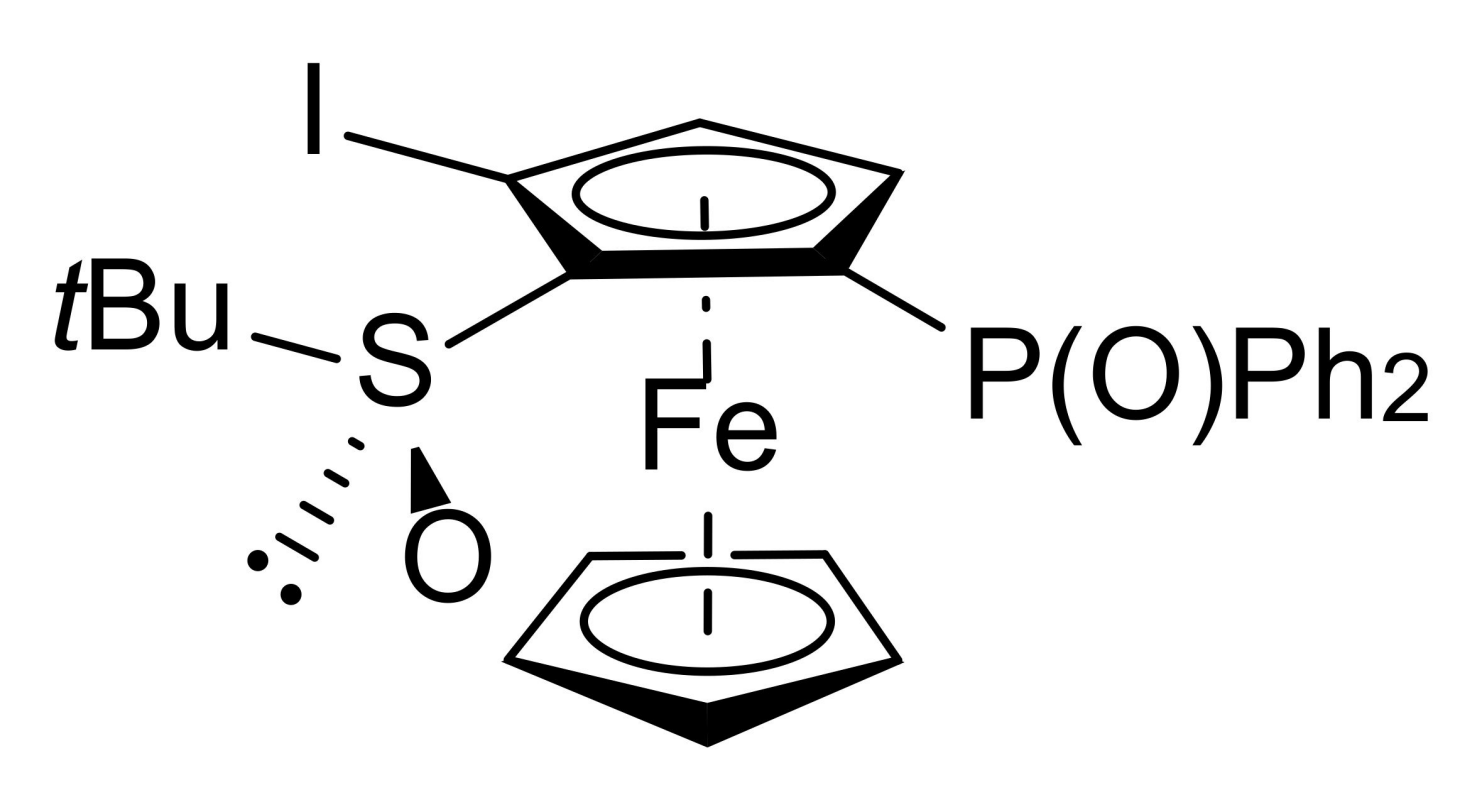	***S*,*S*_P_-2eb**, 40 ^4^
8	***S*,*S*_P_-1e** (PPh_2_)	**C**	ClSiMe_3_	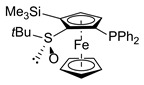	***S*,*S*_P_-2ea**, 70

^1^ See the Materials and Methods section for more details on the electrophilic trapping and subsequent hydrolysis. ^2^ Yields are given after purification, as described in Materials and Methods. ^3^ ***S*,*S*_P_-1a** was recovered in 40% yield. ^4^ Presumed degradation as no ***S*,*S*_P_-1e** was recovered in this case.

**Table 3:**
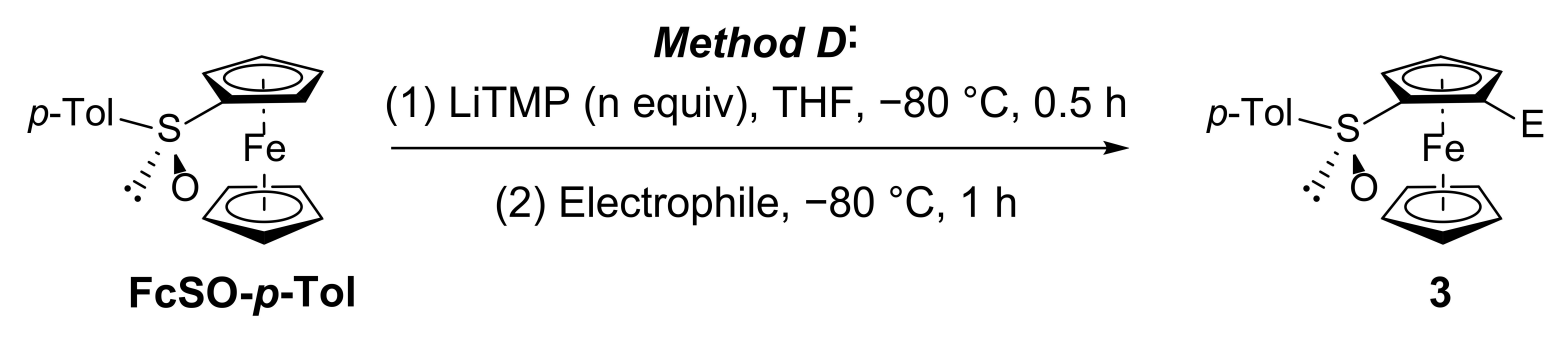
Deprotometalation of racemic *S*-(4-tolyl)ferrocenesulfoxide (***rac*-FcSO-*p*-Tol**) or (*S*)-*S*-(4-tolyl)ferrocenesulfoxide (***S*-FcSO-*p*-Tol**) followed by electrophilic trapping.

Entry	Substrate	n Equiv	Electrophile ^1^	Product 3, Yield (%) ^2^	
1	***rac*-FcSO-*p*-Tol**	1.2	**ClSiMe_3_**	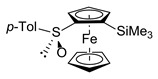	***R*,*R*_P_/*S*,*S*_P_-3a**, 61
2	***rac*-FcSO-*p*-Tol**	1.5	**ClSiMe_3_**	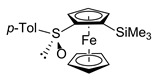	***R*,*R*_P_/*S*,*S*_P_-3a**, 43
				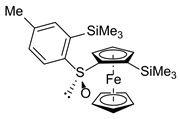	***R*,*R*_P_/*S*,*S*_P_-3a’**, 19
3	***rac*-FcSO-*p*-Tol**	1.1	**I_2_**	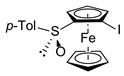	***R*,*S*_P_/*S*,*R*_P_-3b**, 51 ^3^
4		1.5			***R*,*S*_P_/*S*,*R*_P_-3b**, 81
5	***S*-FcSO-*p*-Tol**	2	**DCl/D_2_O**	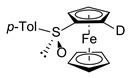	***S*,*S*_P_-3c**, quant. (80% D)

^1^ See the Materials and Methods section for more details on the electrophilic trapping and subsequent hydrolysis. ^2^ Yields are given after purification, as described in Materials and Methods. ^3^ ***rac*-FcSO-*p*-Tol** was recovered in 20% yield.

**Table 4:**
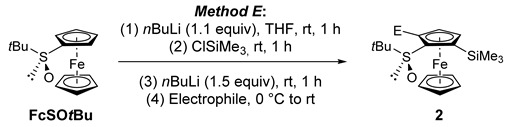
One-pot deprotolithiation-trimethylsilylation-deprotolithiation-electrophilic trapping sequence from *S*-*tert*-butylferrocenesulfoxides.

Entry	Substrate	Electrophile ^1^	Product 2, Yield (%) ^2^	
1	***S*-FcSO*t*Bu**	I_2_	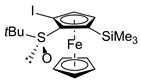	***S*,*S*_P_-2ab**, 83 ^3^
2	***R*-FcSO*t*Bu**		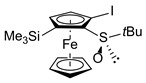	***R*,*R*_P_-2ab**, 53
3	***S*-FcSO*t*Bu**	IMe	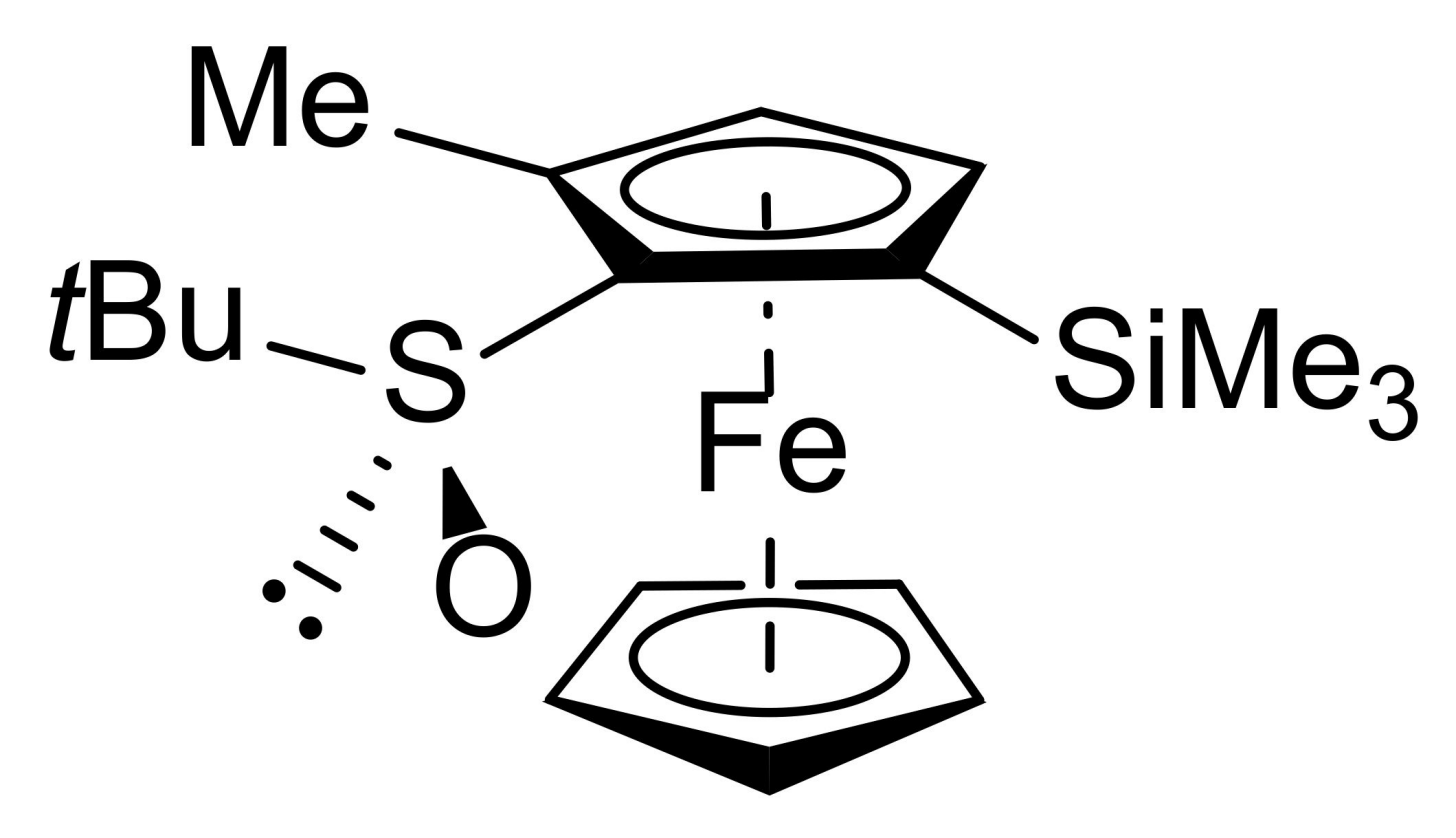	***S*,*S*_P_-2ad**, 74
4	***S*-FcSO*t*Bu**	ClPPh_2_	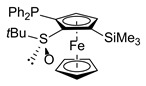	***S*,*R*_P_-2ae**, 48
5	***S*-FcSO*t*Bu**	Ph_2_CO	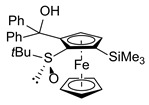	***S*,*S*_P_-2ag**, 51
6	***S*-FcSO*t*Bu**	CH_2_=NMe_2_I	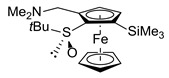	***S*,*S*_P_-2ah**, 66 ^4^
7	***S*-FcSO*t*Bu**	NFSI	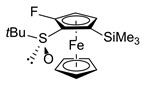	***S*,*S*_P_-2ai**, 67

^1^ See the Materials and Methods section for more details on the electrophilic trapping and subsequent hydrolysis. ^2^ Yields are given after purification, as described in the Materials and Methods section. ^3^ Yield after subsequent desilylation. ^4^ (*S*,*S*_P_)-*S*-*tert*-Butyl-2-formyl-5-(trimethylsilyl)ferrocenesulfoxide (***S*,*S*_P_-2ah’**) was also isolated in 23% yield; this compound might be formed from ***S*,*S*_P_-2ah** during the work-up in the air.

**Table 5:**
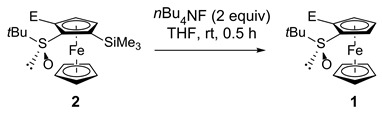
Desilylation of 2,5-disubstituted *S*-*tert*-butylferrocenesulfoxides.

Entry	Substrate 2 (E)	Product 1, Yield (%) ^1^	
1	***R*,*R*_P_-2ab** (I)	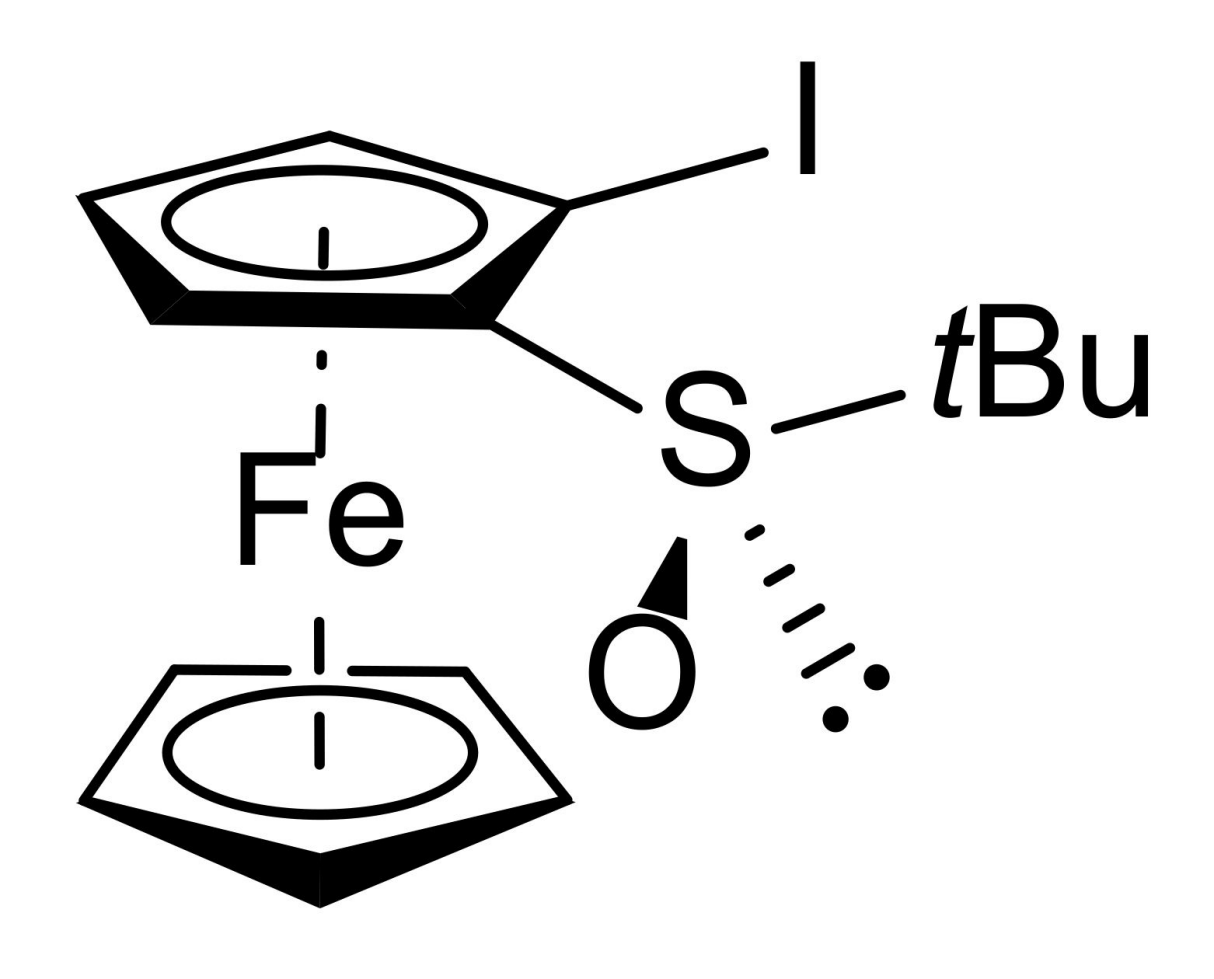	***R*,*R*_P_-1b**, 83
2	***S*,*S*_P_-2ac** (D)	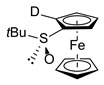	***S*,*R*_P_-1c**, 60
3	***S*,*S*_P_-2ad** (Me)	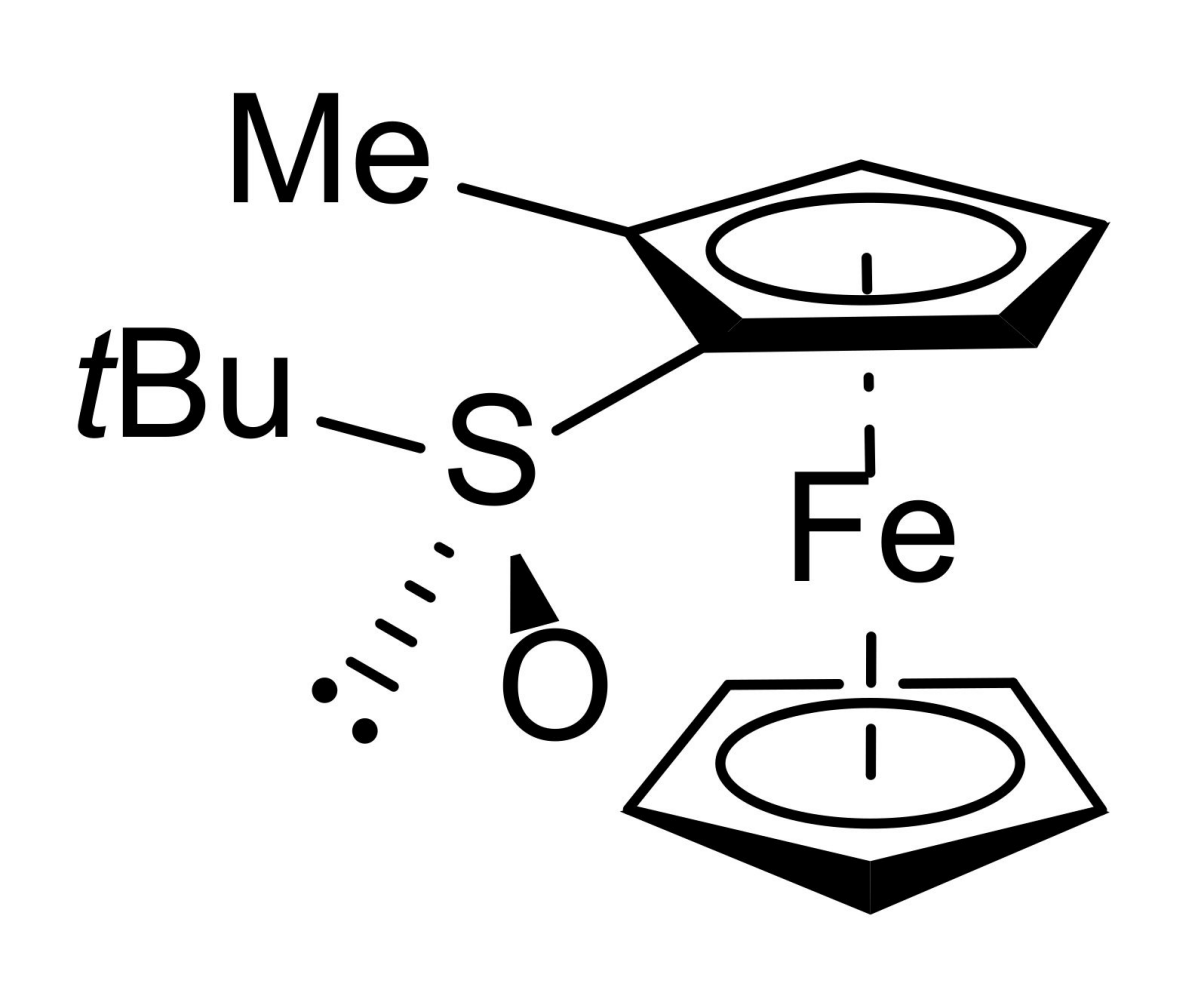	***S*,*R*_P_-1d**, quant.
4	***S*,*S*_P_-2ah** (CH_2_NMe_2_)	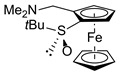	***S*,*R*_P_-1h**, quant.
5	***S*,*S*_P_-2ai** (F)	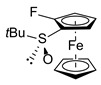	***S*,*R*_P_-1i**, quant.
6 ^2^	***S*-2aa** (SiMe_3_)	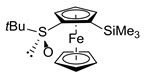	***S*,*S*_P_-1a**, 78
7 ^2^	***rac*-2aa** (SiMe_3_)		***S*,*S*_P_/*R*,*R*_P_-1a**, 67

^1^ Yields are given after purification, as described in the Materials and Methods section. ^2^ 1 equiv of *n*Bu_4_NF was used in this case.

## Data Availability

All data are included in this paper.

## Supplementary Materials

The following supporting information can be downloaded at: https://www.mdpi.com/article/10.3390/molecules27061798/s1, The NMR data of all compounds described; selected NOESY correlations; the calculated values of the Gibbs energies Δ*G*_acid_ [kcal·mol^−1^] for deprotonation.

## Sample Availability

Samples of the compounds are available from the authors.
