# Supplementary material for: Synthesis of Polysubstituted Ferrocenesulfoxides
Source: Molecules. 2022 Mar 9;27(6):1798. doi: 10.3390/molecules27061798 (PMC8955965; doi:10.3390/molecules27061798)

checkCIF/PLATON report

Structure factors have been supplied for datablock(s) MW239, MW253, MW259, MW4852

THIS REPORT IS FOR GUIDANCE ONLY. IF USED AS PART OF A REVIEW PROCEDURE FOR PUBLICATION, IT SHOULD NOT REPLACE THE EXPERTISE OF AN EXPERIENCED CRYSTALLOGRAPHIC REFEREE.

No syntax errors found. CIF dictionary Interpreting this report

Datablock: MW4852

Bond precision:	C-C = 0.0030 A		Wavelength=0.71073
Cell:	a=10.8267(7)	b=10.0035(5)	c=15.0821(7)
	alpha=90	beta=99.897(2)	gamma=90
Temperature:	150 K		

	Calculated	Reported
Volume	1609.16(15)	1609.16(15)
Space group	P 21/c	P 21/c
Hall group	-P 2ybc	-P 2ybc
Moiety formula	C20 H18 Fe2 O S	C20 H18 Fe2 O S
Sum formula	C20 H18 Fe2 O S	C20 H18 Fe2 O S
Mr	418.10	418.10
Dx,g cm-3	1.726	1.726
Z	4	4
Mu (mm-1)	1.935	1.935
F000	856.0	856.0
F000'	859.46	
h,k,lmax	14,13,19	14,12,19
Nref	3691	3652
Tmin,Tmax	0.332,0.793	0.568,0.793
Tmin'	0.307	

Correction method= # Reported T Limits: Tmin=0.568 Tmax=0.793
AbsCorr = MULTI-SCAN

Data completeness= 0.989 Theta(max)= 27.518

R(reflections)= 0.0302(3057) wR2(reflections)= 0.0707(3652)

S = 1.053 Npar= 217

The following ALERTS were generated. Each ALERT has the format
test-name_ALERT_alert-type_alert-level.
Click on the hyperlinks for more details of the test.

Alert level C

```
PLAT911_ALERT_3_C Missing FCF Refl Between Thmin & STh/L=    0.600          6 Report
```

Alert level G

```
PLAT794_ALERT_5_G Tentative Bond Valency for Fe1       (II)      .       2.14 Info
PLAT794_ALERT_5_G Tentative Bond Valency for Fe2       (II)      .       2.12 Info
PLAT802_ALERT_4_G CIF Input Record(s) with more than 80 Characters          3 Info
PLAT910_ALERT_3_G Missing # of FCF Reflection(s) Below Theta(Min).          1 Note
PLAT912_ALERT_4_G Missing # of FCF Reflections Above STh/L=  0.600         32 Note
PLAT933_ALERT_2_G Number of HKL-OMIT Records in Embedded .res File          1 Note
PLAT941_ALERT_3_G Average HKL Measurement Multiplicity ..........         1.0 Low
PLAT978_ALERT_2_G Number C-C Bonds with Positive Residual Density.         12 Info
```

0 **ALERT level A** = Most likely a serious problem - resolve or explain
0 **ALERT level B** = A potentially serious problem, consider carefully
1 **ALERT level C** = Check. Ensure it is not caused by an omission or oversight
8 **ALERT level G** = General information/check it is not something unexpected

0 ALERT type 1 CIF construction/syntax error, inconsistent or missing data
2 ALERT type 2 Indicator that the structure model may be wrong or deficient
3 ALERT type 3 Indicator that the structure quality may be low
2 ALERT type 4 Improvement, methodology, query or suggestion
2 ALERT type 5 Informative message, check

Datablock: MW253

Bond precision: C-C = 0.0078 A Wavelength=0.71073

Cell:	a=7.5042(5)	b=13.5729(5)	c=14.4725(6)
	alpha=90	beta=90	gamma=90
Temperature:	150 K		

	Calculated	Reported
Volume	1474.08(13)	1474.08(13)
Space group	P 21 21 21	P 21 21 21
Hall group	P 2ac 2ab	P 2ac 2ab
Moiety formula	C14 H17 Fe I O S	C14 H17 Fe I O S
Sum formula	C14 H17 Fe I O S	C14 H17 Fe I O S
Mr	416.09	416.08
Dx,g cm-3	1.875	1.875
Z	4	4
Mu (mm-1)	3.244	3.244
F000	816.0	816.0
F000'	816.20	
h,k,lmax	9,17,18	9,17,18
Nref	3384[1949]	3247
Tmin,Tmax	0.213,0.321	0.229,0.321
Tmin'	0.140	

Correction method= # Reported T Limits: Tmin=0.229 Tmax=0.321
AbsCorr = MULTI-SCAN

Data completeness= 1.67/0.96 Theta(max)= 27.486

R(reflections)= 0.0359(3063) wR2(reflections)= 0.0920(3247)

S = 1.101 Npar= 125

The following ALERTS were generated. Each ALERT has the format
test-name_ALERT_alert-type_alert-level.
Click on the hyperlinks for more details of the test.

Alert level C

```
PLAT910_ALERT_3_C Missing # of FCF Reflection(s) Below Theta(Min).          5 Note
PLAT911_ALERT_3_C Missing FCF Refl Between Thmin & STh/L=    0.600         40 Report
PLAT975_ALERT_2_C Check Calcd Resid. Dens.  0.86Ang From O11     .       0.41 eA-3
```

Alert level G

```
PLAT019_ALERT_1_G _diffrn_measured_fraction_theta_full/*_max < 1.0      0.997 Report
PLAT083_ALERT_2_G SHELXL Second Parameter in WGHT  Unusually Large       5.88 Why ?
PLAT171_ALERT_4_G The CIF-Embedded .res File Contains EADP Records          1 Report
PLAT232_ALERT_2_G Hirshfeld Test Diff (M-X)  Fe1      --C3       .        5.3 s.u.
PLAT794_ALERT_5_G Tentative Bond Valency for Fe1       (II)      .       2.12 Info
PLAT912_ALERT_4_G Missing # of FCF Reflections Above STh/L=  0.600          5 Note
PLAT941_ALERT_3_G Average HKL Measurement Multiplicity ..........         2.4 Low
PLAT978_ALERT_2_G Number C-C Bonds with Positive Residual Density.          2 Info
```

---

0 **ALERT level A** = Most likely a serious problem - resolve or explain

0 **ALERT level B** = A potentially serious problem, consider carefully
3 **ALERT level C** = Check. Ensure it is not caused by an omission or oversight
8 **ALERT level G** = General information/check it is not something unexpected

1 ALERT type 1 CIF construction/syntax error, inconsistent or missing data
4 ALERT type 2 Indicator that the structure model may be wrong or deficient
3 ALERT type 3 Indicator that the structure quality may be low
2 ALERT type 4 Improvement, methodology, query or suggestion
1 ALERT type 5 Informative message, check

Datablock: MW259

Bond precision: C-C = 0.0198 A Wavelength=0.71073

Cell:	a=9.243(4)	b=11.592(6)	c=18.243(8)
	alpha=90	beta=90	gamma=90
Temperature:	150 K		

	Calculated	Reported
Volume	1954.6(16)	1954.7(16)
Space group	P 21 21 21	P 21 21 21
Hall group	P 2ac 2ab	P 2ac 2ab
Moiety formula	C17 H25 Fe I O S Si	C17 H25 Fe I O S Si
Sum formula	C17 H25 Fe I O S Si	C17 H25 Fe I O S Si
Mr	488.27	488.27
Dx,g cm-3	1.659	1.659
Z	4	4
Mu (mm-1)	2.518	2.518
F000	976.0	976.0
F000’	976.54	
h,k,lmax	11,14,23	11,14,23
Nref	4275[2436]	4064
Tmin,Tmax	0.586,0.818	0.508,0.818
Tmin’	0.454	

Correction method= # Reported T Limits: Tmin=0.508 Tmax=0.818
AbsCorr = MULTI-SCAN

Data completeness= 1.67/0.95 Theta(max)= 27.038

R(reflections)= 0.0605(2808) wR2(reflections)= 0.1491(4064)

S = 0.980 Npar= 205

The following ALERTS were generated. Each ALERT has the format
test-name_ALERT_alert-type_alert-level.
Click on the hyperlinks for more details of the test.

Alert level C

```
PLAT213_ALERT_2_C Atom C11              has ADP max/min Ratio .....        3.8 oblate
PLAT234_ALERT_4_C Large Hirshfeld Difference C9       --C10      .        0.18 Ang.
PLAT250_ALERT_2_C Large U3/U1 Ratio for Average U(i,j) Tensor ....         2.2 Note
PLAT342_ALERT_3_C Low Bond Precision on  C-C Bonds ...............     0.01985 Ang.
PLAT911_ALERT_3_C Missing FCF Refl Between Thmin & STh/L=    0.600          18 Report
PLAT971_ALERT_2_C Check Calcd Resid. Dens.  1.00Ang From I1               1.95 eA-3
PLAT971_ALERT_2_C Check Calcd Resid. Dens.  1.05Ang From I1               1.90 eA-3
```

Alert level G

```
PLAT431_ALERT_2_G Short Inter HL..A Contact  I1       ..O14      .        2.96 Ang.
                                           3/2-x,1-y,-1/2+z  =      2_664 Check
PLAT794_ALERT_5_G Tentative Bond Valency for Fe1       (II)      .        2.13 Info
PLAT910_ALERT_3_G Missing # of FCF Reflection(s) Below Theta(Min).           2 Note
PLAT912_ALERT_4_G Missing # of FCF Reflections Above STh/L=  0.600          58 Note
PLAT941_ALERT_3_G Average HKL Measurement Multiplicity ..........          3.4 Low
PLAT978_ALERT_2_G Number C-C Bonds with Positive Residual Density.           0 Info
```

0 **ALERT level A** = Most likely a serious problem - resolve or explain
0 **ALERT level B** = A potentially serious problem, consider carefully
7 **ALERT level C** = Check. Ensure it is not caused by an omission or oversight
6 **ALERT level G** = General information/check it is not something unexpected

0 ALERT type 1 CIF construction/syntax error, inconsistent or missing data
6 ALERT type 2 Indicator that the structure model may be wrong or deficient
4 ALERT type 3 Indicator that the structure quality may be low
2 ALERT type 4 Improvement, methodology, query or suggestion
1 ALERT type 5 Informative message, check

Datablock: MW239

Bond precision:	C-C = 0.0068 A		Wavelength=0.71073
Cell:	a=9.2687(9)	b=11.6233(9)	c=18.3737(18)
	alpha=90	beta=90	gamma=90
Temperature:	150 K		

	Calculated	Reported
Volume	1979.5(3)	1979.5(3)
Space group	P 21 21 21	P 21 21 21
Hall group	P 2ac 2ab	P 2ac 2ab
Moiety formula	C17 H24 F Fe I O S Si	C17 H24 F Fe I O S Si
Sum formula	C17 H24 F Fe I O S Si	C17 H24 F Fe I O S Si
Mr	506.26	506.26
Dx,g cm-3	1.699	1.699
Z	4	4
Mu (mm-1)	2.497	2.497
F000	1008.0	1008.0
F000'	1008.61	
h,k,lmax	12,15,23	12,15,23
Nref	4518[2570]	4510
Tmin,Tmax	0.247,0.342	0.170,0.342
Tmin'	0.226	

Correction method= # Reported T Limits: Tmin=0.170 Tmax=0.342
AbsCorr = MULTI-SCAN

Data completeness= 1.75/1.00 Theta(max)= 27.442

R(reflections)= 0.0286(4349) wR2(reflections)= 0.0691(4510)

S = 1.128 Npar= 206

The following ALERTS were generated. Each ALERT has the format
test-name_ALERT_alert-type_alert-level.
Click on the hyperlinks for more details of the test.

Alert level G

```
PLAT431_ALERT_2_G Short Inter HL..A Contact  I1      ..O15       .      2.93 Ang.
                                        1/2-x,1-y,1/2+z  =      2_565 Check
PLAT794_ALERT_5_G Tentative Bond Valency for Fe1       (II)      .      2.10 Info
PLAT910_ALERT_3_G Missing # of FCF Reflection(s) Below Theta(Min).         1 Note
PLAT912_ALERT_4_G Missing # of FCF Reflections Above STh/L=  0.600         1 Note
PLAT941_ALERT_3_G Average HKL Measurement Multiplicity ...........       4.6 Low
PLAT978_ALERT_2_G Number C-C Bonds with Positive Residual Density.         2 Info
```

---

0 **ALERT level A** = Most likely a serious problem - resolve or explain
0 **ALERT level B** = A potentially serious problem, consider carefully
0 **ALERT level C** = Check. Ensure it is not caused by an omission or oversight
6 **ALERT level G** = General information/check it is not something unexpected

0 ALERT type 1 CIF construction/syntax error, inconsistent or missing data

```
2 ALERT type 2 Indicator that the structure model may be wrong or deficient
2 ALERT type 3 Indicator that the structure quality may be low
1 ALERT type 4 Improvement, methodology, query or suggestion
1 ALERT type 5 Informative message, check
```

It is advisable to attempt to resolve as many as possible of the alerts in all categories. Often the minor alerts point to easily fixed oversights, errors and omissions in your CIF or refinement strategy, so attention to these fine details can be worthwhile. In order to resolve some of the more serious problems it may be necessary to carry out additional measurements or structure refinements. However, the purpose of your study may justify the reported deviations and the more serious of these should normally be commented upon in the discussion or experimental section of a paper or in the "special_details" fields of the CIF. checkCIF was carefully designed to identify outliers and unusual parameters, but every test has its limitations and alerts that are not important in a particular case may appear. Conversely, the absence of alerts does not guarantee there are no aspects of the results needing attention. It is up to the individual to critically assess their own results and, if necessary, seek expert advice.

Publication of your CIF in IUCr journals

A basic structural check has been run on your CIF. These basic checks will be run on all CIFs submitted for publication in IUCr journals (*Acta Crystallographica*, *Journal of Applied Crystallography*, *Journal of Synchrotron Radiation*); however, if you intend to submit to *Acta Crystallographica Section C* or *E* or *IUCrData*, you should make sure that full publication checks are run on the final version of your CIF prior to submission.

Publication of your CIF in other journals

Please refer to the *Notes for Authors* of the relevant journal for any special instructions relating to CIF submission.

PLATON version of 20/01/2022; check.def file version of 19/01/2022

Datablock MW4852 - ellipsoid plot

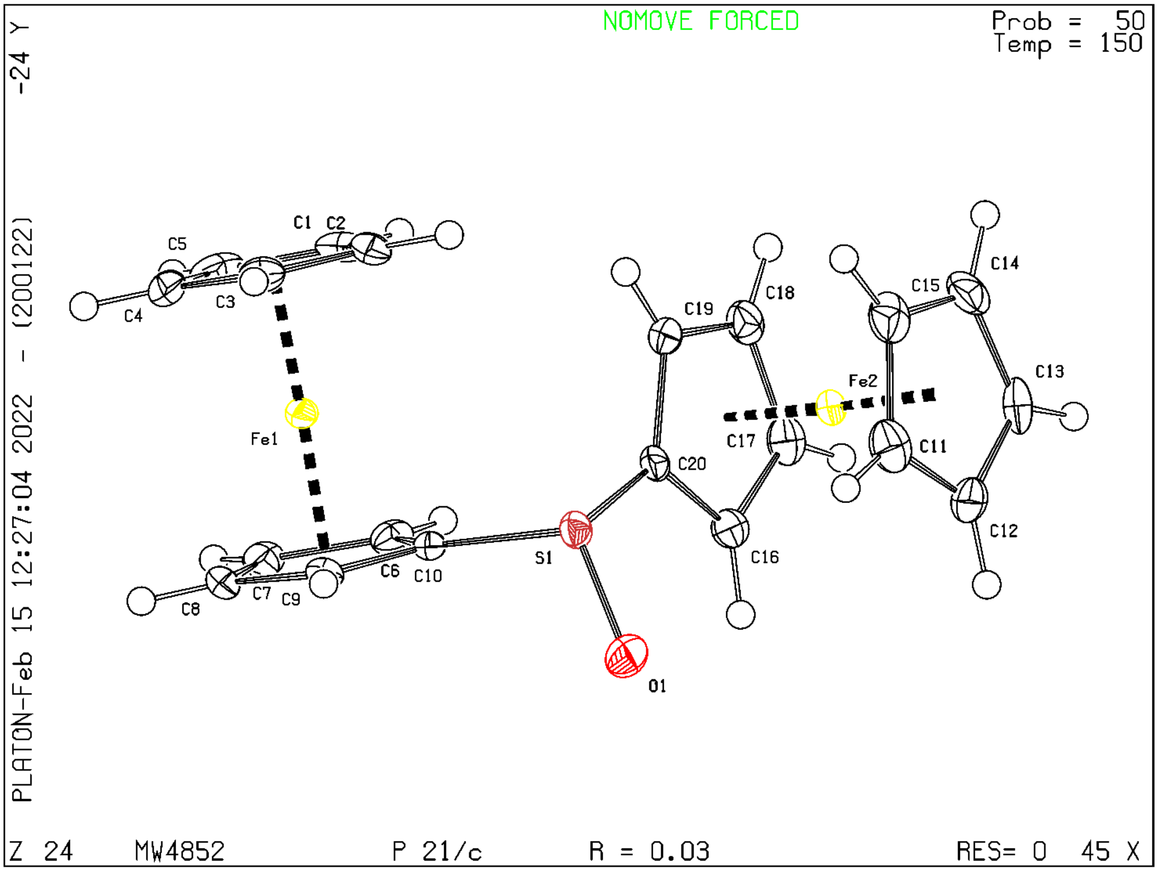

Datablock MW253 - ellipsoid plot

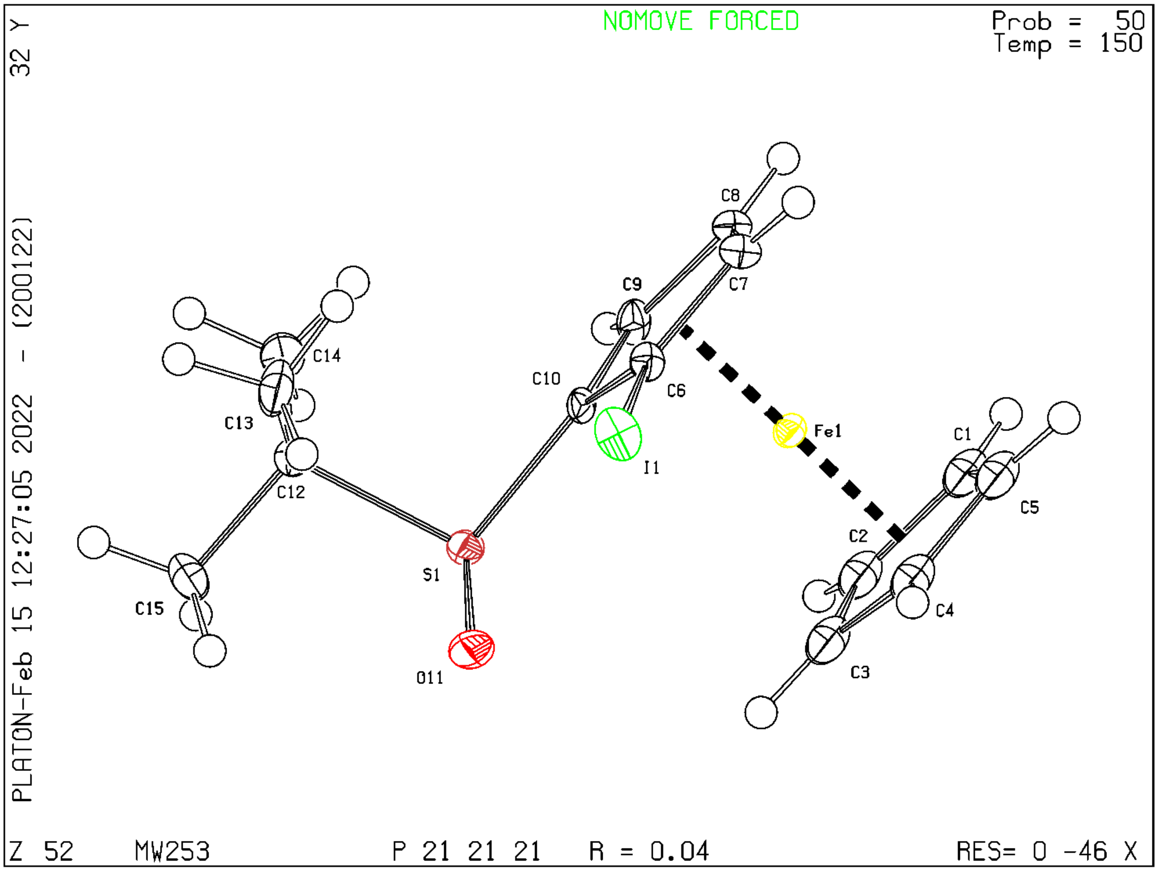

Datablock MW259 - ellipsoid plot

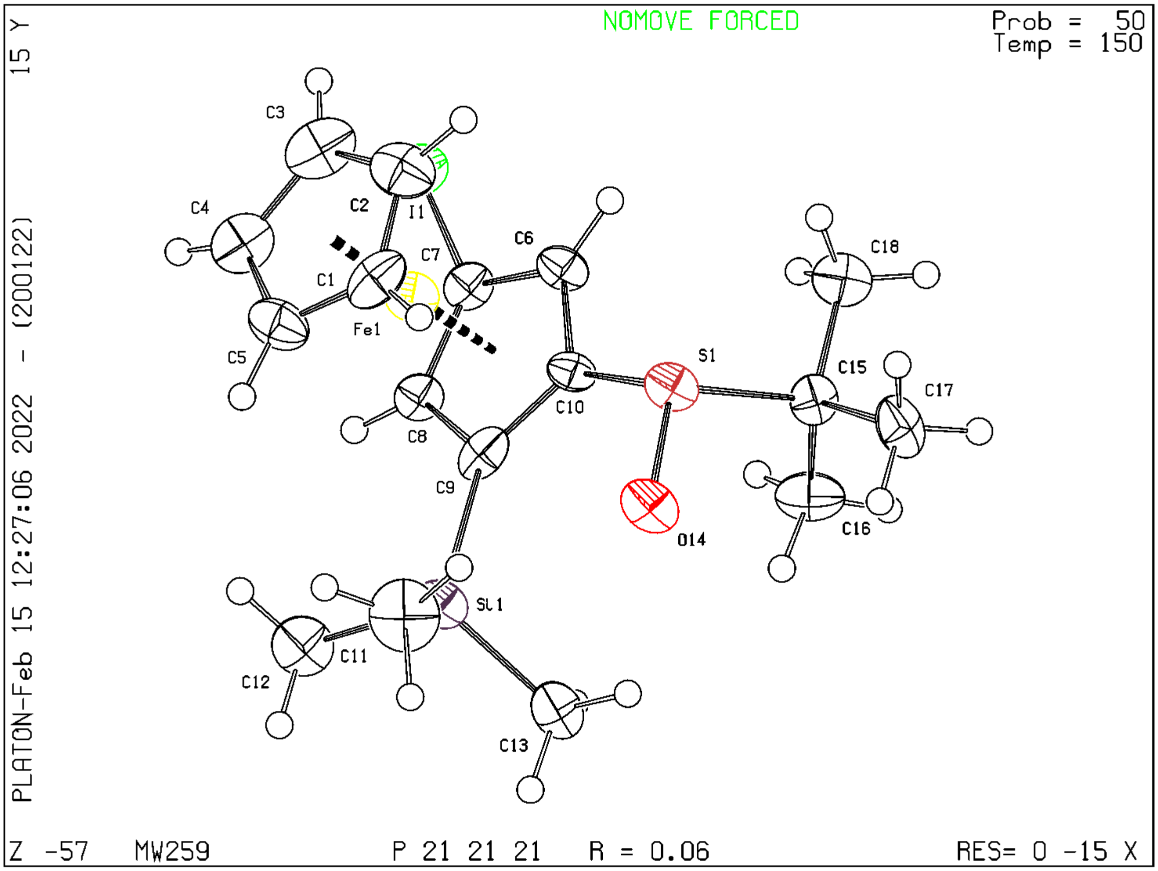

Datablock MW239 - ellipsoid plot

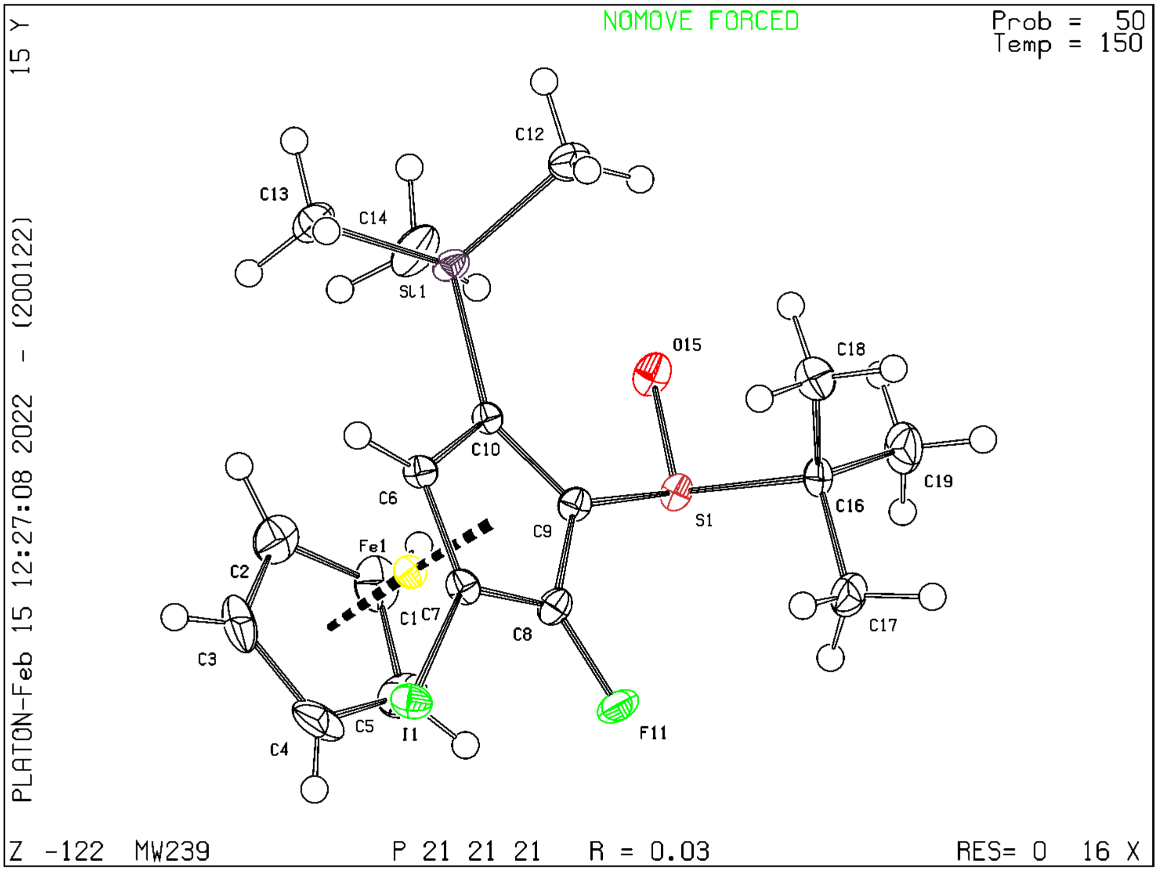

Supplement: Supplementary file 1 [file molecules-27-01798-s001.zip › checkcif.pdf]
