# Supplementary material for: Synthesis of Polysubstituted Ferrocenesulfoxides
Source: Molecules. 2022 Mar 9;27(6):1798. doi: 10.3390/molecules27061798 (PMC8955965; doi:10.3390/molecules27061798)

Supplementary Materials

Synthesis of polysubstituted ferrocenesulfoxides

Min Wen [1], William Erb [1,*], Florence Mongin [1],
Yury S. Halauko [2,*], Oleg A. Ivashkevich [3], Vadim E. Matulis [3] and Thierry Roisnel [1]

[1] *Univ Rennes, CNRS, ISCR (Institut des Sciences Chimiques de Rennes) - UMR 6226, F-35000 Rennes, France*

[2] *UNESCO Chair of Belarusian State University, 220030 Minsk, Belarus*

[3] *Research Institute for Physico-Chemical Problems of Belarusian State University, 220030 Minsk, Belarus*

william.erb@univ-rennes1.fr (W. Erb); hys@tut.by (Y. S. Halauko)

Table of contents

NMR Spectra S2
Selected NOESY correlations S153
Calculated values of the Gibbs energies ΔG_{acid} for deprotonation S156

NMR Spectra

Diferrocenesulfoxide (FcSOFc)

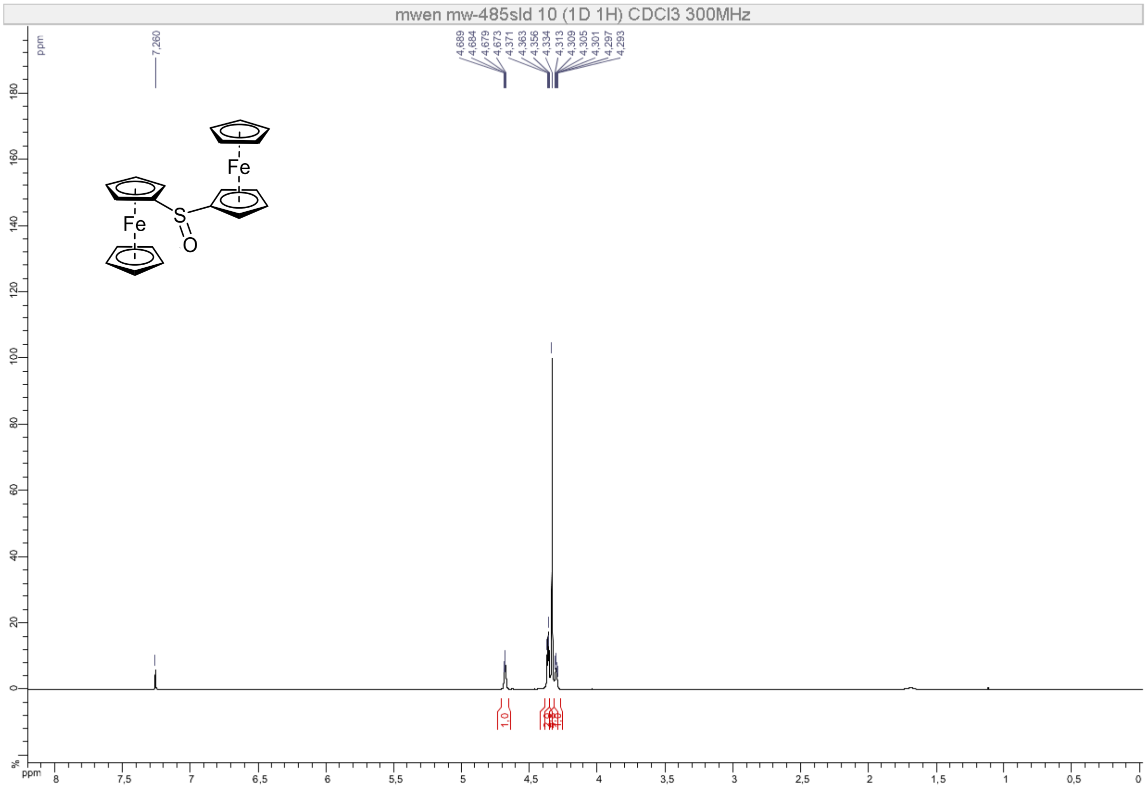

(*tert*-Butylthio)ferrocene

^{1}H NMR (500 MHz, $CDCl_3$)

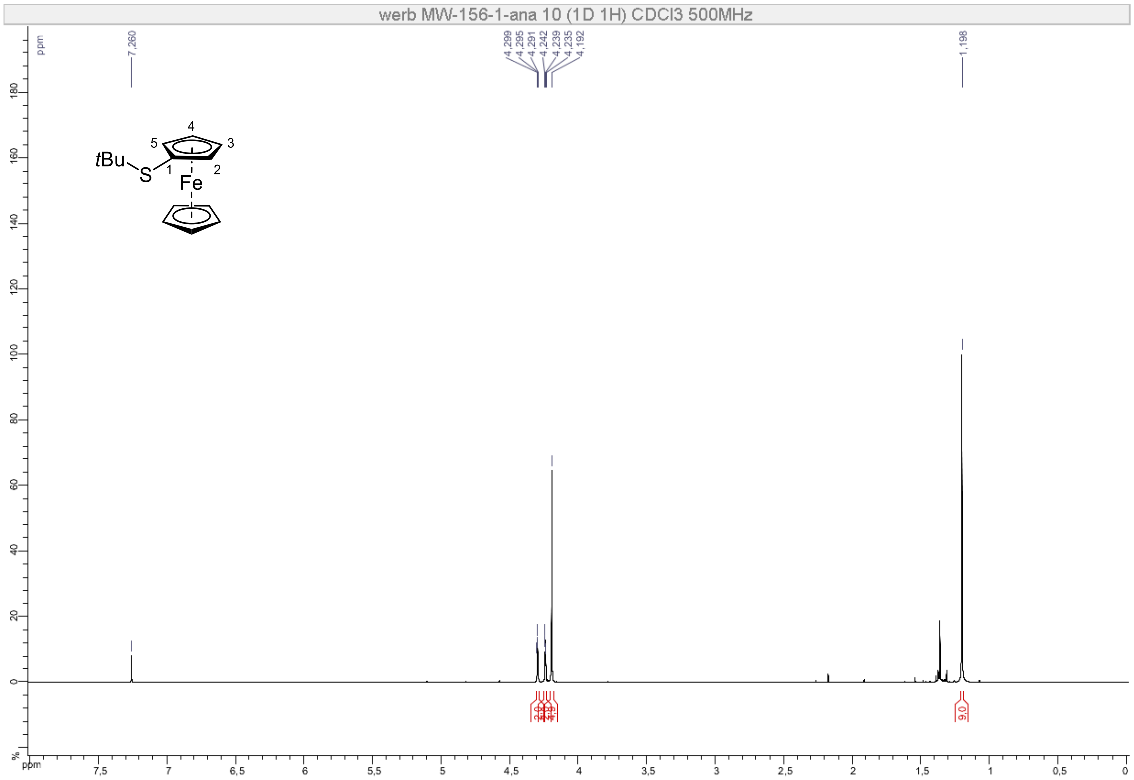

^{13}C NMR (126 MHz, $CDCl_3$)

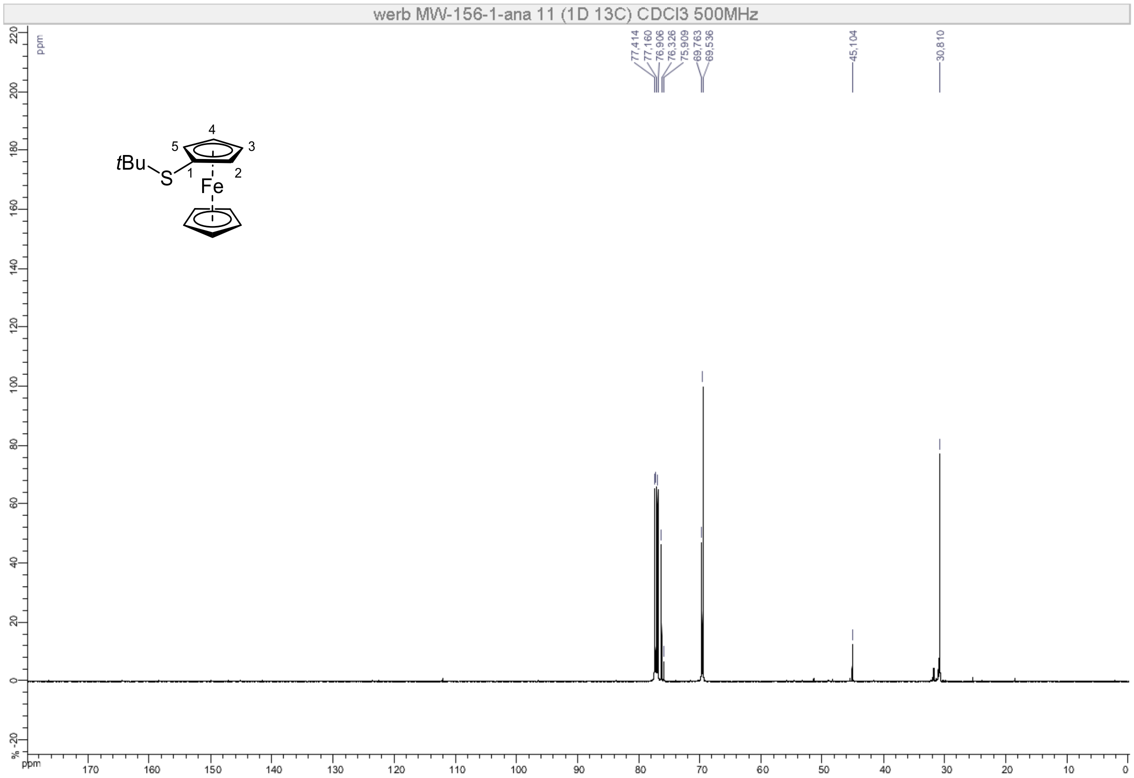

DEPT 135 (126 MHz, $CDCl_3$)

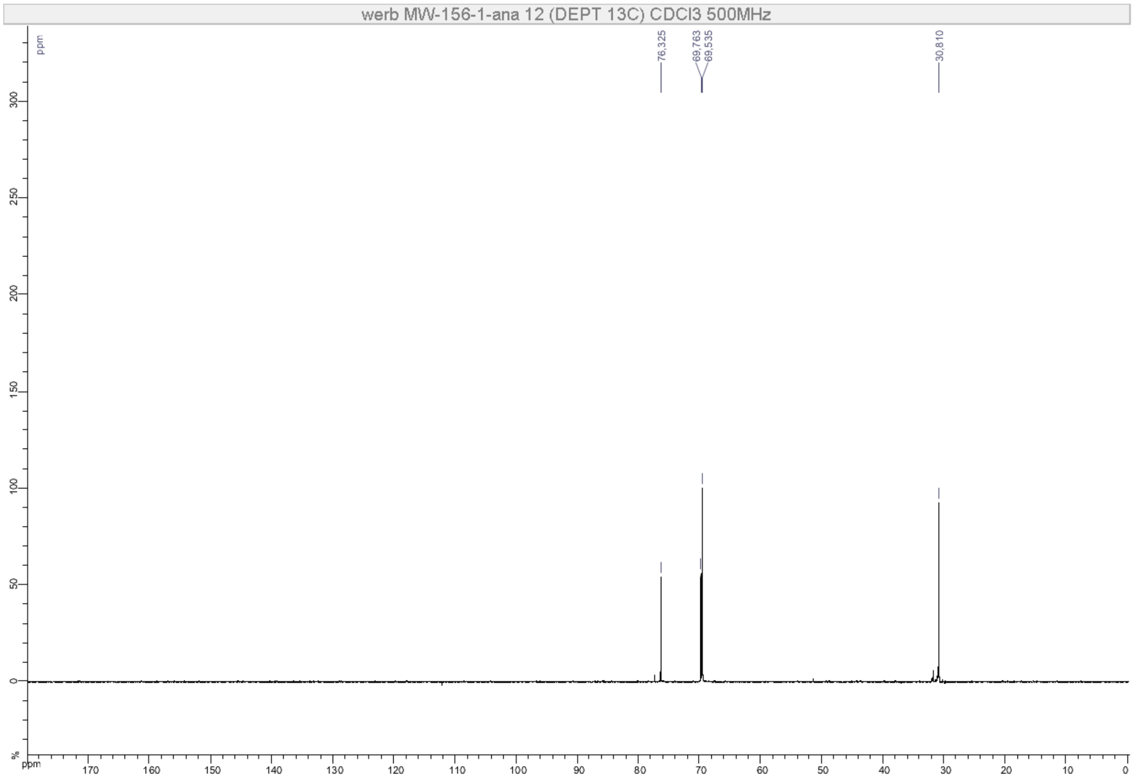

COSY (500 MHz, $CDCl_3$)

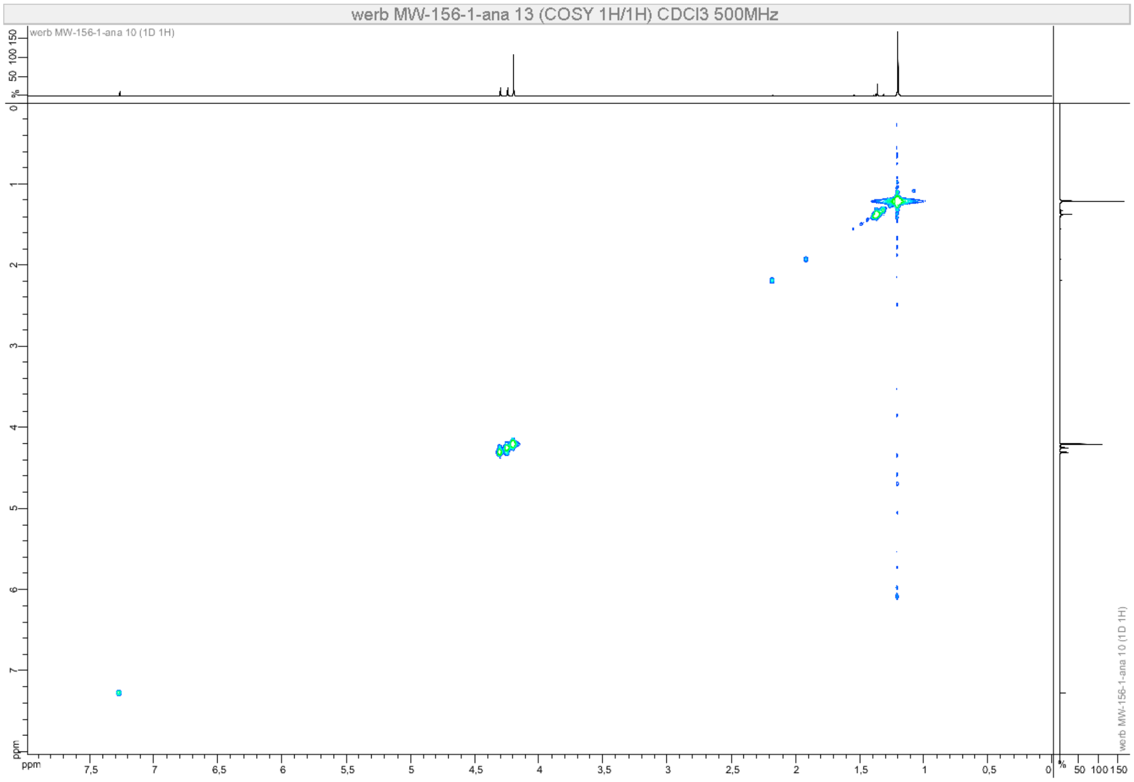

HSQC (500 MHz, $CDCl_3$)

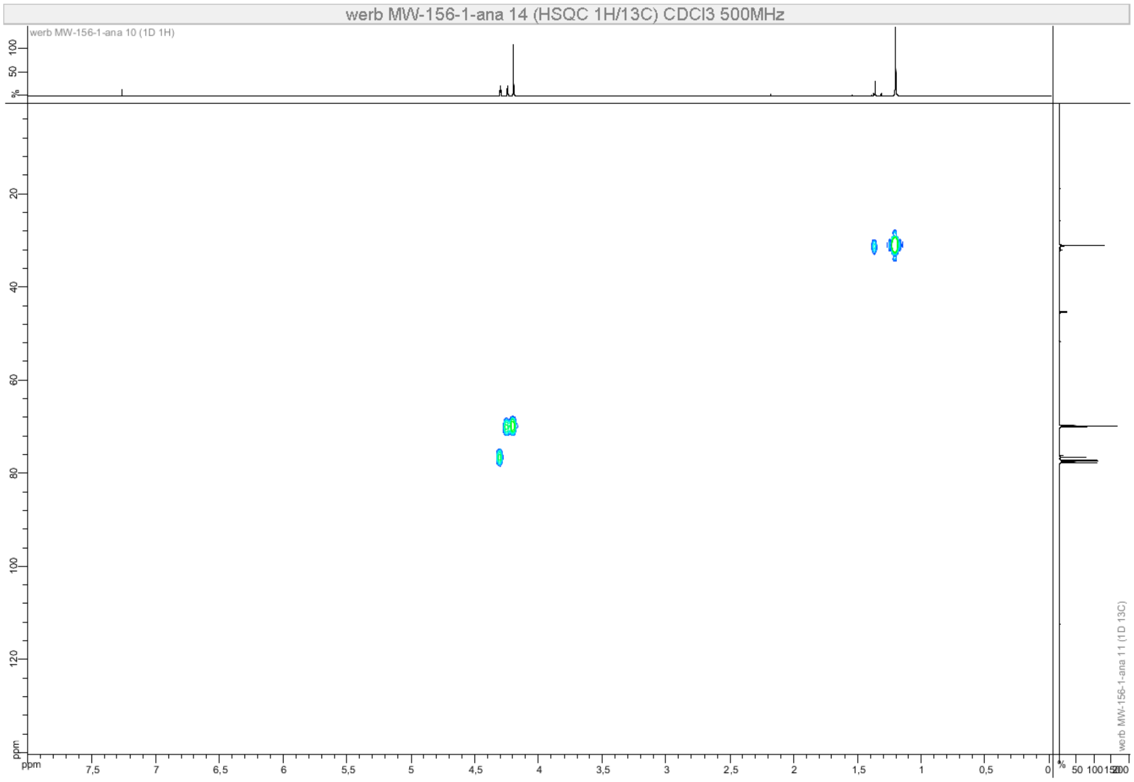

HMBC (500 MHz, $CDCl_3$)

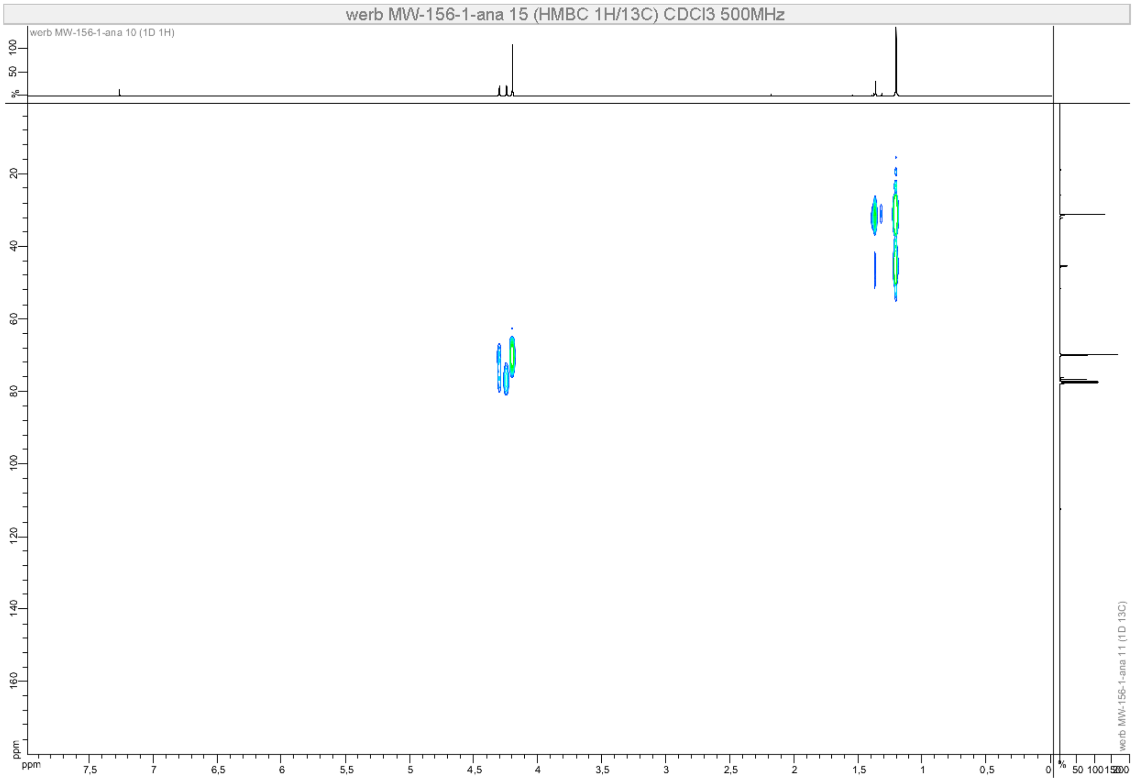

NOESY (500 MHz, $CDCl_3$)

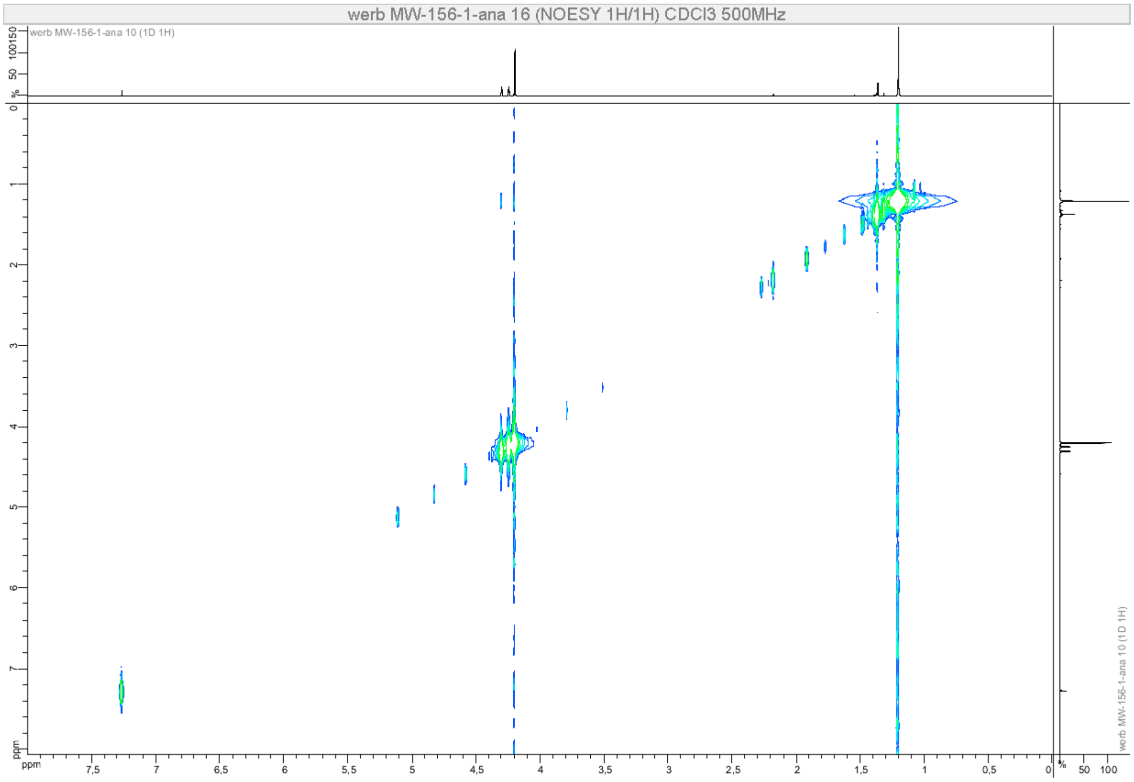

Racemic *S-tert*-Butylferrocenesulfoxide (*rac*-FcSO*t*Bu)

^{1}H NMR (300 MHz, $CDCl_3$)

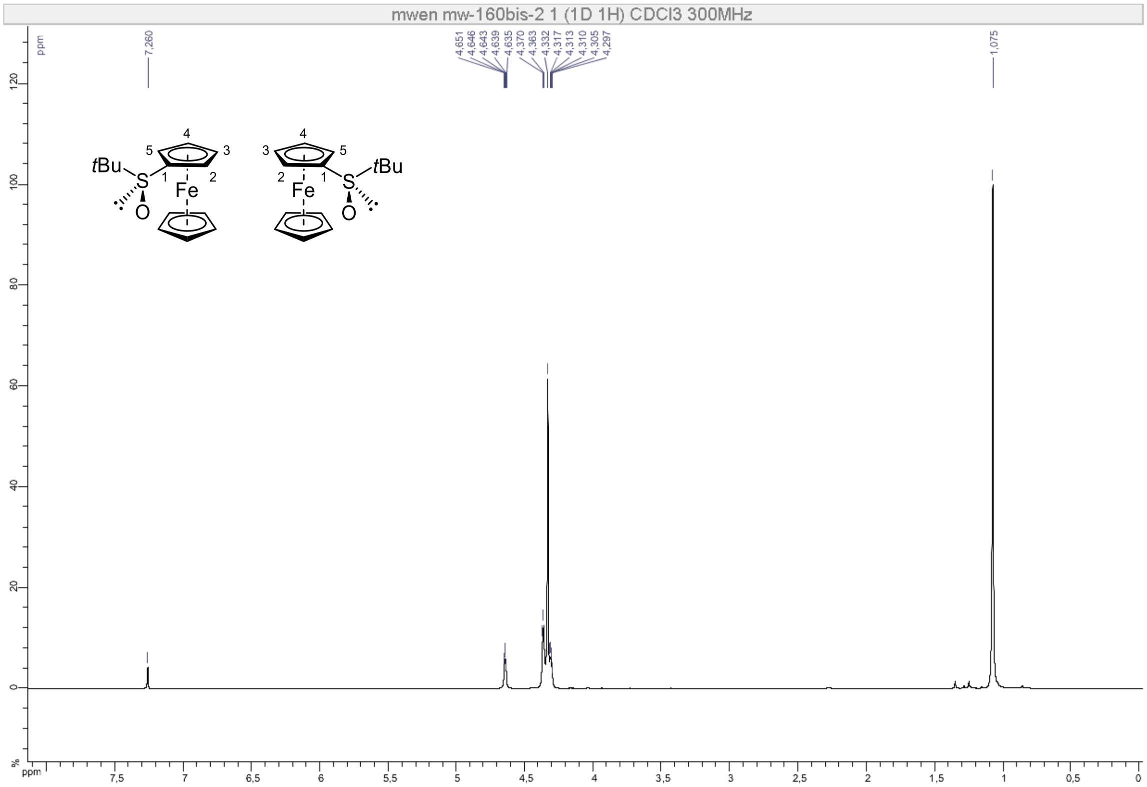

(4-Tolylthio)ferrocene

^{1}H NMR (500 MHz, $CDCl_3$)

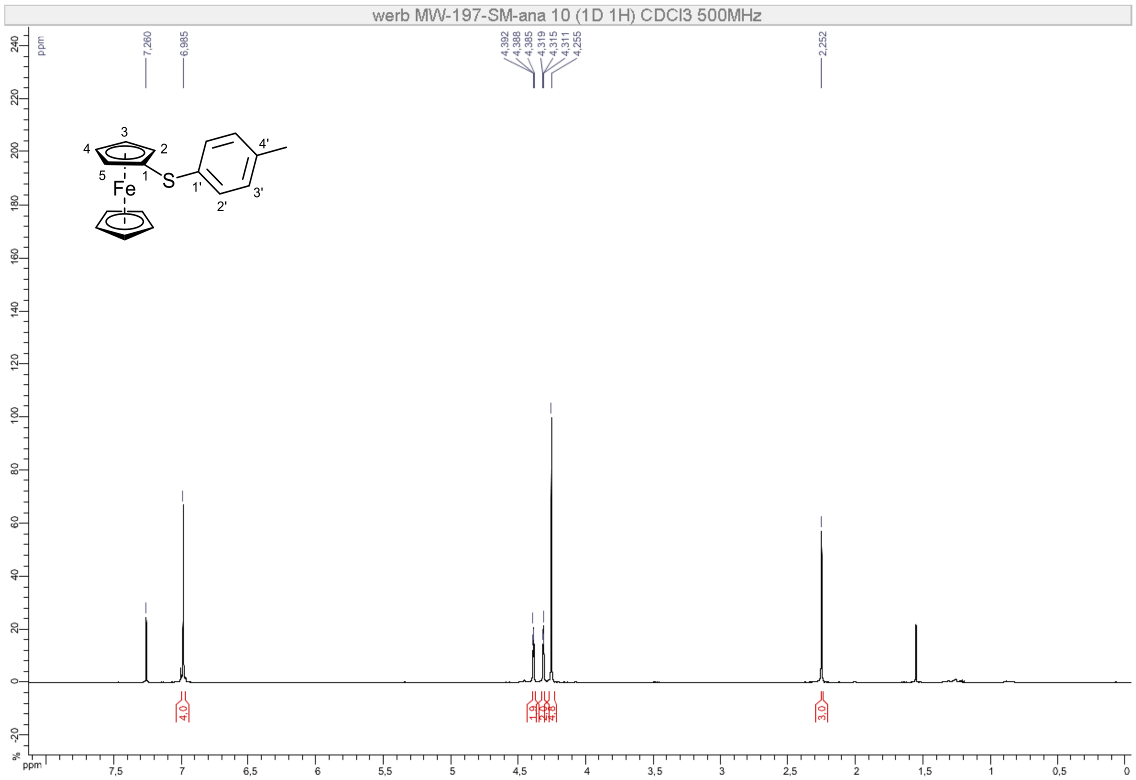

^{13}C NMR (126 MHz, $CDCl_3$)

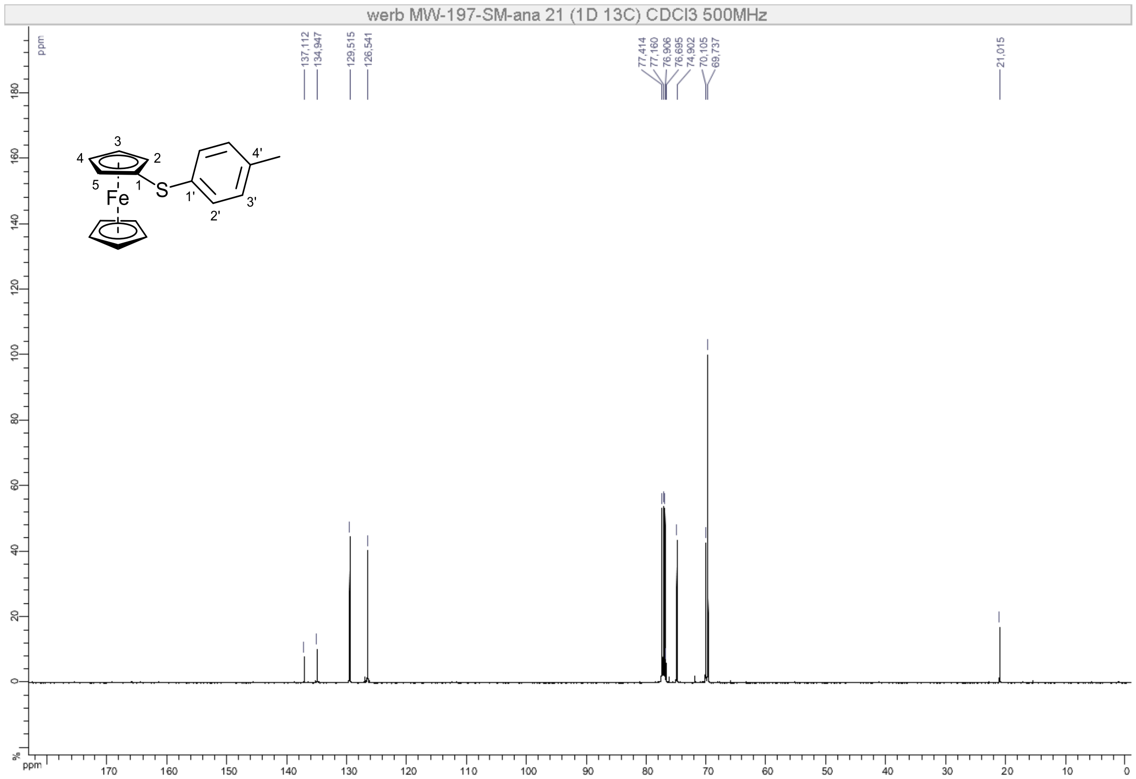

COSY (500 MHz, $CDCl_3$)

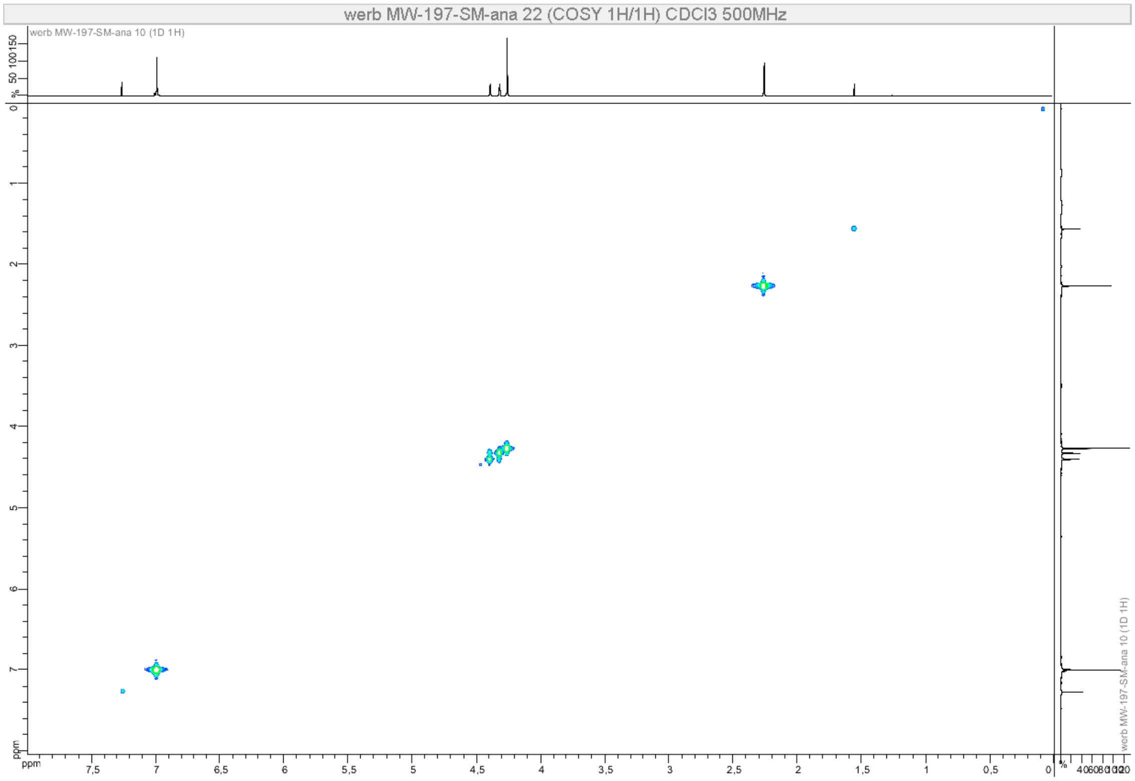

HSQC (500 MHz, $CDCl_3$)

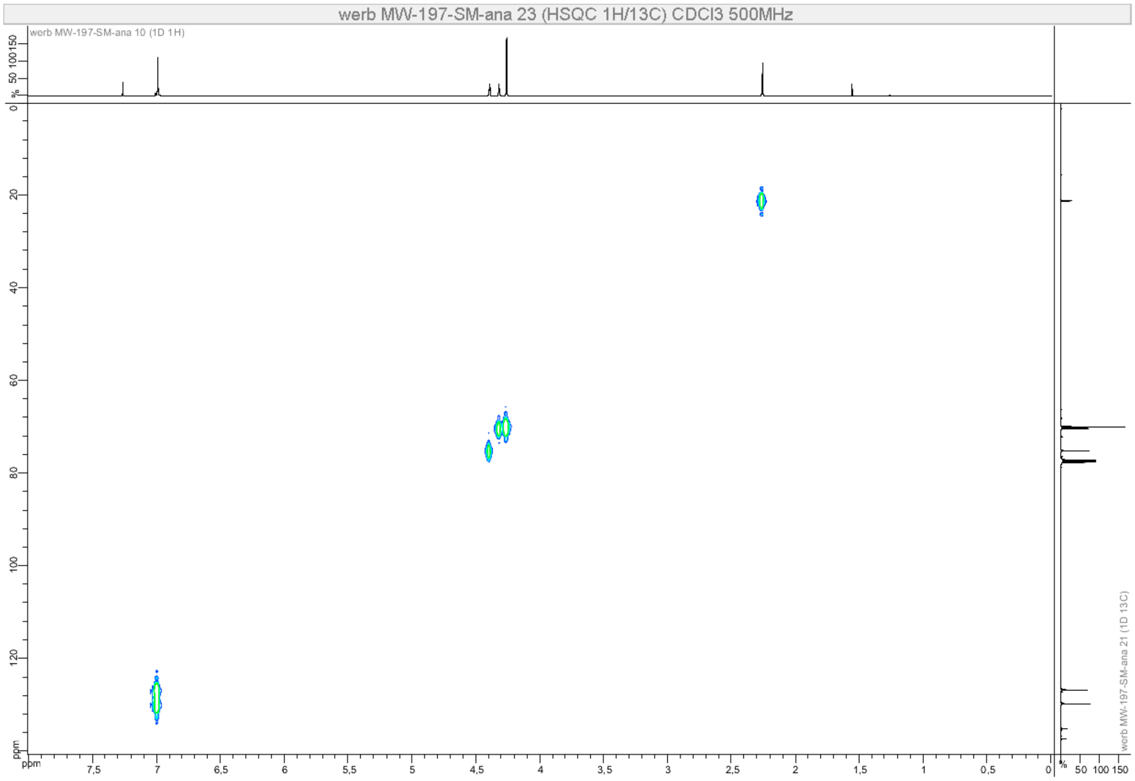

HMBC (500 MHz, $CDCl_3$)

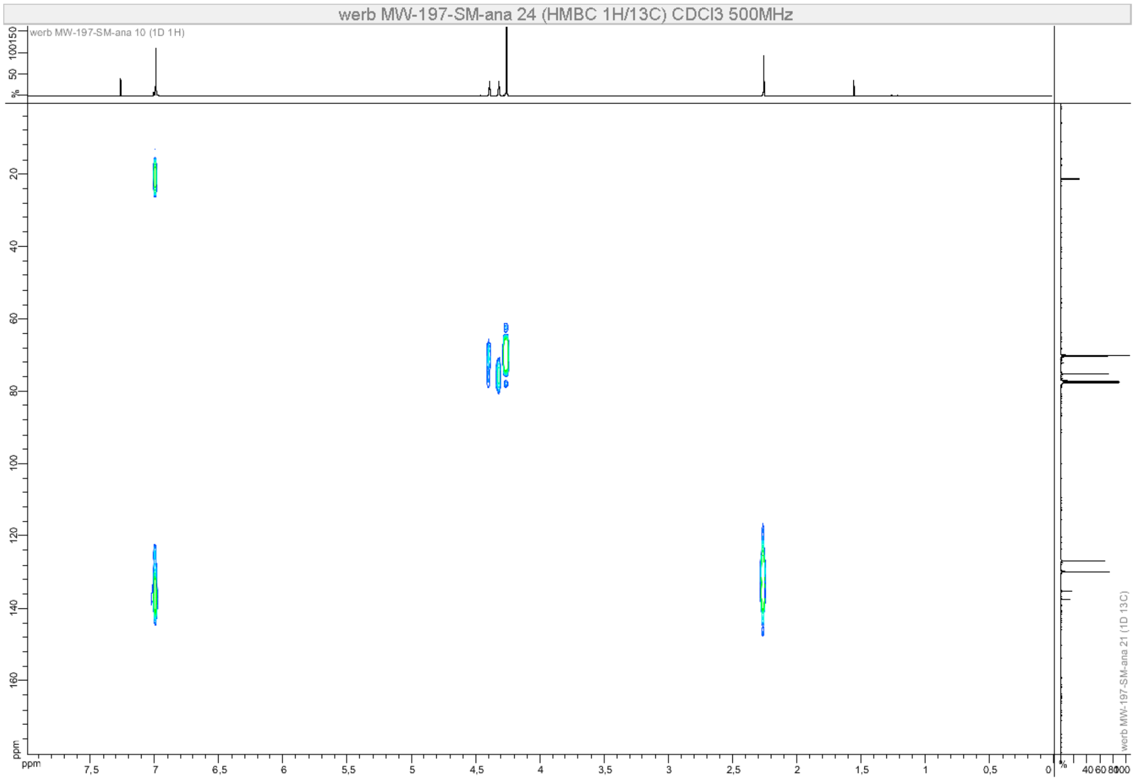

NOESY (500 MHz, $CDCl_3$)

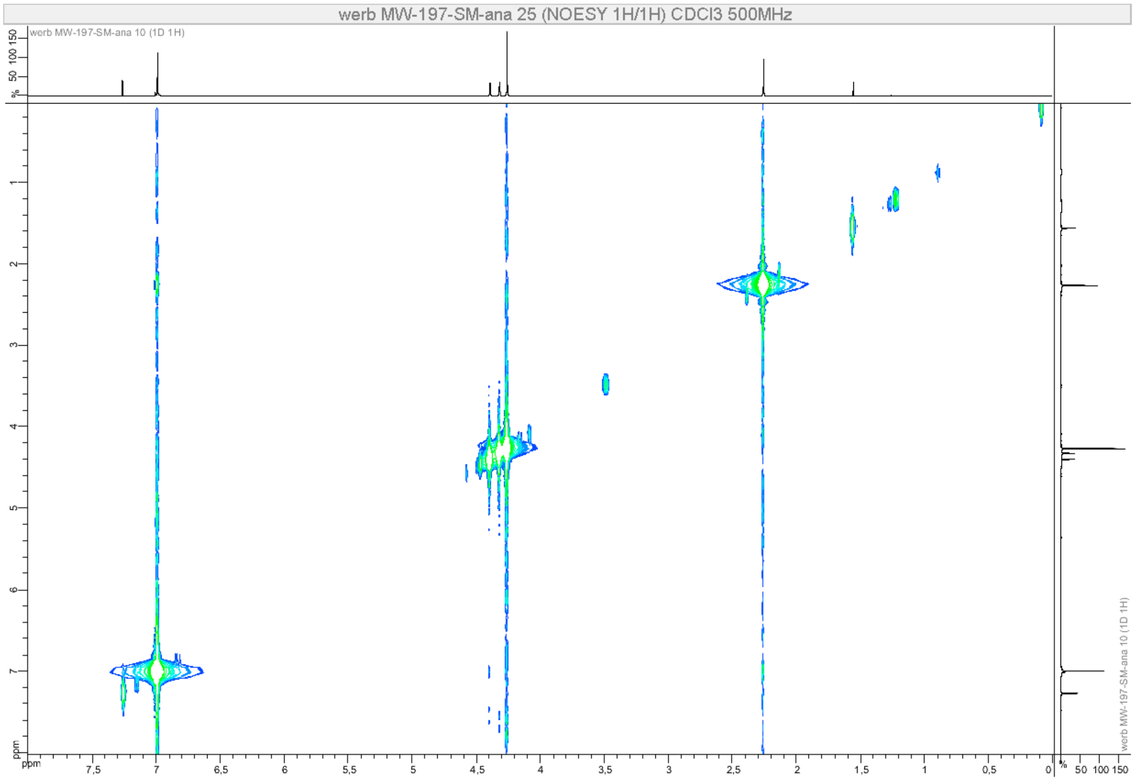

Racemic *S*-(4-Tolyl)ferrocenesulfoxide (*rac*-FcSO-*p*-Tol)

^{1}H NMR (300 MHz, $CDCl_3$)

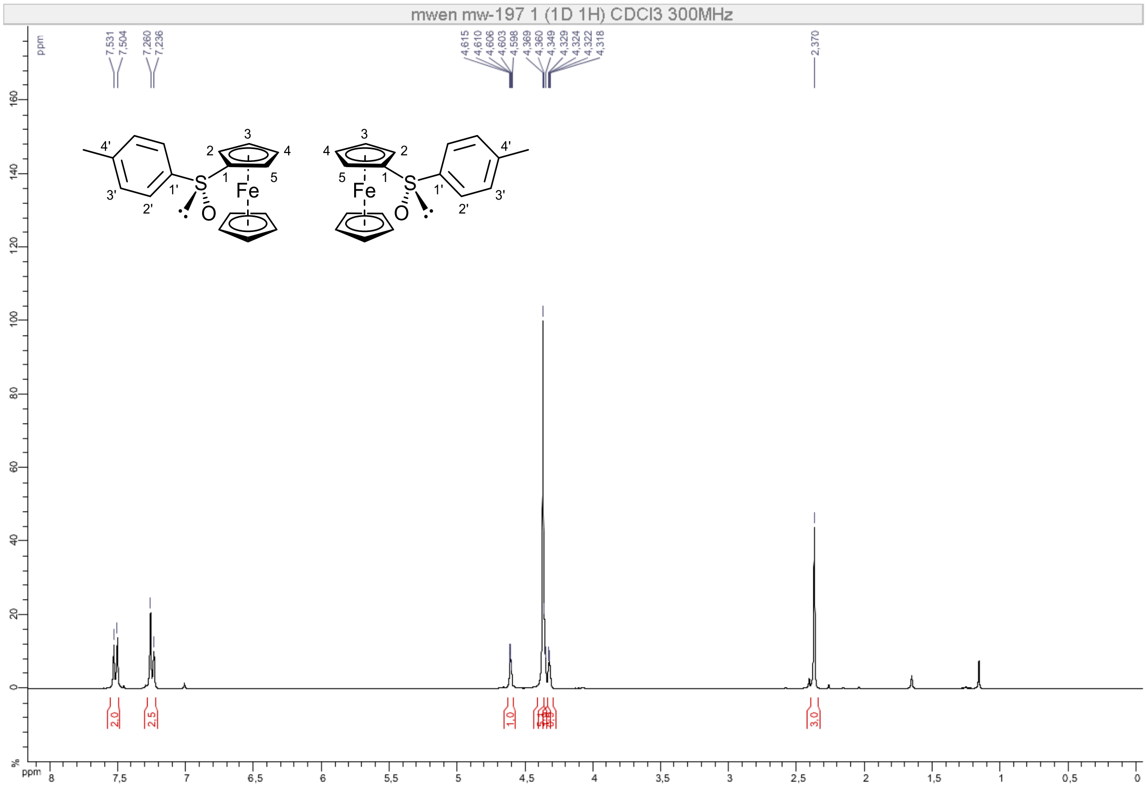

(*S*,*S*P)-*S*-*tert*-Butyl-2-(trimethylsilyl)ferrocenesulfoxide (*S*,*S*P-1a)

^{1}H NMR (500 MHz, $CDCl_3$)

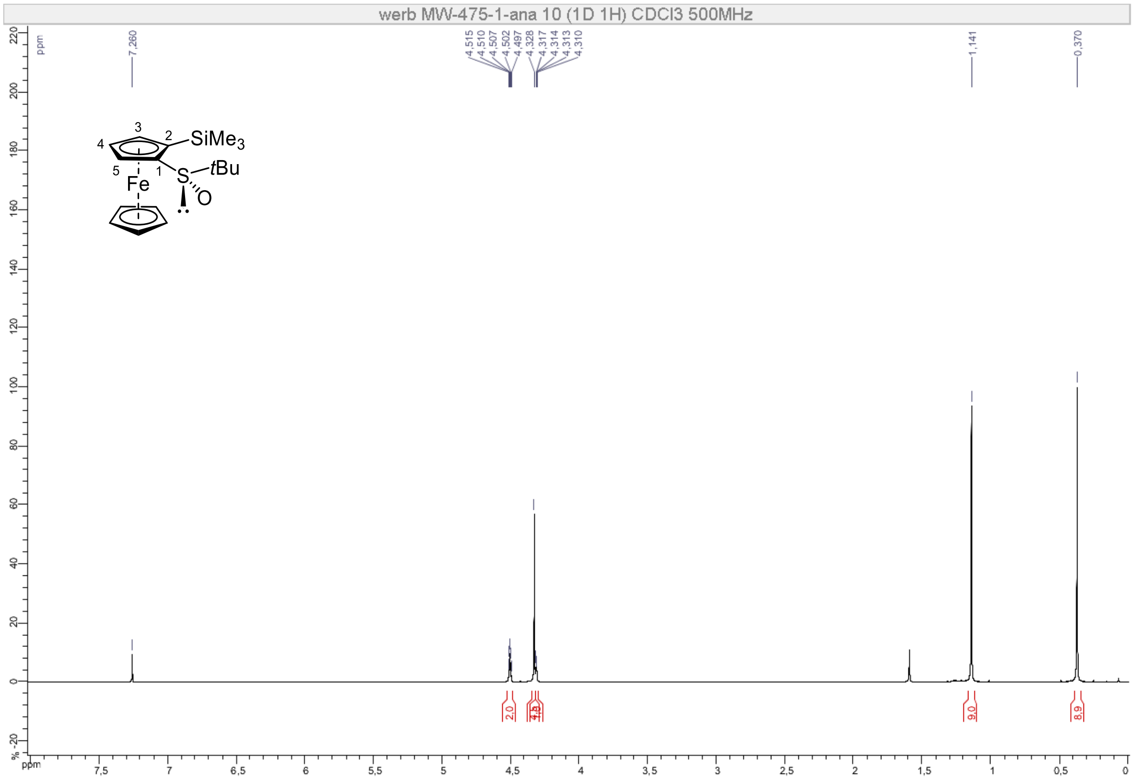

^{13}C NMR (126 MHz, $CDCl_3$)

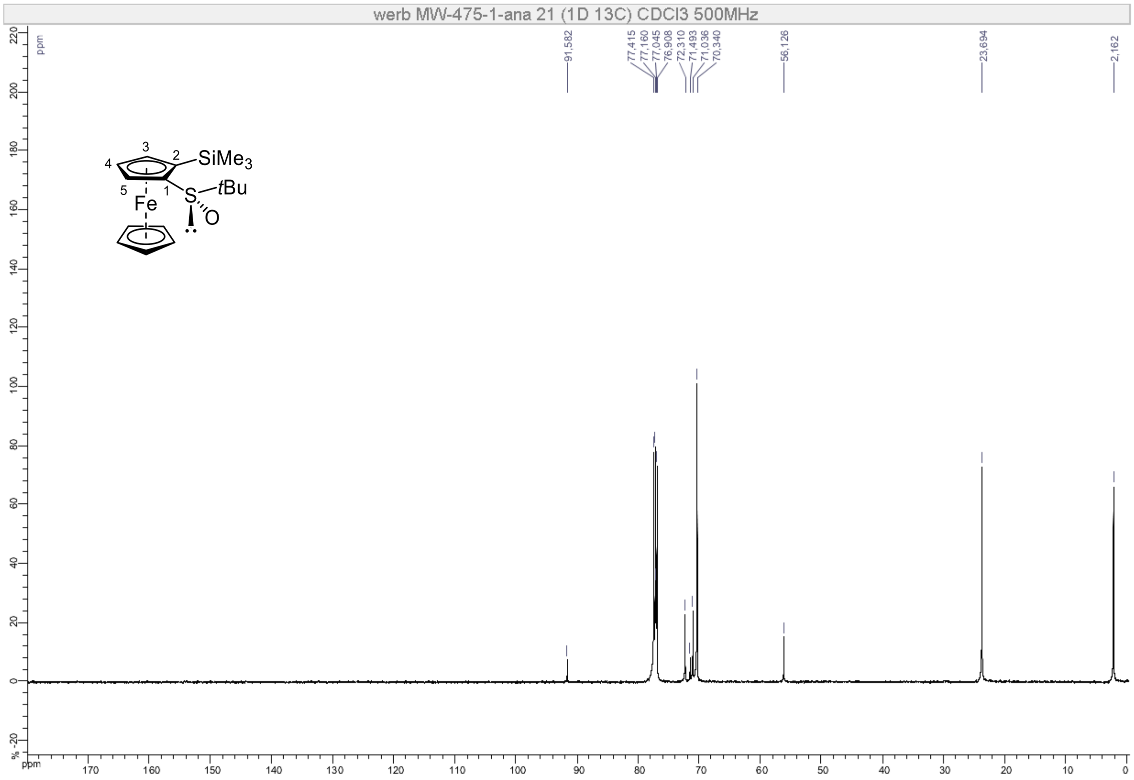

COSY (500 MHz, $CDCl_3$)

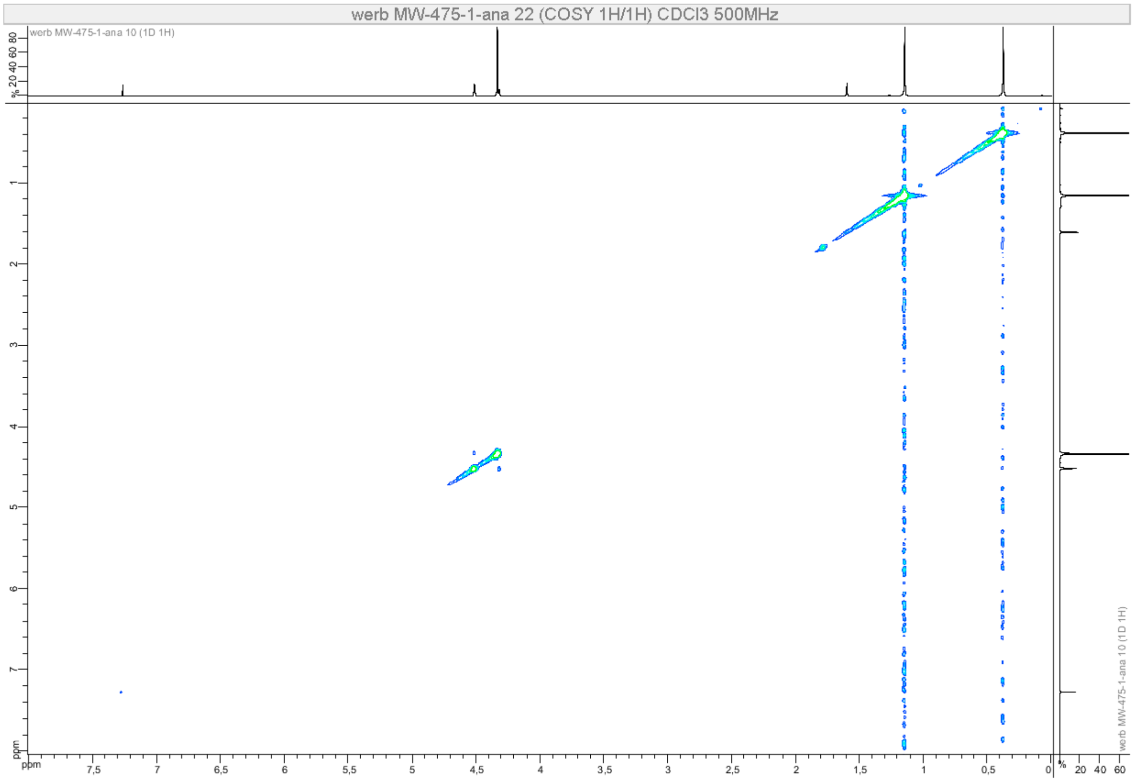

HSQC (500 MHz, $CDCl_3$)

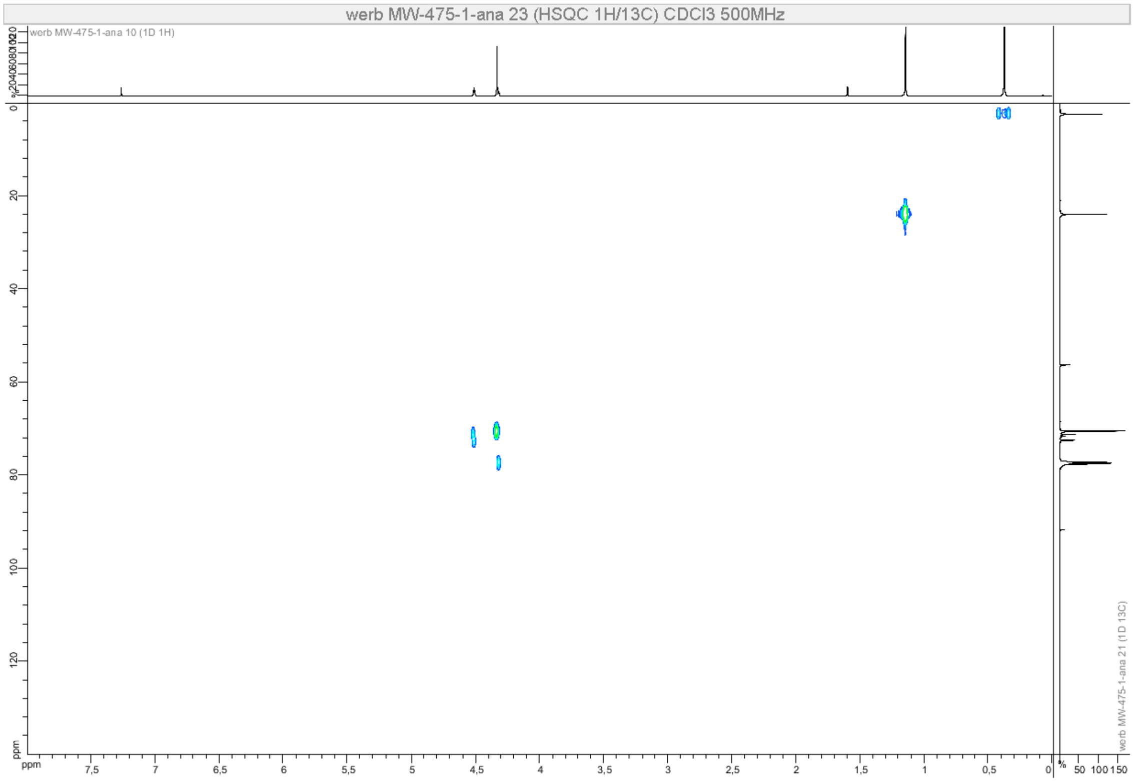

HMBC (500 MHz, $CDCl_3$)

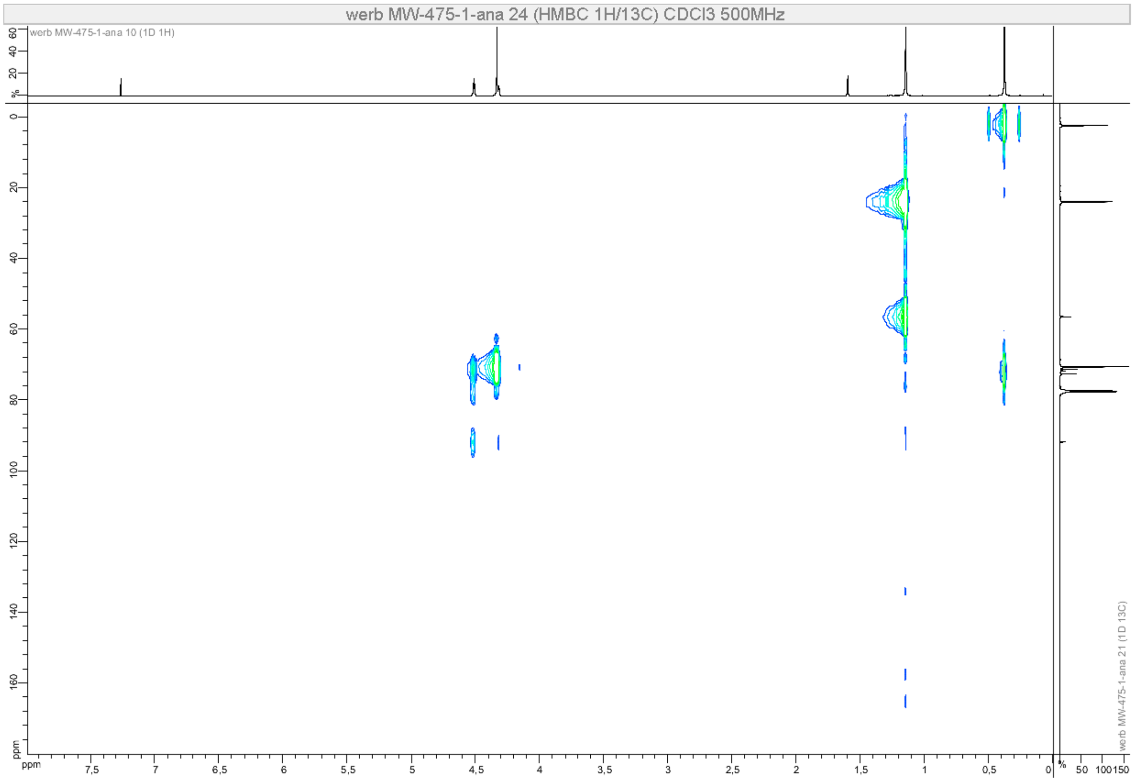

NOESY (500 MHz, $CDCl_3$)

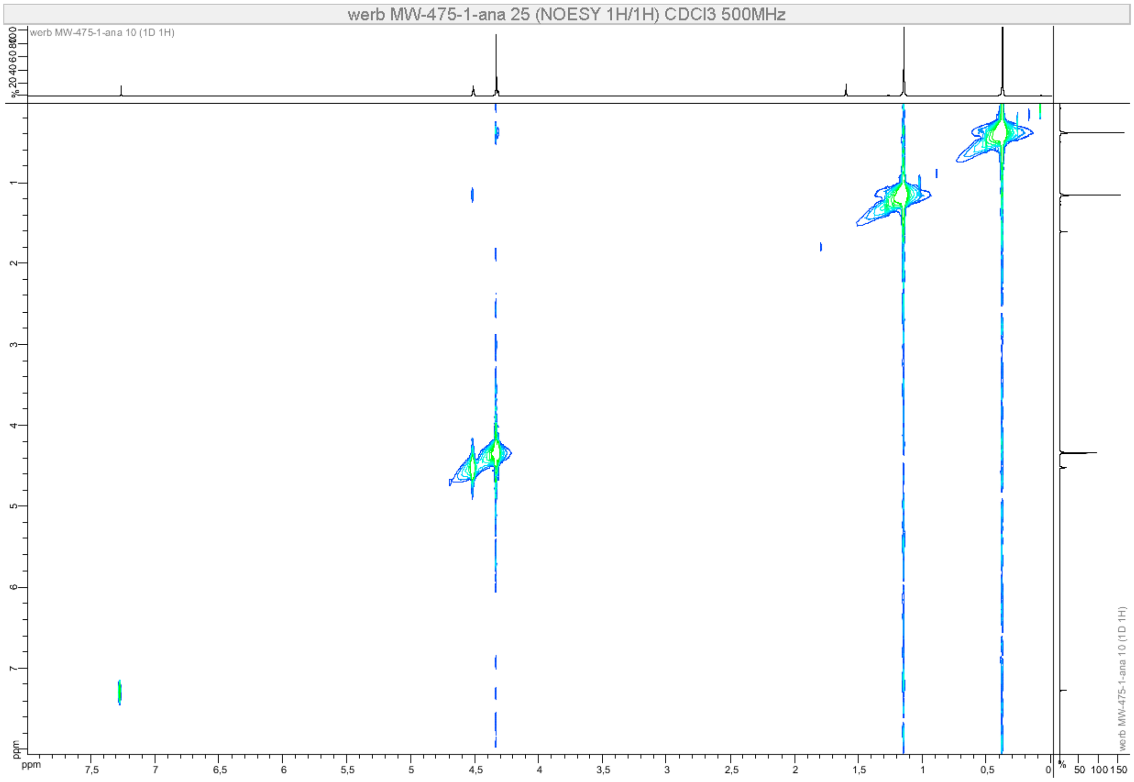

(*R*,*R*P)-*S*-*tert*-Butyl-2-(trimethylsilyl)ferrocenesulfoxide (*R*,*R*P-1a)

^{1}H NMR (300 MHz, $CDCl_3$)

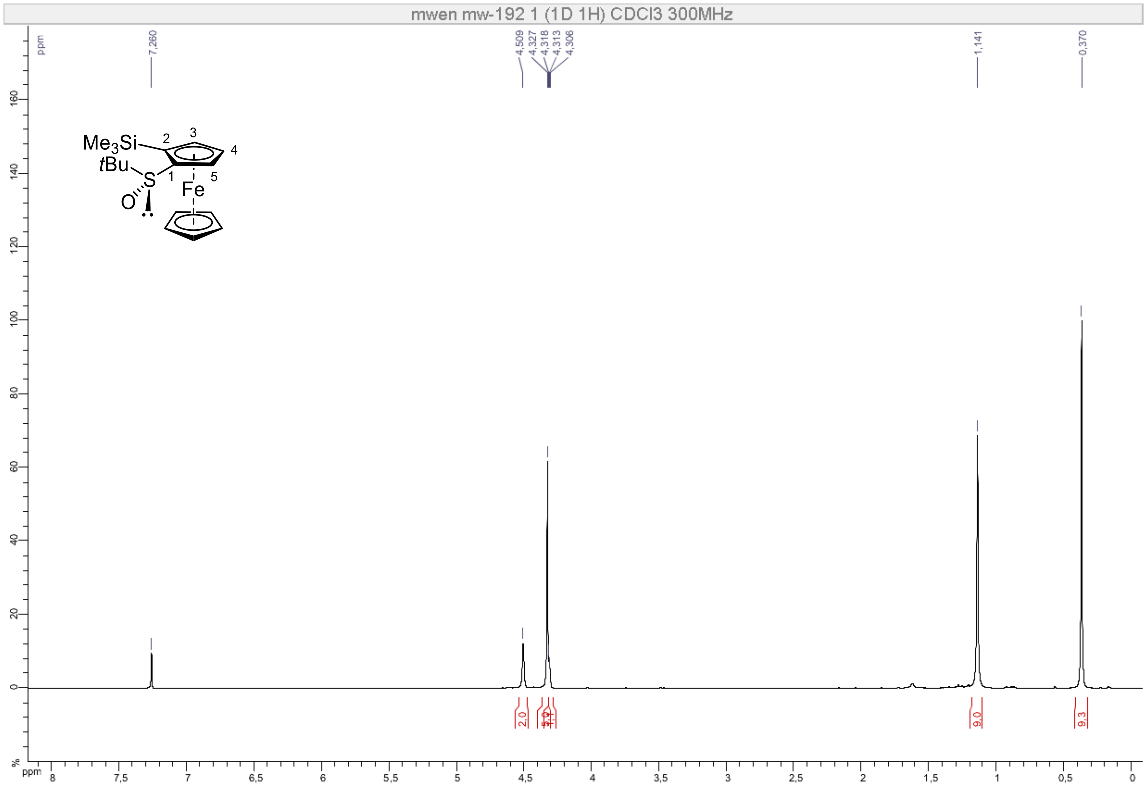

(*R*,*R*P)- and (*S*,*S*P)-*S*-*tert*-Butyl-2-(trimethylsilyl)ferrocenesulfoxide (*R*,*R*P/*S*,*S*P-1a)

^{1}H NMR (300 MHz, $CDCl_3$)

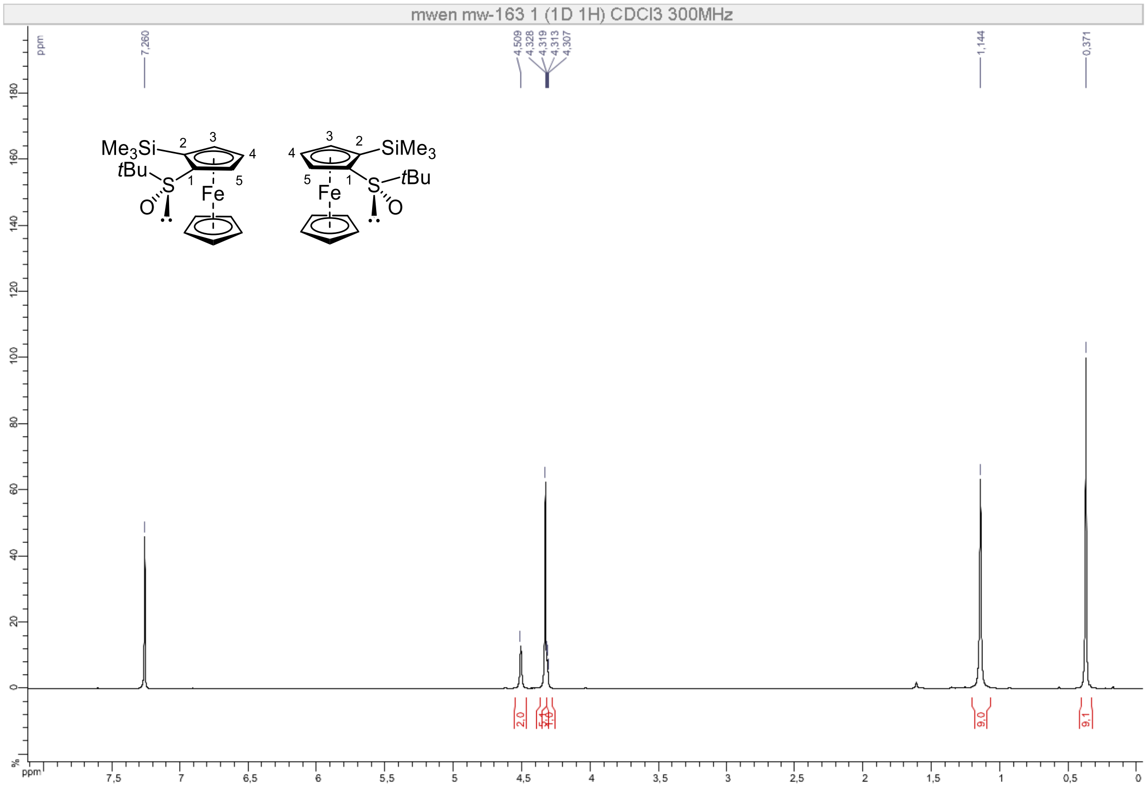

(S,R_P)-*S*-*tert*-Butyl-2-iodoferrocenesulfoxide (S,R_P-1b)

^{1}H NMR (500 MHz, $CDCl_3$)

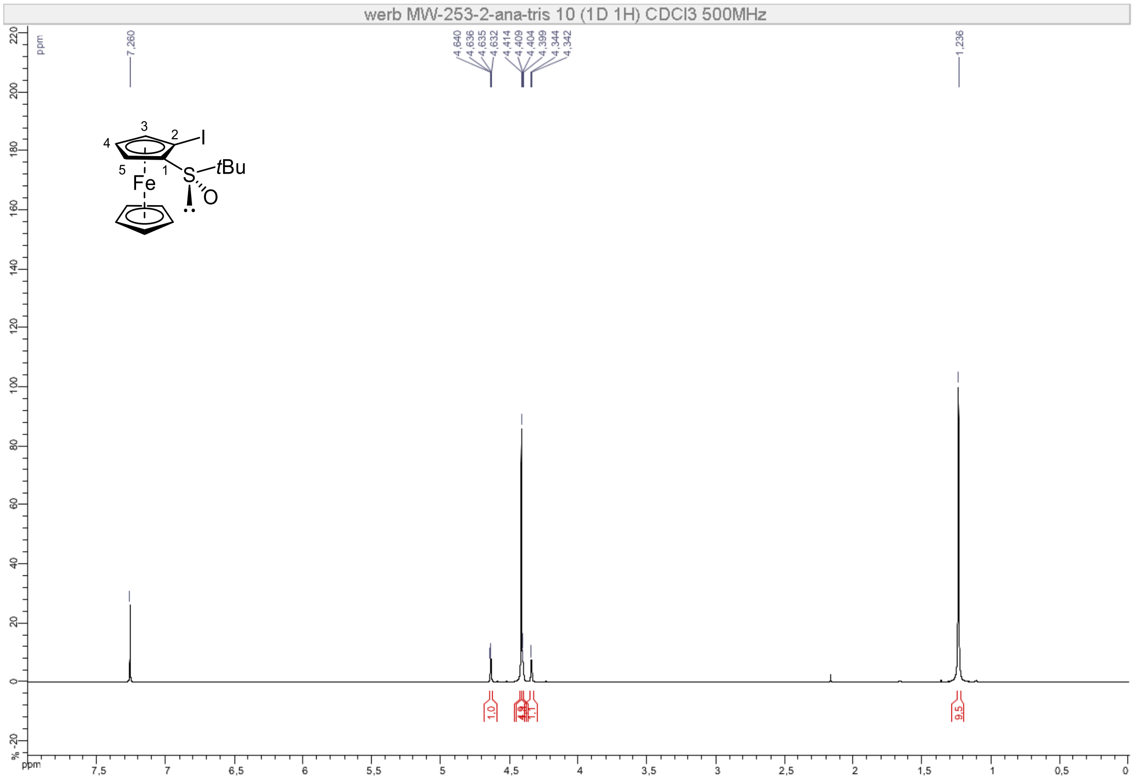

^{13}C NMR (126 MHz, $CDCl_3$)

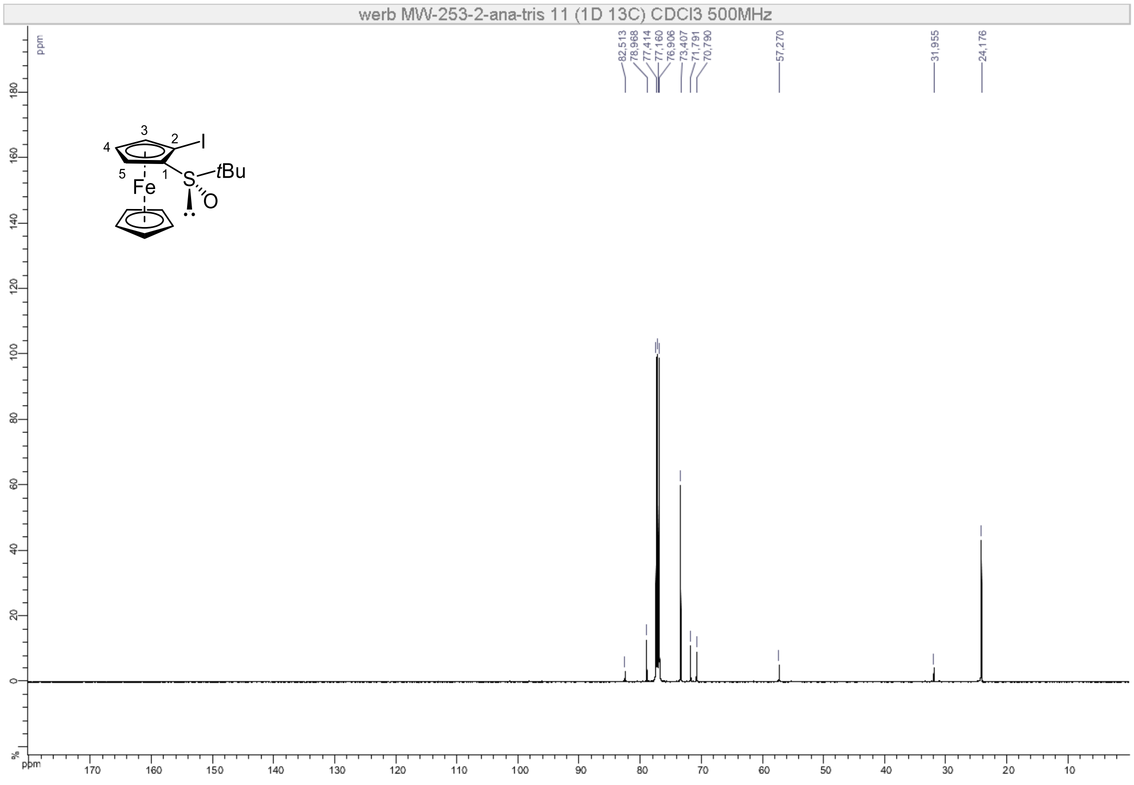

DEPT 135 (126 MHz, $CDCl_3$)

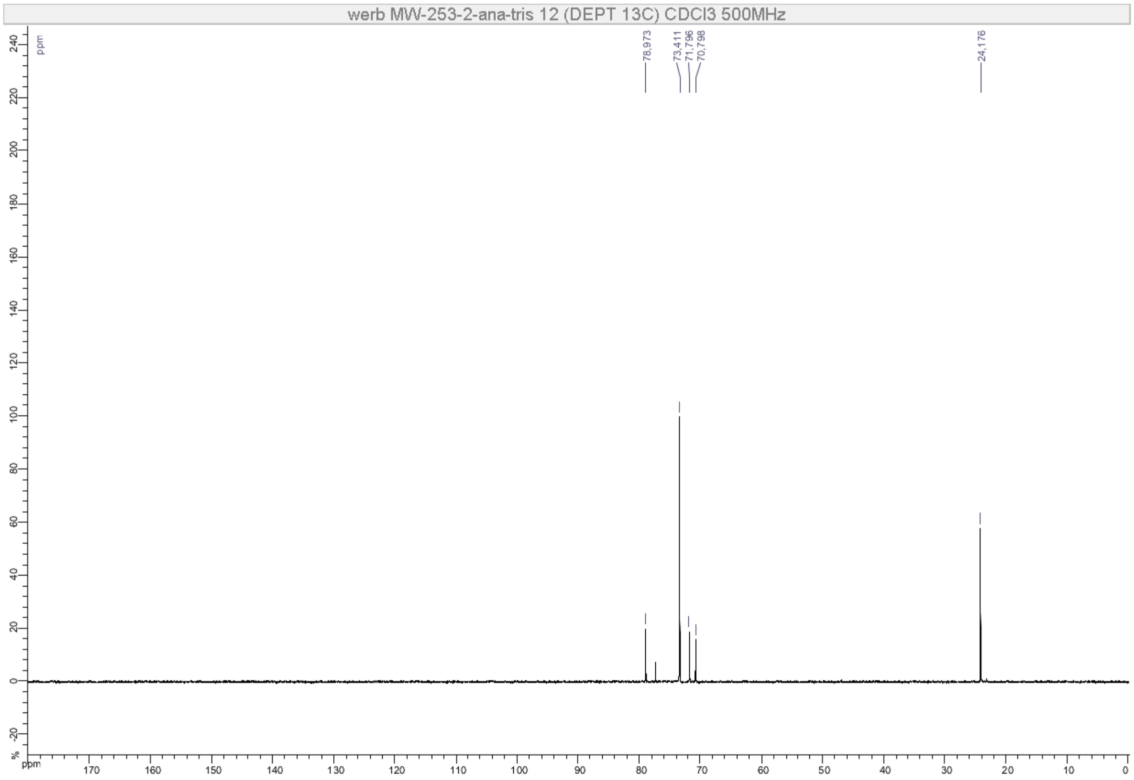

COSY (500 MHz, $CDCl_3$)

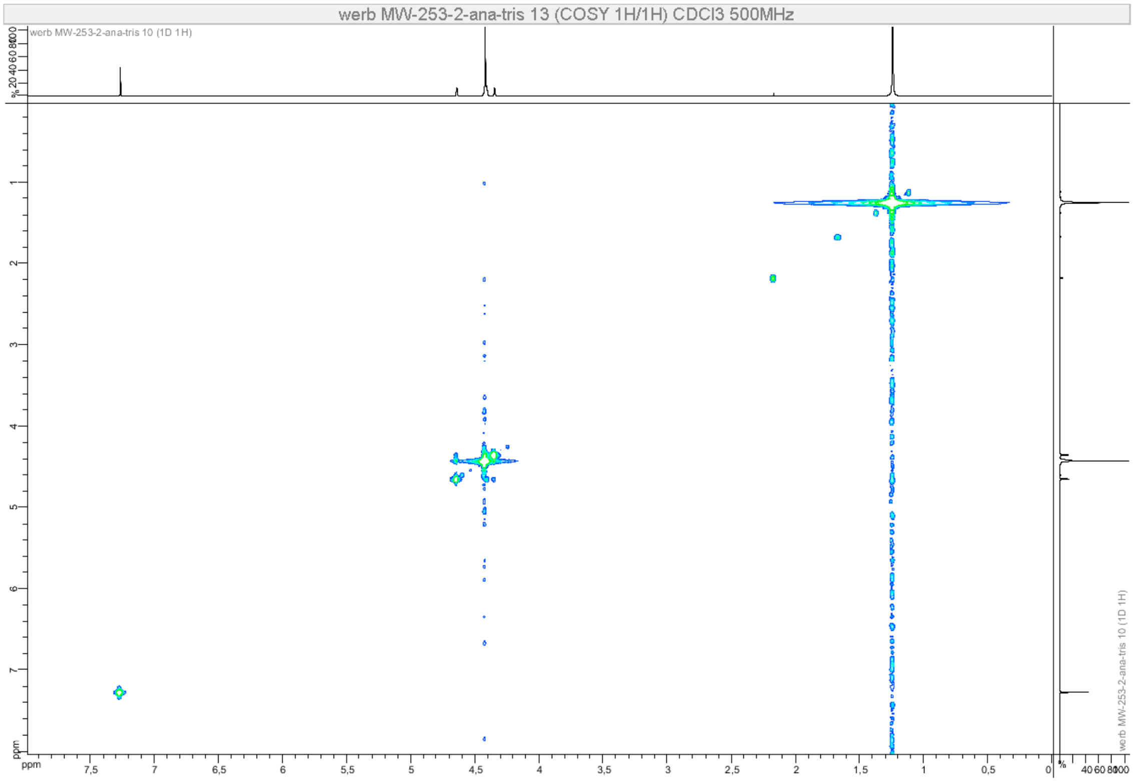

HSQC (500 MHz, $CDCl_3$)

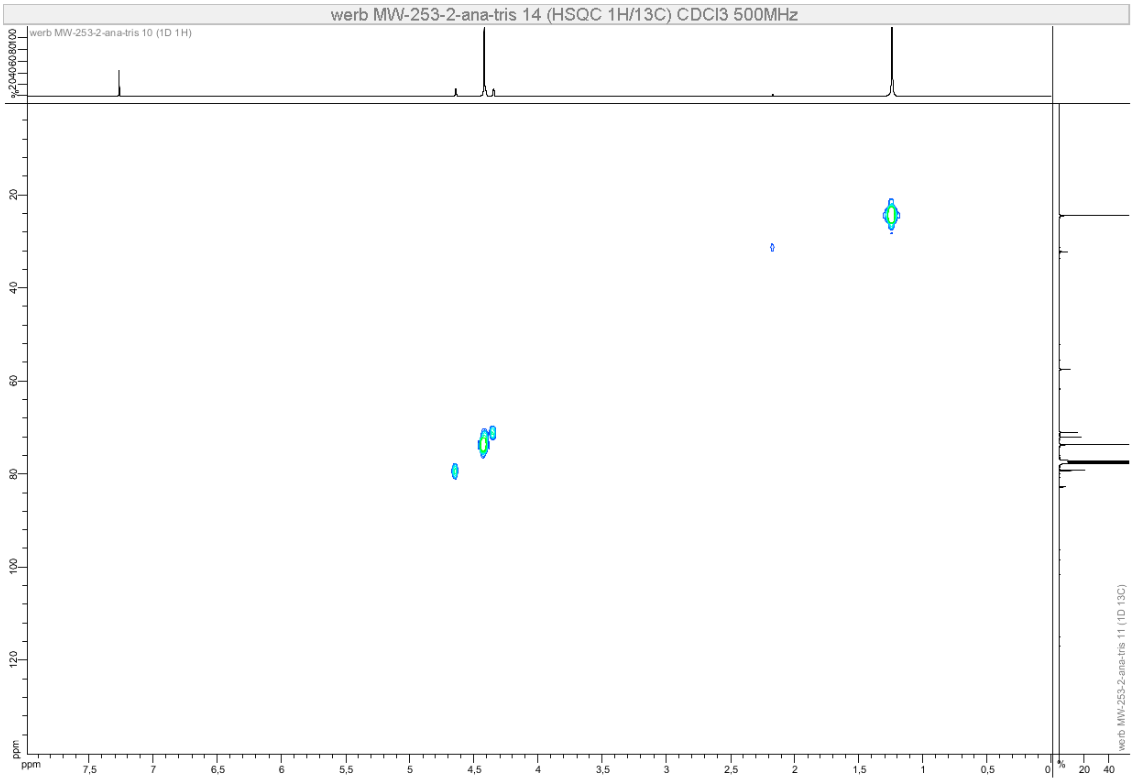

HMBC (500 MHz, $CDCl_3$)

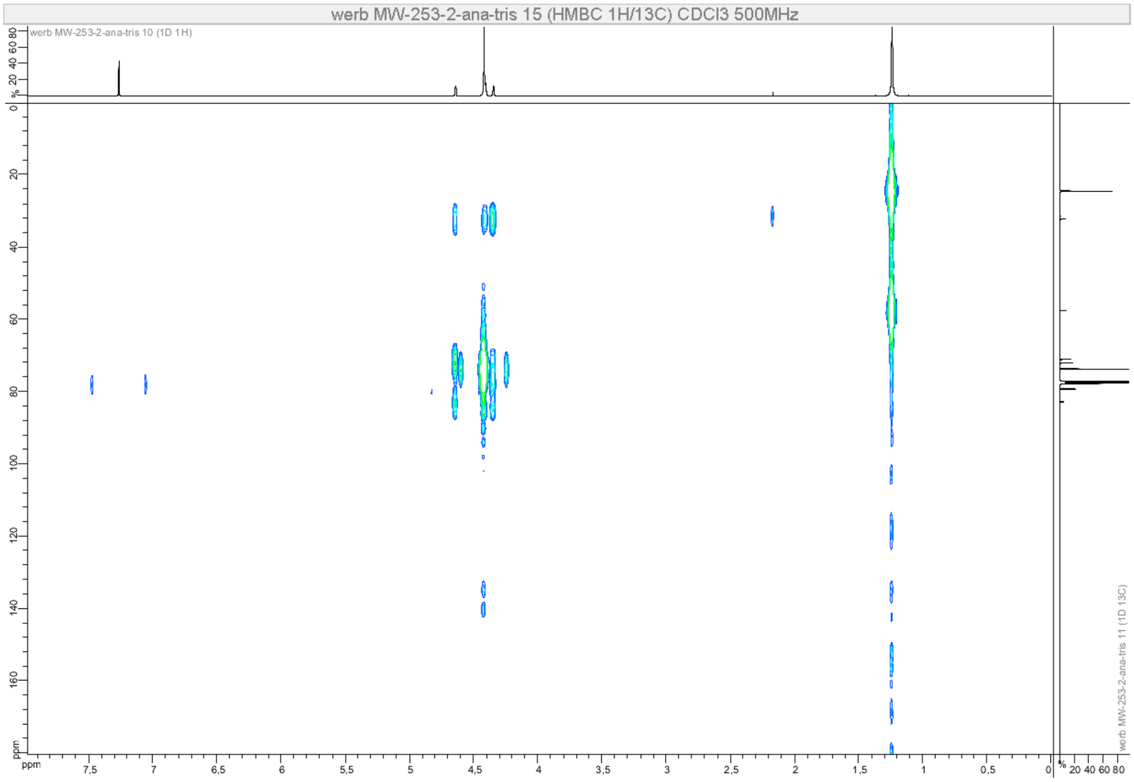

NOESY (500 MHz, $CDCl_3$)

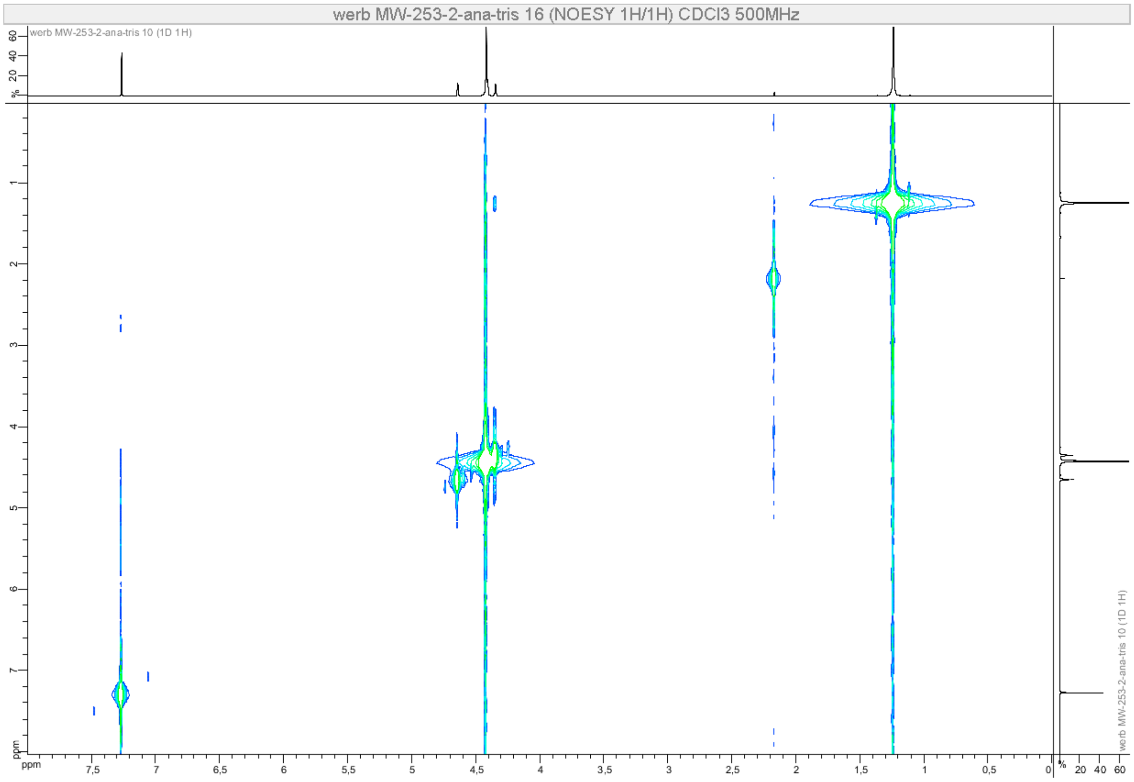

(*S*,*R*P)- and (*R*,*S*P)-*S*-*tert*-Butyl-2-iodoferrocenesulfoxide (*S*,*R*P/*R*,*S*P-1b)

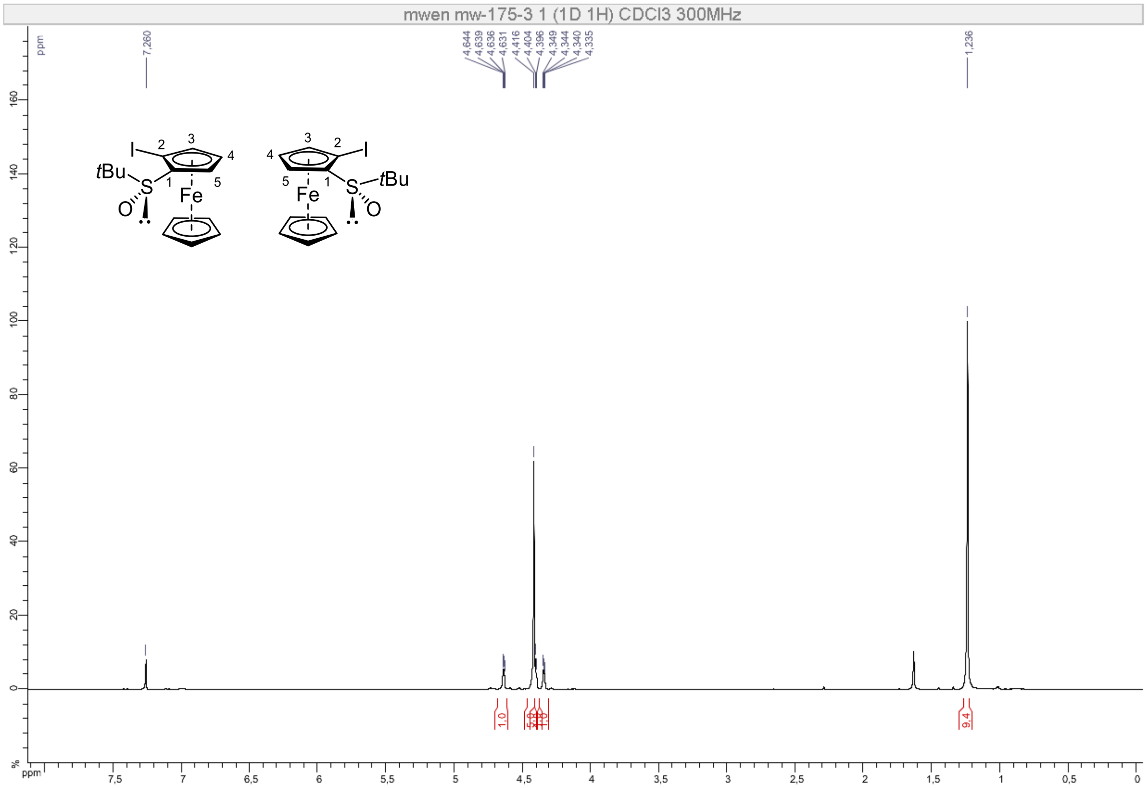

(*S*,*S*P)-*S*-*tert*-Butyl-2-deuterioferrocenesulfoxide (*S*,*S*P-1c)

^{1}H NMR (500 MHz, $CDCl_3$)

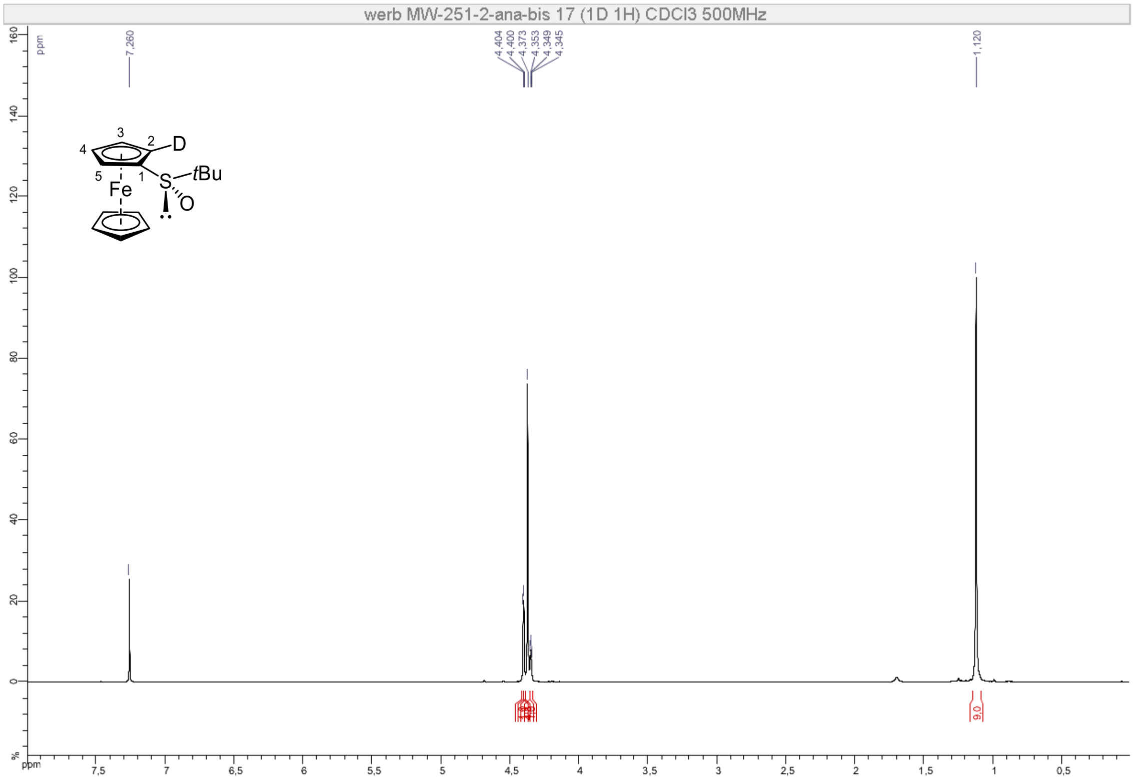

^{13}C NMR (126 MHz, $CDCl_3$)

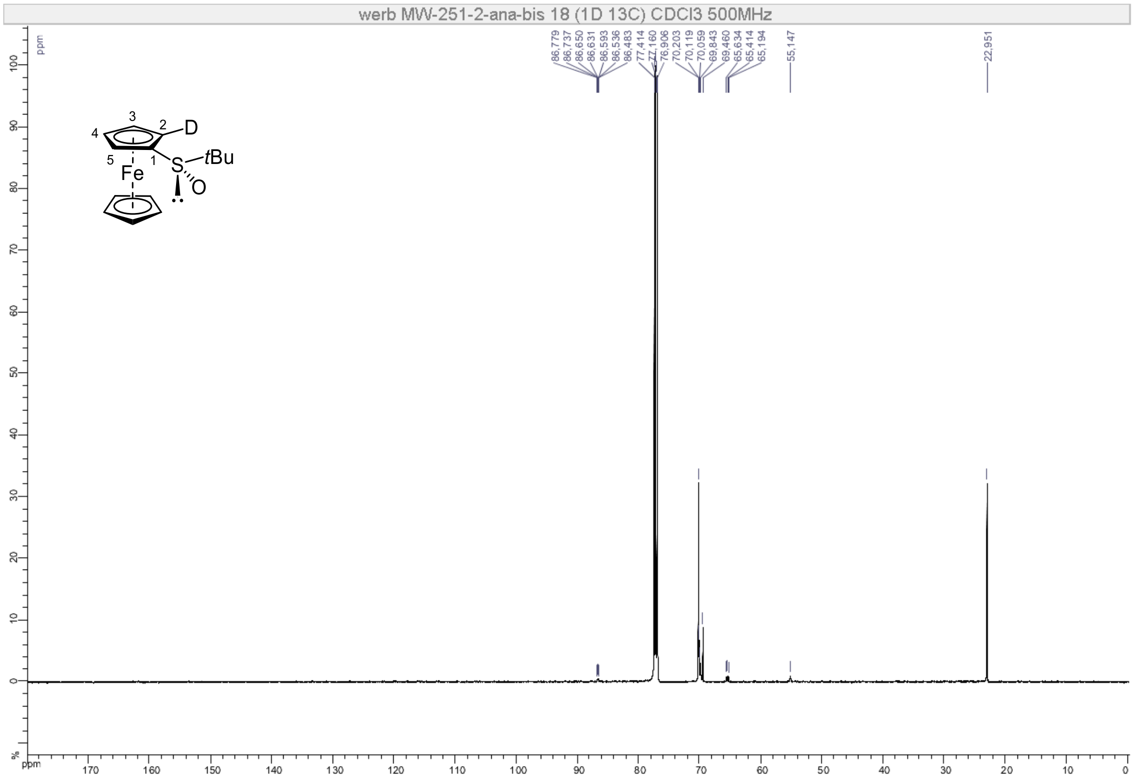

DEPT 135 (126 MHz, $CDCl_3$)

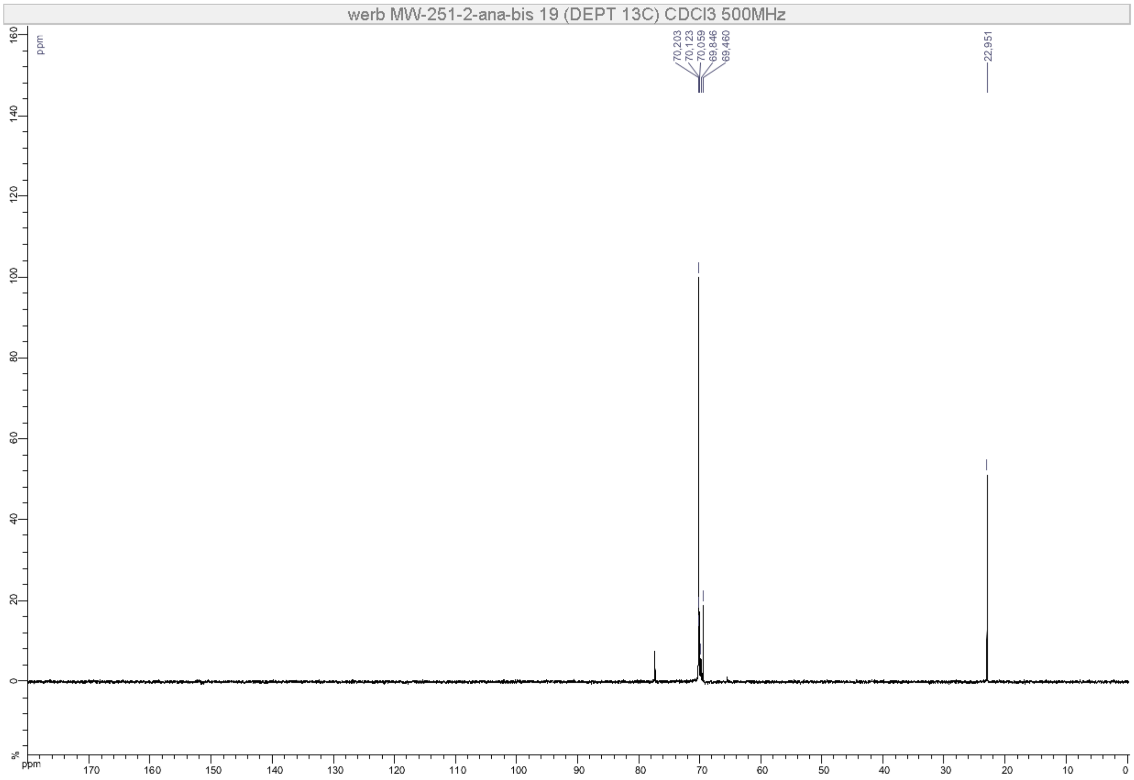

COSY (500 MHz, $CDCl_3$)

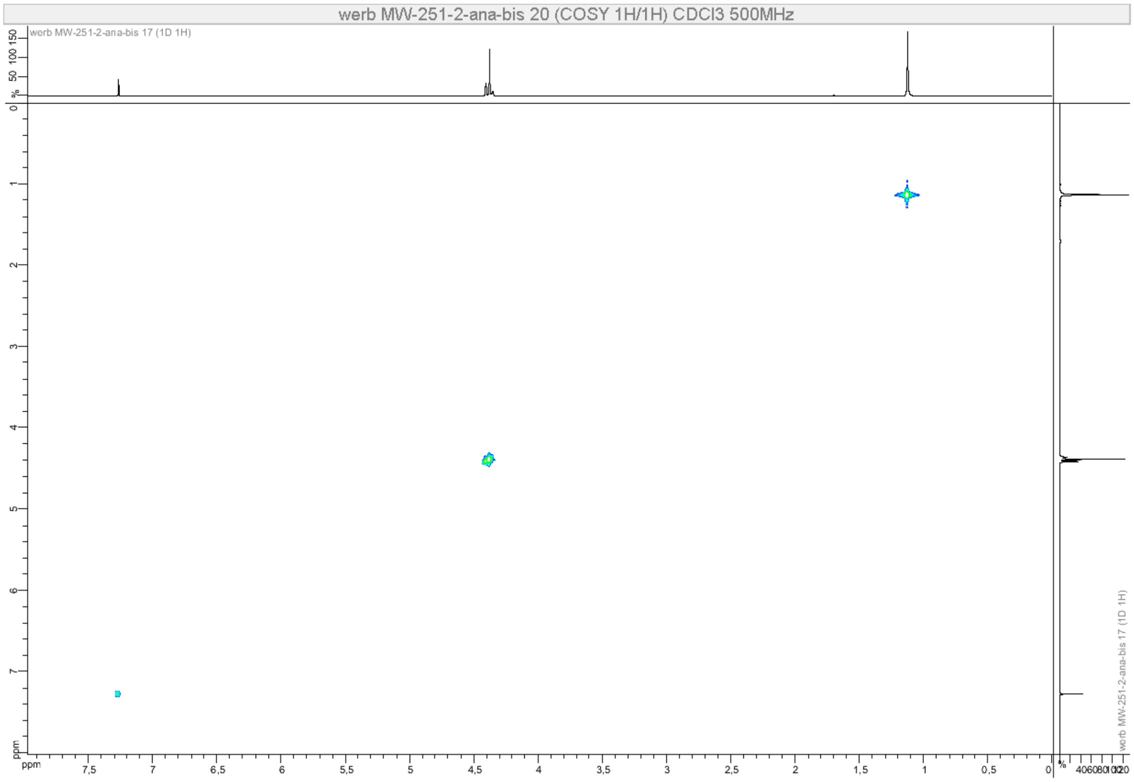

HSQC (500 MHz, $CDCl_3$)

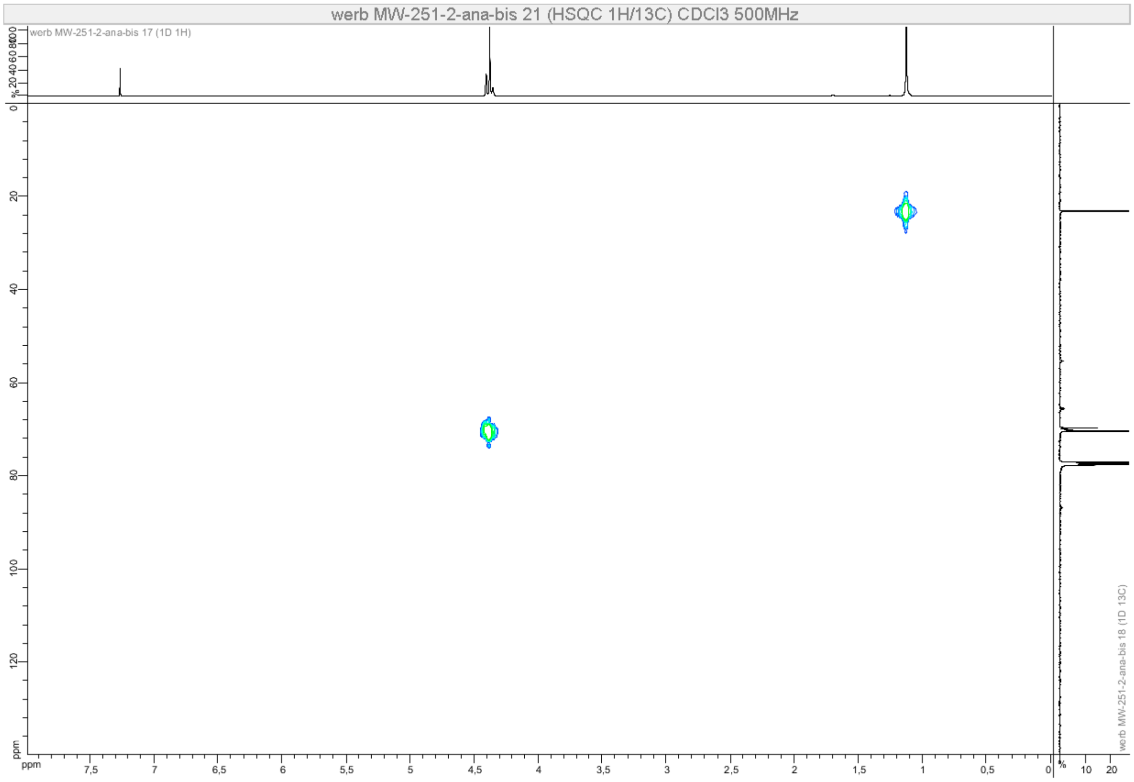

HMBC (500 MHz, $CDCl_3$)

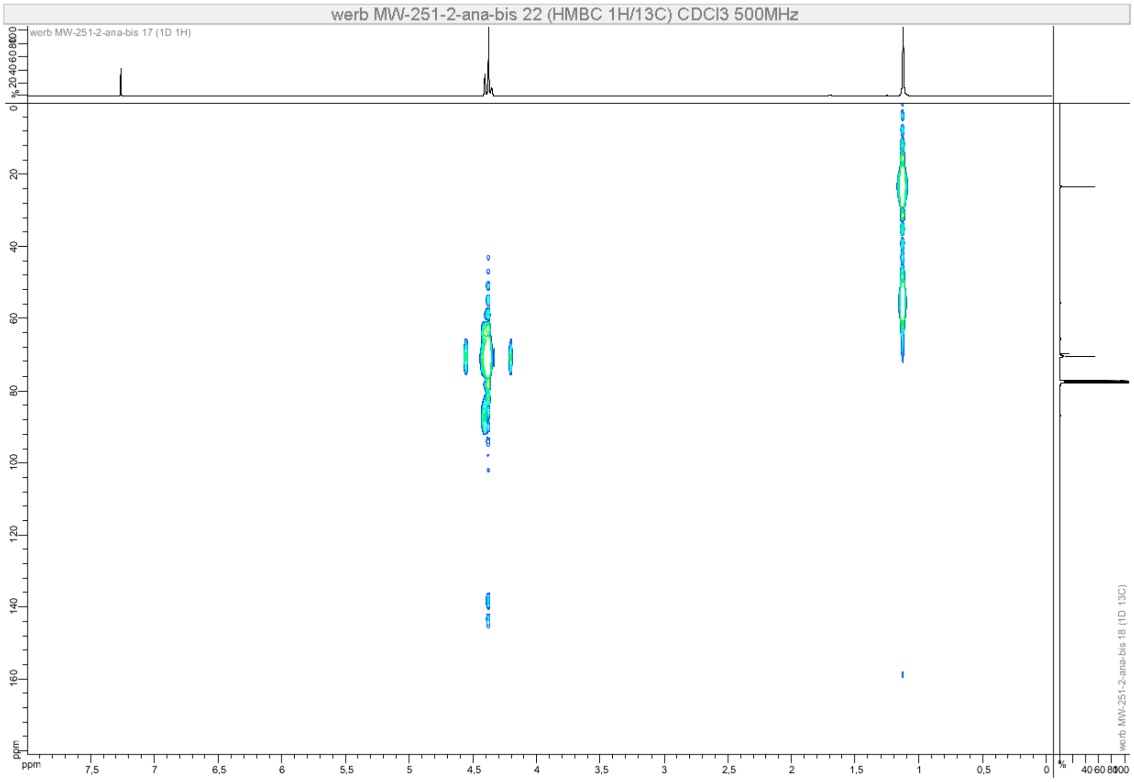

NOESY (500 MHz, $CDCl_3$)

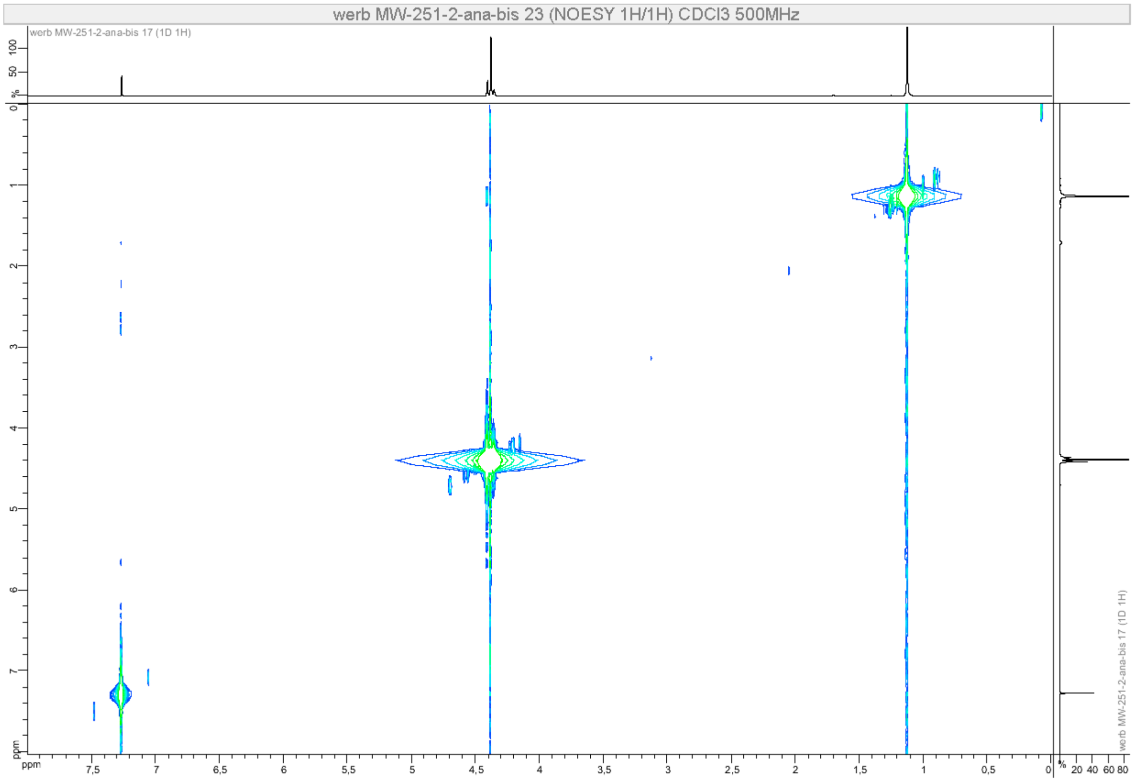

(*S*,*S*P)-*S*-*tert*-Butyl-2-methylferrocenesulfoxide (*S*,*S*P-1d)

^{1}H NMR (500 MHz, $CDCl_3$)

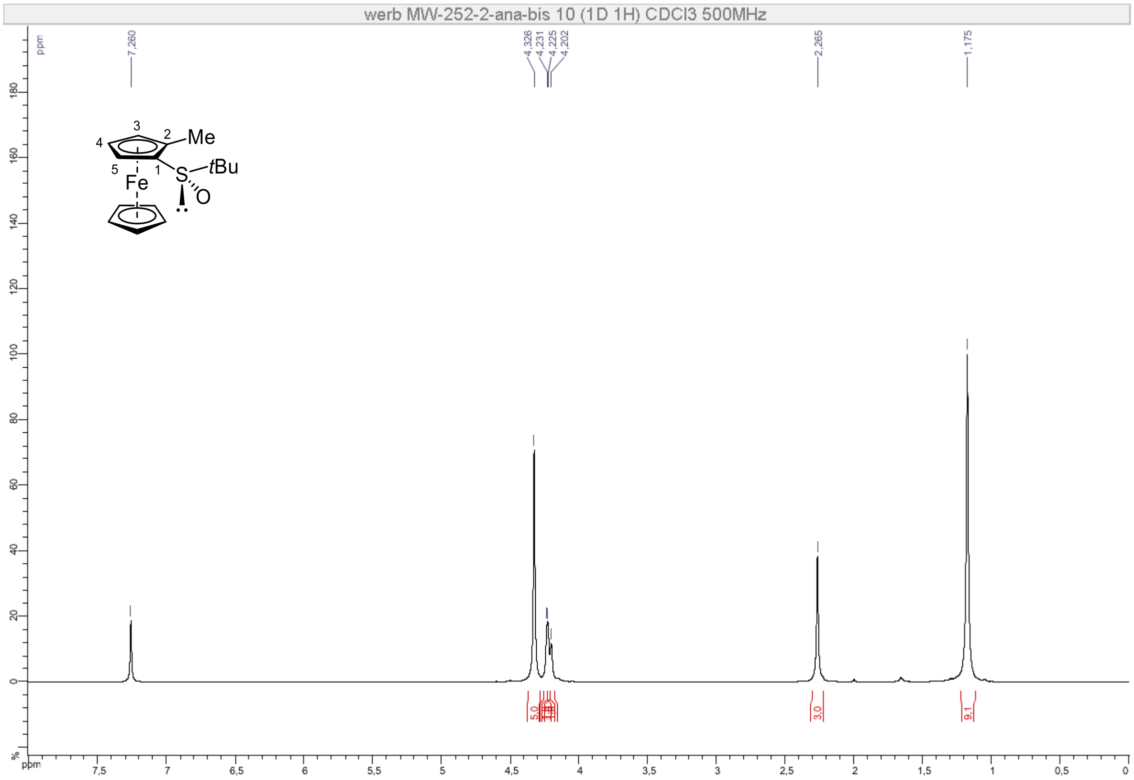

^{13}C NMR (126 MHz, $CDCl_3$)

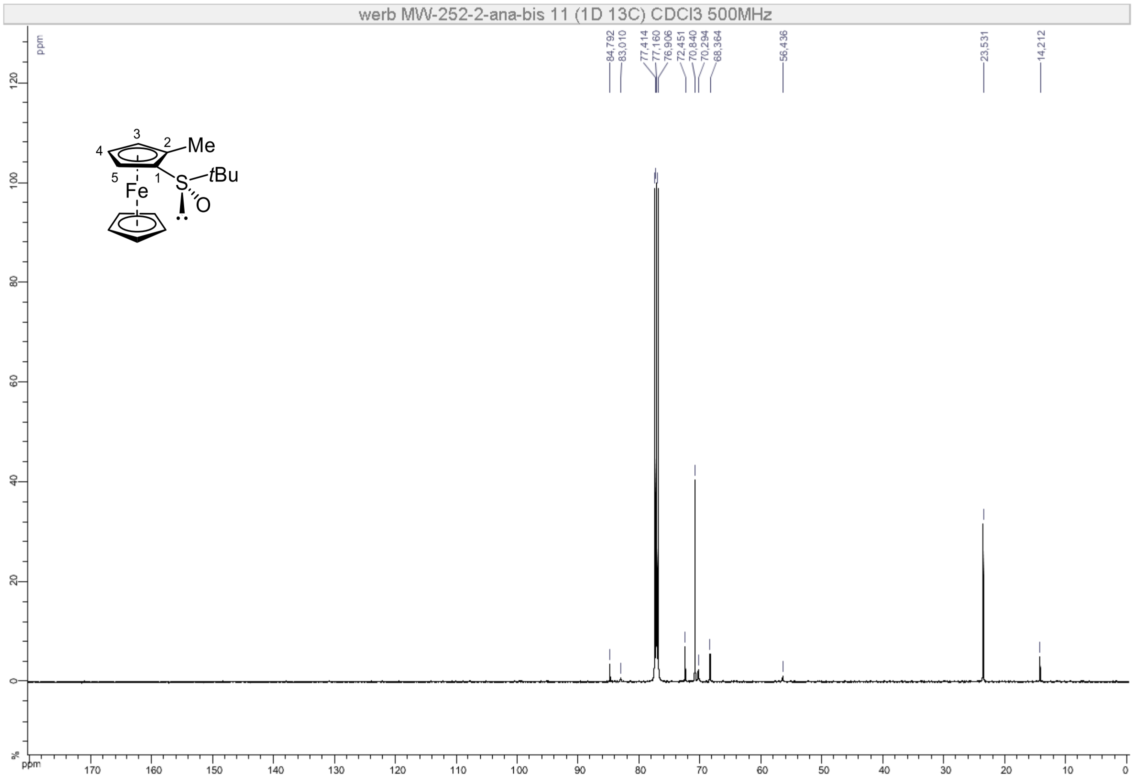

DEPT 135 (126 MHz, $CDCl_3$)

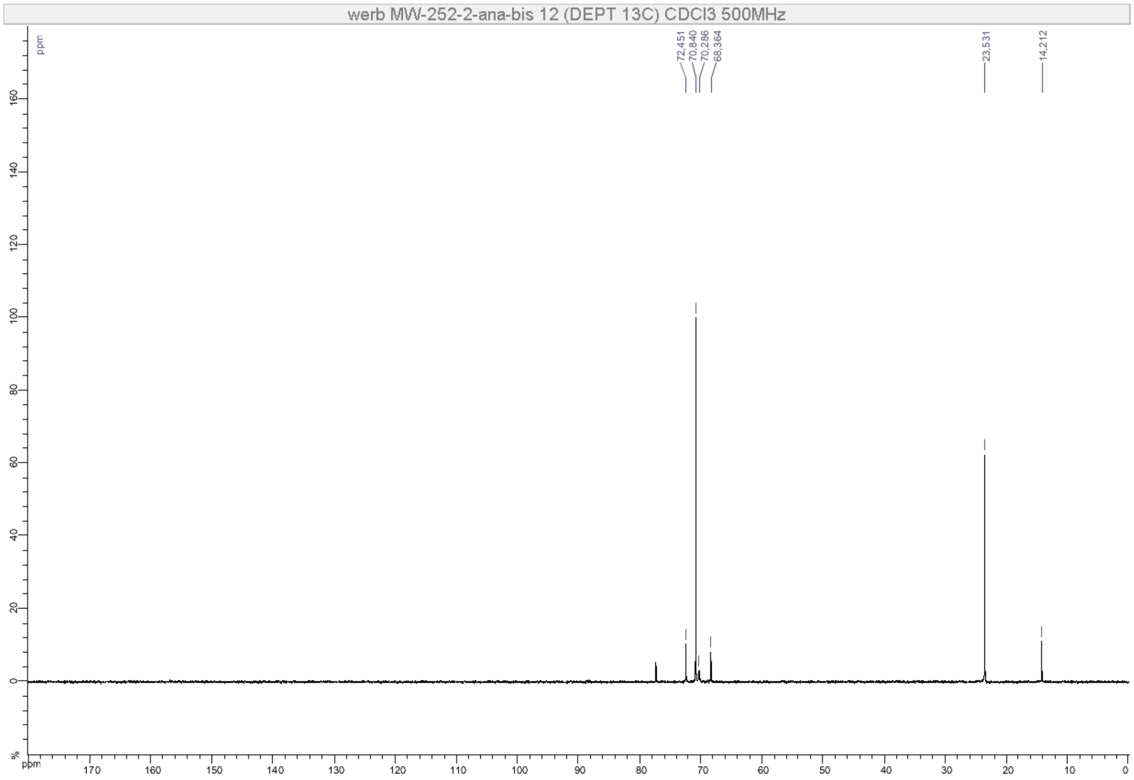

COSY (500 MHz, $CDCl_3$)

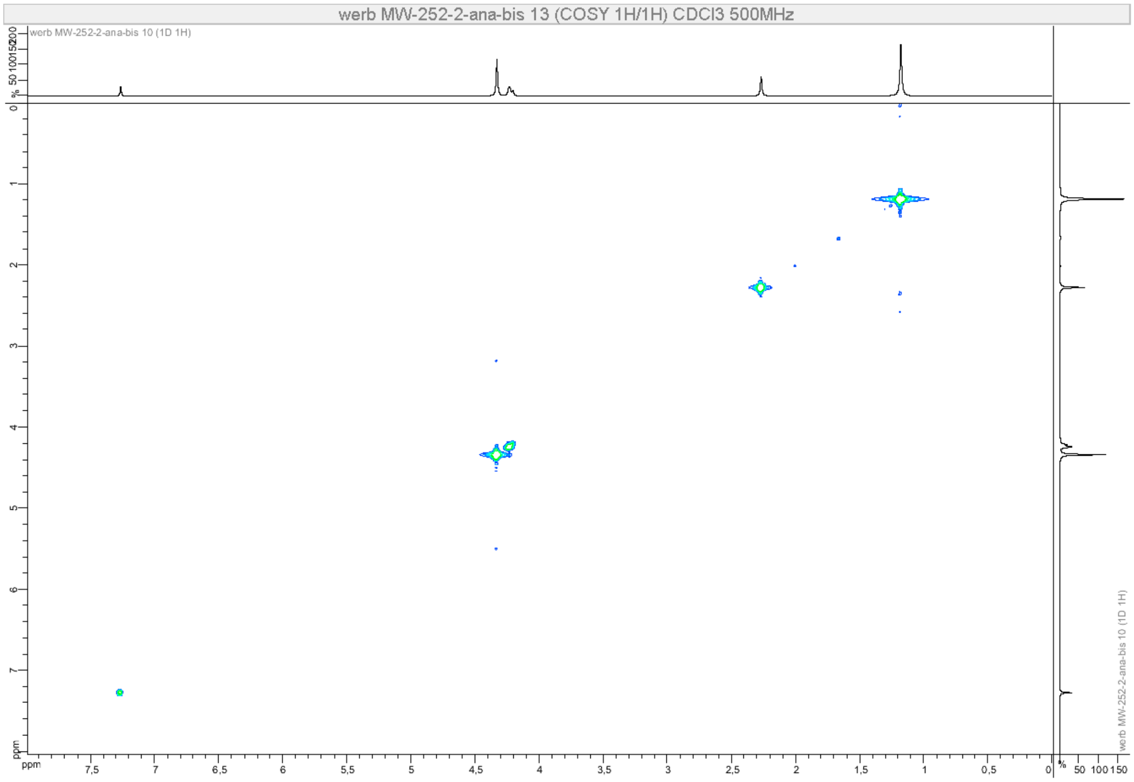

HSQC (500 MHz, $CDCl_3$)

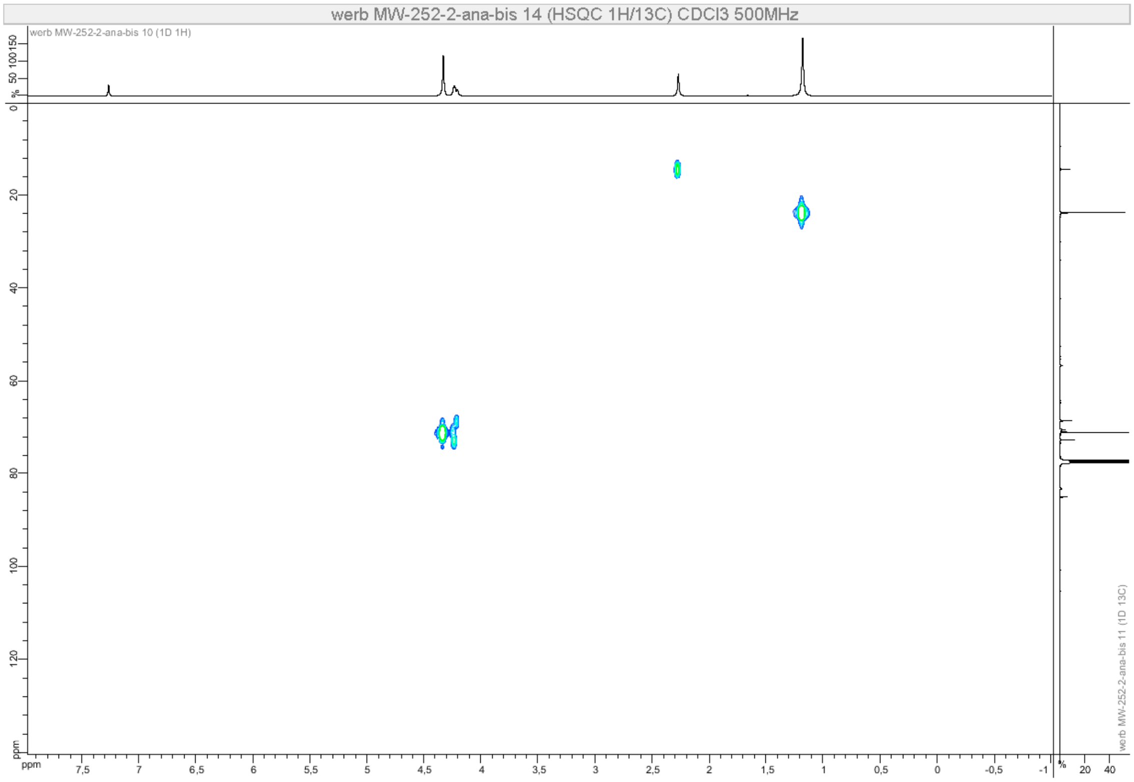

HMBC (500 MHz, $CDCl_3$)

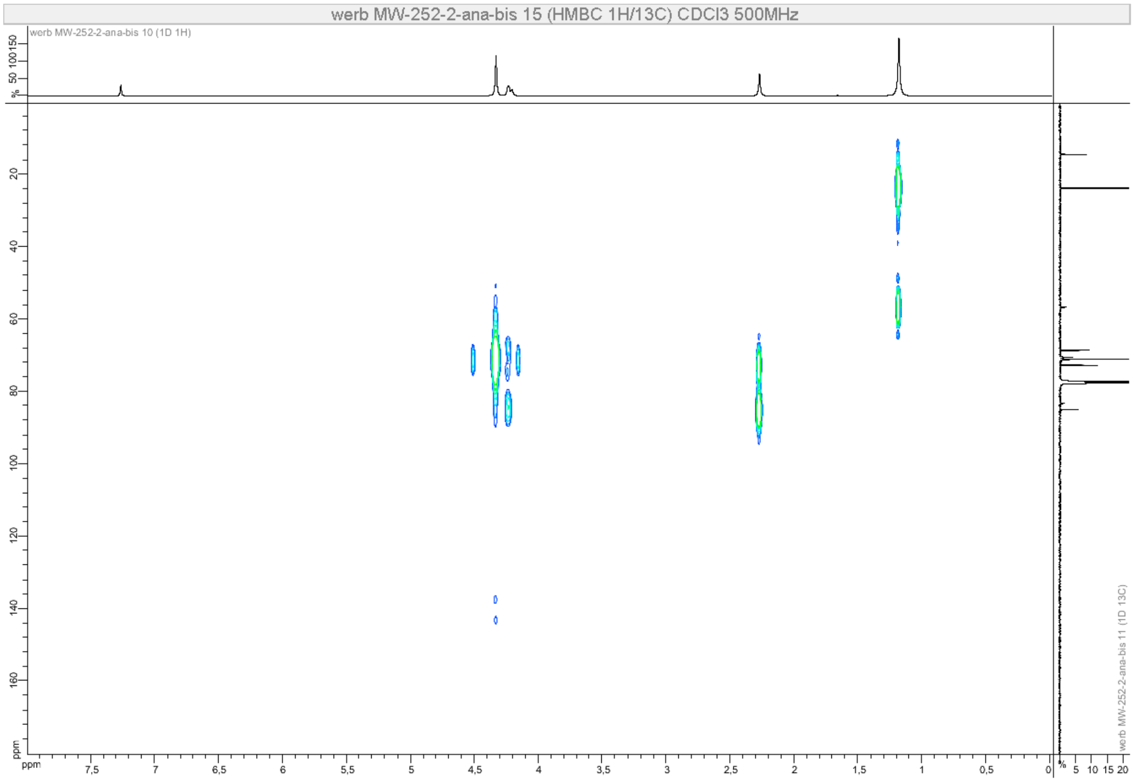

NOESY (500 MHz, $CDCl_3$)

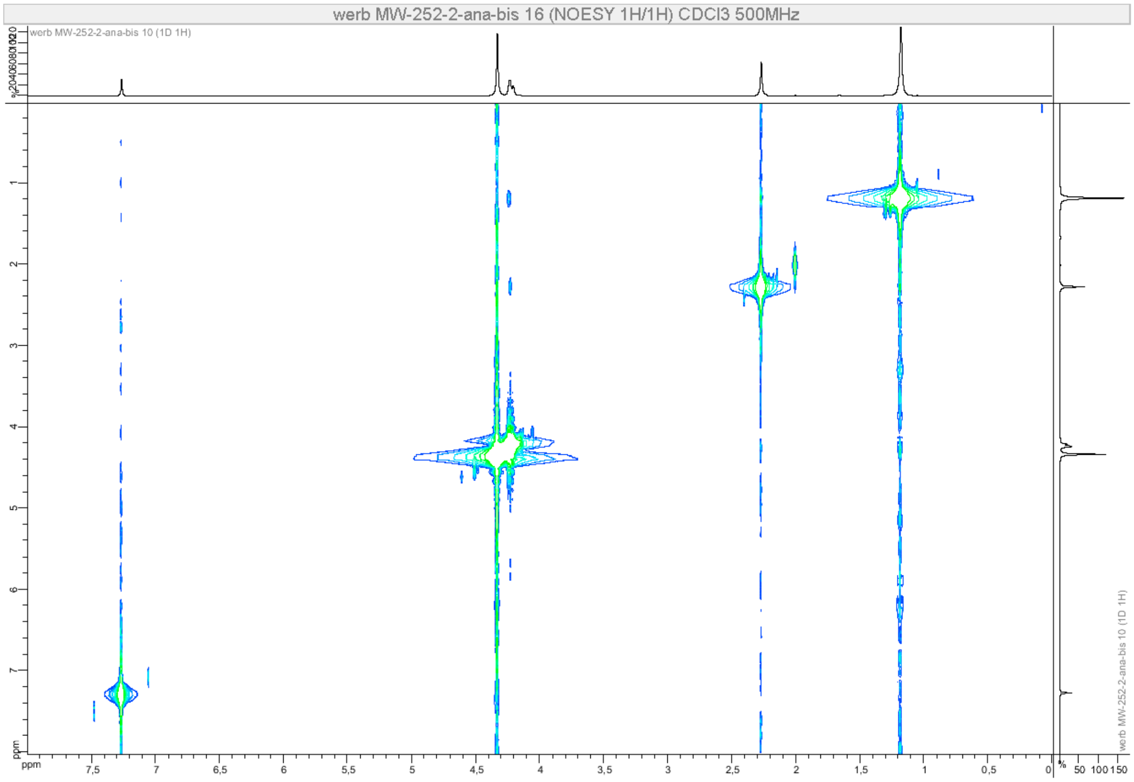

(*S*)-*S*-*tert*-Butyl-2,5-bis(trimethylsilyl)ferrocenesulfoxide (*S*-2aa)

^{1}H NMR (300 MHz, $CDCl_3$)

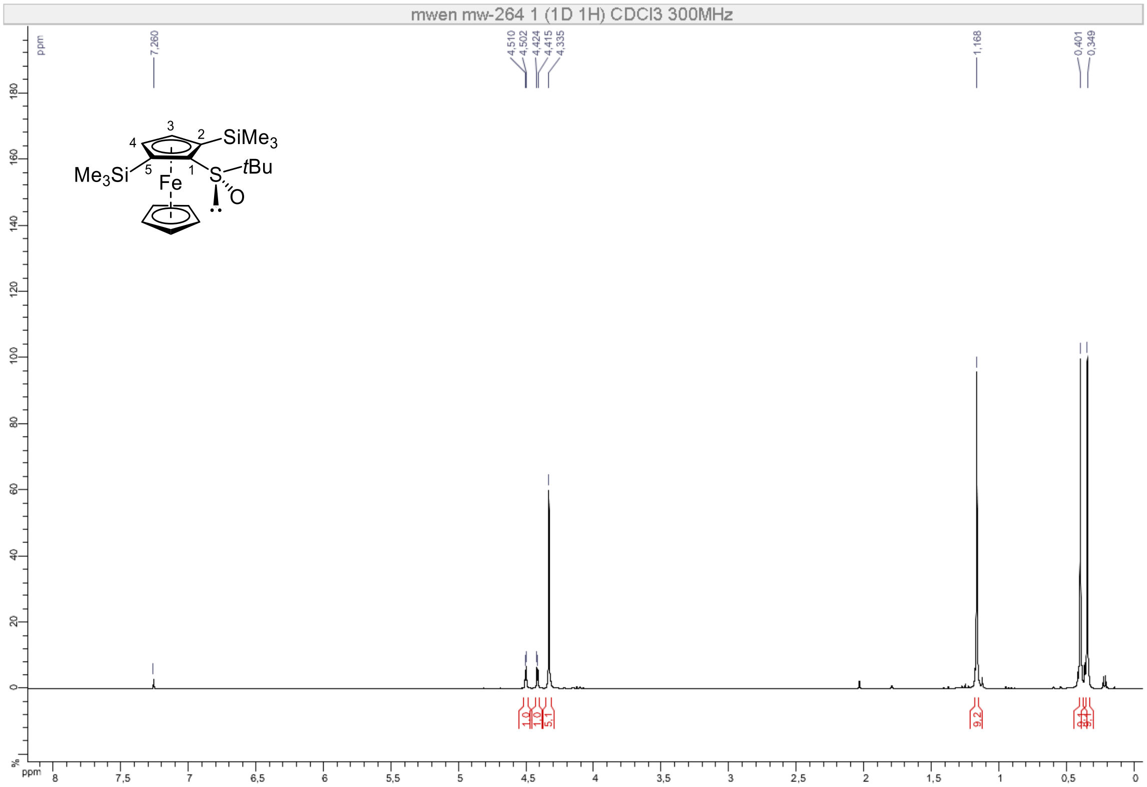

(*R*)- and (*S*)-*S*-*tert*-Butyl-2,5-bis(trimethylsilyl)ferrocenesulfoxide (*rac*-2aa)

^{1}H NMR (500 MHz, $CDCl_3$)

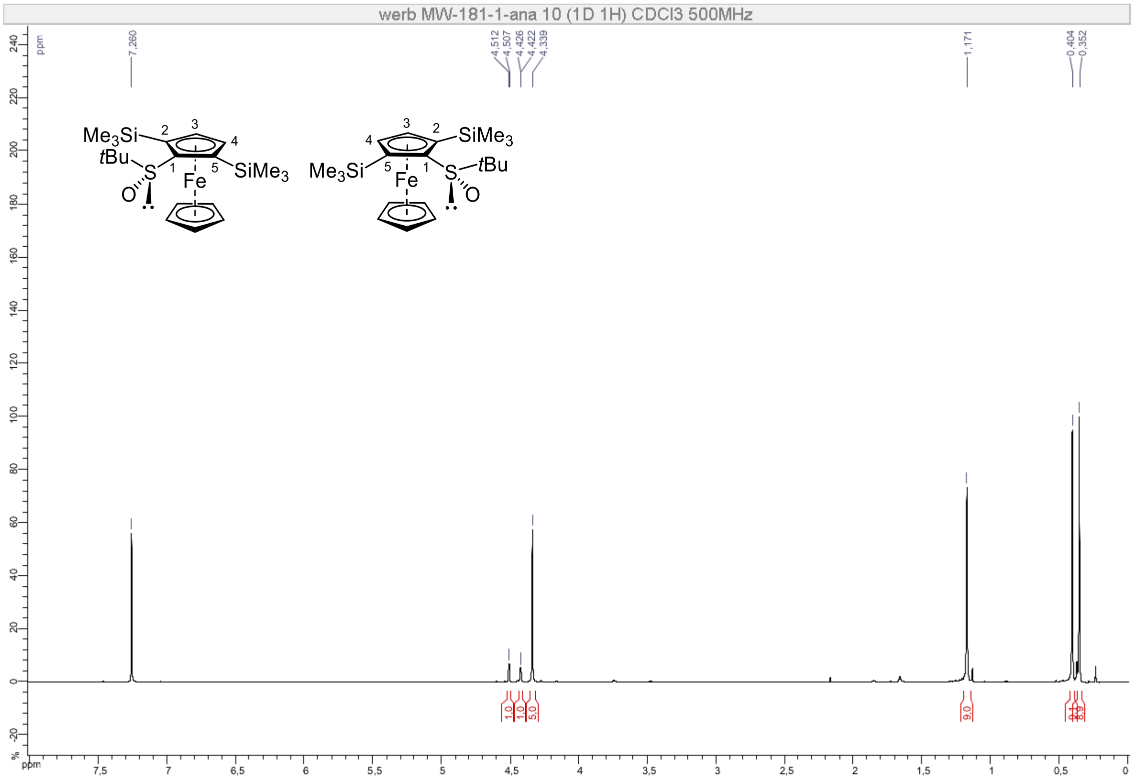

^{13}C NMR (126 MHz, $CDCl_3$)

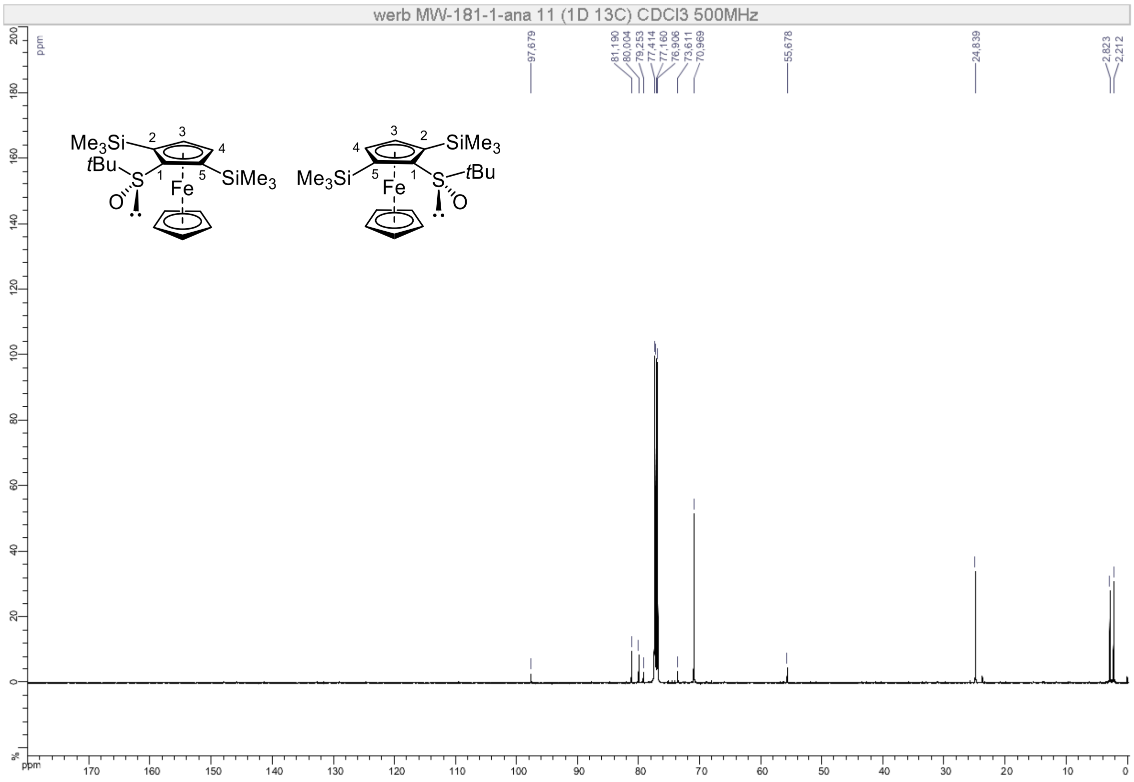

COSY (500 MHz, $CDCl_3$)

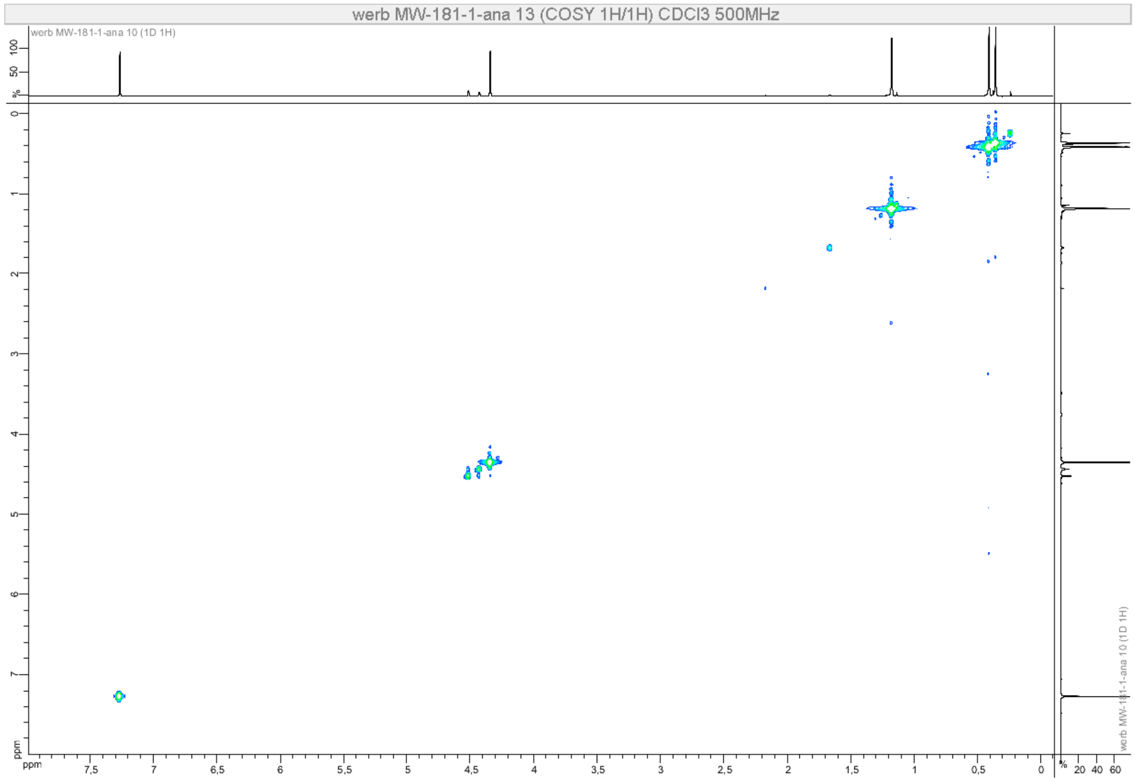

HSQC (500 MHz, $CDCl_3$)

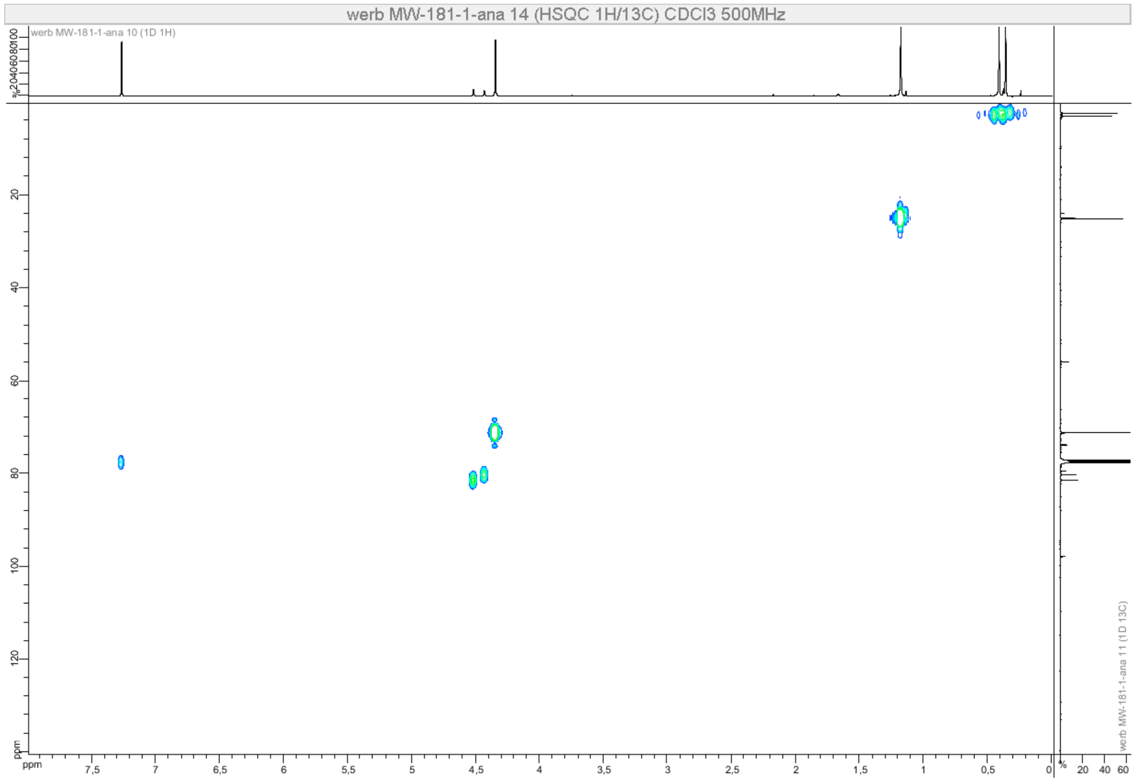

HMBC (500 MHz, $CDCl_3$)

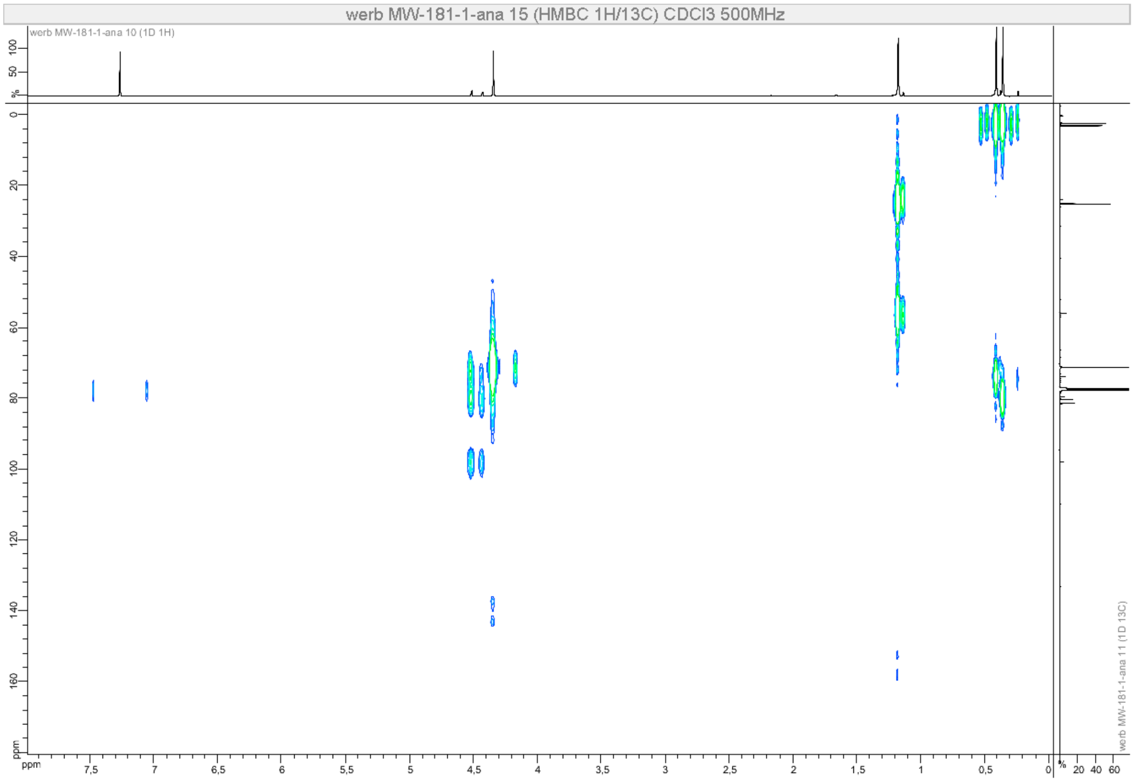

NOESY (500 MHz, $CDCl_3$)

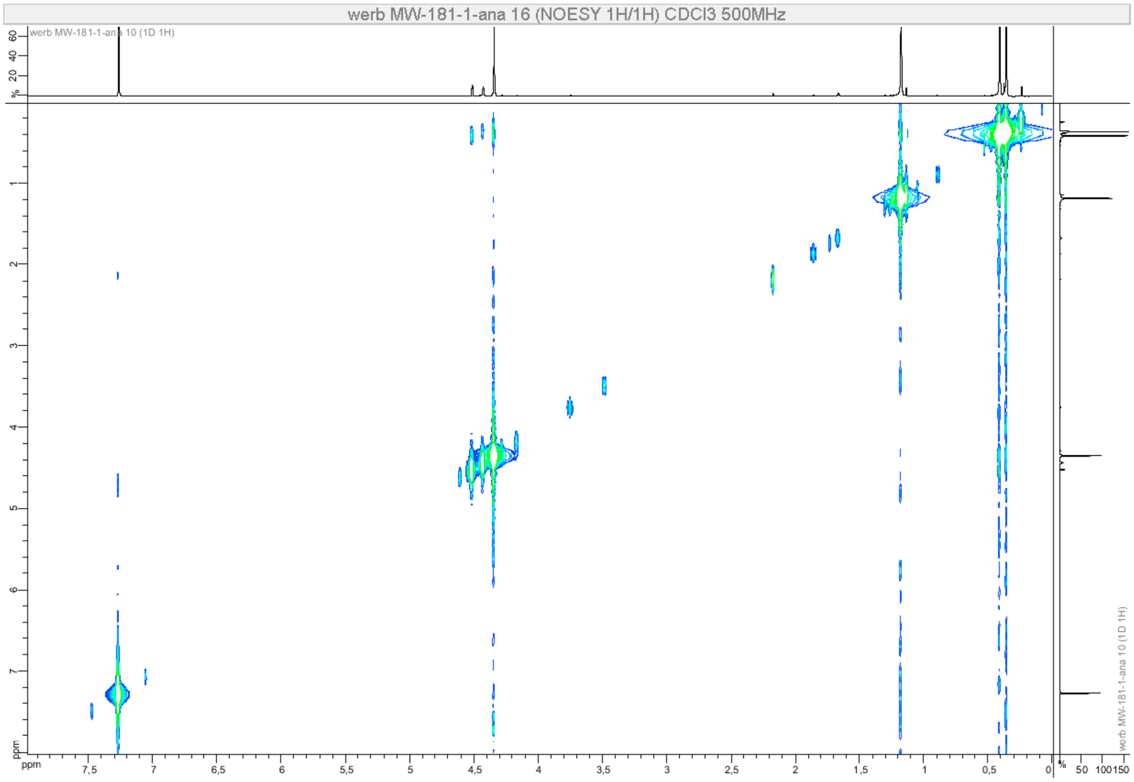

(*S*,*S*P)-*S*-*tert*-Butyl-2-deuterio-5-(trimethylsilyl)ferrocenesulfoxide (*S*,*S*P-2ac)

^{1}H NMR (500 MHz, $CDCl_3$)

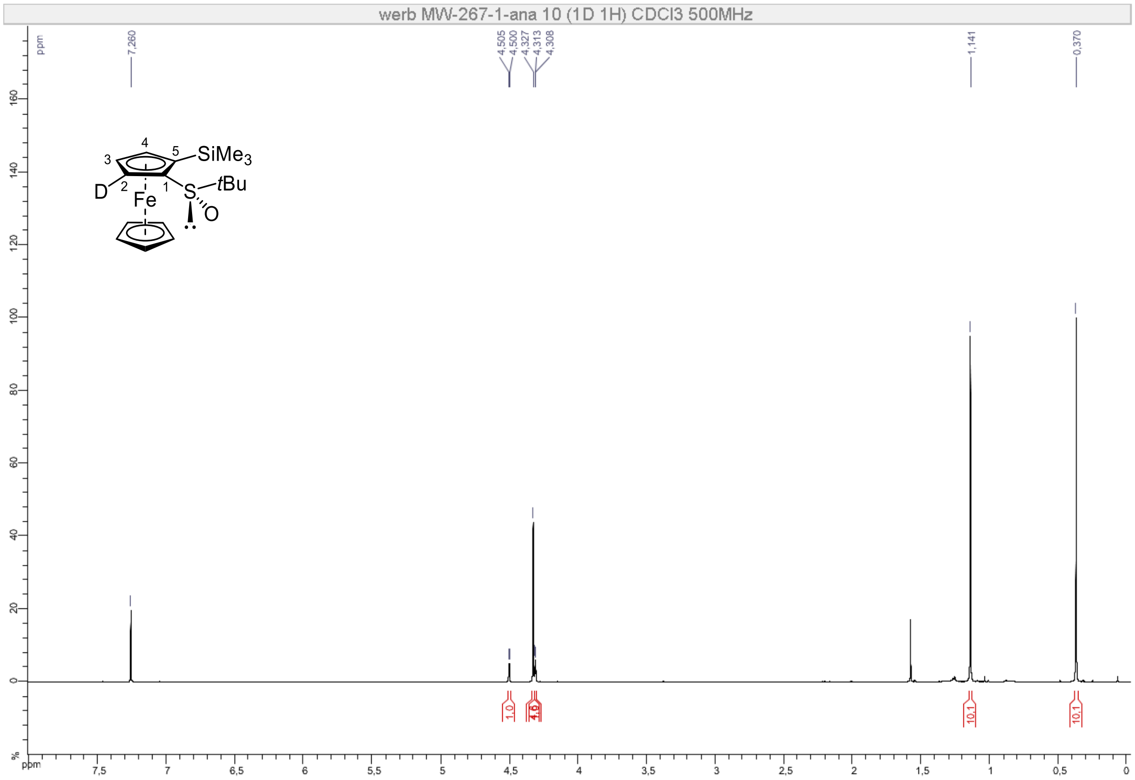

^{13}C NMR (126 MHz, $CDCl_3$)

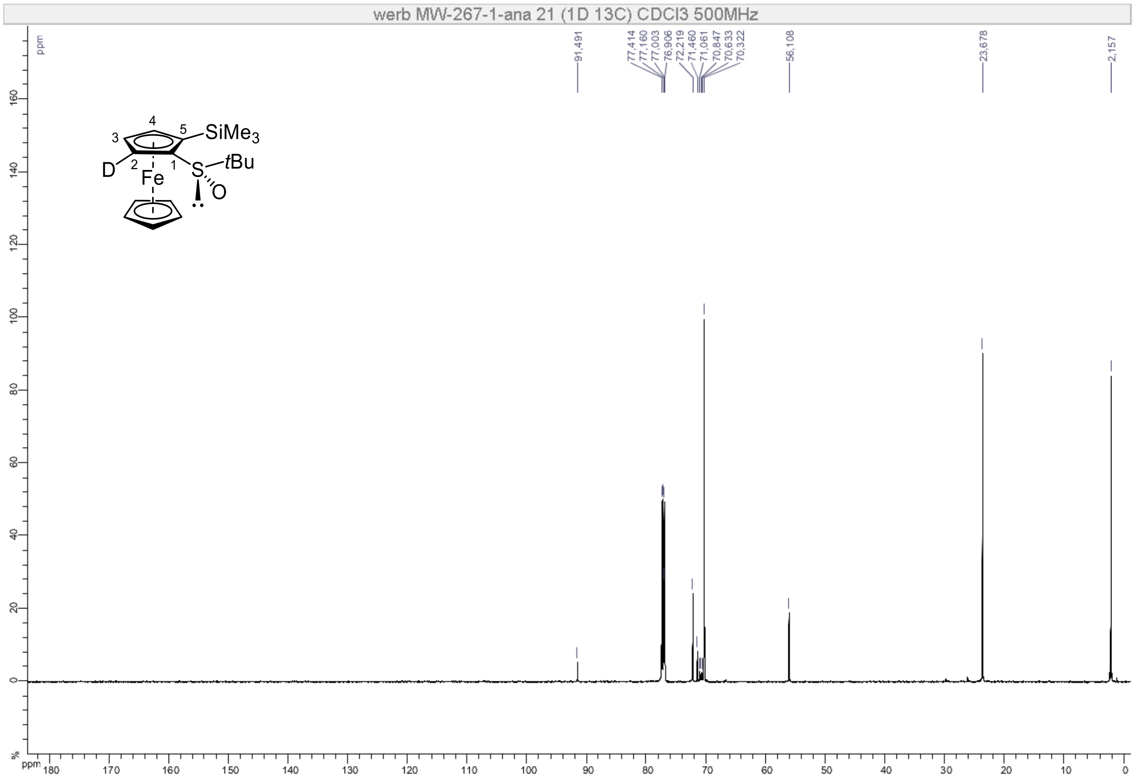

COSY (500 MHz, $CDCl_3$)

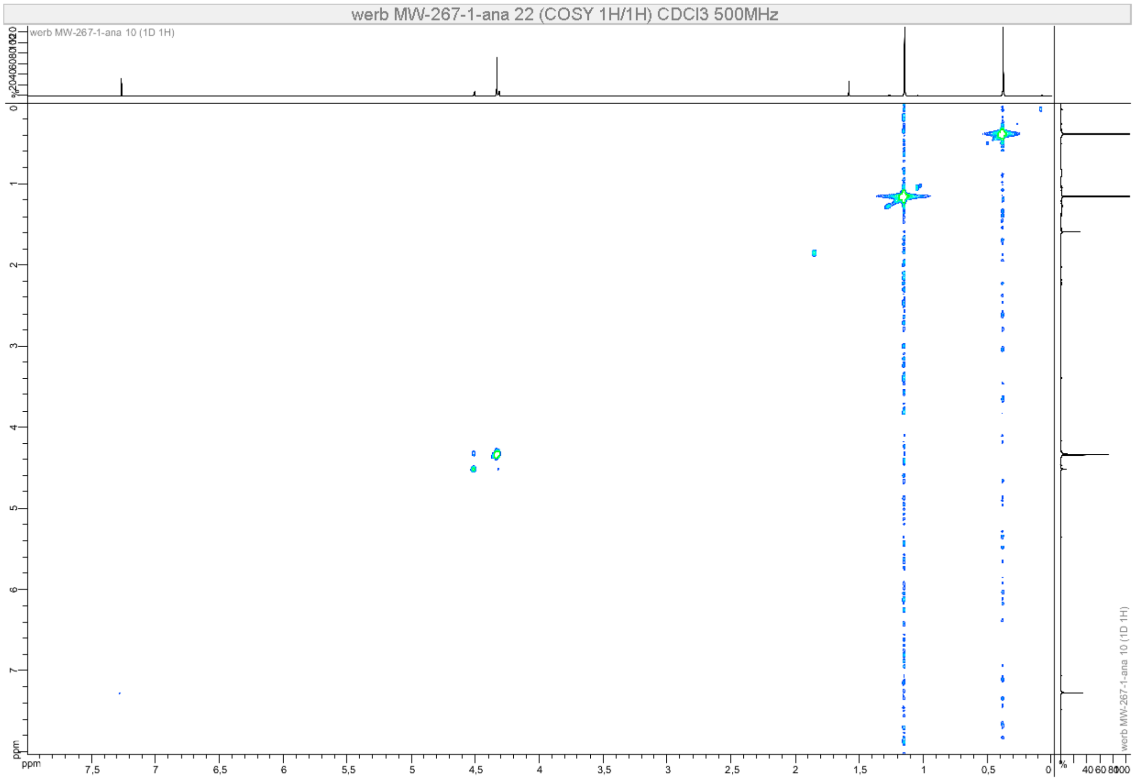

HSQC (500 MHz, $CDCl_3$)

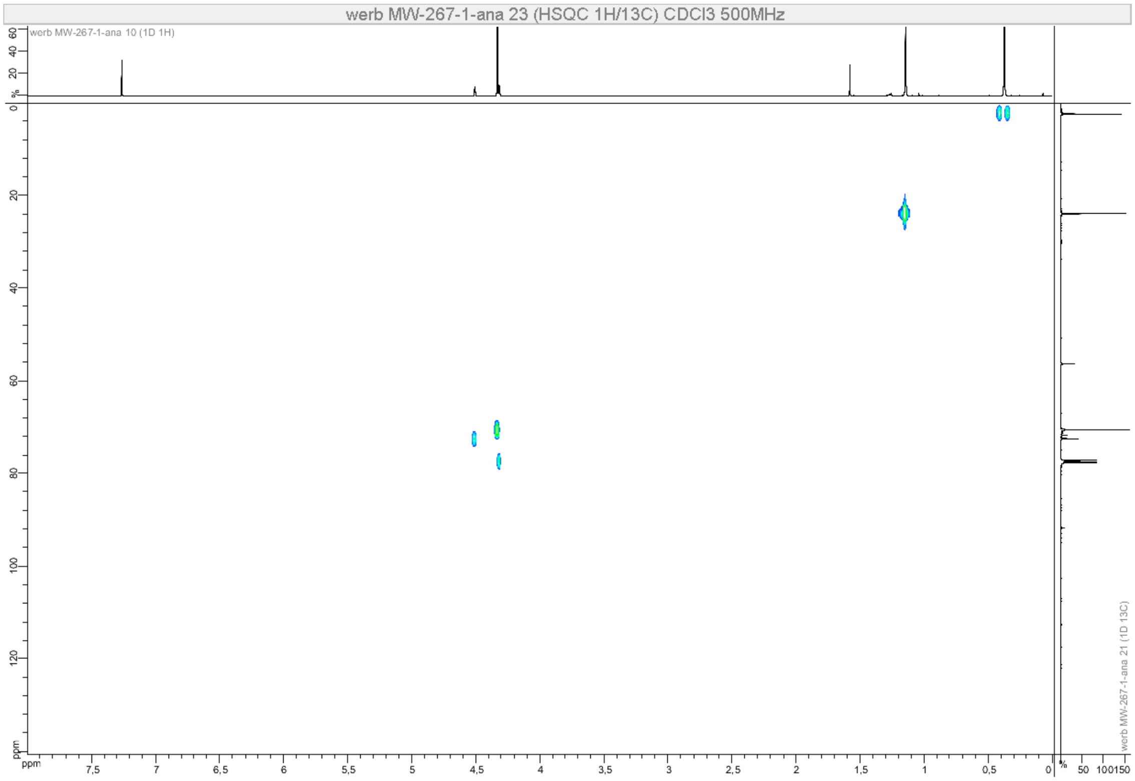

HMBC (500 MHz, $CDCl_3$)

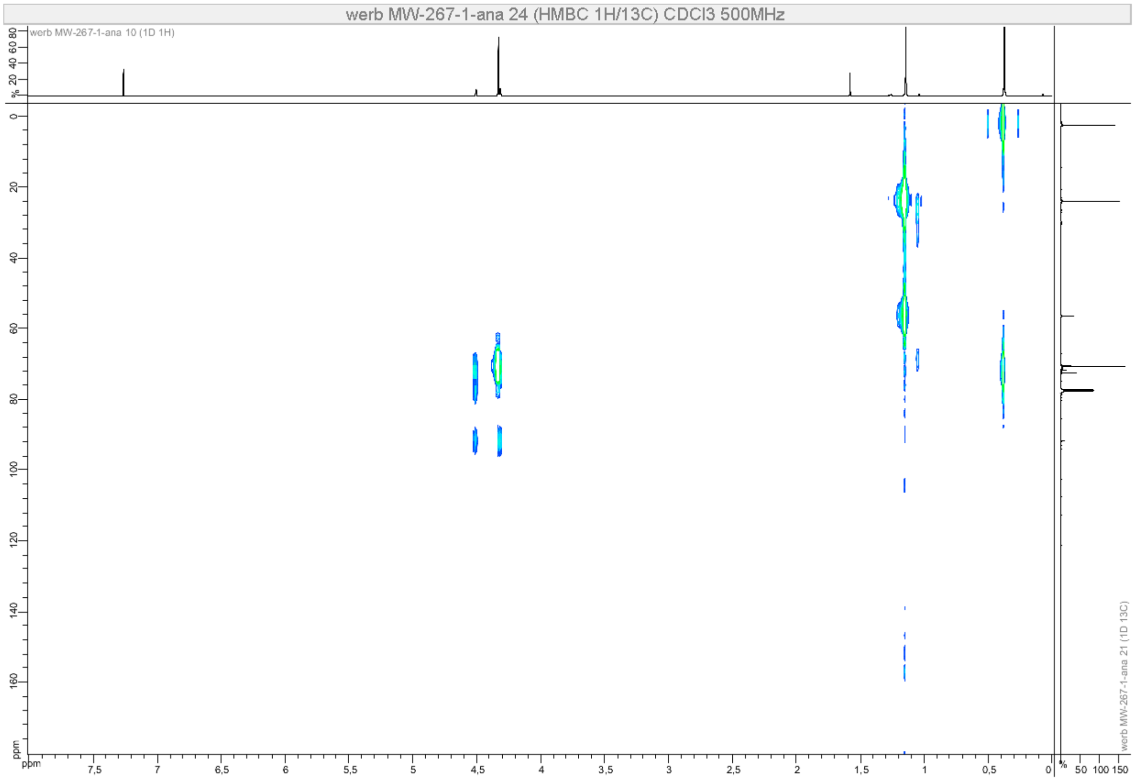

NOESY (500 MHz, $CDCl_3$)

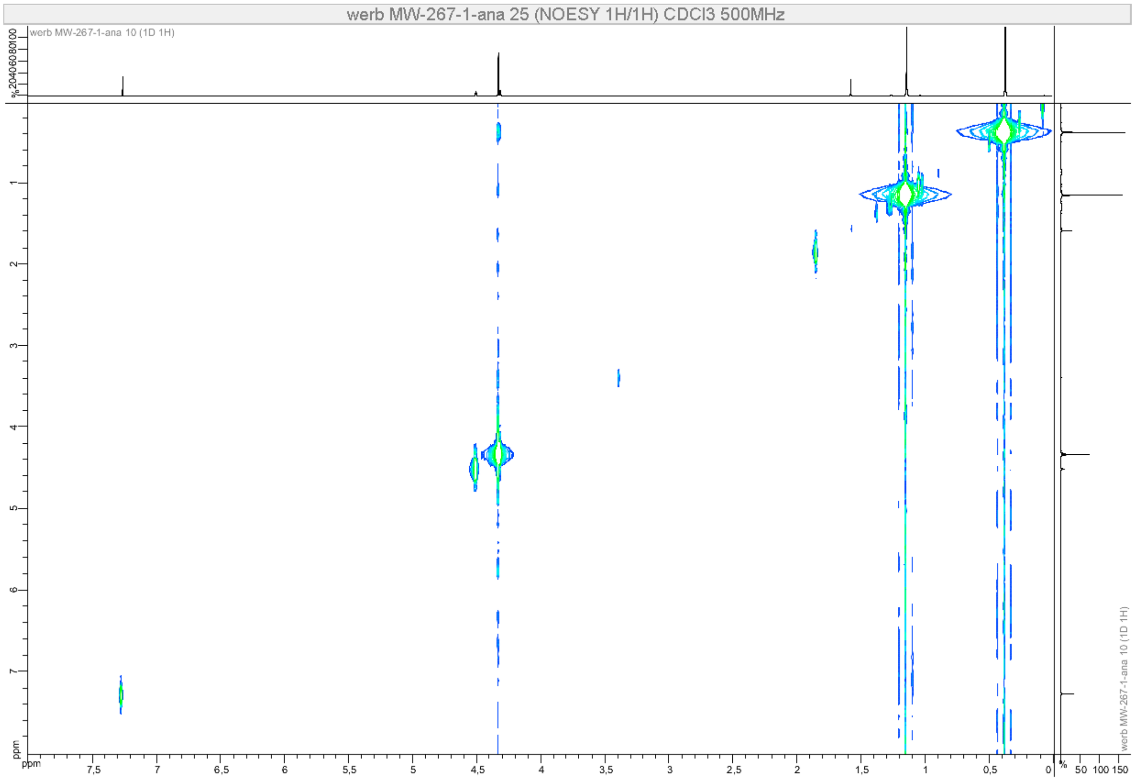

(S,R_P)-*S*-*tert*-Butyl-2-deuterioferrocenesulfoxide (S,R_P-1c)

^{1}H NMR (500 MHz, $CDCl_3$)

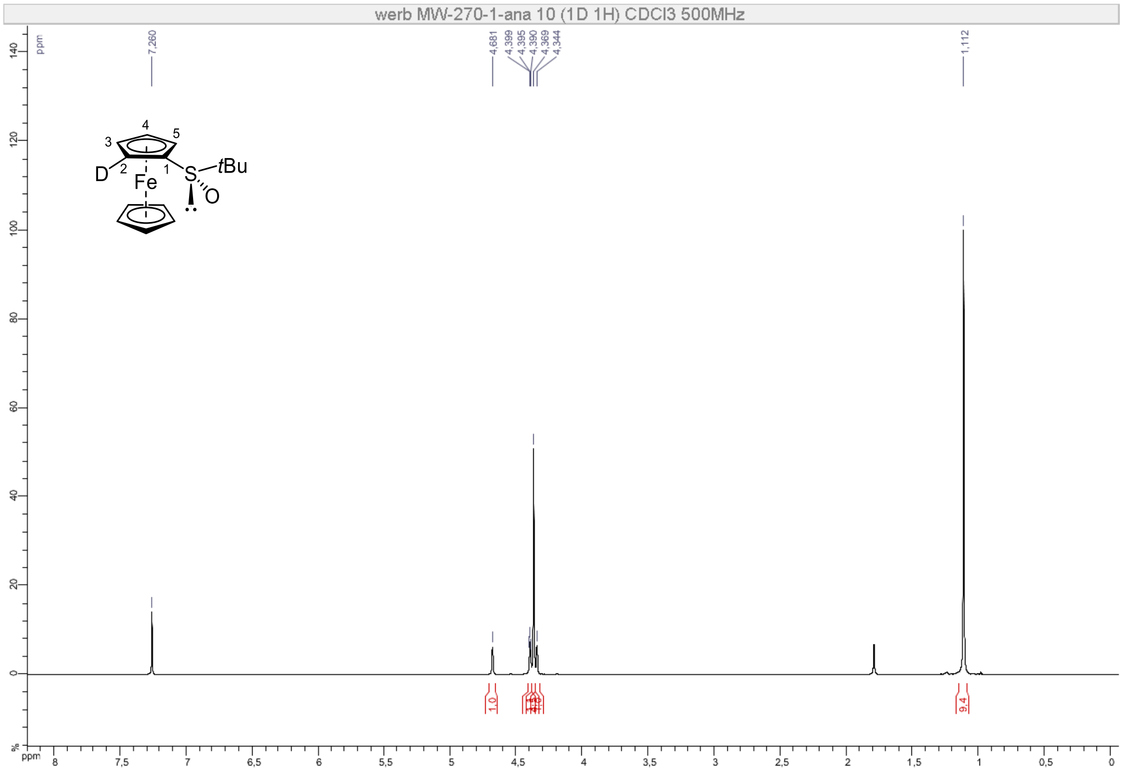

^{13}C NMR (126 MHz, $CDCl_3$)

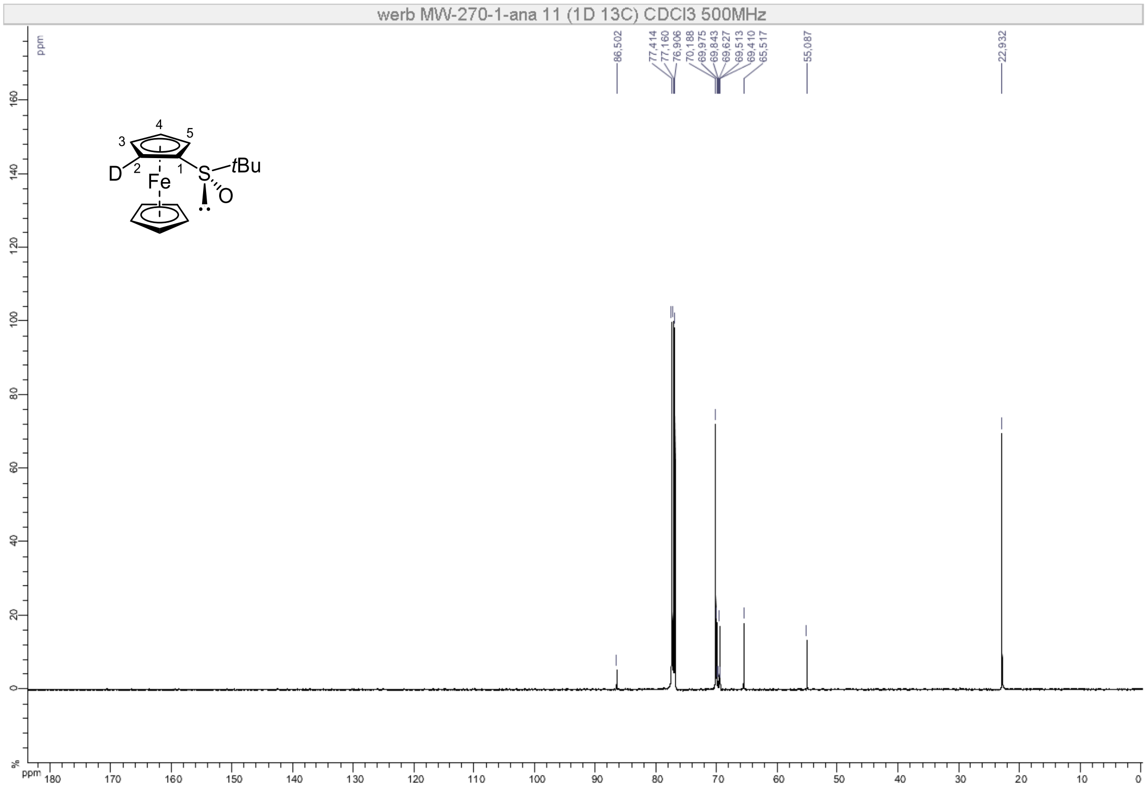

DEPT 135 (126 MHz, $CDCl_3$)

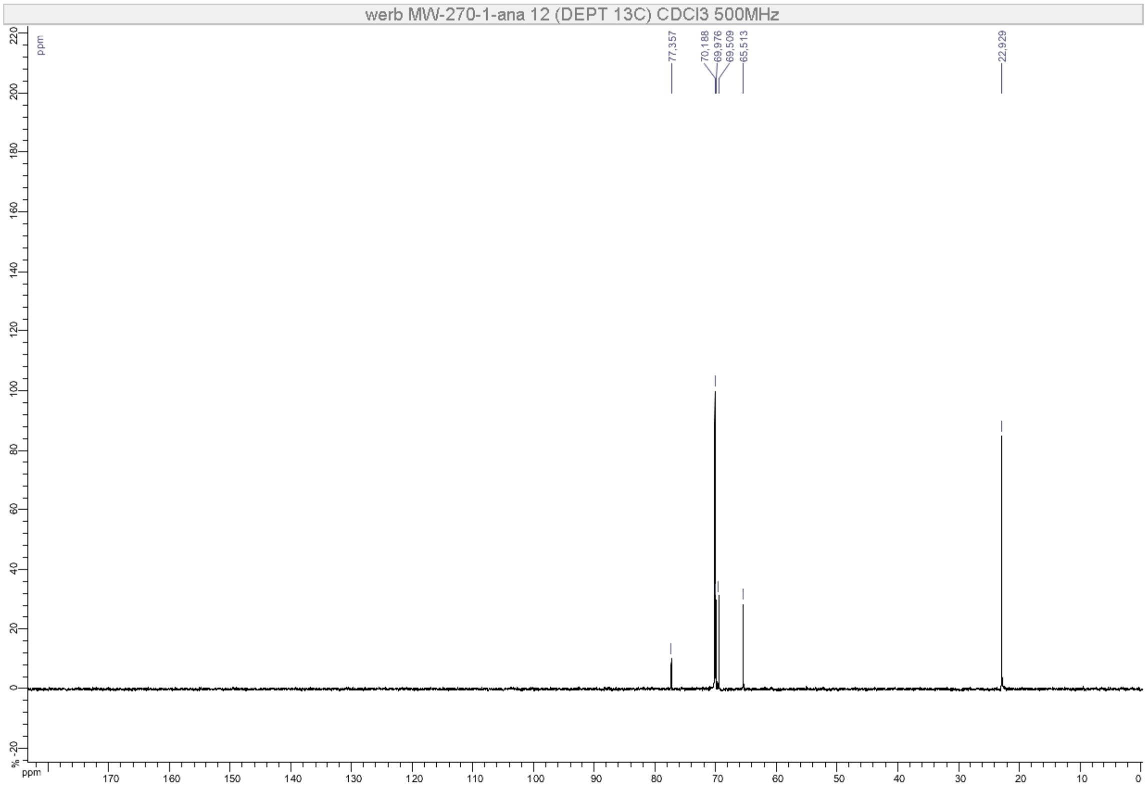

COSY (500 MHz, $CDCl_3$)

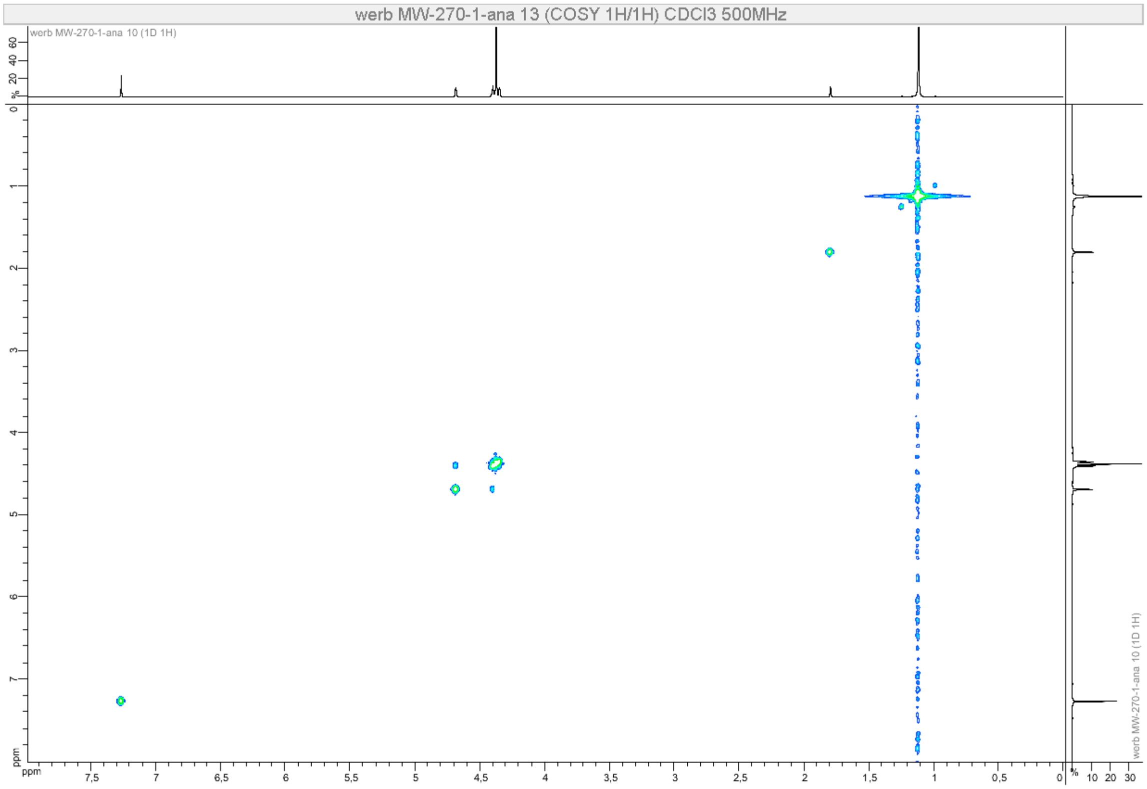

HSQC (500 MHz, $CDCl_3$)

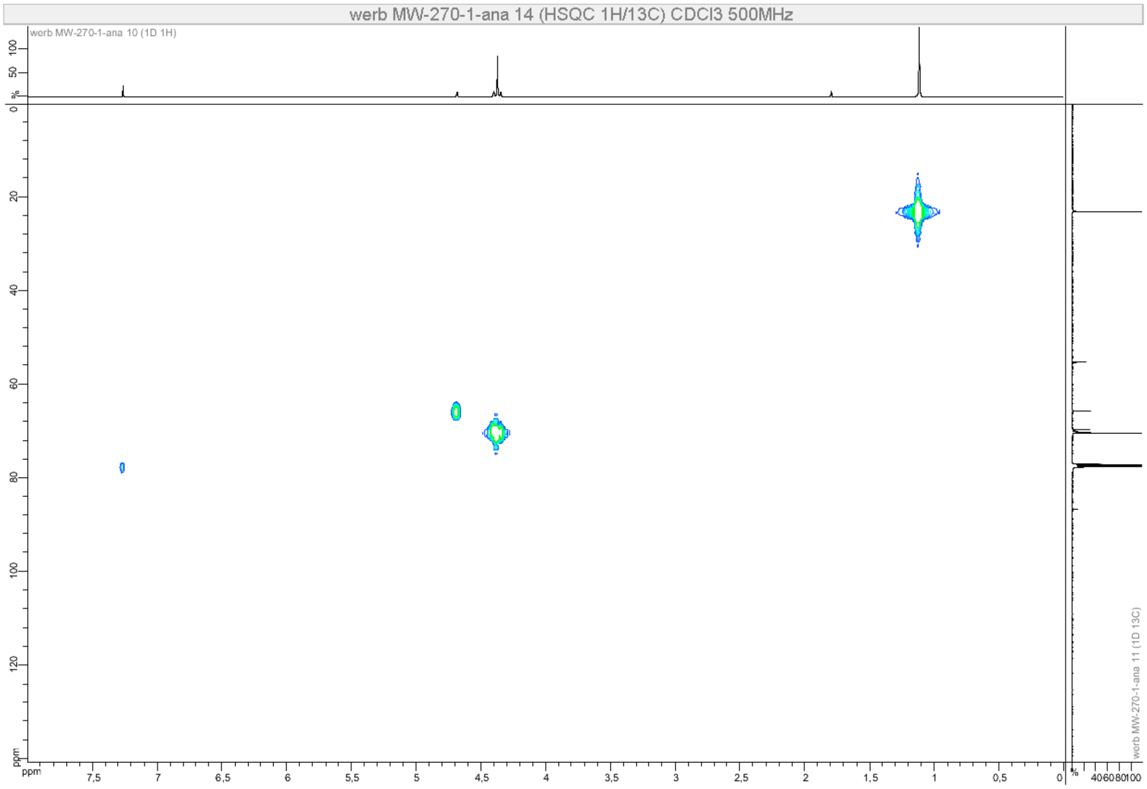

HMBC (500 MHz, $CDCl_3$)

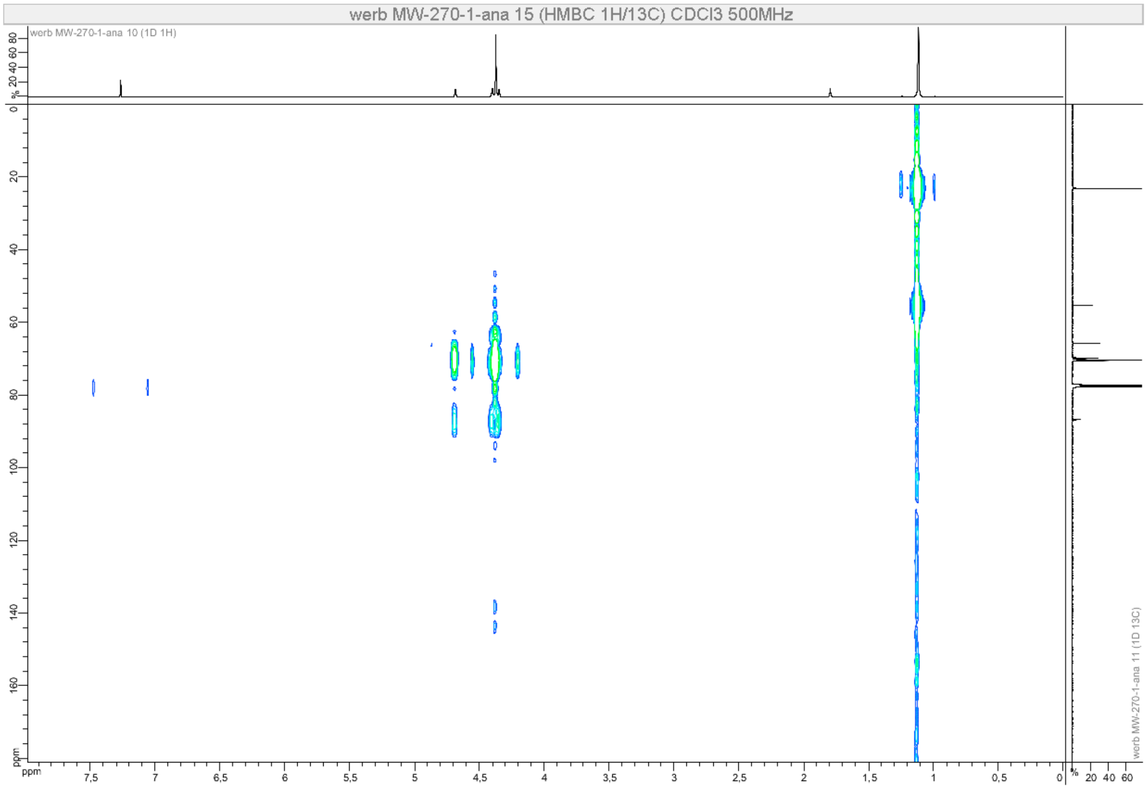

NOESY (500 MHz, $CDCl_3$)

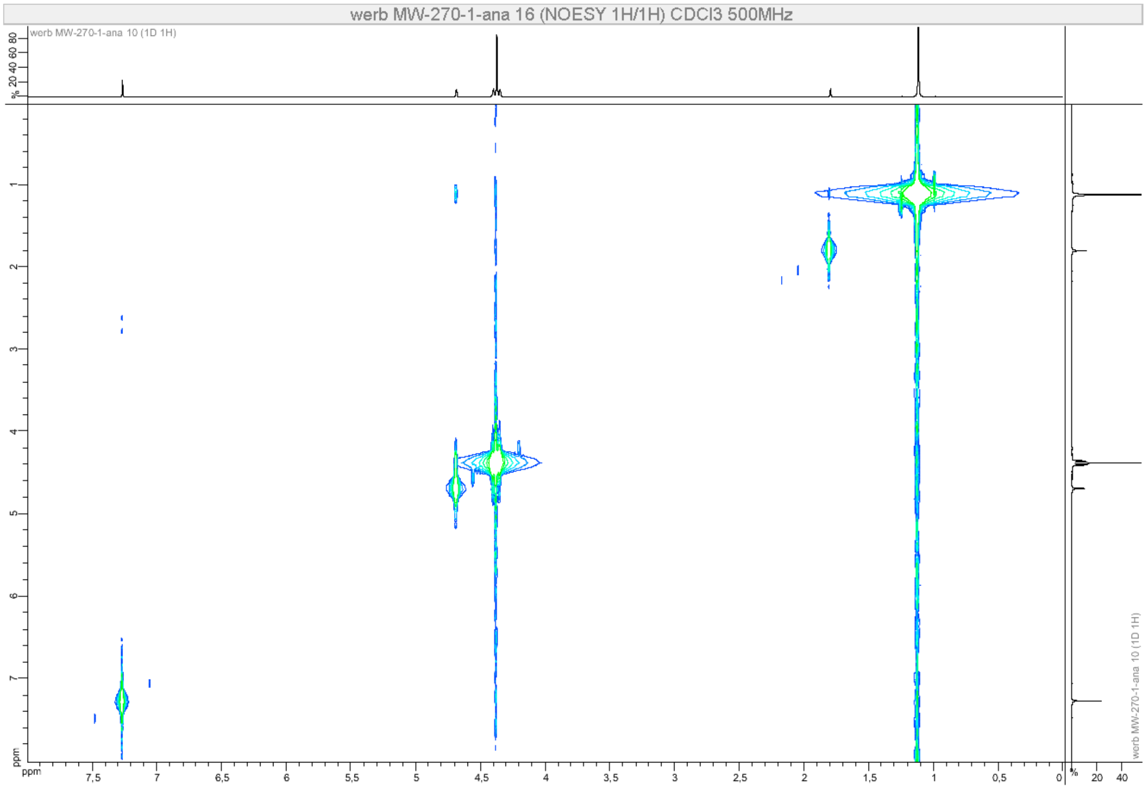

(*S*,*S*P)-*S*-*tert*-Butyl-2-iodo-5-(trimethylsilyl)ferrocenesulfoxide (*S*,*S*P-2ab)

^{1}H NMR (500 MHz, $CDCl_3$)

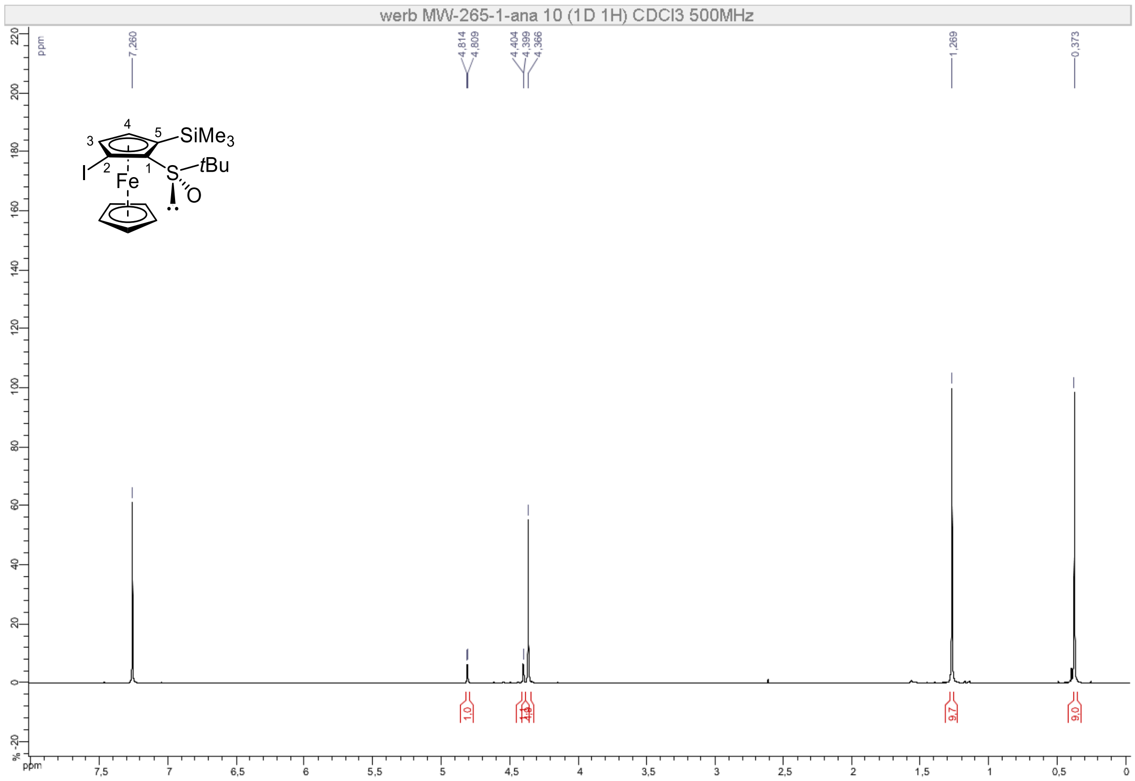

^{13}C NMR (126 MHz, $CDCl_3$)

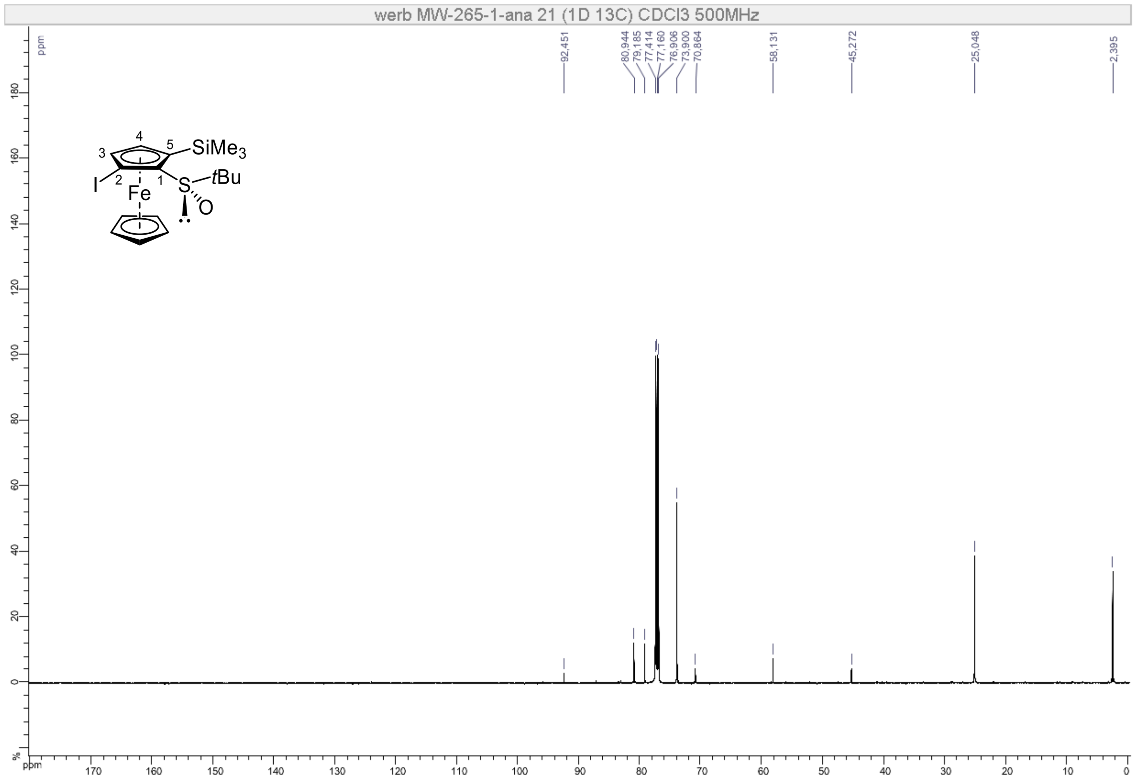

COSY (500 MHz, $CDCl_3$)

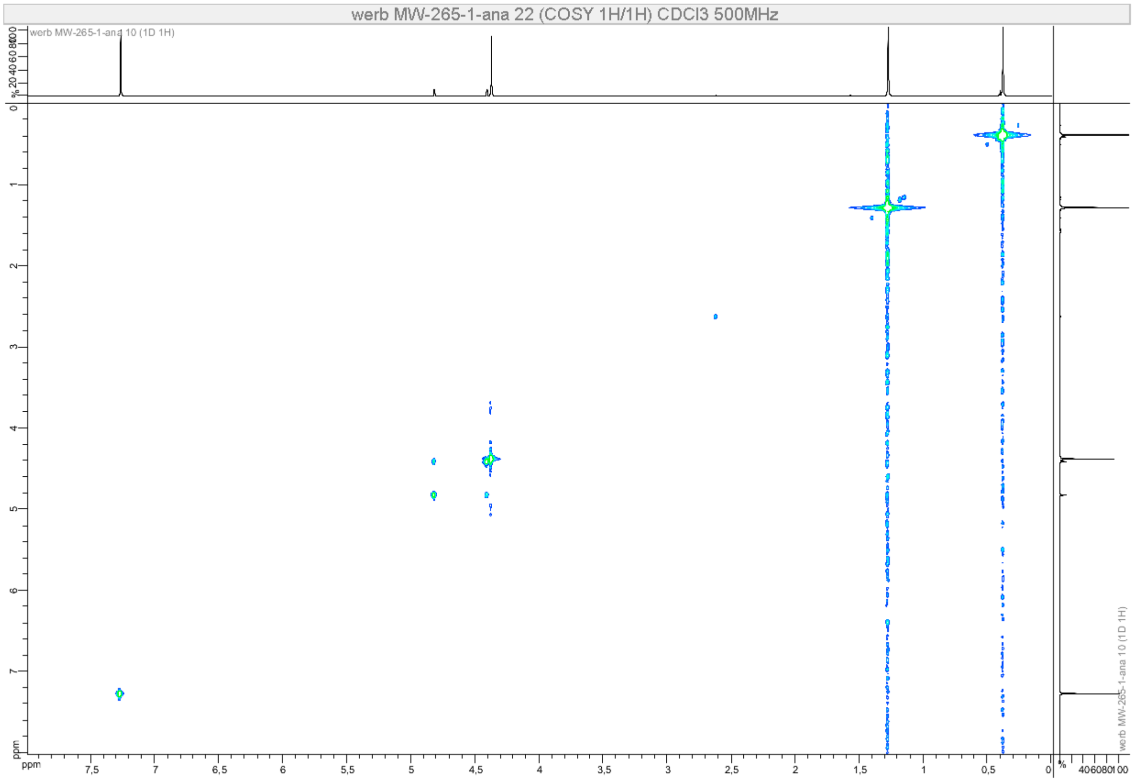

HSQC (500 MHz, $CDCl_3$)

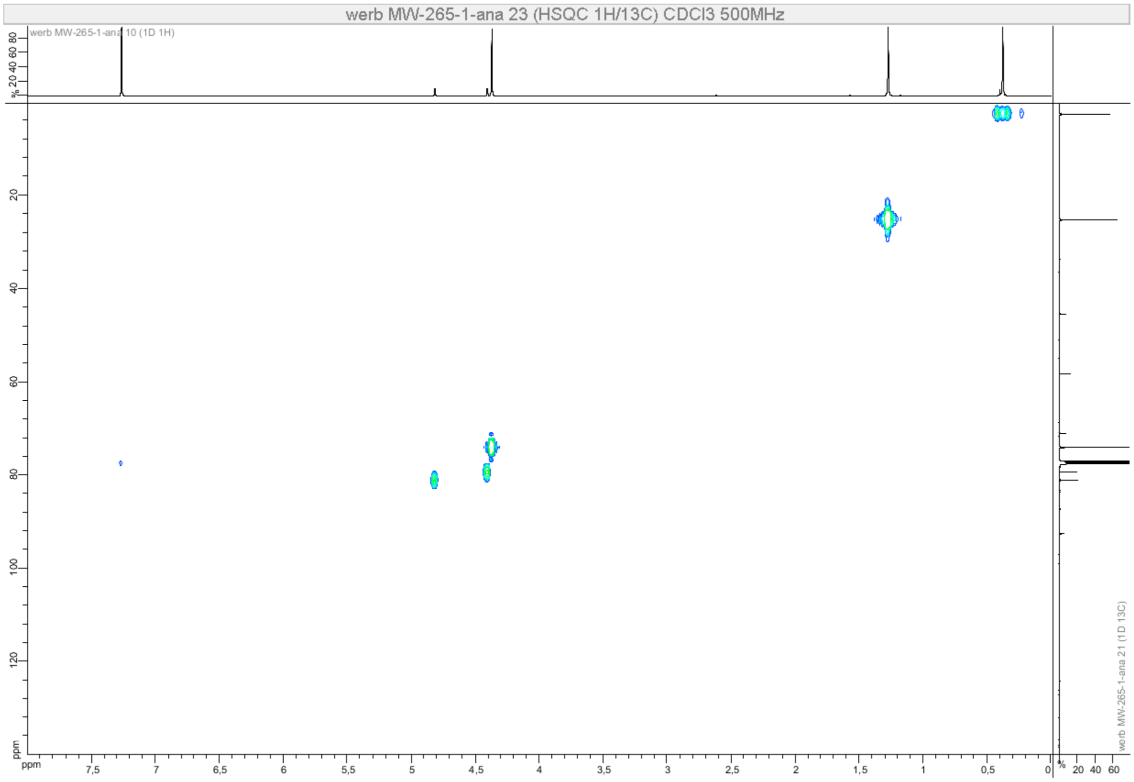

HMBC (500 MHz, $CDCl_3$)

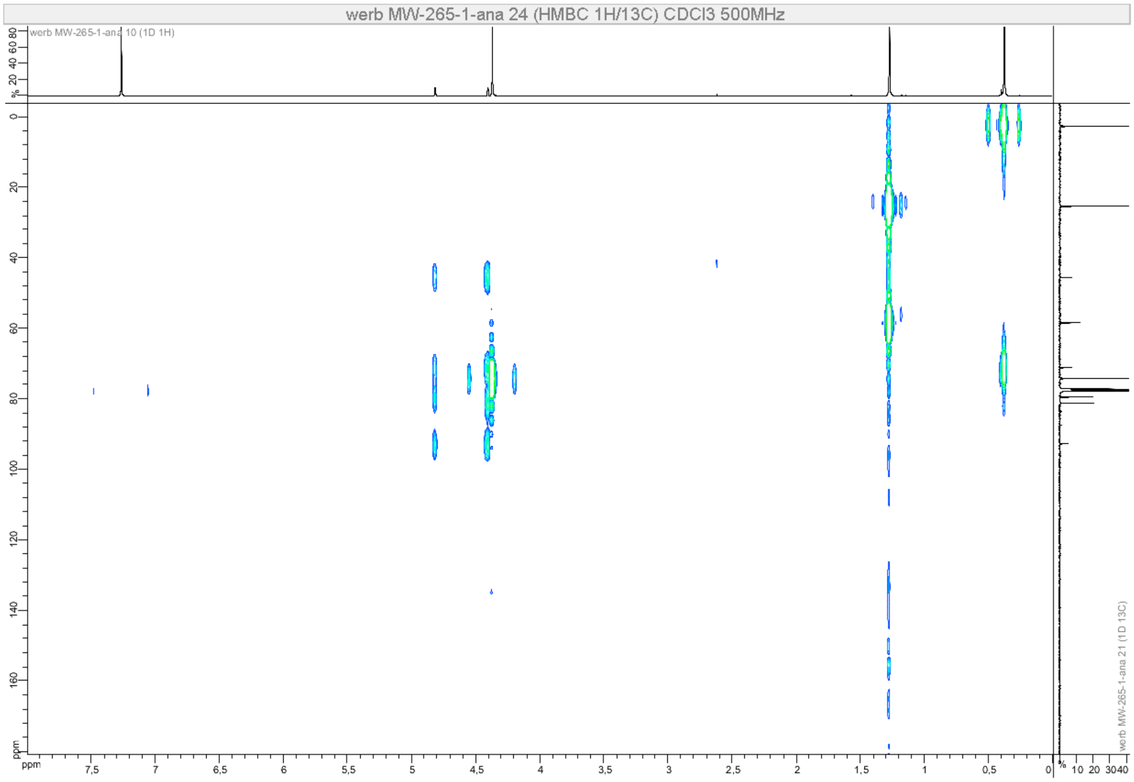

NOESY (500 MHz, $CDCl_3$)

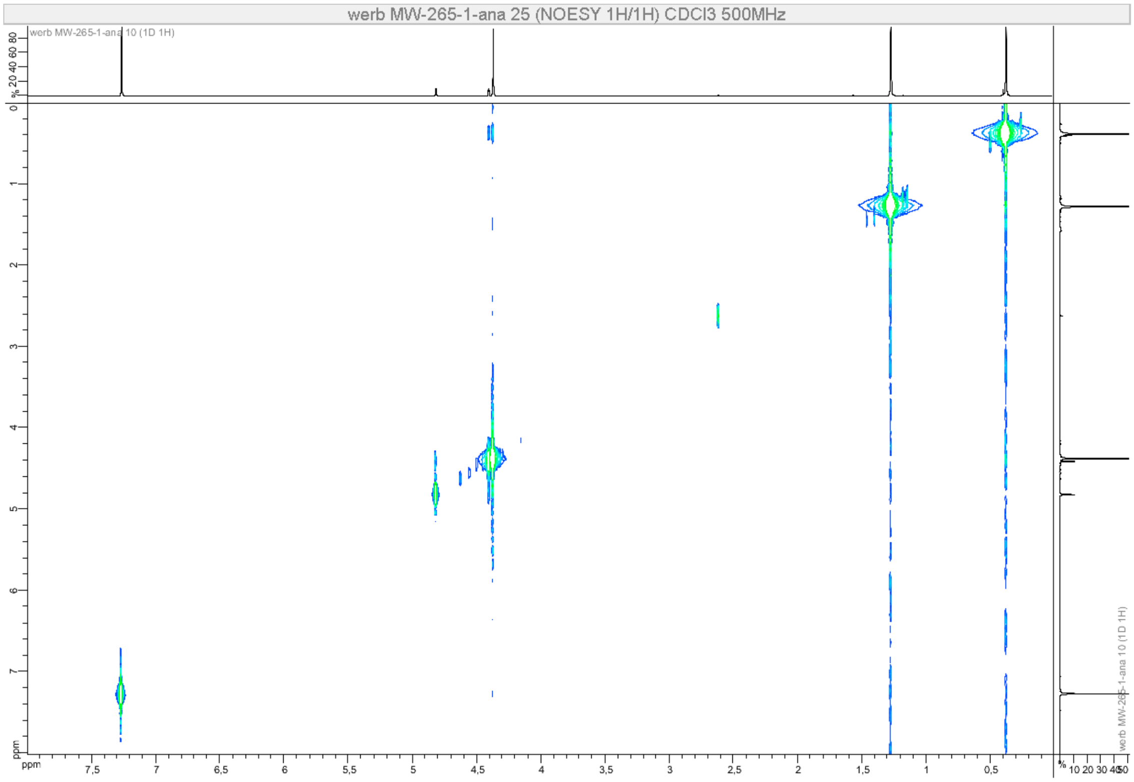

(*R*,*R*P)-*S*-*tert*-Butyl-2-iodo-5-(trimethylsilyl)ferrocenesulfoxide (*R*,*R*P-2ab)

^{1}H NMR (500 MHz, $CDCl_3$)

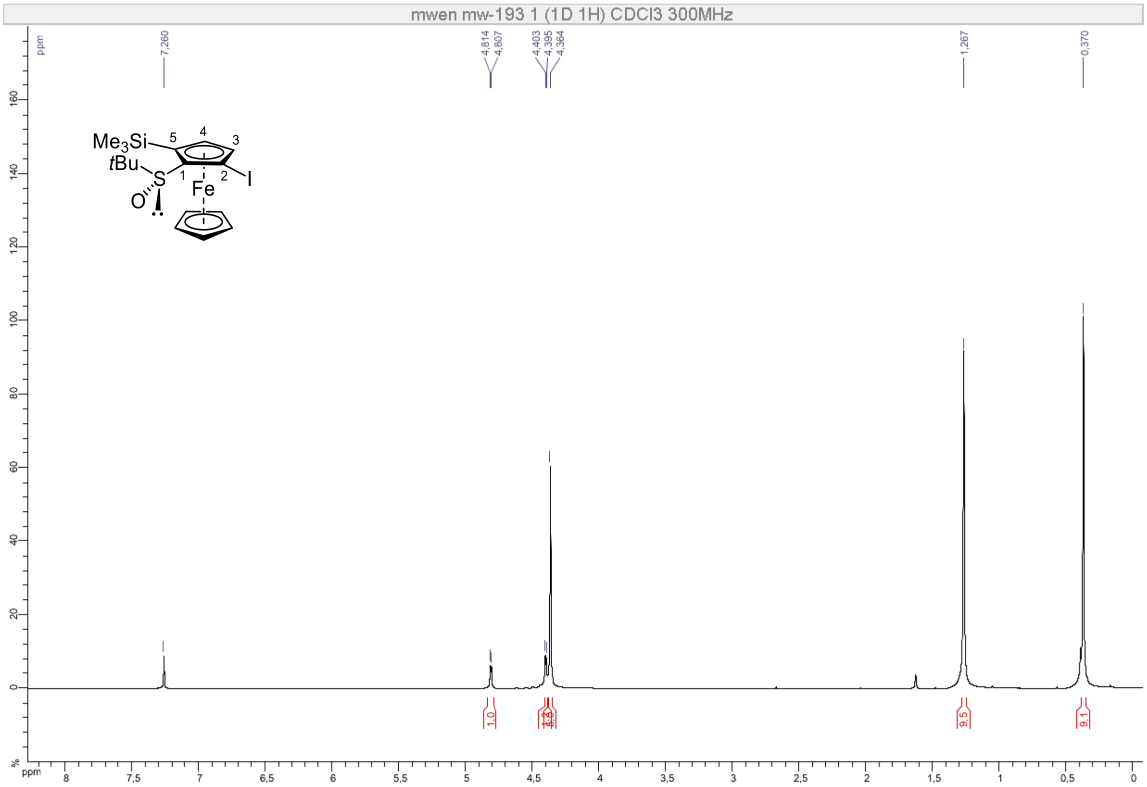

(*R*,*R*P)-*S*-*tert*-Butyl-2-iodoferrocenesulfoxide (*R*,*R*P-1b)

^{1}H NMR (500 MHz, $CDCl_3$)

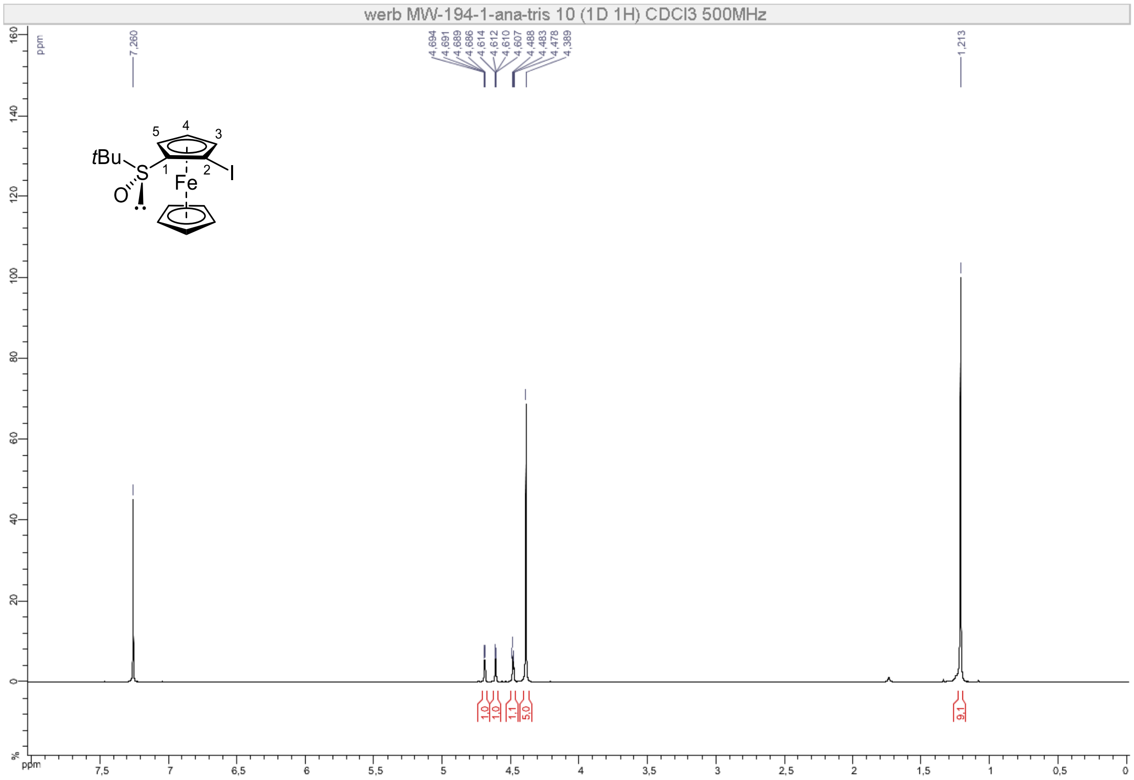

^{13}C NMR (126 MHz, $CDCl_3$)

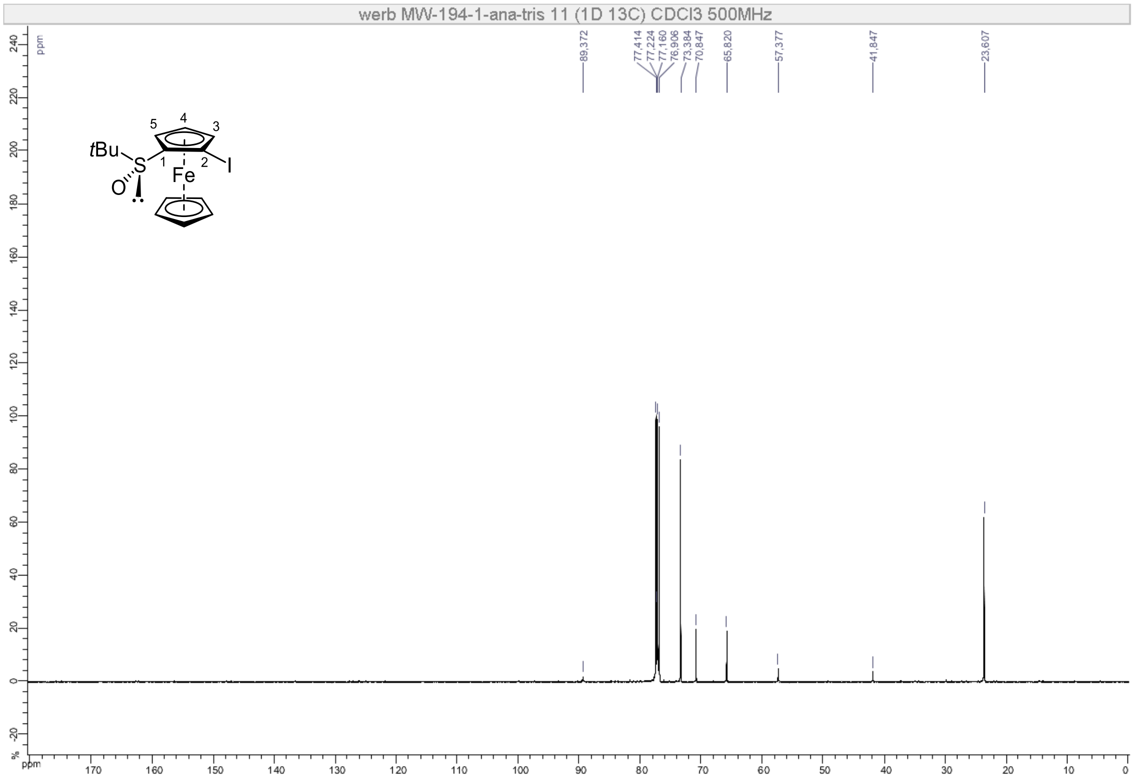

COSY (500 MHz, $CDCl_3$)

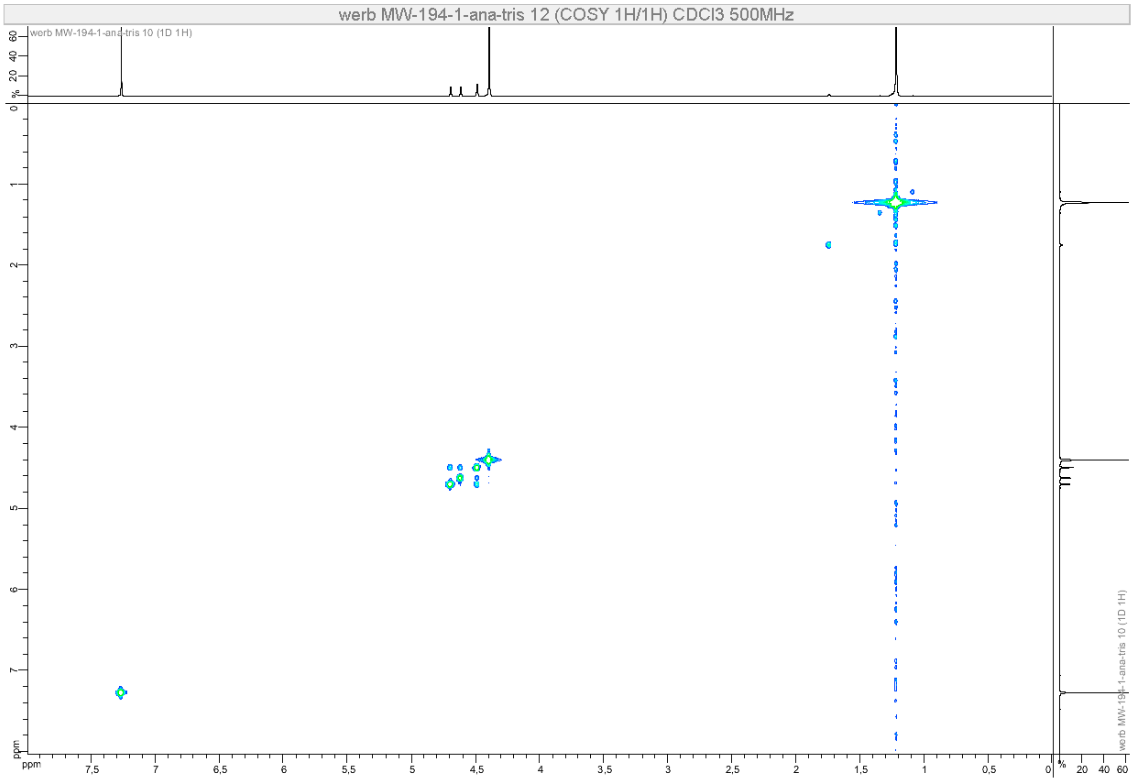

HSQC (500 MHz, $CDCl_3$)

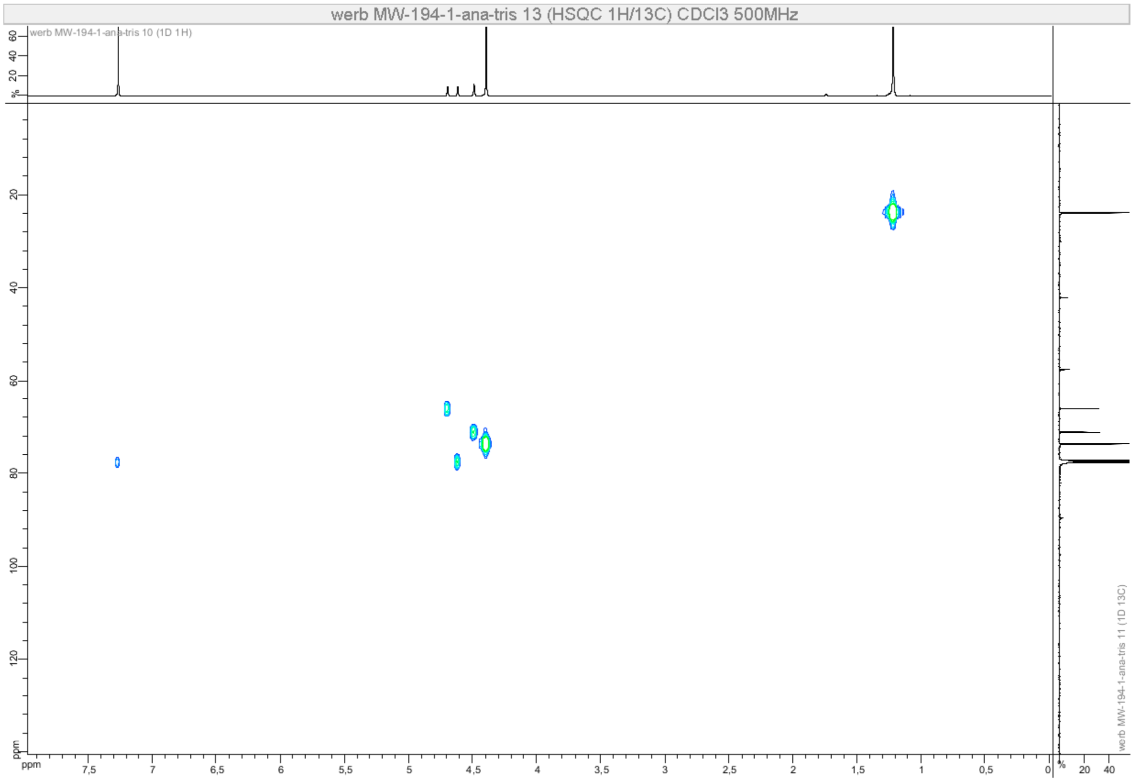

HMBC (500 MHz, $CDCl_3$)

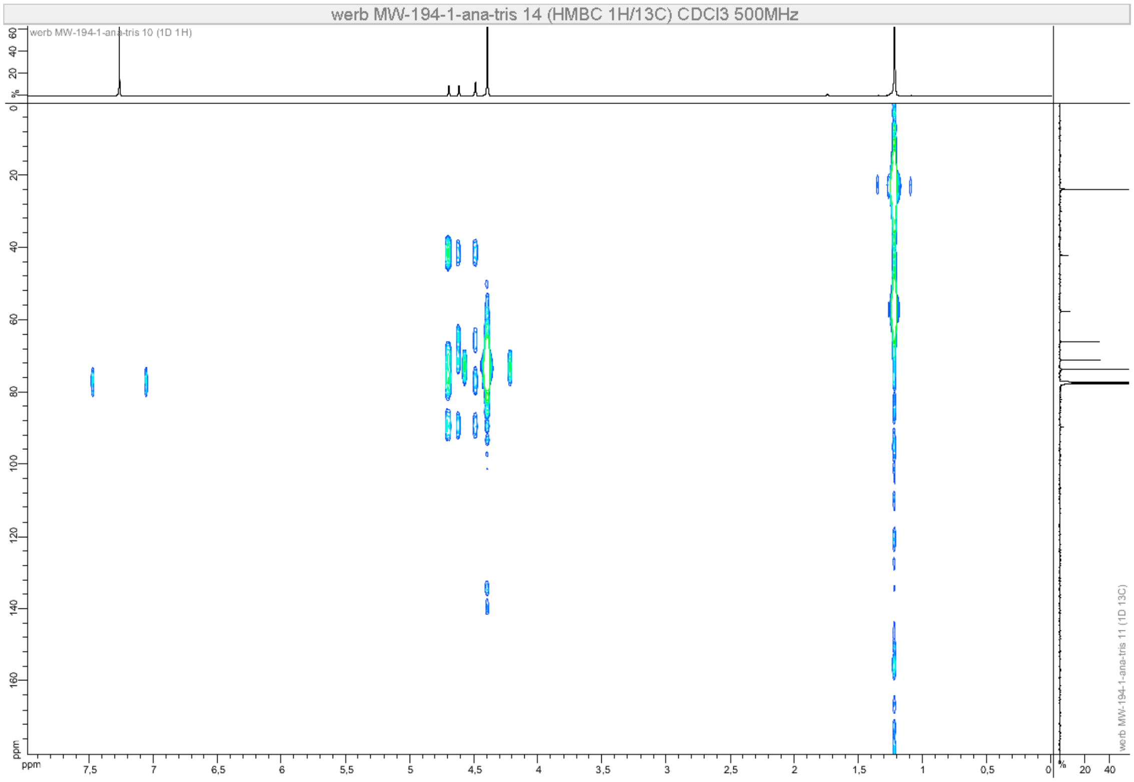

NOESY (500 MHz, $CDCl_3$)

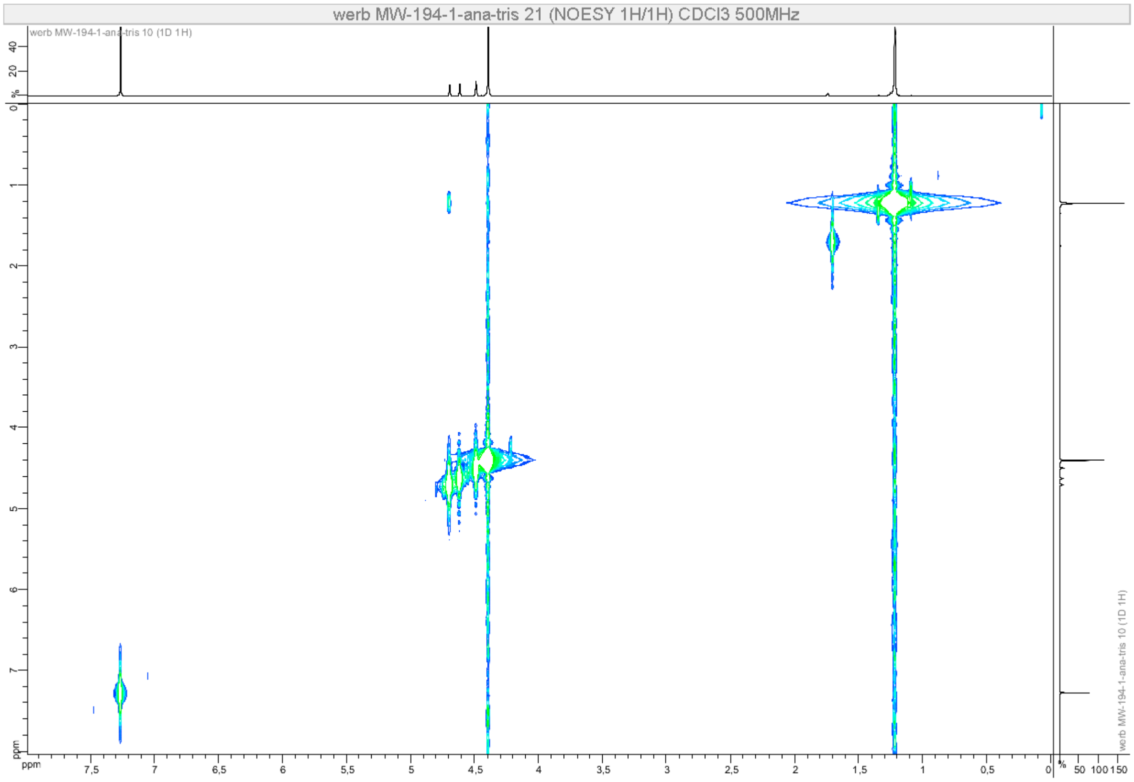

(*S*,*S*P)-*S*-*tert*-Butyl-2-(diphenylphosphinyl)-5-iodoferrocenesulfoxide (*S*,*S*P-2eb)

^{1}H NMR (500 MHz, CDCl$_3$)

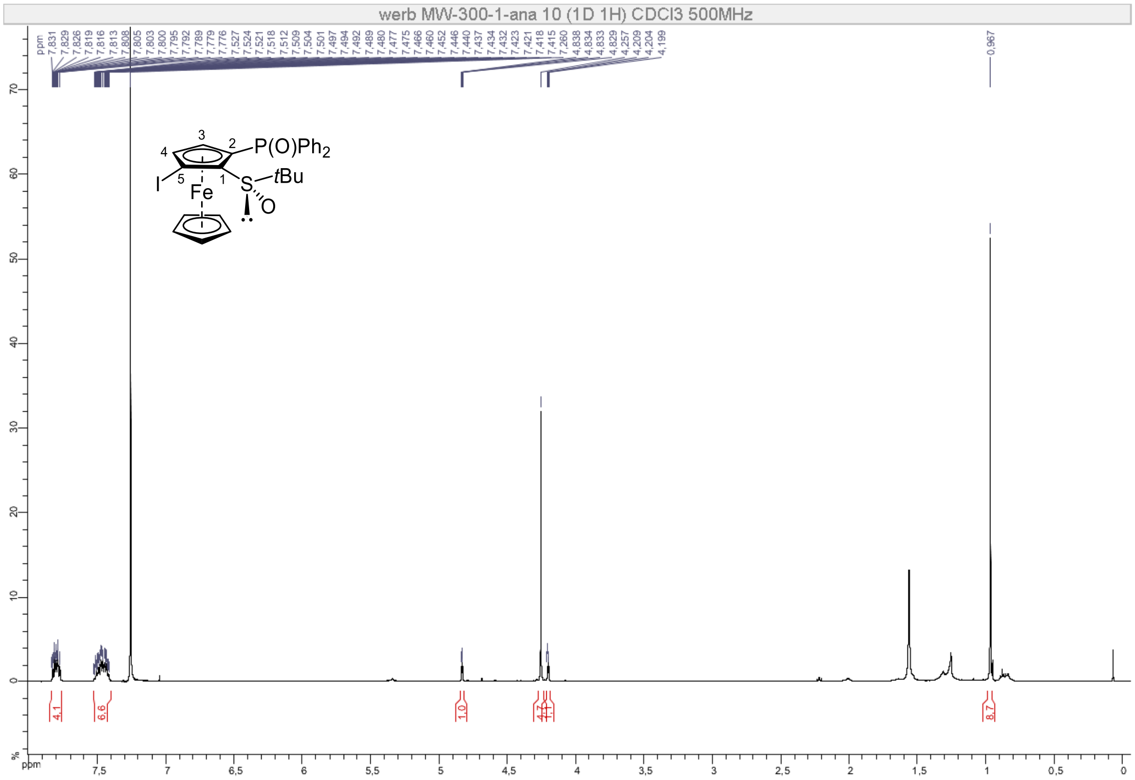

^{13}C NMR (126 MHz, CDCl$_3$)

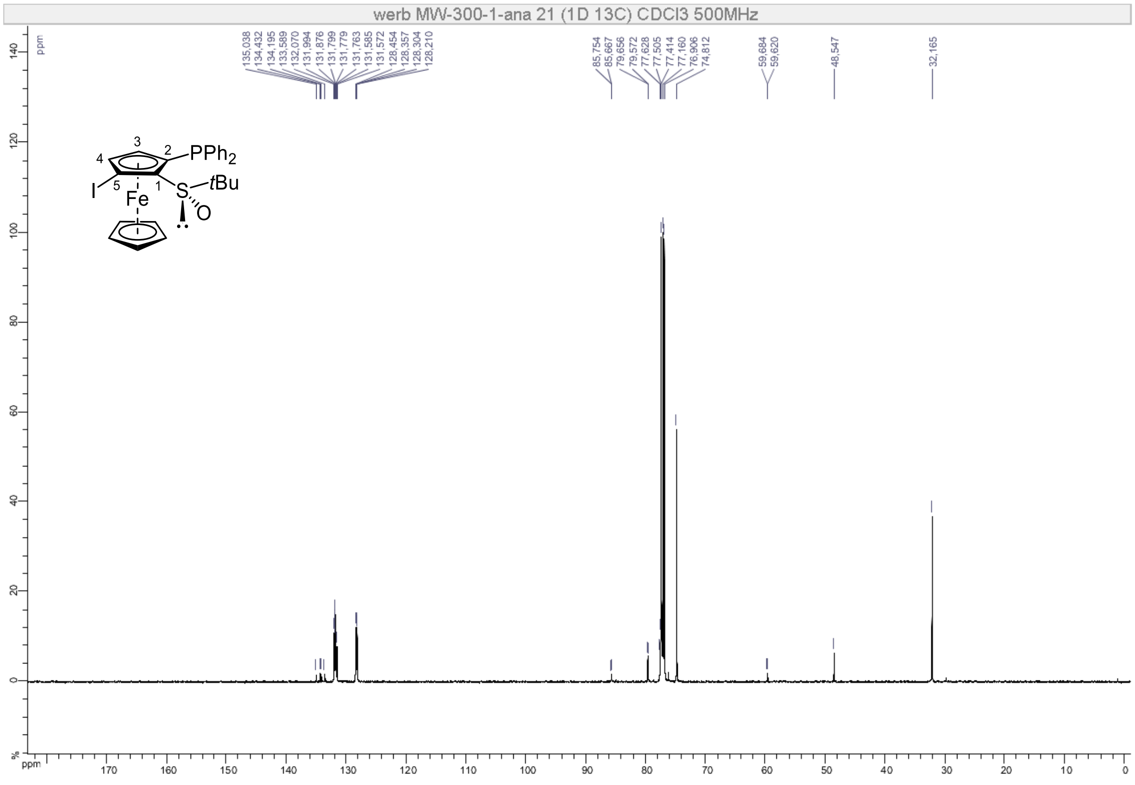

COSY (500 MHz, $CDCl_3$)

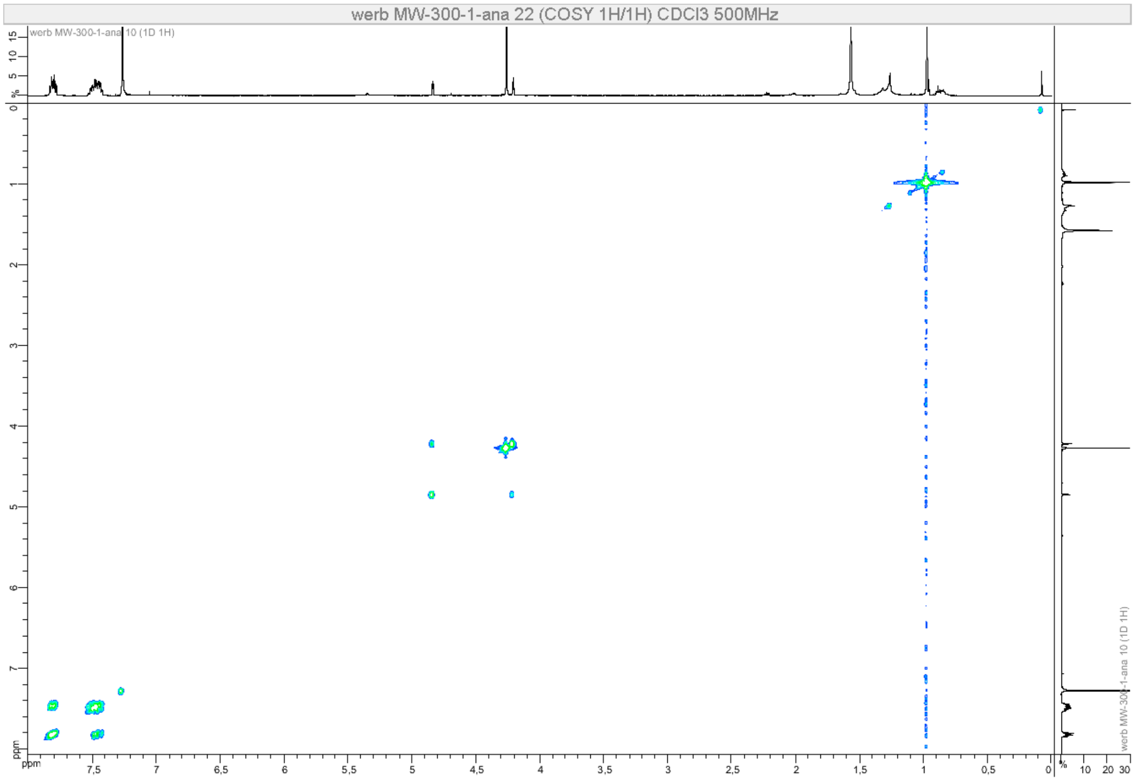

HSQC (500 MHz, $CDCl_3$)

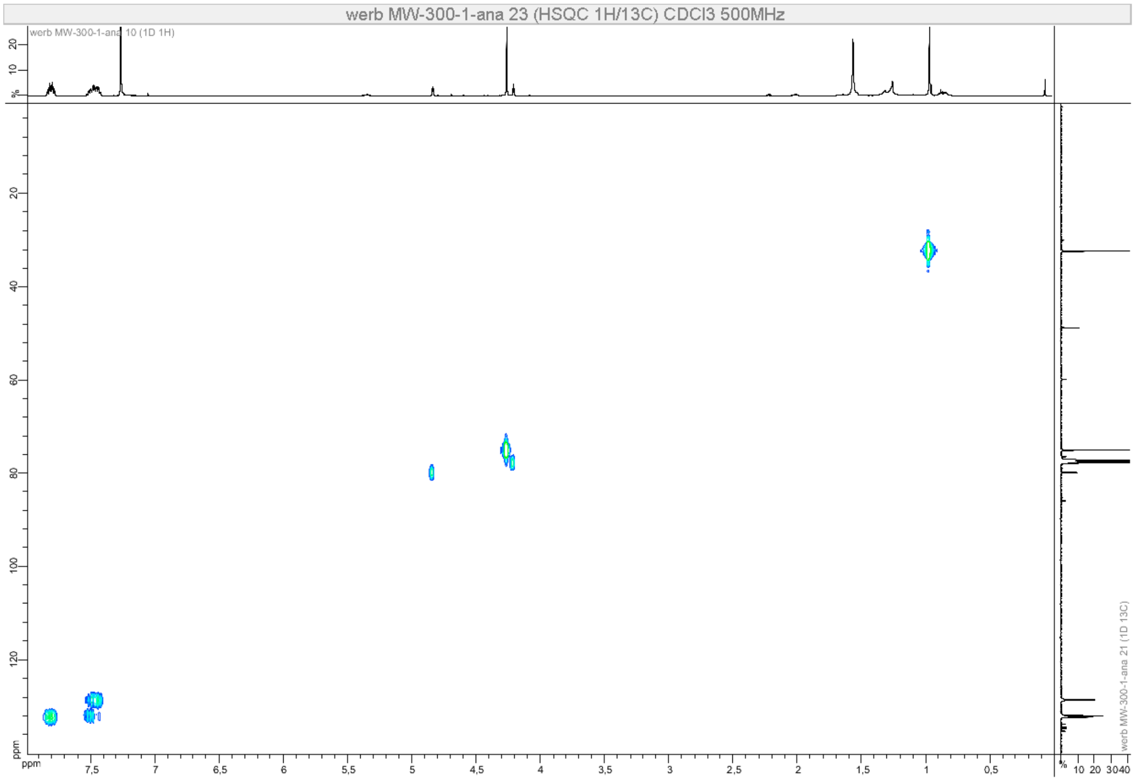

HMBC (500 MHz, $CDCl_3$)

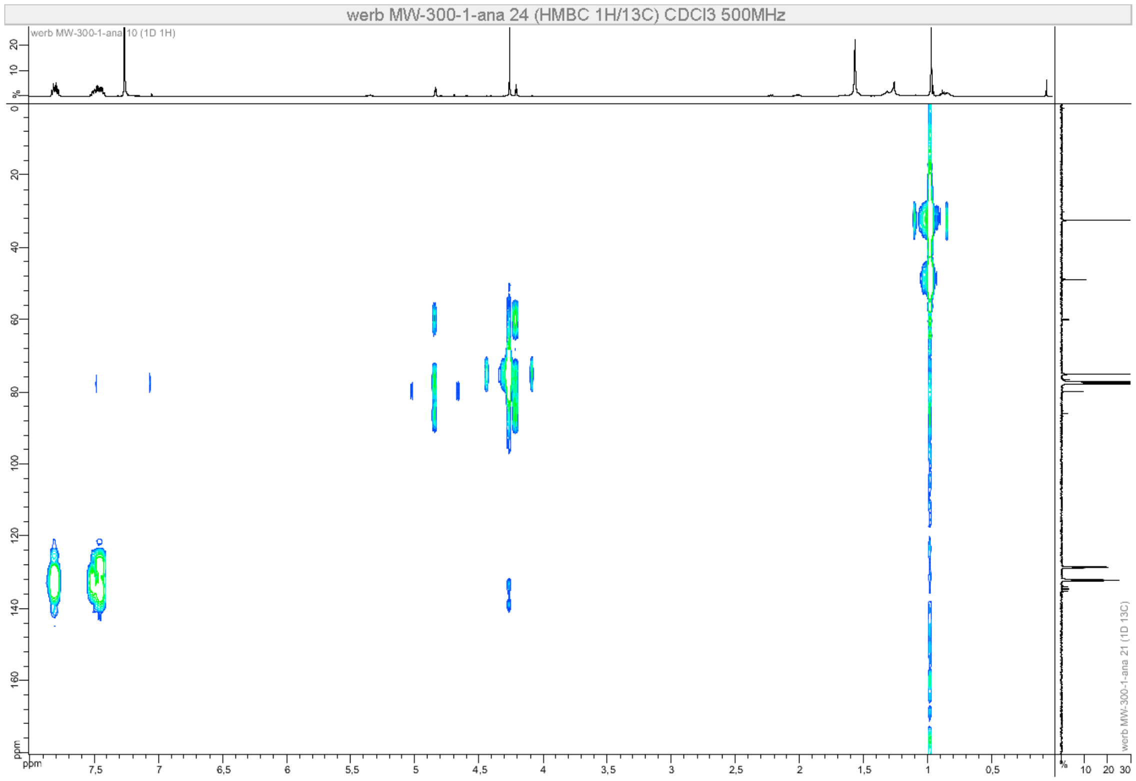

NOESY (500 MHz, $CDCl_3$)

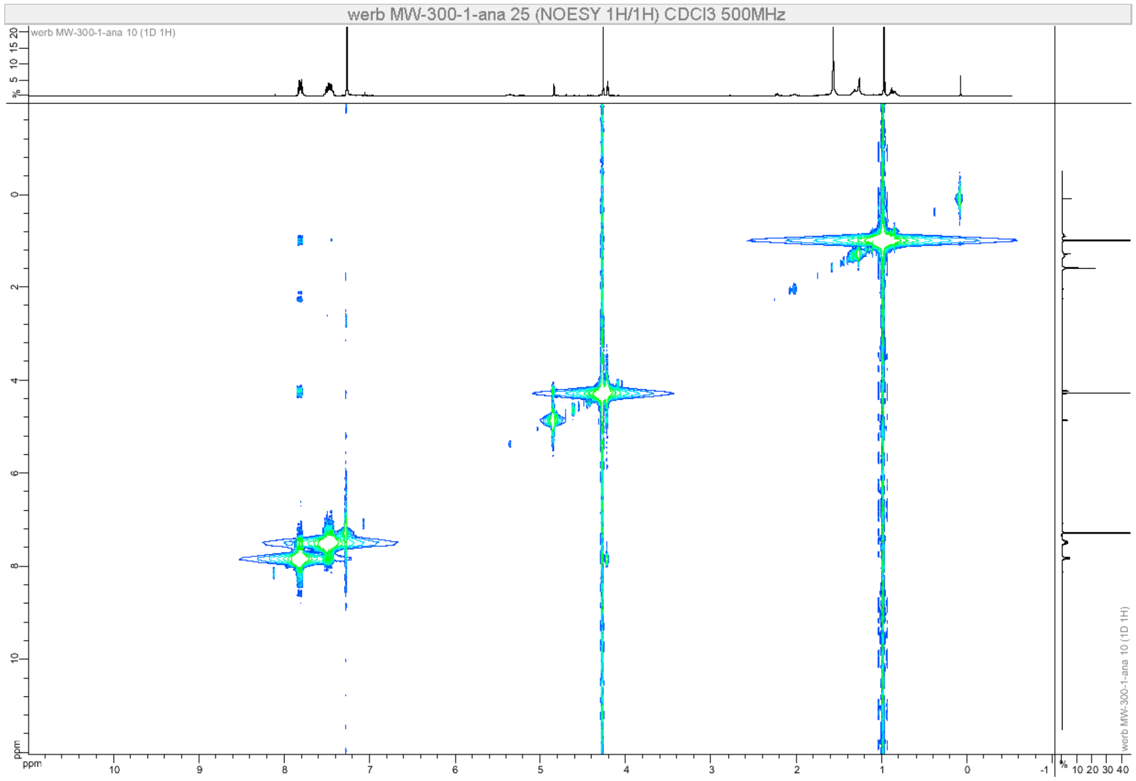

^{31}P NMR (202 MHz, $CDCl_3$)

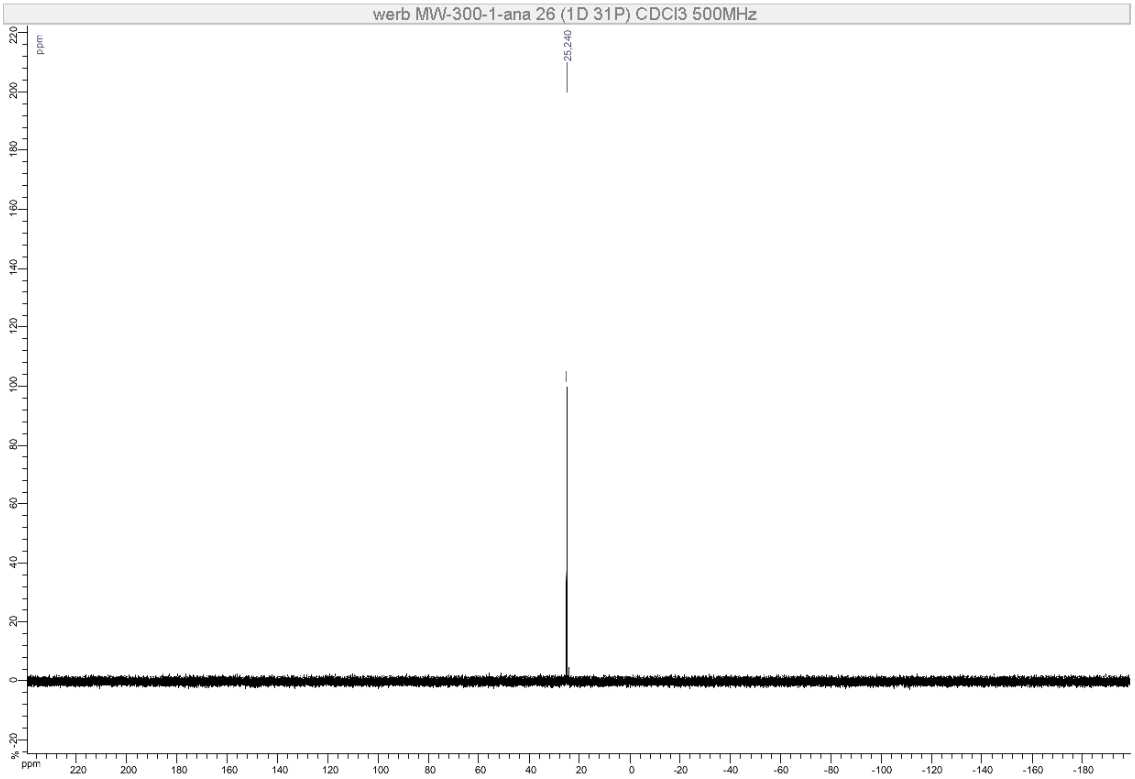

(*S*,*R*P)-2-Bromo-*S*-*tert*-butyl-5-(trimethylsilyl)ferrocenesulfoxide (*S*,*R*P-2ja)

^{1}H NMR (500 MHz, $CDCl_3$)

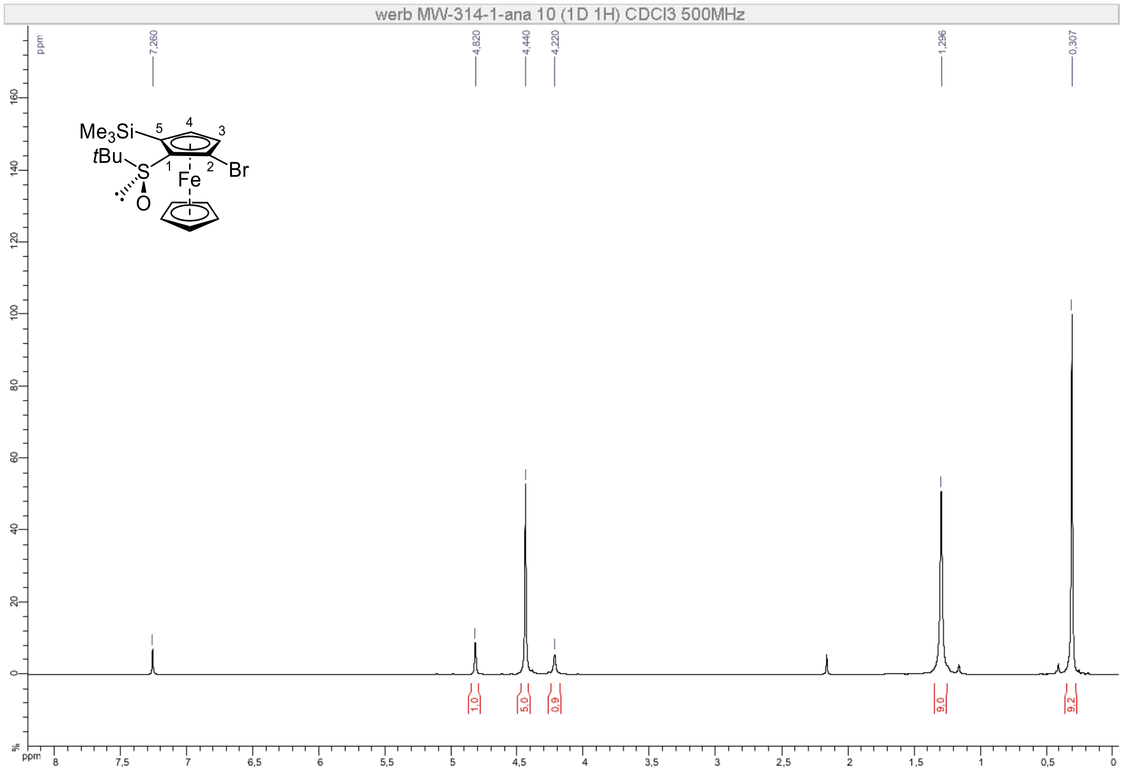

^{13}C NMR (126 MHz, $CDCl_3$)

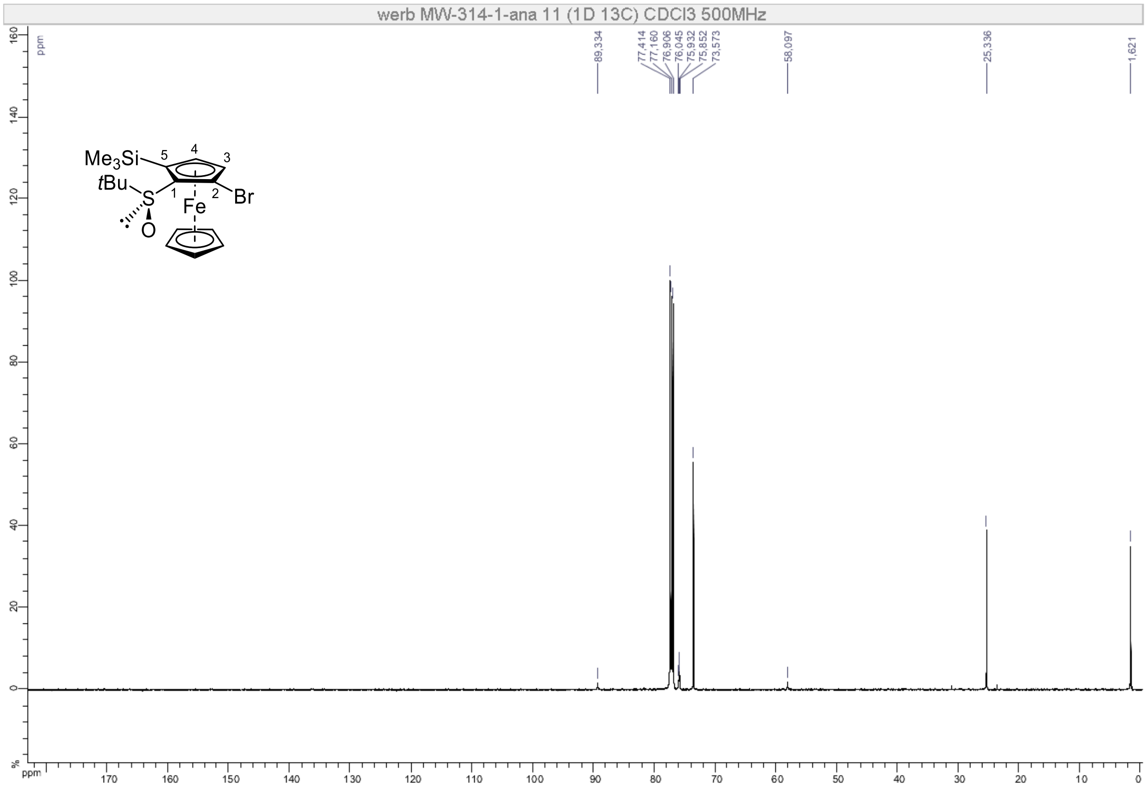

COSY (500 MHz, $CDCl_3$)

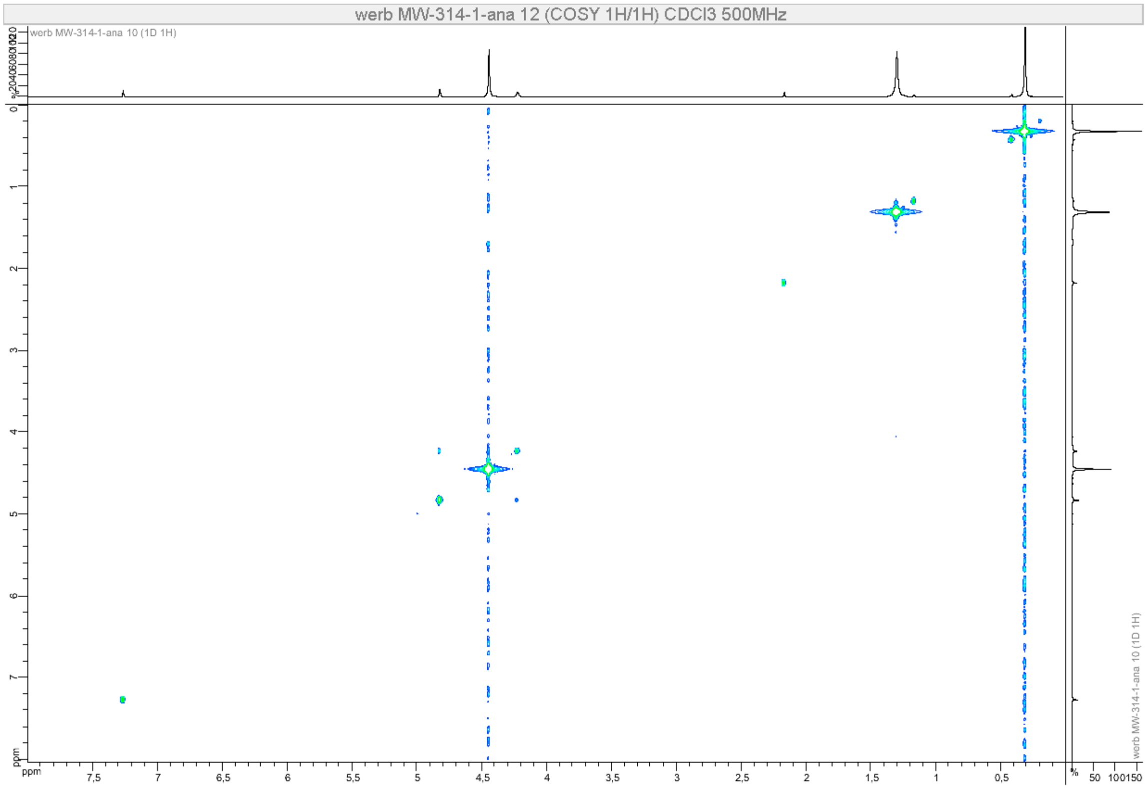

HSQC (500 MHz, $CDCl_3$)

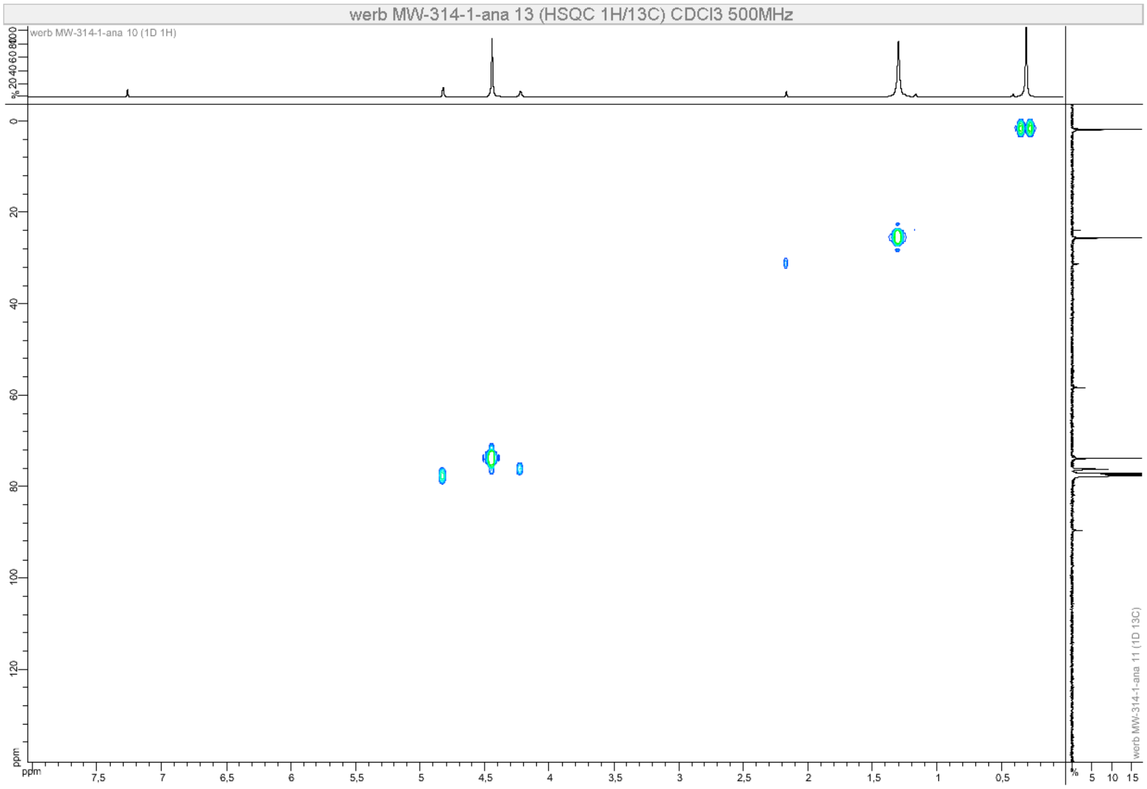

HMBC (500 MHz, $CDCl_3$)

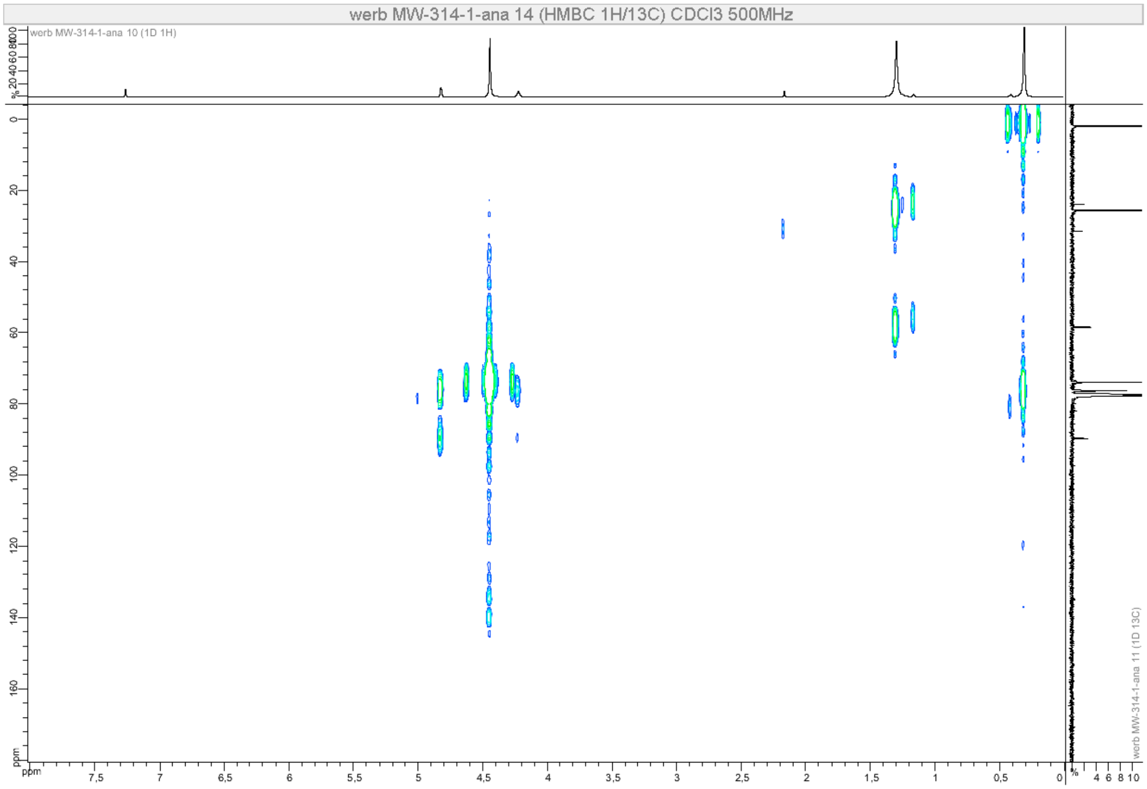

(S,S_P)-*S-tert*-Butyl-2-(diphenylphosphino)ferrocenesulfoxide (S,S_P-1e)

^{1}H NMR (500 MHz, $CDCl_3$)

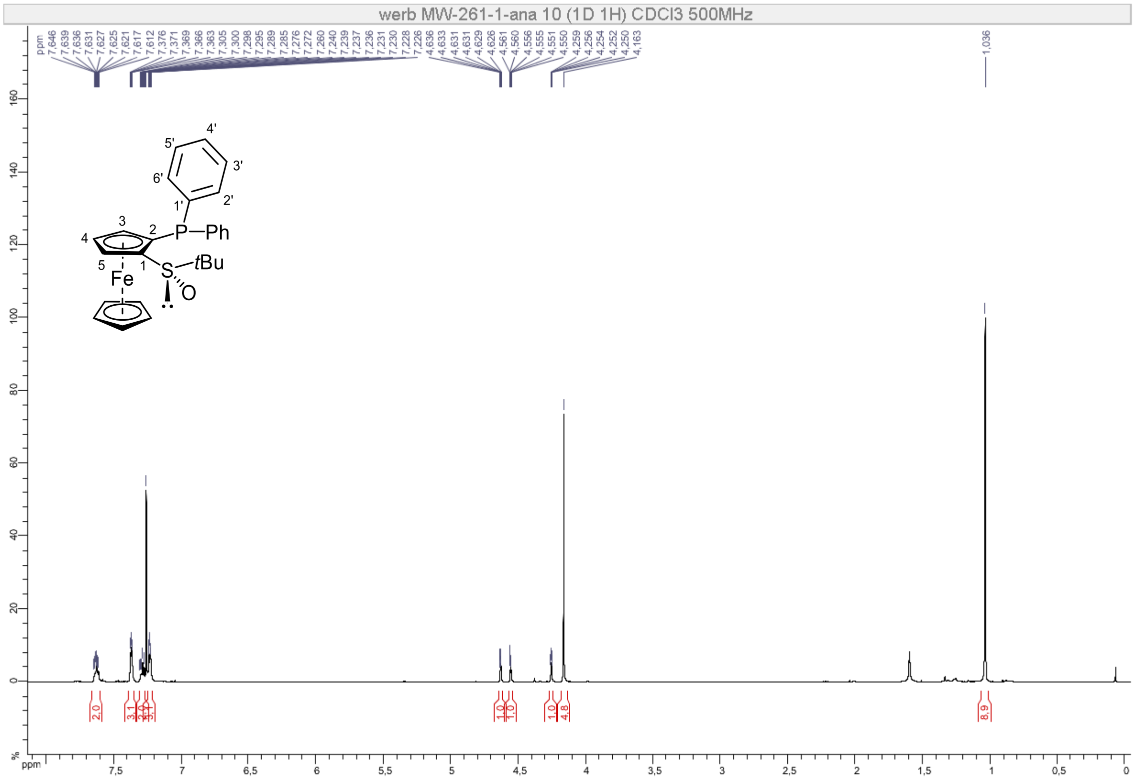

^{13}C NMR (126 MHz, $CDCl_3$)

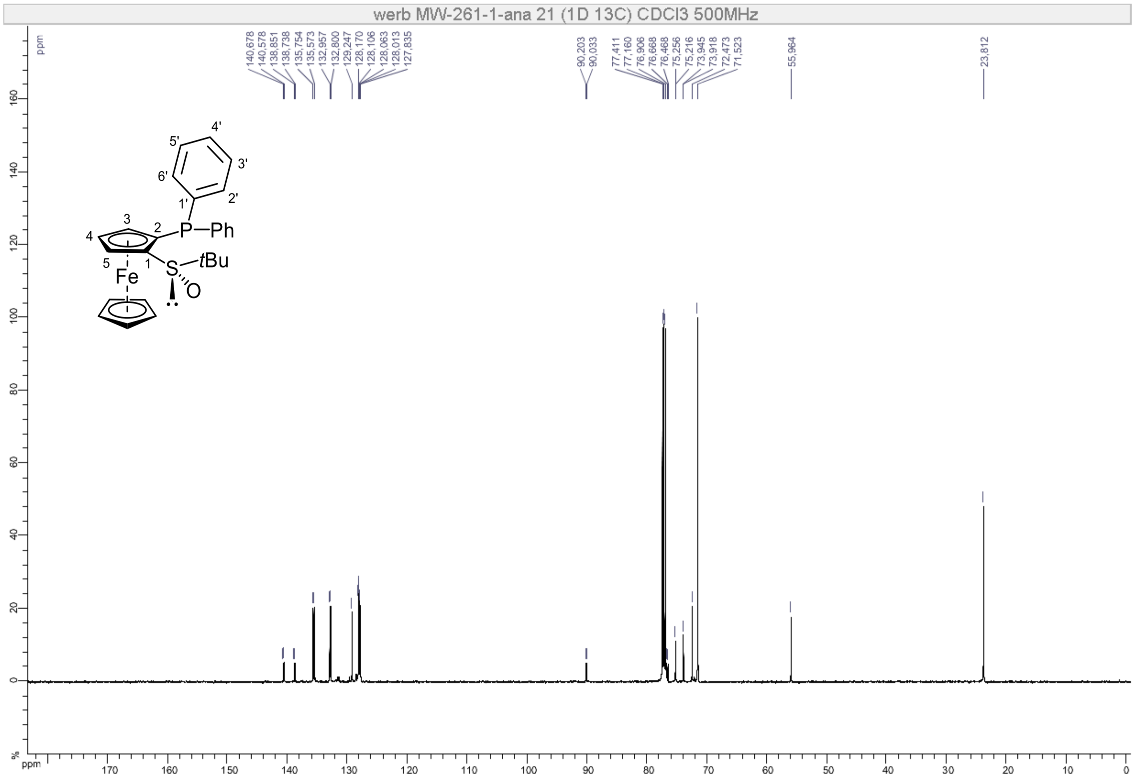

COSY (500 MHz, $CDCl_3$)

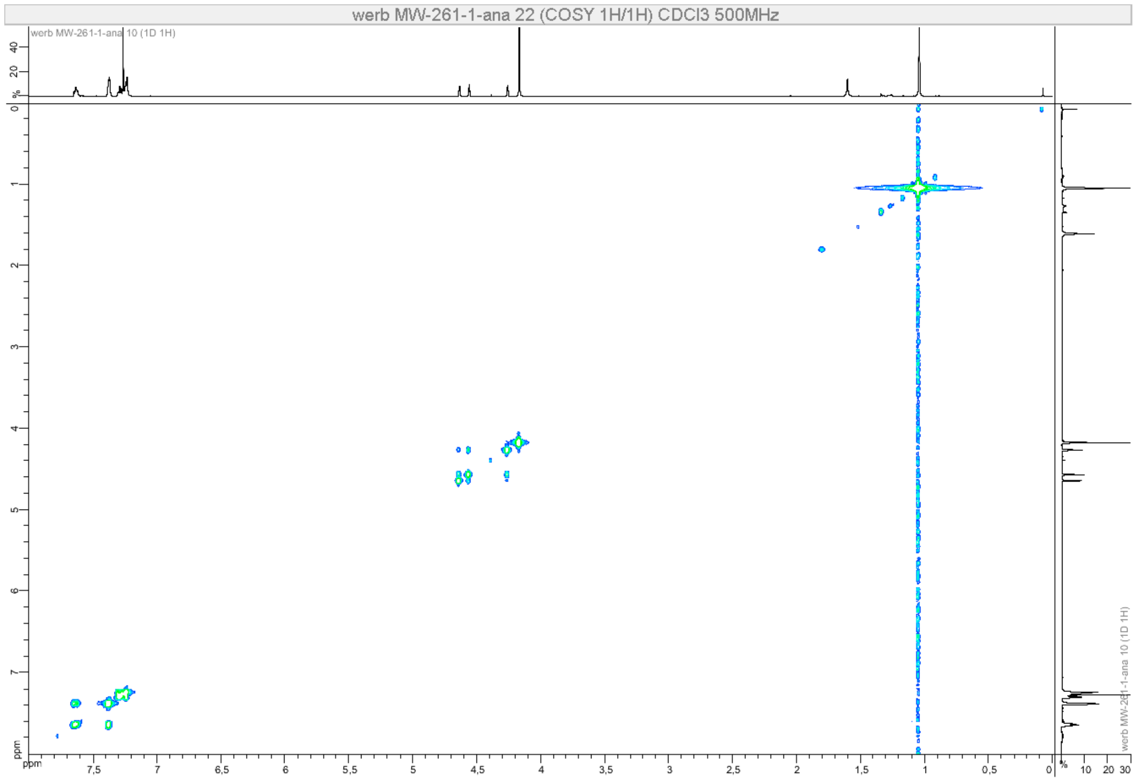

HSQC (500 MHz, $CDCl_3$)

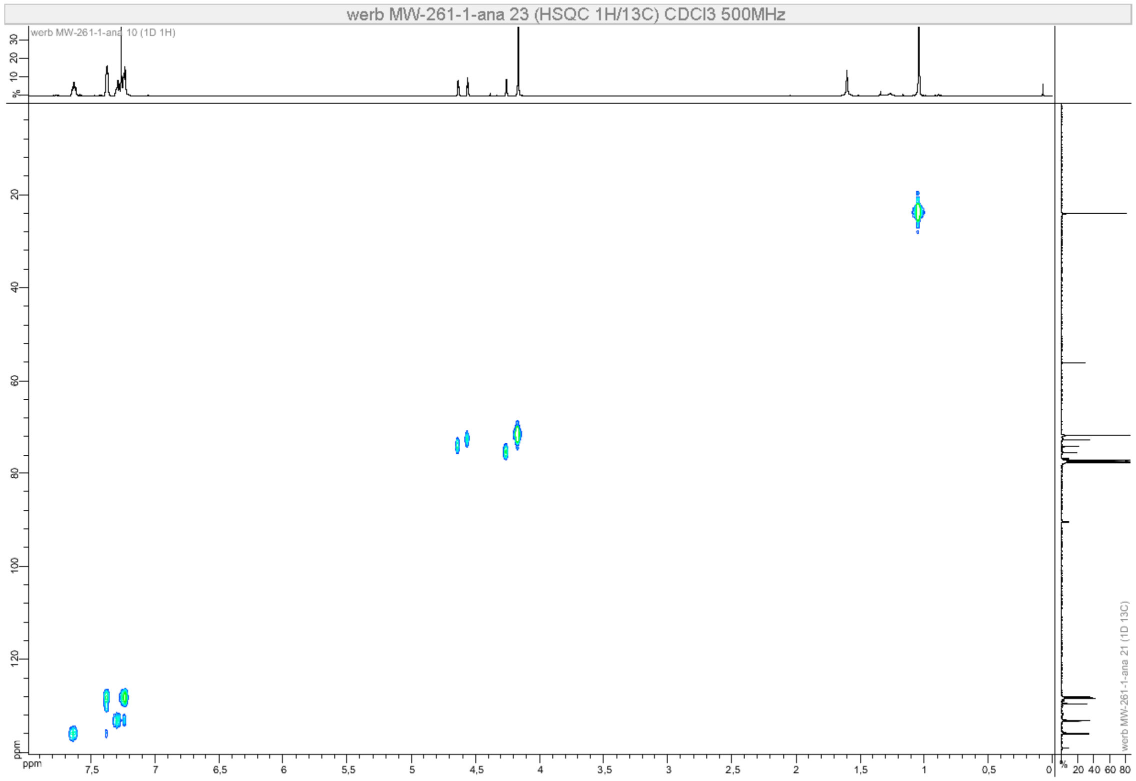

HMBC (500 MHz, $CDCl_3$)

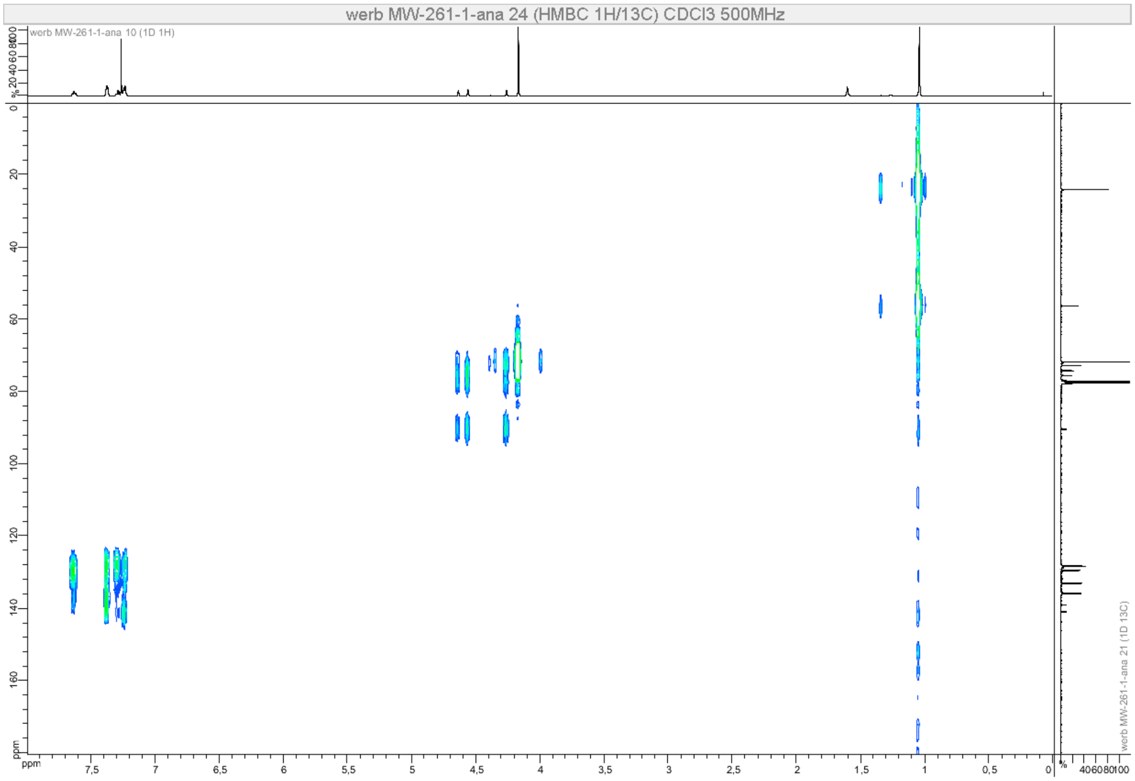

NOESY (500 MHz, $CDCl_3$)

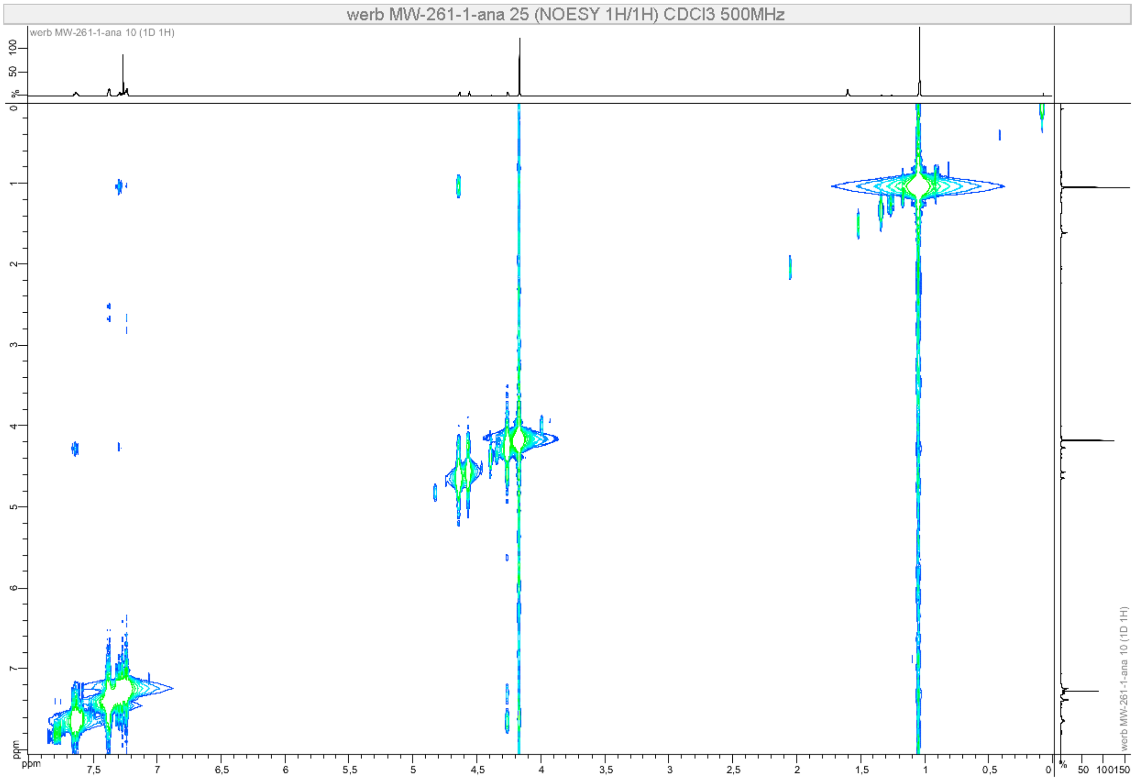

^{31}P NMR (202 MHz, $CDCl_3$)

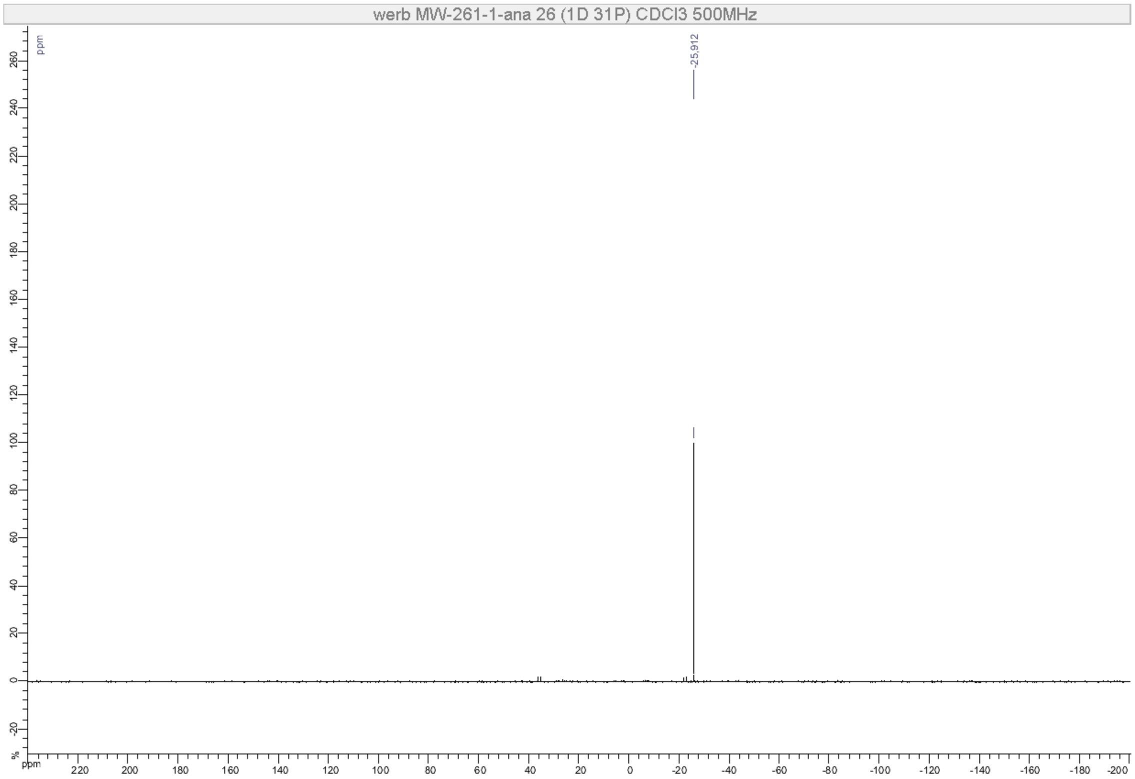

(*S*,*R*P)-*S*-*tert*-Butyl-2-(tributylstannyl)ferrocenesulfoxide (*S*,*R*P-1f)

^{1}H NMR (500 MHz, $CDCl_3$)

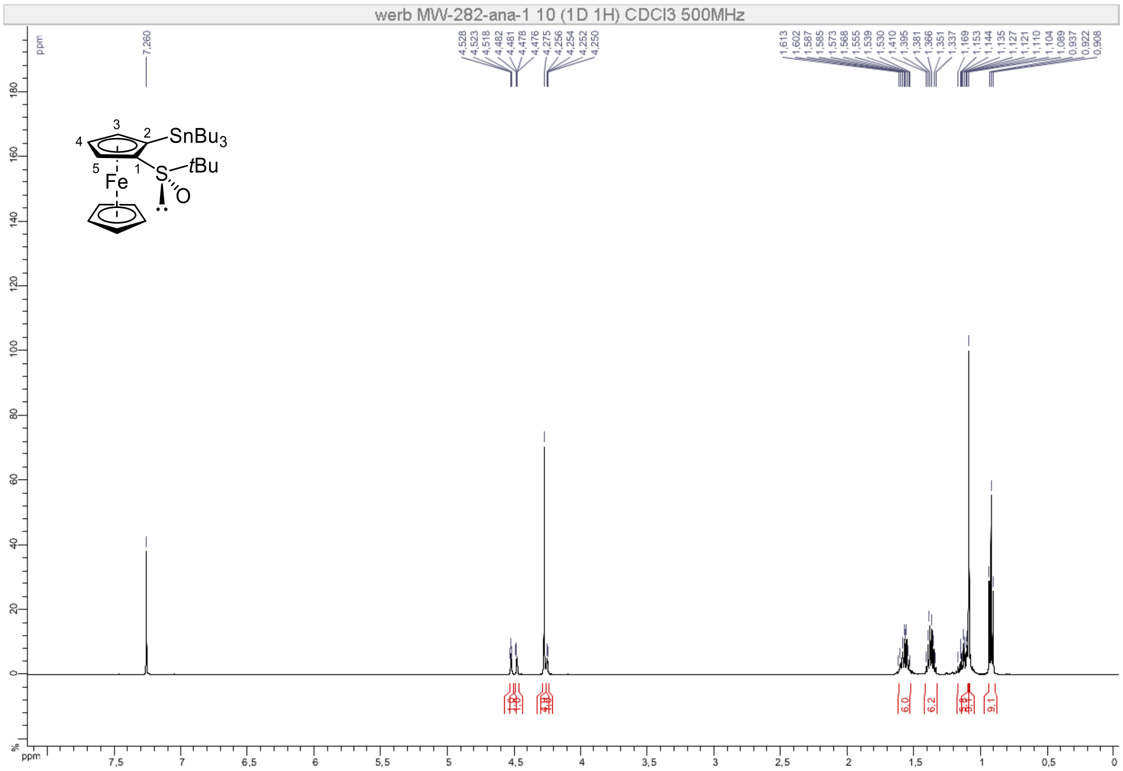

^{13}C NMR (126 MHz, $CDCl_3$)

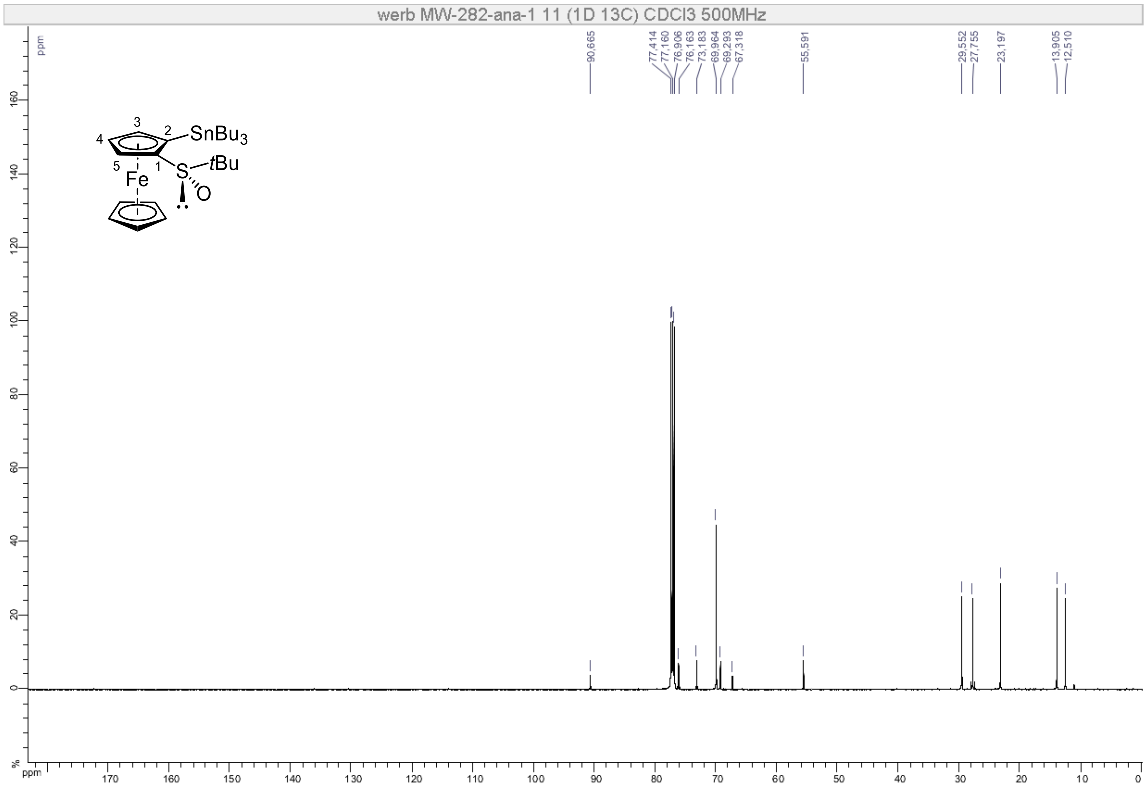

DEPT 135 (126 MHz, $CDCl_3$)

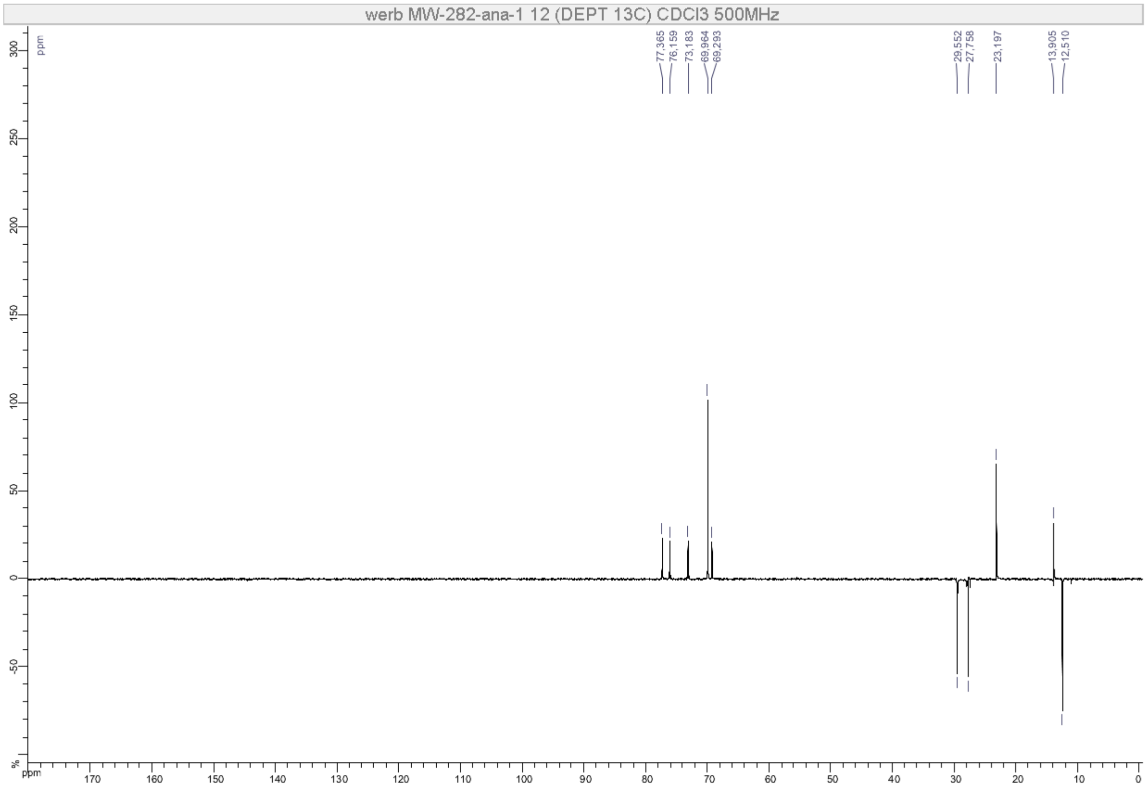

COSY (500 MHz, $CDCl_3$)

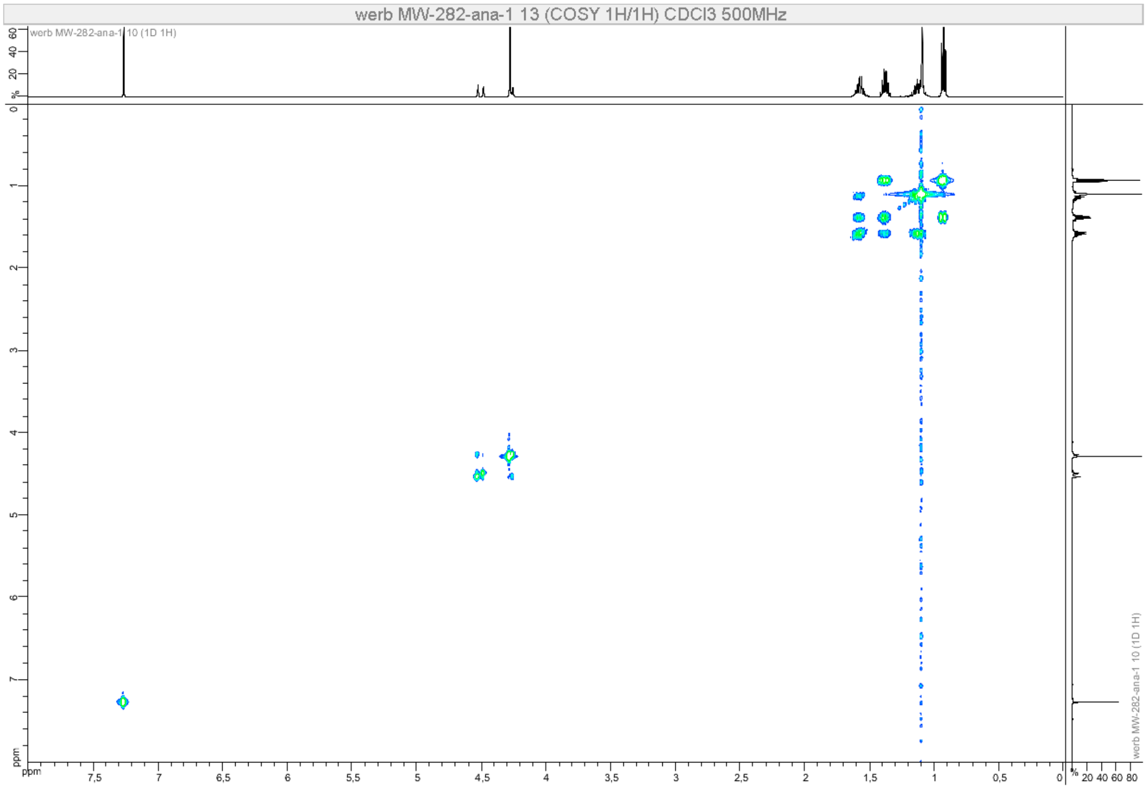

HSQC (500 MHz, $CDCl_3$)

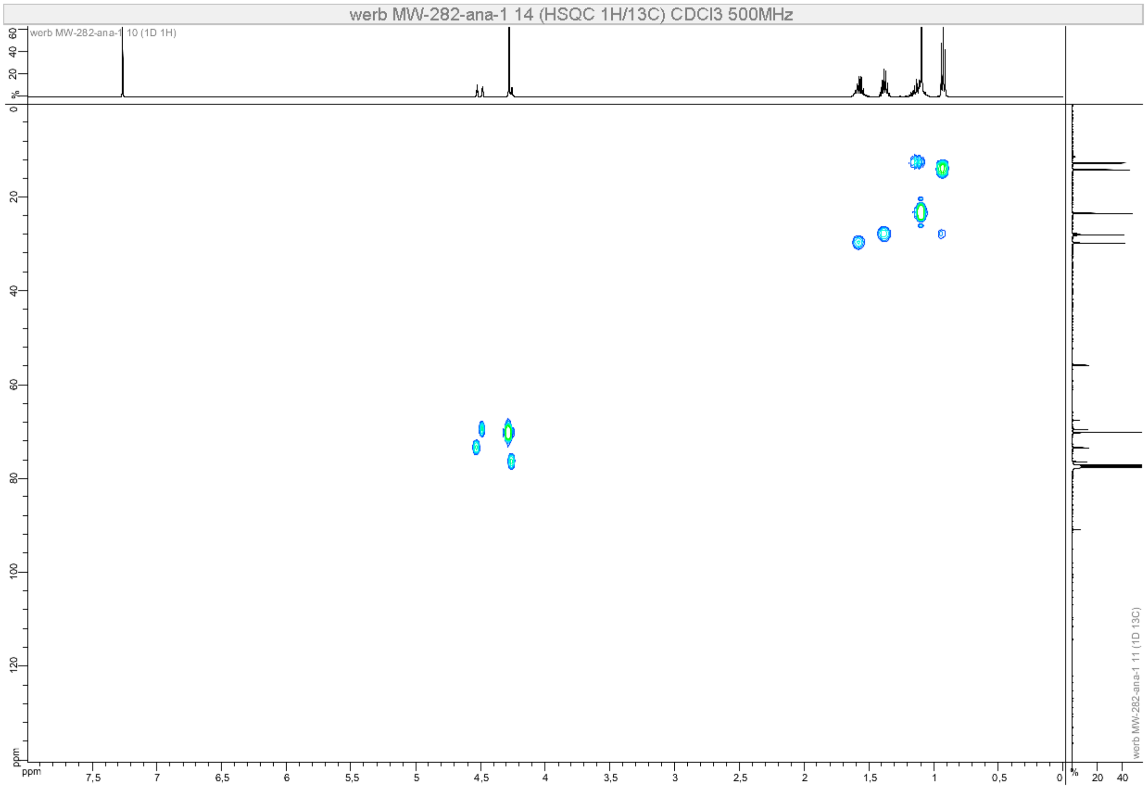

HMBC (500 MHz, $CDCl_3$)

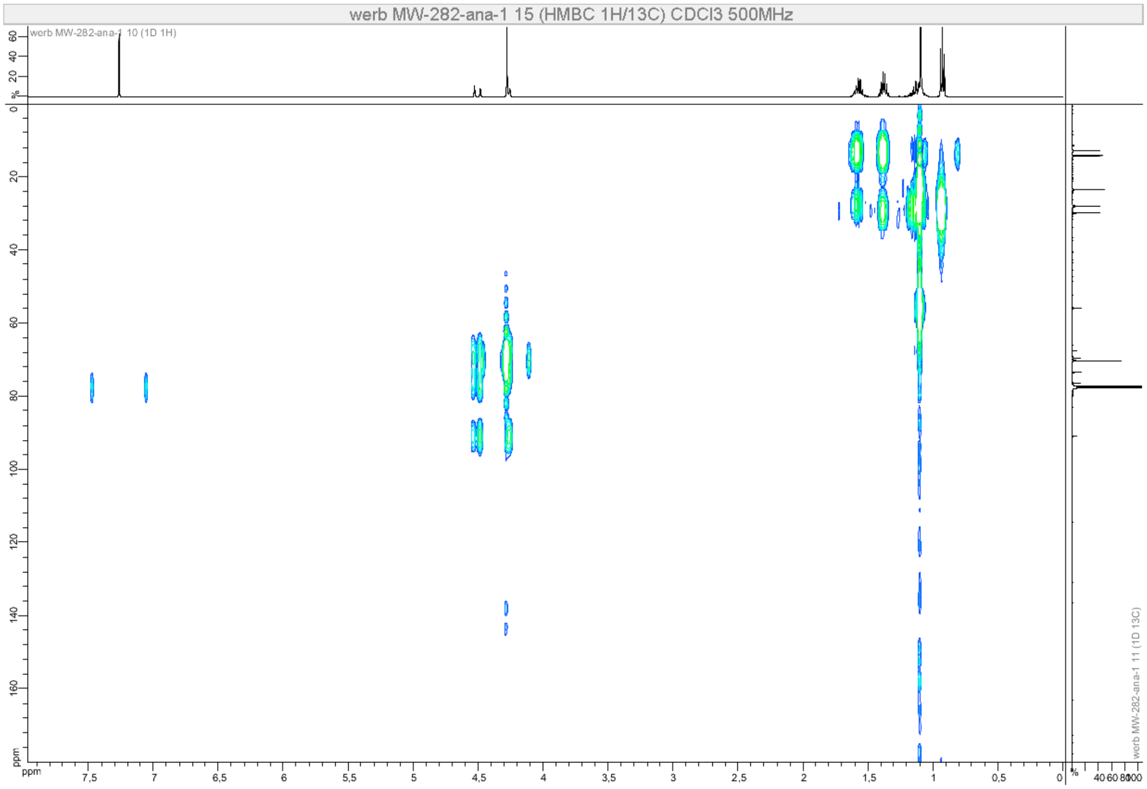

NOESY (500 MHz, $CDCl_3$)

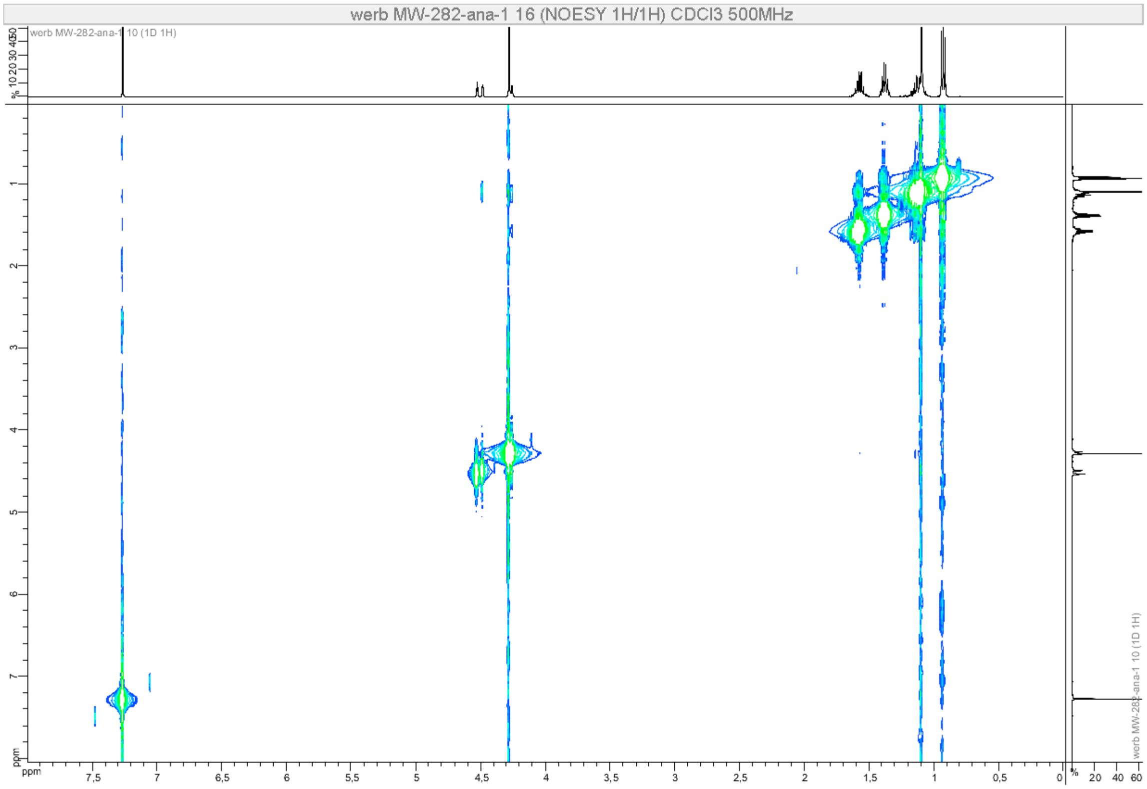

(*S*,*S*P)-*S*-*tert*-Butyl-2-[(α,α-diphenyl)hydroxymethyl]-5-(trimethylsilyl)ferrocenesulfoxide (*S*,*S*P-2ag)

^{1}H NMR (500 MHz, $CDCl_3$)

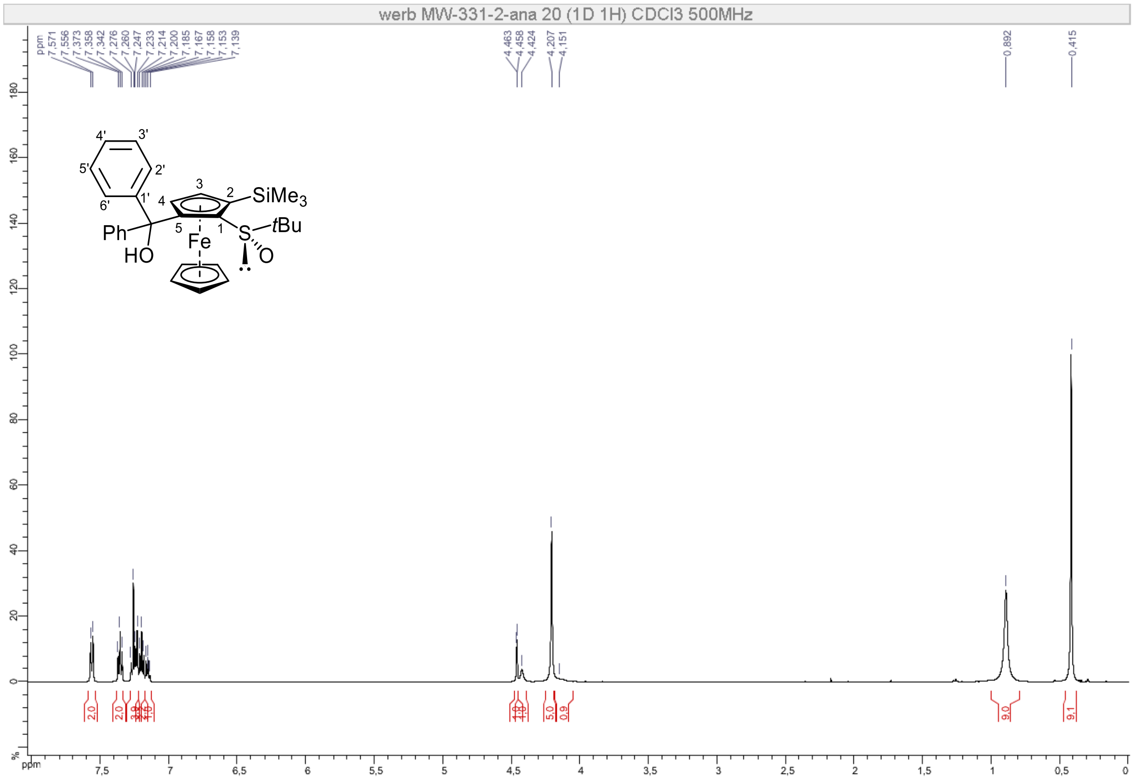

^{13}C NMR (126 MHz, $CDCl_3$)

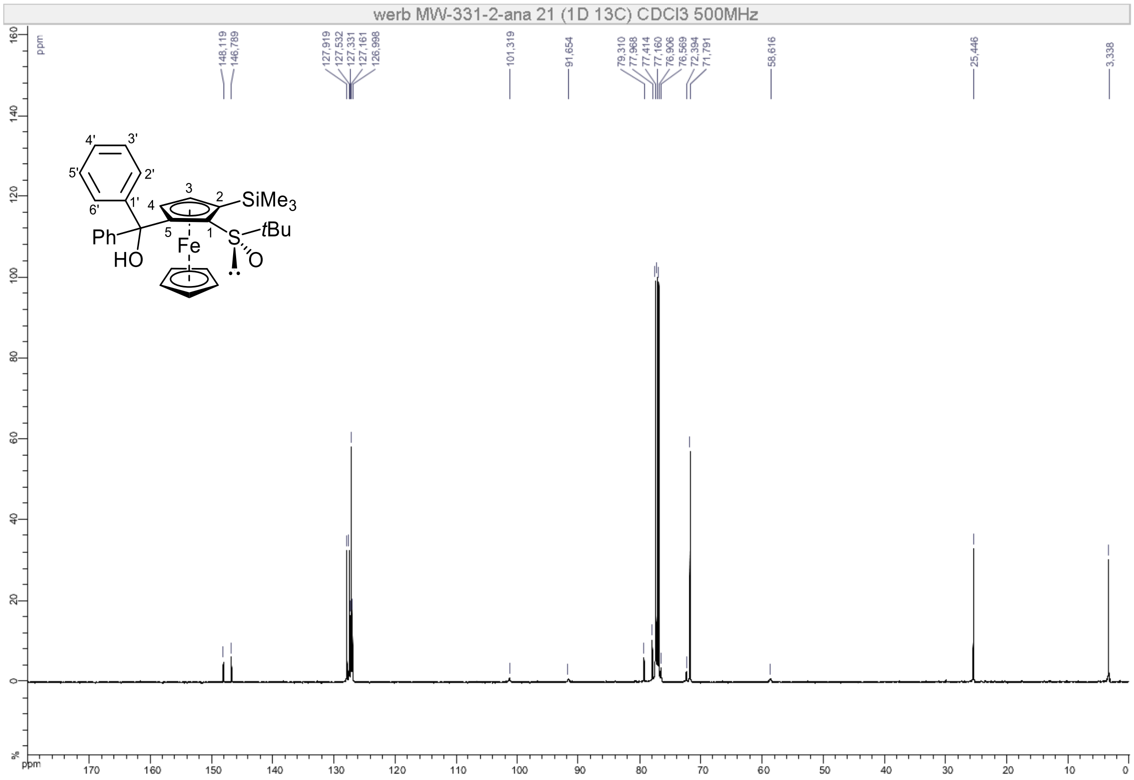

COSY (500 MHz, $CDCl_3$)

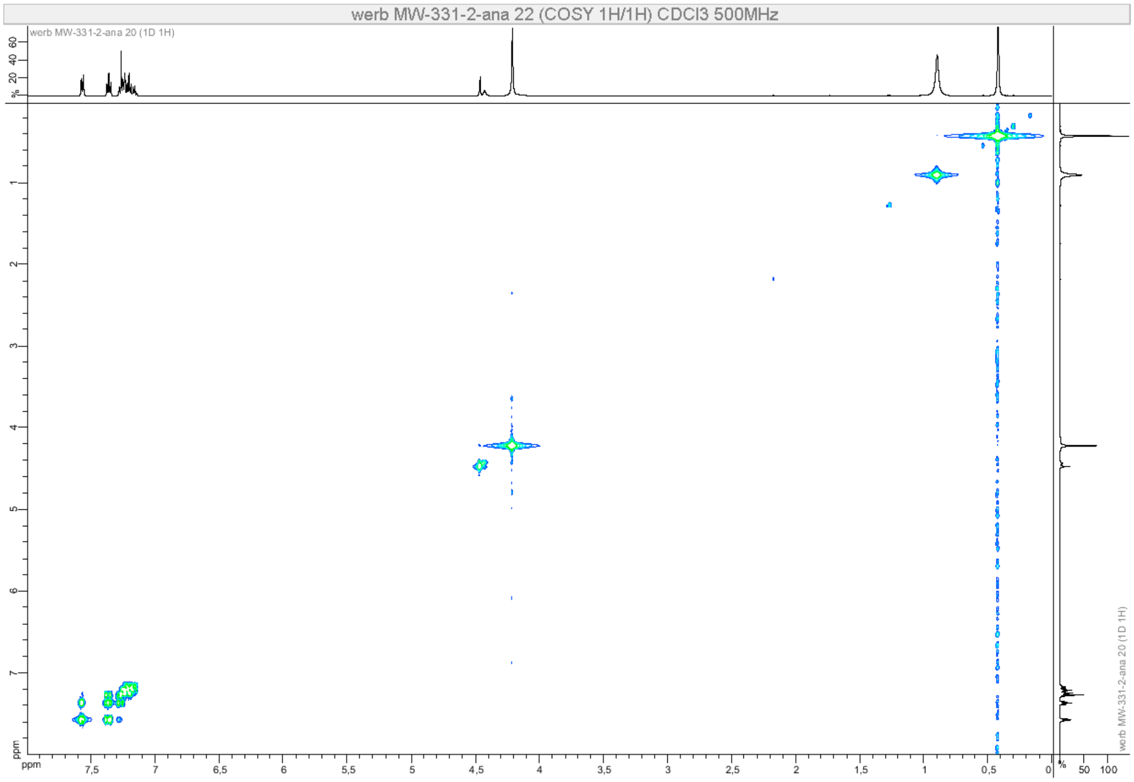

HSQC (500 MHz, $CDCl_3$)

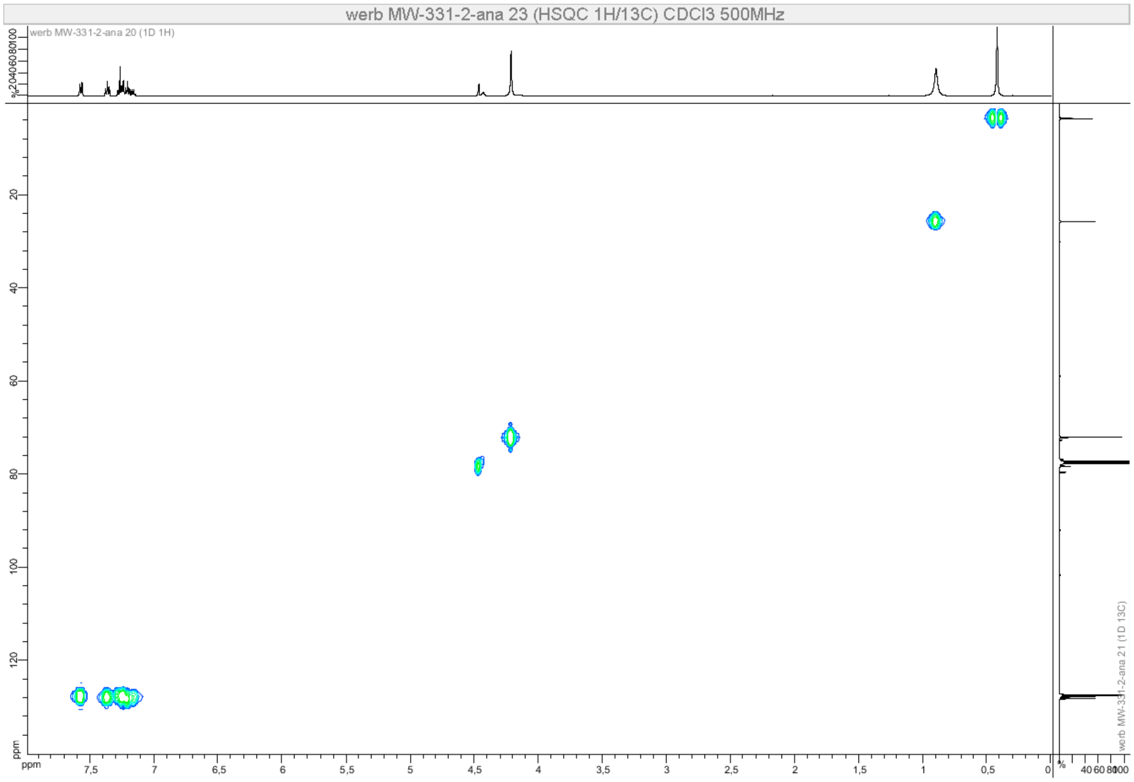

HMBC (500 MHz, $CDCl_3$)

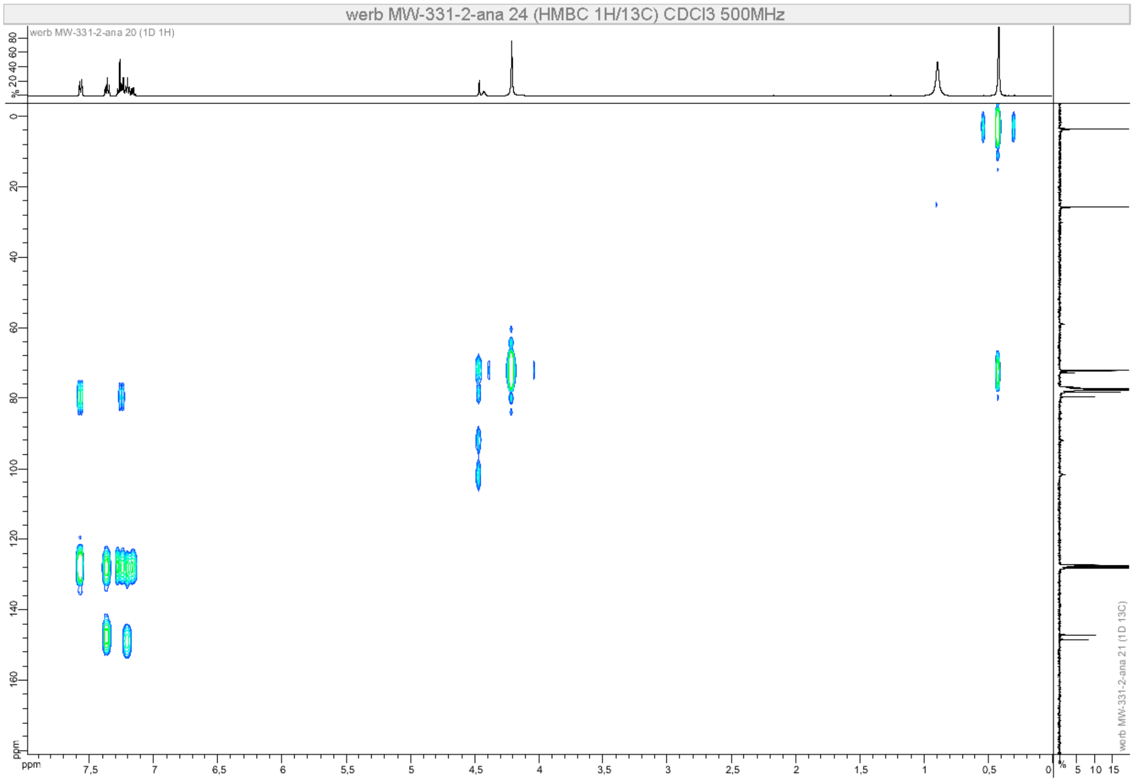

NOESY (500 MHz, $CDCl_3$)

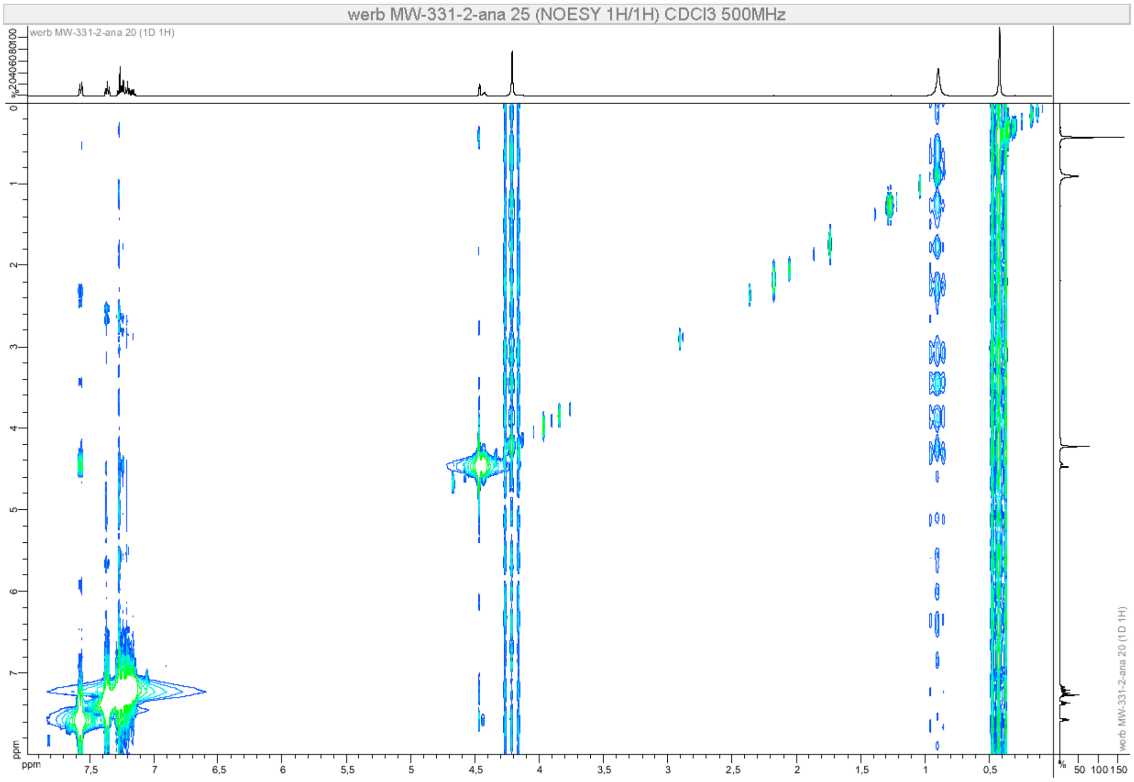

(*S*,*S*P)-*S*-*tert*-Butyl-2-(diphenylphosphino)-5-(trimethylsilyl)ferrocenesulfoxide (*S*,*S*P-2ea)

^{1}H NMR (500 MHz, $CDCl_3$)

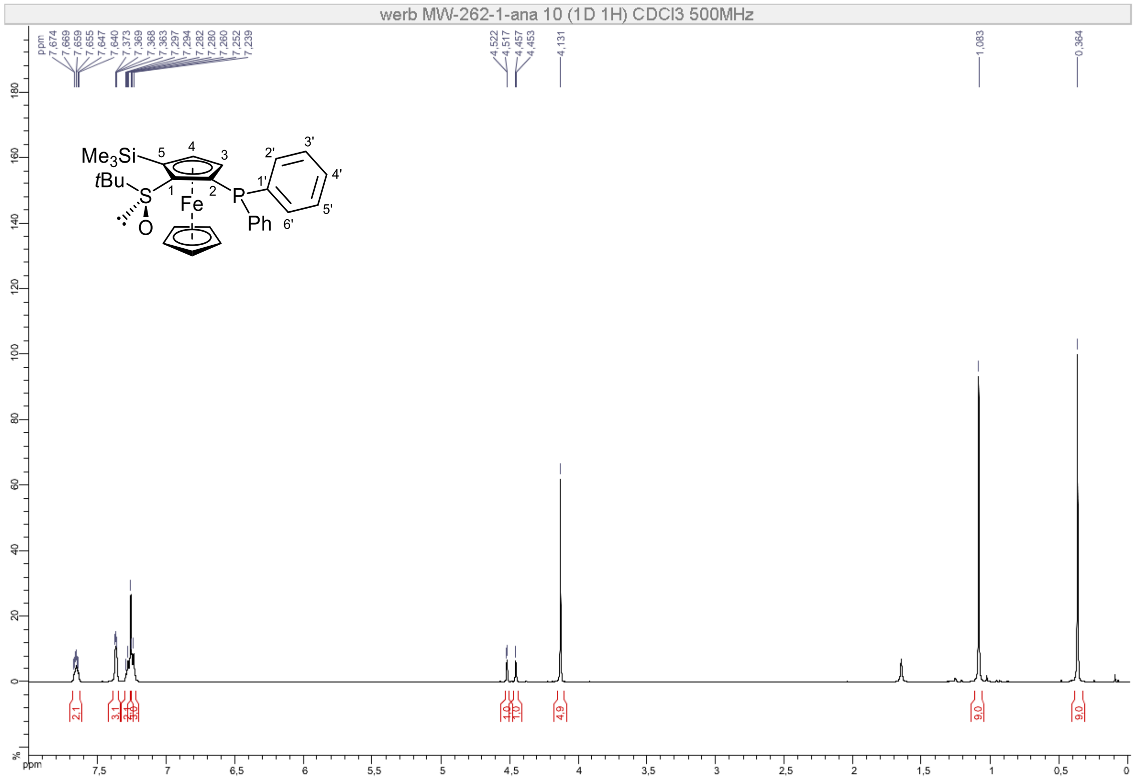

^{13}C NMR (126 MHz, $CDCl_3$)

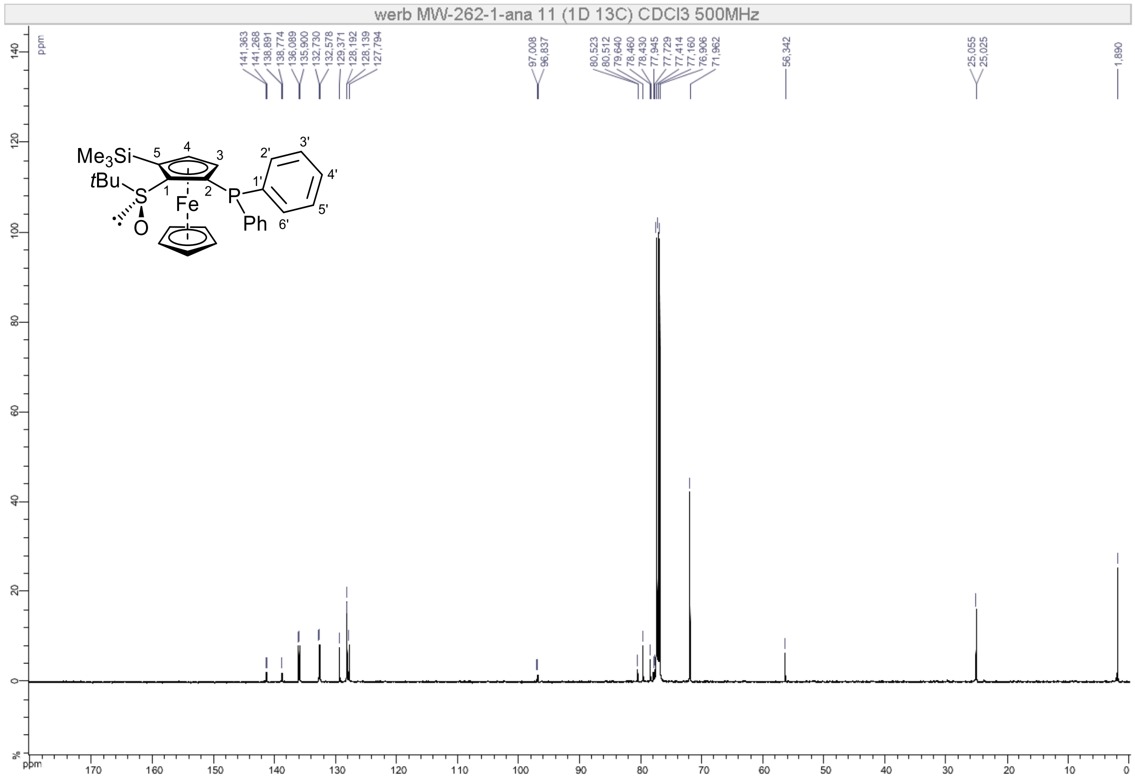

DEPT 135 (126 MHz, $CDCl_3$)

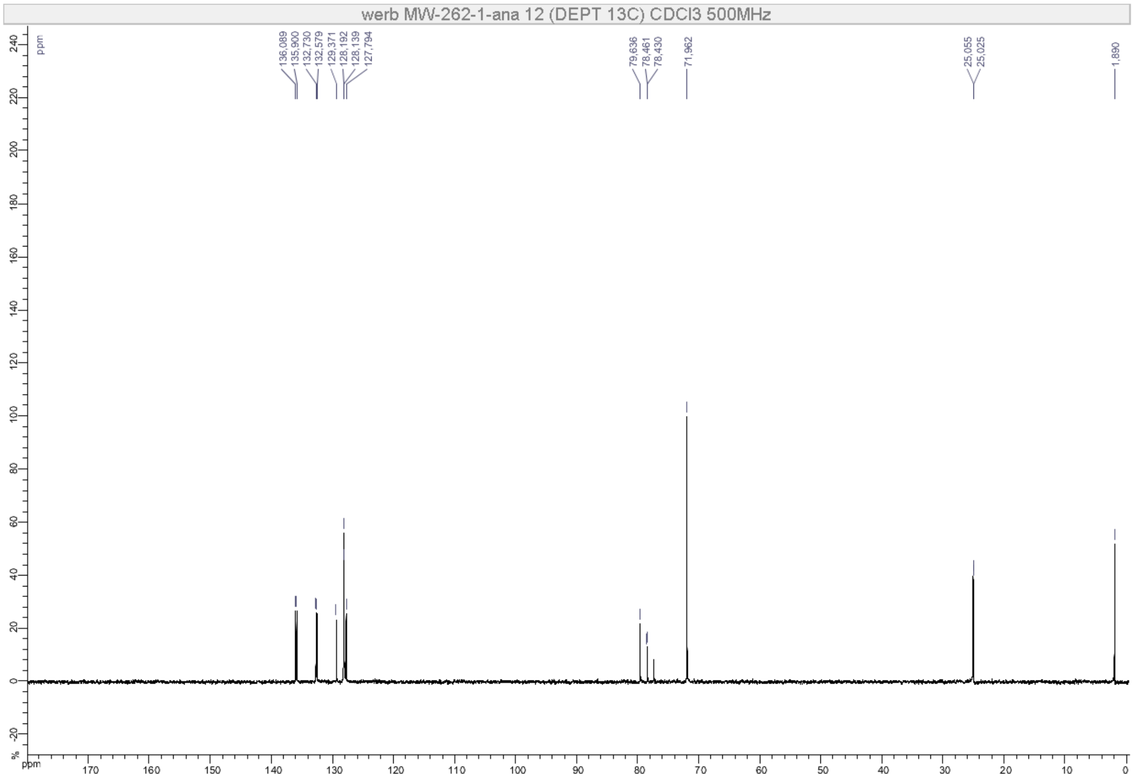

COSY (500 MHz, $CDCl_3$)

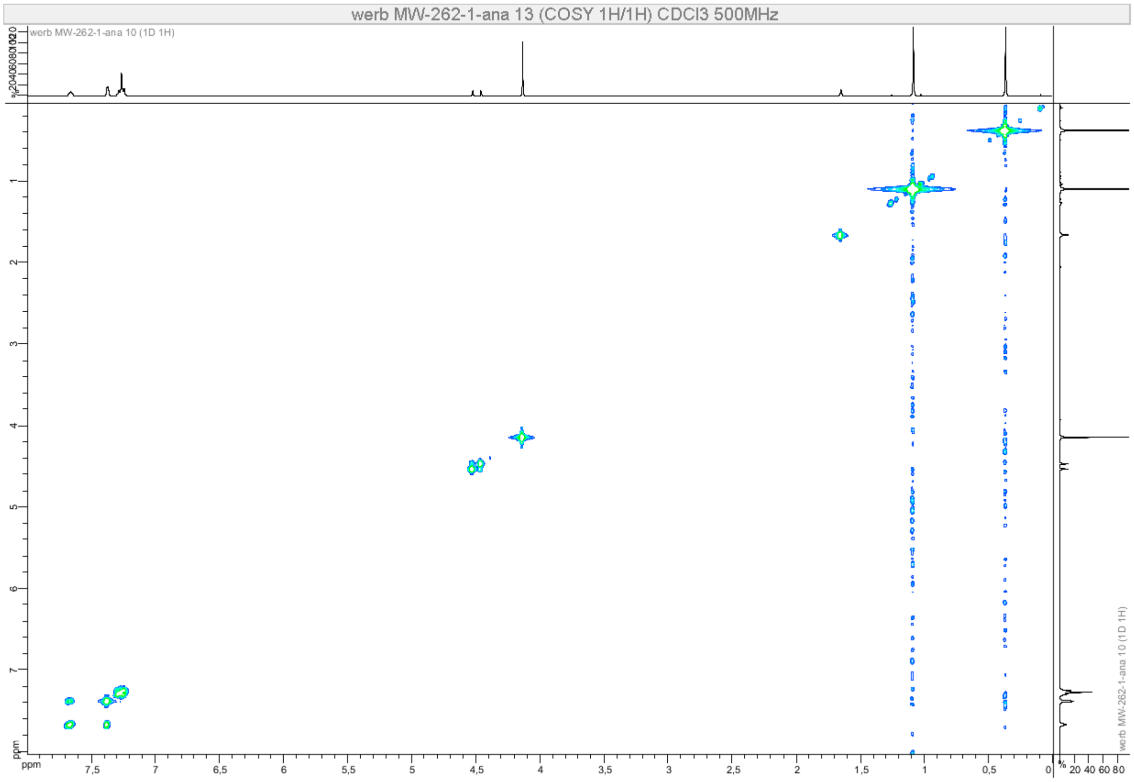

HSQC (500 MHz, $CDCl_3$)

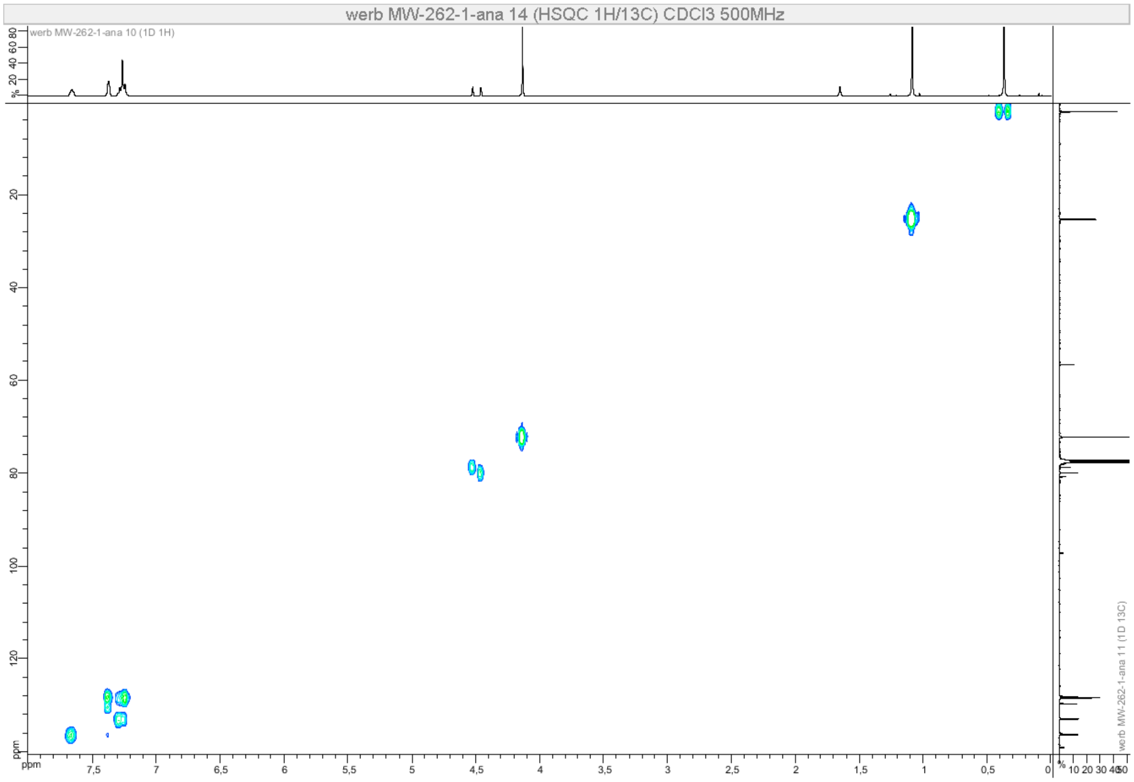

HMBC (500 MHz, $CDCl_3$)

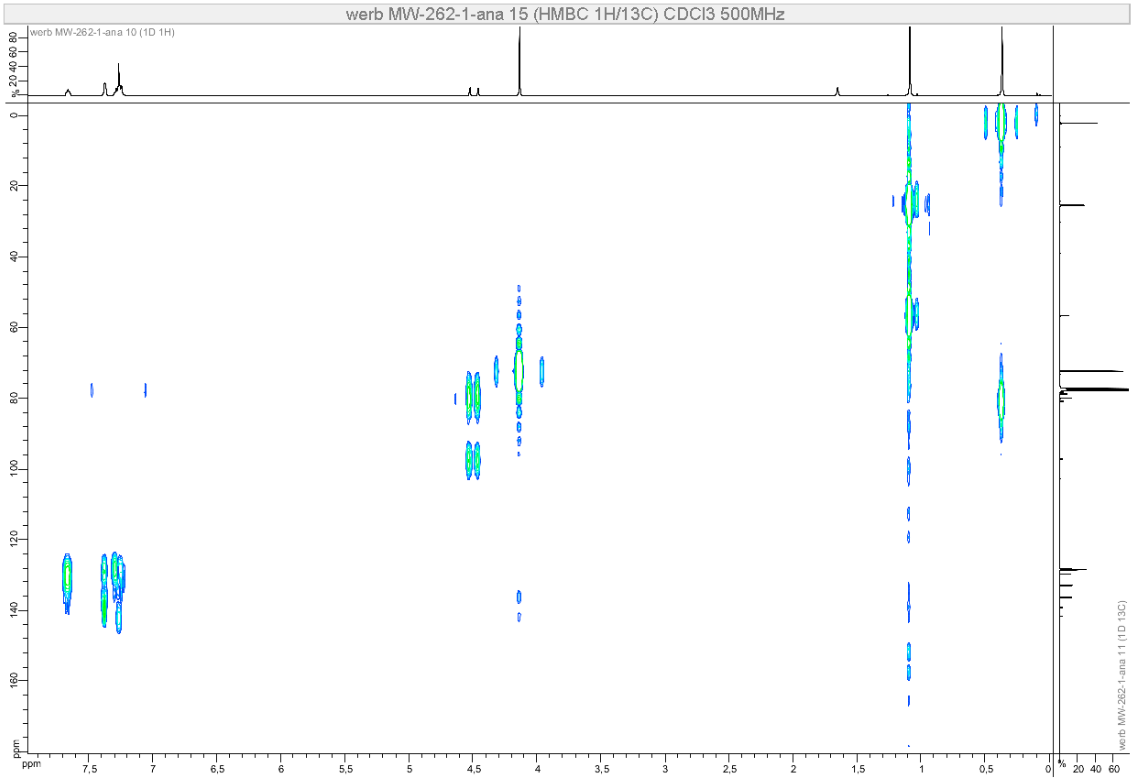

NOESY (500 MHz, $CDCl_3$)

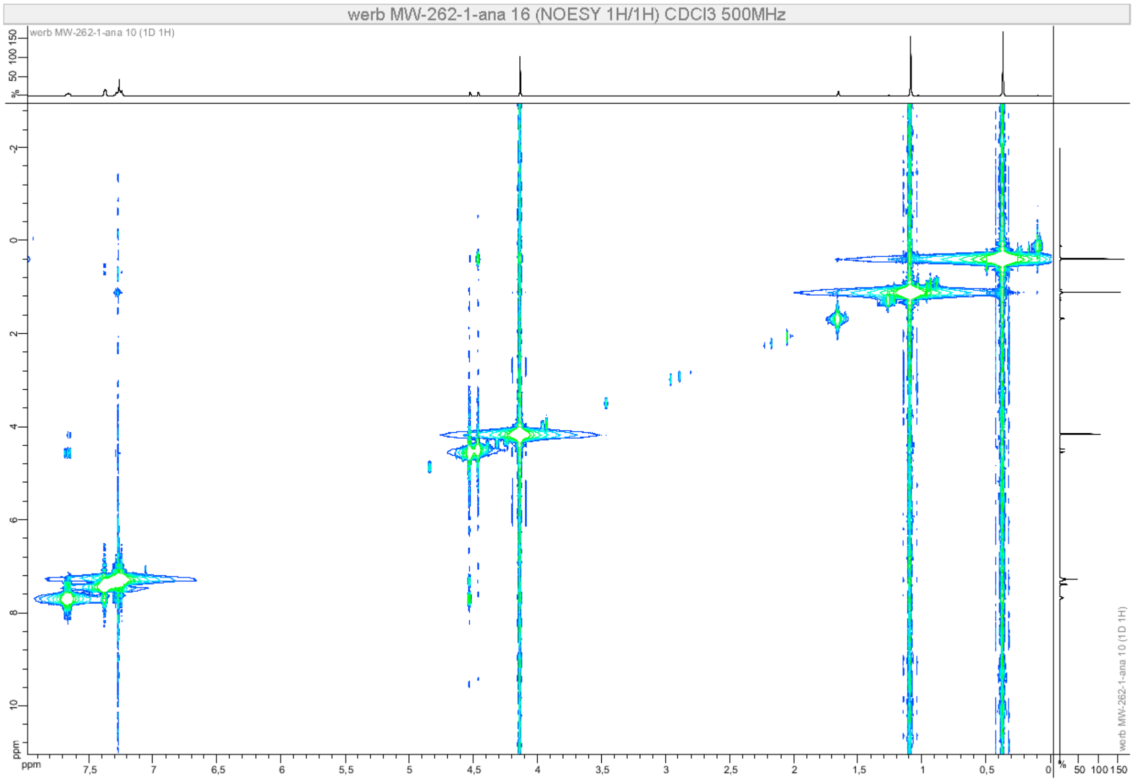

^{31}P NMR (202 MHz, $CDCl_3$)

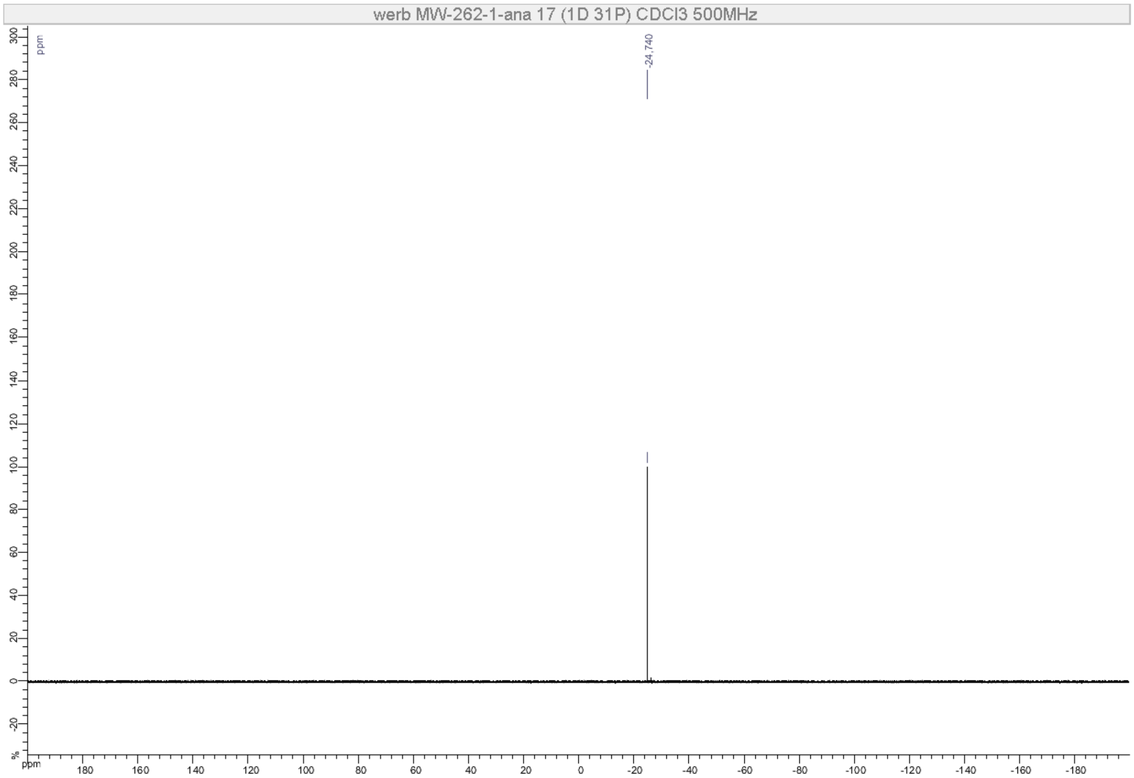

(*S*,*S*P)-*S*-*tert*-Butyl-4-iodo-2-(trimethylsilyl)ferrocenesulfoxide (*S*,*S*P-7)

^{1}H NMR (500 MHz, $CDCl_3$)

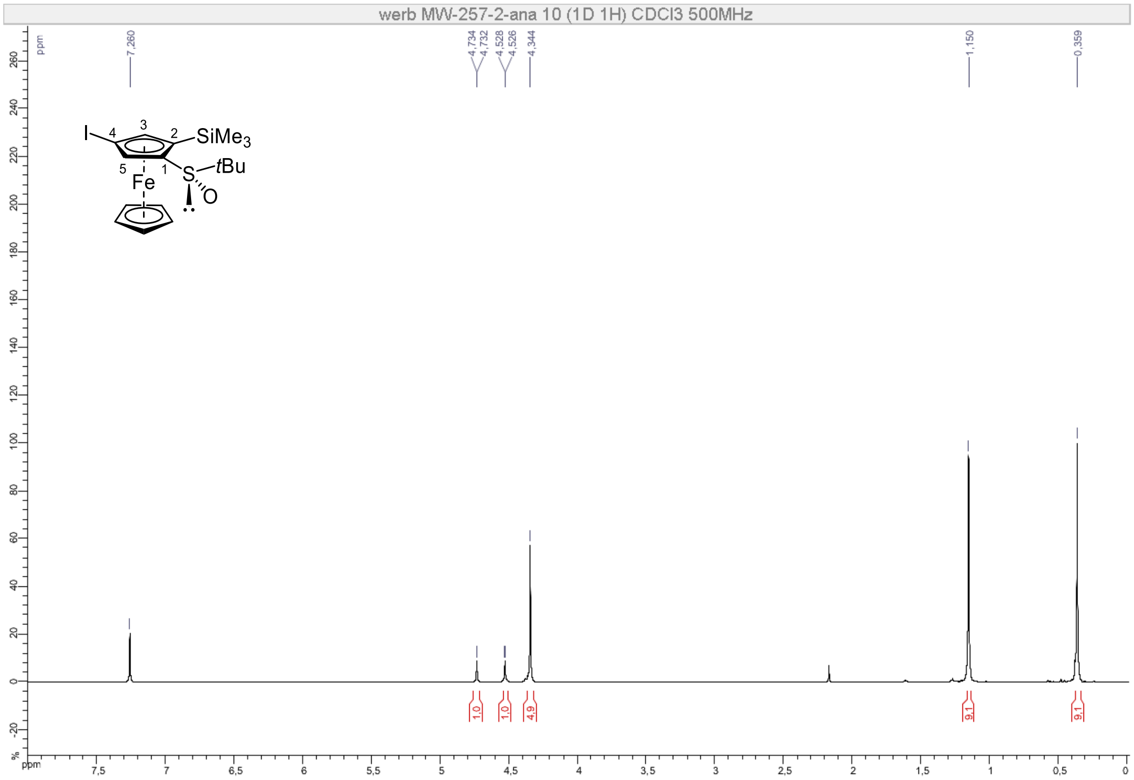

^{13}C NMR (126 MHz, $CDCl_3$)

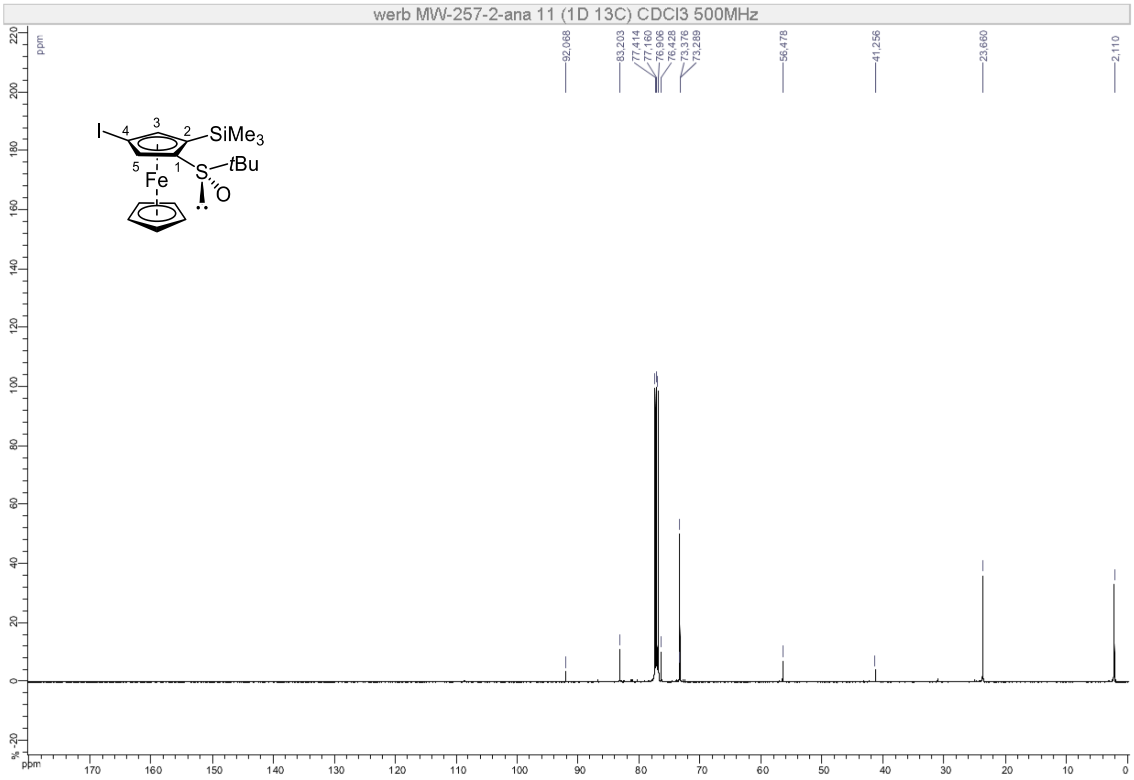

DEPT 135 (126 MHz, $CDCl_3$)

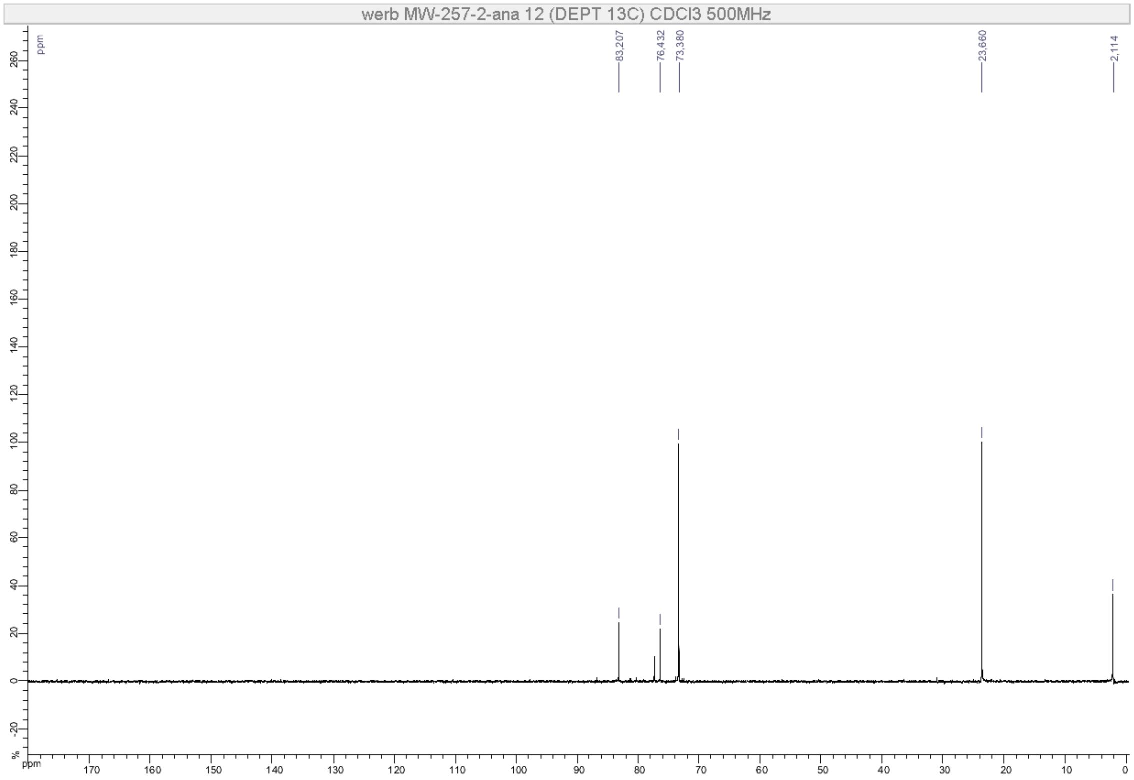

COSY (500 MHz, $CDCl_3$)

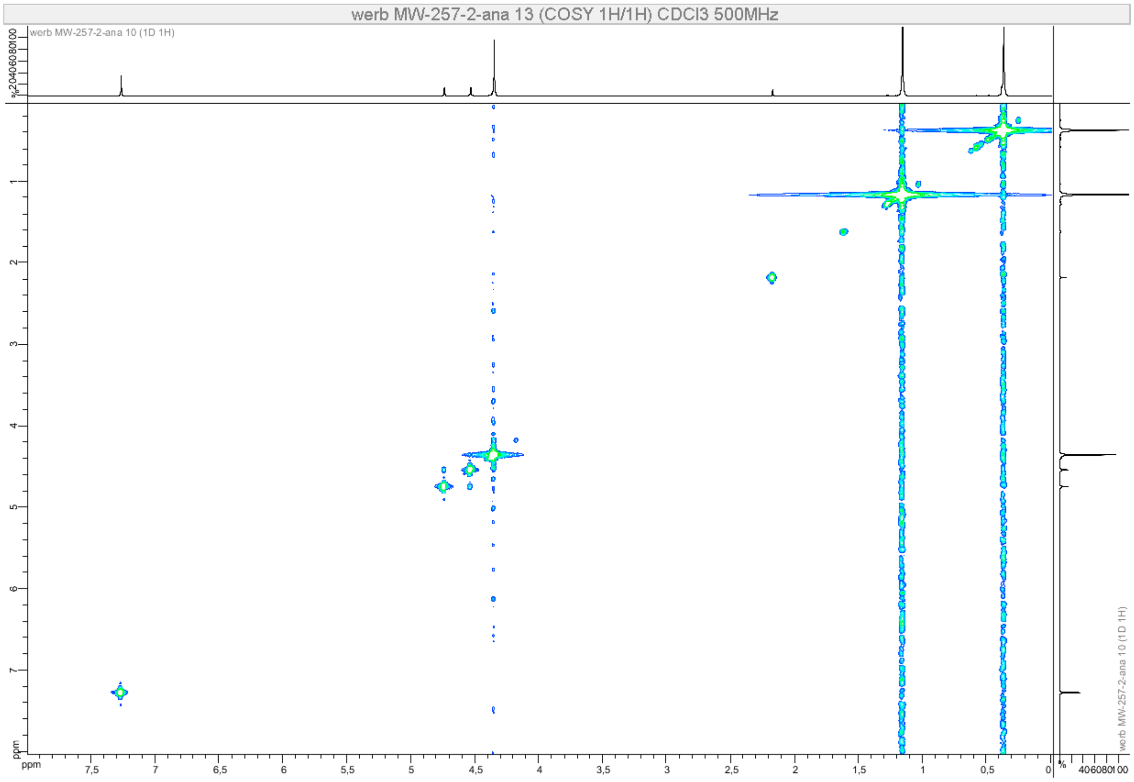

HSQC (500 MHz, $CDCl_3$)

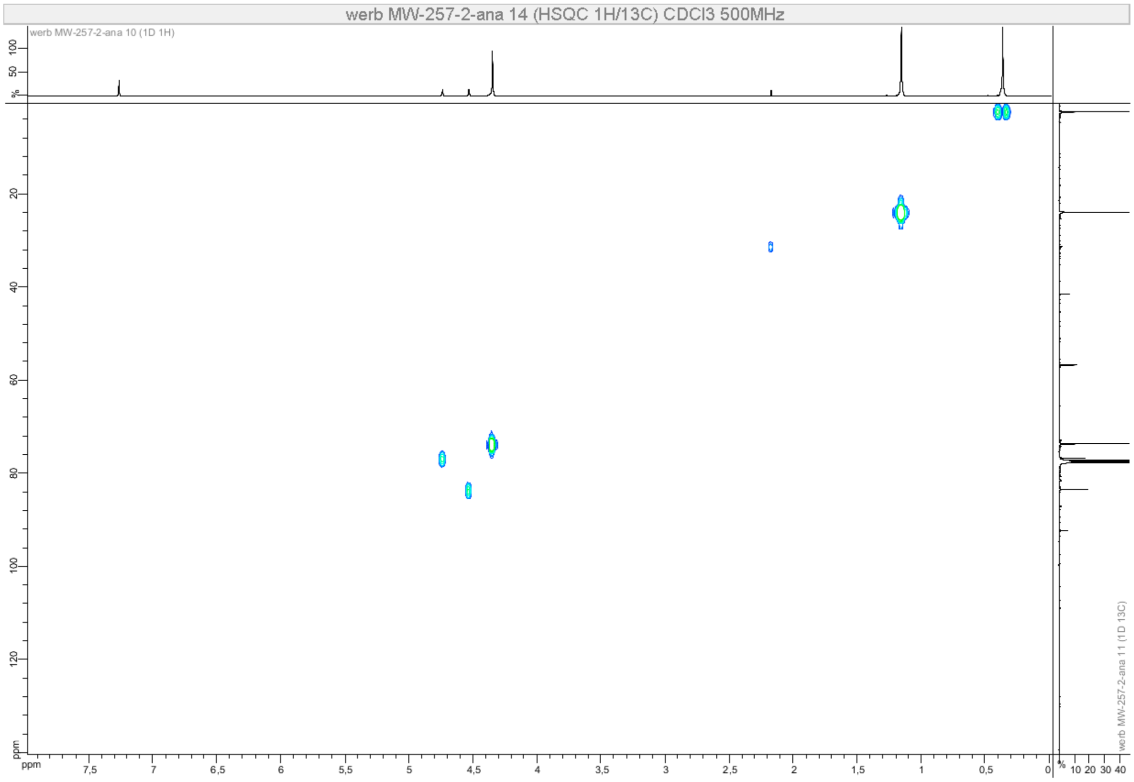

HMBC (500 MHz, $CDCl_3$)

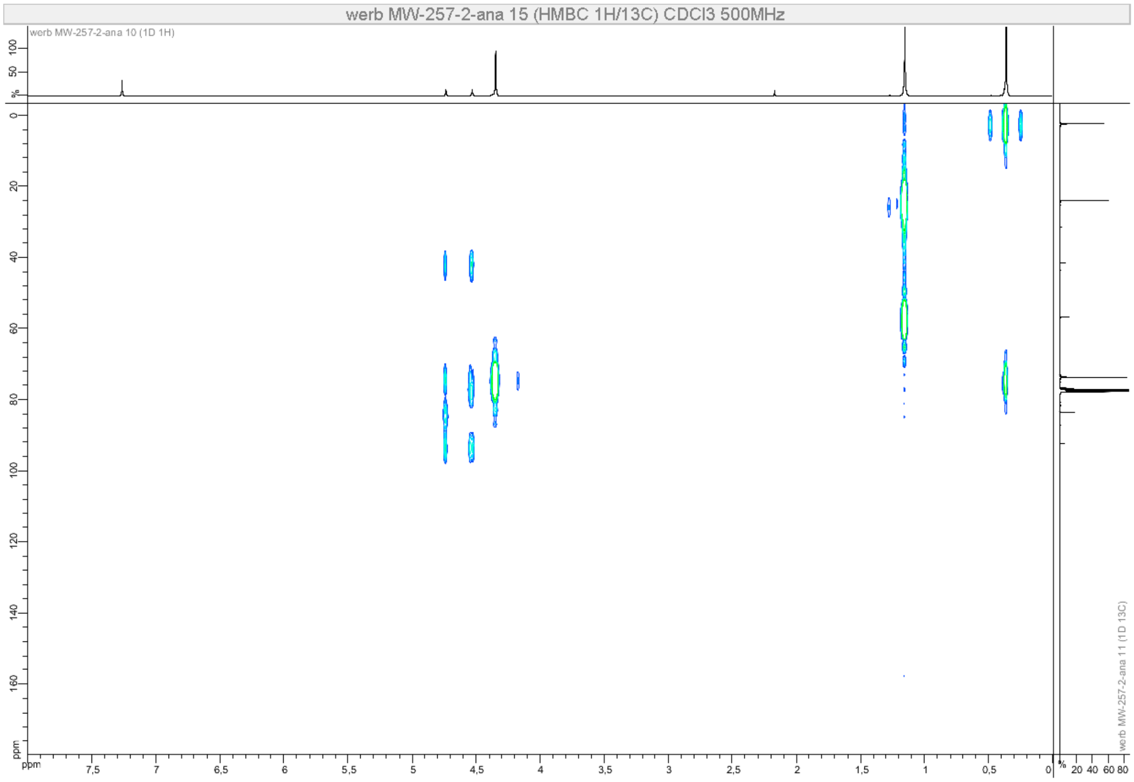

(*S*,*S*P)-*S*-*tert*-Butyl-2-chloro-5-fluoro-4-methyl-3-(trimethylsilyl)ferrocenesulfoxide (*S*,*S*P-12)

^{1}H NMR (500 MHz, $CDCl_3$)

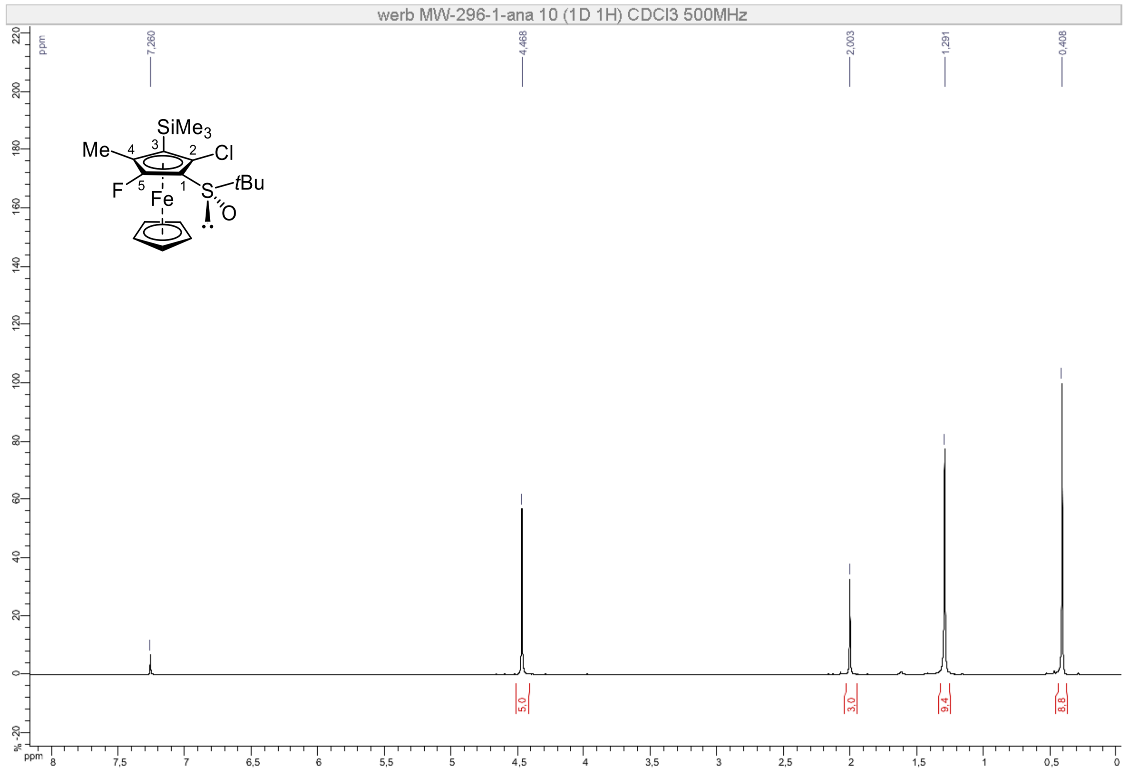

^{13}C NMR (126 MHz, $CDCl_3$)

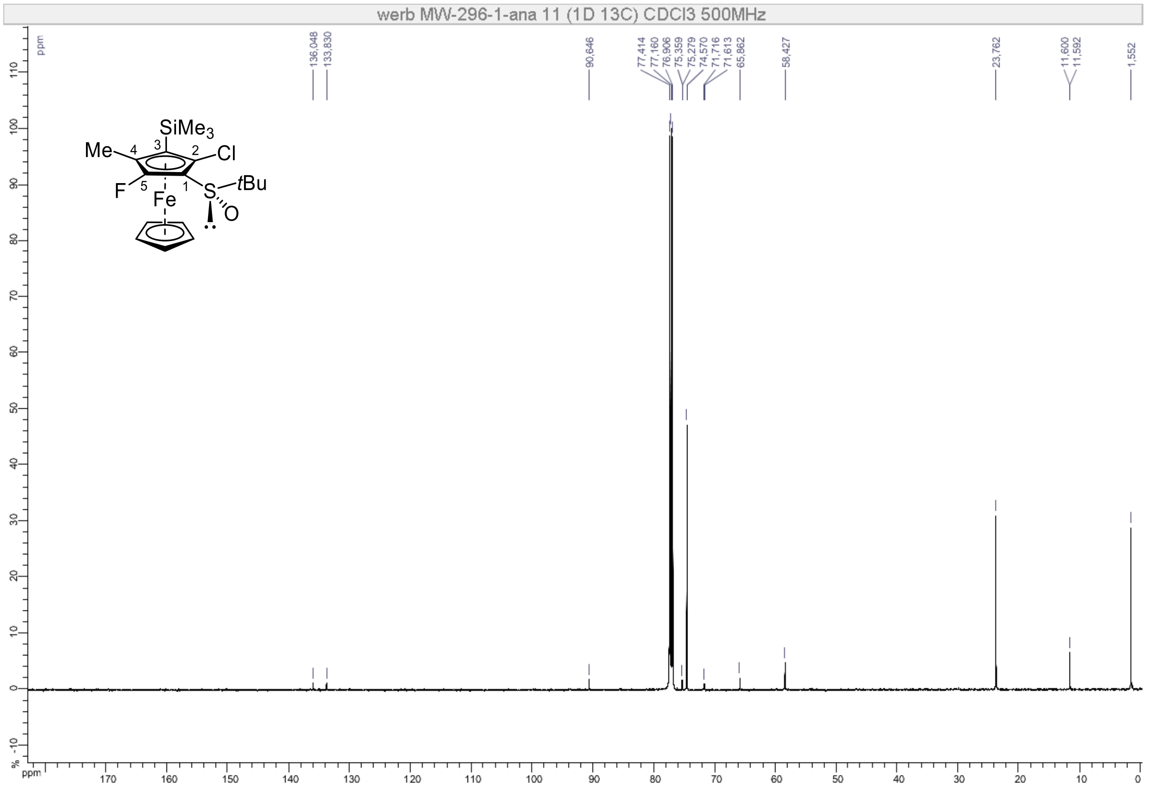

DEPT 135 (126 MHz, $CDCl_3$)

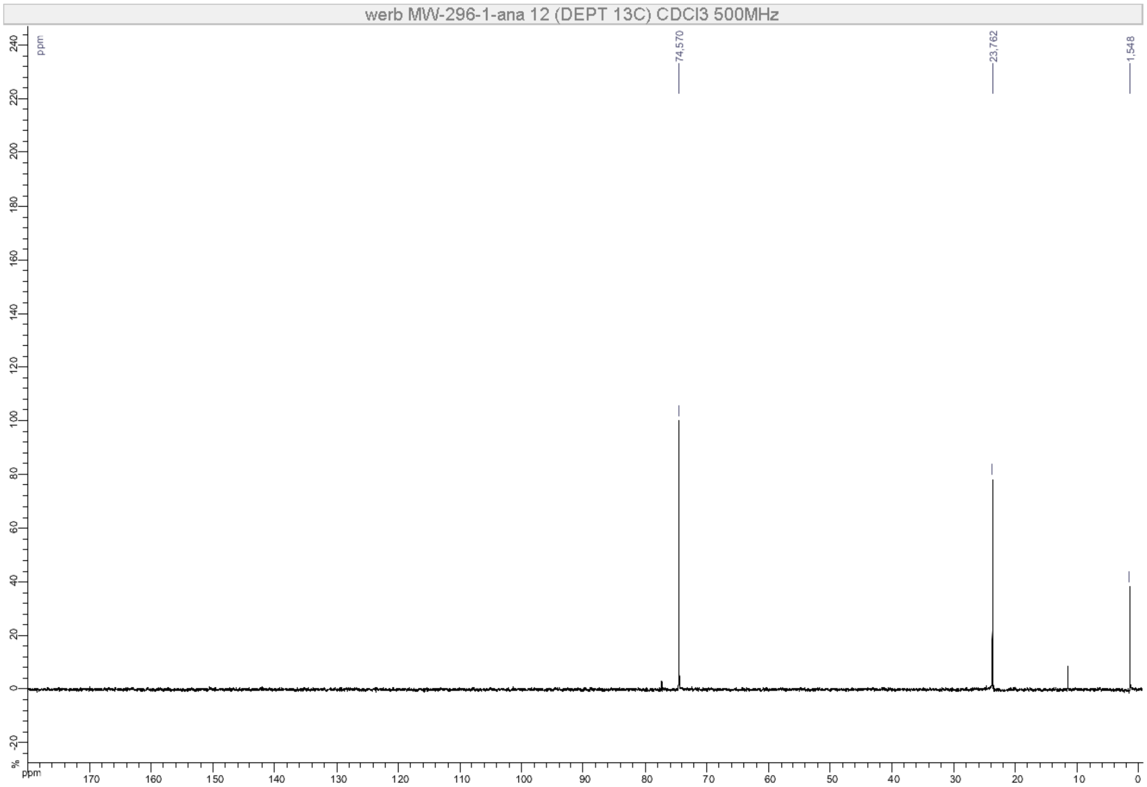

COSY (500 MHz, $CDCl_3$)

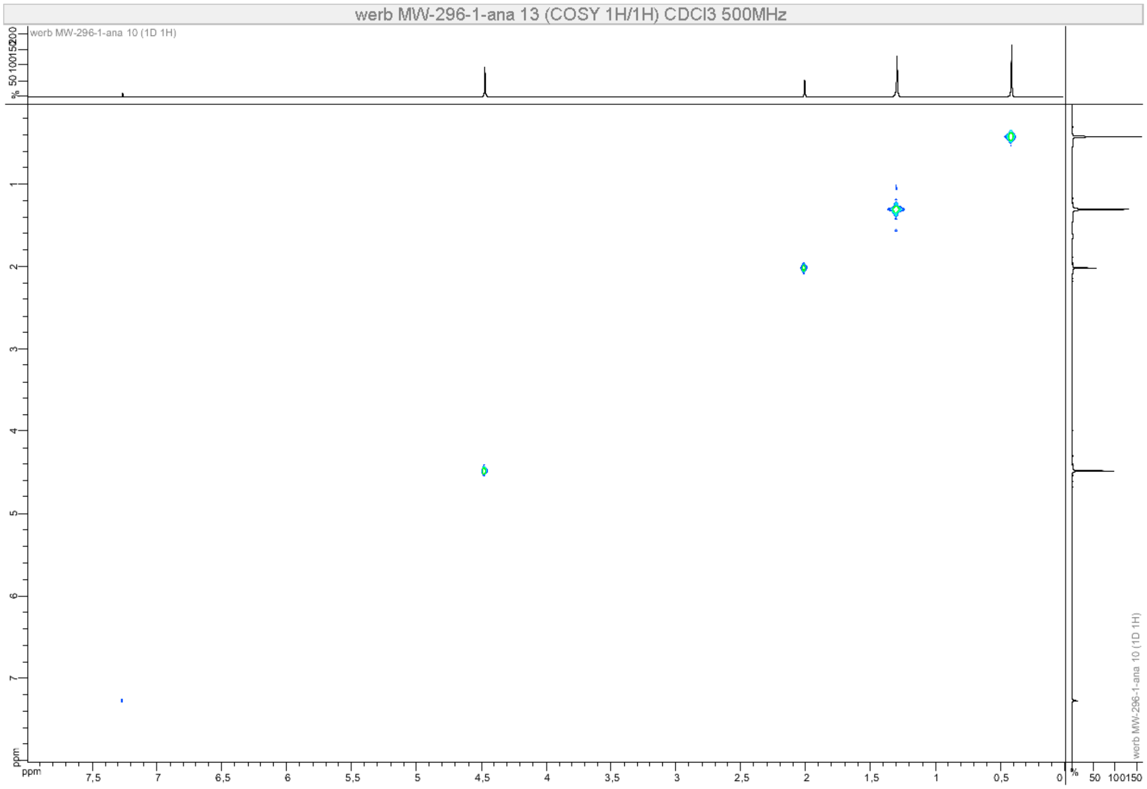

HSQC (500 MHz, $CDCl_3$)

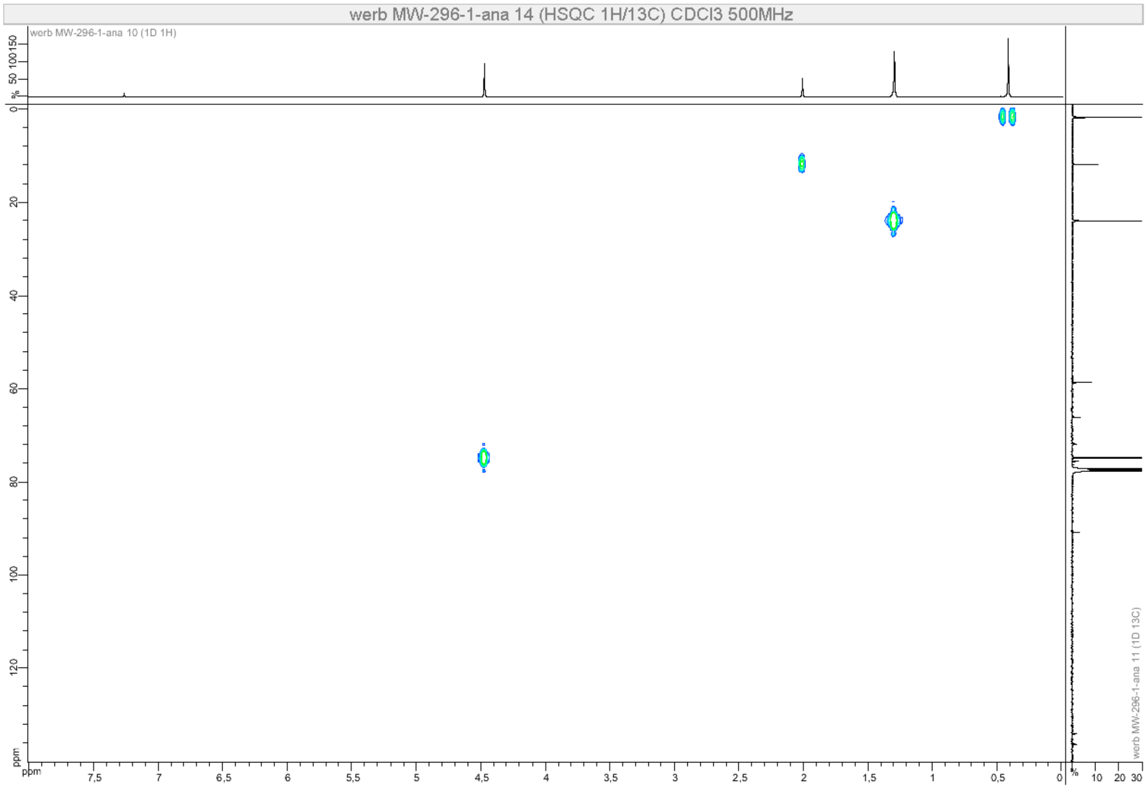

HMBC (500 MHz, $CDCl_3$)

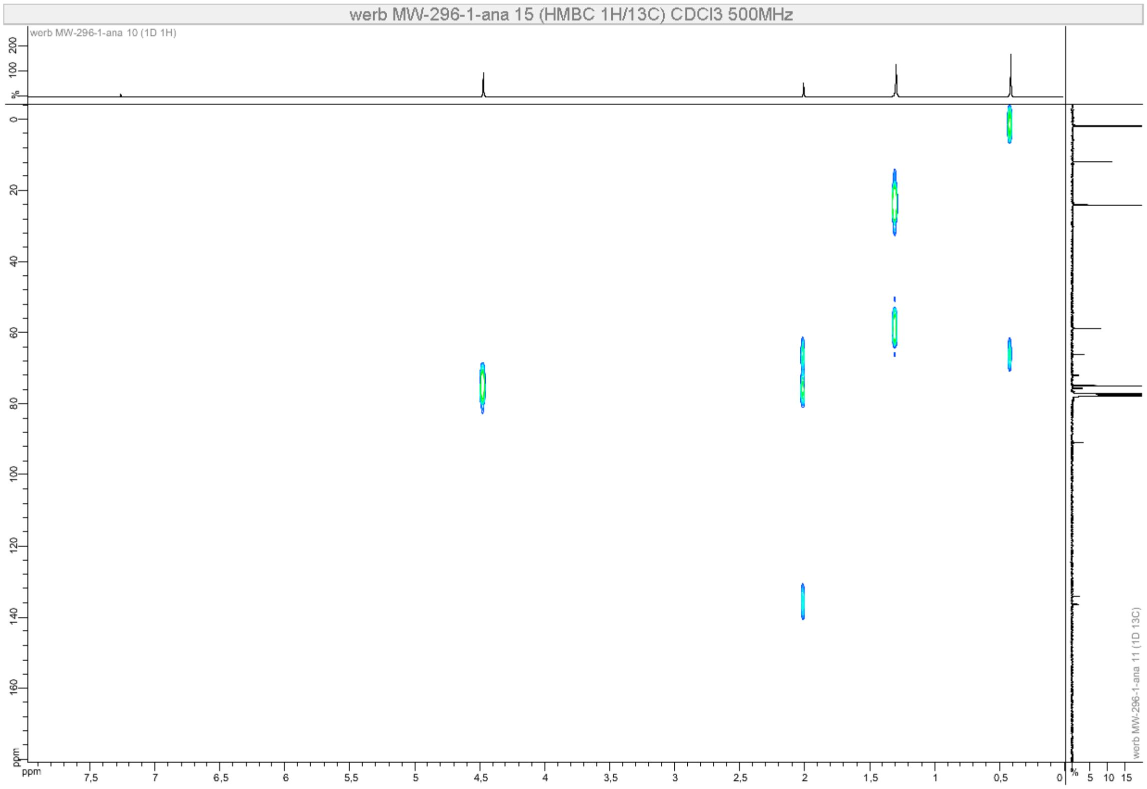

NOESY (500 MHz, $CDCl_3$)

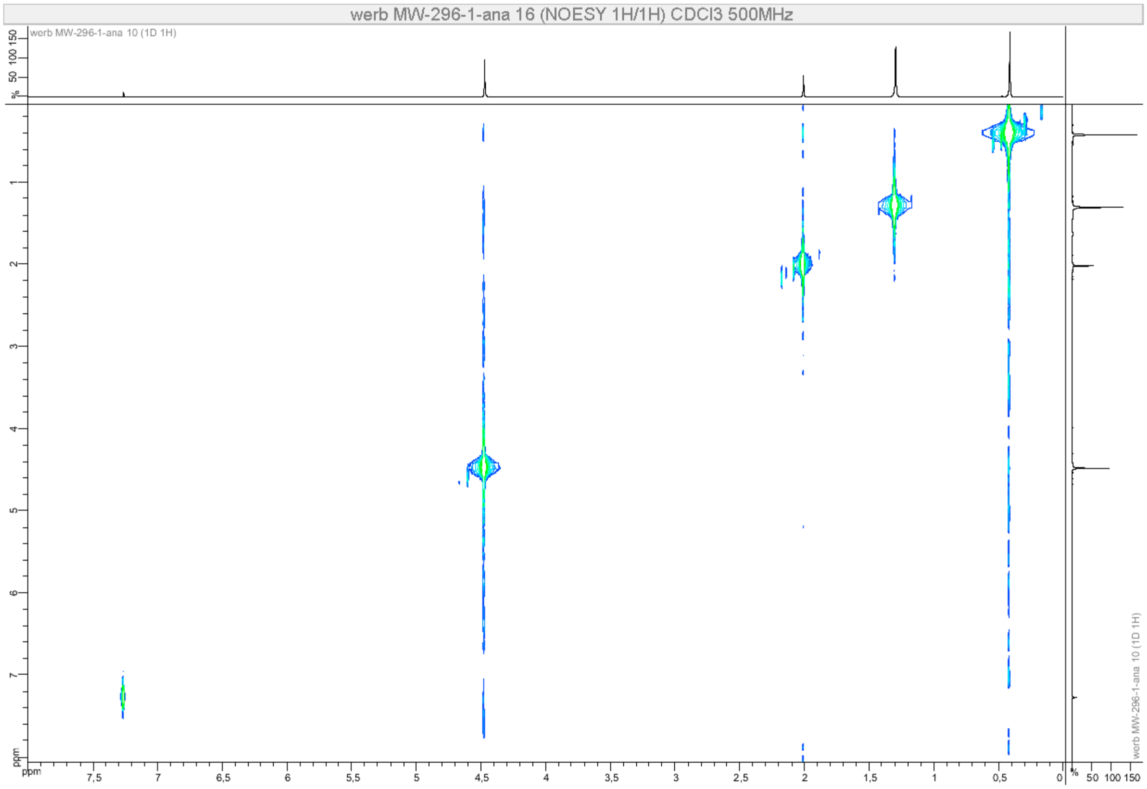

^{19}F NMR (471 MHz, $CDCl_3$)

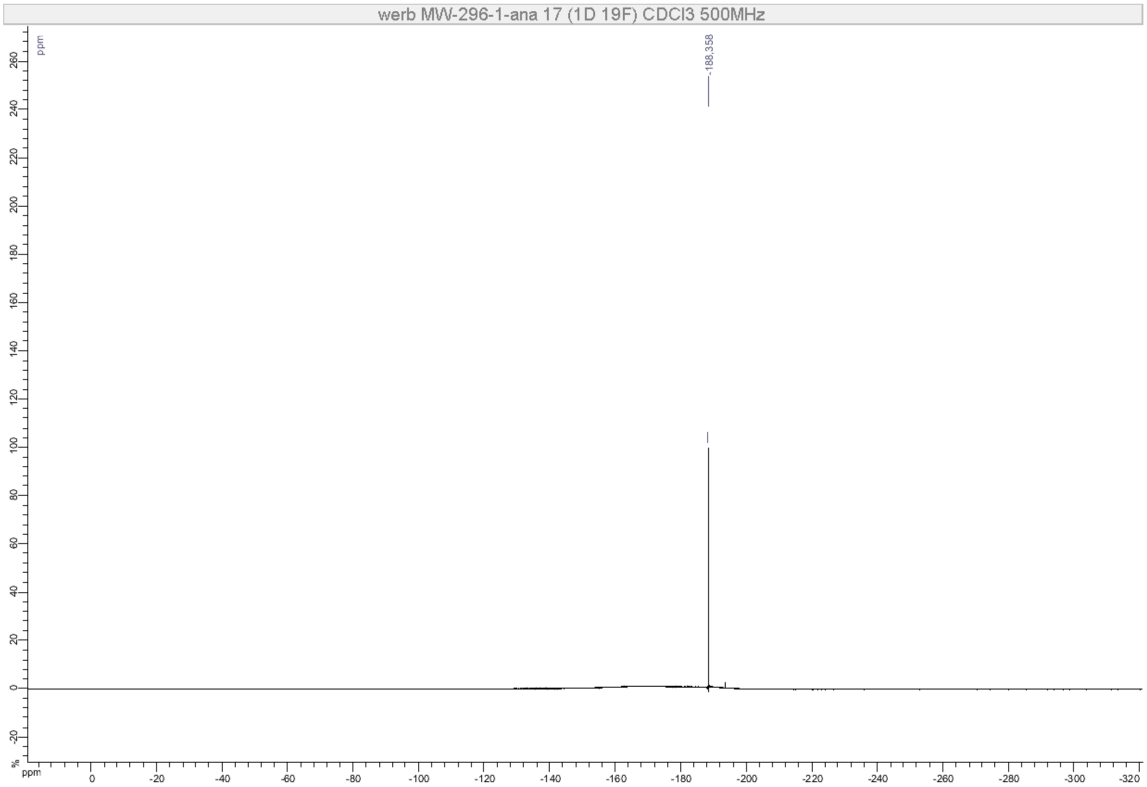

(*R*,*R*P)- and (*S*,*S*P)-*S*-(4-Tolyl)-2-(trimethylsilyl)ferrocenesulfoxide (*R*,*R*P/*S*,*S*P-3a)

^{1}H NMR (500 MHz, $CDCl_3$)

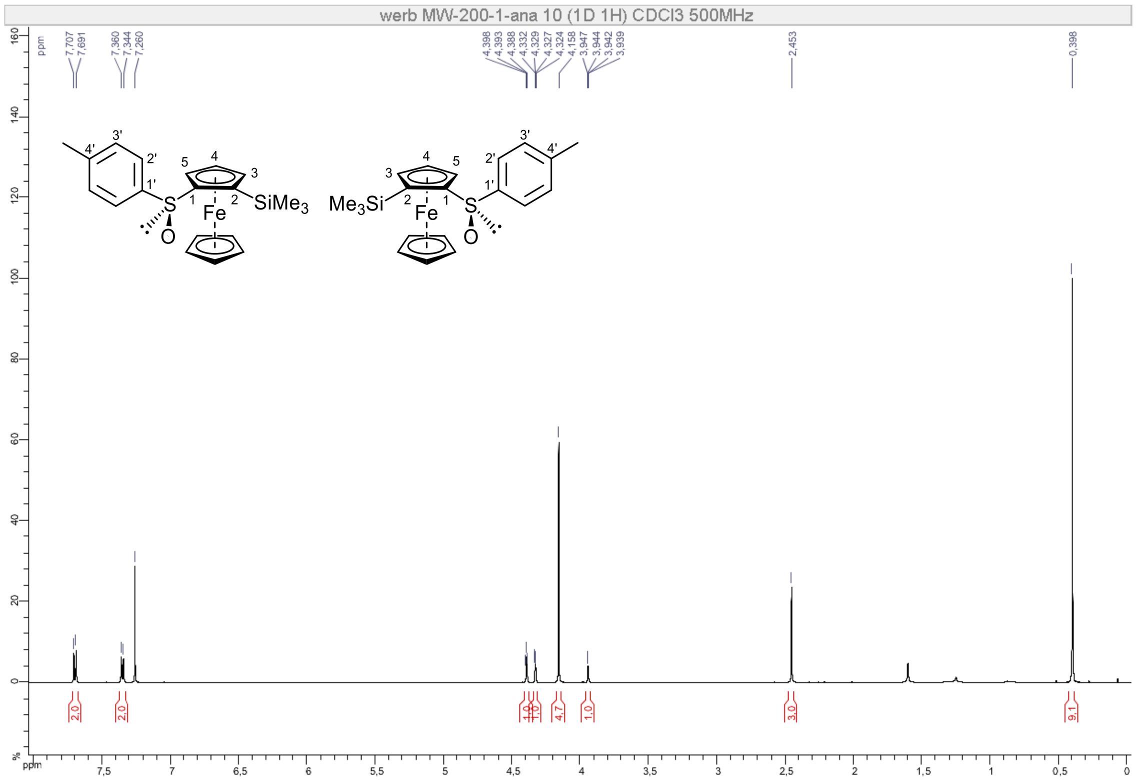

^{13}C NMR (126 MHz, $CDCl_3$)

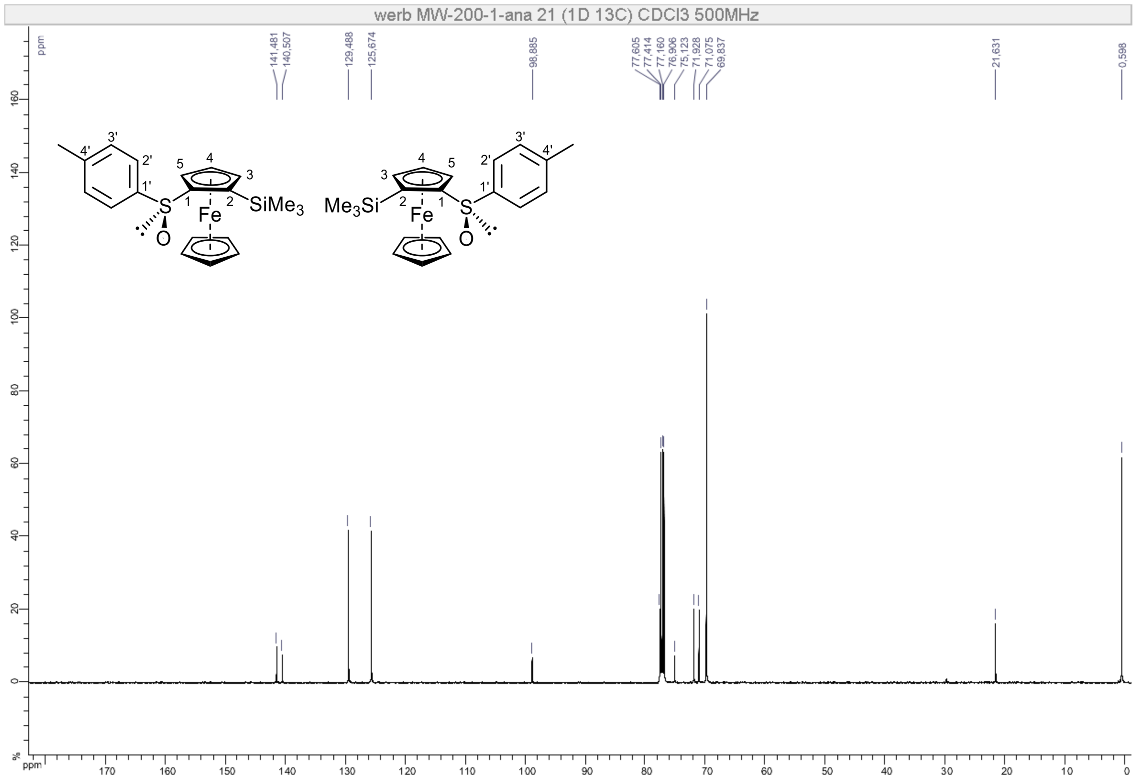

COSY (500 MHz, $CDCl_3$)

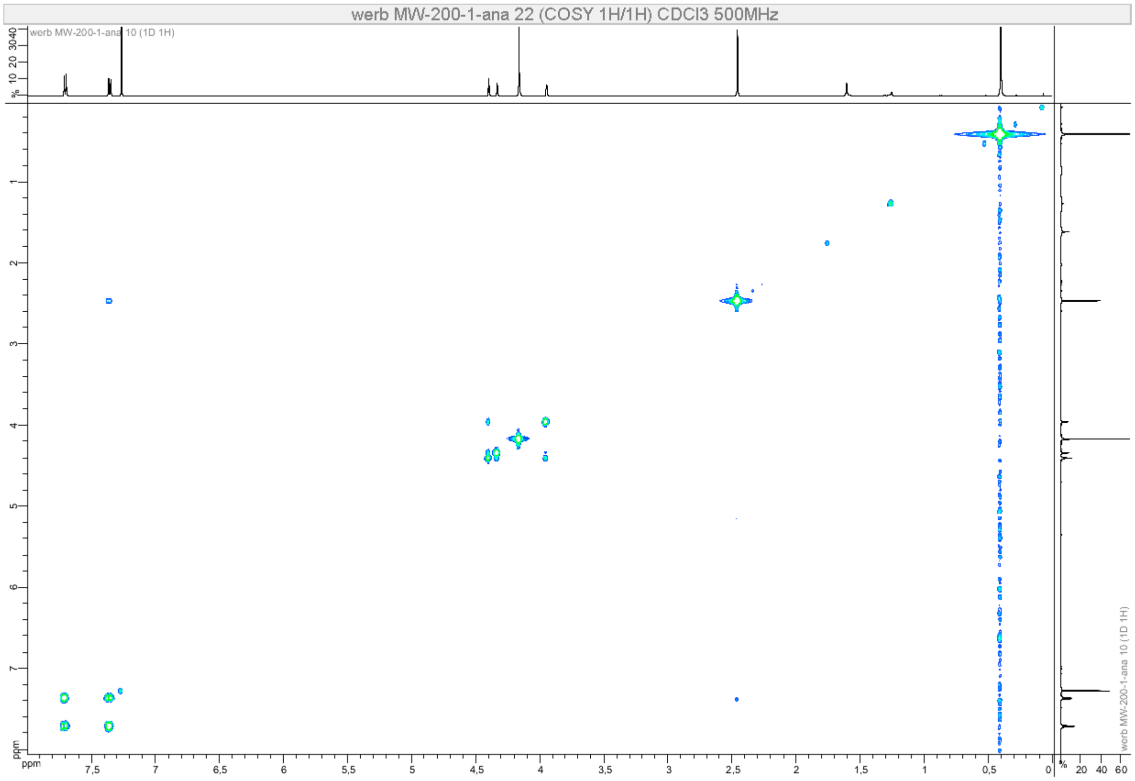

HSQC (500 MHz, $CDCl_3$)

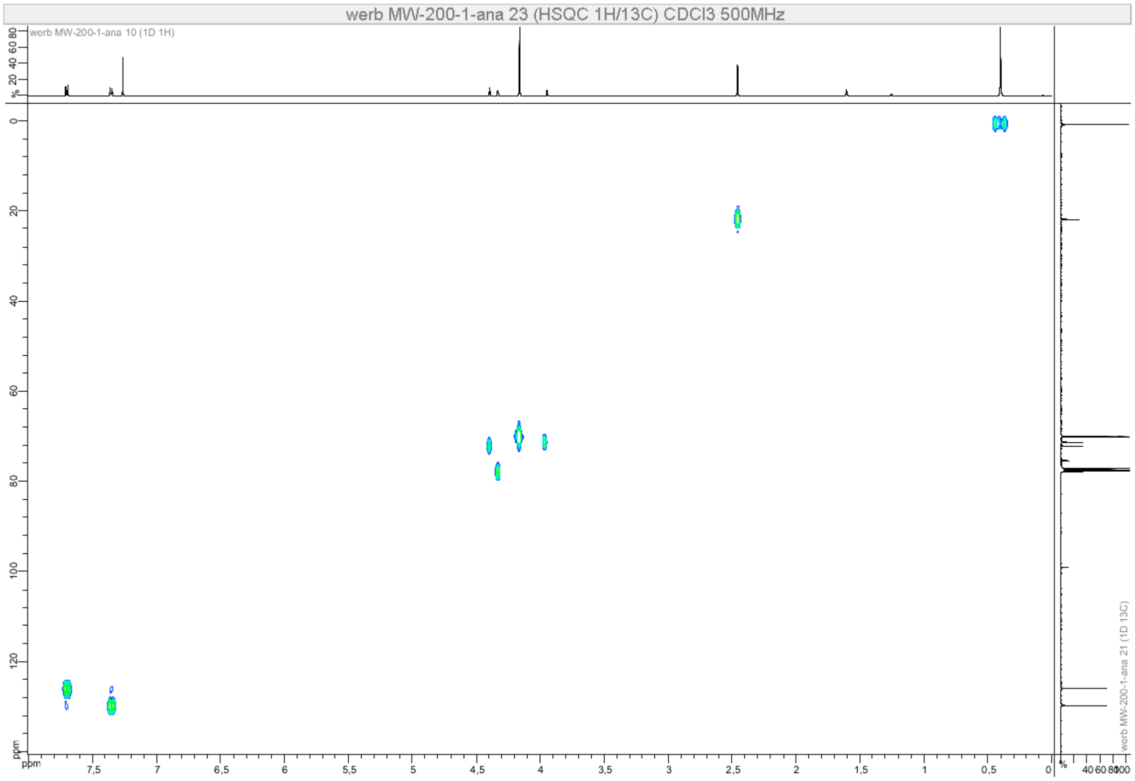

HMBC (500 MHz, $CDCl_3$)

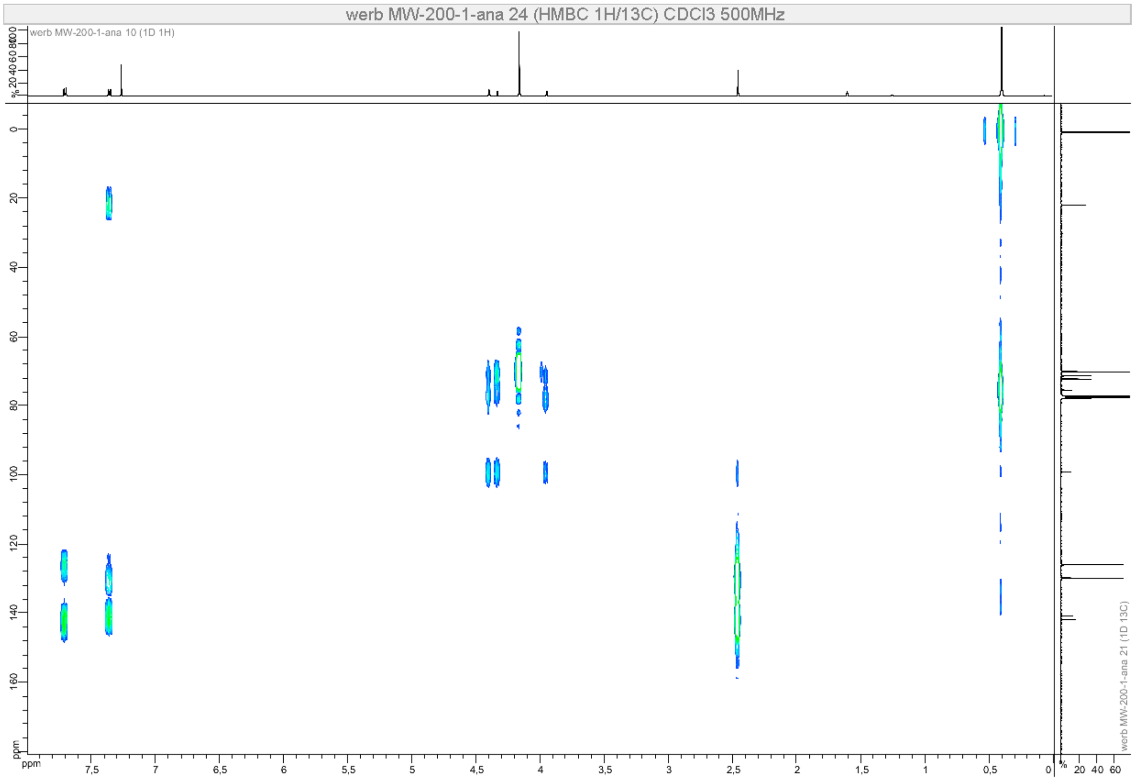

NOESY (500 MHz, $CDCl_3$)

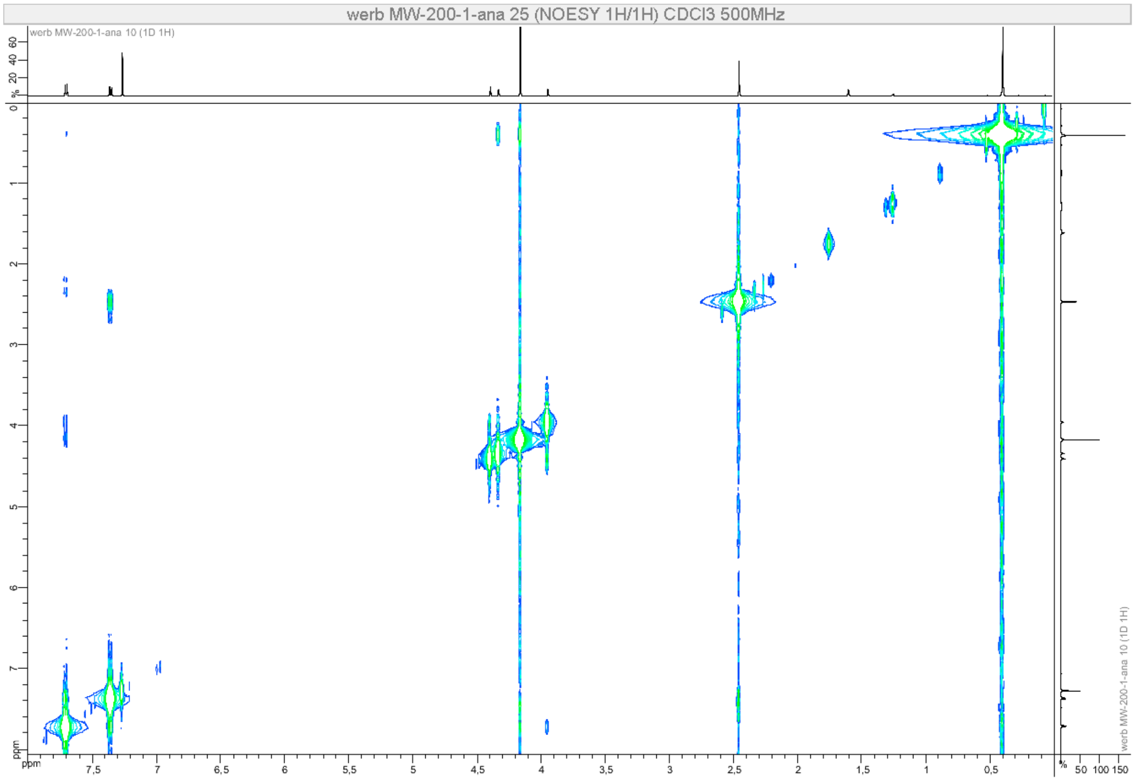

($\boldsymbol{R}$,$\boldsymbol{R}_P$)- and ($\boldsymbol{S}$,$\boldsymbol{S}_P$)-$\boldsymbol{S}$-[4-Methyl-2-(trimethylsilyl)phenyl]-2-(trimethylsilyl)ferrocenesulfoxide ($\boldsymbol{R}$,$\boldsymbol{R}_P$/$\boldsymbol{S}$,$\boldsymbol{S}_P$-3a')

^{1}H NMR (500 MHz, $CDCl_3$)

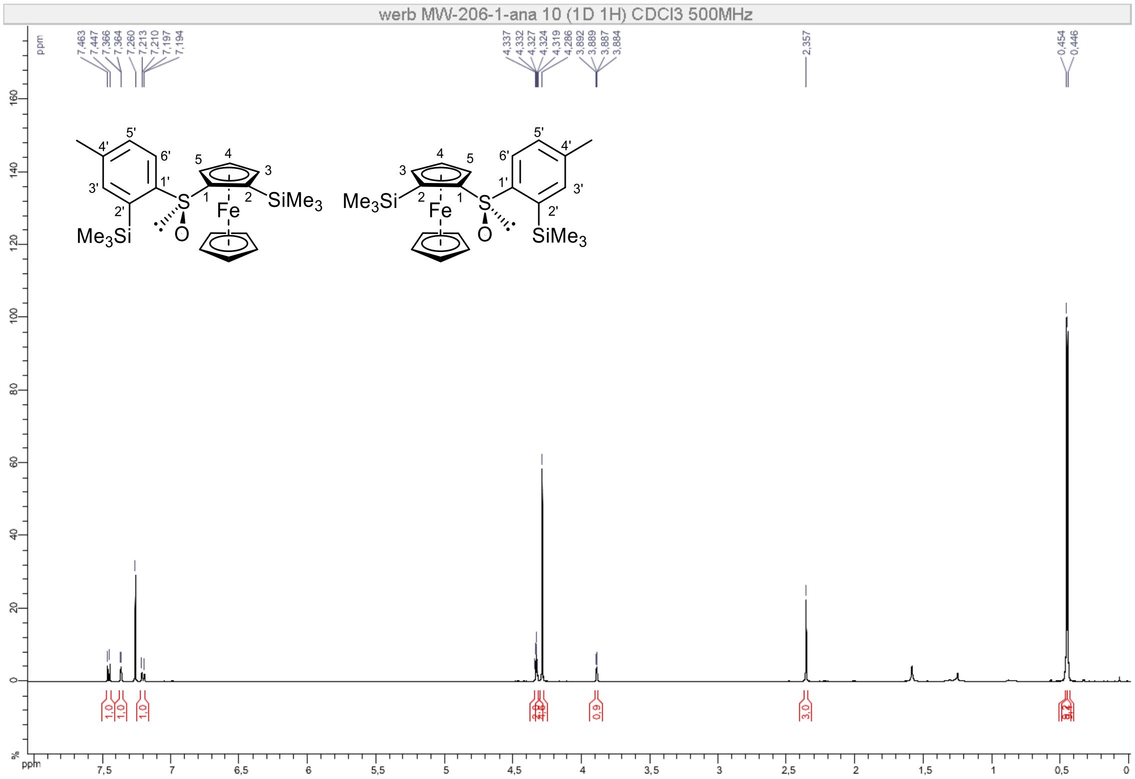

^{13}C NMR (126 MHz, $CDCl_3$)

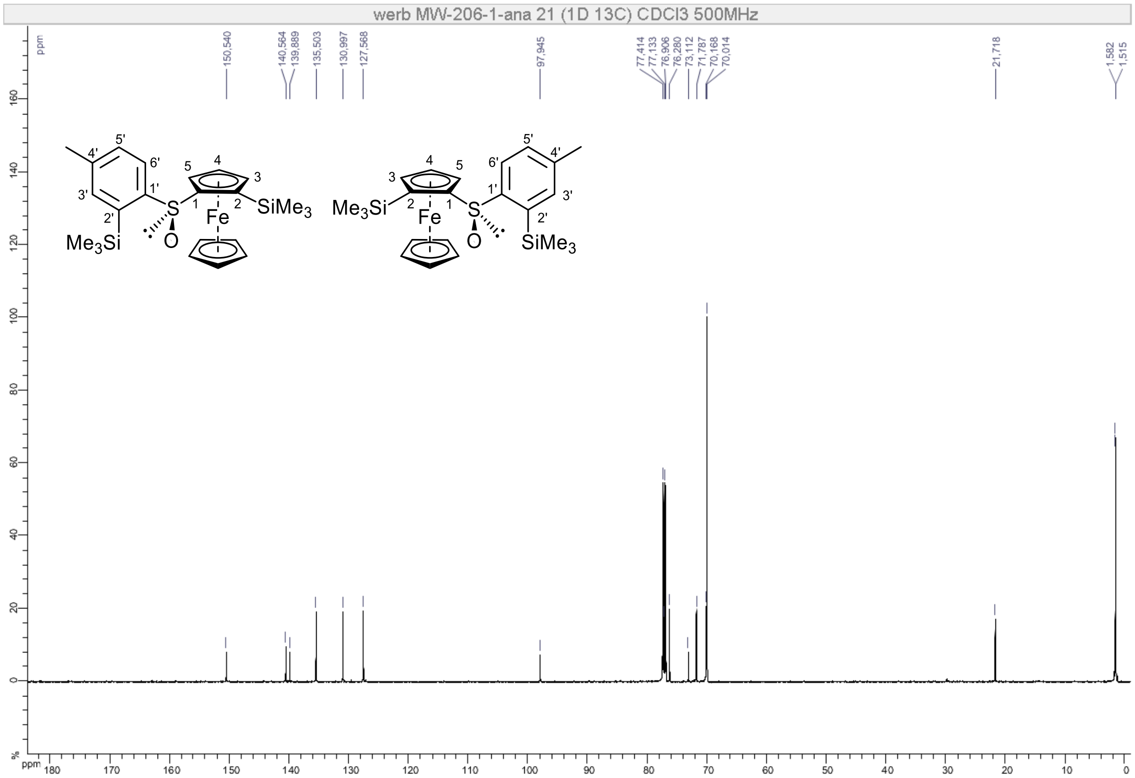

COSY (500 MHz, $CDCl_3$)

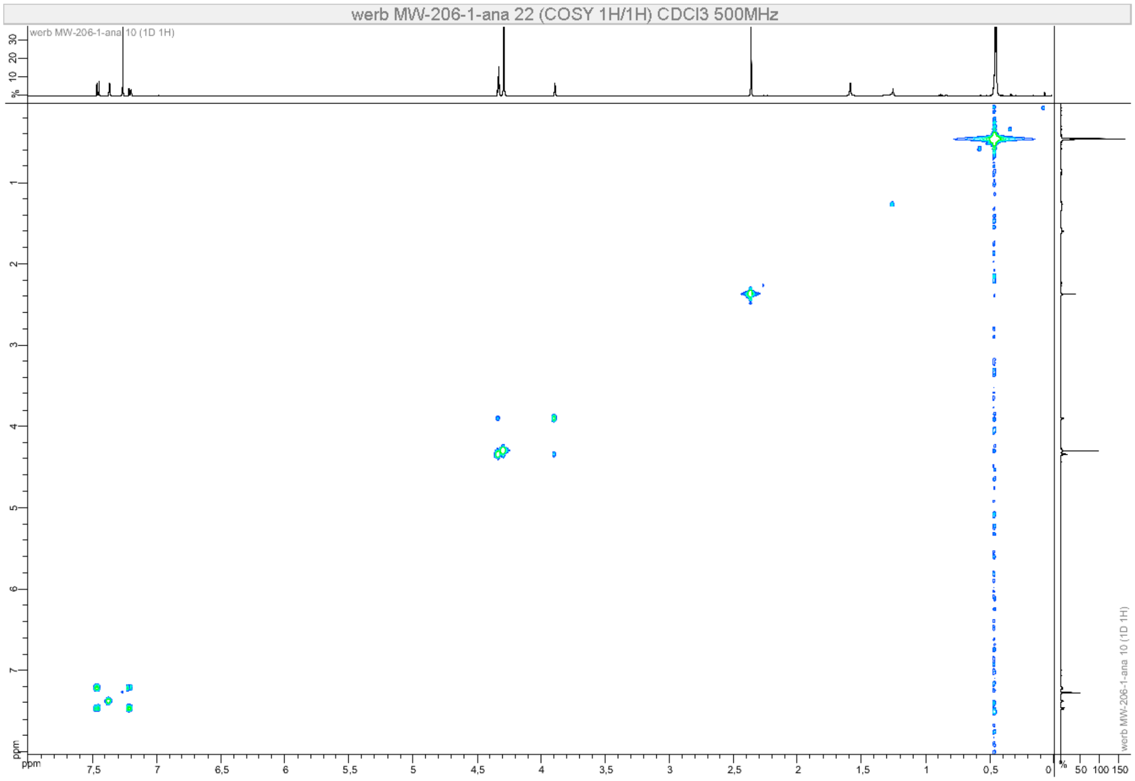

HSQC (500 MHz, $CDCl_3$)

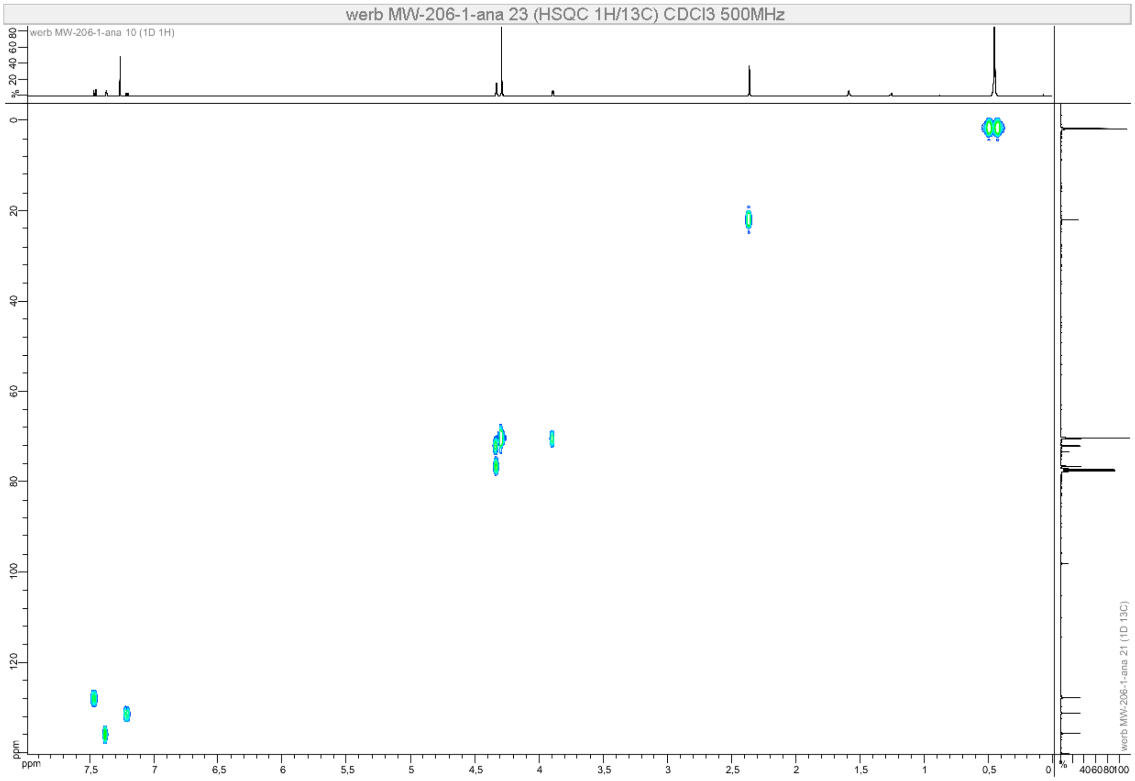

HMBC (500 MHz, $CDCl_3$)

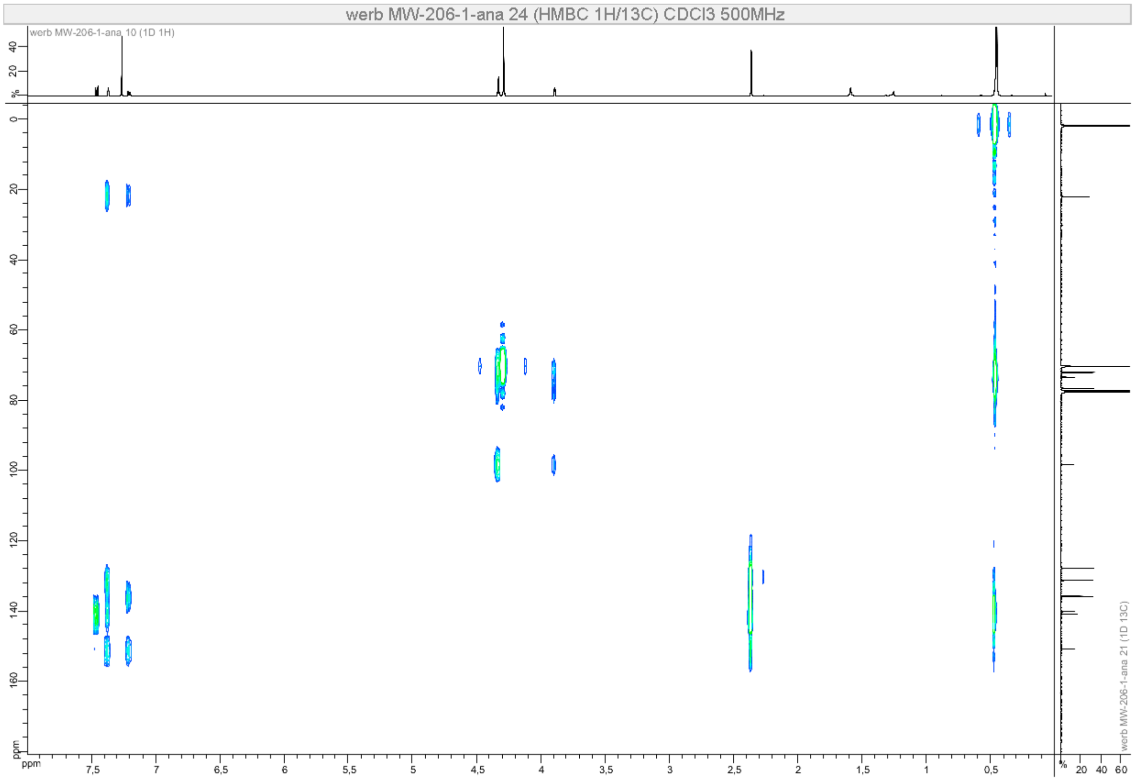

NOESY (500 MHz, $CDCl_3$)

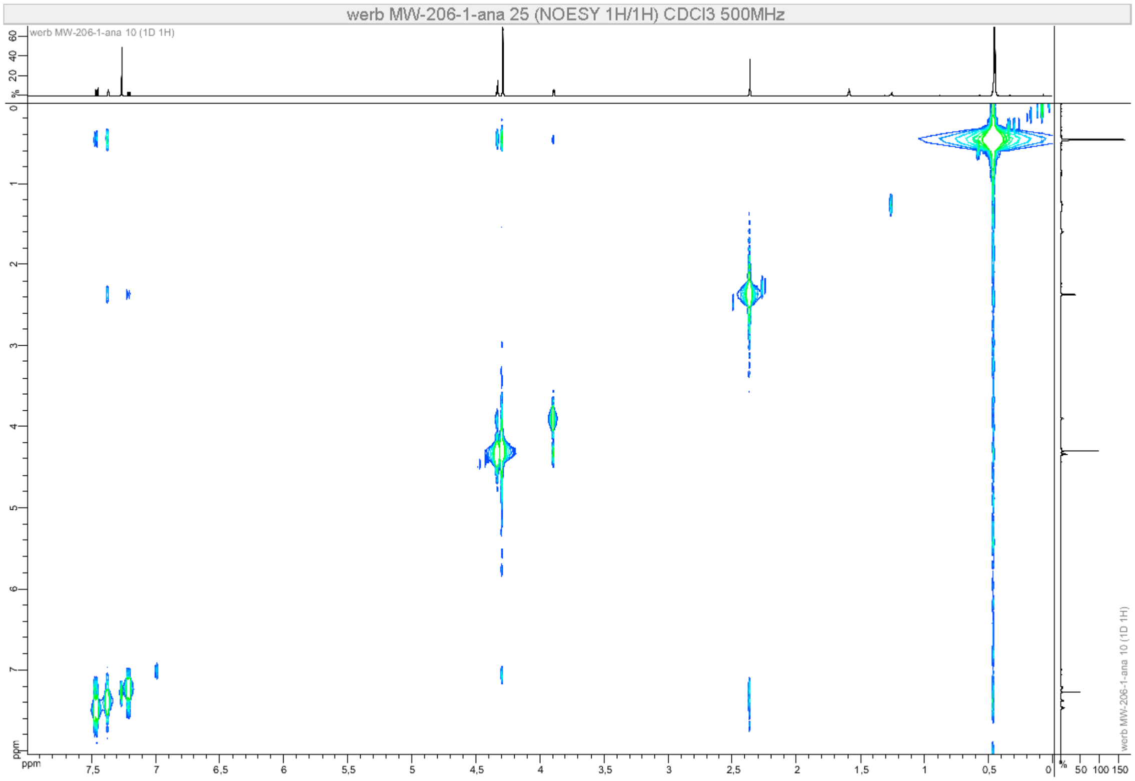

(R,S_P)- and (S,R_P)-2-Iodo-S-(4-tolyl)ferrocenesulfoxide (R,S_P/S,R_P-3b)

^{1}H NMR (500 MHz, $CDCl_3$)

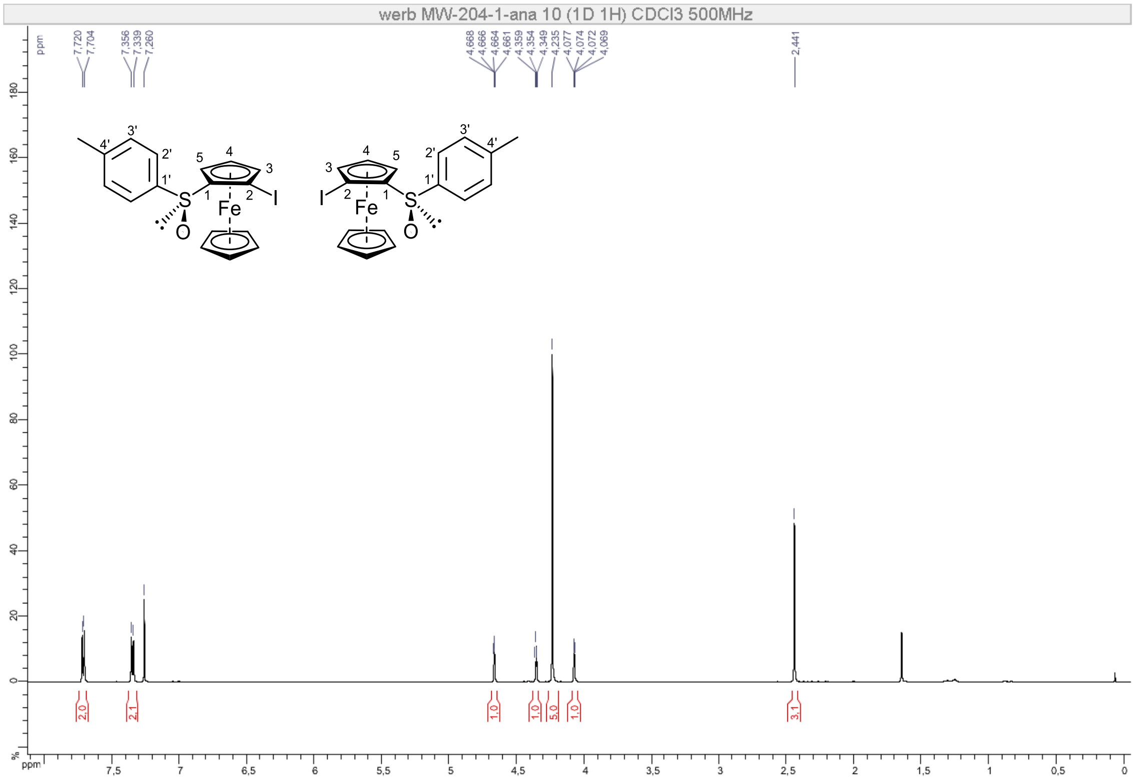

^{13}C NMR (126 MHz, $CDCl_3$)

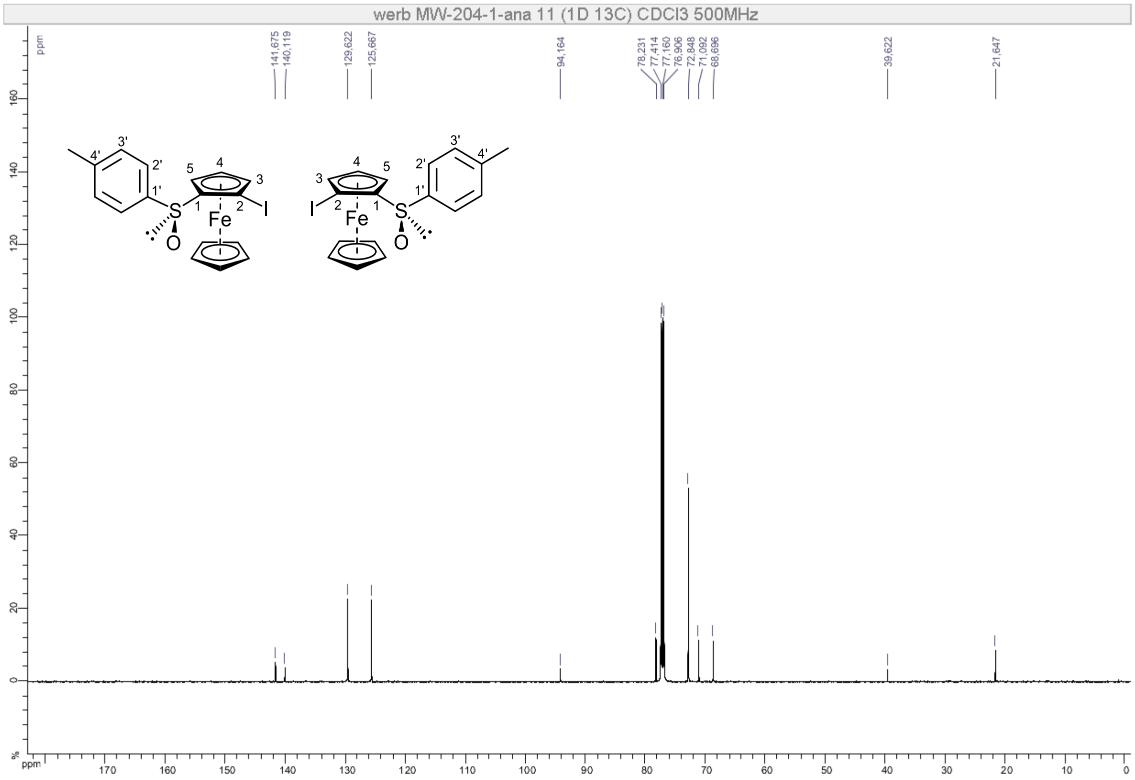

COSY (500 MHz, $CDCl_3$)

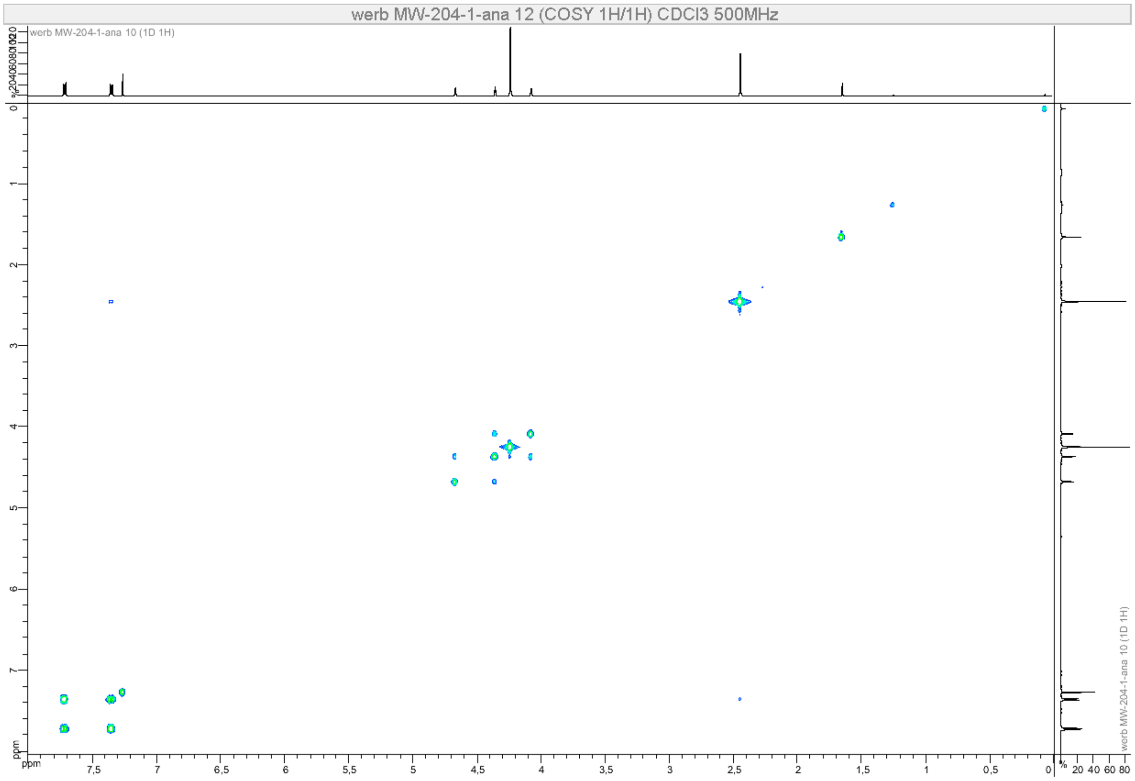

HSQC (500 MHz, $CDCl_3$)

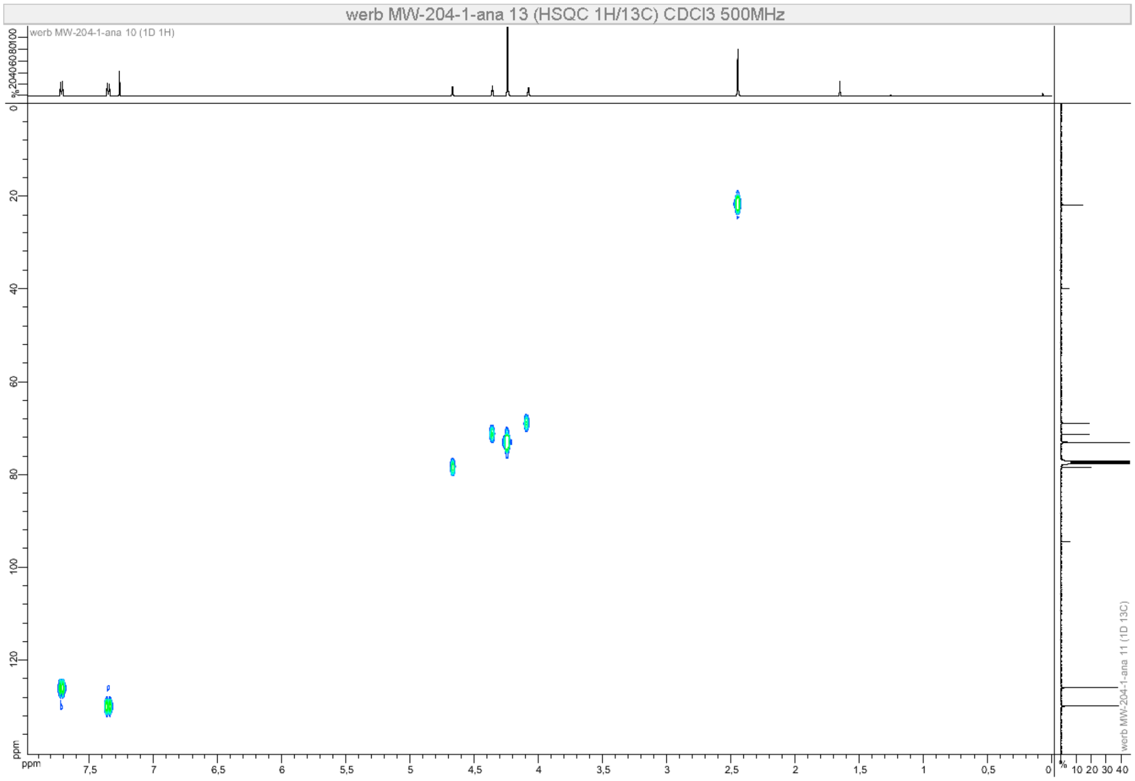

HMBC (500 MHz, $CDCl_3$)

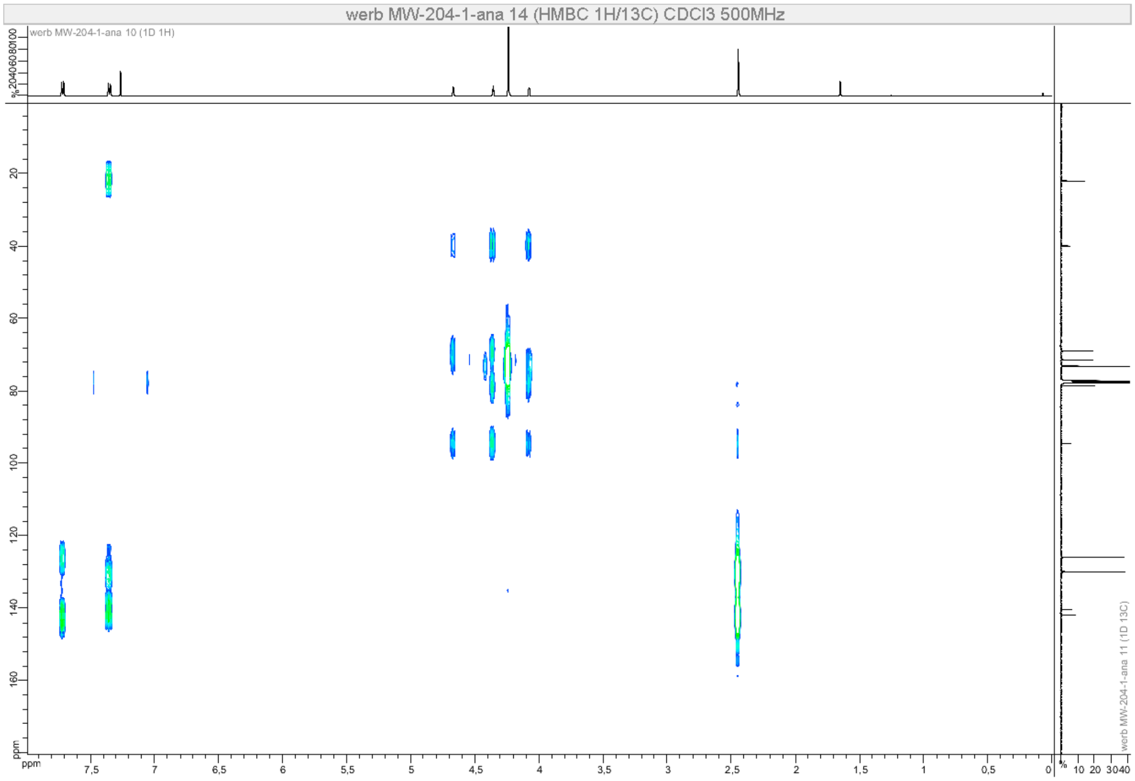

NOESY (500 MHz, $CDCl_3$)

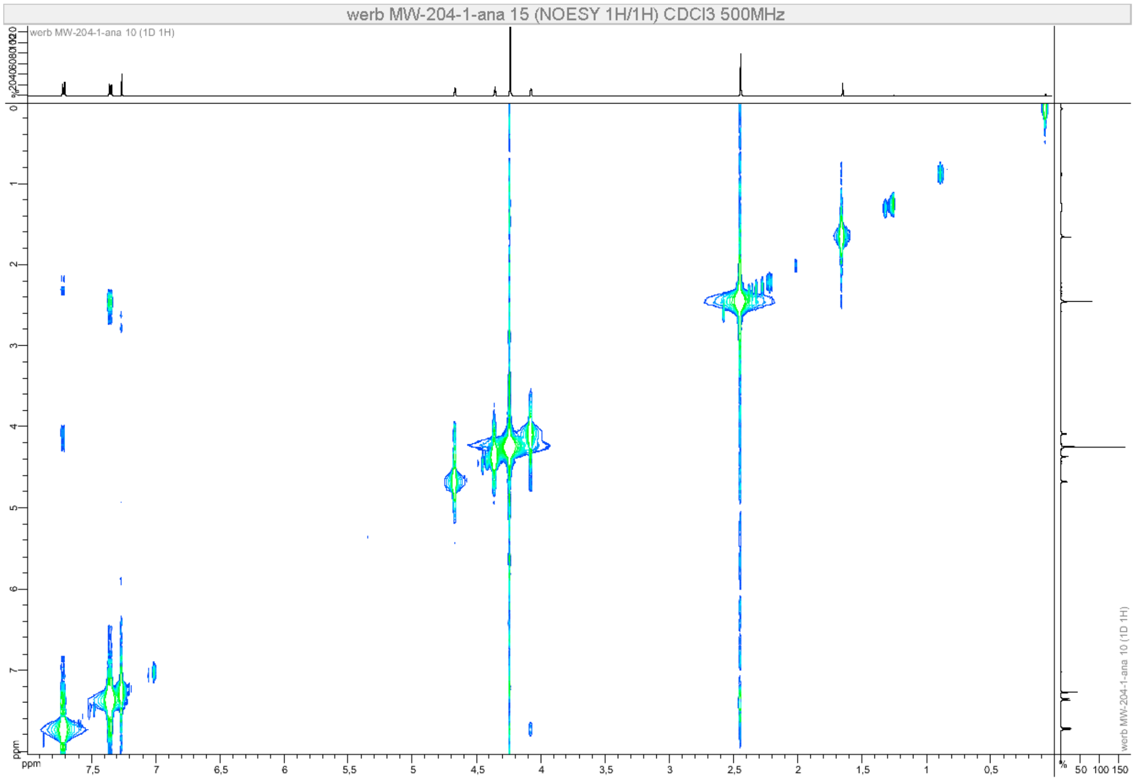

(*S*,*S*P)-2-Deuterio-*S*-(4-tolyl)ferrocenesulfoxide (*S*,*S*P-3c)

^{1}H NMR (500 MHz, $CDCl_3$)

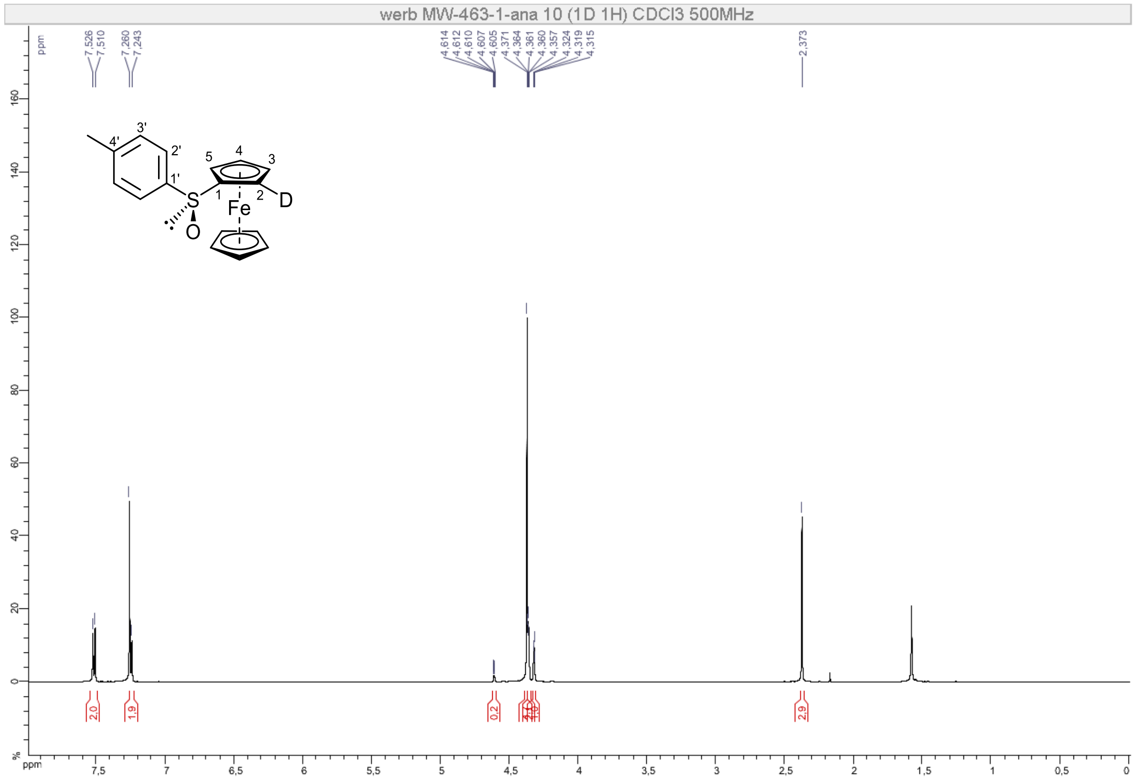

^{13}C NMR (126 MHz, $CDCl_3$)

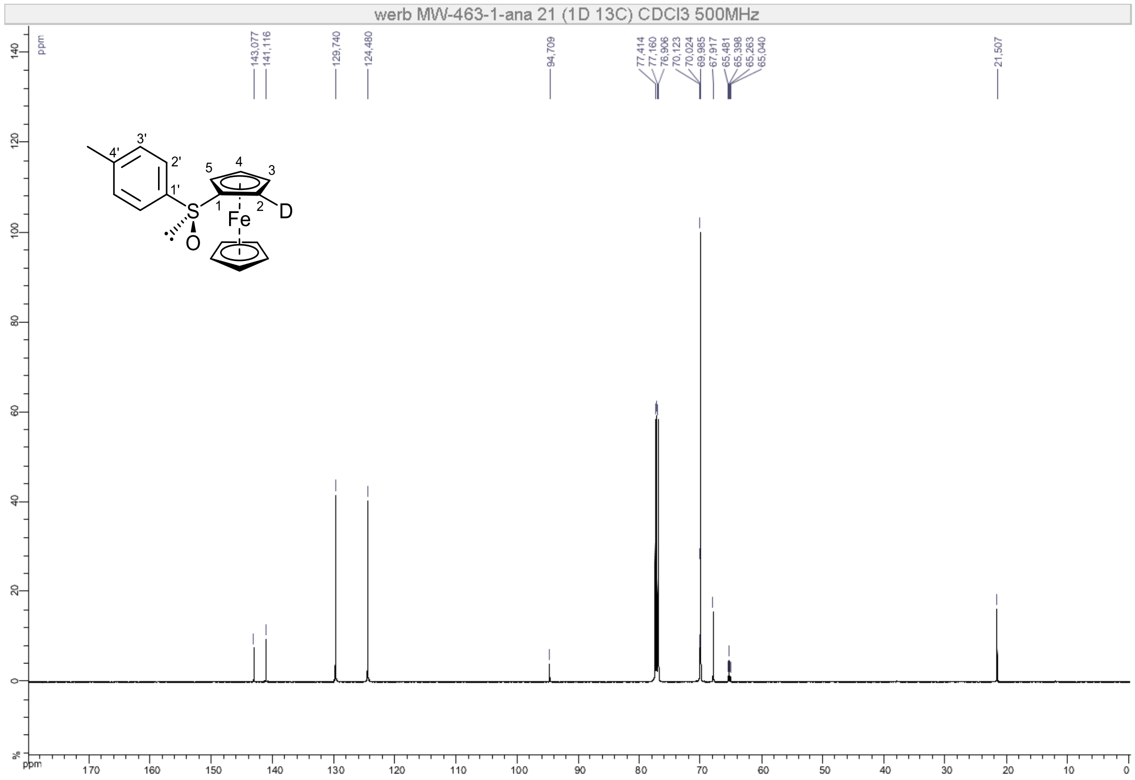

COSY (500 MHz, $CDCl_3$)

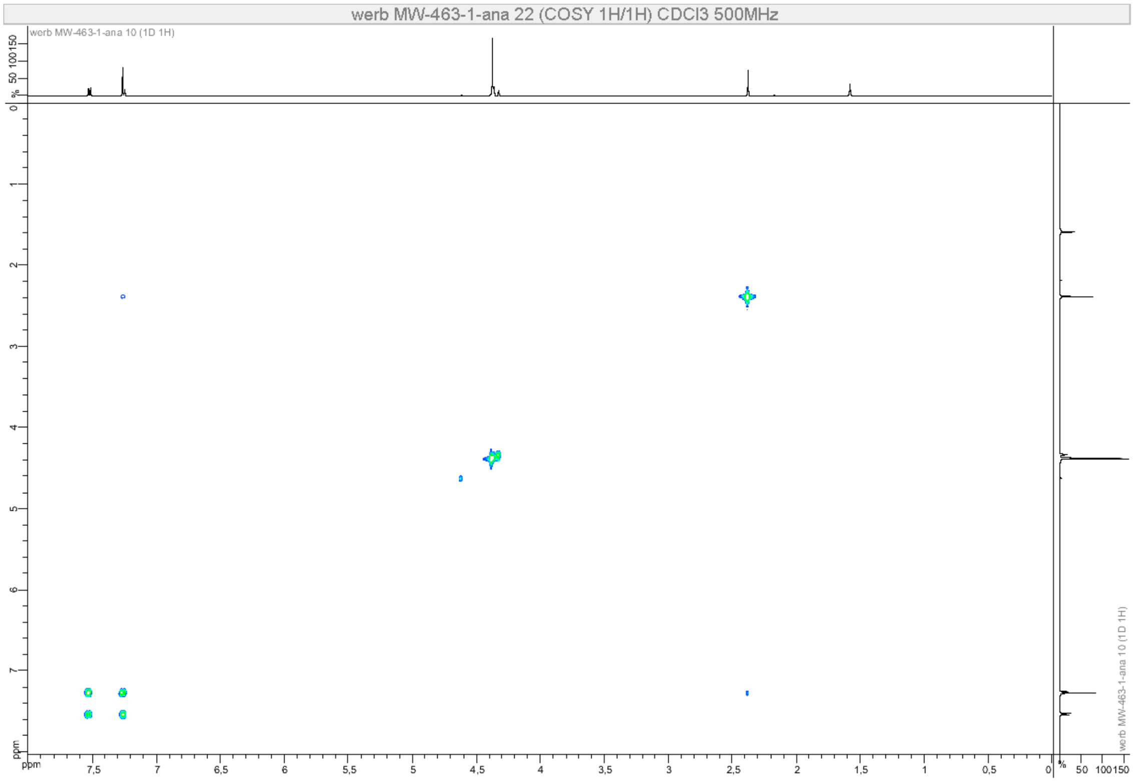

HSQC (500 MHz, $CDCl_3$)

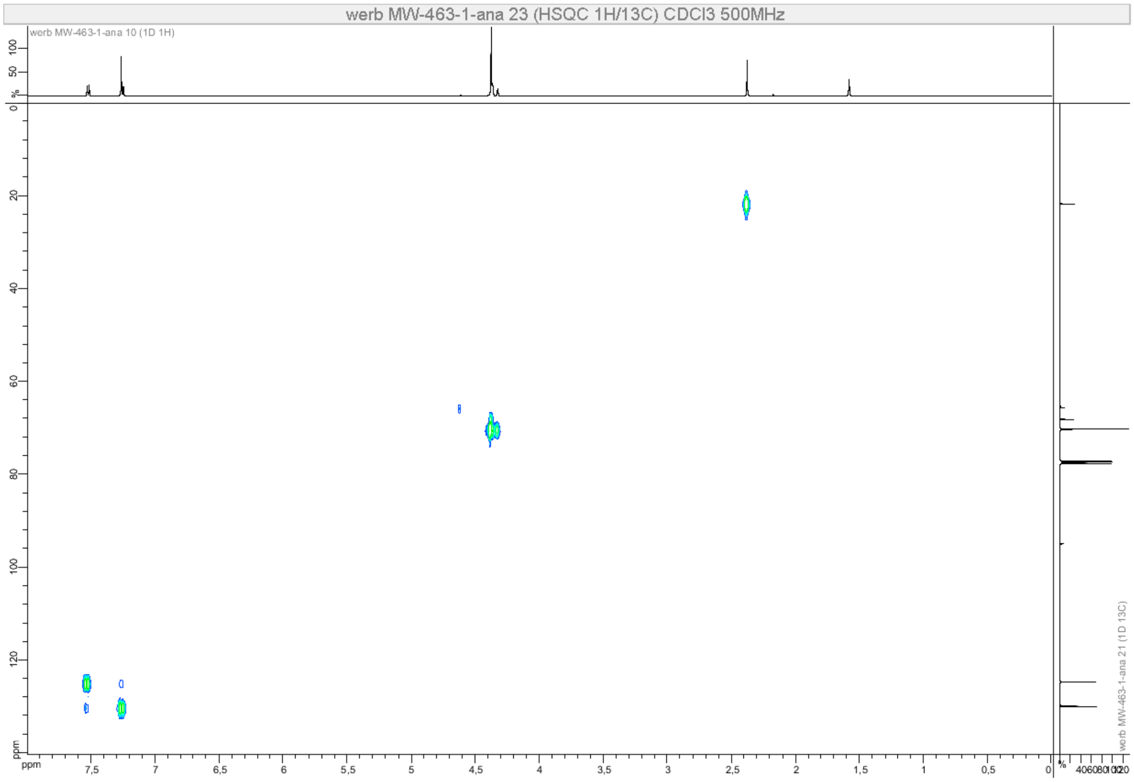

HMBC (500 MHz, $CDCl_3$)

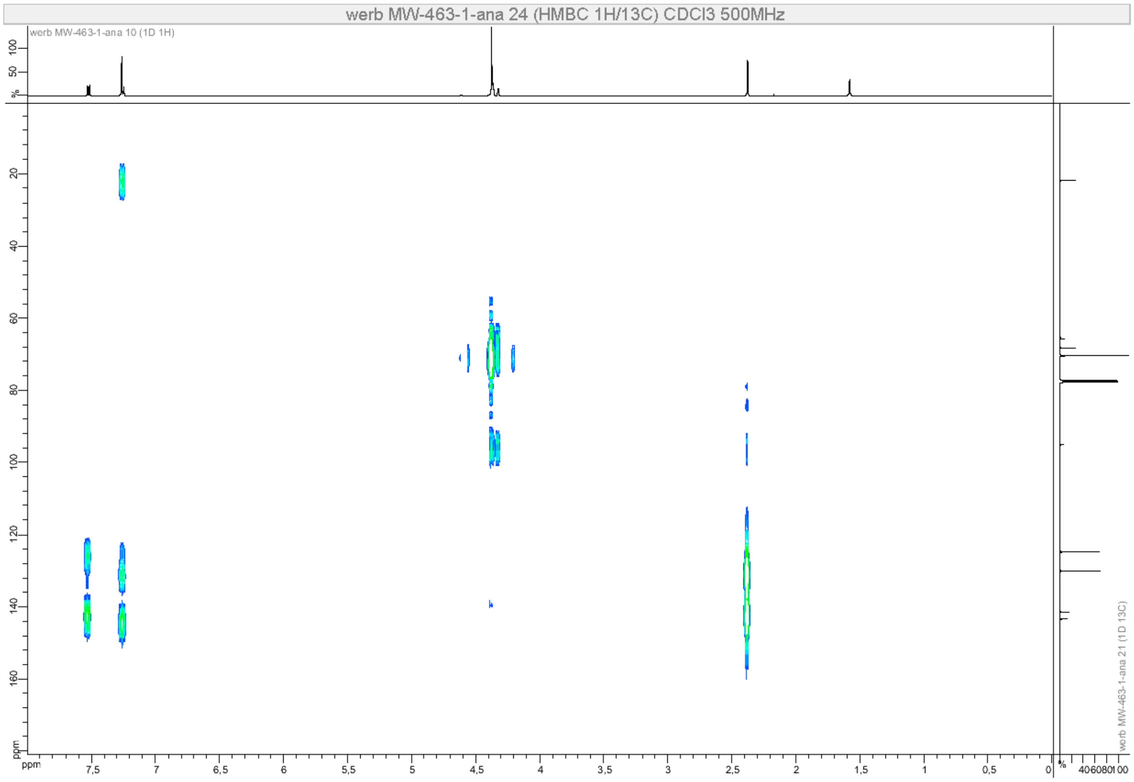

NOESY (500 MHz, $CDCl_3$)

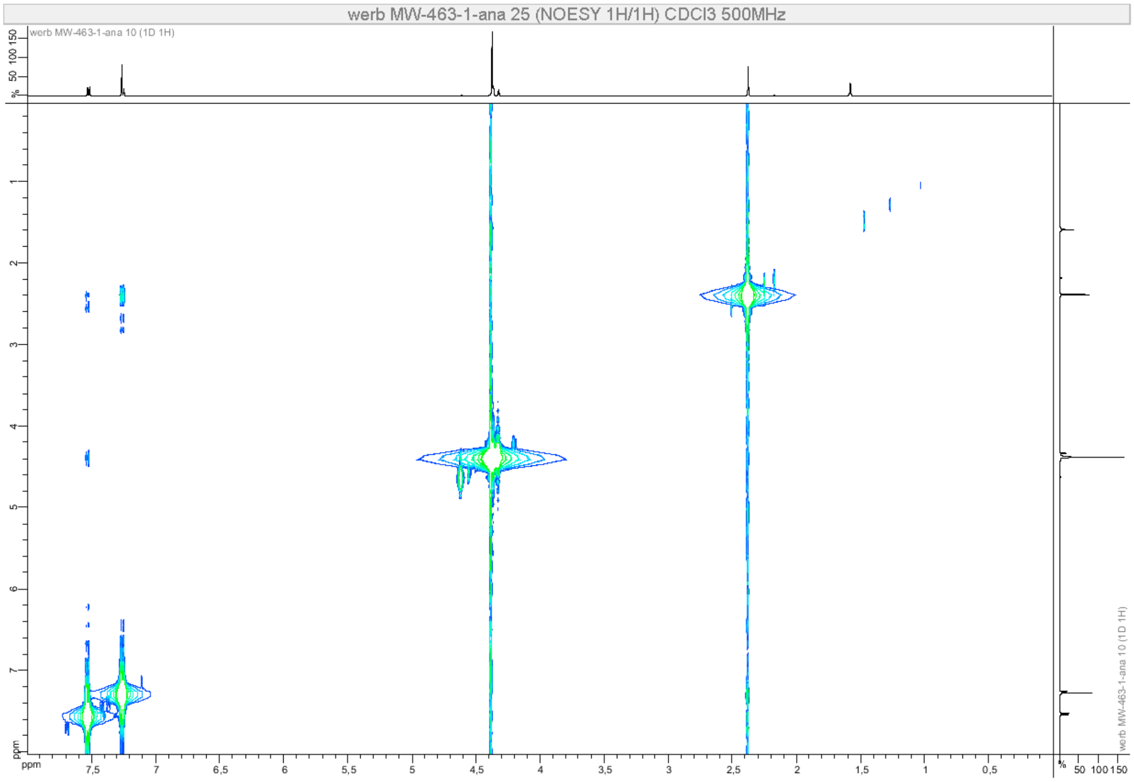

(*R*,*R*P)- and (*S*,*S*P)-*S*-(2-Iodo-4-tolyl)-2-(trimethylsilyl)ferrocenesulfoxide (*R*,*R*P/*S*,*S*P-4)

^{1}H NMR (500 MHz, $CDCl_3$)

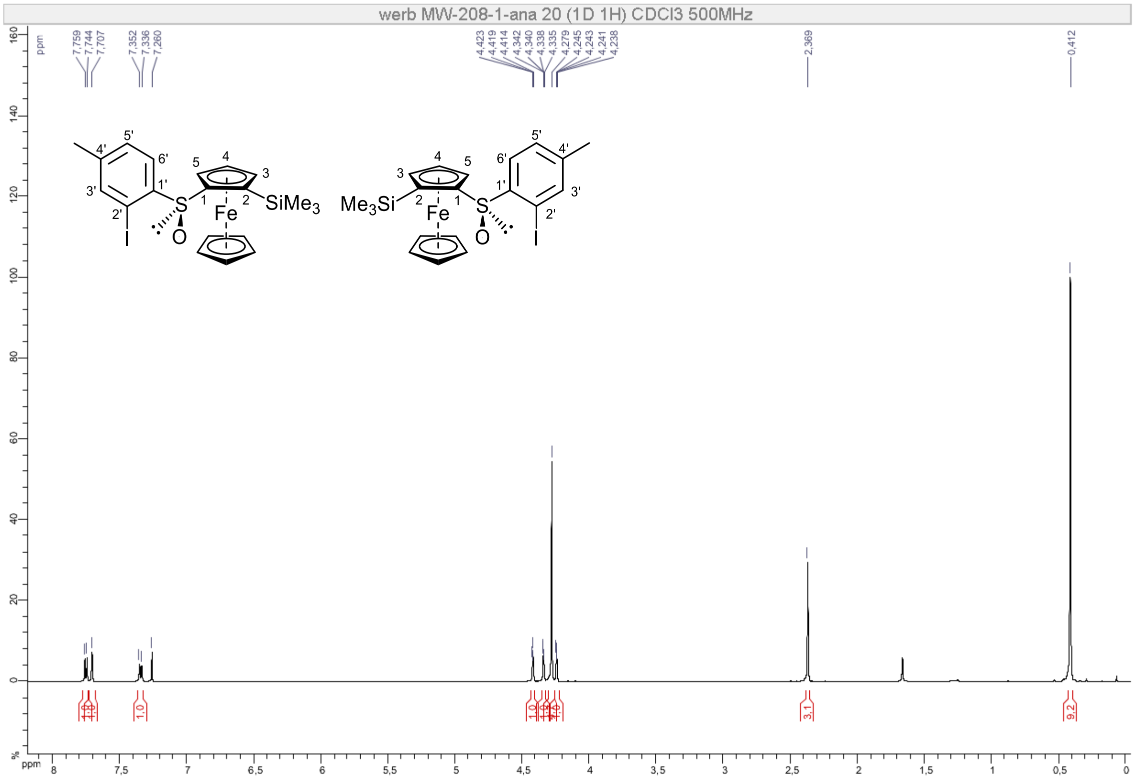

^{13}C NMR (126 MHz, $CDCl_3$)

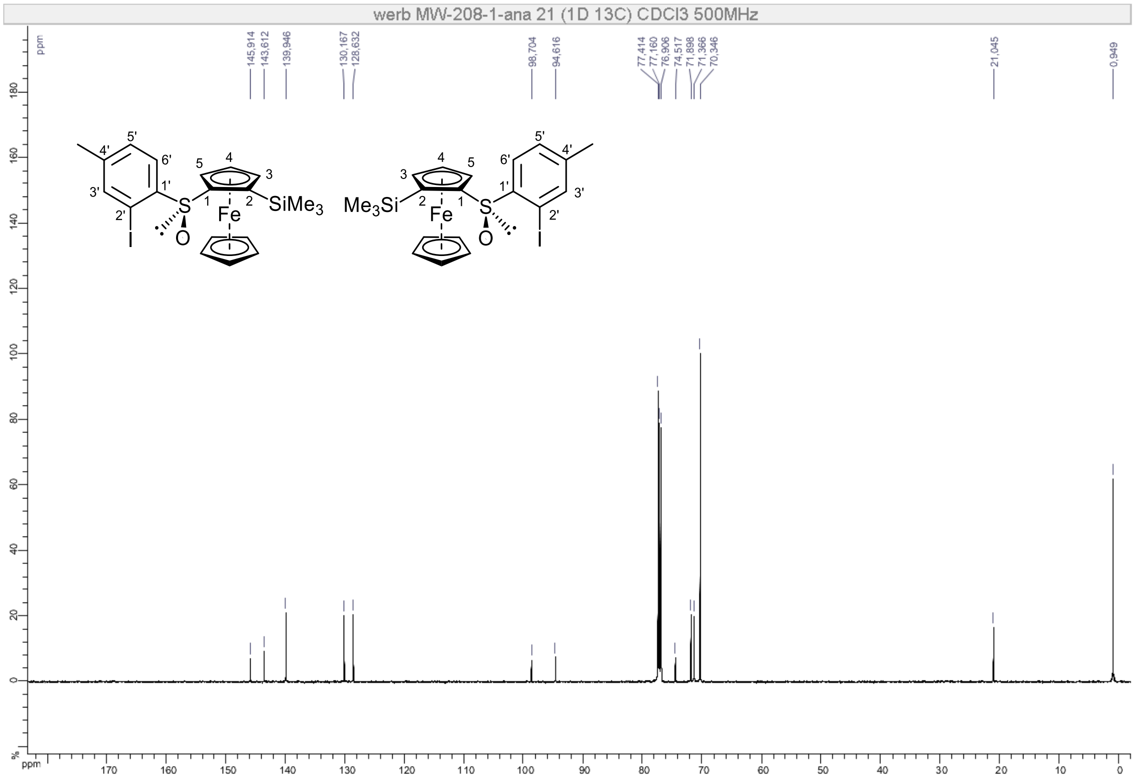

COSY (500 MHz, $CDCl_3$)

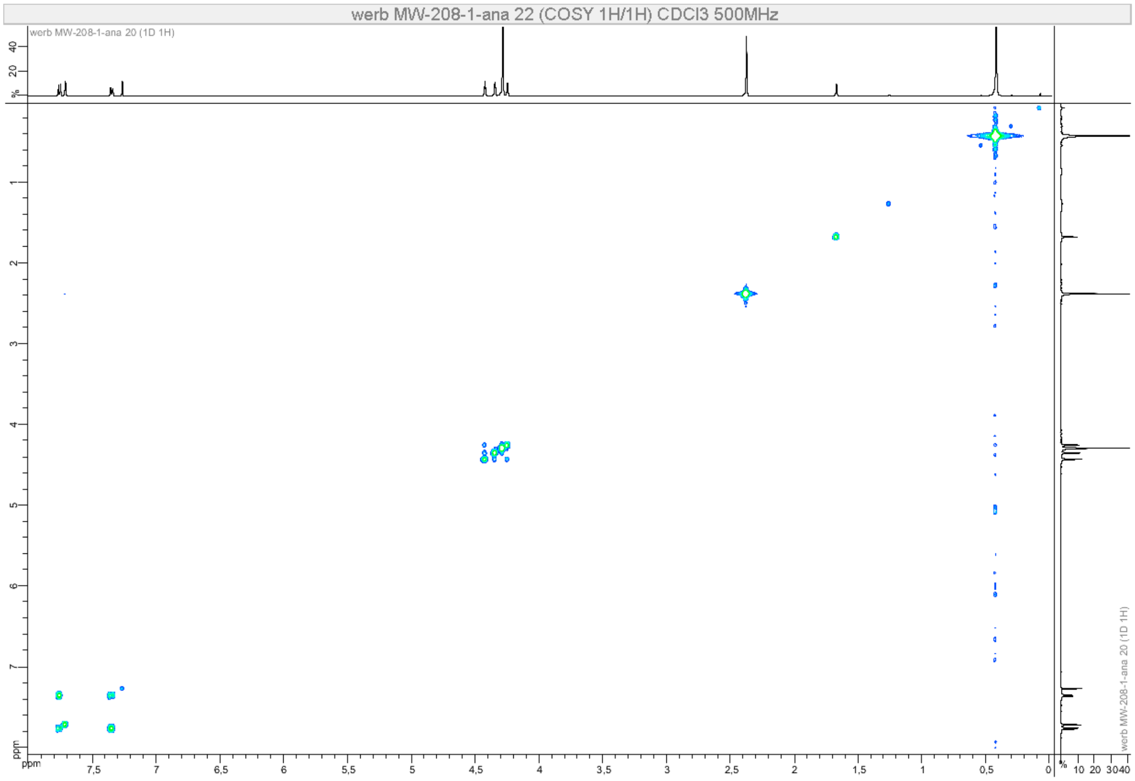

HSQC (500 MHz, $CDCl_3$)

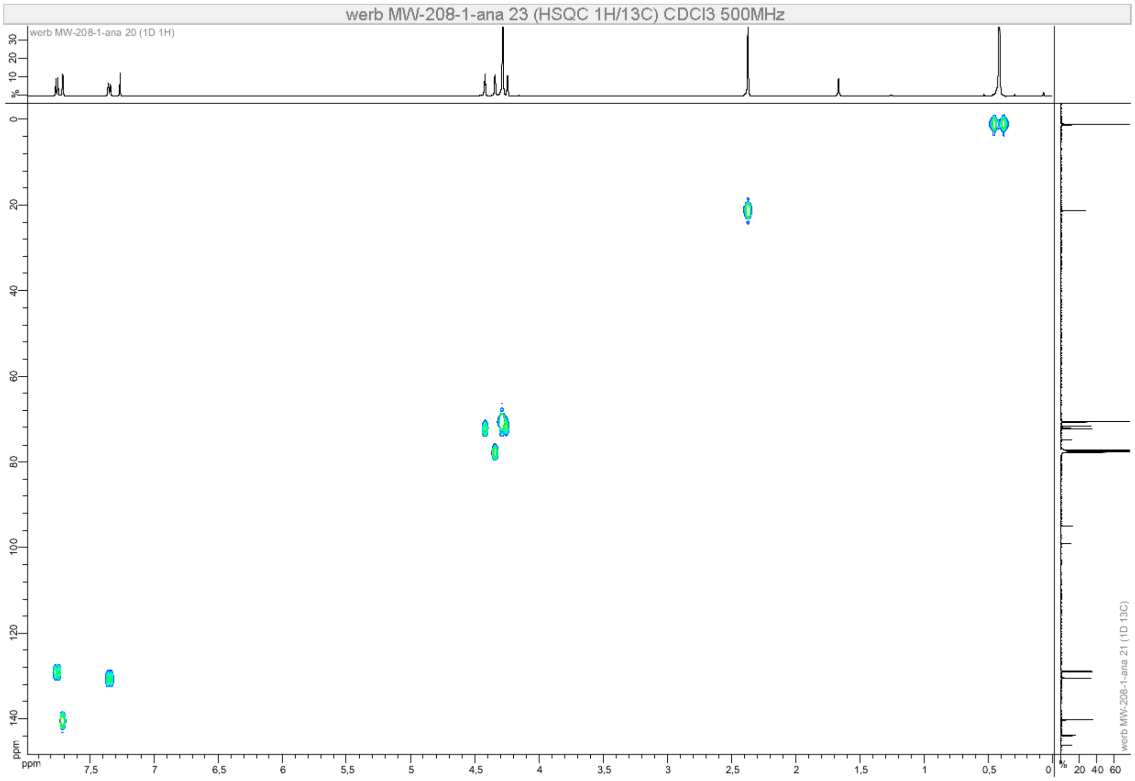

HMBC (500 MHz, $CDCl_3$)

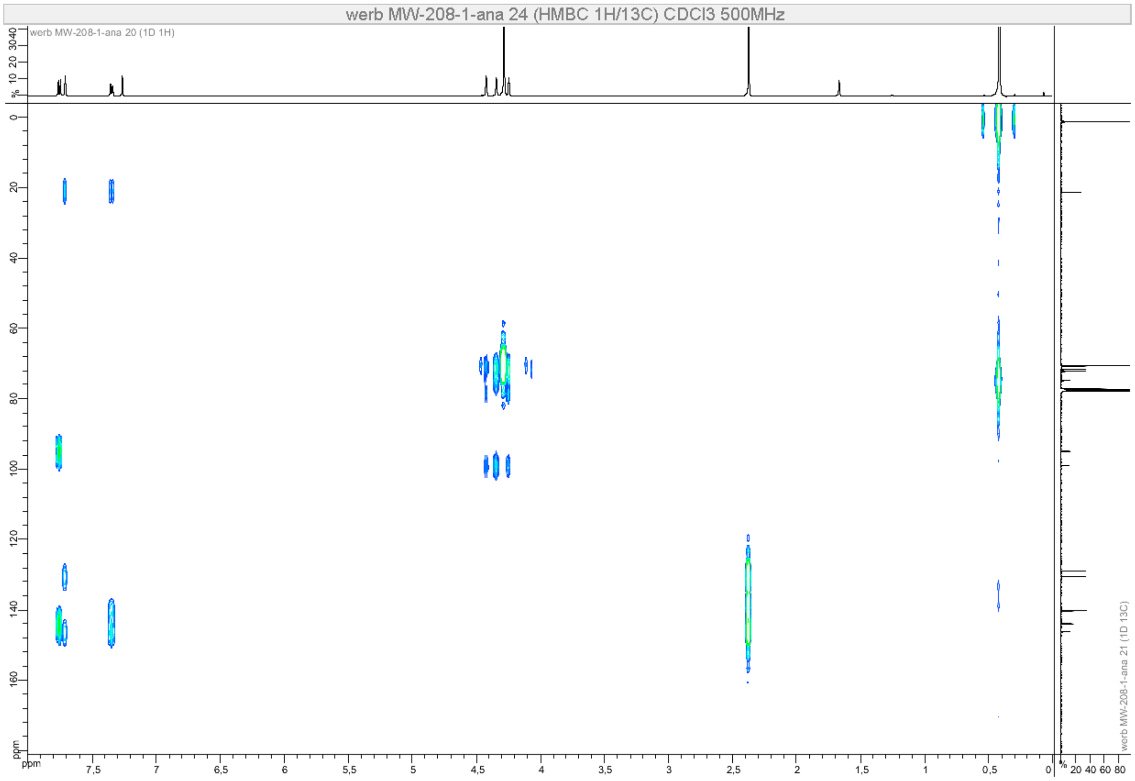

NOESY (500 MHz, $CDCl_3$)

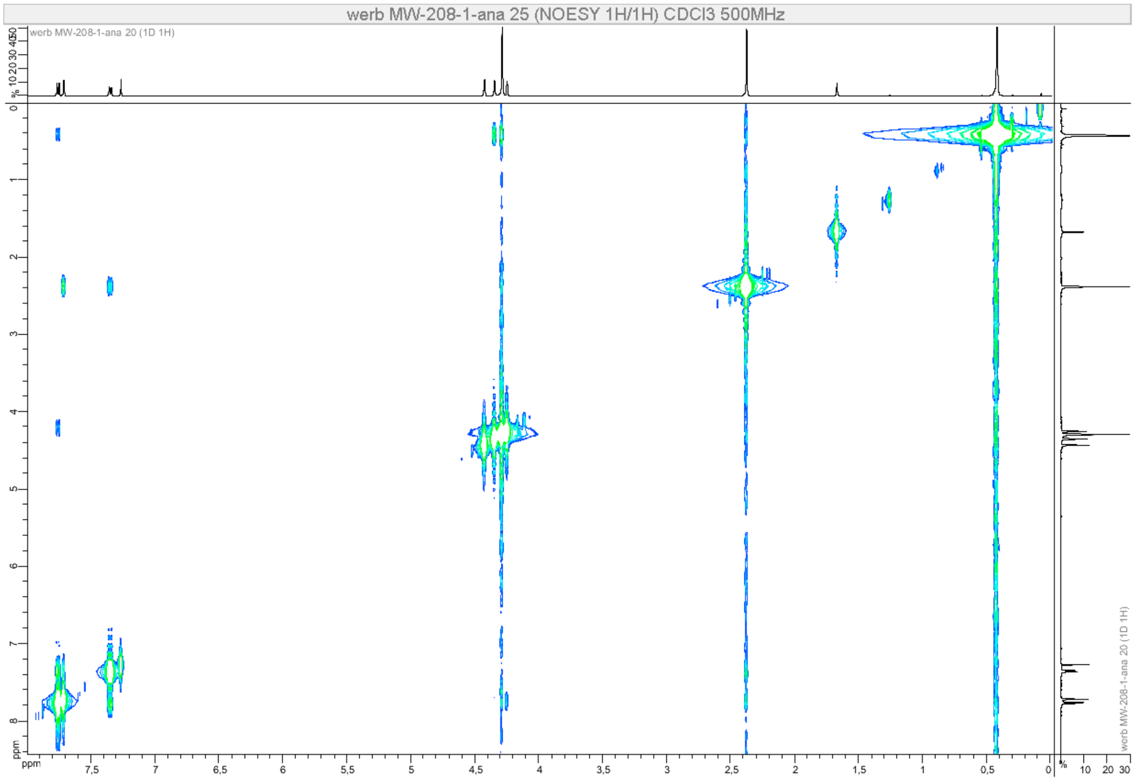

Racemic *S*-(2-Iodo-4-tolyl)ferrocenesulfoxide (*rac*-5)

^{1}H NMR (500 MHz, $CDCl_3$)

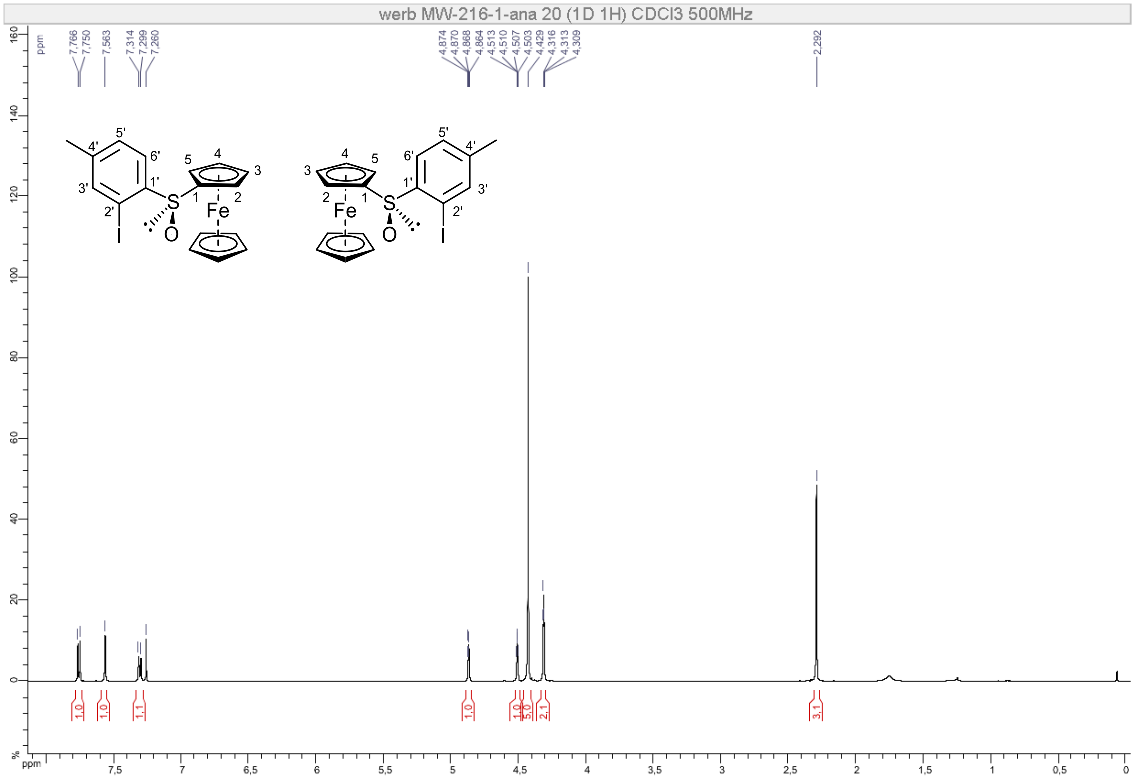

^{13}C NMR (126 MHz, $CDCl_3$)

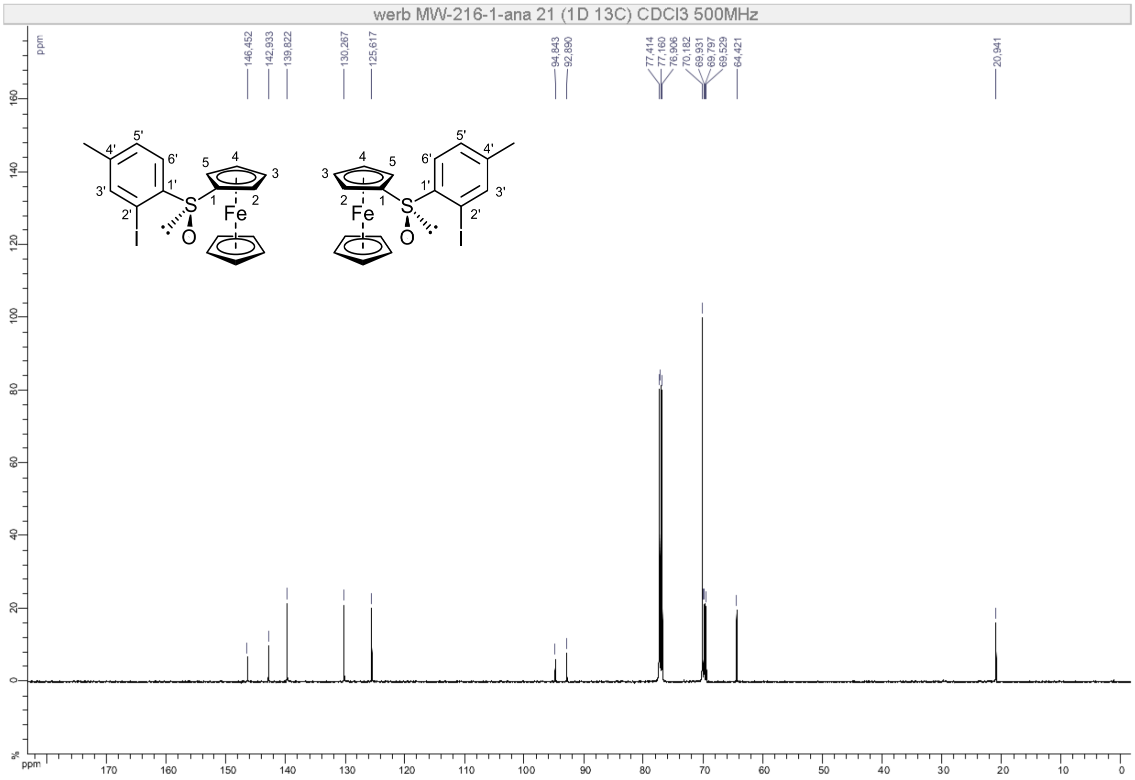

COSY (500 MHz, $CDCl_3$)

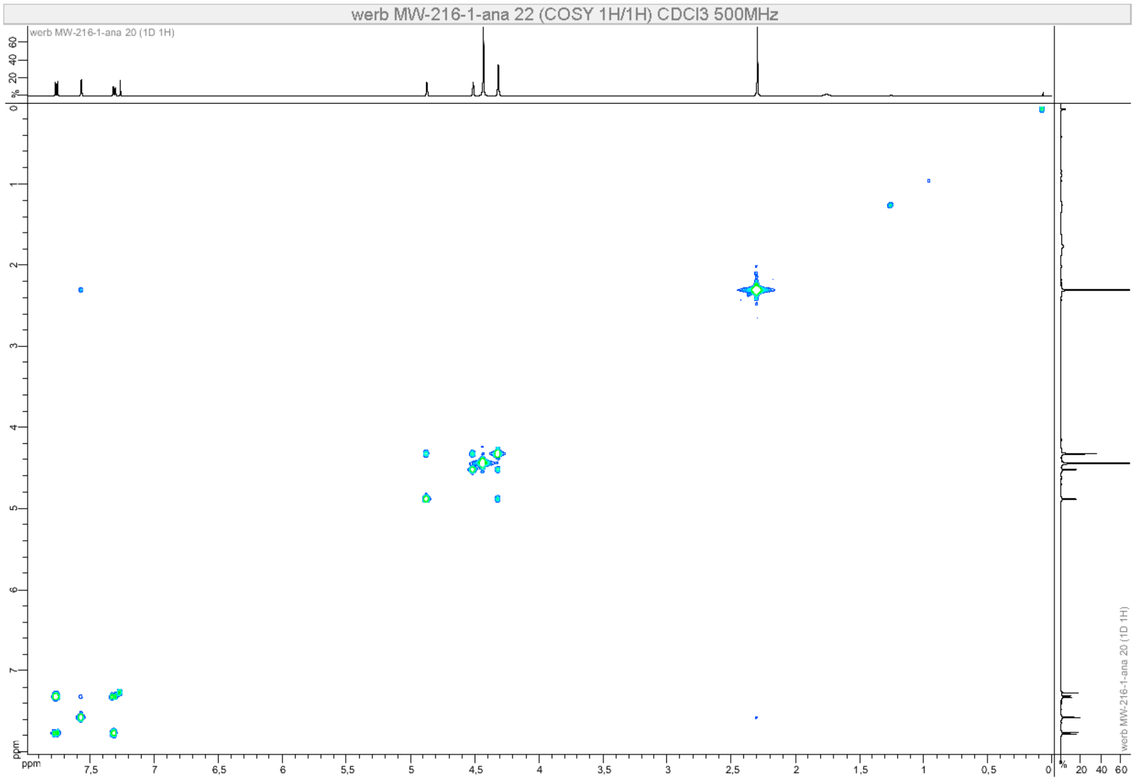

HSQC (500 MHz, $CDCl_3$)

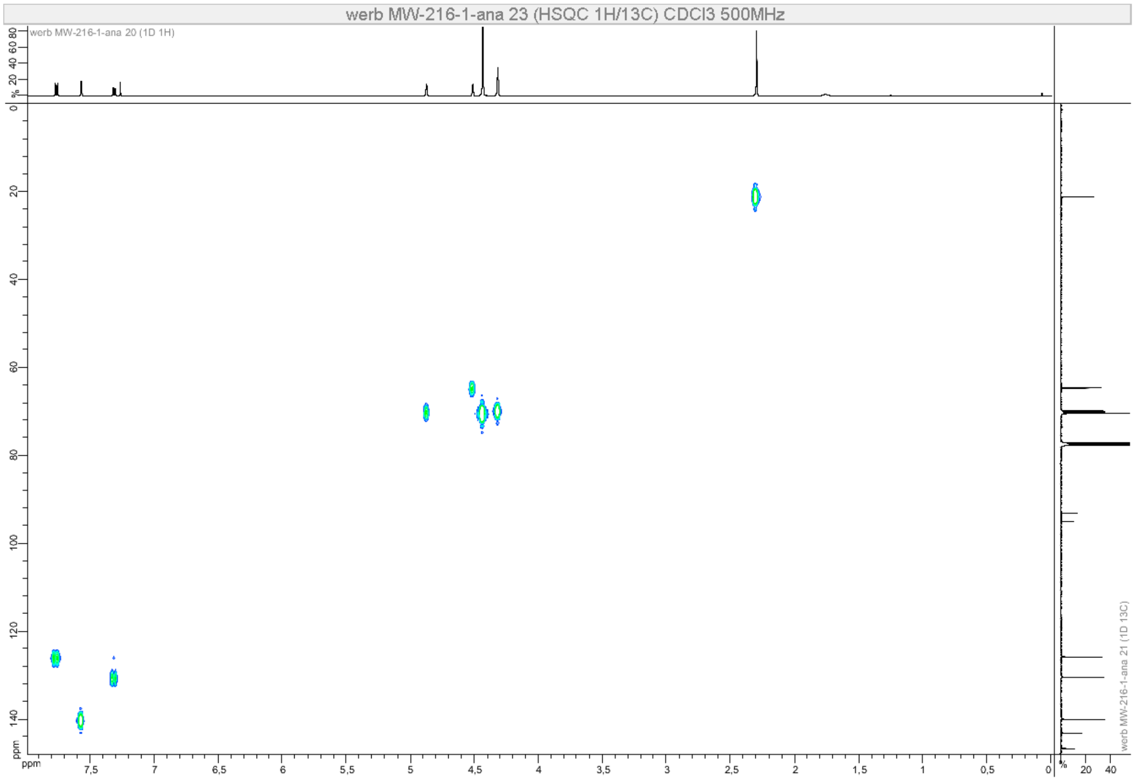

HMBC (500 MHz, $CDCl_3$)

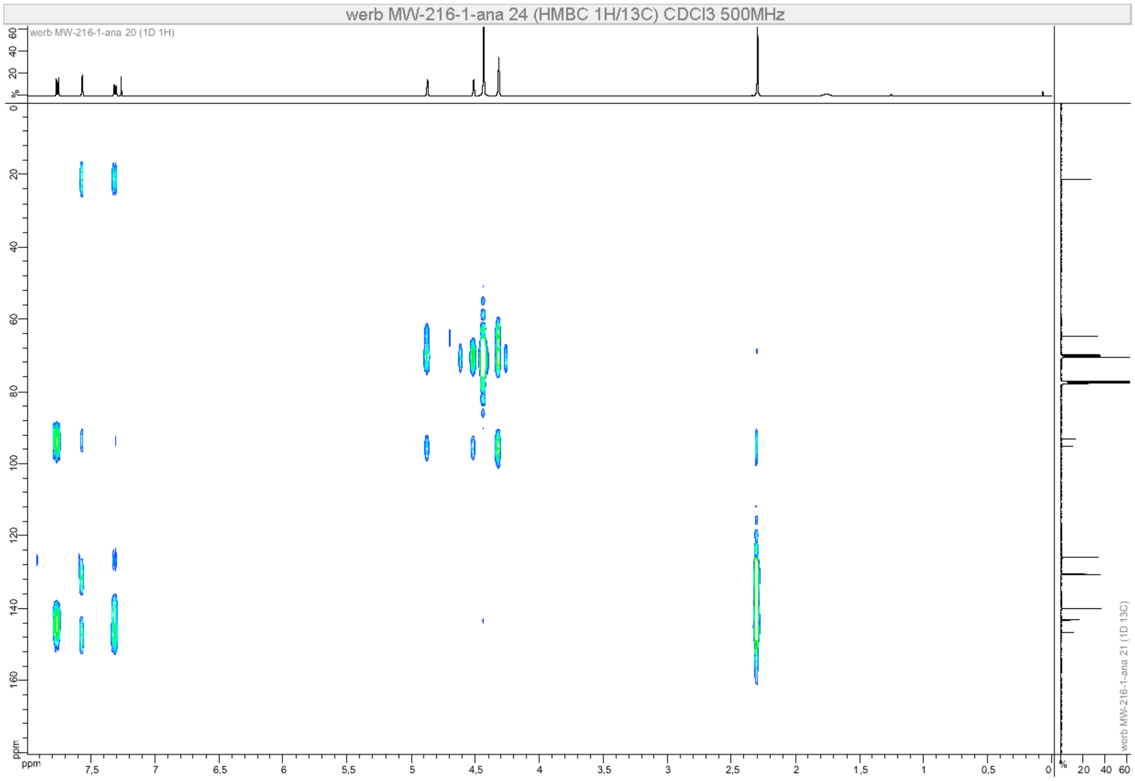

(*R*,*S*P)- and (*S*,*R*P)-2-Iodo-*S*-(4-tolyl)-5-(trimethylsilyl)ferrocenesulfoxide (*R*,*S*P/*S*,*R*P-6)

^{1}H NMR (500 MHz, $CDCl_3$)

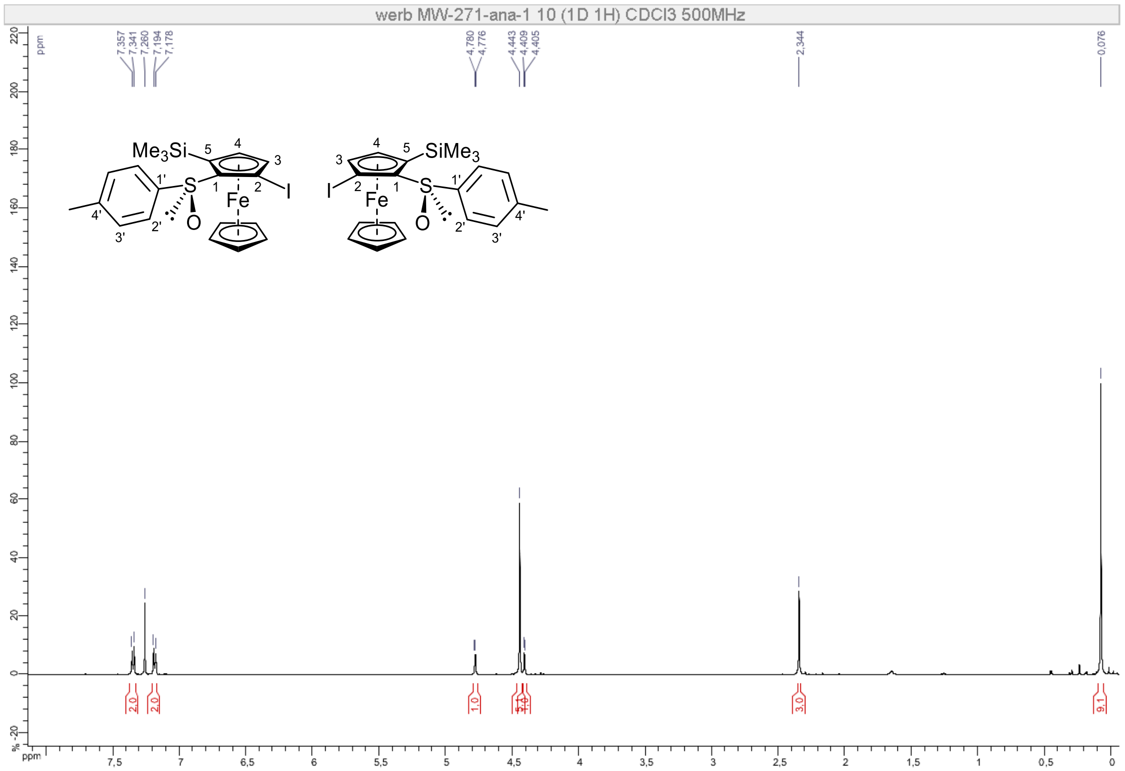

^{13}C NMR (126 MHz, $CDCl_3$)

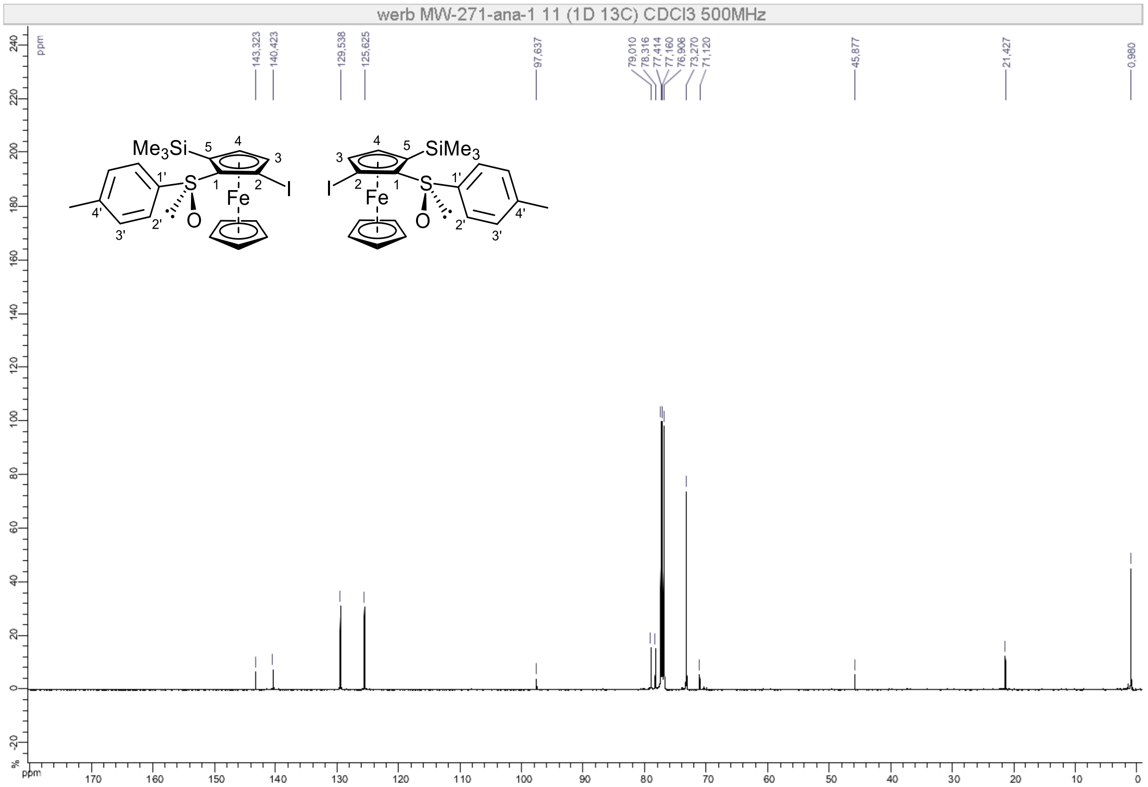

DEPT 135 (126 MHz, $CDCl_3$)

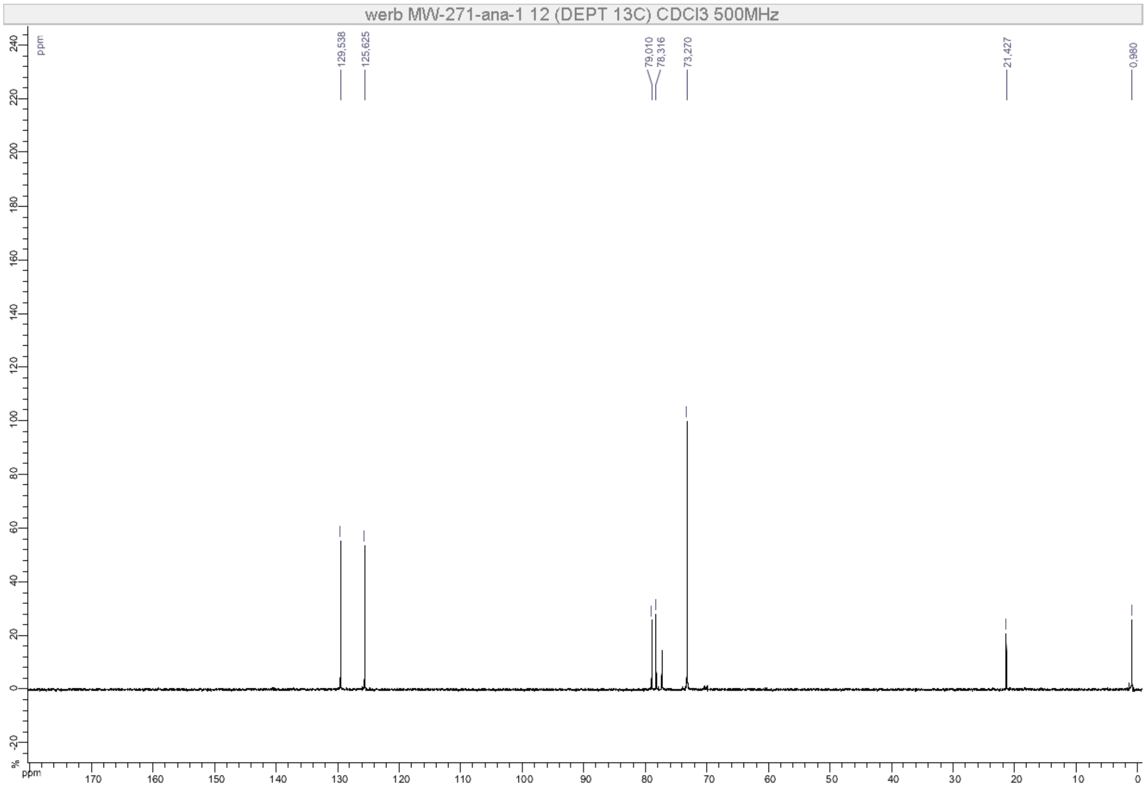

COSY (500 MHz, $CDCl_3$)

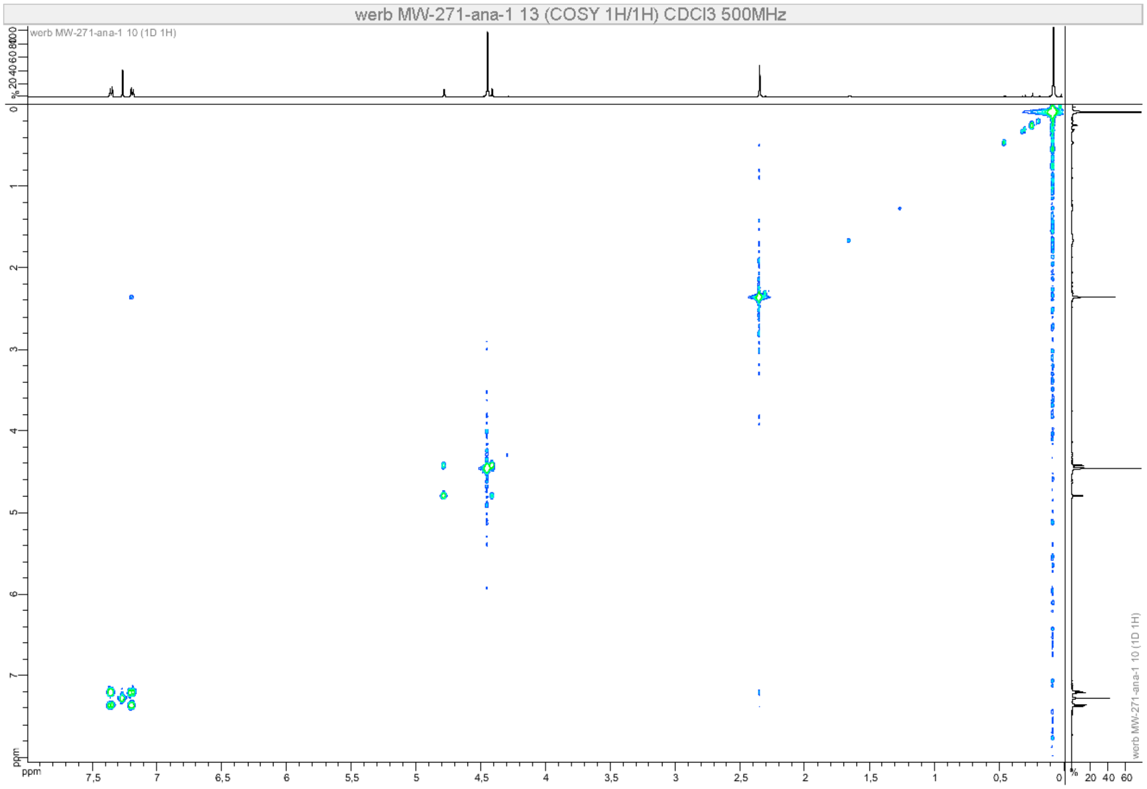

HSQC (500 MHz, $CDCl_3$)

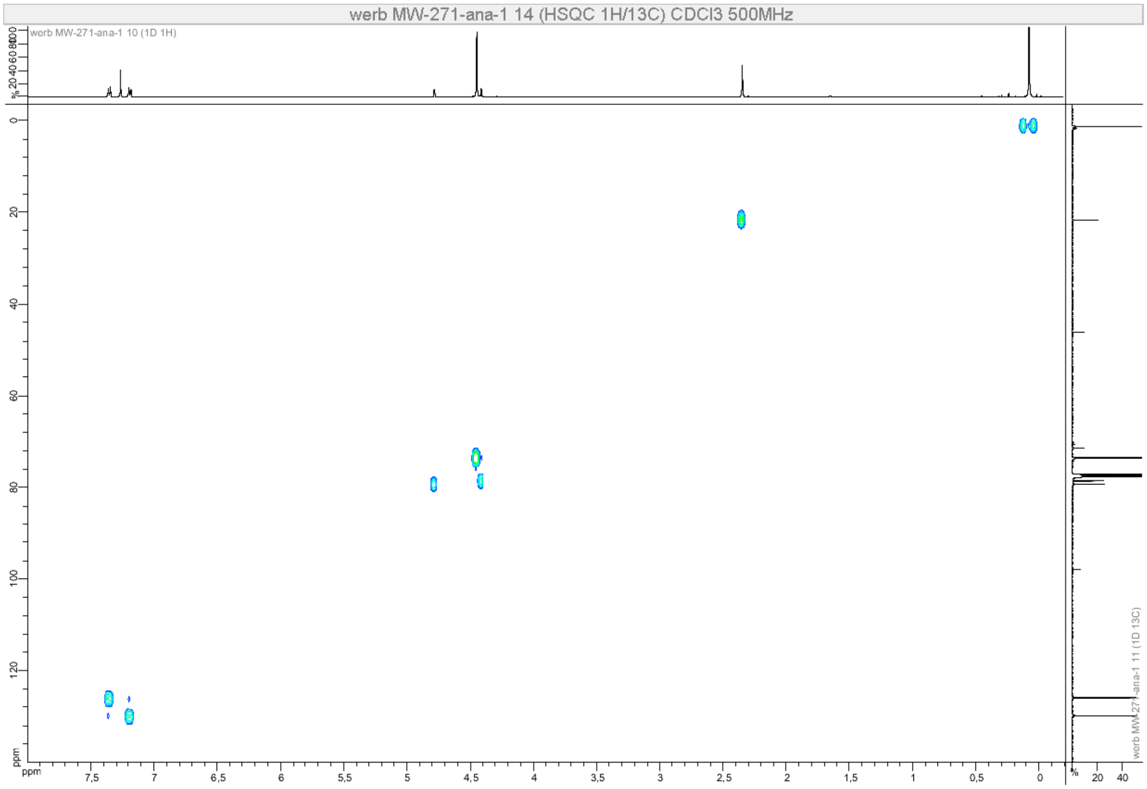

HMBC (500 MHz, $CDCl_3$)

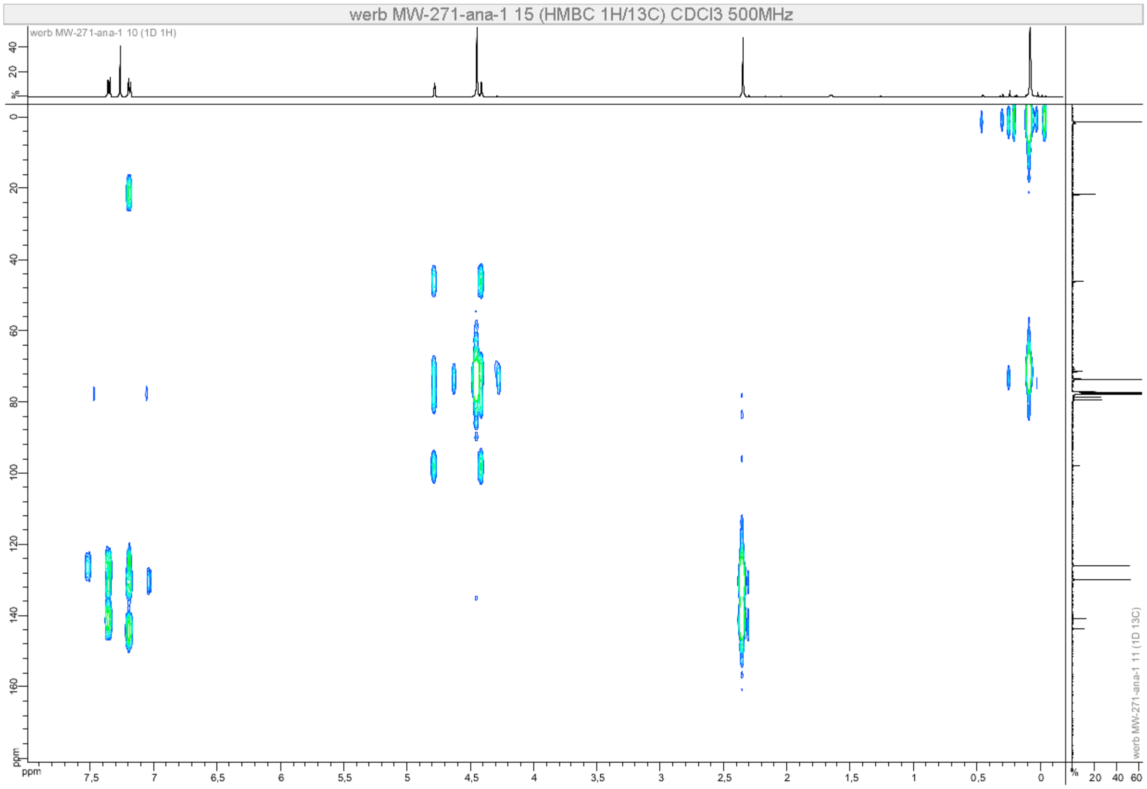

NOESY (500 MHz, $CDCl_3$)

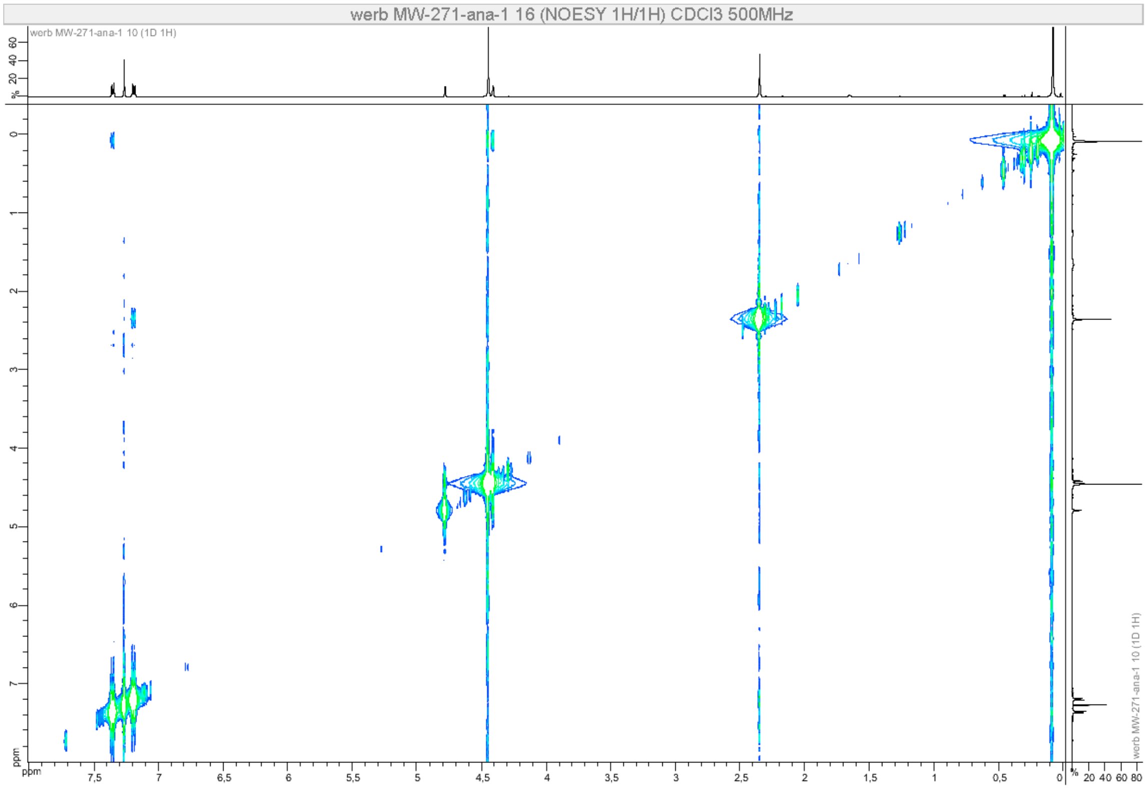

(*S*,*S*P)-*S*-*tert*-Butyl-2-iodoferrocenesulfoxide (*S*,*S*P-2ab)

^{1}H NMR (300 MHz, $CDCl_3$)

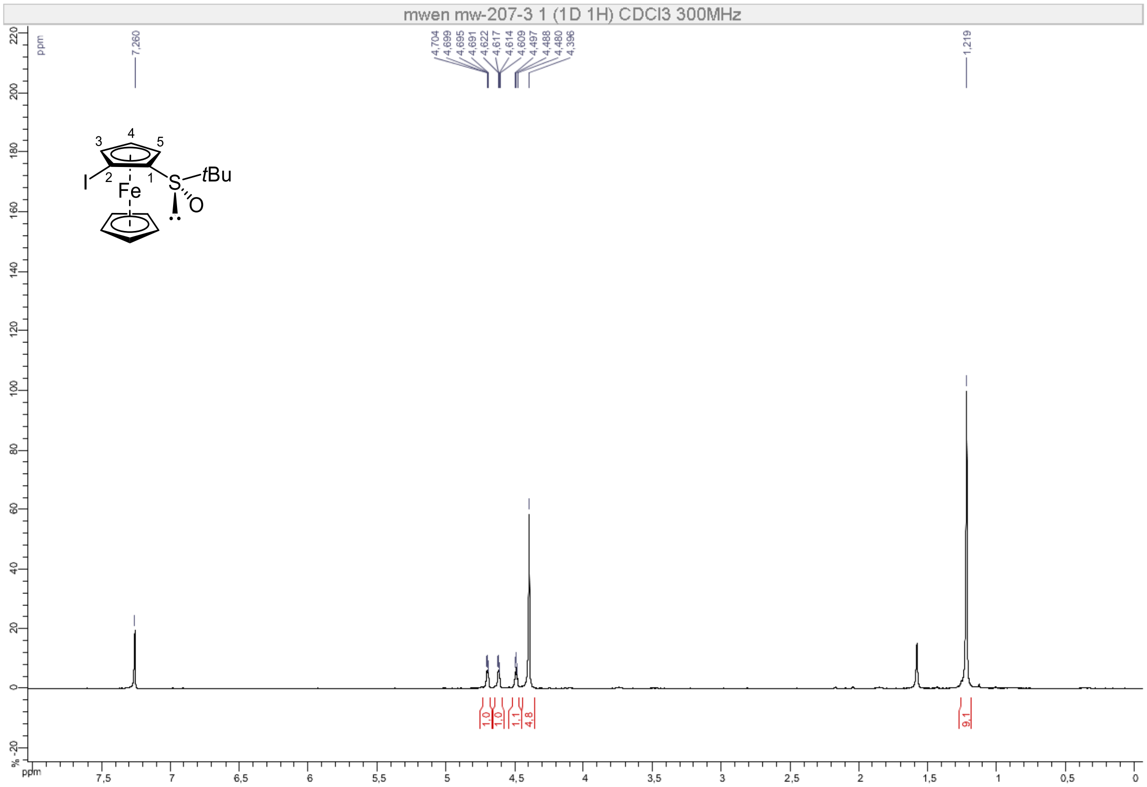

(S,S_P)-*S-tert*-Butyl-2-methyl-5-(trimethylsilyl)ferrocenesulfoxide (S,S_P-2ad)

^{1}H NMR (500 MHz, $CDCl_3$)

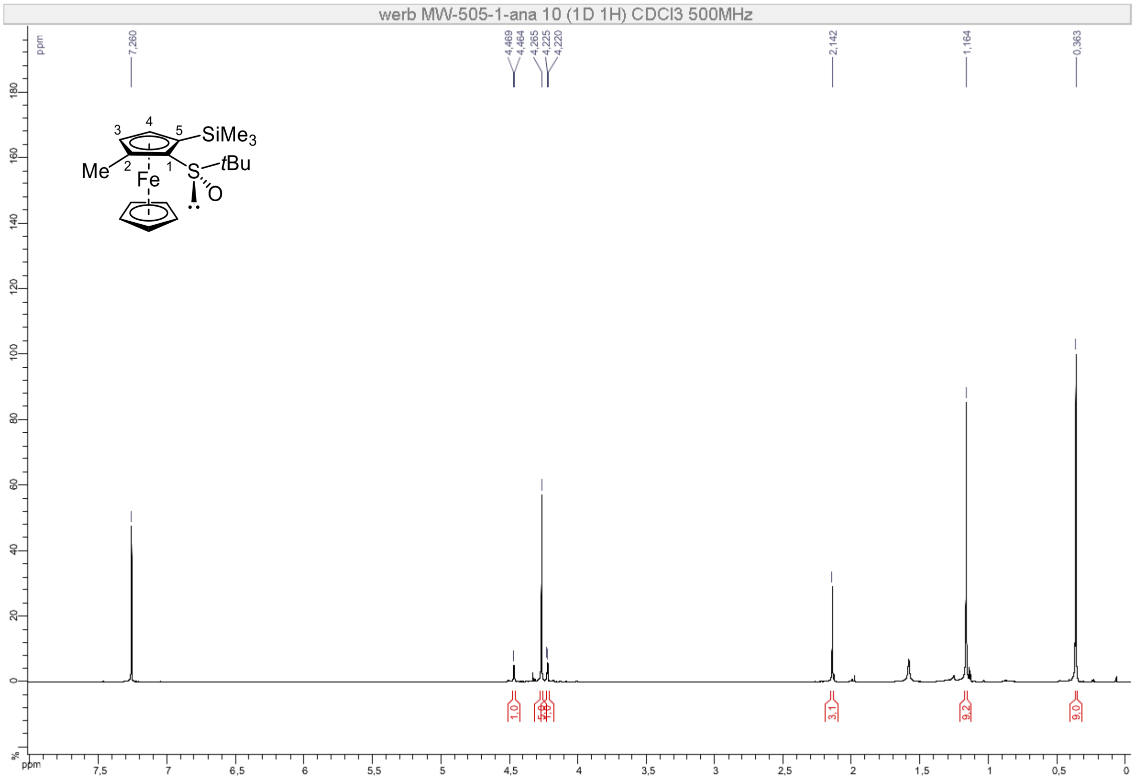

^{13}C NMR (126 MHz, $CDCl_3$)

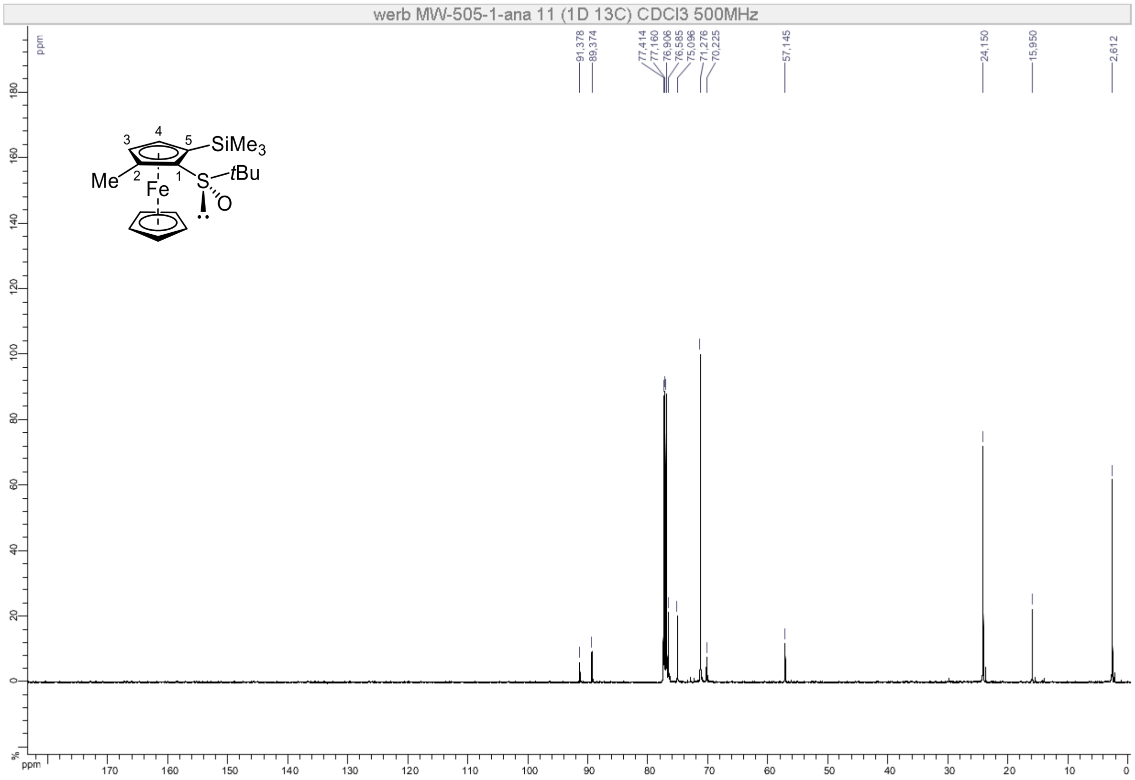

COSY (500 MHz, $CDCl_3$)

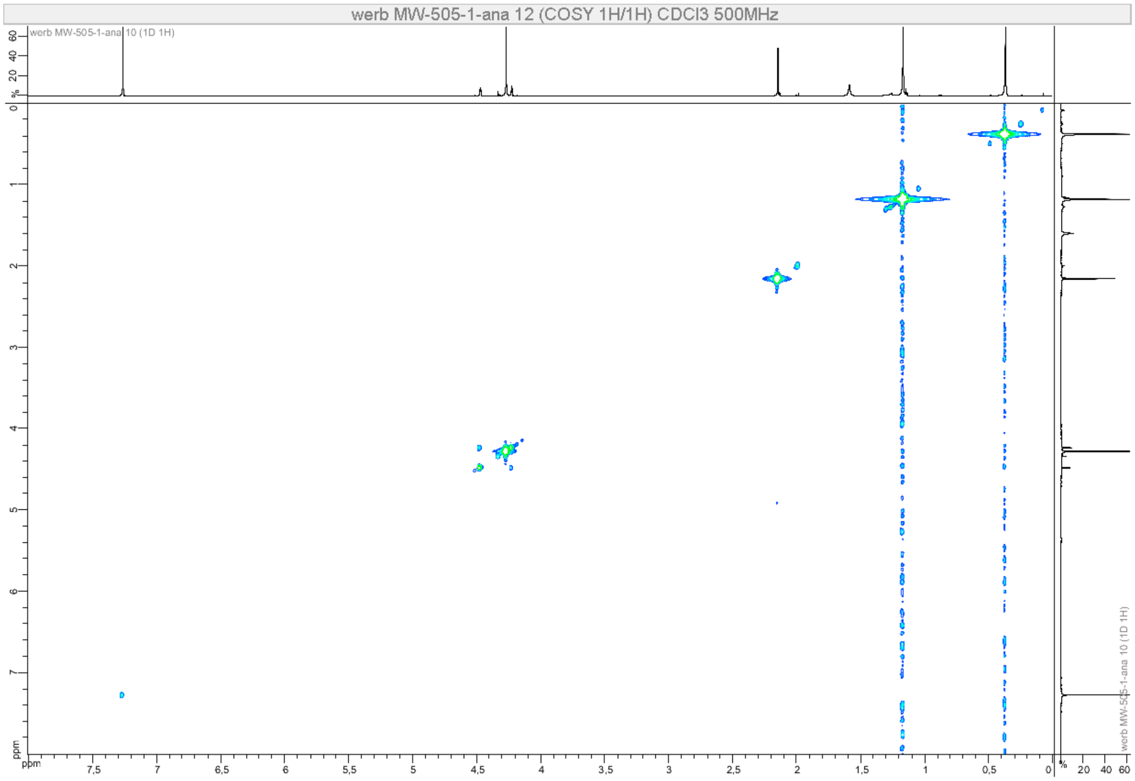

HSQC (500 MHz, $CDCl_3$)

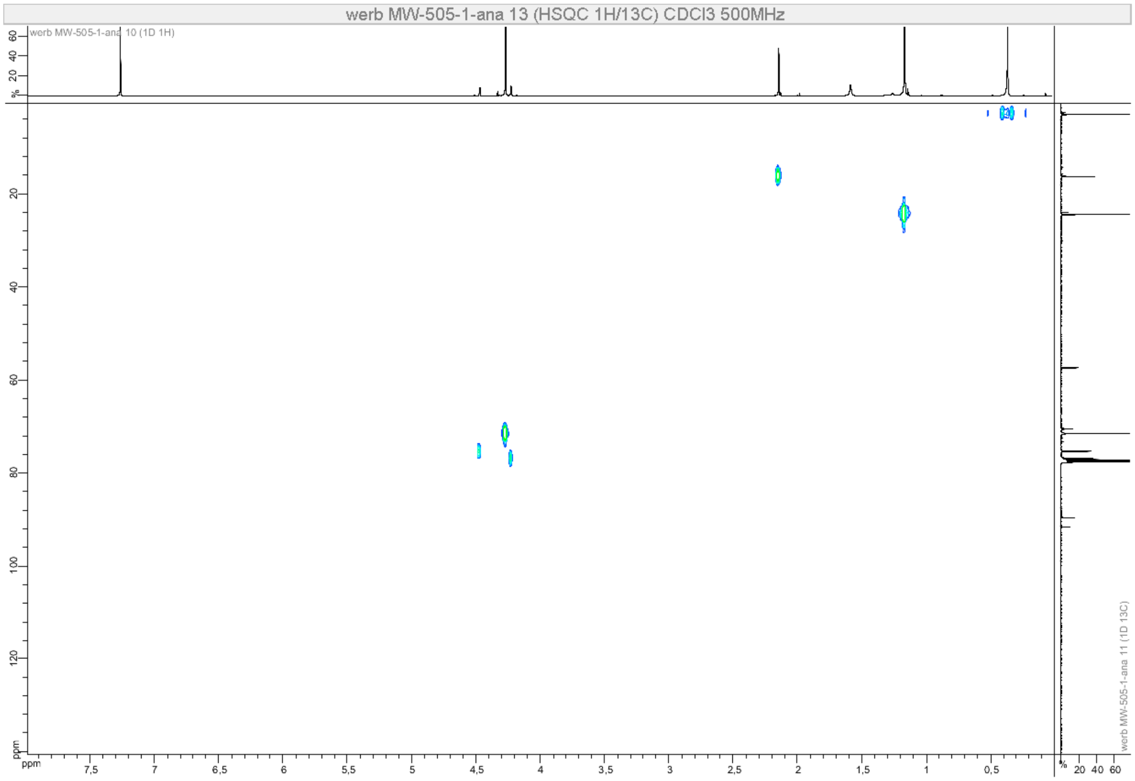

HMBC (500 MHz, $CDCl_3$)

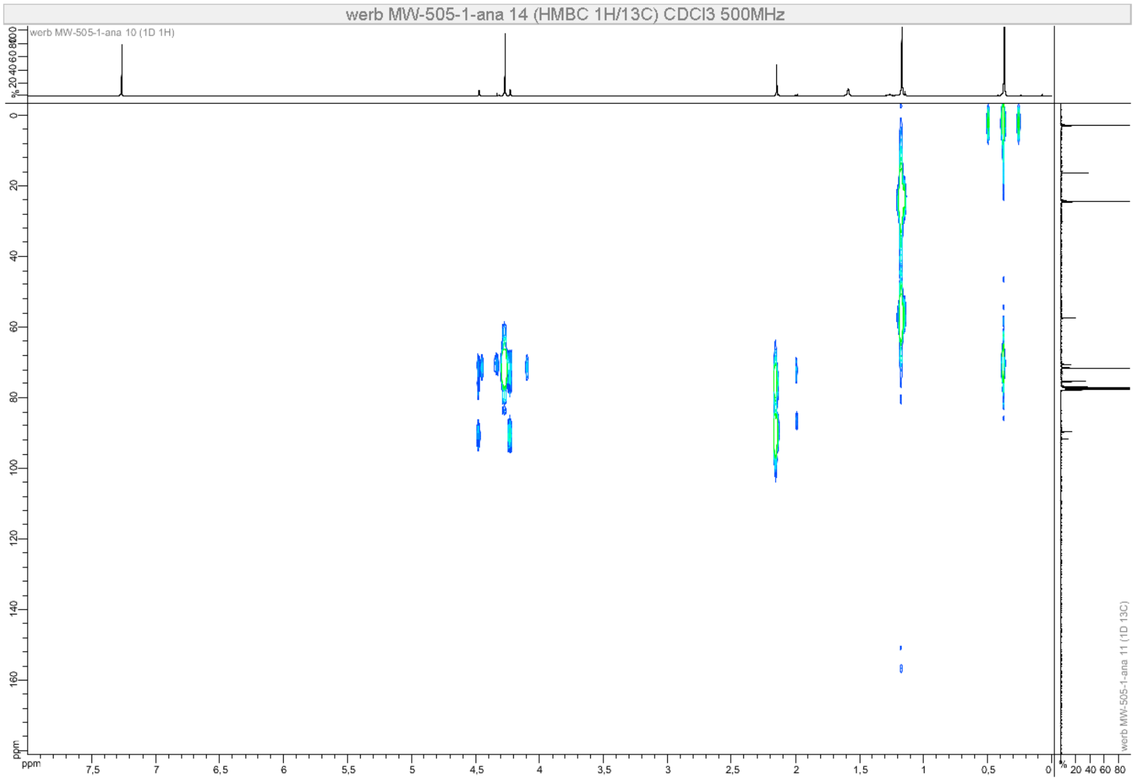

NOESY (500 MHz, $CDCl_3$)

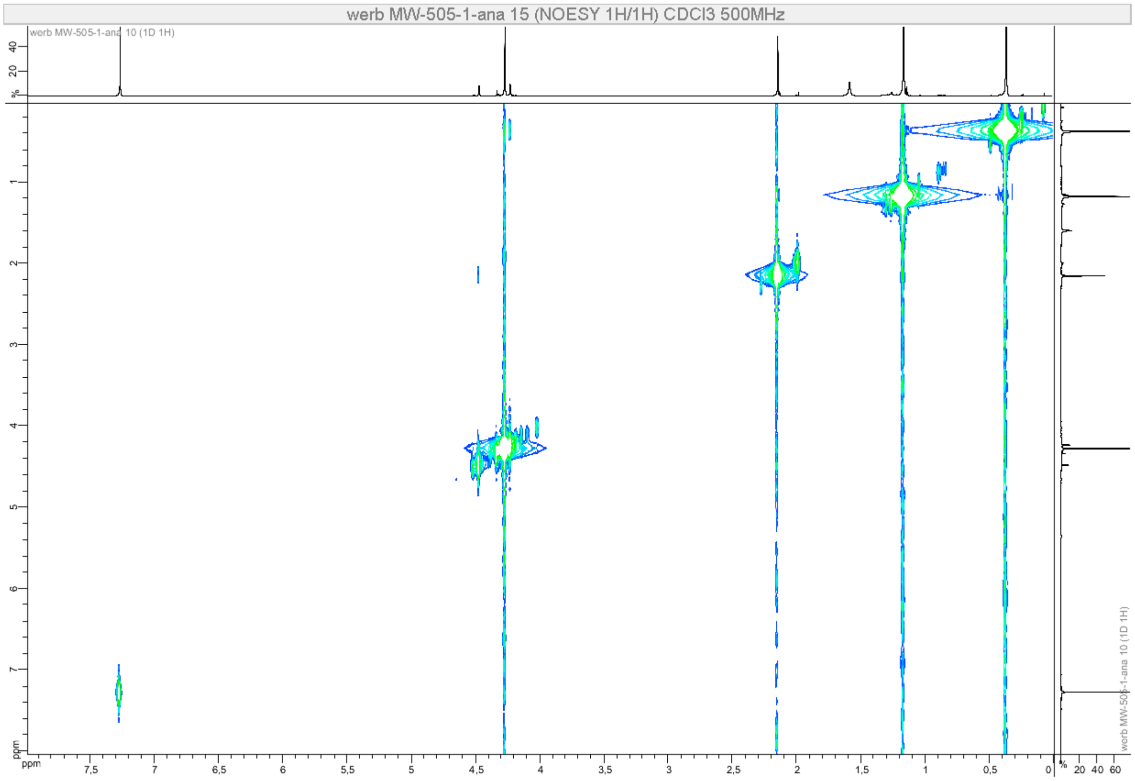

(*S*,*R*P)-*S*-*tert*-Butyl-2-methylferrocenesulfoxide (*S*,*R*P-1d)

^{1}H NMR (500 MHz, $CDCl_3$)

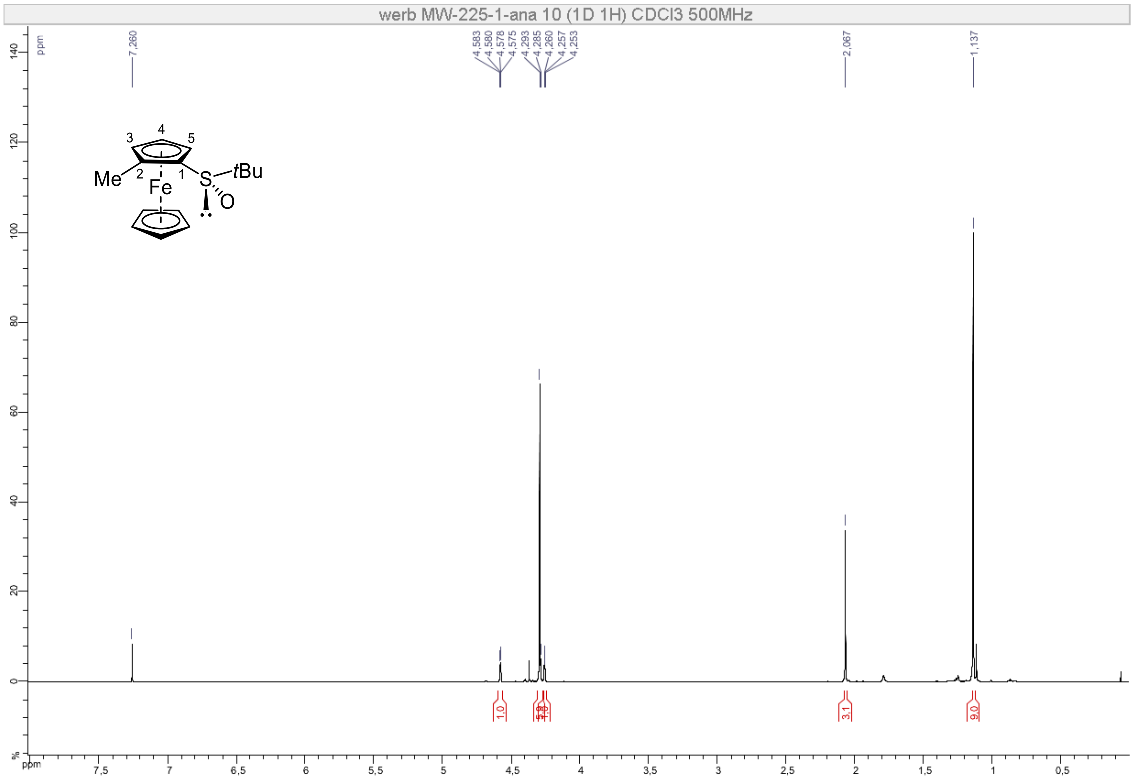

^{13}C NMR (126 MHz, $CDCl_3$)

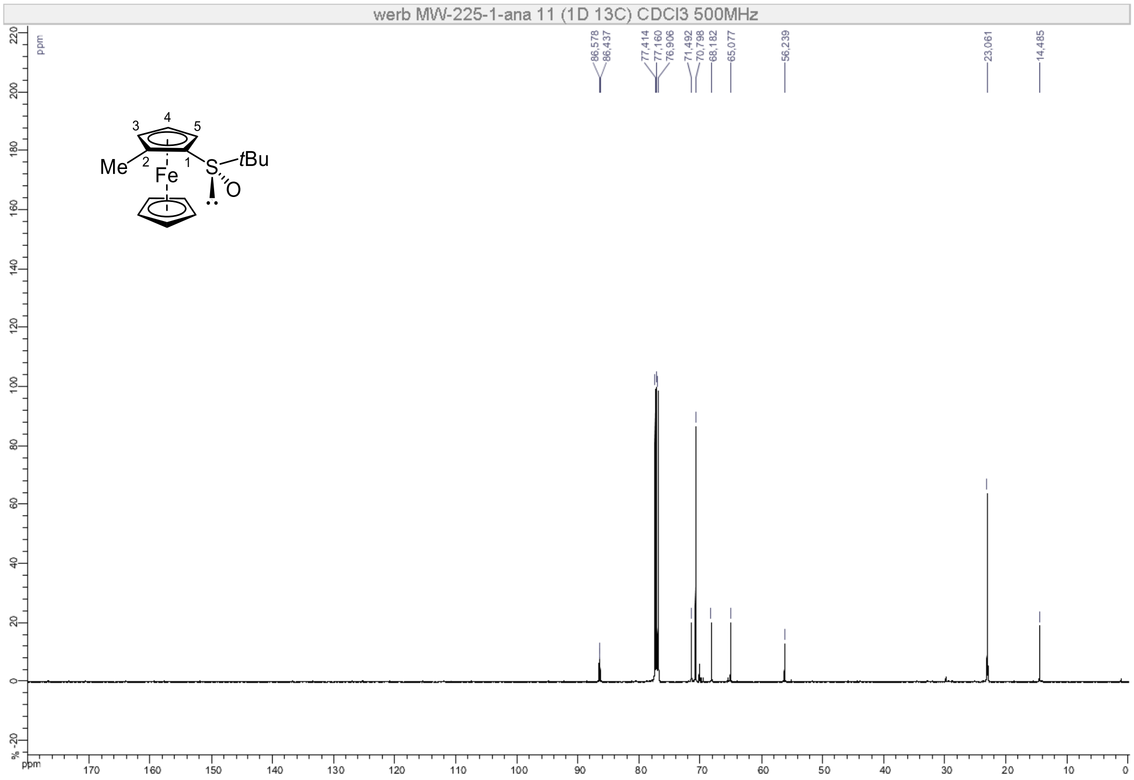

DEPT 135 (126 MHz, $CDCl_3$)

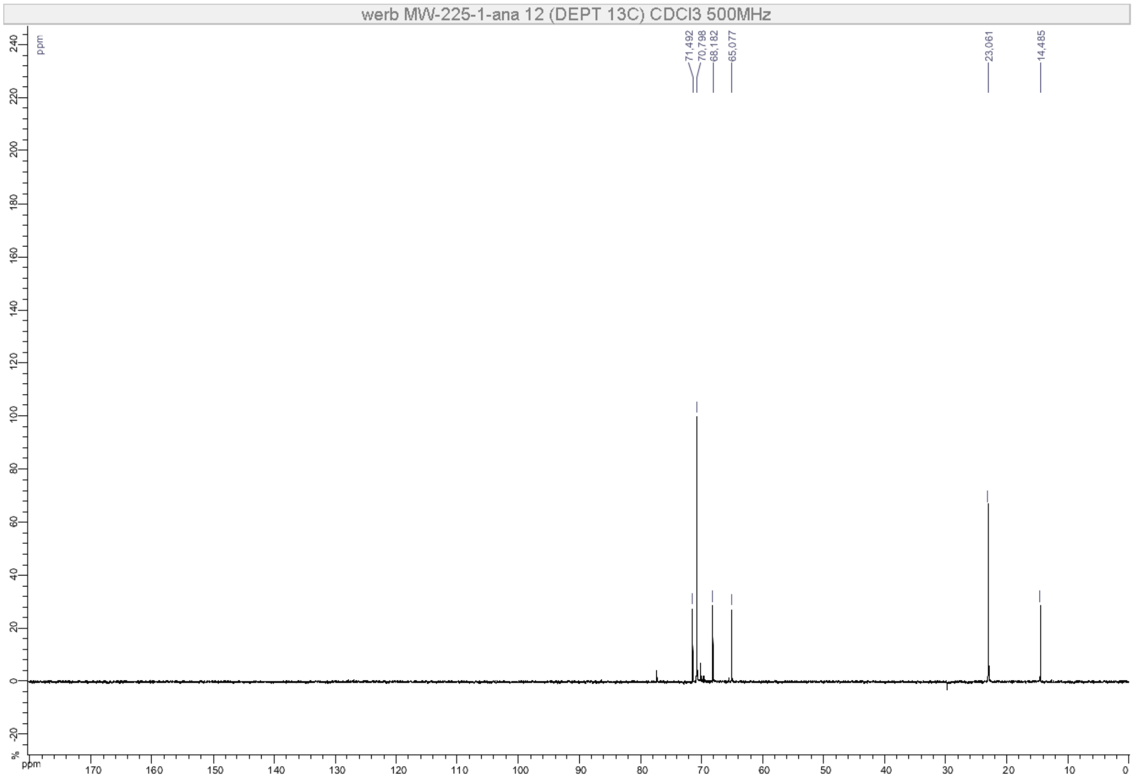

COSY (500 MHz, $CDCl_3$)

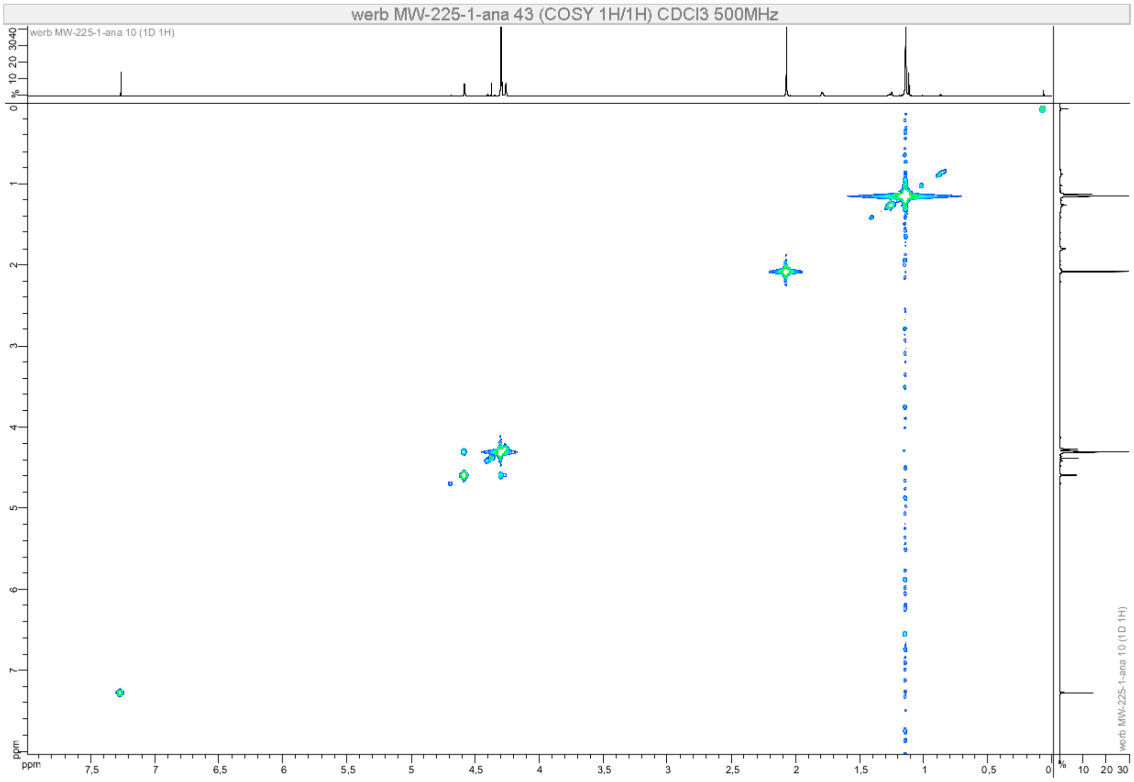

HSQC (500 MHz, $CDCl_3$)

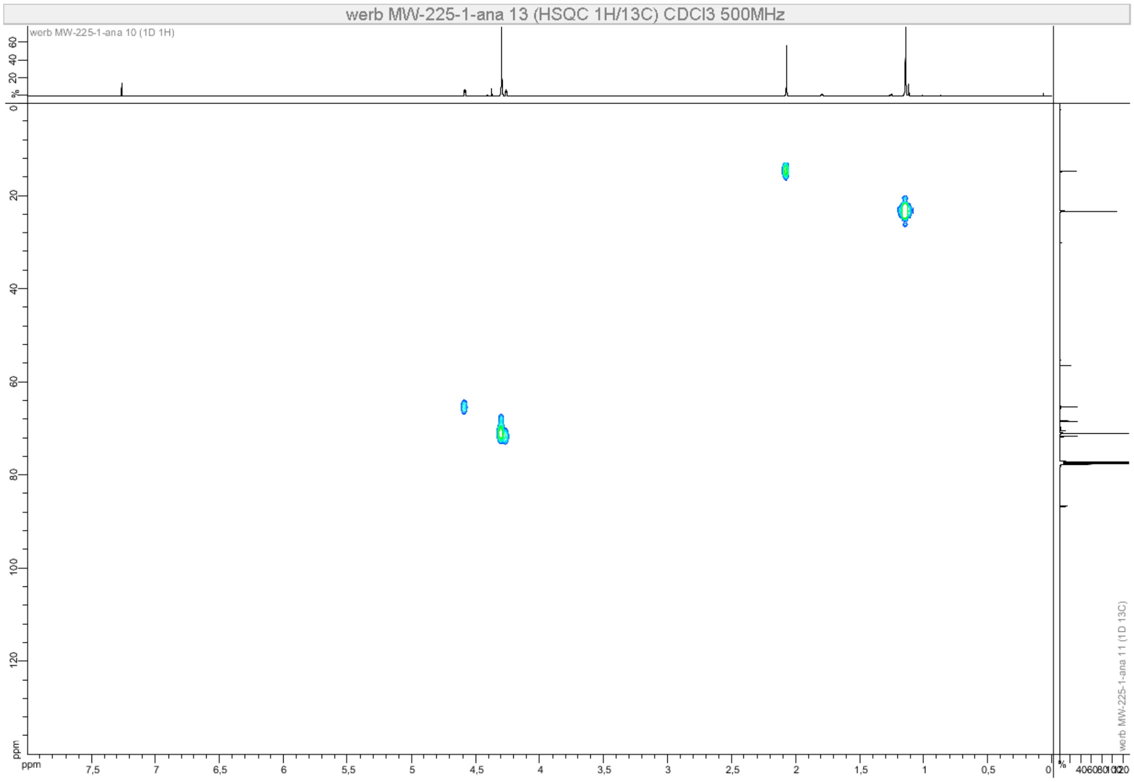

HMBC (500 MHz, $CDCl_3$)

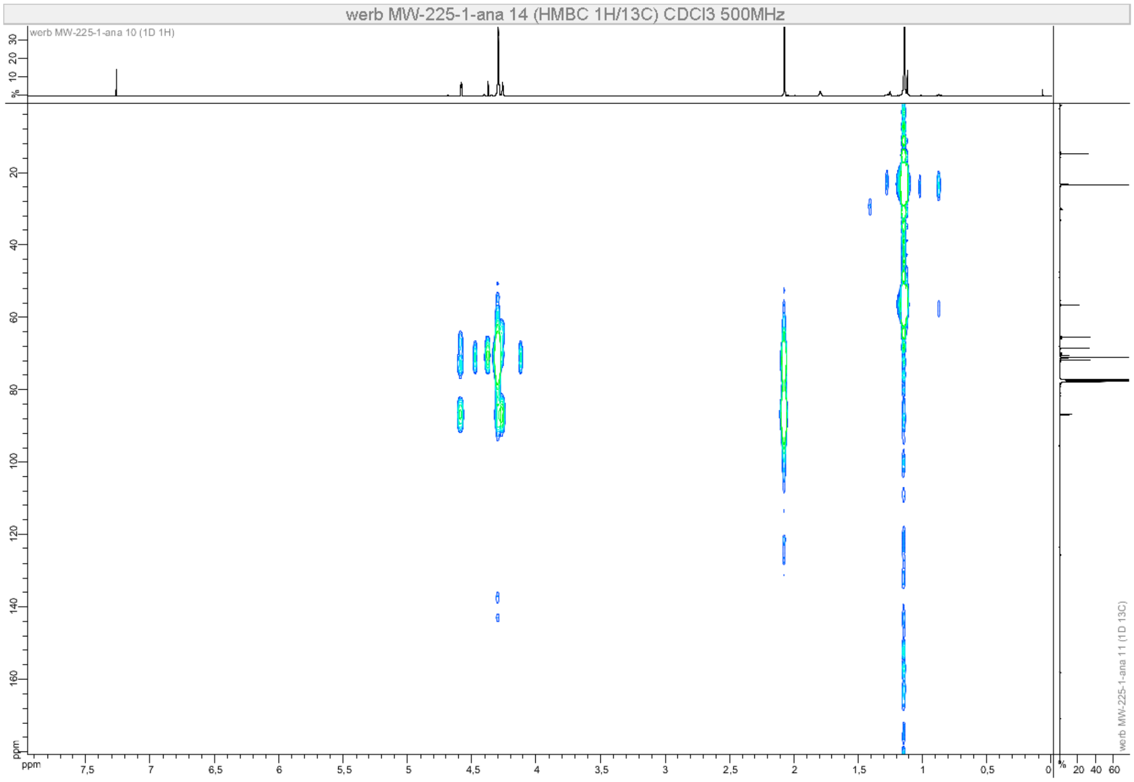

NOESY (500 MHz, $CDCl_3$)

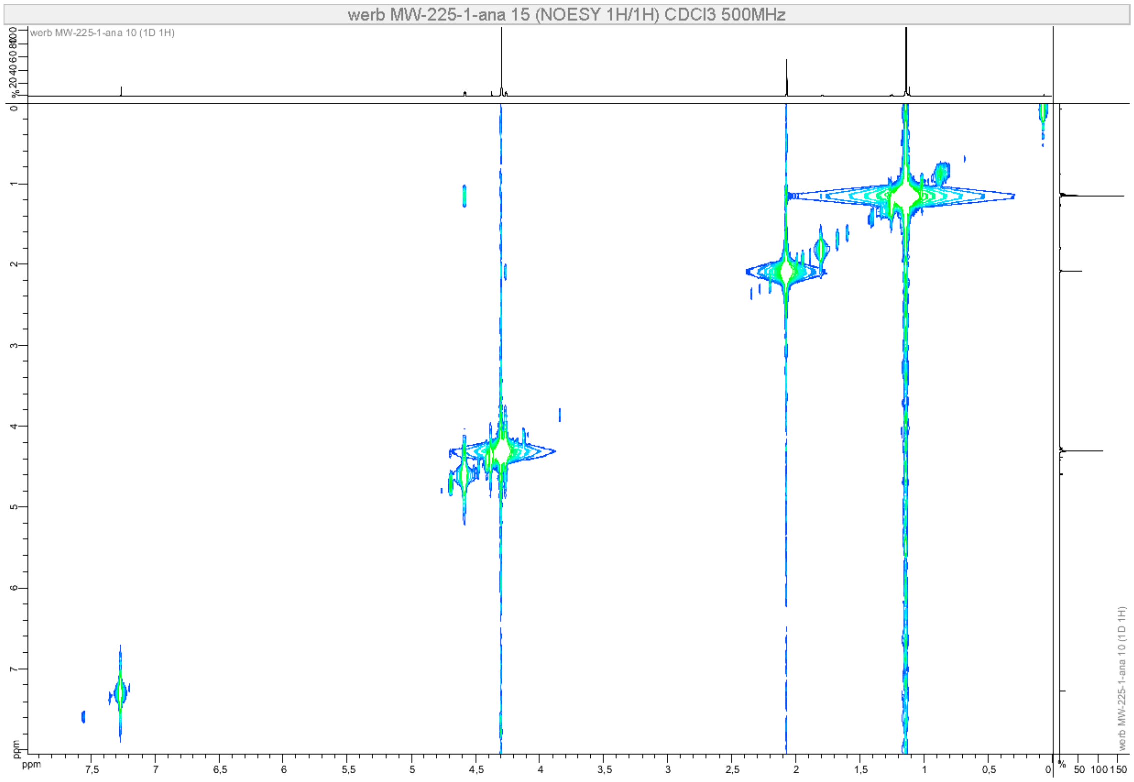

(*S*,*R*P)-*S*-*tert*-Butyl-2-(diphenylphosphino)-5-(trimethylsilyl)ferrocenesulfoxide (*S*,*R*P-2ae)

^{1}H NMR (500 MHz, $CDCl_3$)

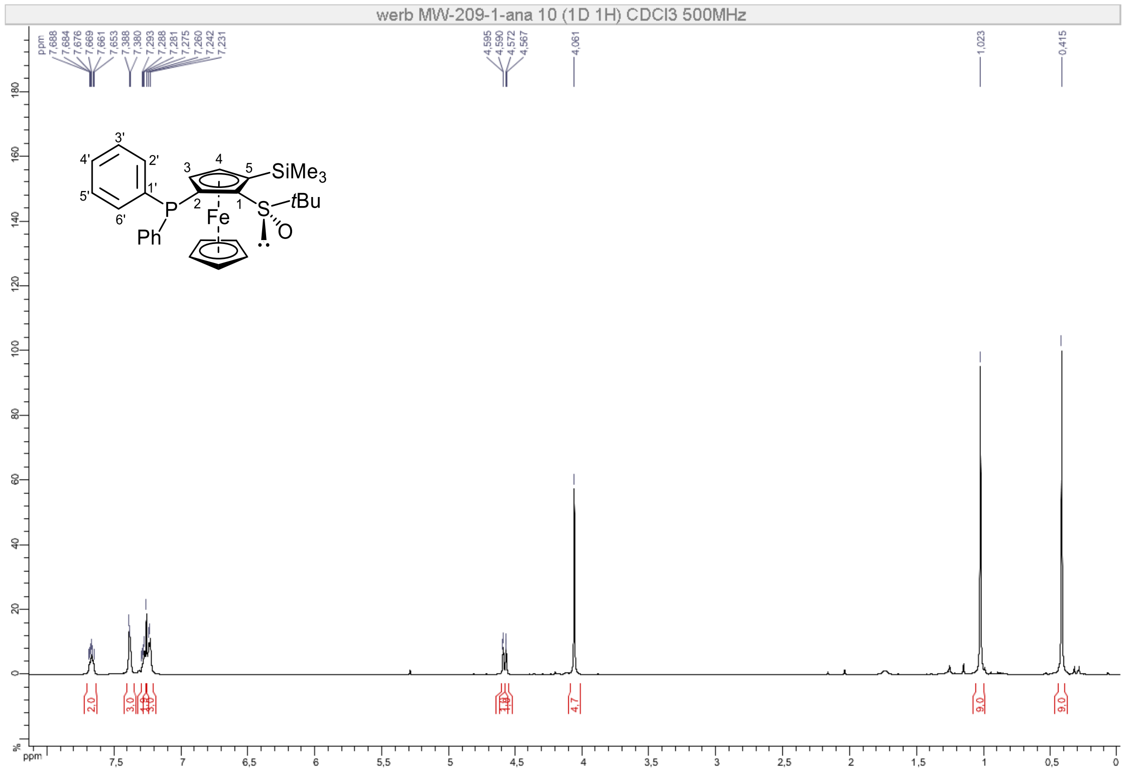

^{13}C NMR (126 MHz, $CDCl_3$)

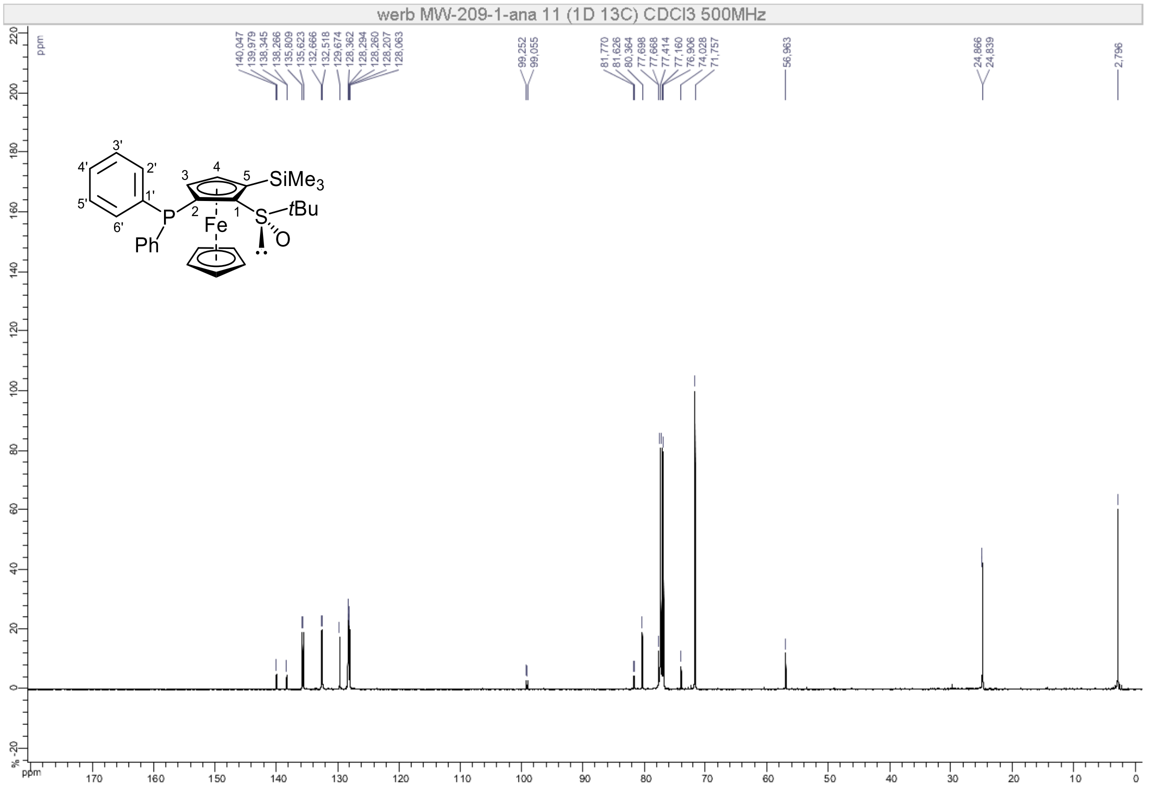

DEPT 135 (126 MHz, $CDCl_3$)

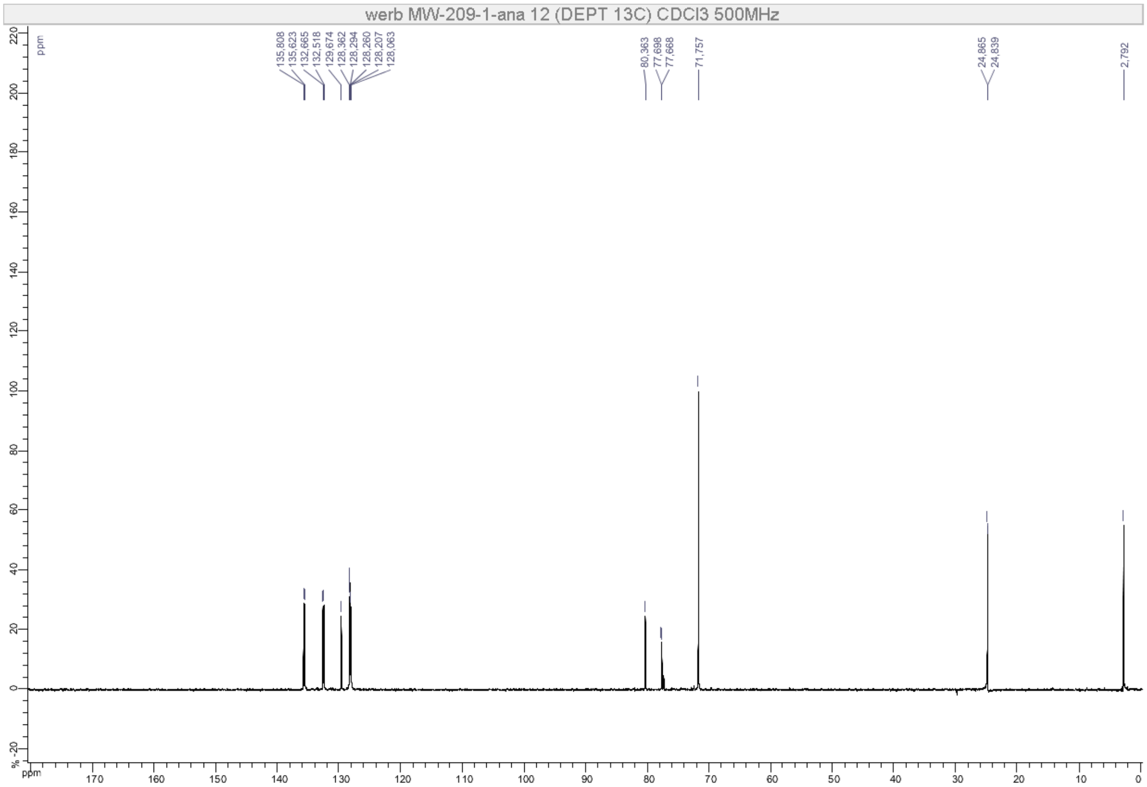

COSY (500 MHz, $CDCl_3$)

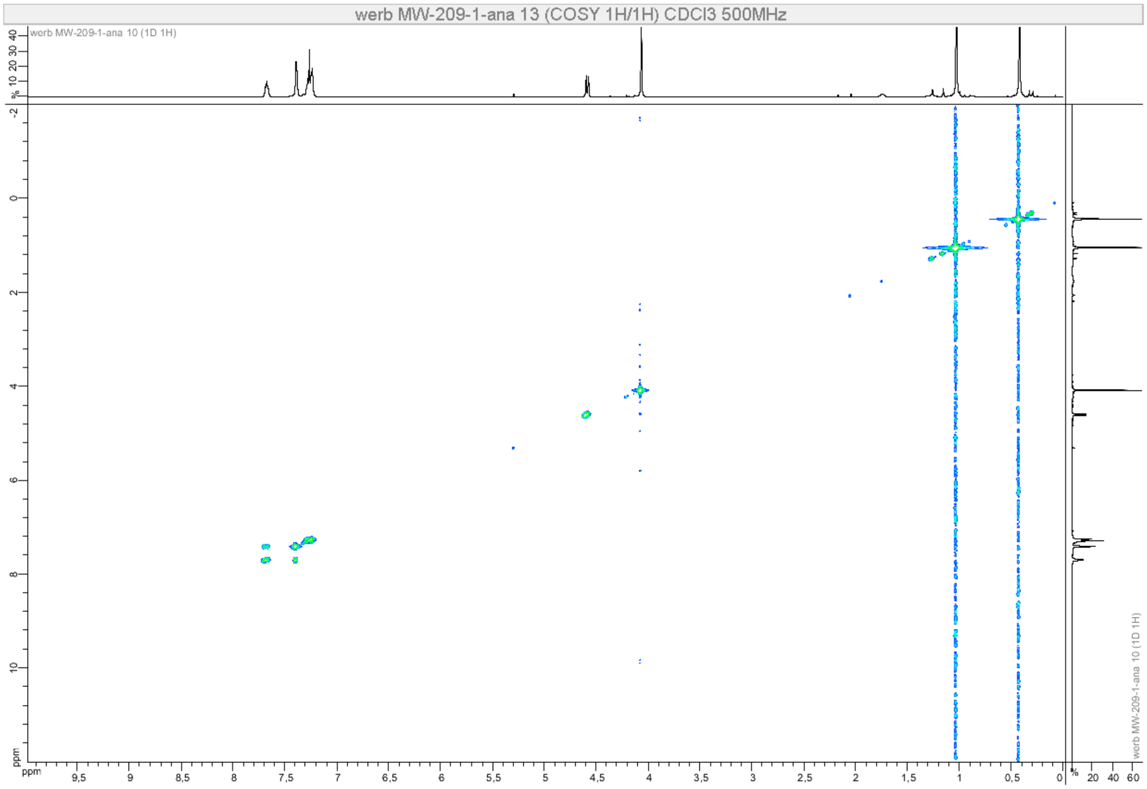

HSQC (500 MHz, $CDCl_3$)

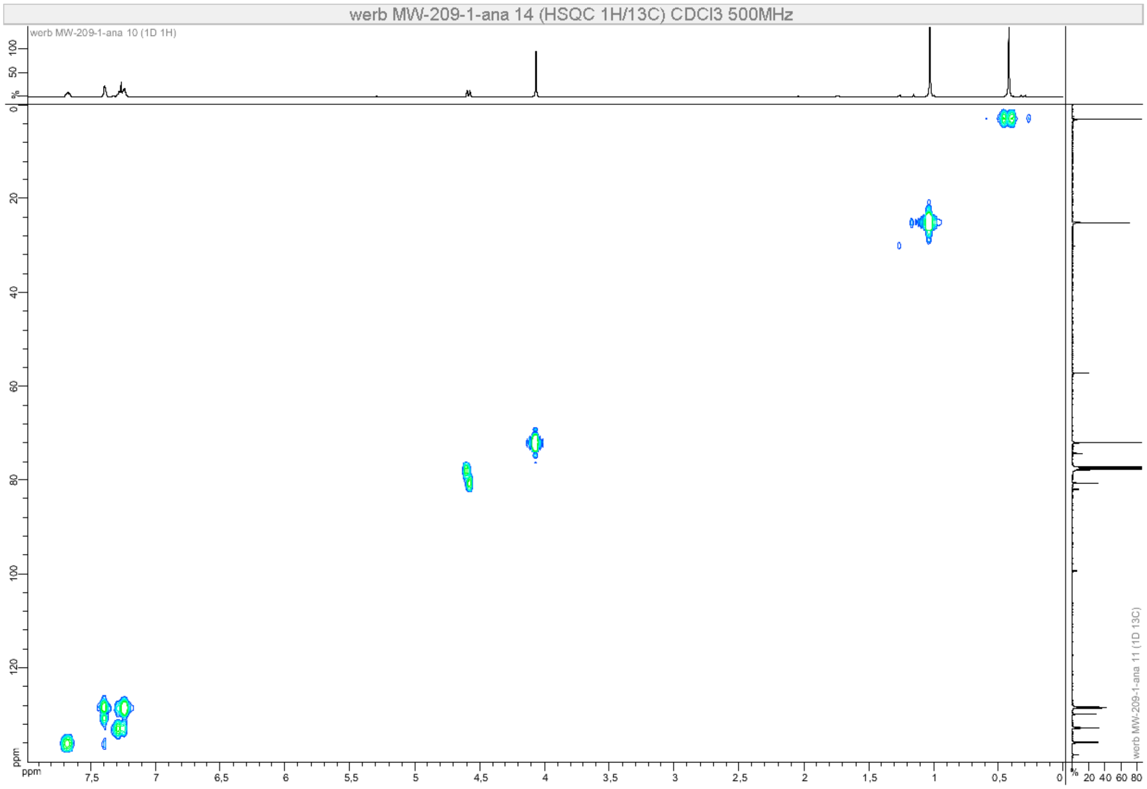

HMBC (500 MHz, $CDCl_3$)

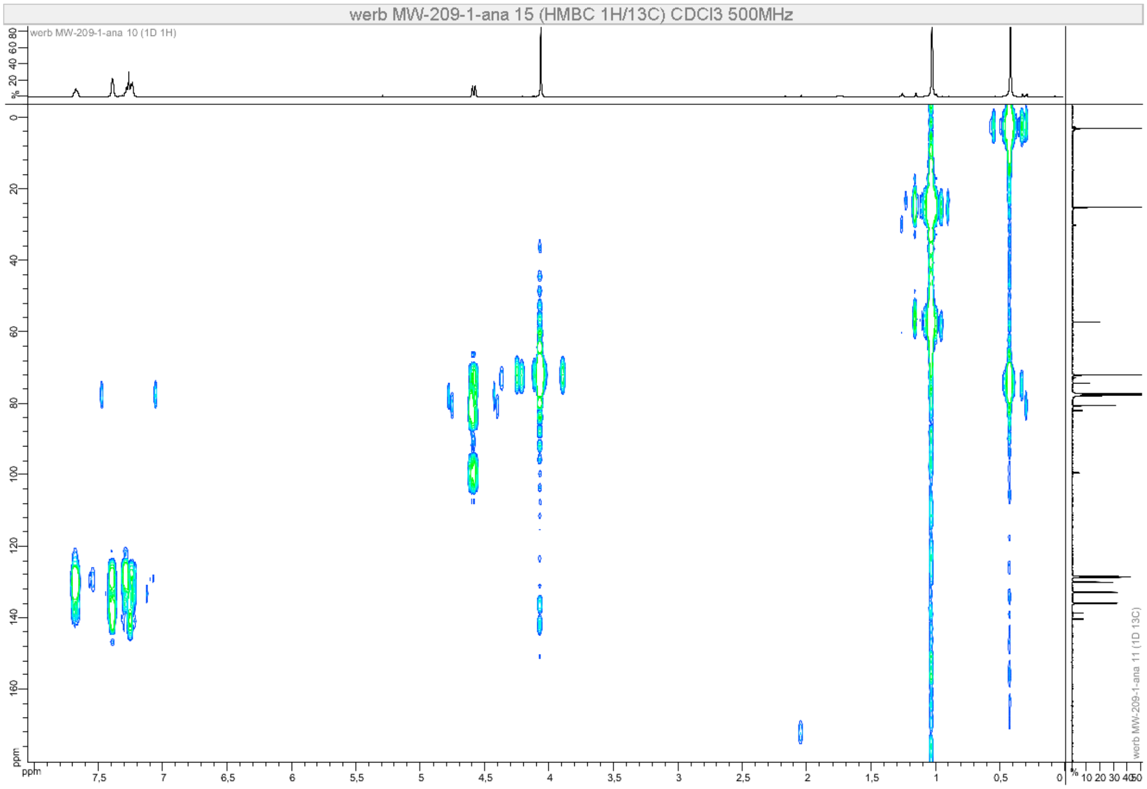

NOESY (500 MHz, $CDCl_3$)

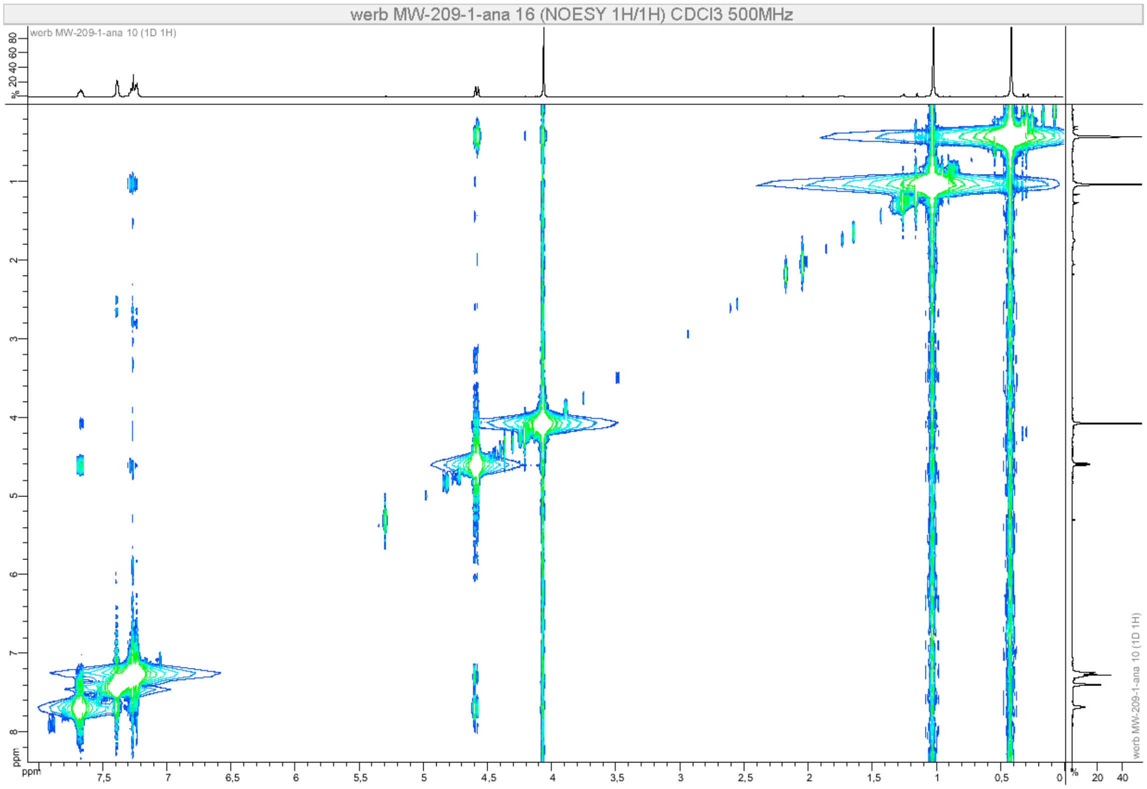

^{31}P NMR (121 MHz, $CDCl_3$)

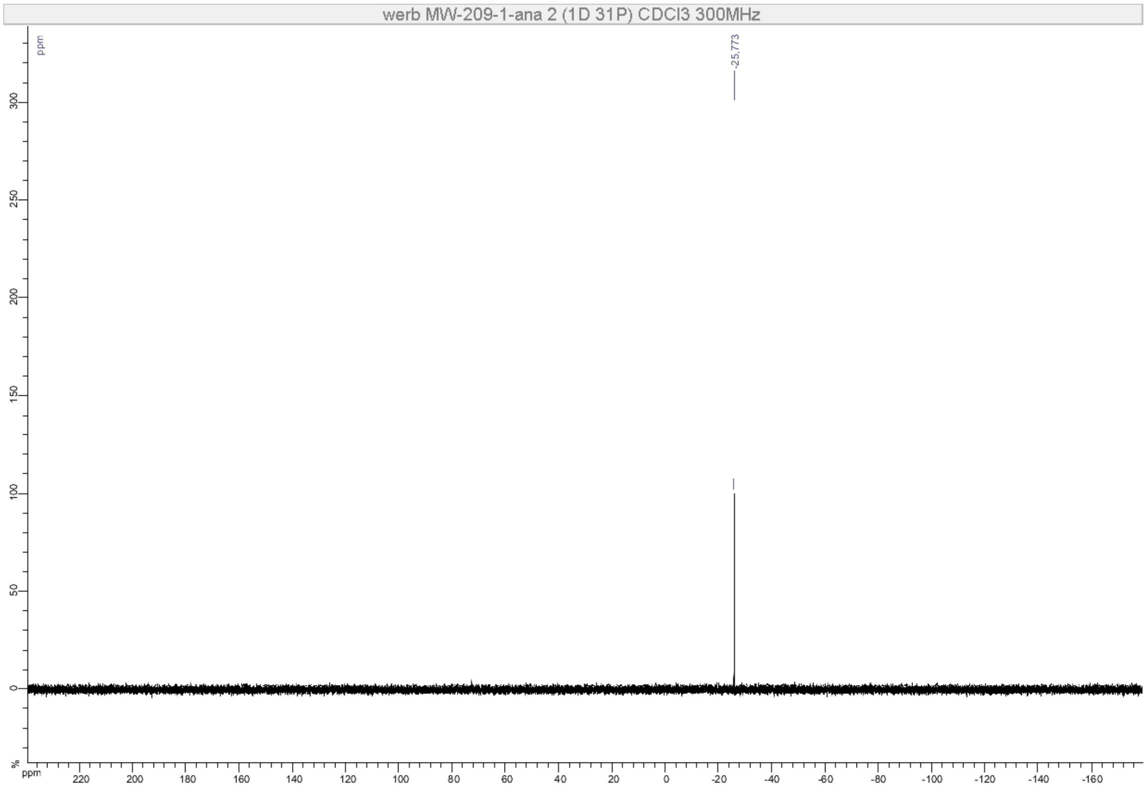

(*S*,*S*P)-*S*-*tert*-Butyl-2-[(α,α-diphenyl)hydroxymethyl]-5-(trimethylsilyl)ferrocenesulfoxide (*S*,*S*P-2ag)

^{1}H NMR (300 MHz, $CDCl_3$)

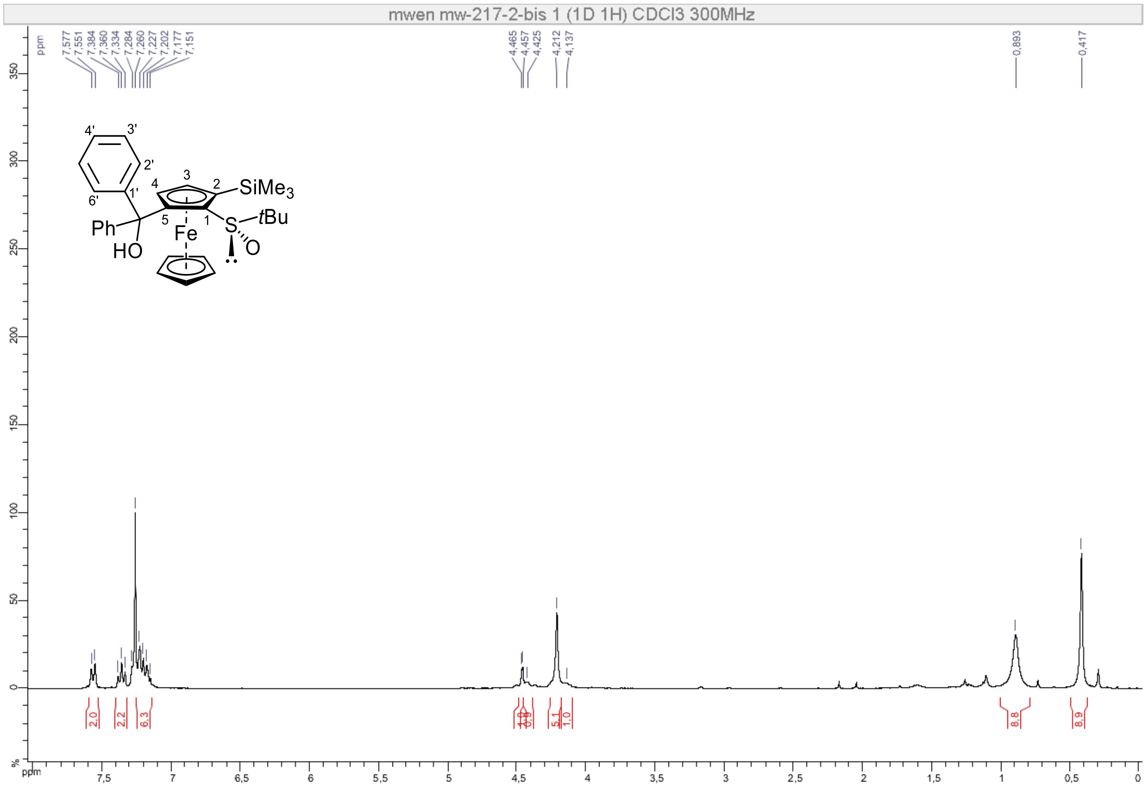

(*S*,*S*P)-*S*-*tert*-Butyl-2-(dimethylaminomethyl)-5-(trimethylsilyl)ferrocenesulfoxide (*S*,*S*P-2ah)

^{1}H NMR (500 MHz, $CDCl_3$)

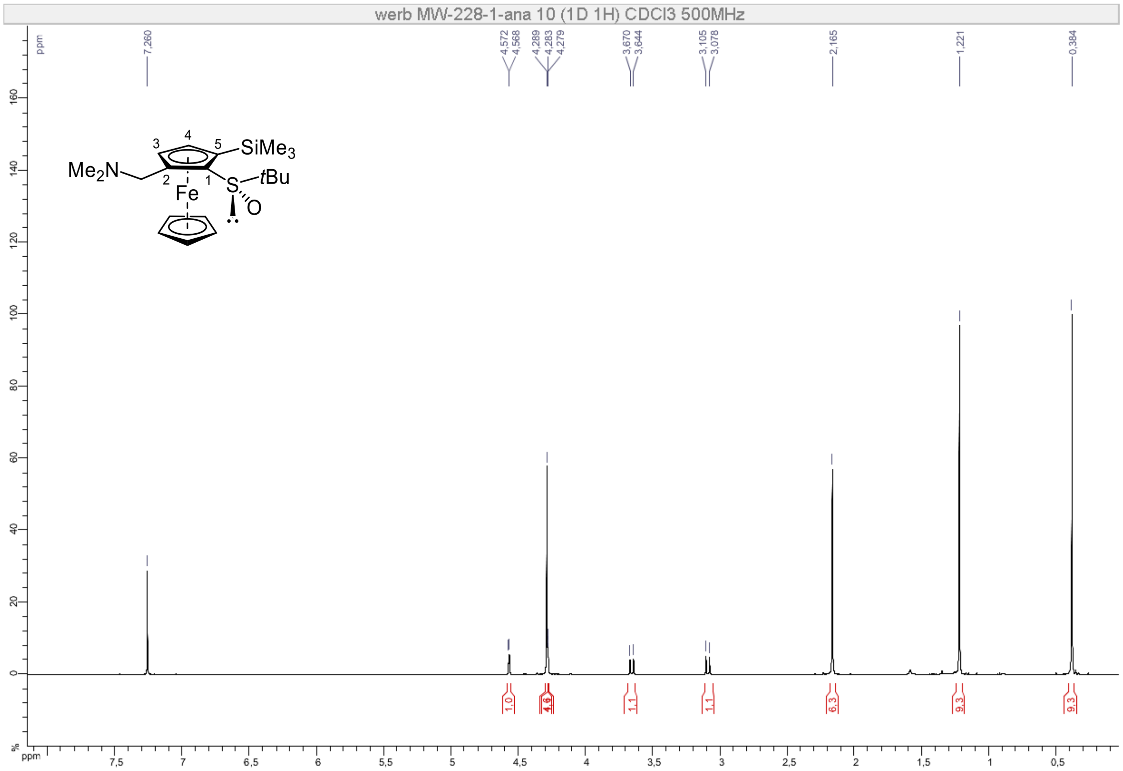

^{13}C NMR (126 MHz, $CDCl_3$)

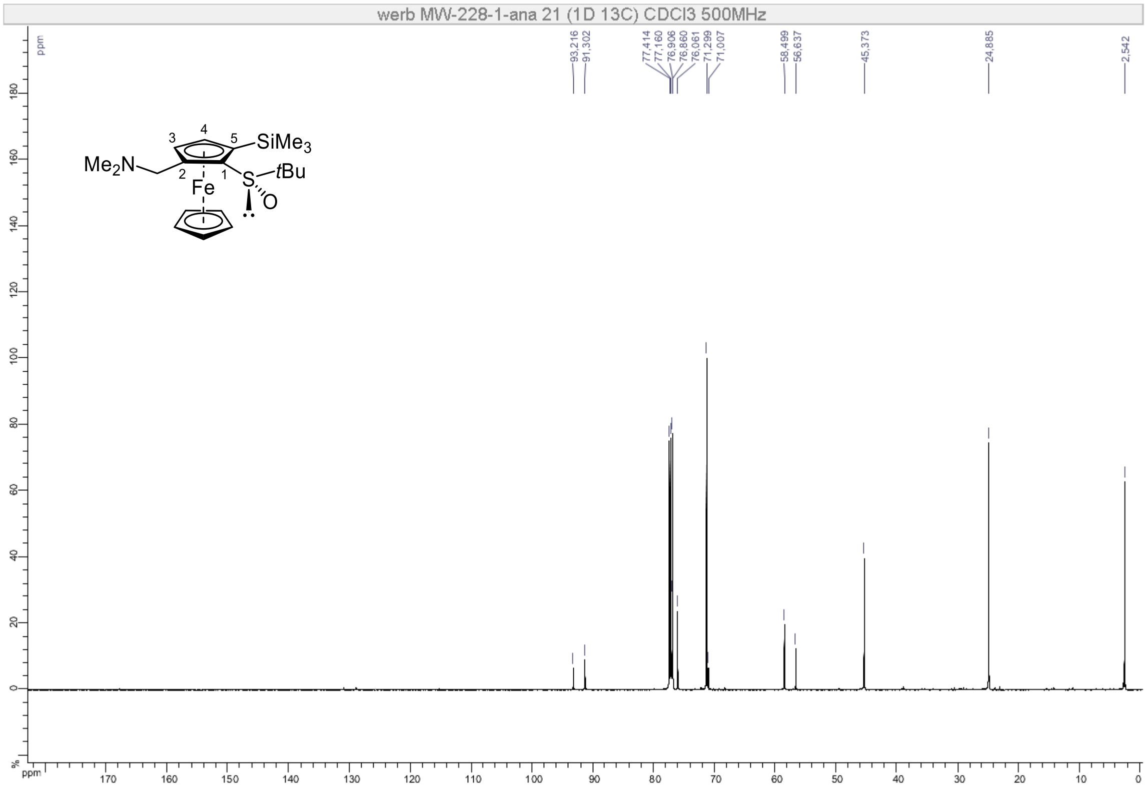

COSY (500 MHz, $CDCl_3$)

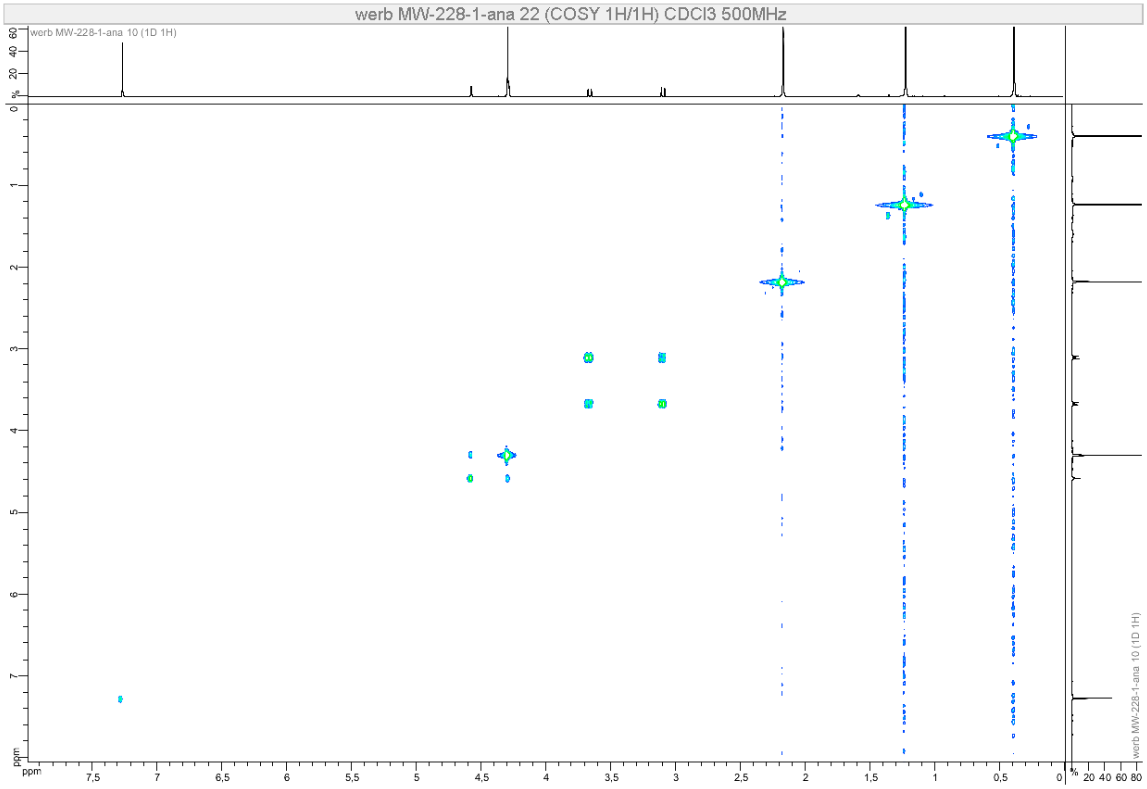

HSQC (500 MHz, $CDCl_3$)

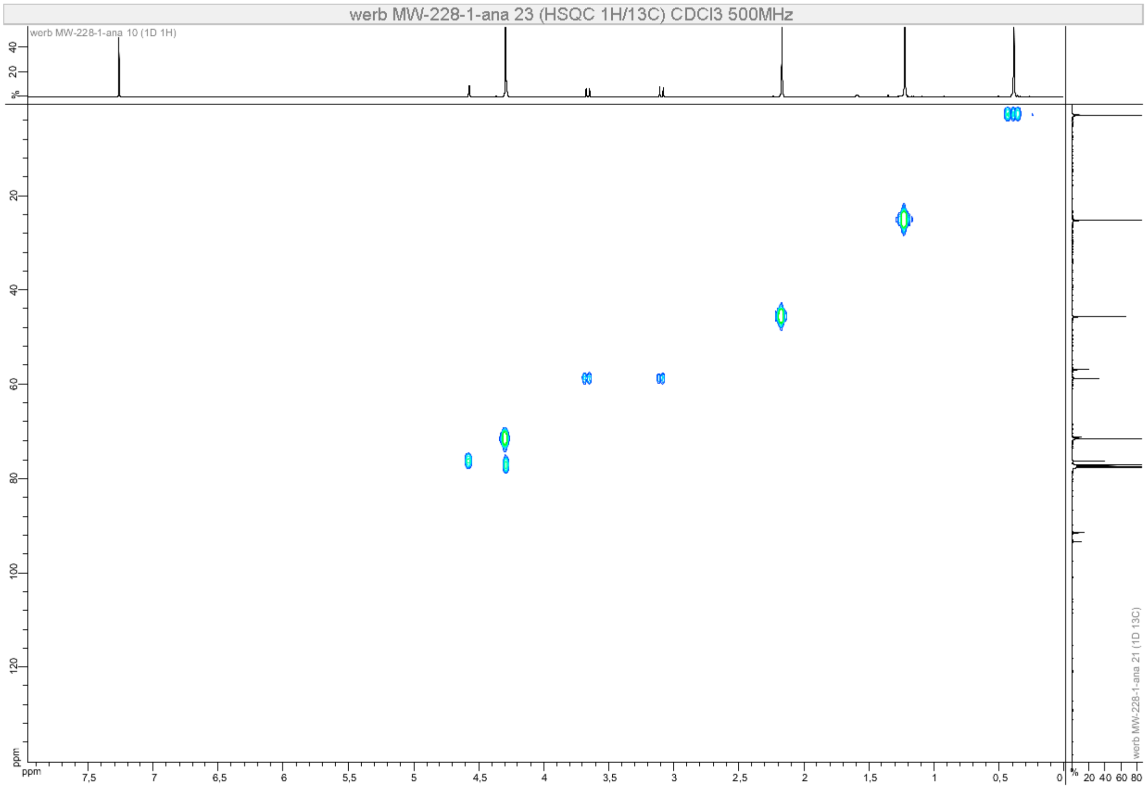

HMBC (500 MHz, $CDCl_3$)

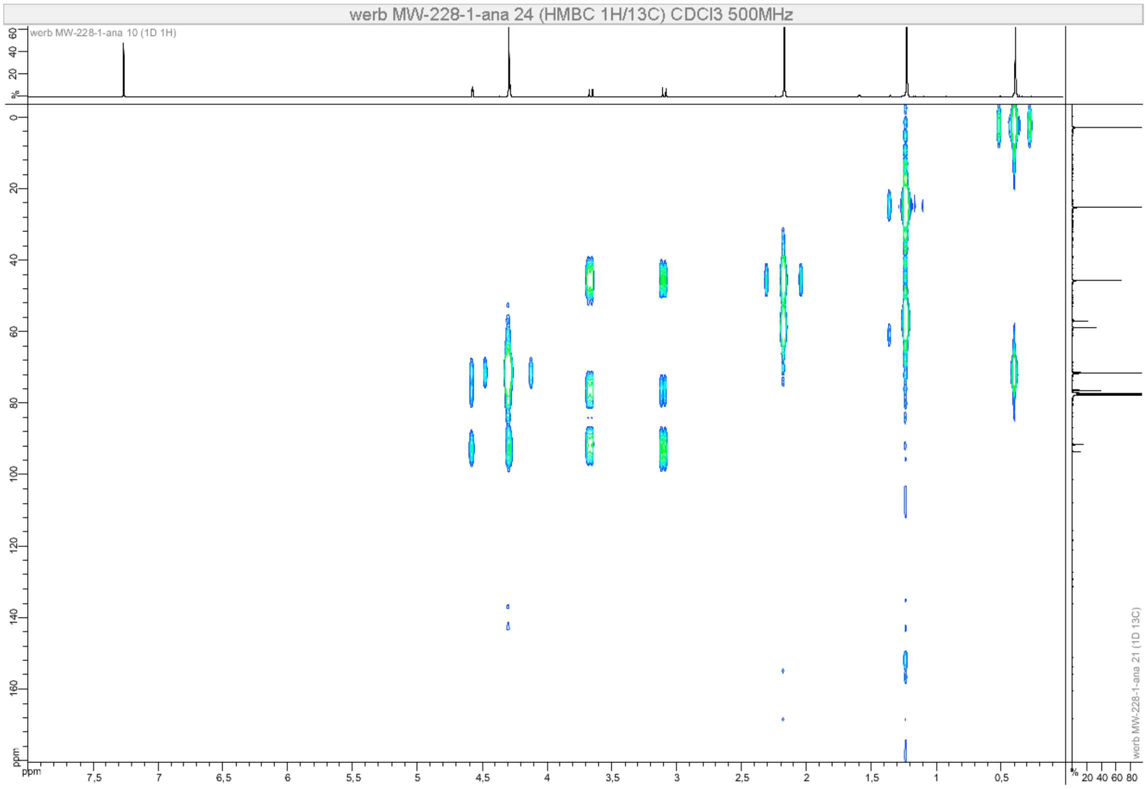

NOESY (500 MHz, $CDCl_3$)

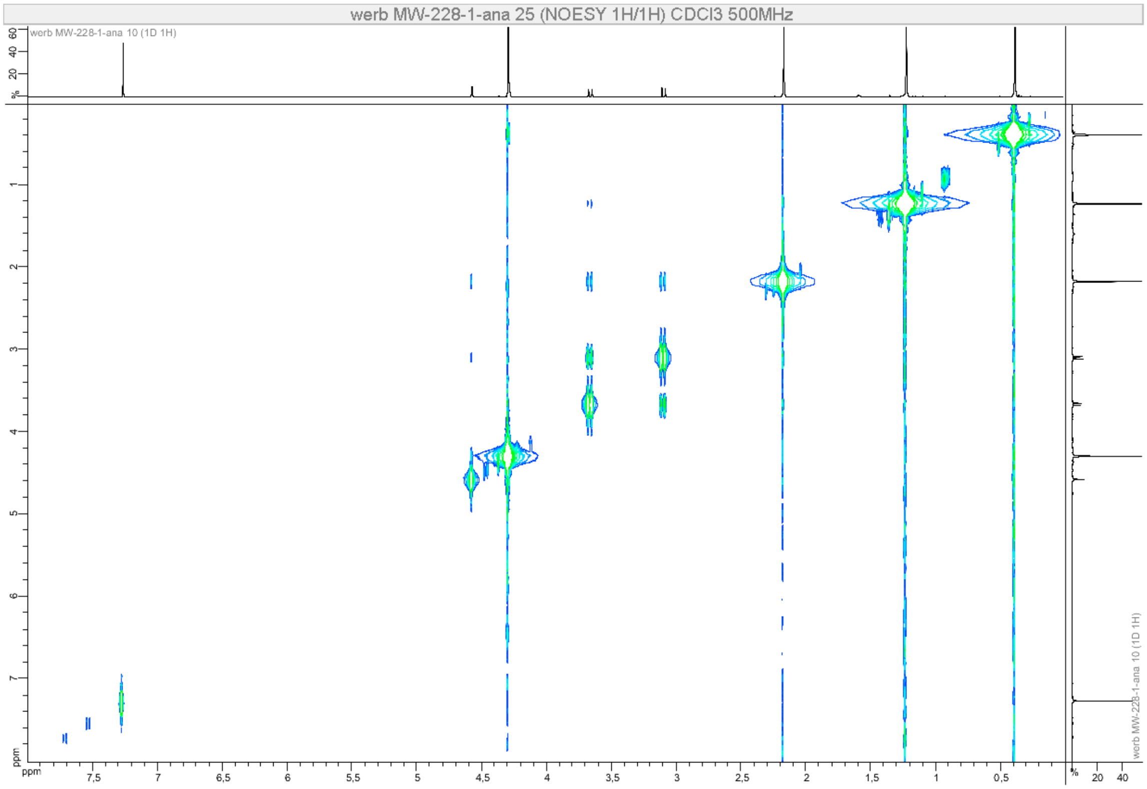

(*S*,*S*P)-*S*-*tert*-Butyl-2-formyl-5-(trimethylsilyl)ferrocenesulfoxide (*S*,*S*P-2ah')

^{1}H NMR (300 MHz, $CDCl_3$)

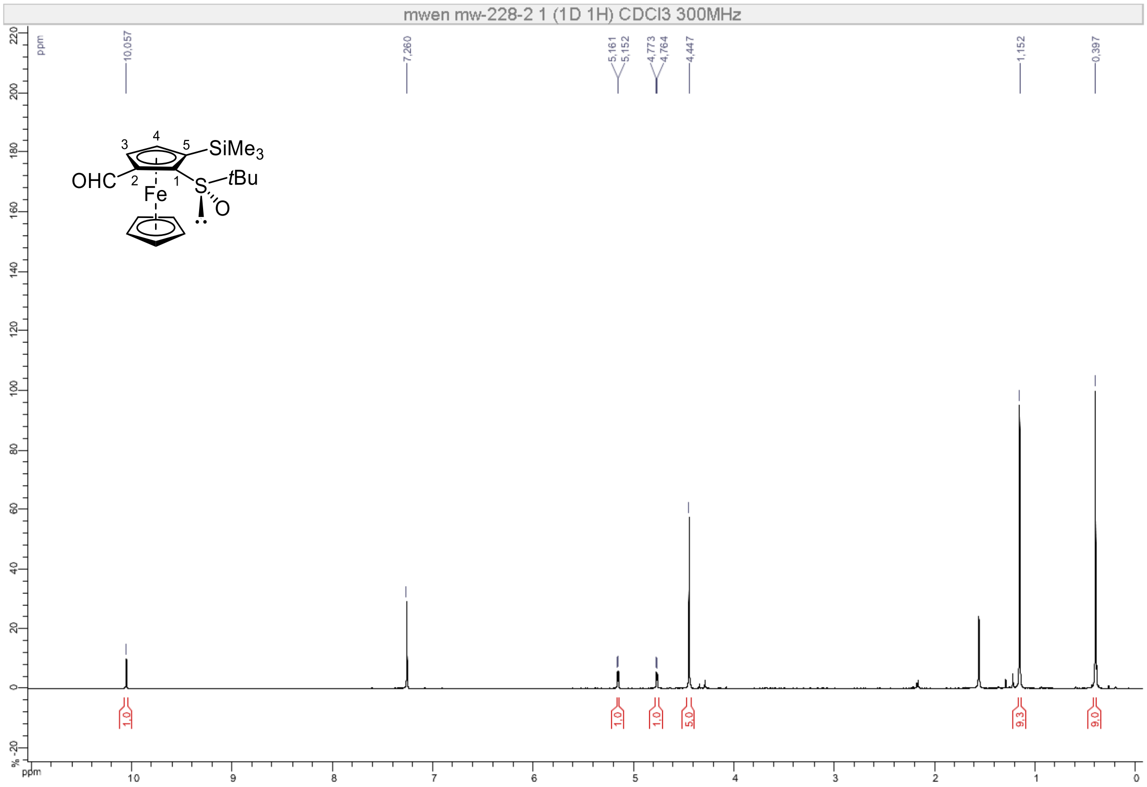

(*S*,*R*P)-*S*-*tert*-Butyl-2-(dimethylaminomethyl)ferrocenesulfoxide (*S*,*R*P-1h)

^{1}H NMR (500 MHz, $CDCl_3$)

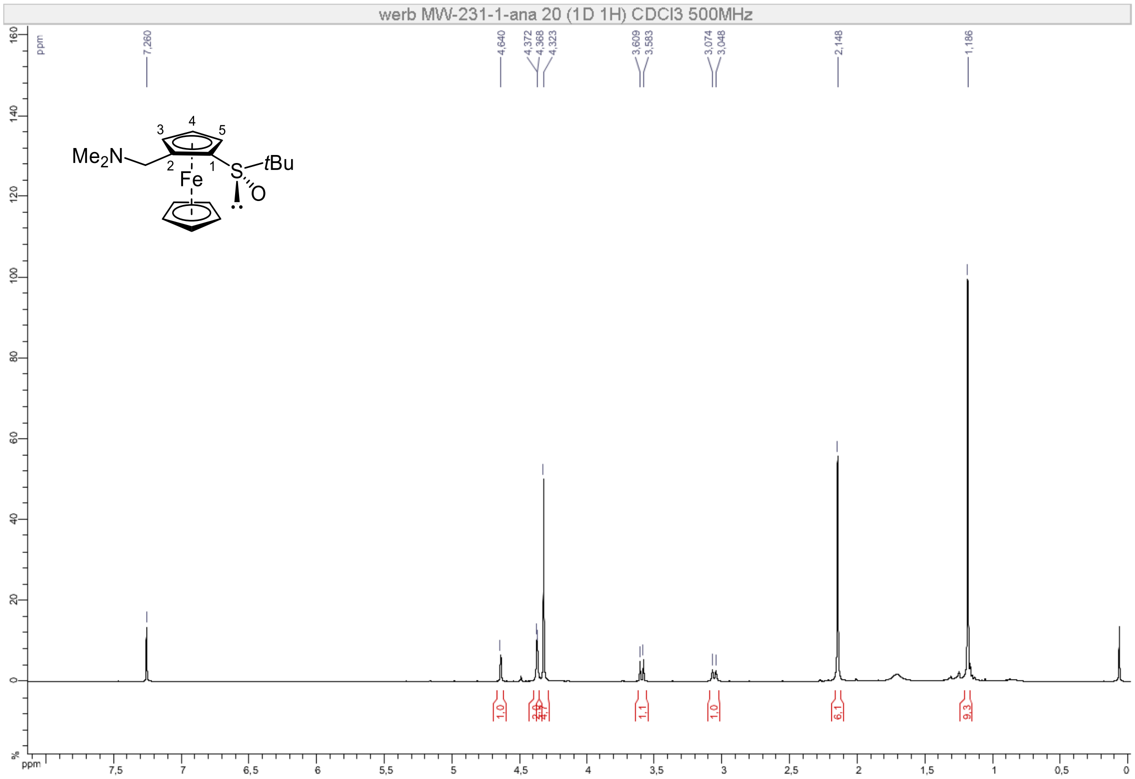

^{13}C NMR (126 MHz, $CDCl_3$)

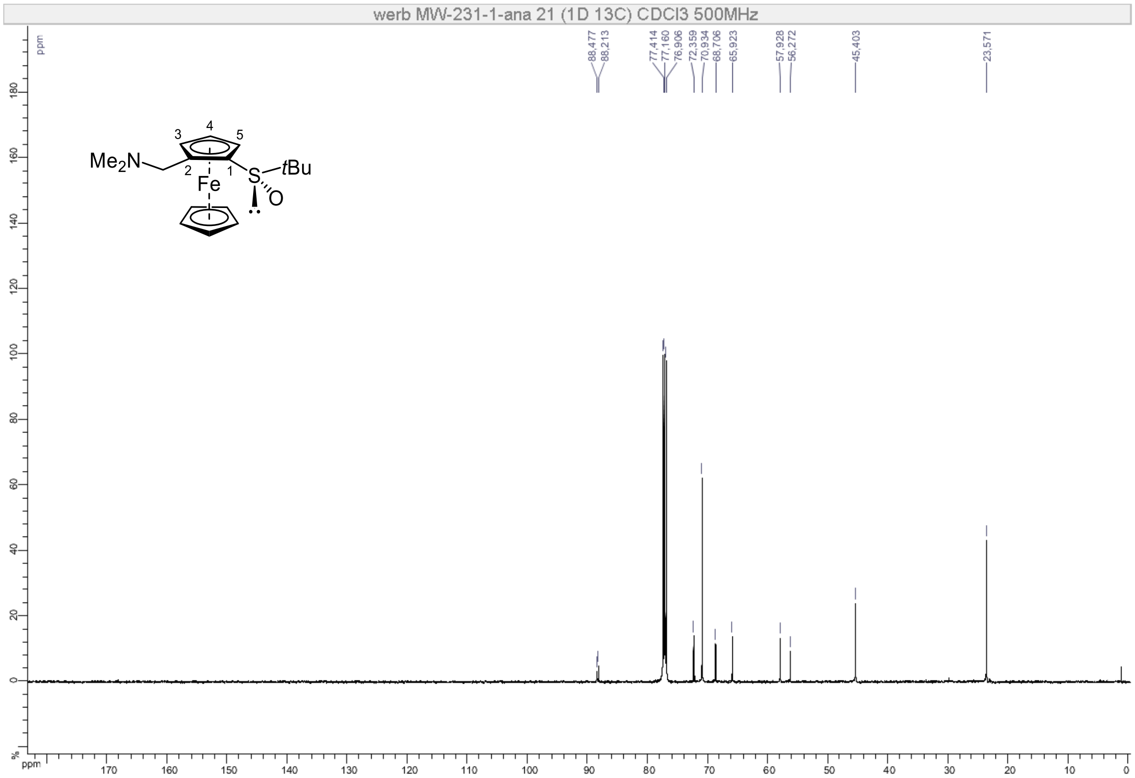

COSY (500 MHz, $CDCl_3$)

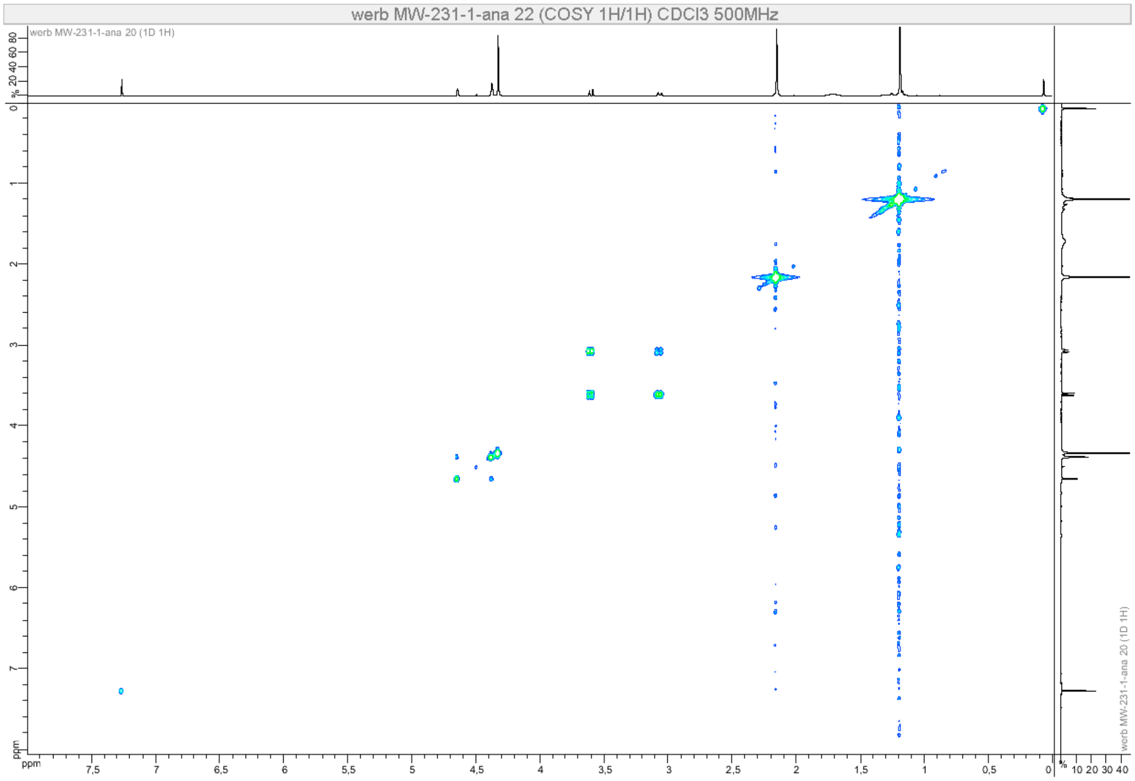

HSQC (500 MHz, $CDCl_3$)

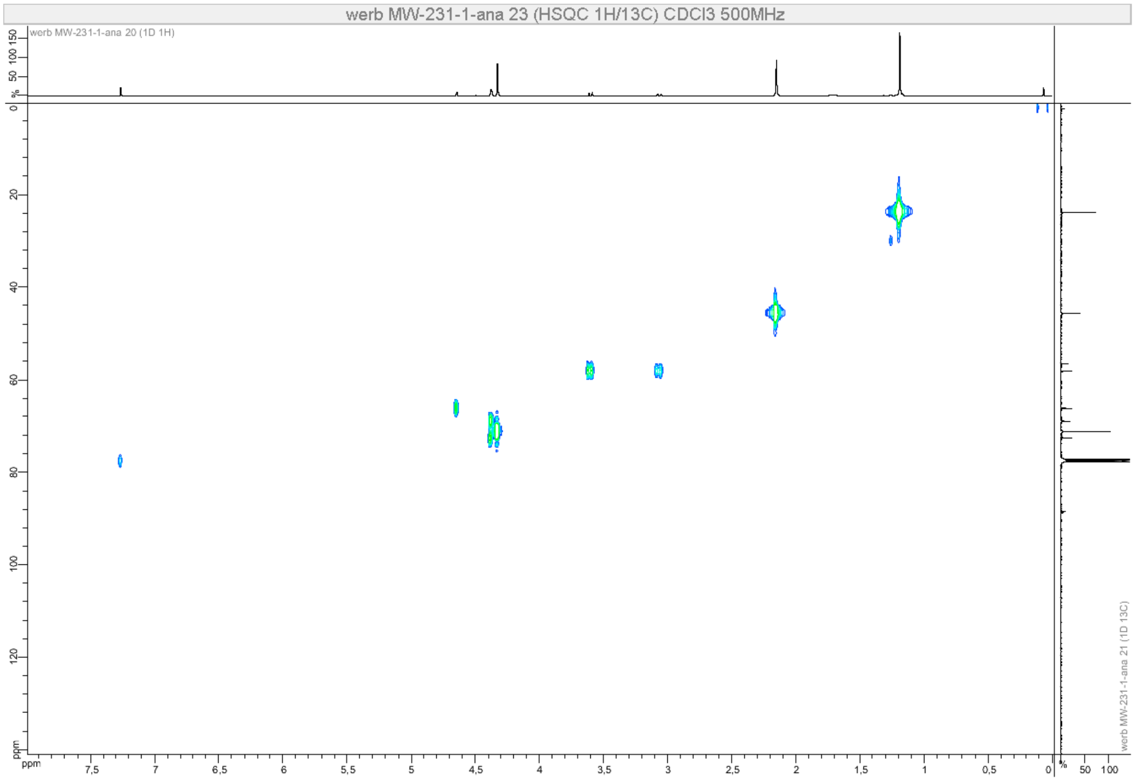

HMBC (500 MHz, $CDCl_3$)

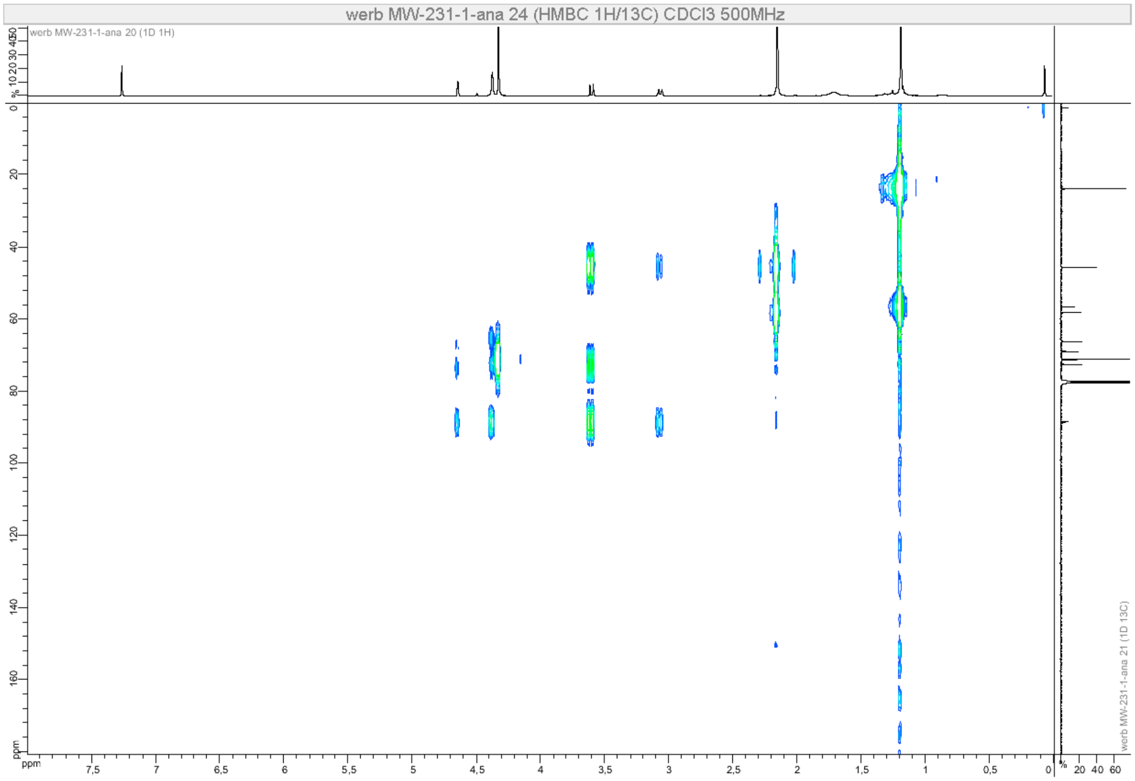

NOESY (500 MHz, $CDCl_3$)

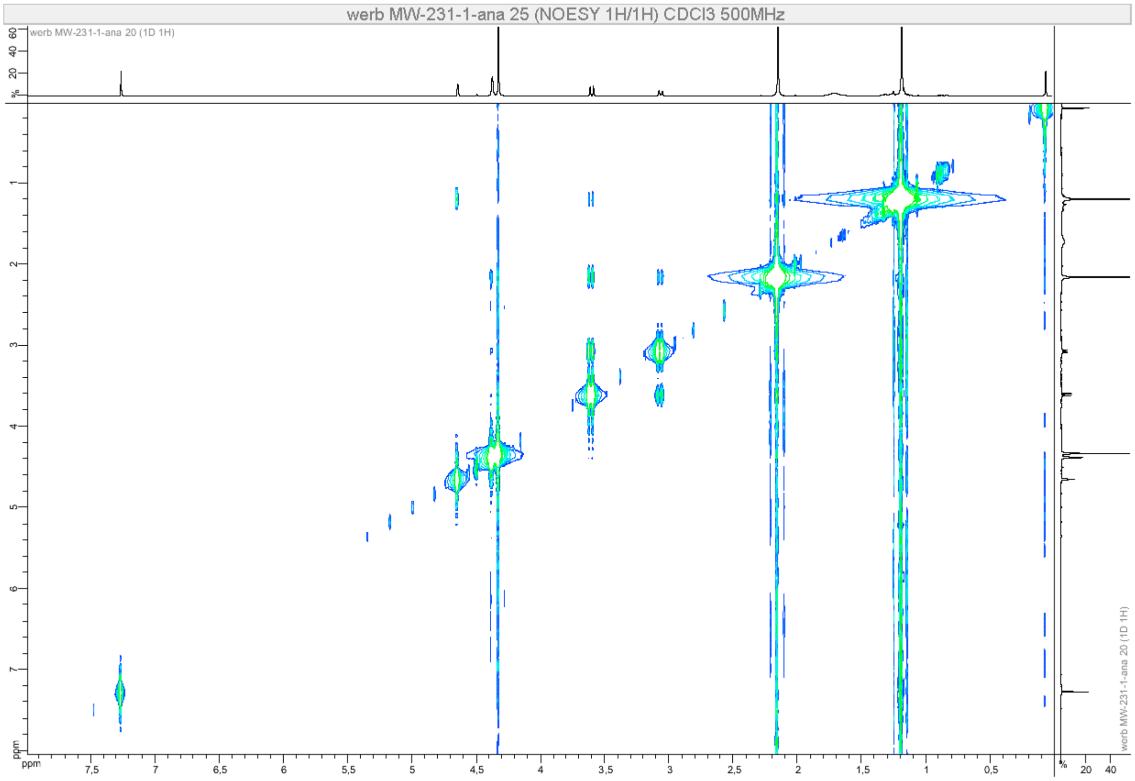

(*S*,*S*P)-*S*-*tert*-Butyl-2-fluoro-5-(trimethylsilyl)ferrocenesulfoxide (*S*,*S*P-2ai)

^{1}H NMR (500 MHz, $CDCl_3$)

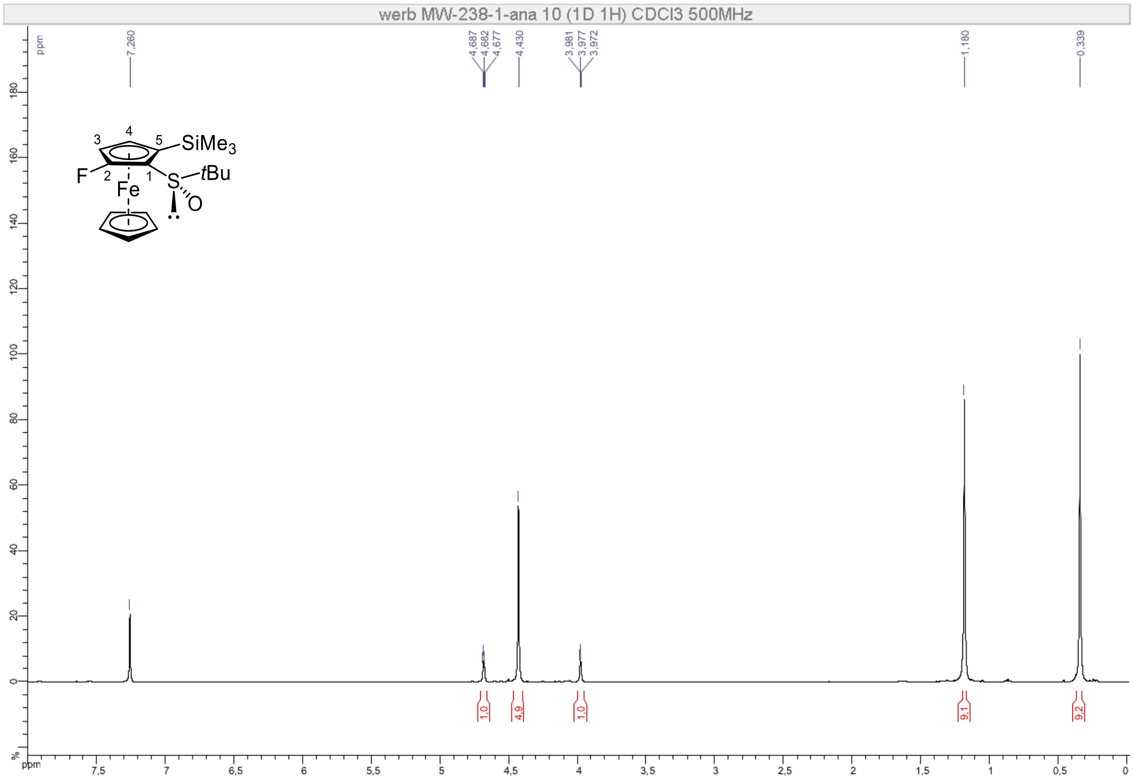

^{13}C NMR (126 MHz, $CDCl_3$)

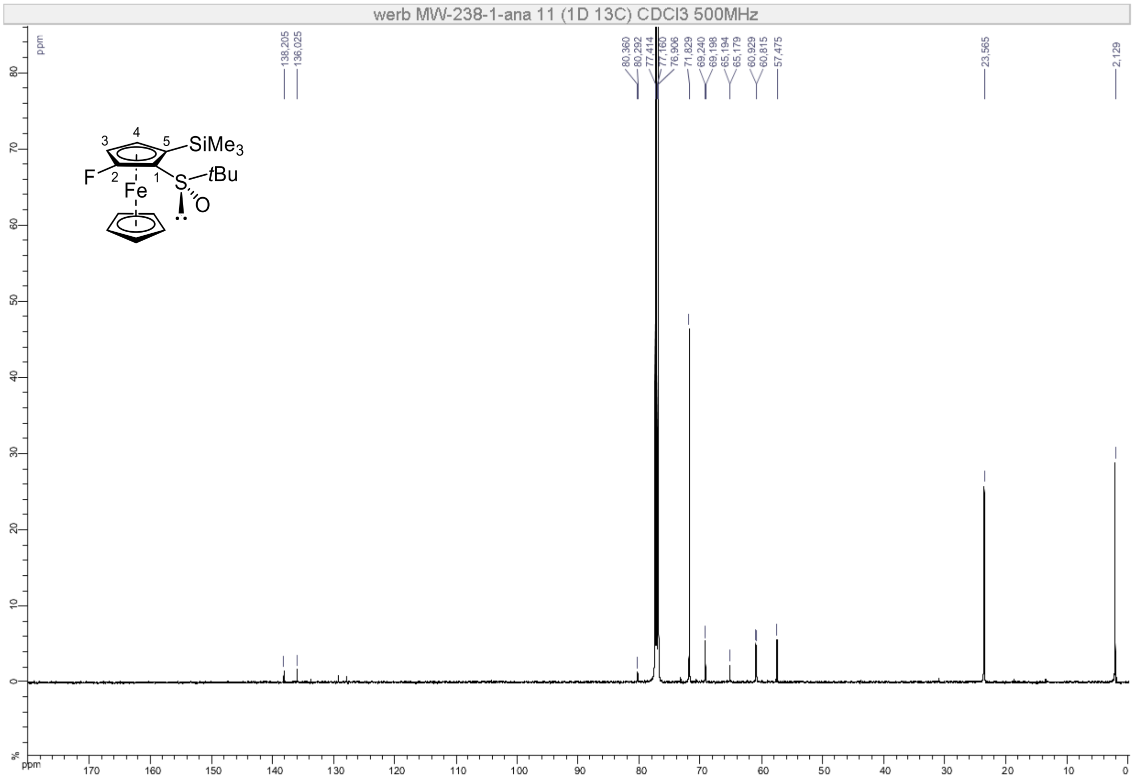

DEPT 135 (126 MHz, $CDCl_3$)

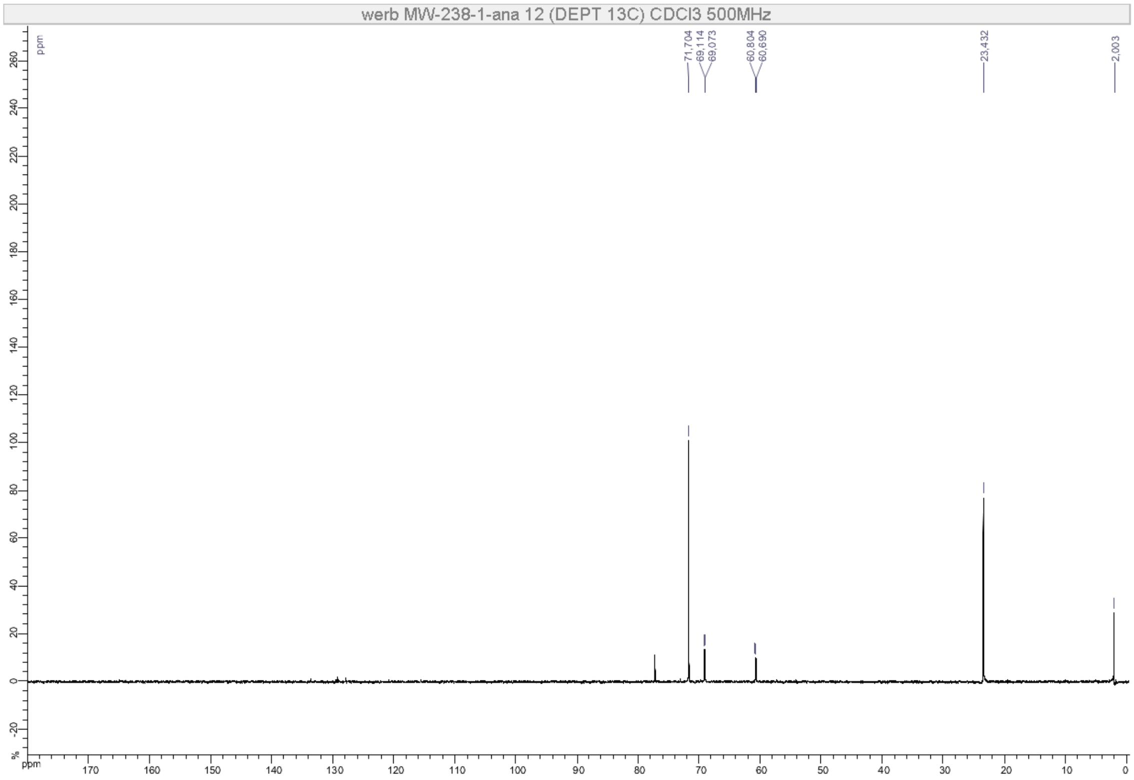

COSY (500 MHz, $CDCl_3$)

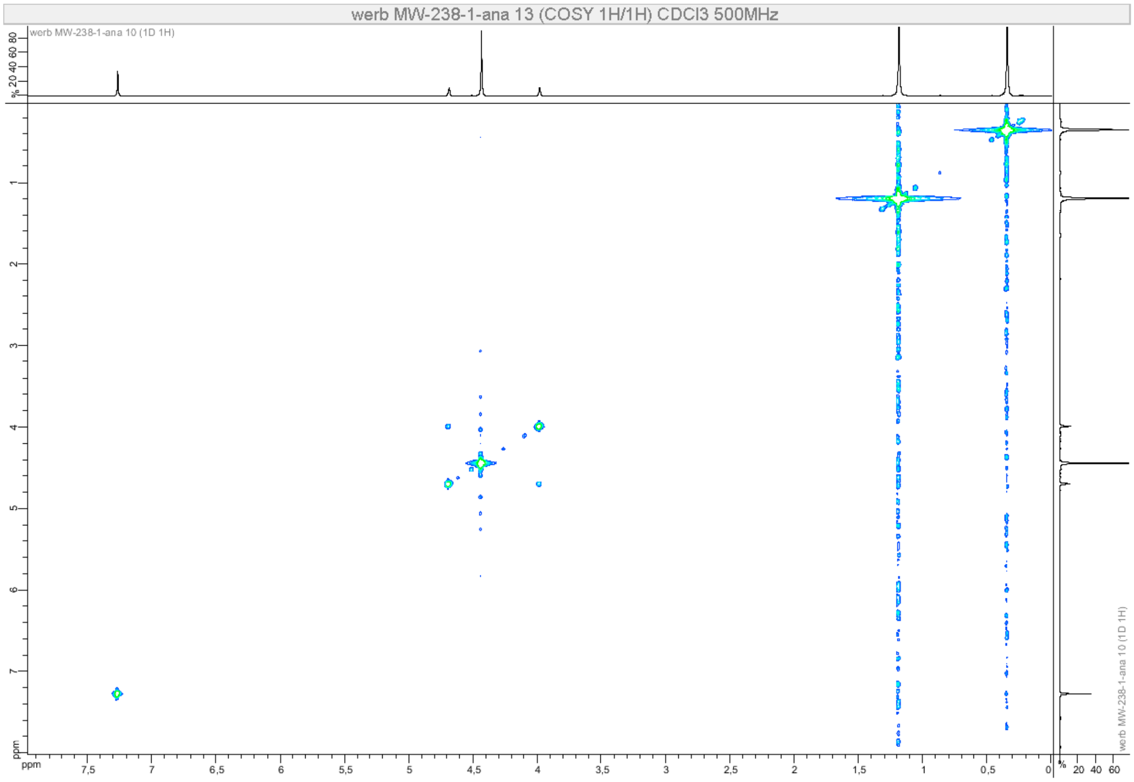

HSQC (500 MHz, $CDCl_3$)

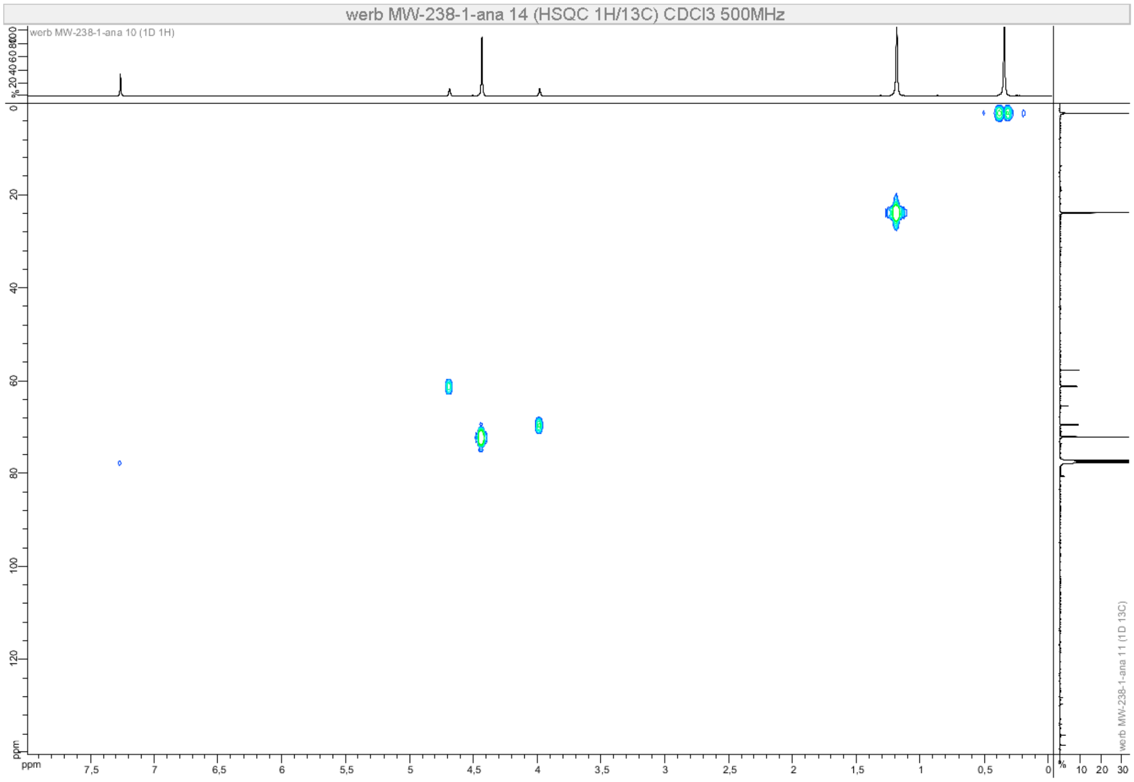

HMBC (500 MHz, $CDCl_3$)

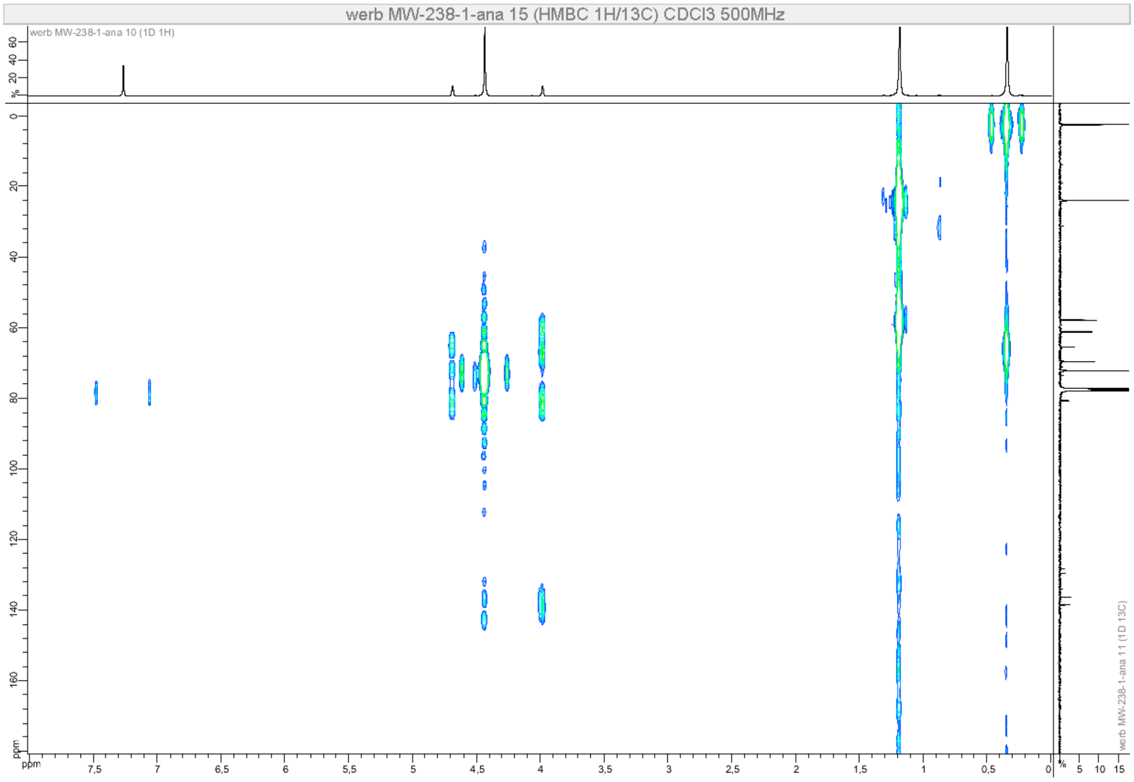

NOESY (500 MHz, $CDCl_3$)

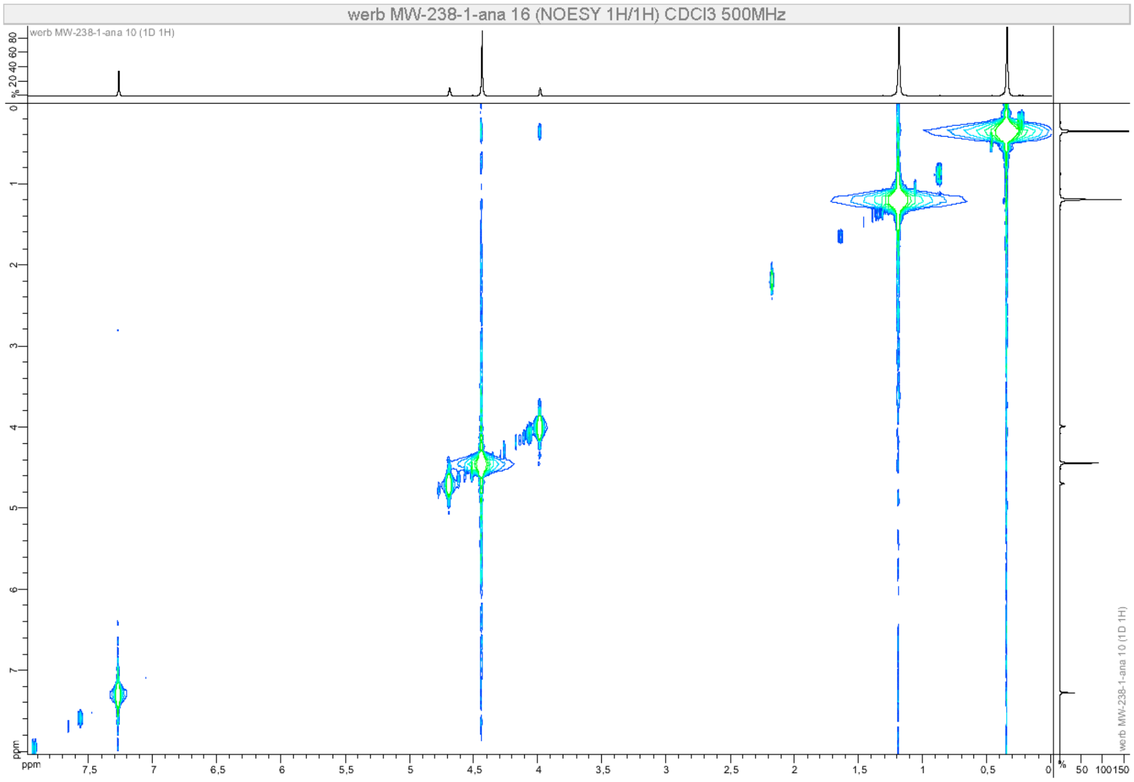

^{19}F NMR (471 MHz, $CDCl_3$)

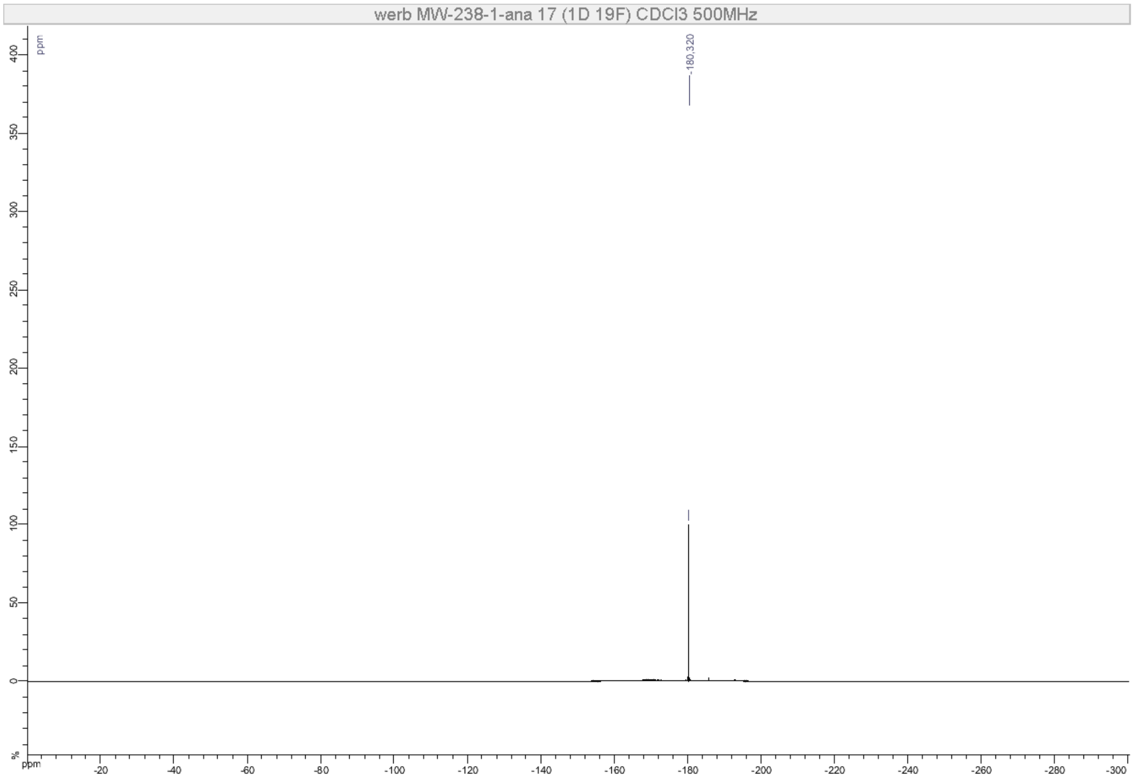

(*S*P)-2-(*tert*-Butylthio)-1-fluoro-3-(trimethylsilyl)ferrocene (*S*P-8ai)

^{1}H NMR (300 MHz, $CDCl_3$)

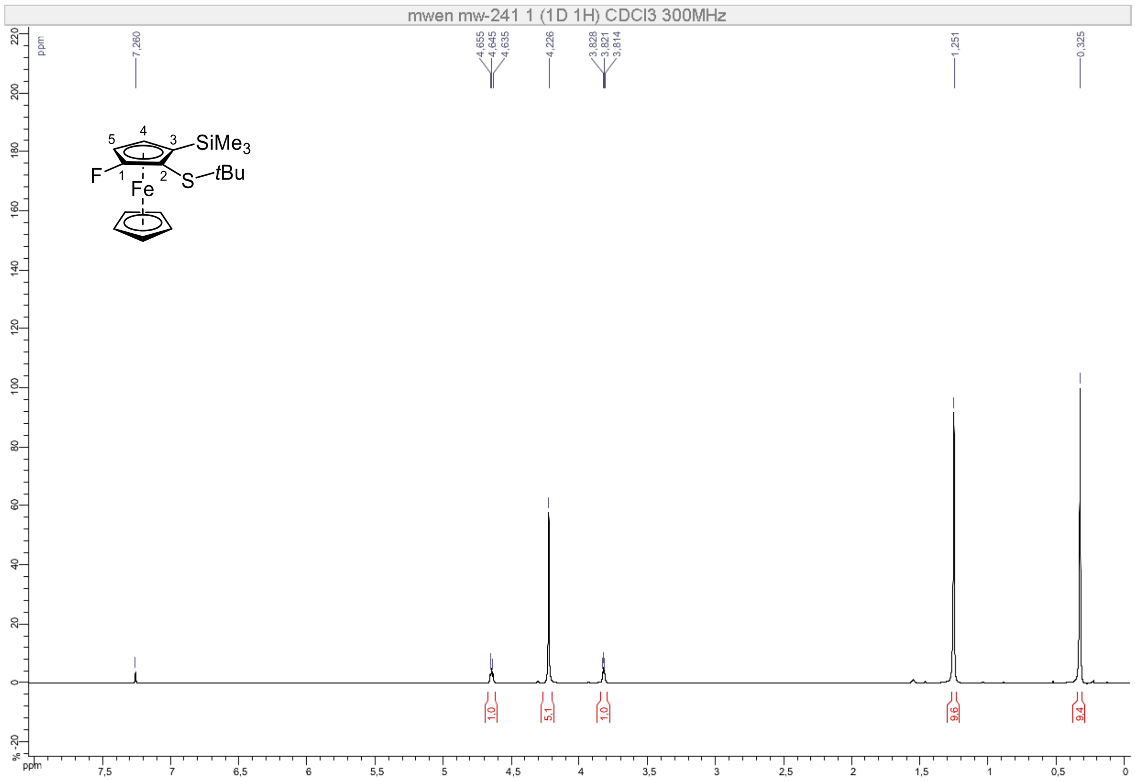

(*S*,*R*P)-*S*-*tert*-Butyl-2-fluoroferrocenesulfoxide (*S*,*R*P-1i)

^{1}H NMR (500 MHz, CDCl$_3$)

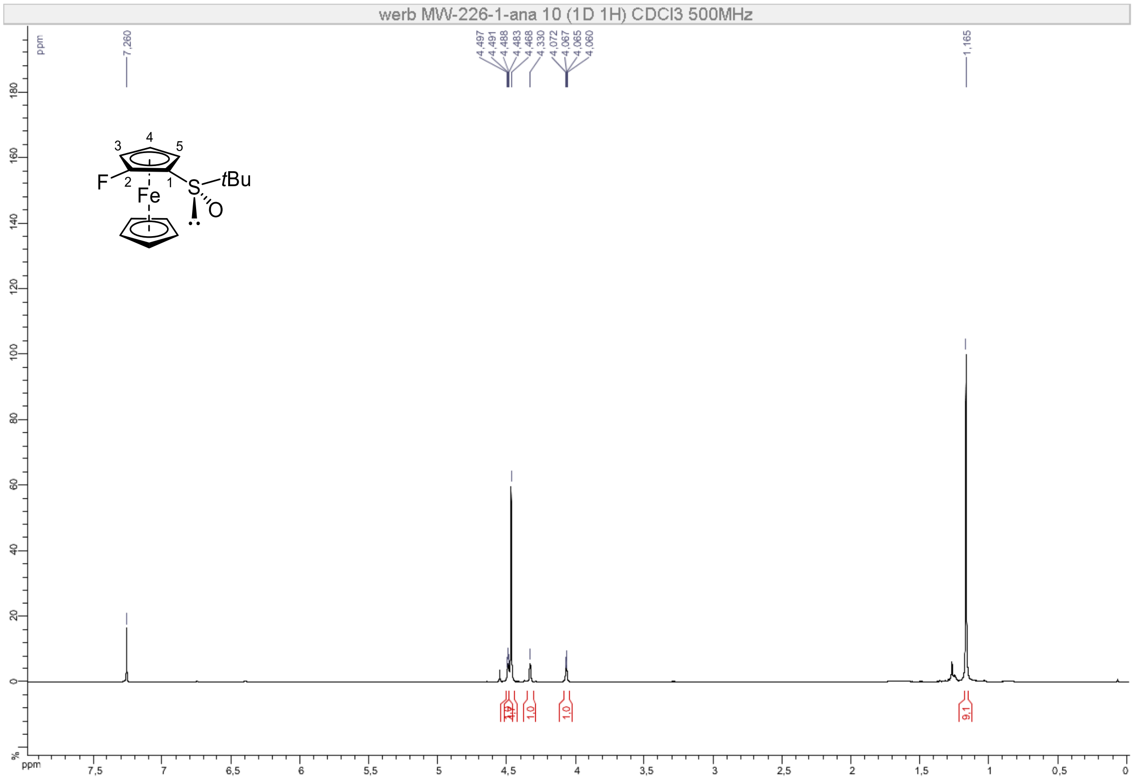

^{13}C NMR (126 MHz, CDCl$_3$)

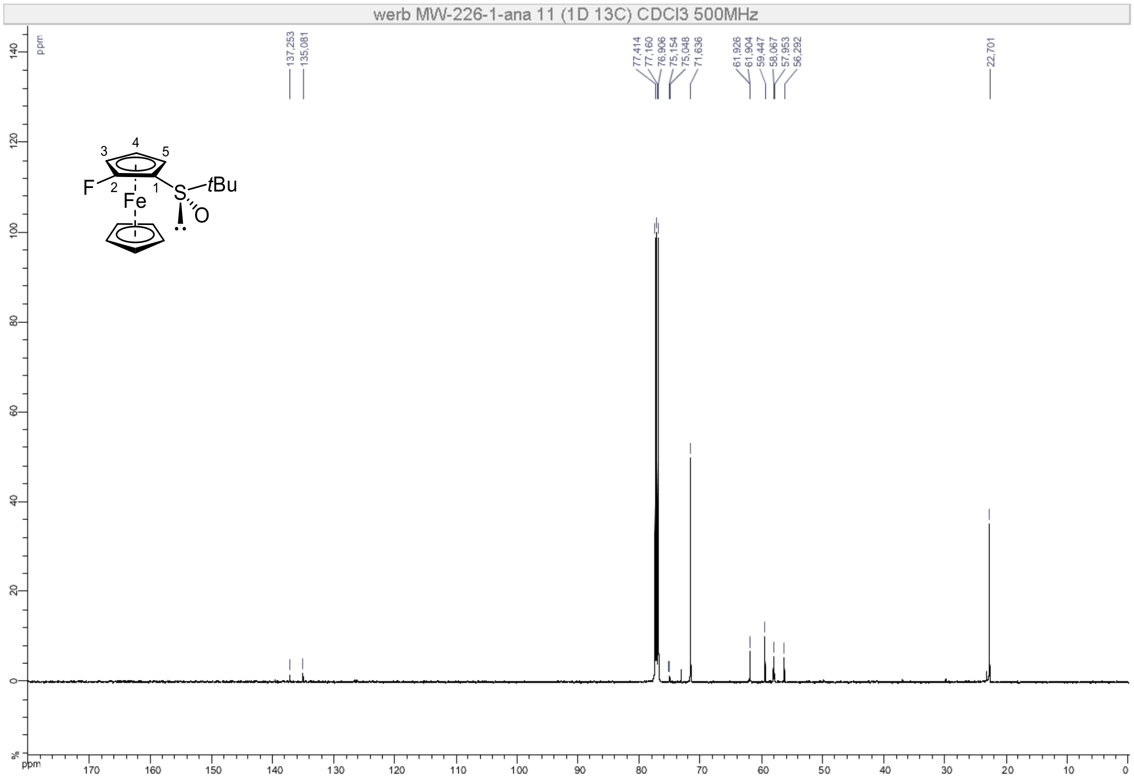

DEPT 135 (126 MHz, $CDCl_3$)

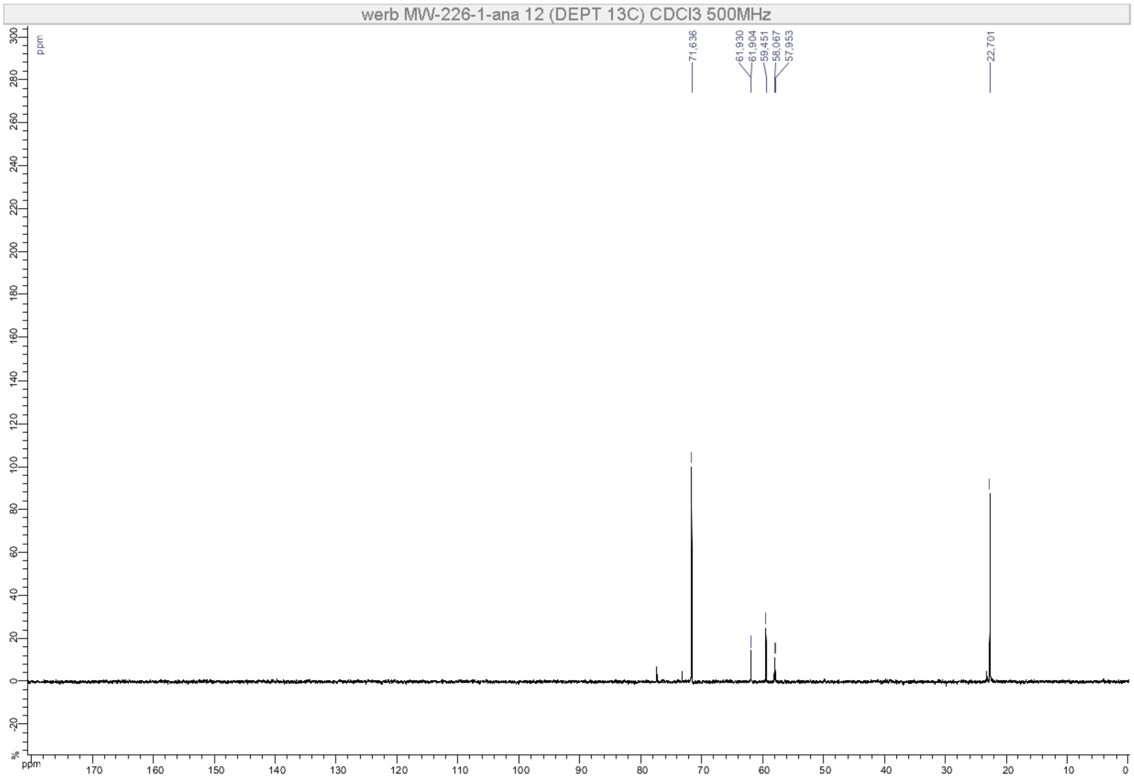

COSY (500 MHz, $CDCl_3$)

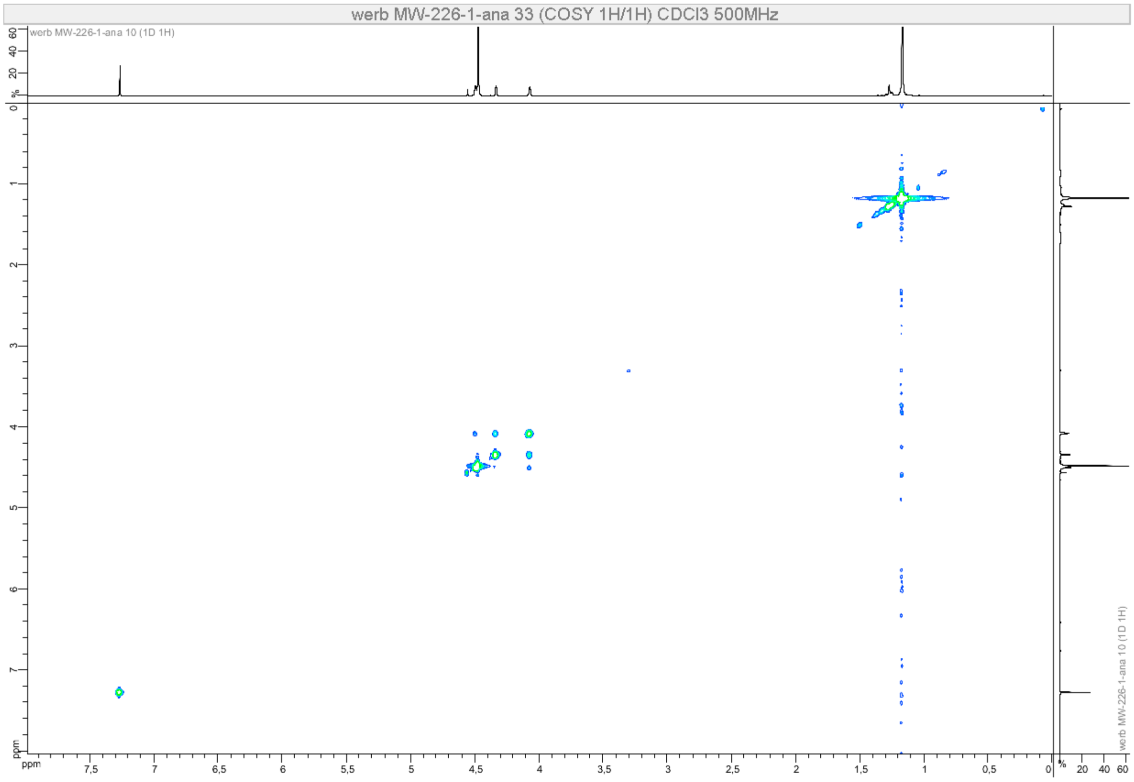

HSQC (500 MHz, $CDCl_3$)

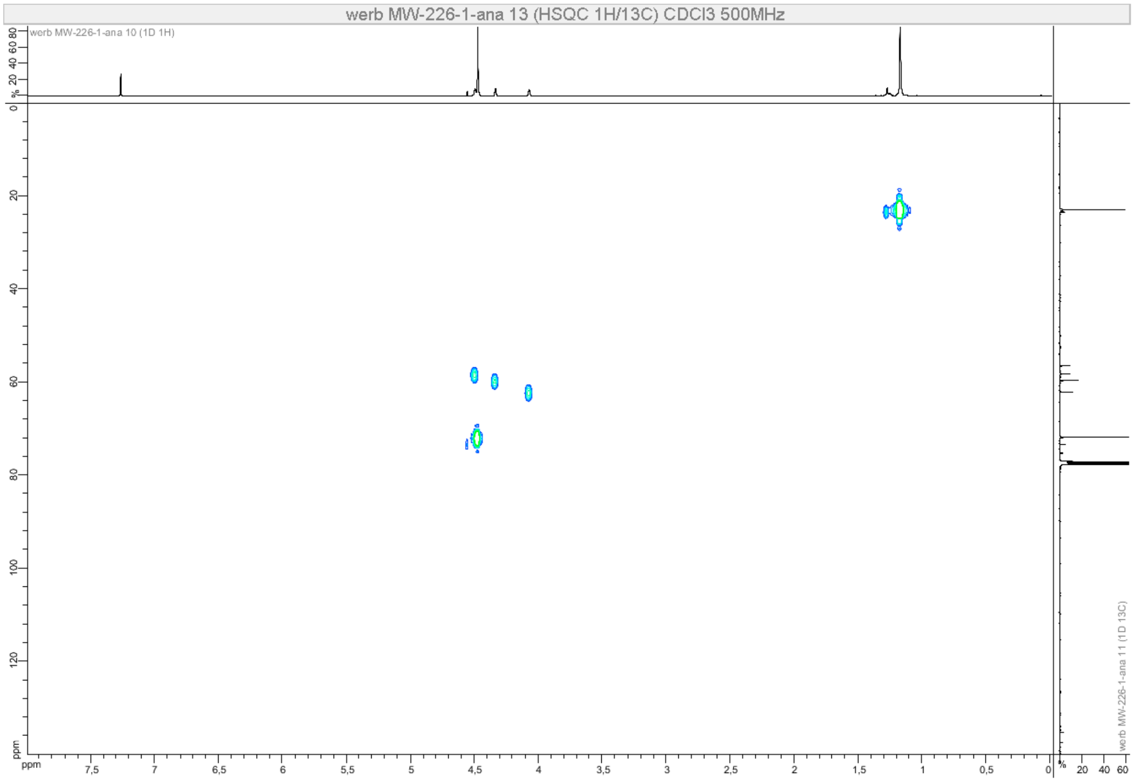

HMBC (500 MHz, $CDCl_3$)

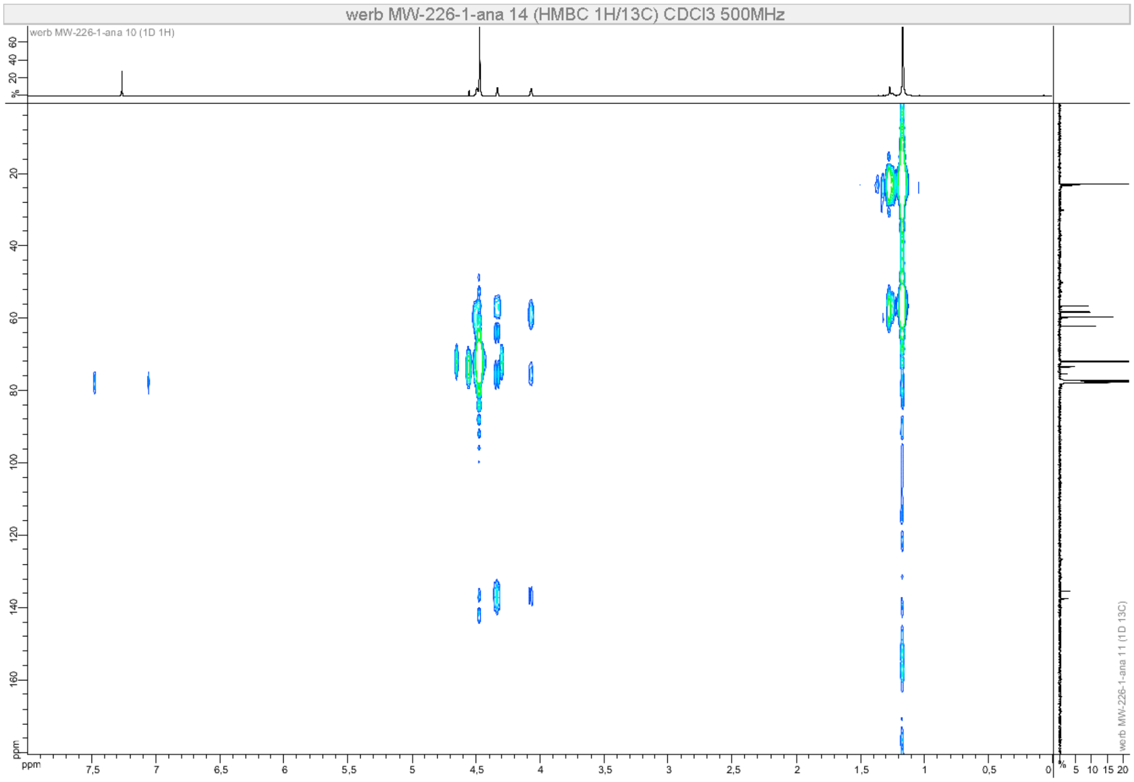

NOESY (500 MHz, $CDCl_3$)

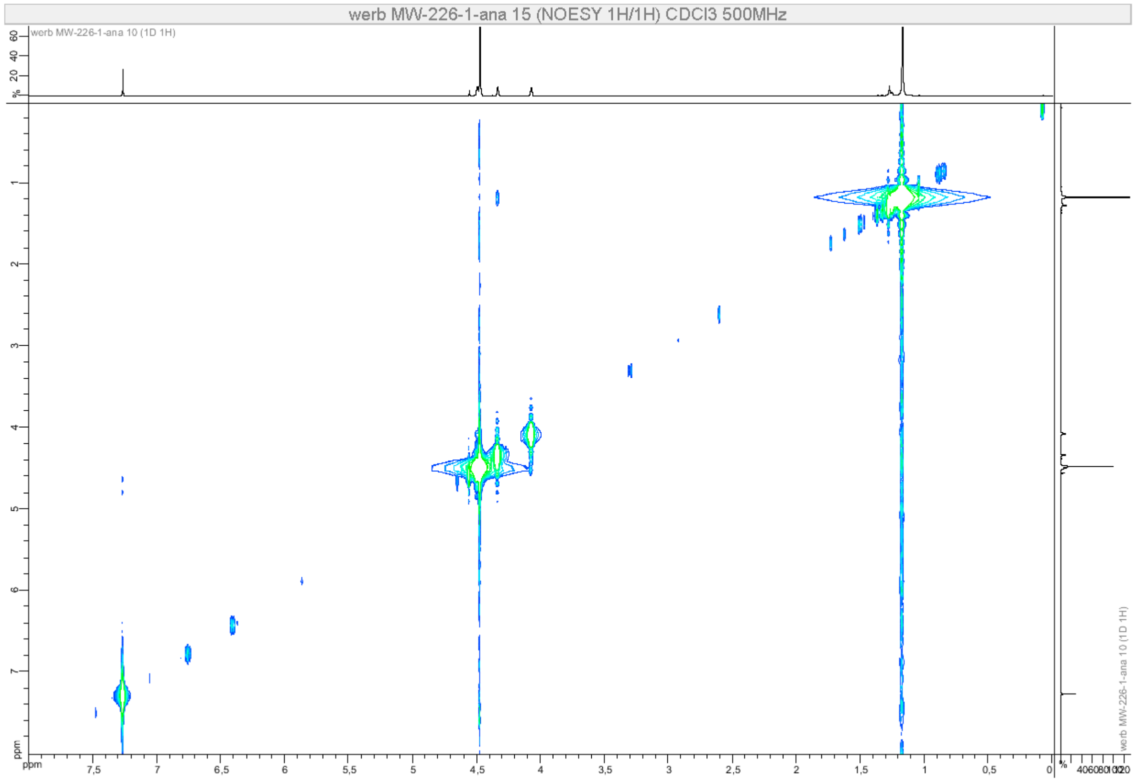

^{19}F NMR (471 MHz, $CDCl_3$)

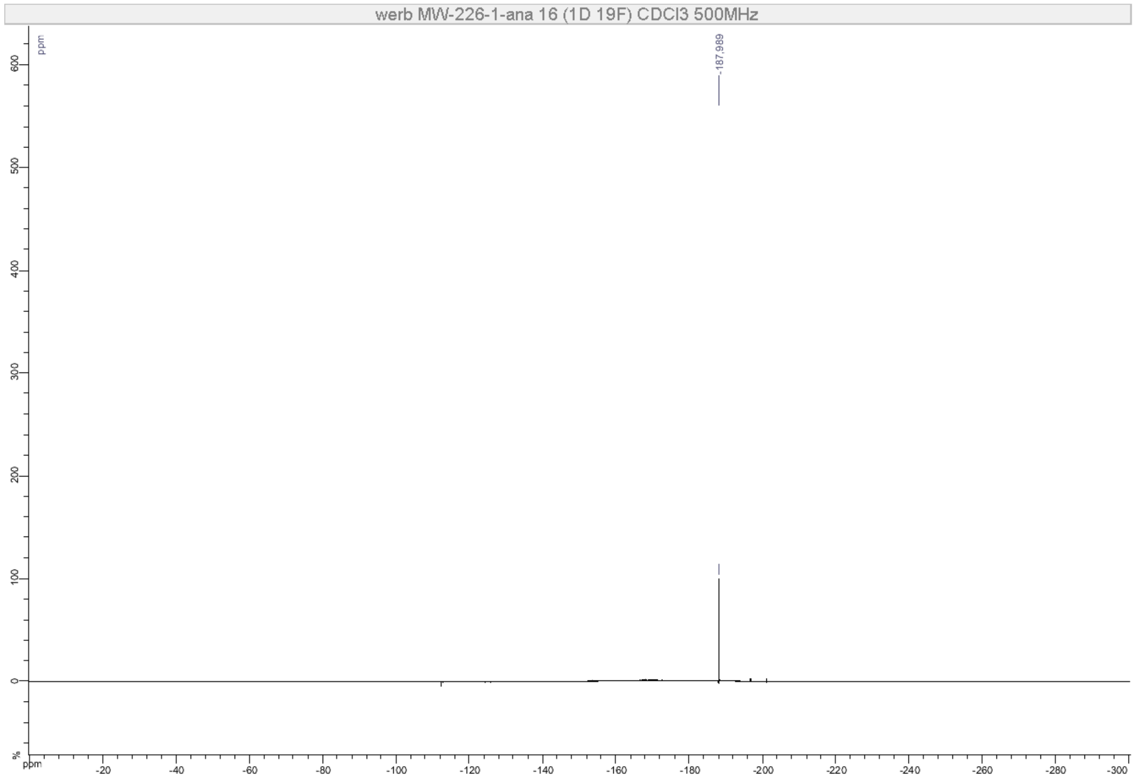

(R_P)-1-(*tert*-Butylthio)-2-fluoroferrocene (R_P-8i)

^{1}H NMR (500 MHz, $CDCl_3$)

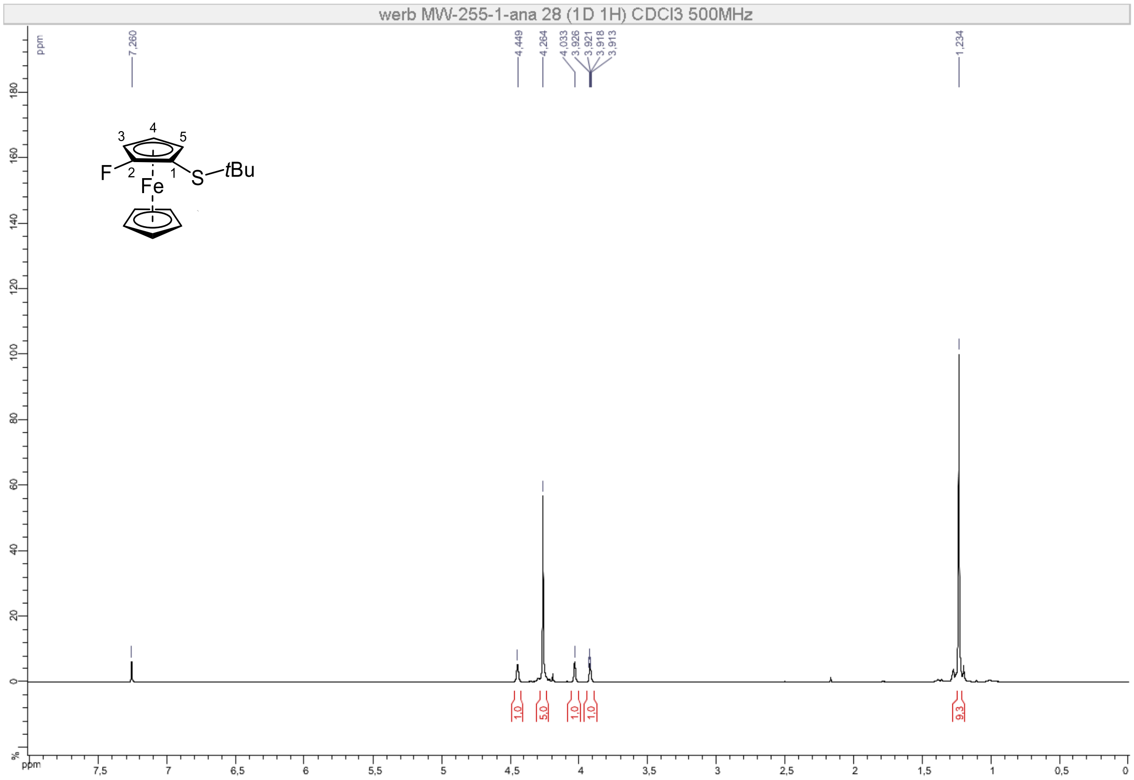

^{13}C NMR (126 MHz, $CDCl_3$)

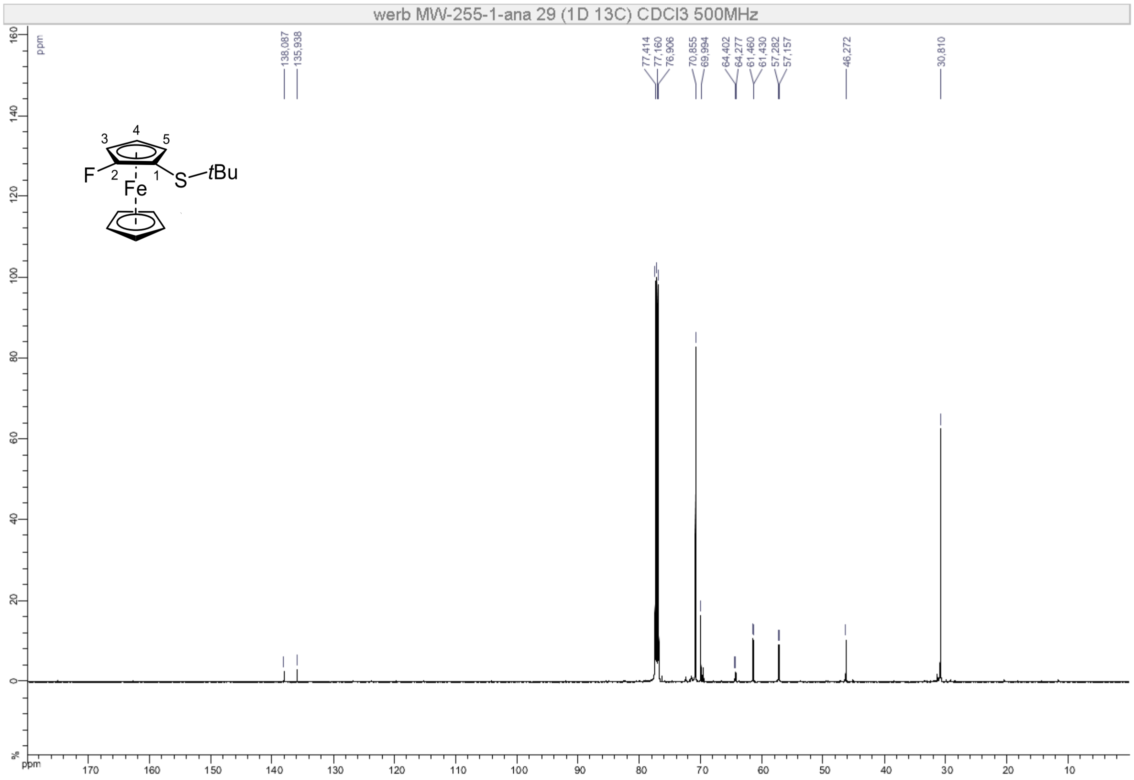

DEPT 135 (126 MHz, $CDCl_3$)

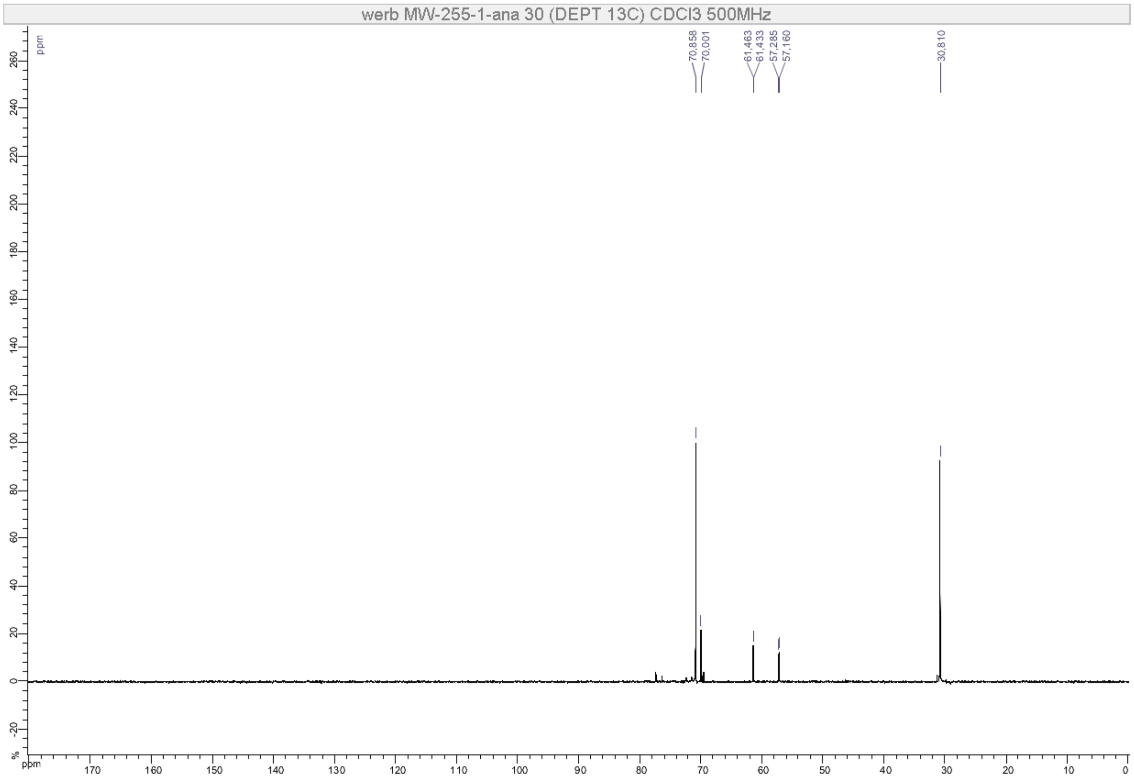

COSY (500 MHz, $CDCl_3$)

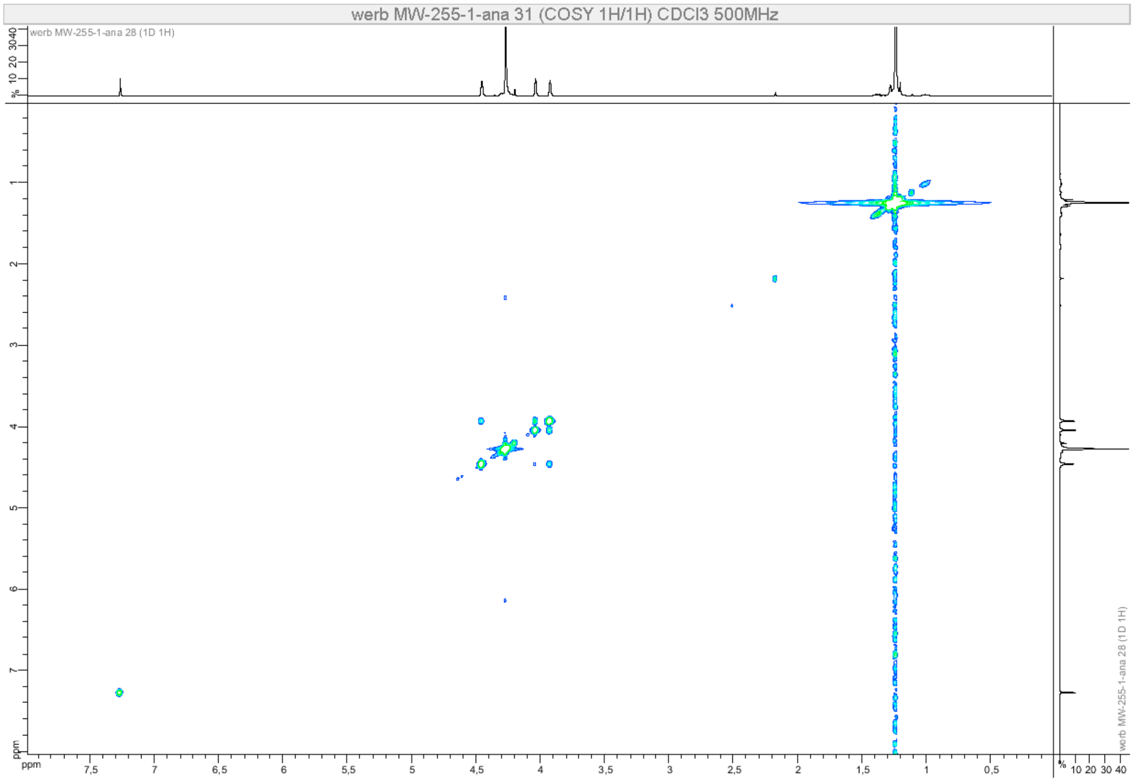

HSQC (500 MHz, $CDCl_3$)

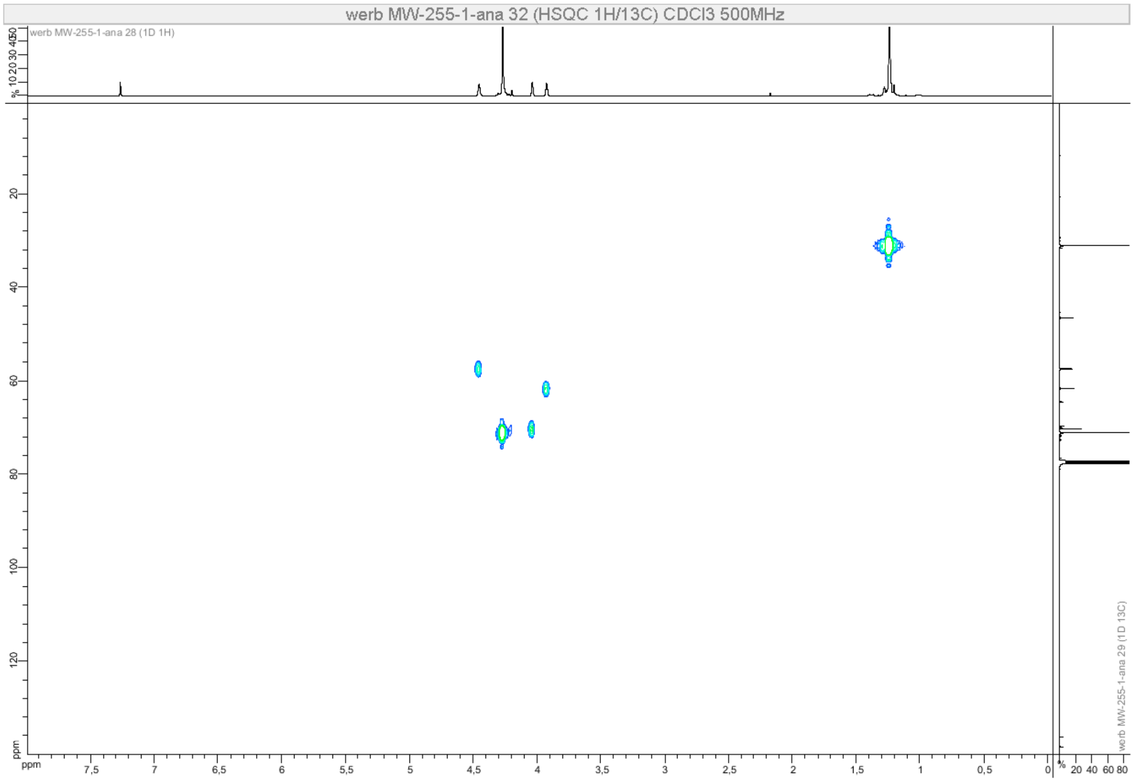

HMBC (500 MHz, $CDCl_3$)

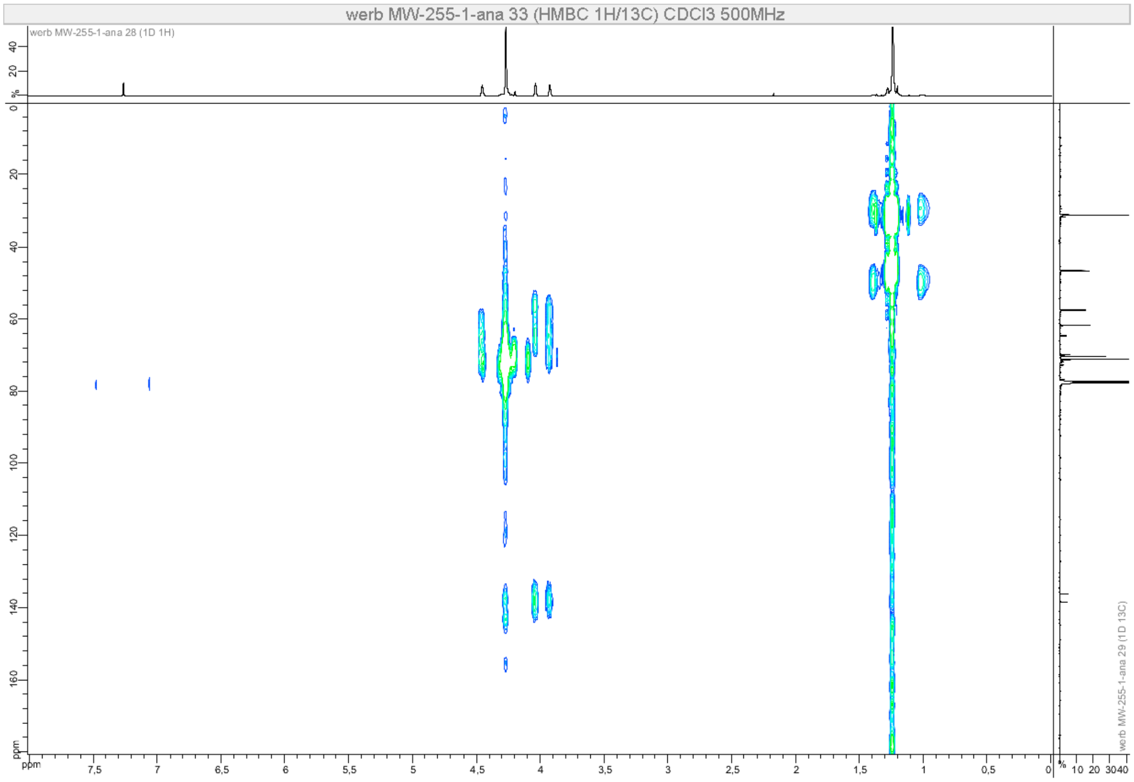

NOESY (500 MHz, $CDCl_3$)

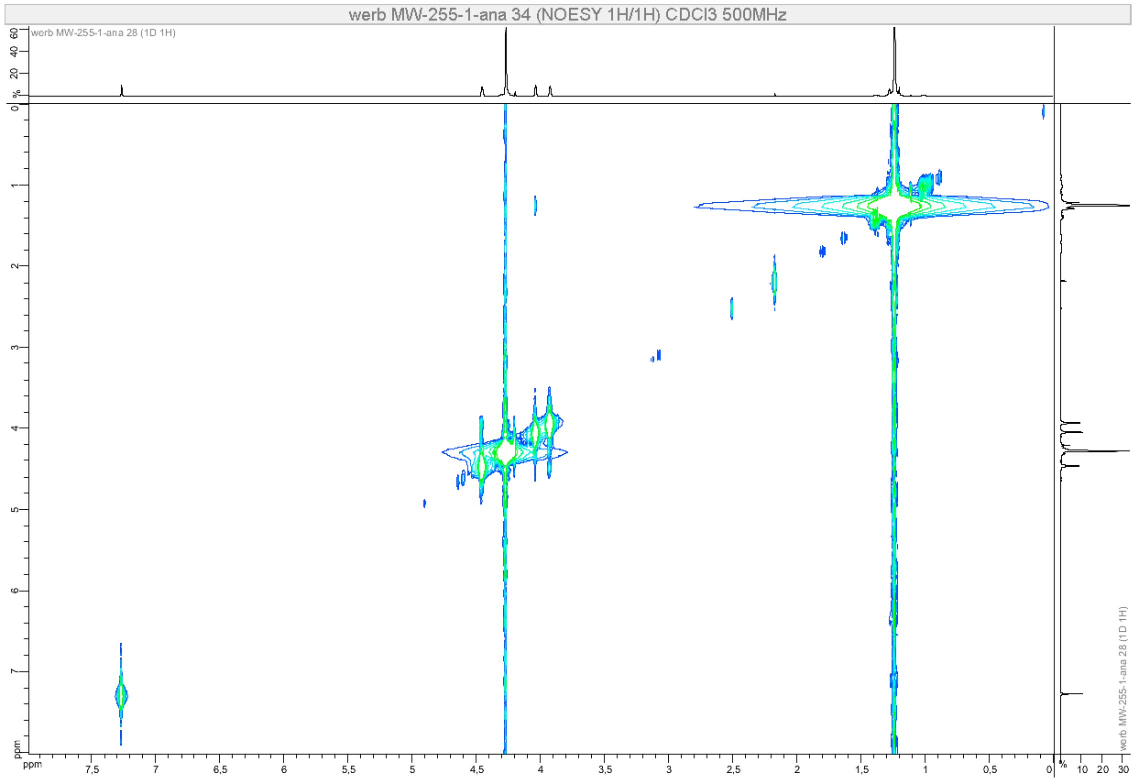

^{19}F NMR (471 MHz, $CDCl_3$)

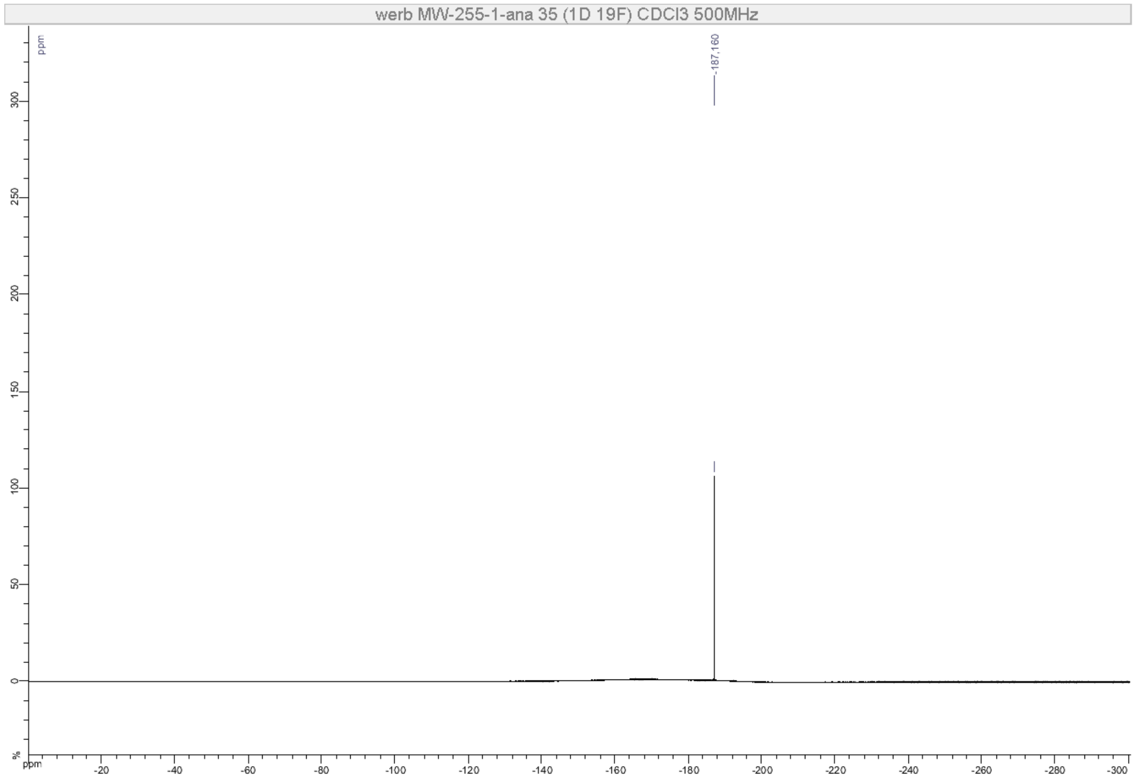

(S,R_P)-S-*tert*-Butyl-2-fluoro-5-iodoferrocenesulfoxide (S,R_P-2bi)

^{1}H NMR (500 MHz, $CDCl_3$)

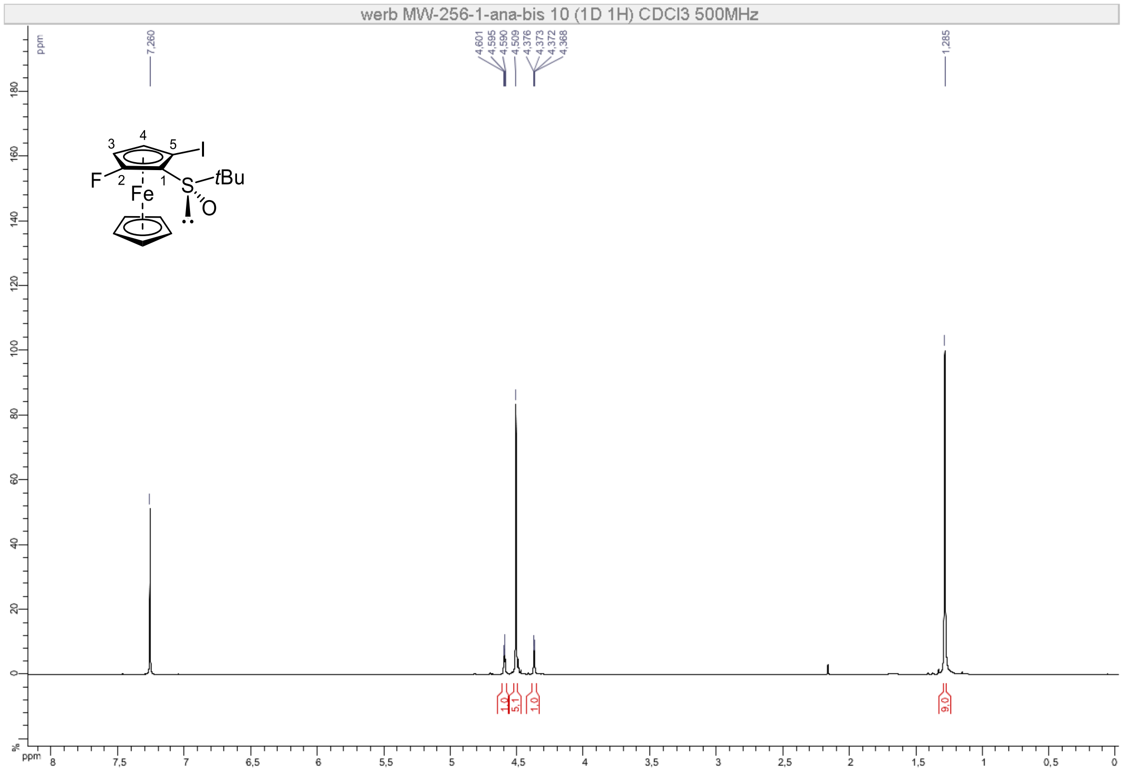

^{13}C NMR (126 MHz, $CDCl_3$)

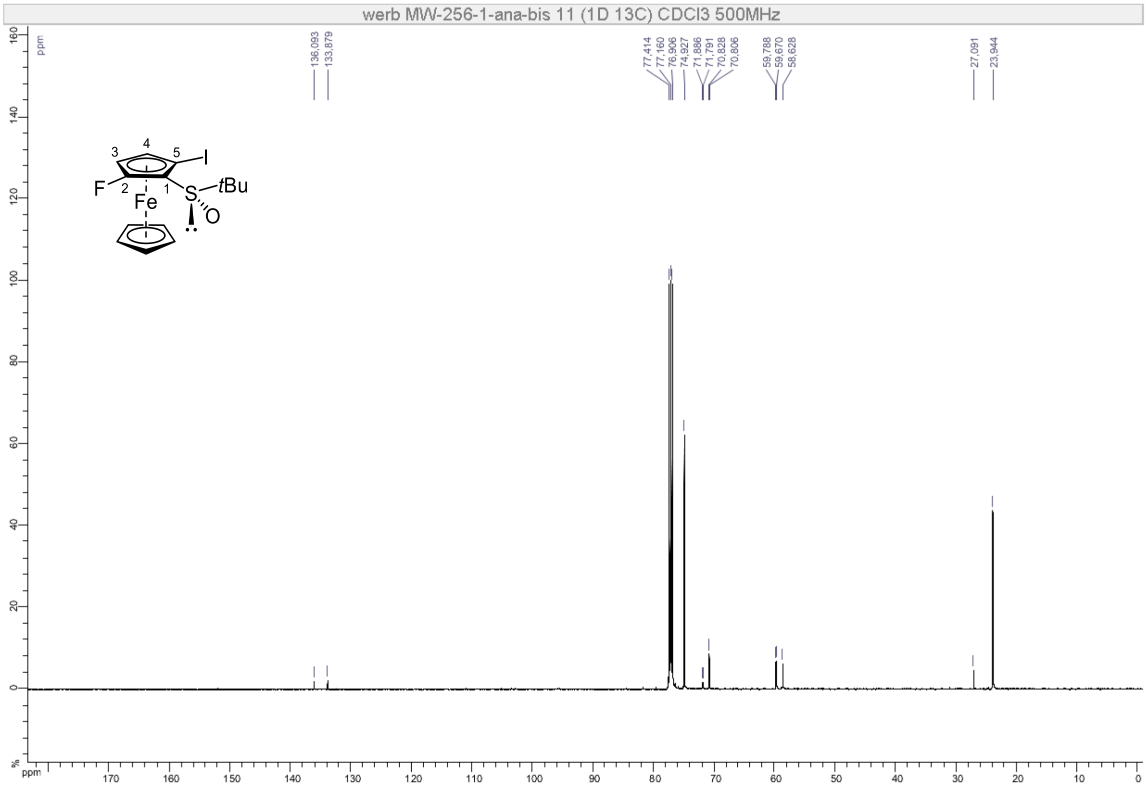

DEPT 135 (126 MHz, $CDCl_3$)

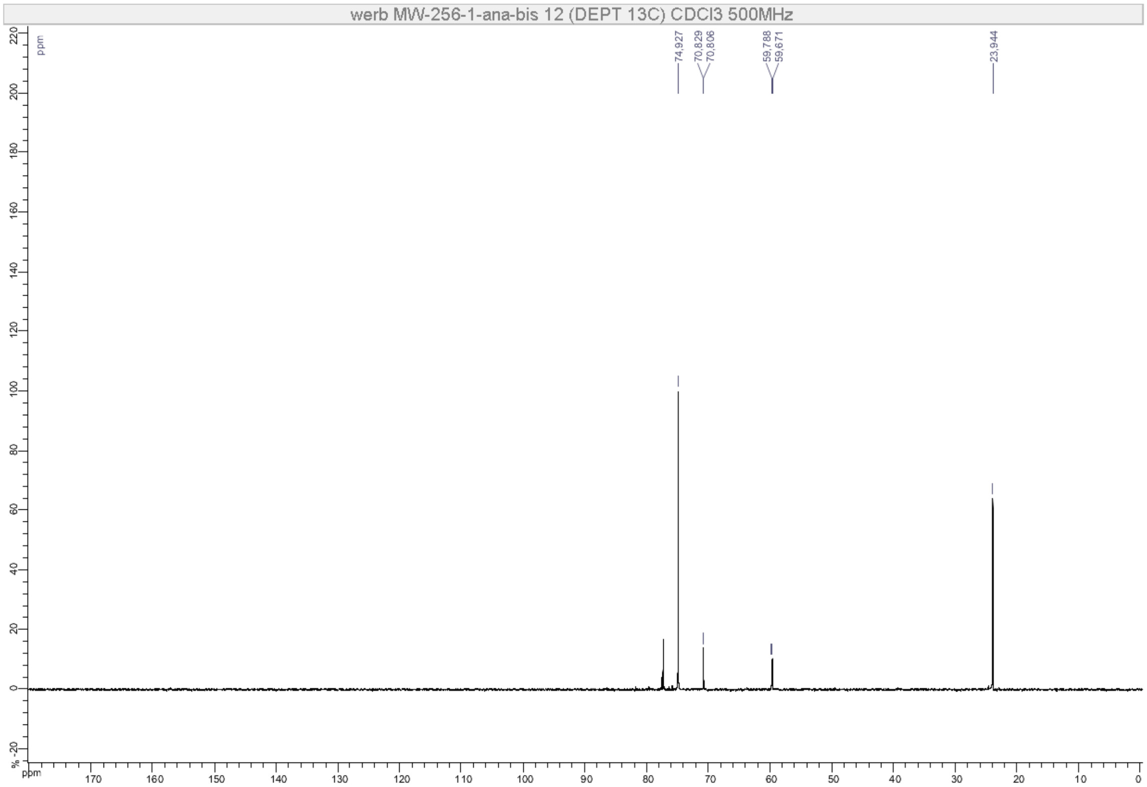

COSY (500 MHz, $CDCl_3$)

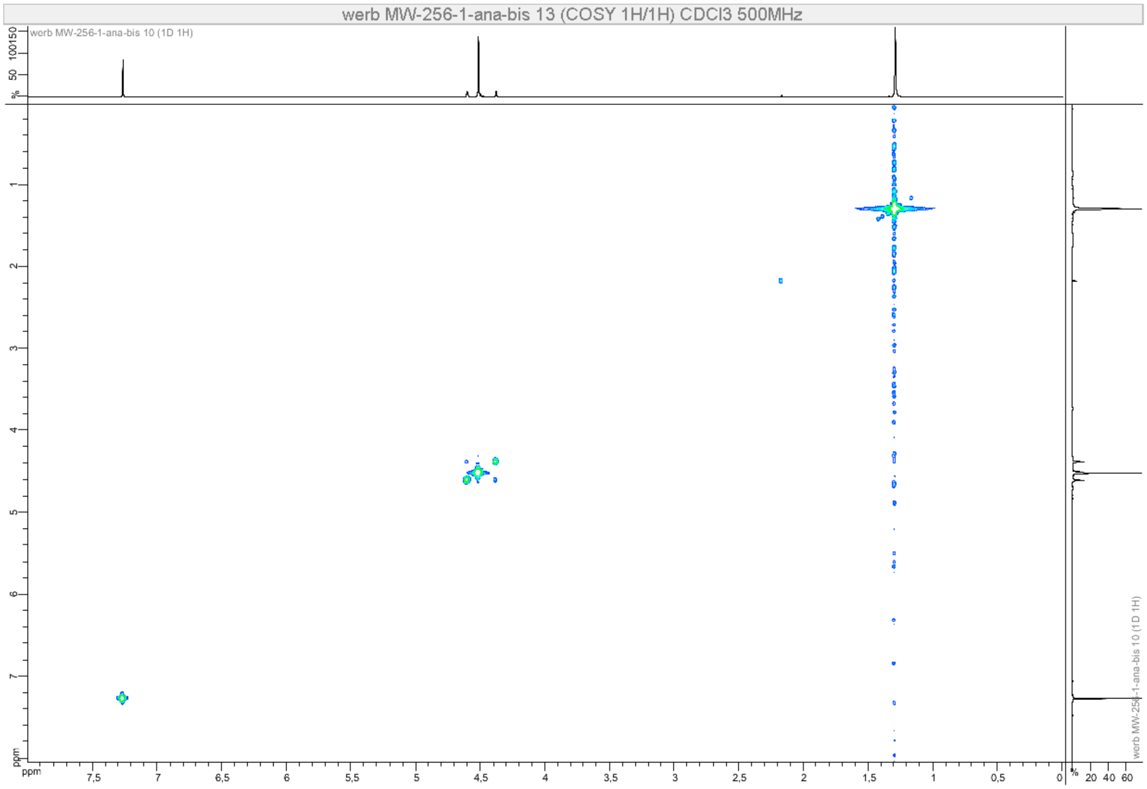

HSQC (500 MHz, $CDCl_3$)

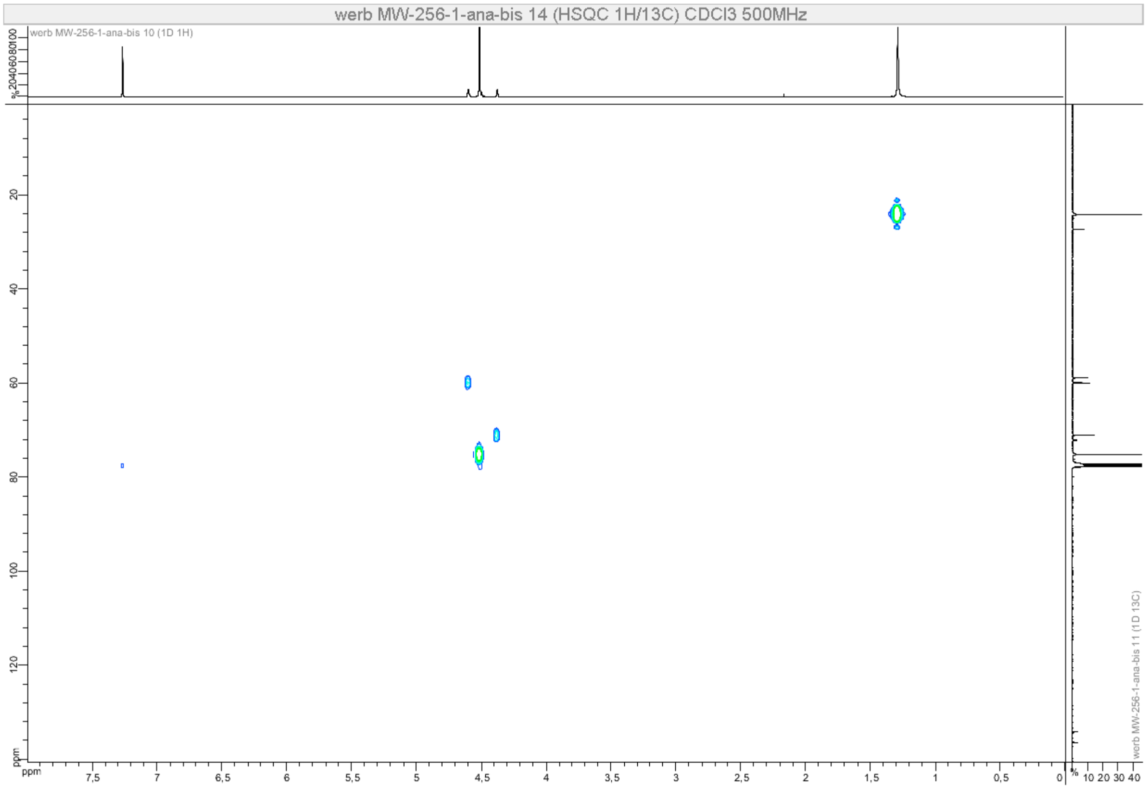

HMBC (500 MHz, $CDCl_3$)

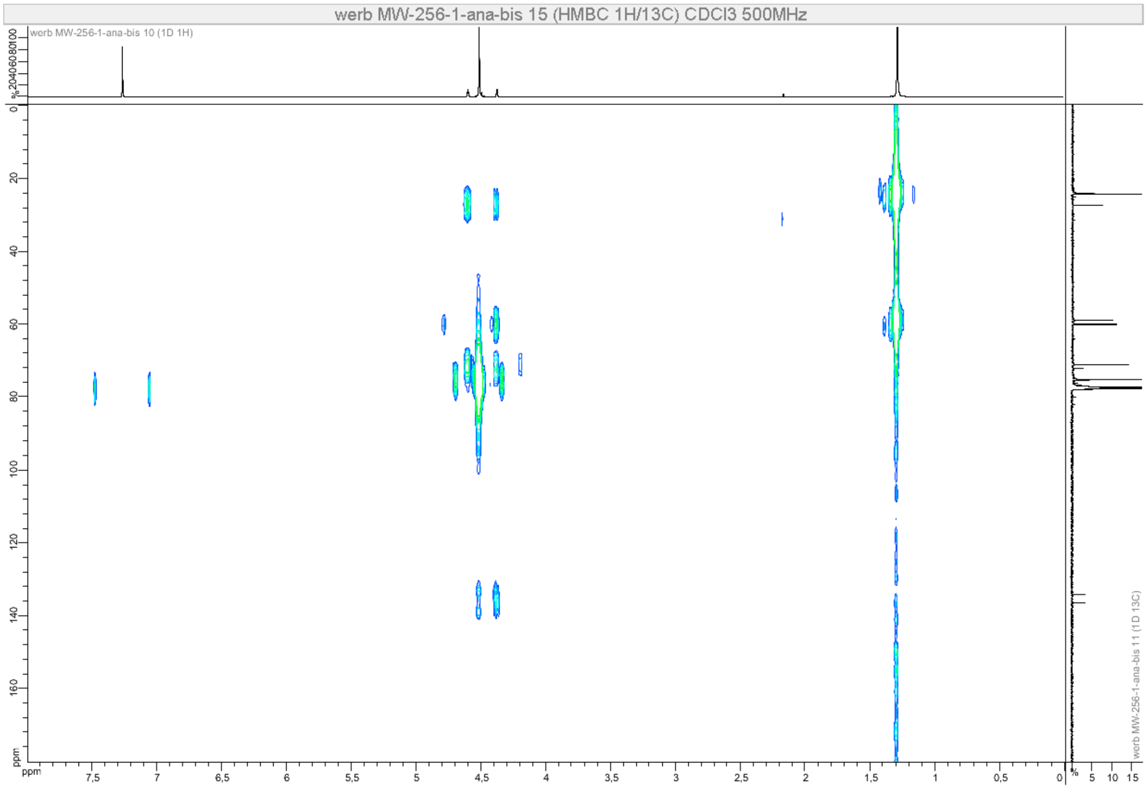

^{19}F NMR (471 MHz, $CDCl_3$)

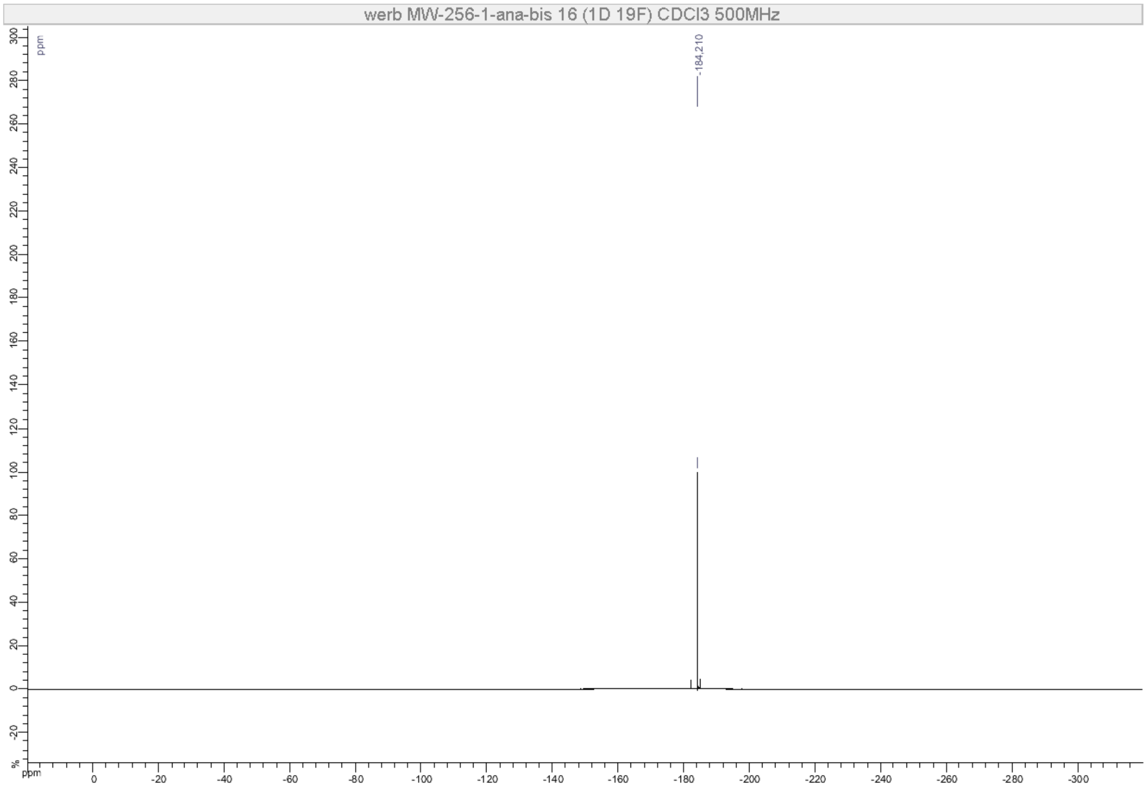

(S,S_P)-*S-tert*-Butyl-2-(diphenylphosphino)-5-fluoroferrocenesulfoxide (S,S_P-2ei)

^{1}H NMR (500 MHz, $CDCl_3$)

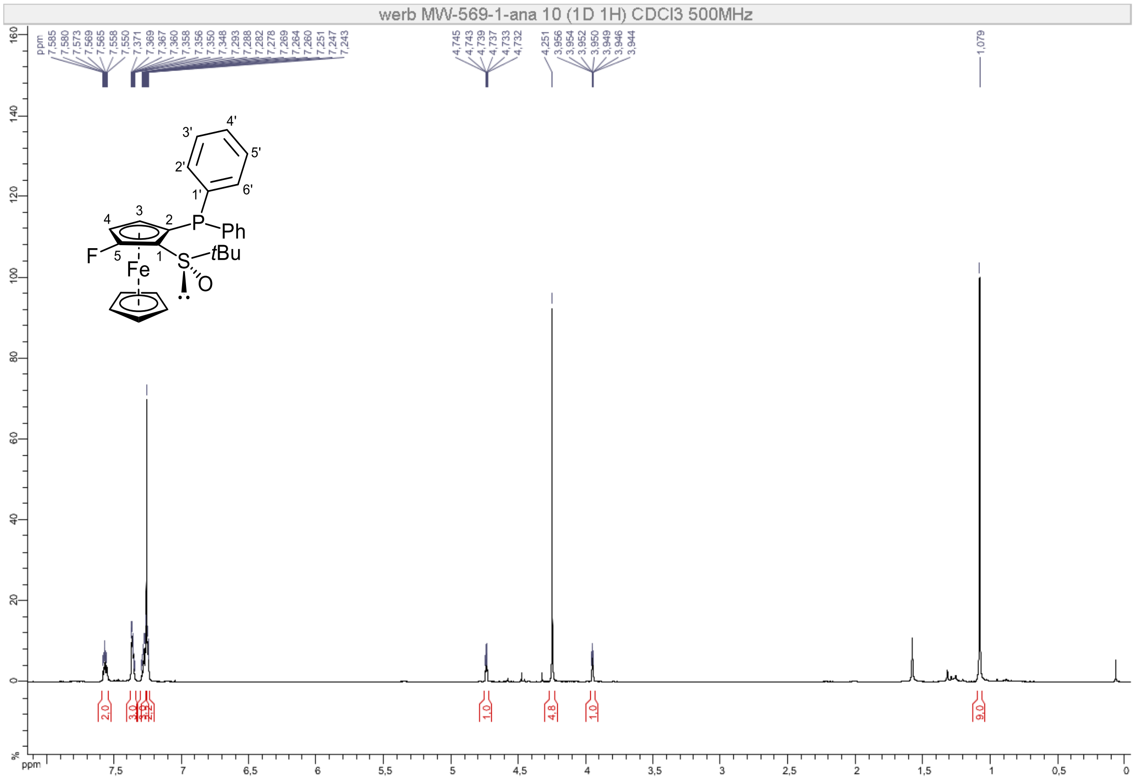

^{13}C NMR (126 MHz, $CDCl_3$)

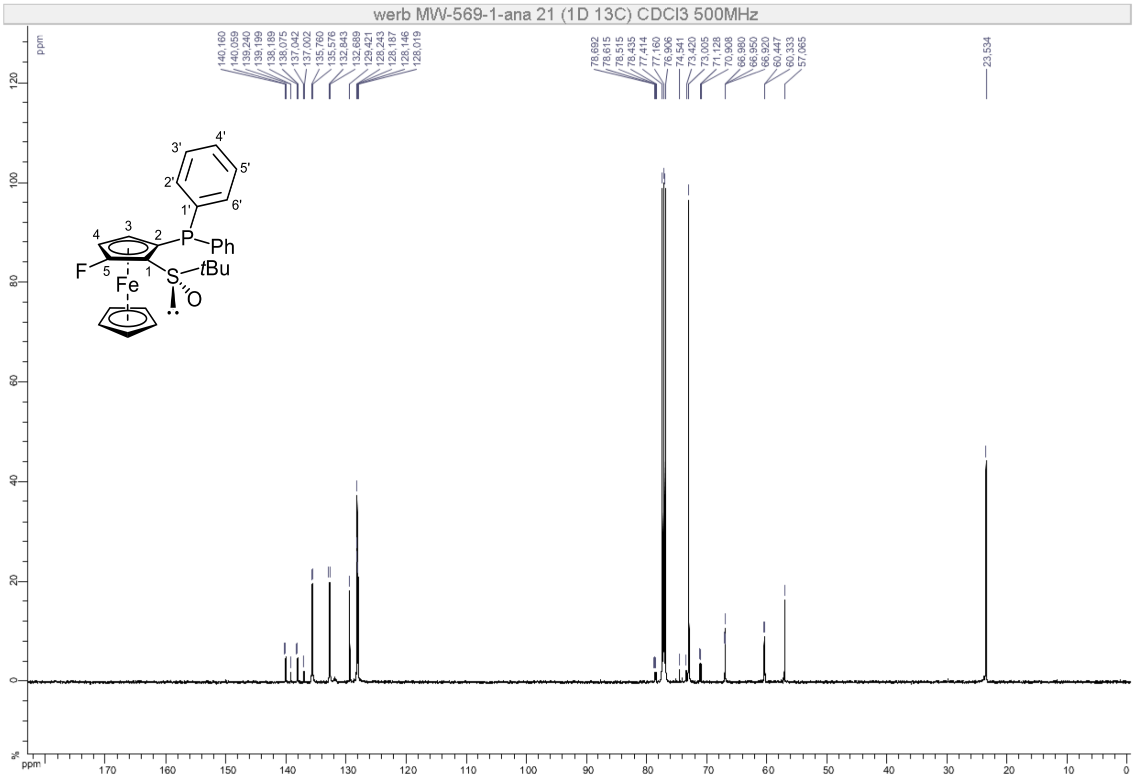

COSY (500 MHz, $CDCl_3$)

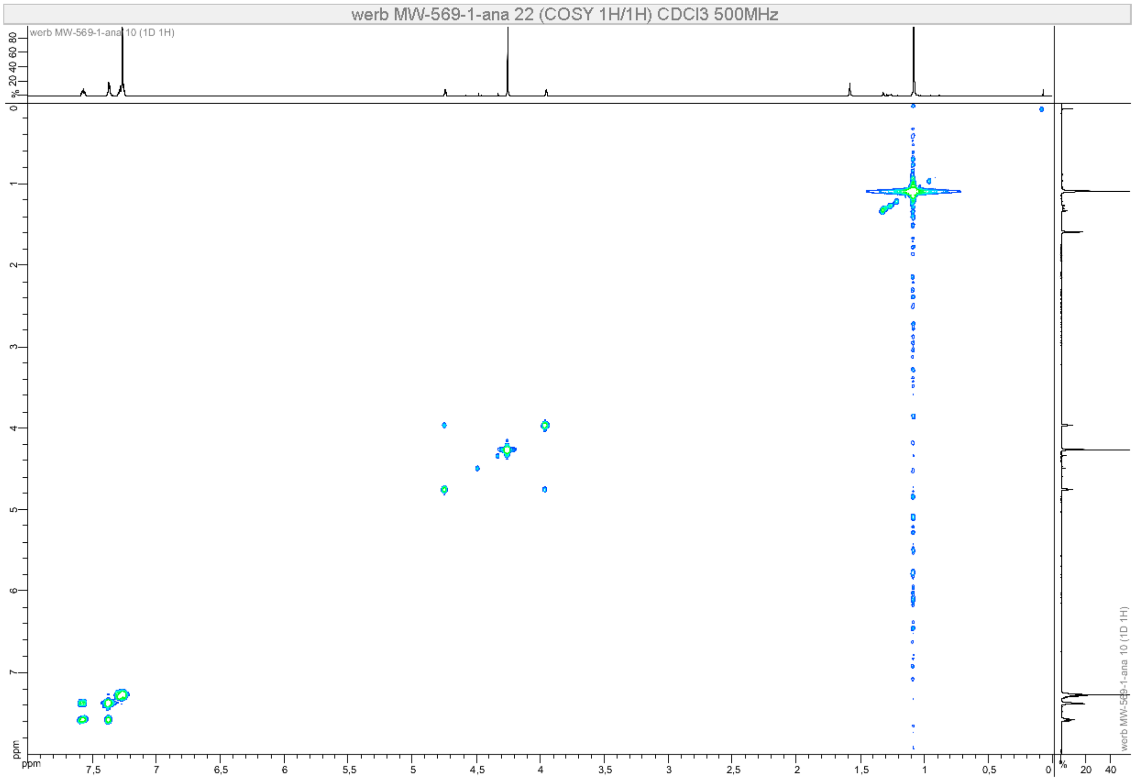

HSQC (500 MHz, $CDCl_3$)

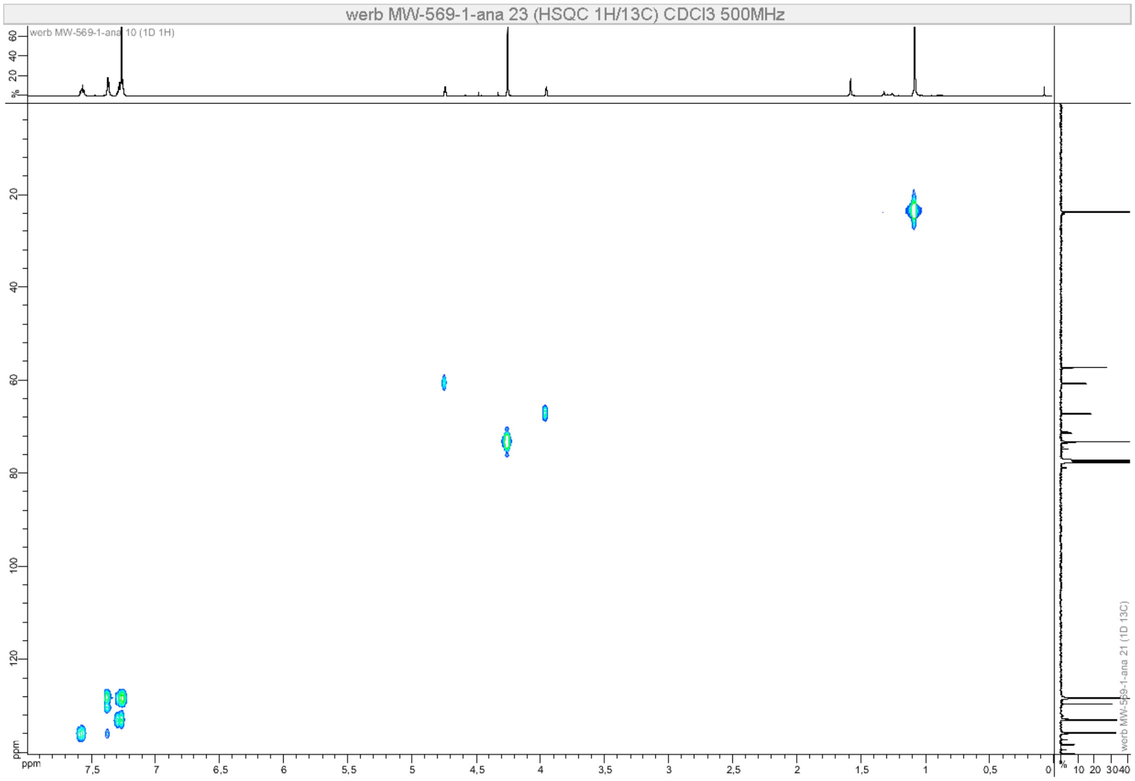

HMBC (500 MHz, $CDCl_3$)

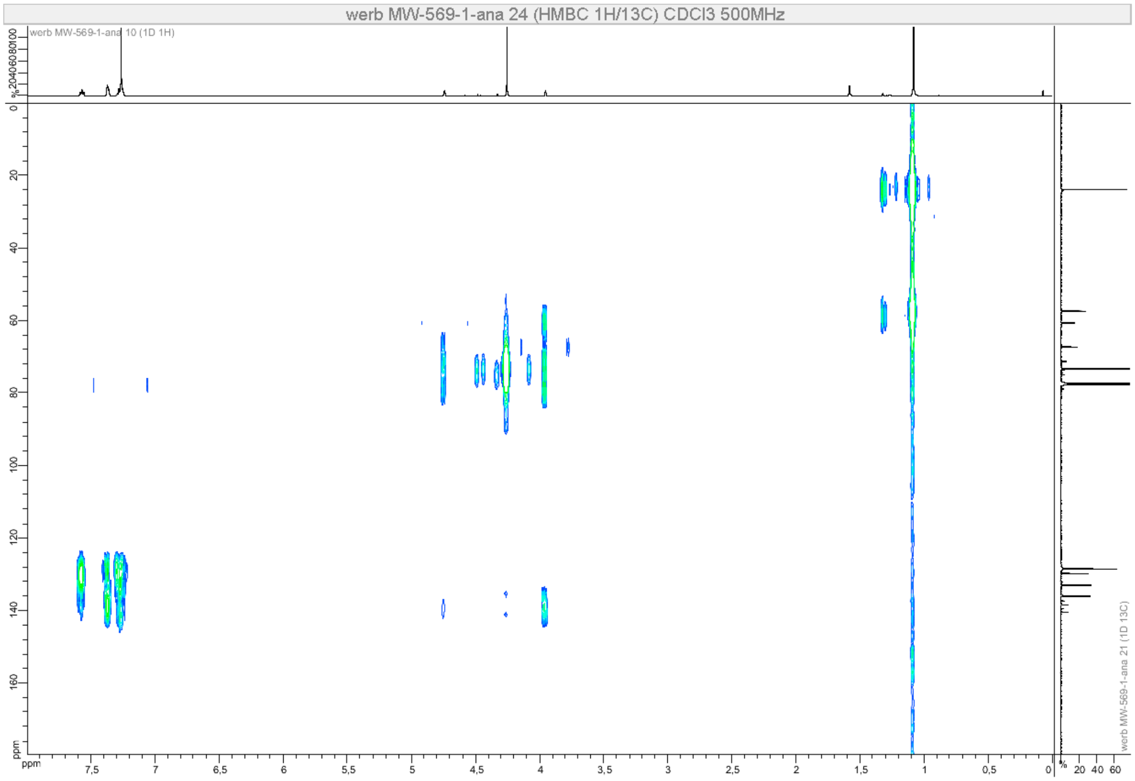

NOESY (500 MHz, $CDCl_3$)

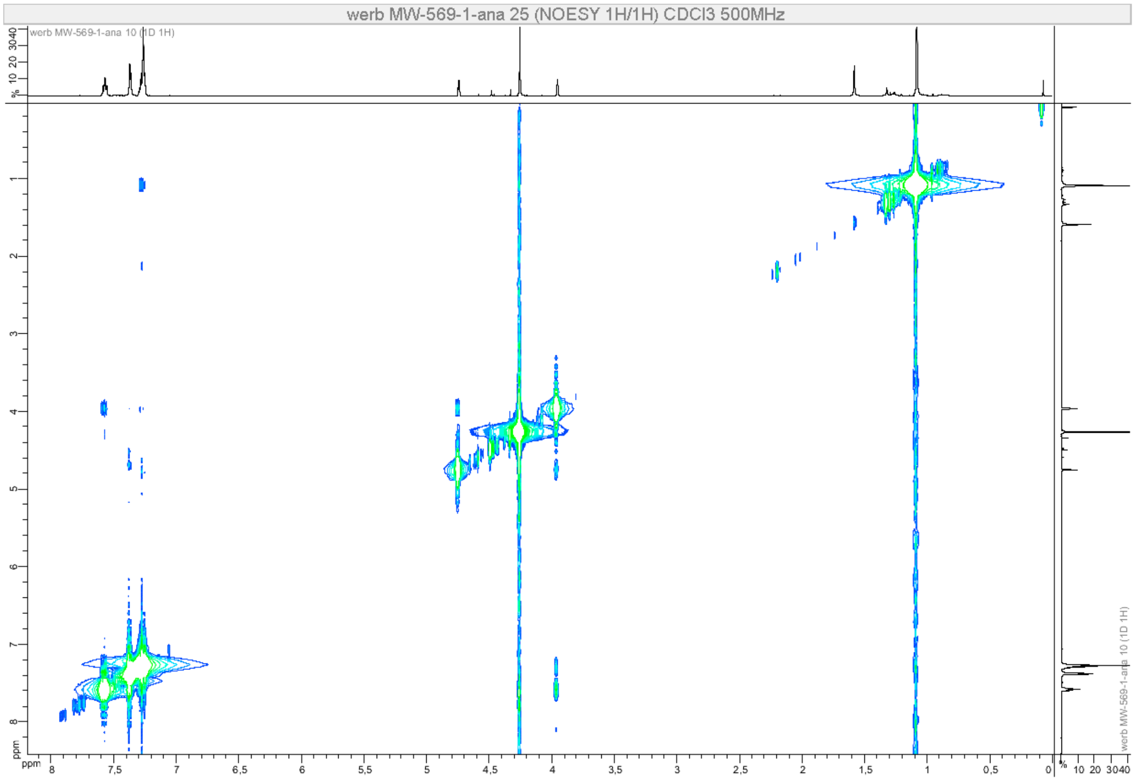

^{19}F NMR (471 MHz, $CDCl_3$)

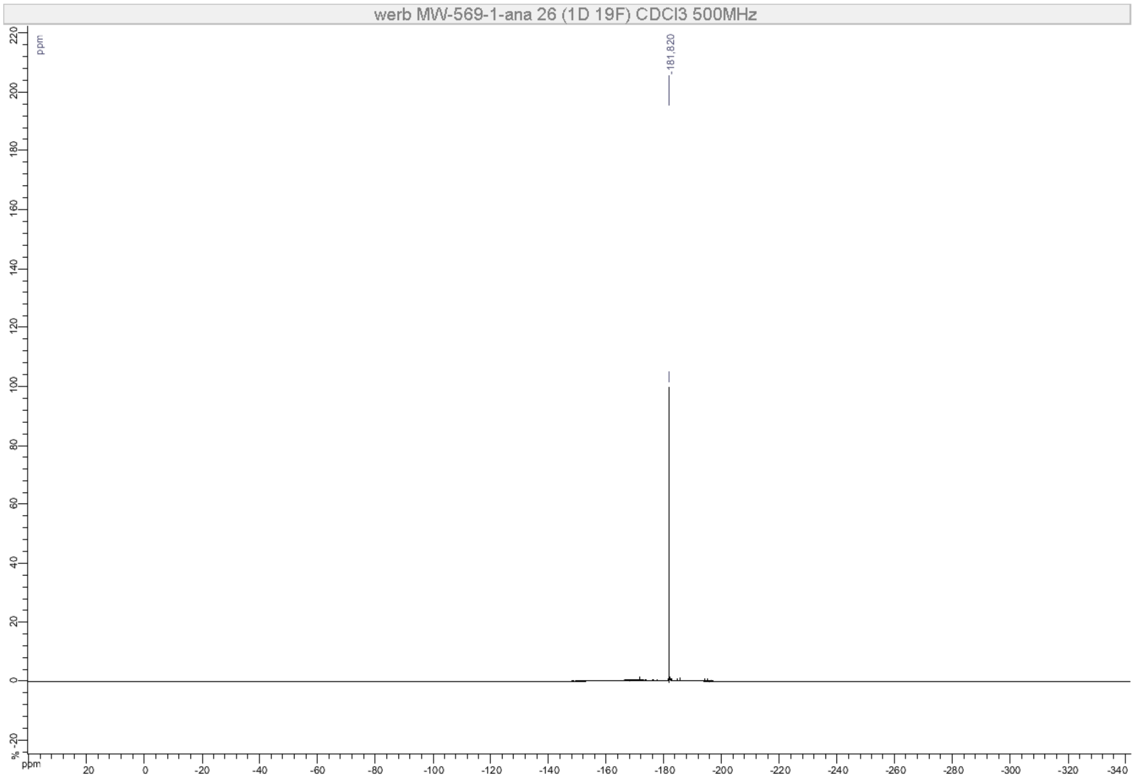

^{31}P NMR (202 MHz, $CDCl_3$)

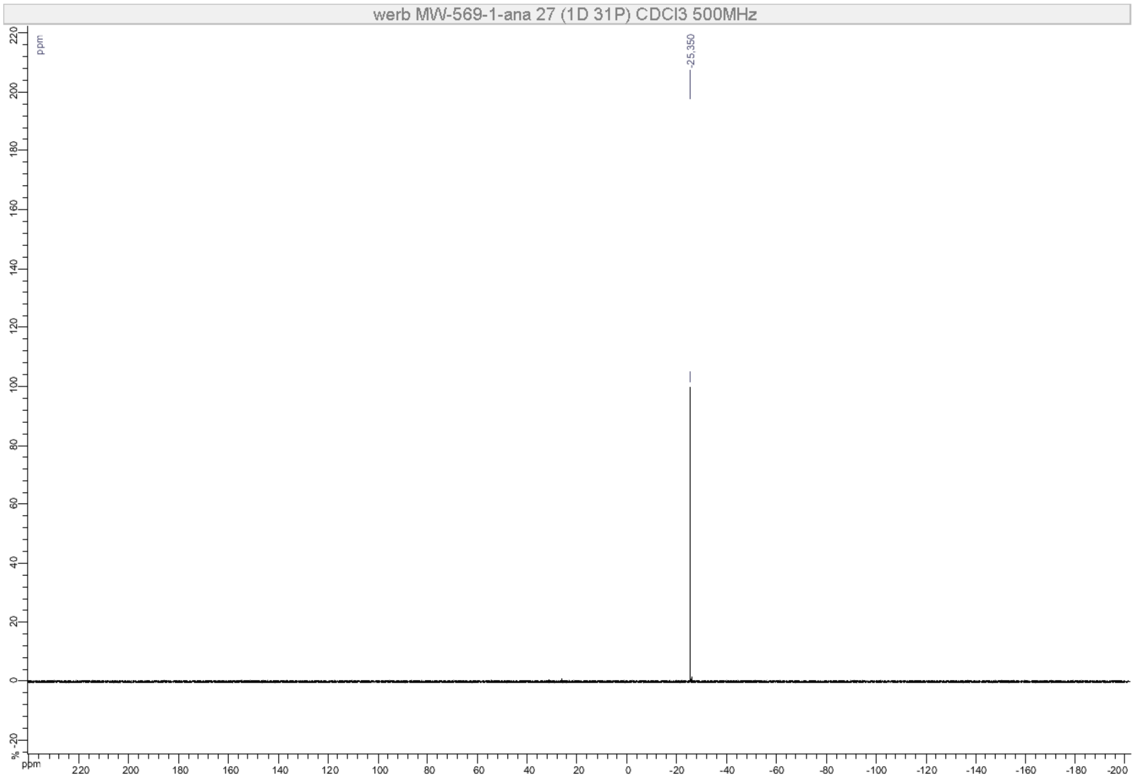

(*S*,*S*P)-*S*-*tert*-Butyl-2-fluoro-3-iodo-5-(trimethylsilyl)ferrocenesulfoxide (*S*,*S*P-9b)

^{1}H NMR (500 MHz, $CDCl_3$)

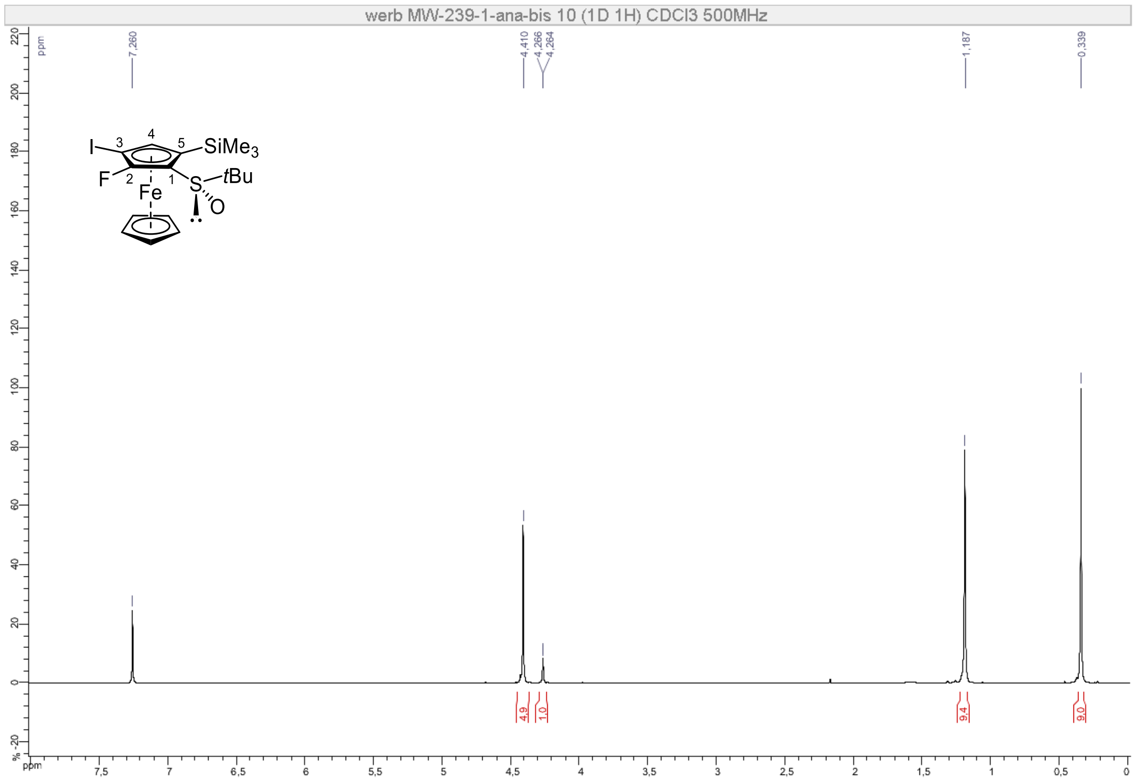

^{13}C NMR (126 MHz, $CDCl_3$)

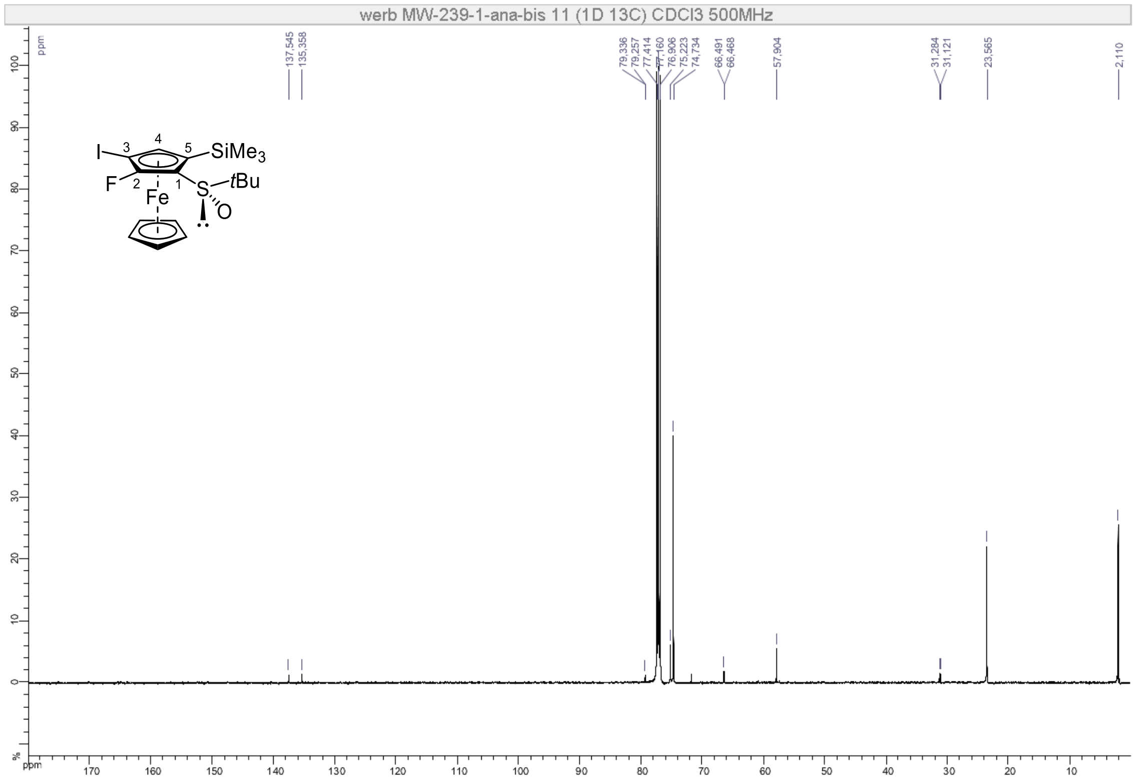

DEPT 135 (126 MHz, $CDCl_3$)

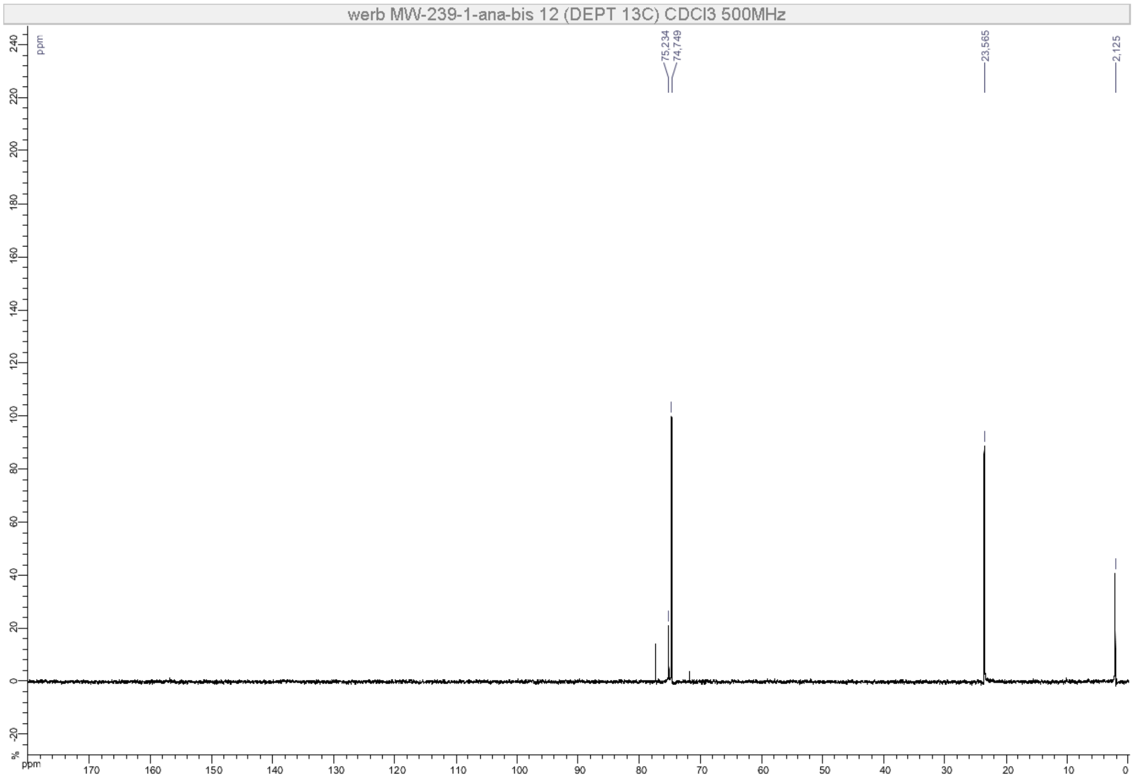

COSY (500 MHz, $CDCl_3$)

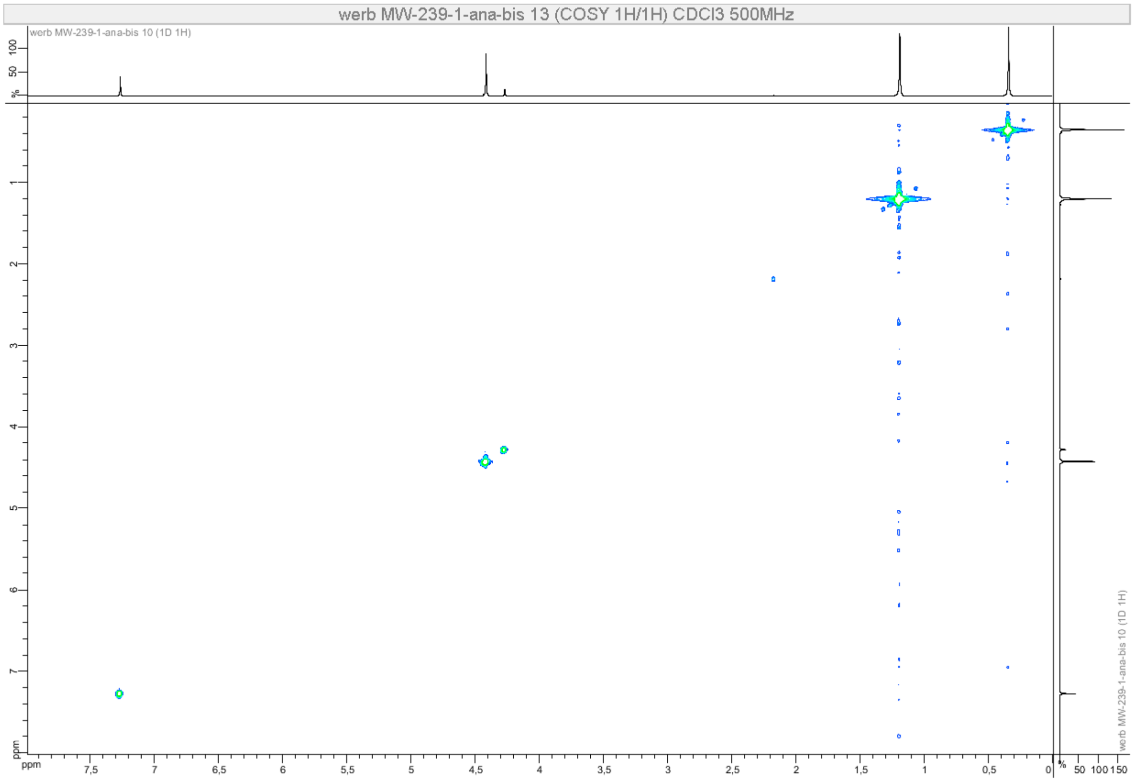

HSQC (500 MHz, $CDCl_3$)

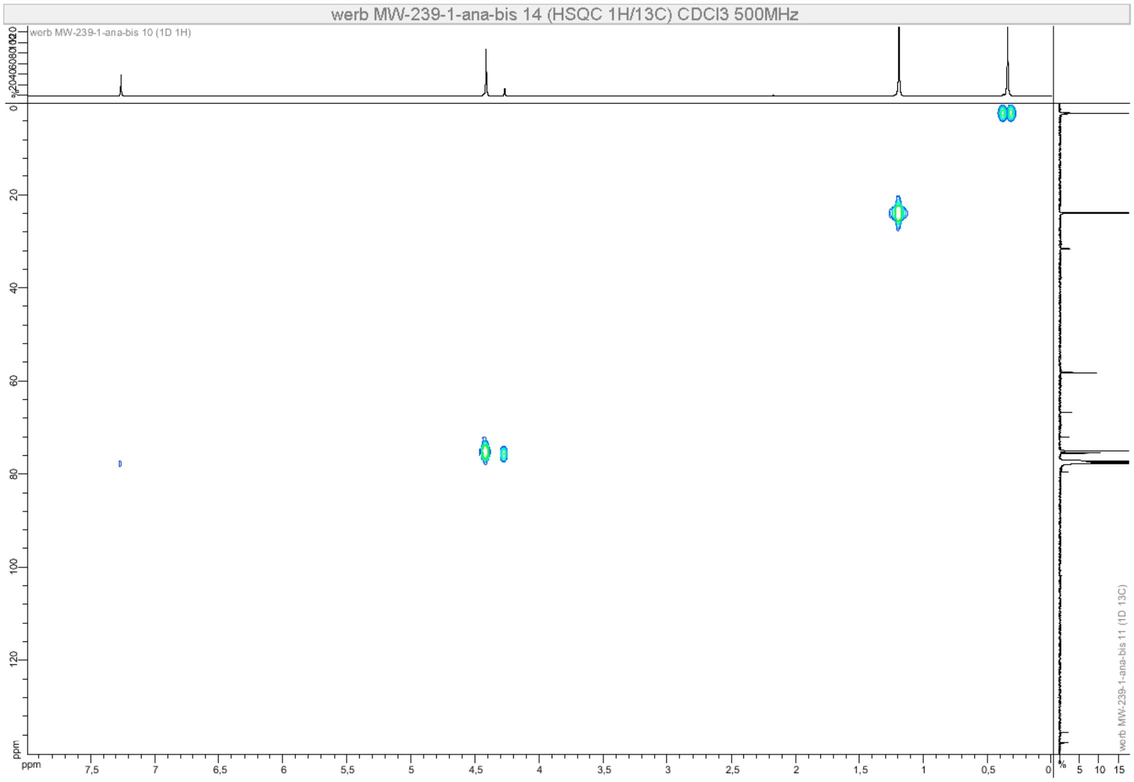

HMBC (500 MHz, $CDCl_3$)

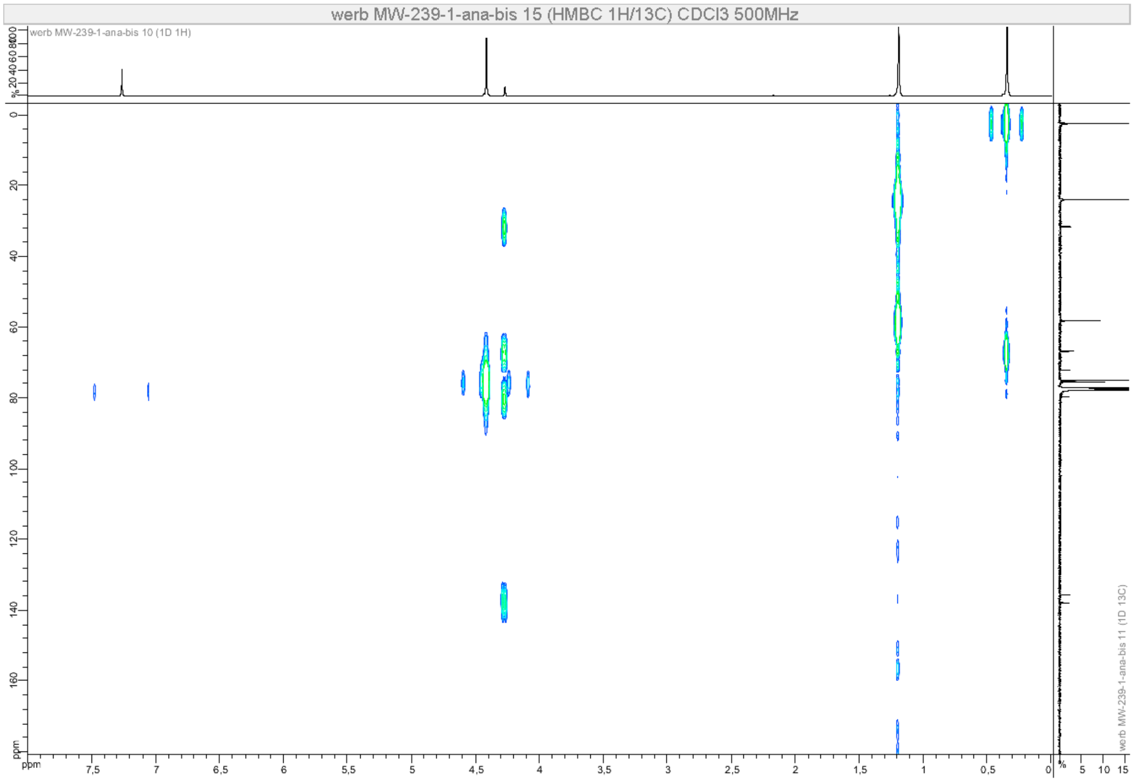

NOESY (500 MHz, $CDCl_3$)

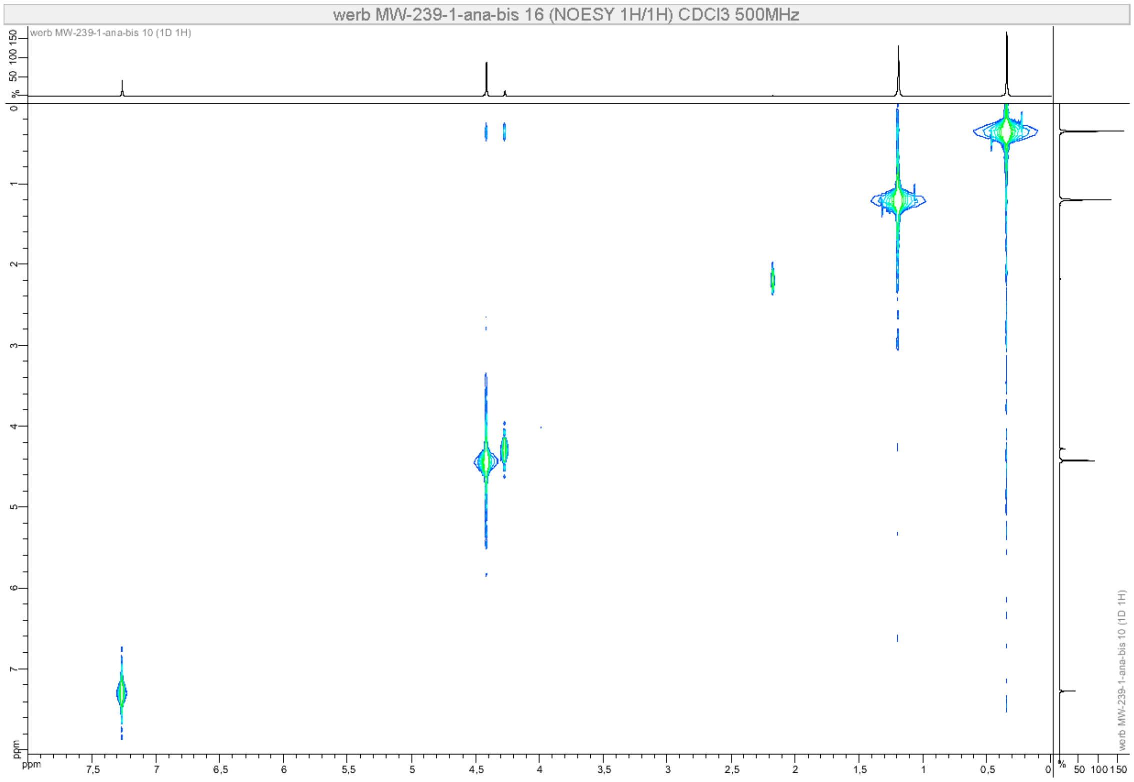

^{19}F NMR (471 MHz, $CDCl_3$)

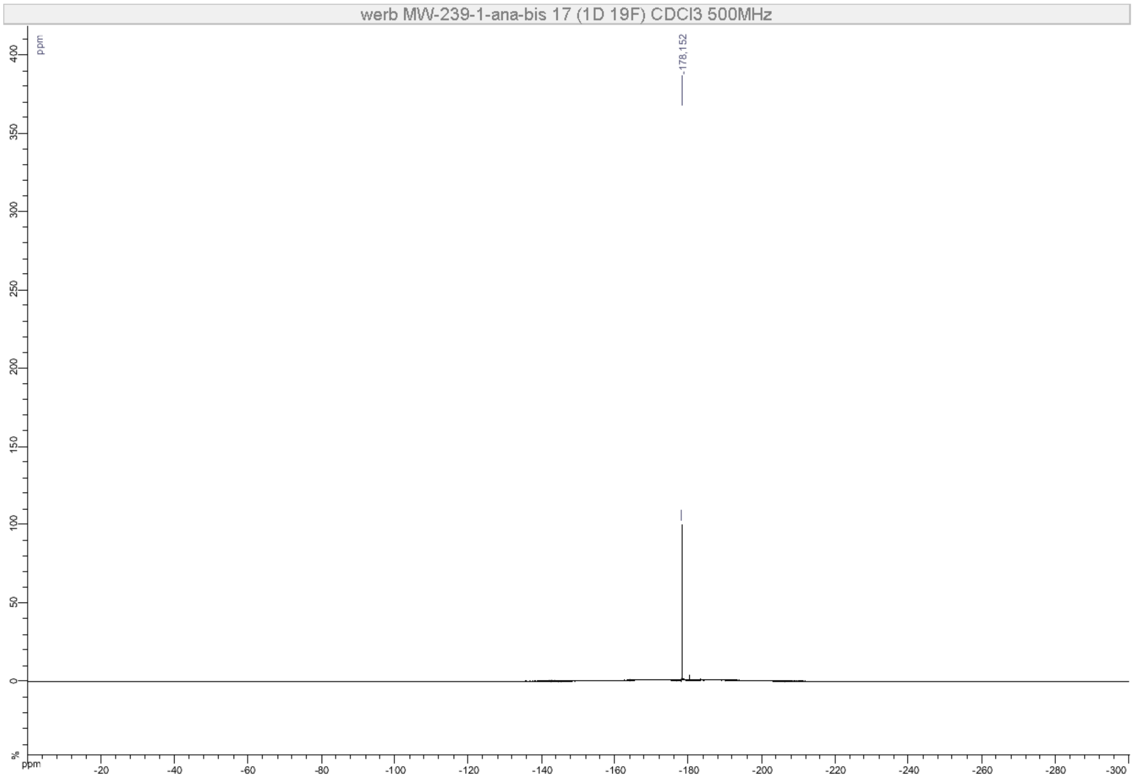

(*S*,*S*P)-*S*-*tert*-Butyl-2-fluoro-3-methyl-5-(trimethylsilyl)ferrocenesulfoxide (*S*,*S*P-9d)

^{1}H NMR (500 MHz, $CDCl_3$)

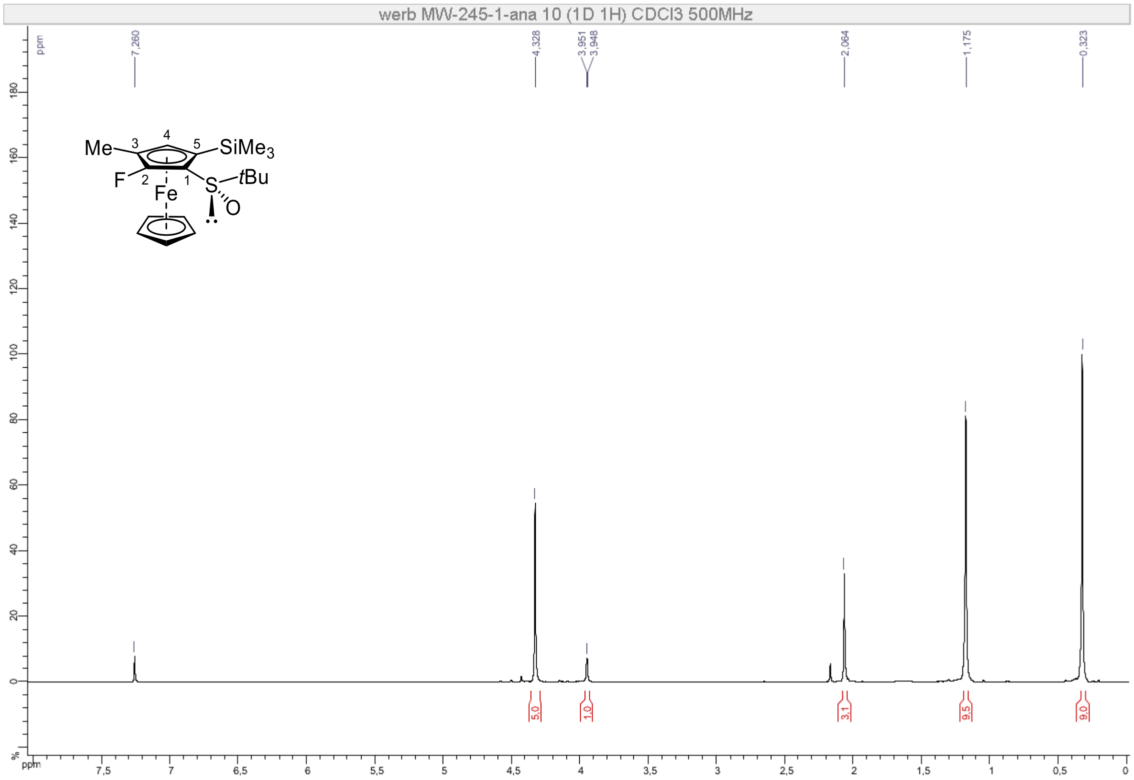

^{13}C NMR (126 MHz, $CDCl_3$)

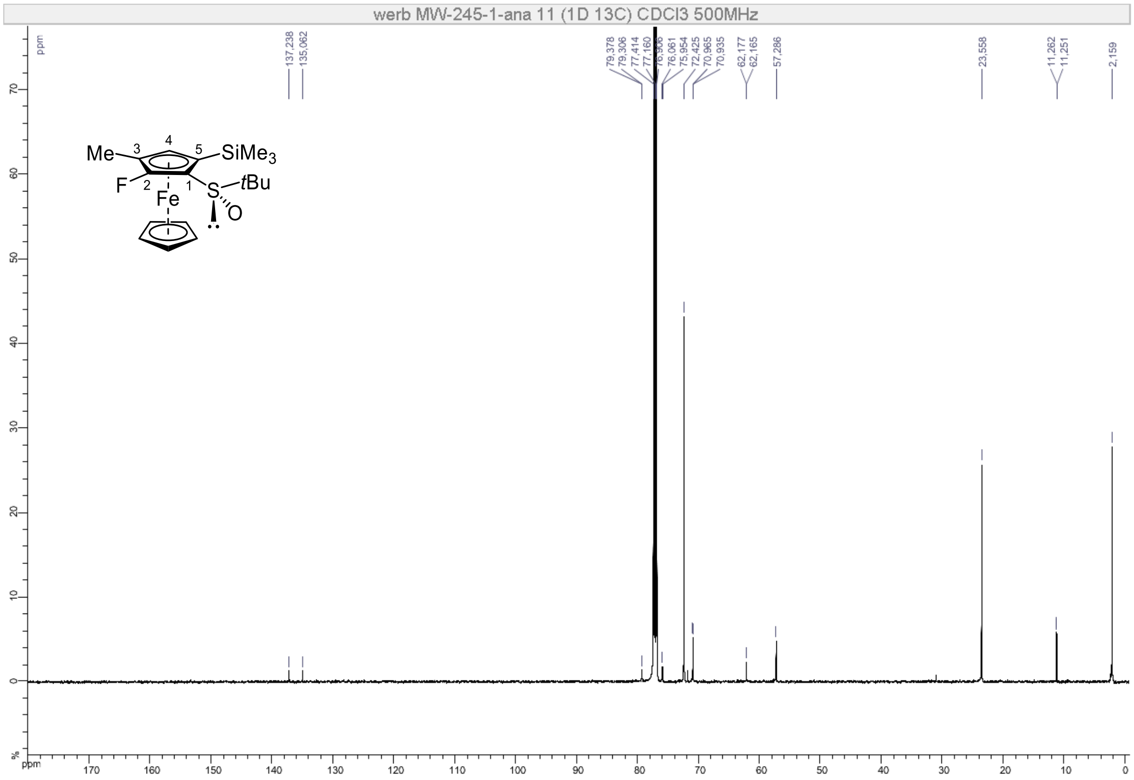

DEPT 135 (126 MHz, $CDCl_3$)

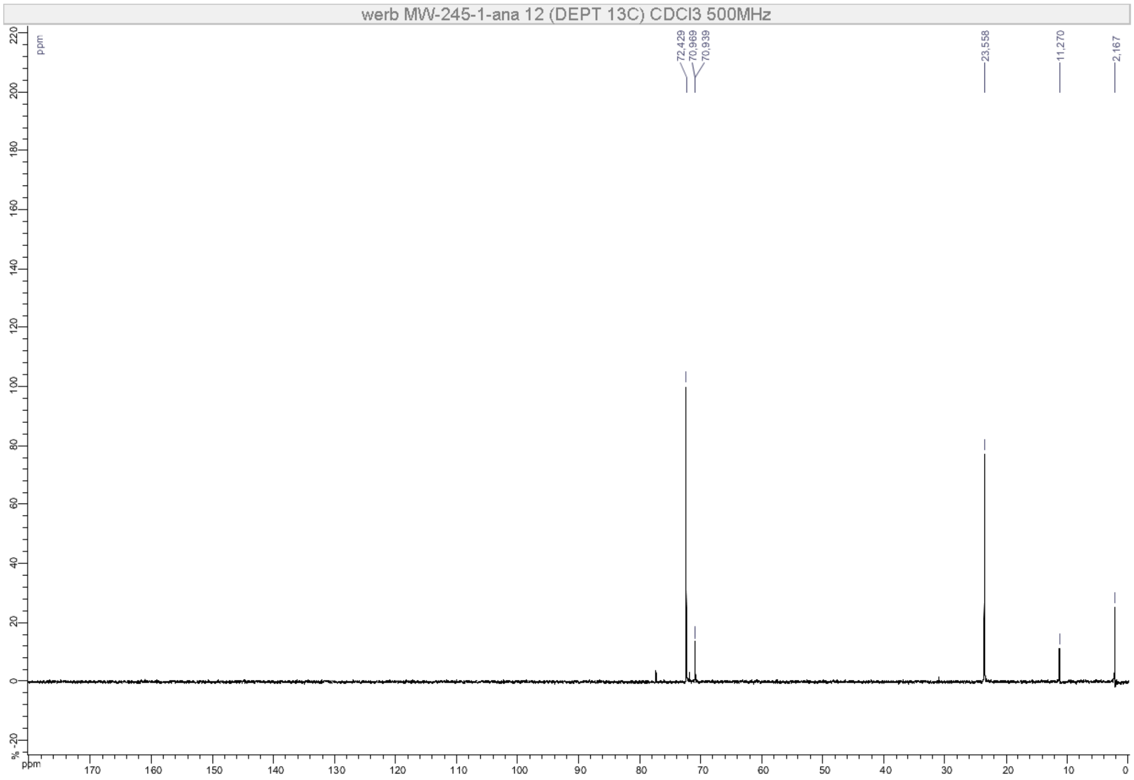

COSY (500 MHz, $CDCl_3$)

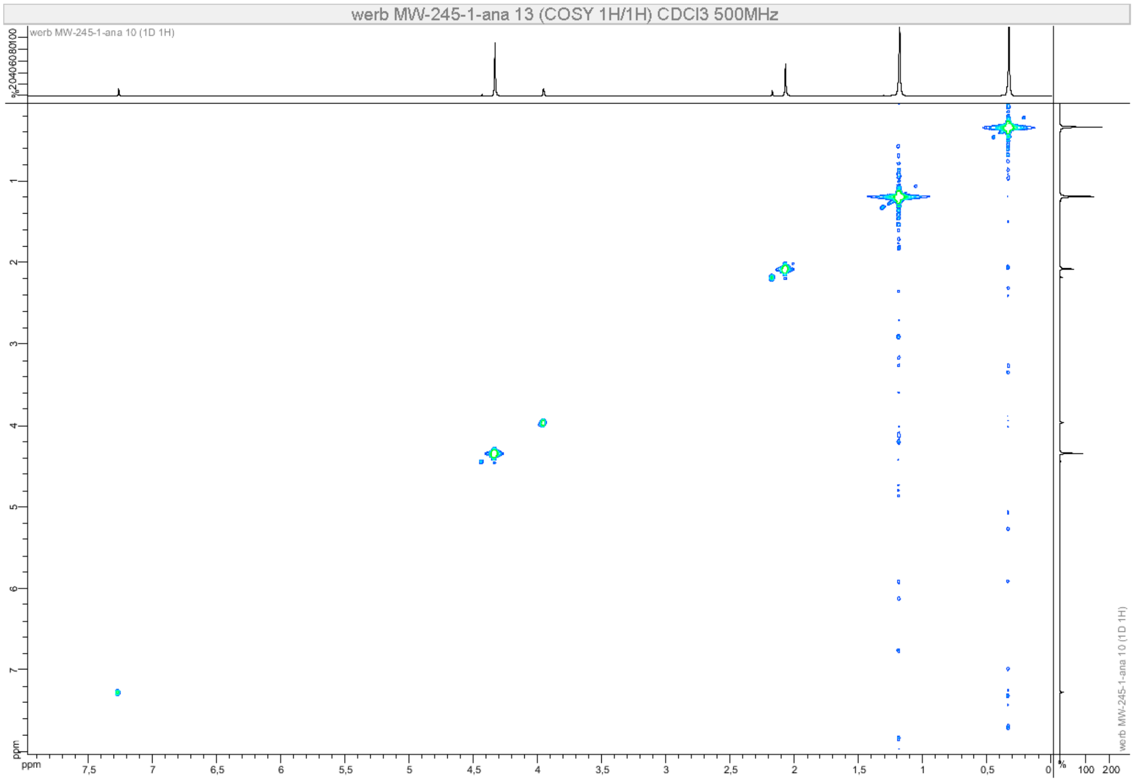

HSQC (500 MHz, $CDCl_3$)

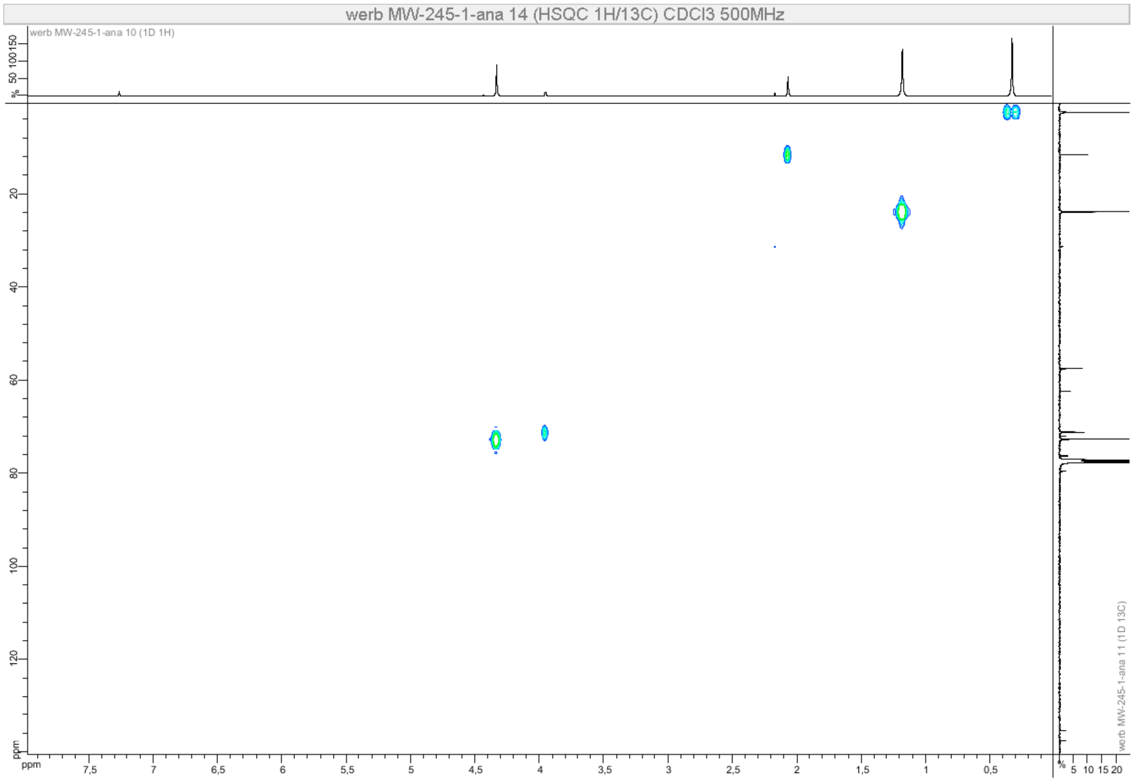

HMBC (500 MHz, $CDCl_3$)

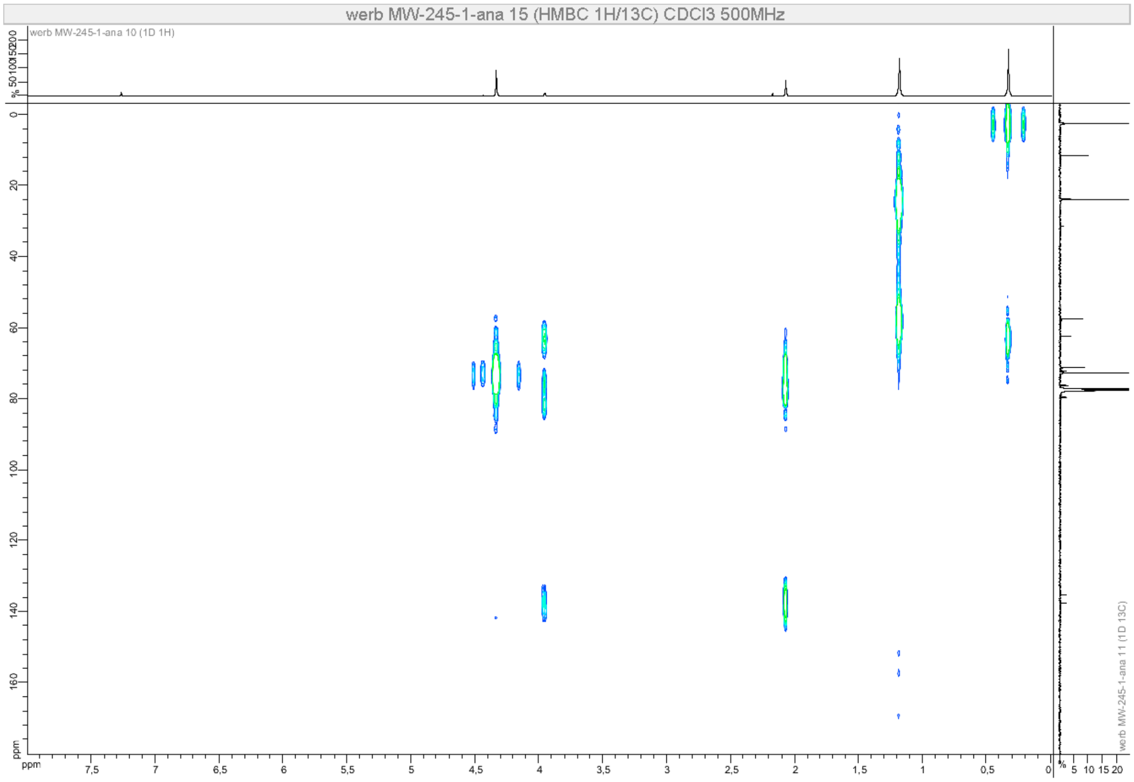

NOESY (500 MHz, $CDCl_3$)

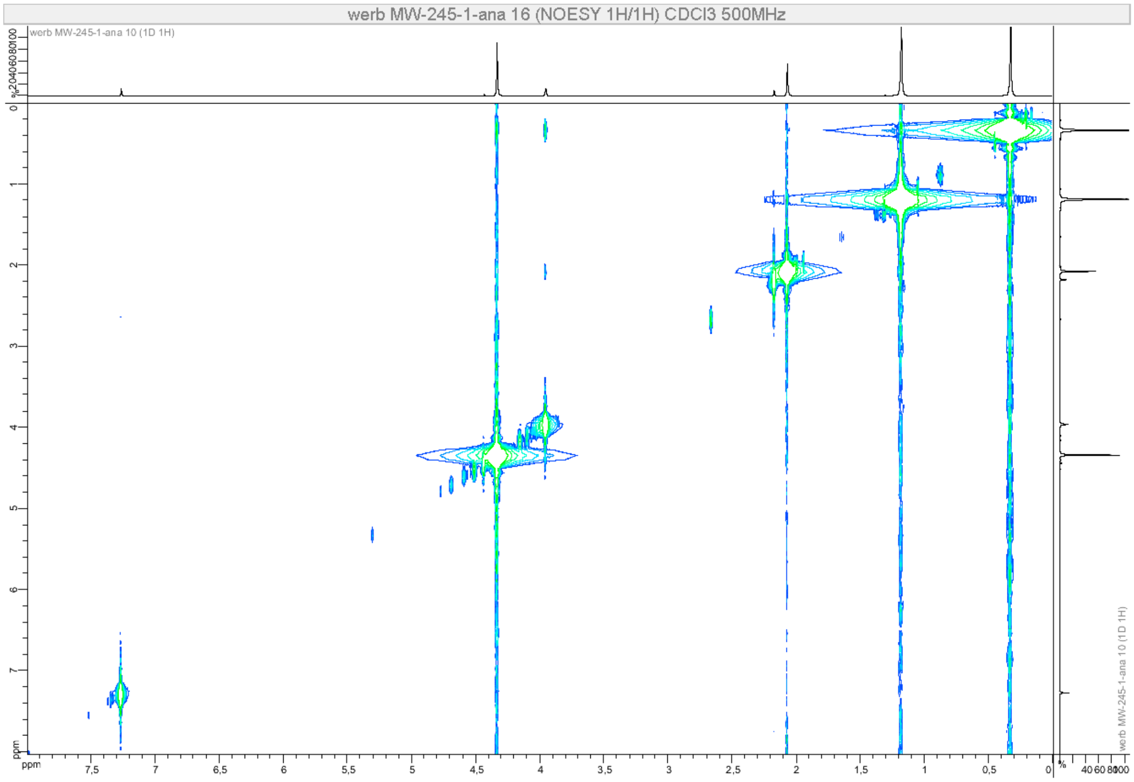

^{19}F NMR (471 MHz, $CDCl_3$)

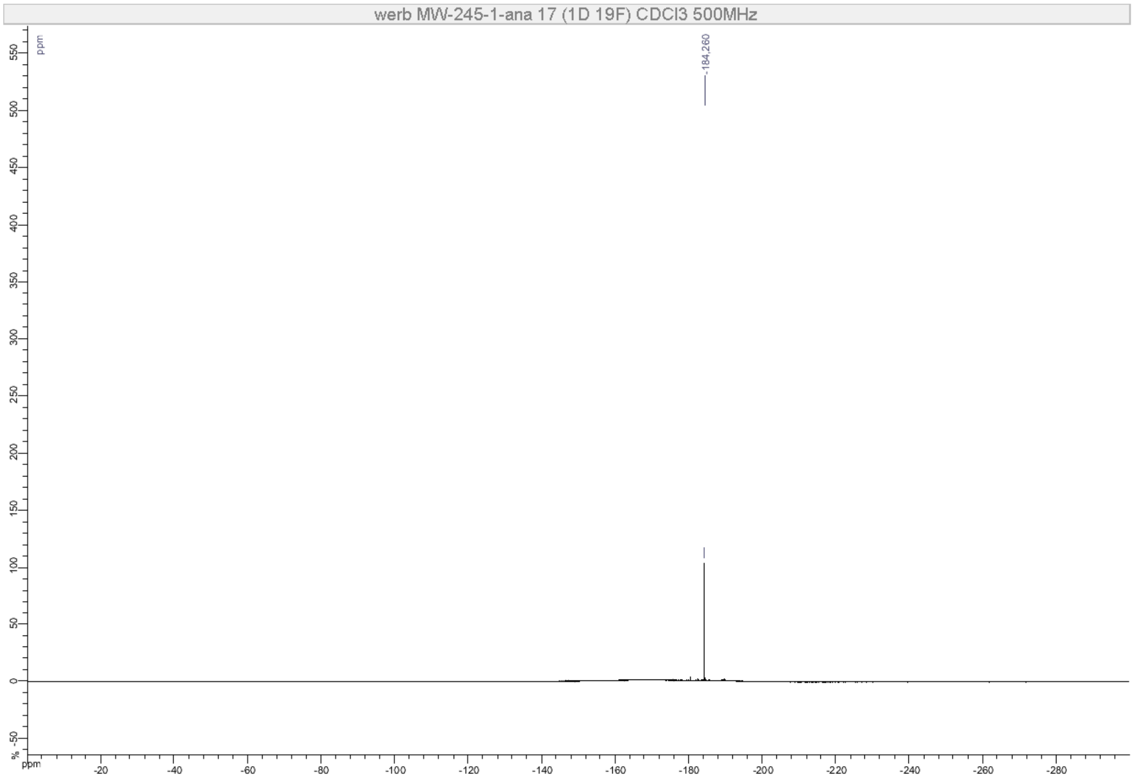

(*S*,*R*P)-*S*-*tert*-Butyl-2-fluoro-3-methylferrocenesulfoxide (*S*,*R*P-10)

^{1}H NMR (300 MHz, $CDCl_3$)

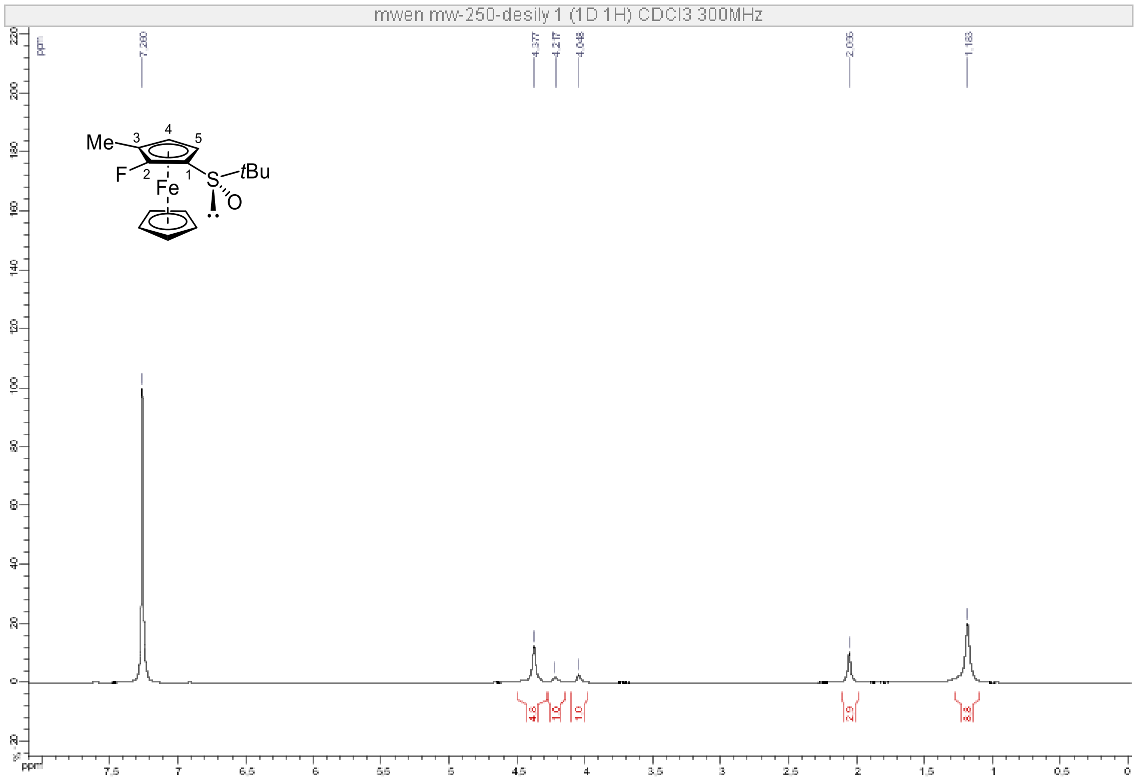

(*S*,*R*P)-*S*-*tert*-Butyl-2-fluoro-5-iodo-3-methylferrocenesulfoxide (*S*,*R*P-11b)

^{1}H NMR (300 MHz, $CDCl_3$)

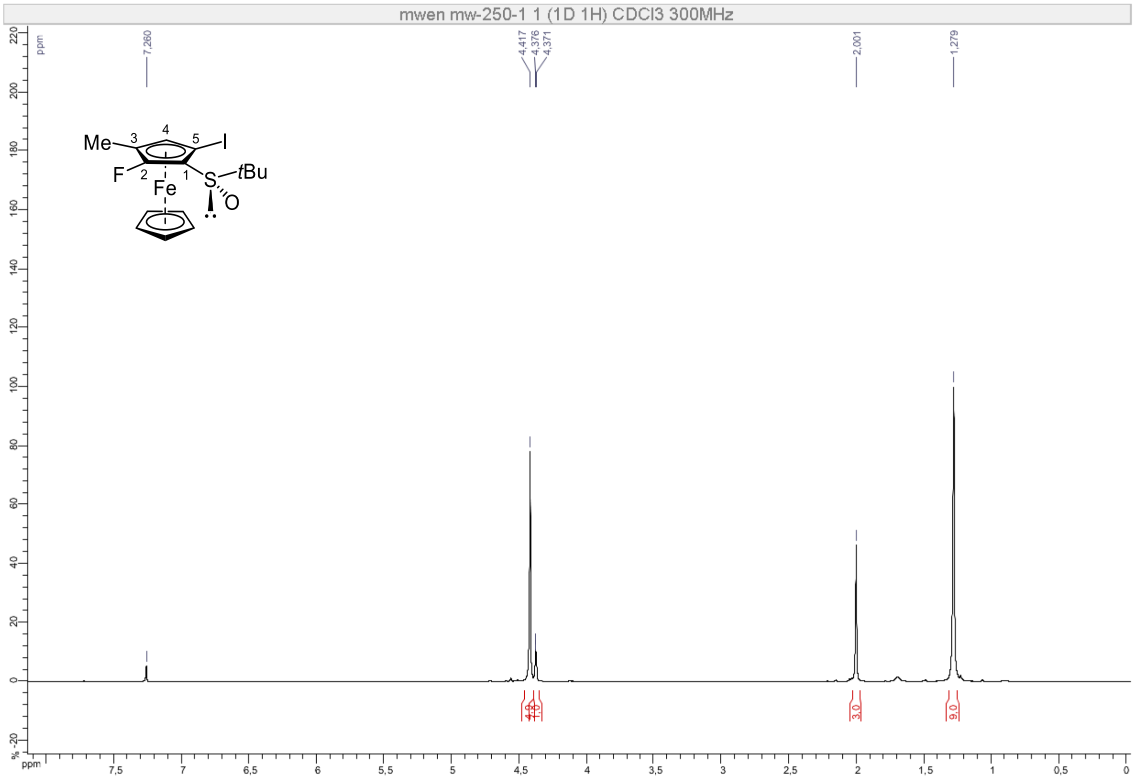

(*S*,*R*P)-*S*-*tert*-Butyl-5-chloro-2-fluoro-3-methylferrocenesulfoxide (*S*,*R*P-11k)

^{1}H NMR (500 MHz, CDCl$_3$)

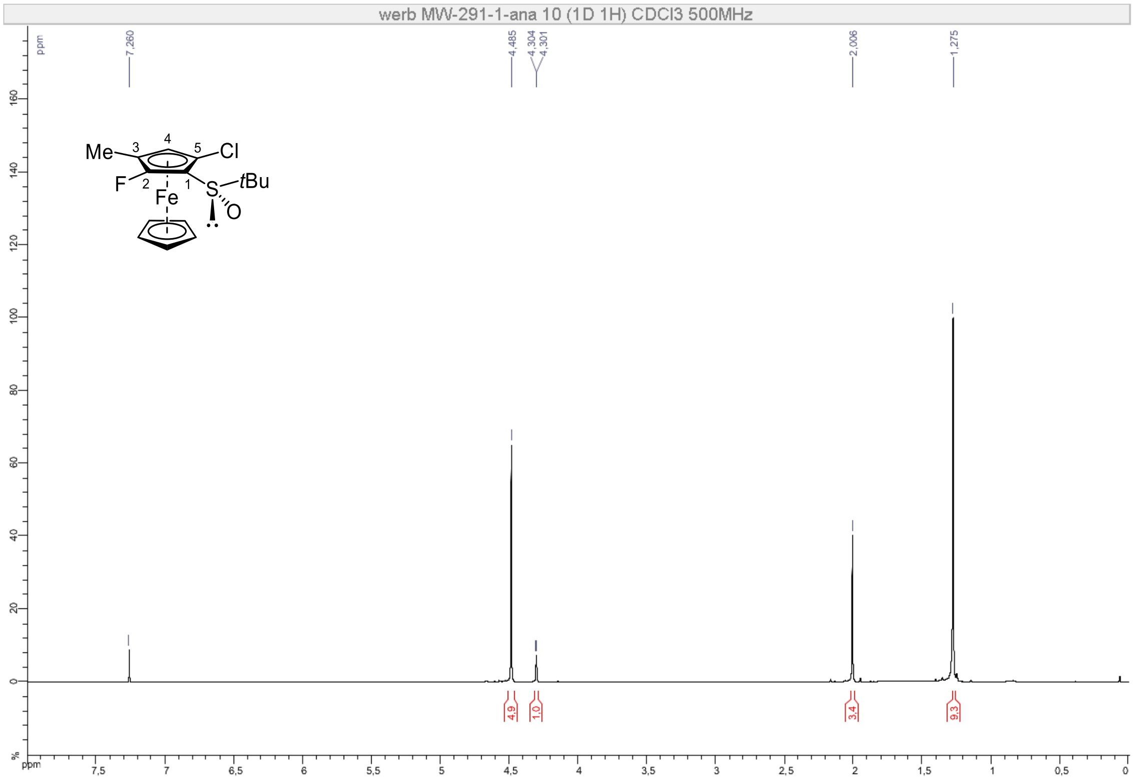

^{13}C NMR (126 MHz, CDCl$_3$)

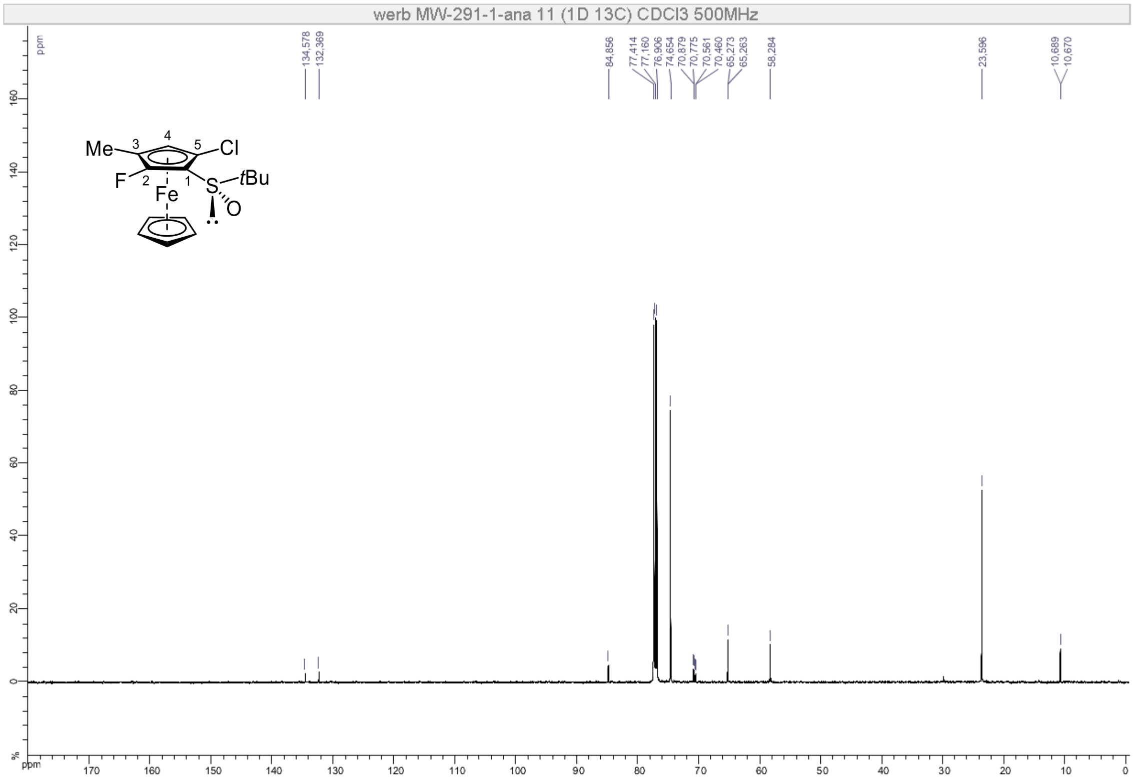

HSQC (500 MHz, $CDCl_3$)

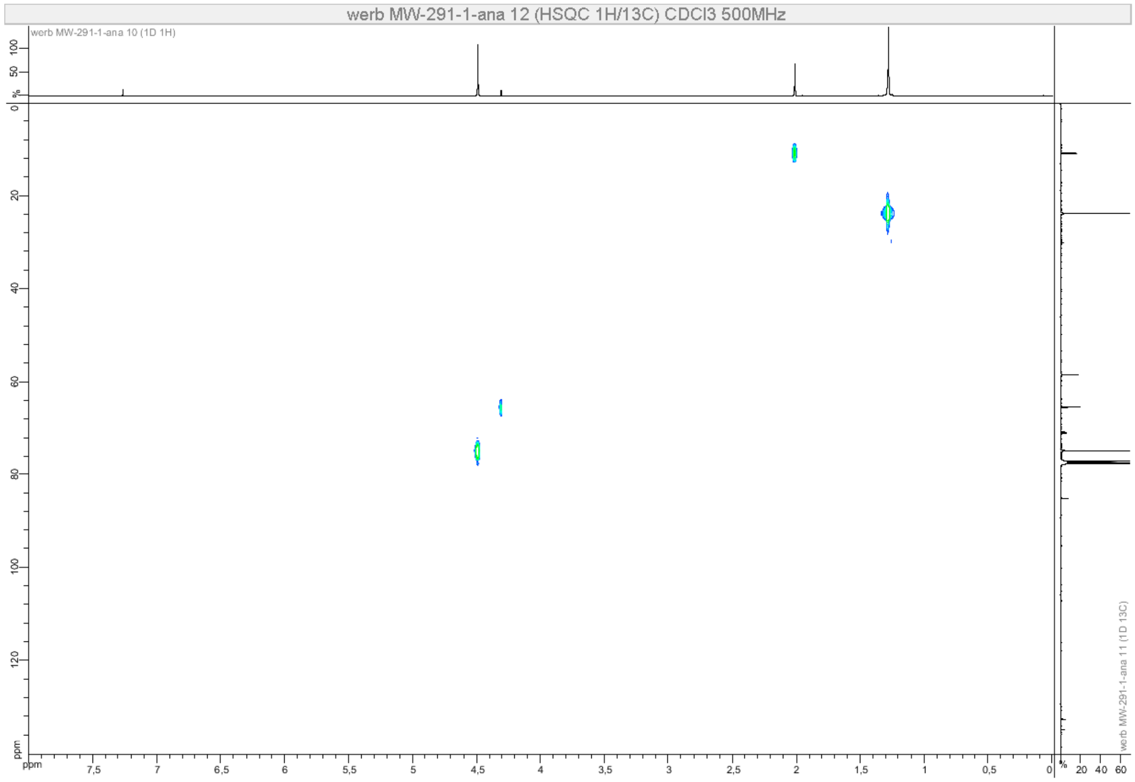

HMBC (500 MHz, $CDCl_3$)

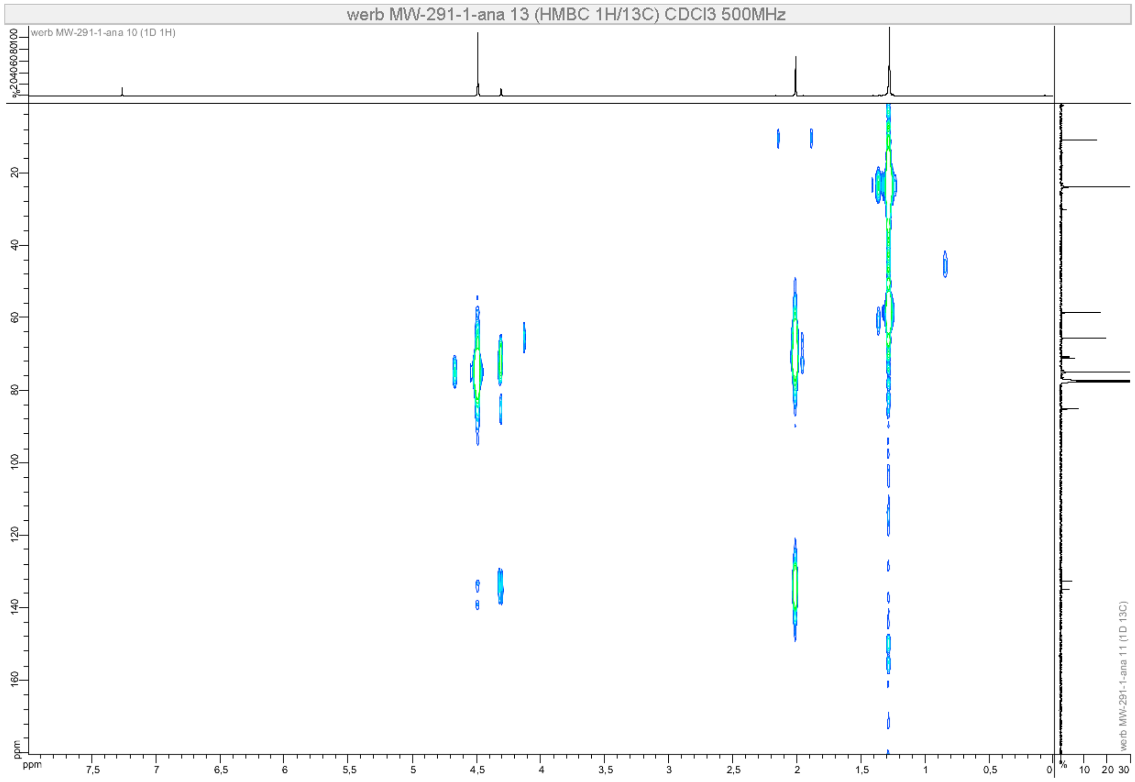

^{19}F NMR (471 MHz, $CDCl_3$)

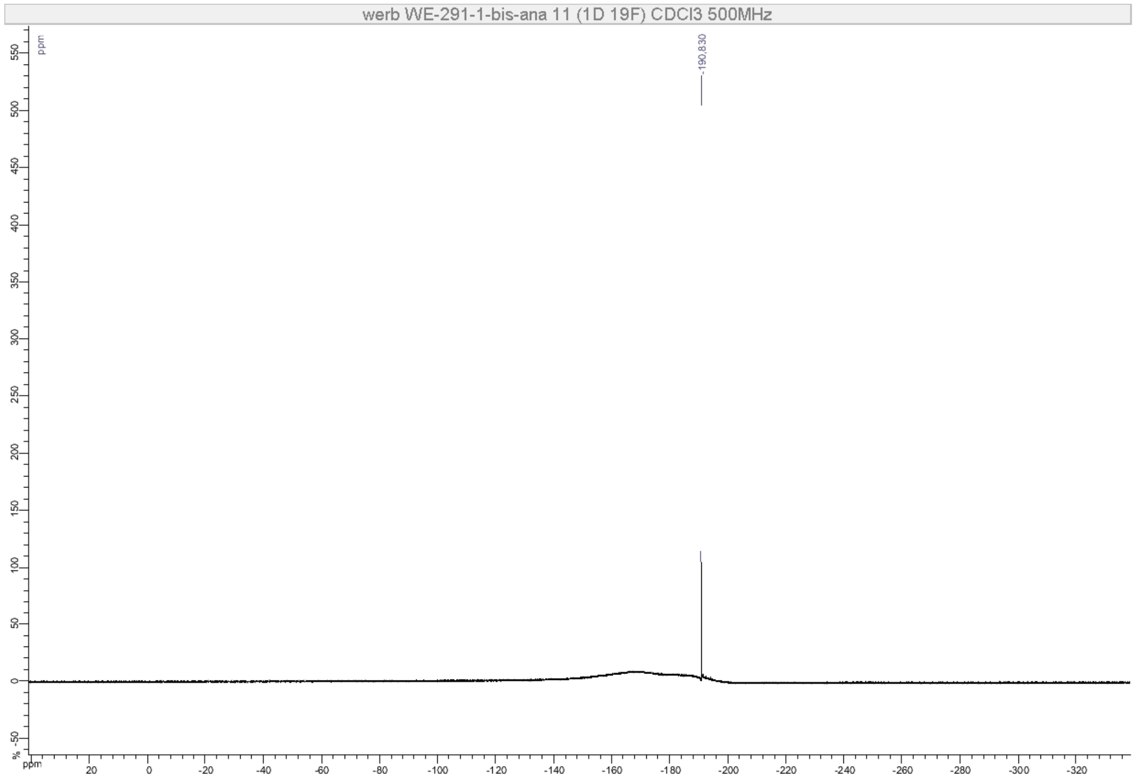

Selected NOESY correlations

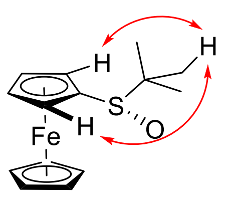	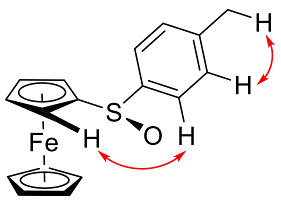	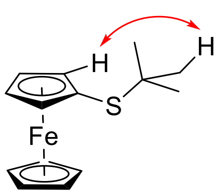
***S*-FcSO*t*Bu**	**R-FcSO-*p*-tol**	**(*tert*-Butylthio)ferrocene**
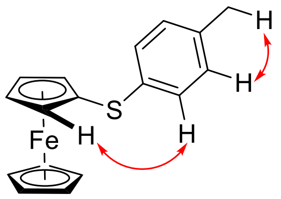	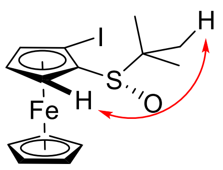	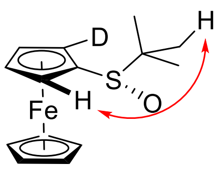
**(4-Tolylthio)ferrocene**	**S,R_P-1b**	**S,S_P-1c**
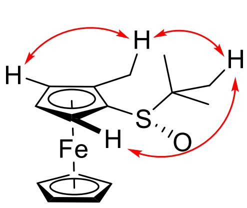	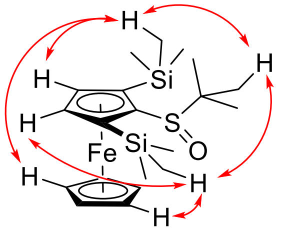	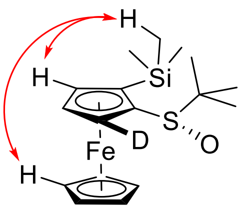
**S,S_P-1d**	***rac*-2aa**	**S,S_P-2ac**
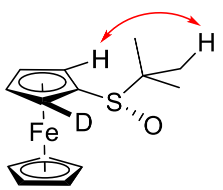	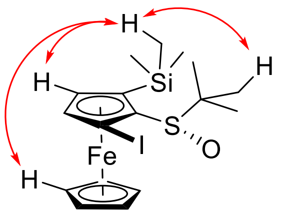	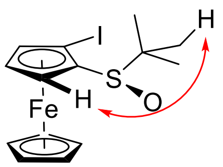
**S,R_P-1c**	**R,R_P-2ab**	**R,R_P-1b**
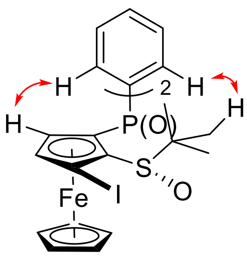	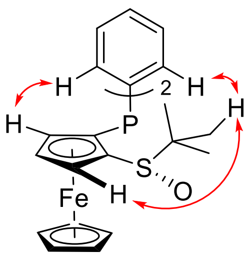	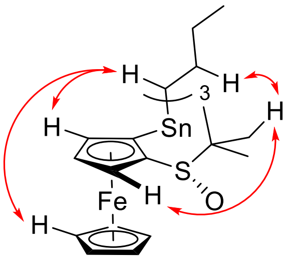
**S,S_P-2eb**	**S,S_P-1e**	**S,R_P-1f**

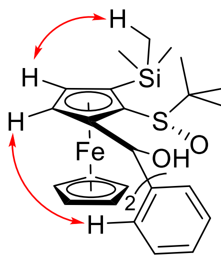	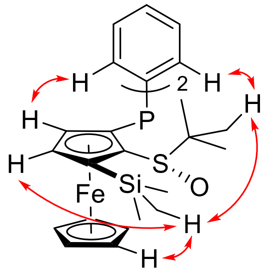	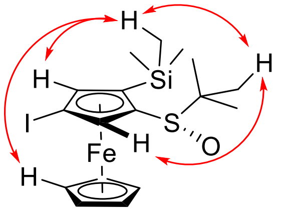
**S,S_{P}-2ag**	**S,S_{P}-2ea**	**S,S_{P}-7**
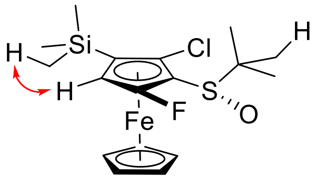	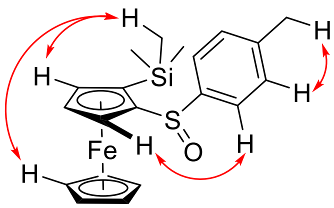	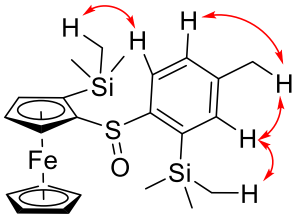
**S,S_{P}-12**	**$R,R_{P}/S,S_{P}$-3a**	**$R,R_{P}/S,S_{P}$-3a’** (*)
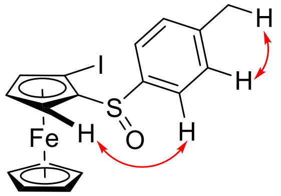	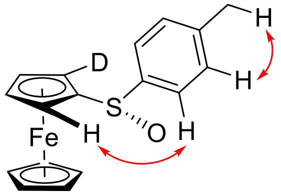	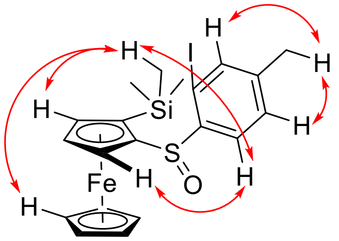
**$R,S_{P}/S,R_{P}$-3b**	**S,S_{P}-3c**	**$R,R_{P}/S,S_{P}$-4**
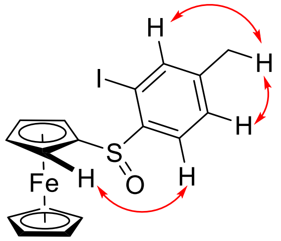	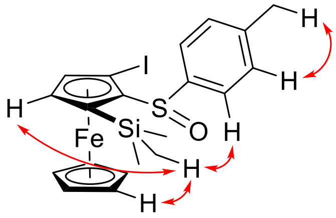	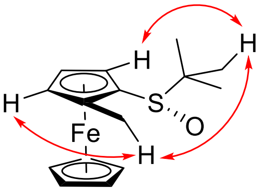
***rac*-5**	**$R,S_{P}/S,R_{P}$-6**	**S,R_{P}-1d**
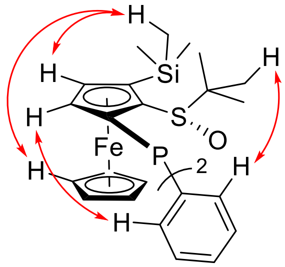	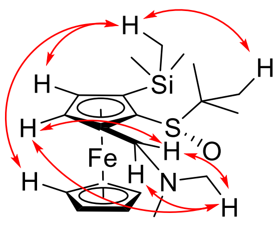	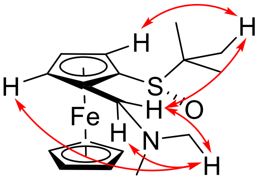
**S,R_{P}-2ae**	**S,S_{P}-2ah**	**S,R_{P}-1h**

(*) Not resolved enough for attribution.

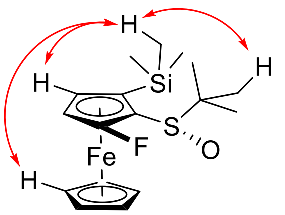	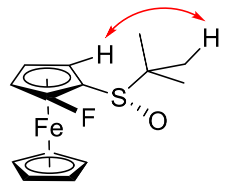	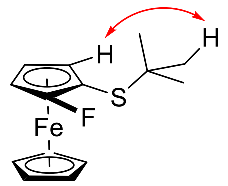
**S,S_{P}-2ai**	**S,R_{P}-1i**	**R_{P}-8i**
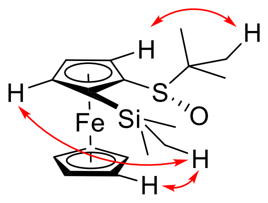	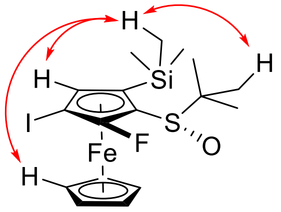	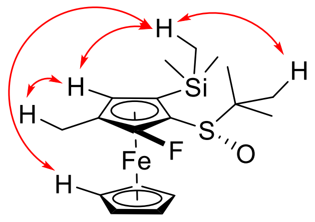
**S,R_{P}-1a**	**S,S_{P}-9b**	**S,S_{P}-9d**

Calculated values of the Gibbs energies ΔG_{acid} [kcal·mo1^{-1}] for deprotonation

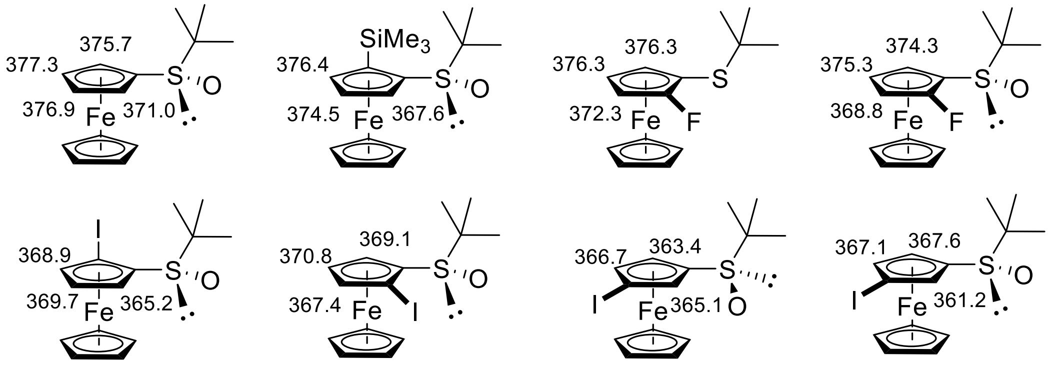

Supplement: Supplementary file 1 [file molecules-27-01798-s001.zip › SM_sulfoxides 2.pdf]
